# An Unprecedented Number of Cytochrome P450s Are Involved in Secondary Metabolism in *Salinispora* Species

**DOI:** 10.3390/microorganisms10050871

**Published:** 2022-04-21

**Authors:** Nsikelelo Allison Malinga, Nomfundo Nzuza, Tiara Padayachee, Puleng Rosinah Syed, Rajshekhar Karpoormath, Dominik Gront, David R. Nelson, Khajamohiddin Syed

**Affiliations:** 1 Department of Biochemistry and Microbiology, Faculty of Science and Agriculture, University of Zululand, KwaDlangezwa 3886, South Africa; nsikelelo.malinga@gmail.com (N.A.M.); nomfundonzuza11@gmail.com (N.N.); teez07padayachee@gmail.com (T.P.); 2 Department of Pharmaceutical Chemistry, College of Health Sciences, University of KwaZulu-Natal, Durban 4000, South Africa; prosinah@gmail.com (P.R.S.); karpoormath@ukzn.ac.za (R.K.); 3 Faculty of Chemistry, Biological and Chemical Research Centre, University of Warsaw, Pasteura 1, 02-093 Warsaw, Poland; dgront@gmail.com; 4 Department of Microbiology, Immunology and Biochemistry, University of Tennessee Health Science Center, Memphis, TN 38163, USA

**Keywords:** natural products, secondary metabolites, actinomycete, marine, *Salinispora arenicola*, cytochrome P450, biosynthetic gene clusters, genome-data mining, diversity, *Streptomyces*, *Mycobacterium*

## Abstract

Cytochrome P450 monooxygenases (CYPs/P450s) are heme thiolate proteins present in species across the biological kingdoms. By virtue of their broad substrate promiscuity and regio- and stereo-selectivity, these enzymes enhance or attribute diversity to secondary metabolites. Actinomycetes species are well-known producers of secondary metabolites, especially *Salinispora* species. Despite the importance of P450s, a comprehensive comparative analysis of P450s and their role in secondary metabolism in *Salinispora* species is not reported. We therefore analyzed P450s in 126 strains from three different species *Salinispora arenicola*, *S. pacifica,* and *S. tropica*. The study revealed the presence of 2643 P450s that can be grouped into 45 families and 103 subfamilies. CYP107 and CYP125 families are conserved, and CYP105 and CYP107 families are bloomed (a P450 family with many members) across *Salinispora* species. Analysis of P450s that are part of secondary metabolite biosynthetic gene clusters (smBGCs) revealed *Salinispora* species have an unprecedented number of P450s (1236 P450s-47%) part of smBGCs compared to other bacterial species belonging to the genera *Streptomyces* (23%) and *Mycobacterium* (11%), phyla *Cyanobacteria* (8%) and *Firmicutes* (18%) and the classes *Alphaproteobacteria* (2%) and *Gammaproteobacteria* (18%). A peculiar characteristic of up to six P450s in smBGCs was observed in *Salinispora* species. Future characterization *Salinispora* species P450s and their smBGCs have the potential for discovering novel secondary metabolites.

## 1. Introduction

Cytochrome P450 monooxygenases (CYPs/P450s) comprise a superfamily of heme-thiolate proteins. P450s are present in all species of different biological kingdoms, including in viruses considered non-living entities [1,2]. This suggests that these enzymes play an important role in species’ primary and secondary metabolism. These enzymes were initially identified as monooxygenases due to their ability to introduce one oxygen atom into a substrate [3]. Subsequent research revealed that P450s are catalytically diverse enzymes performing some unusual enzymatic reactions [4,5,6,7,8]. The regio- and stereo-specific oxidation of many substrates by P450s caught the attention of researchers for biotechnological exploration of these enzymes [9,10,11,12]. P450s reactions are essential in designing drugs such that drug toxicity of prodrugs is primarily assessed against these enzymes [13]. Also, P450s play a vital role in xenobiotic compounds’ detoxification [14]. Microbial P450s, especially from lower eukaryotes such as fungal CYP51, have been used as an azole drug target [15,16]. The study also suggested that fungal CYP53 can act as a potential alternative drug target [17]. One of the best examples of P450s biotechnological applications includes the synthesis of antibiotics and anticancer drugs [18,19,20,21].

The utilization of P450s in the generation of secondary metabolites or natural products, organic compounds not directly involved in an organism’s normal growth, development, or reproduction, is gaining momentum as reactions catalyzed by these enzymes contribute to the secondary metabolite diversity [22,23]. Secondary metabolites, their structural diversity, bioactivity, and ecological functions, including their application in almost all areas of biology, have been thoroughly reviewed [24,25,26,27,28,29]. For example, secondary metabolites are widely used in human and veterinary medicine, agriculture, and manufacturing [30]. 

Secondary metabolites in organisms are produced by a set of genes usually located next to each other as a cluster known as secondary metabolite biosynthetic gene cluster (smBGCs) [30,31]. Earlier, researchers used to clone and sequence smBGCs to identify the genes/proteins involved in producing a particular secondary metabolite. The onset of genome sequencing and the advancement of science, especially in bioinformatics, led to the development of software programs that can automatically detect smBGCs [32]. Due to this advancement, many smBGCs were reported in species belonging to different biological kingdoms [30,31,33].

In the bacterial kingdom, species belonging to the phylum *Actinobacteria* are well-known for producing secondary metabolites [33,34,35,36], especially species of the genus *Streptomyces* [37]. It is a well-known fact that two-thirds of the clinically valuable antibiotics come from *Streptomyces* species [37]. Actinomycetes belonging to the genus *Salinispora* produce biotechnologically valuable secondary metabolites [38,39,40,41,42,43,44,45,46,47]. Salinosporamide A, a secondary metabolite, is one of the best examples, which is now under clinical trials as an anticancer drug [48]. 

*Salinispora* is the first genus of *Actinobacteria* identified for its requirement of seawater for growth [49]. This genus includes three distinct but closely related species *Salinispora arenicola*, *S. pacifica*, and *S. tropica* [36,50,51]. *Salinispora* species are widely distributed in tropical and subtropical marine environments with distinct geographical patterns [49,52]. The genome sequence of *S. tropica* revealed a large percentage of its genome (~9.9%) is dedicated to natural products biosynthesis, which was greater than any other natural product producing actinomycetes [47]. The genome sequencing analysis revealed that P450s were also part of smBGCs [47]. CYP107 from *S. arenicola* CNS-205 *is* involved in the biosynthesis of secondary metabolites, saliniketal, and rifampicin [53]. Apart from these notable mentions, no information is available on *Salinispora* species P450s. 

Despite knowing that *Salinispora* species produce different types of human valuable secondary metabolites/natural products and the role of P450s in attributing diversity to these compounds, to date, comparative analysis of P450s and their role in secondary metabolism in *Salinispora* species is not reported. This study is aimed to address this research gap by performing genome-wide data mining, identification, annotation (assigning family and subfamily), and phylogenetic analysis of P450s in *Salinispora* species. The study also encompasses identification of P450s part of smBGCs, and comparative analysis of *Salinispora* P450 features with other bacterial species belonging to the genera, *Streptomyces* and *Mycobacterium*, phyla *Firmicutes* and *Bacteroidetes*, and the classes *Alpha*- and *Gamma*-*proteobacteria*.

## 2. Materials and Methods

### 2.1. Species and Database Information

A total of 126 *Salinispora* species genomes (permanent and finished draft genomes) are available for public use at the Joint Genome Institute Integrated Microbial Genomes and Microbiomes (JGI IMG/M) [54,55] were used in this study (last accessed on 2 February 2022). Information on the species and their genome IDs used in the study is provided in Table S1.

### 2.2. Genome Data Mining and Identification of P450s

Genome data mining and identification of P450s in *Salinispora* species were carried out following the protocol described elsewhere [56,57]. Each *Salinispora* species genome available at JGI IMG/M [54,55] was searched for P450s using the InterPro code “IPR001128”. The hit protein sequences were then searched for the presence of P450 characteristic motifs such as EXXR and CXG [58,59]. Proteins with one of these motifs or short amino acid length are considered P450-fragments. P450 fragments were not considered for the final P450 family and subfamily count. 

### 2.3. Assigning Family and Subfamily to P450s

Above selected P450s were assigned to different families and subfamilies based on the International P450 Nomenclature Committee rule [60,61,62], proteins with a percentage identity greater than 40% were assigned to the same family as named homolog P450s, and those that had greater than 55% identity were assigned to the same subfamily as named homolog P450s. Proteins with a percentage identity of less than 40% were assigned to a new family. *Salinispora* species P450s, along with P450-fragments, are presented in Table S2.

### 2.4. Phylogenetic Analysis of P450s

Phylogenetic analysis of P450s was carried out following the procedure described elsewhere [63,64]. The phylogenetic tree of P450s was constructed using protein sequences. Firstly, the MAFFT v6.864 [65] was used to align the Trex web server’s protein sequences [66]. The alignments were then used to interpret the best tree by the Trex web server [66]. Finally, the best-inferred tree was visualized, colored, and generated by a web-based tool, VisuaLife [67].

### 2.5. Salinispora Species P450s Profile Heat-Maps

P450 profile heat-maps were generated following a method described elsewhere [64,68] to check the presence and absence of or co-presence of or conserved nature of P450 families in *Salinispora* species. Briefly, a tab-delimited file was imported into Multi-Experiment Viewer (Mev) [69], and hierarchical clustering using a Euclidean distance metric was used to cluster the data. 126 *Salinispora* species formed the vertical axis, and P450 families formed the horizontal axis. Data were presented as −3 for family absence (green) and 3 for family presence (red). 

### 2.6. Identification of P450s Part of smBGCs

P450s that are part of smBGCs were identified following the method described elsewhere [56,57]. Briefly, for each *Salinispora* species genome available at JGI IMG/M [54,55], the smBGCs were searched for the presence of P450s using the P450 gene ID. The cluster type is noted if a P450 is found as part of the cluster. Results were recorded on Excel spreadsheets and represented species-wise smBGCs, smBGC type, and P450s part of specific smBGCs. Among 126, only 103 *Salinispora* species smBGCs information is available at JGI IMG/M [54,55]. Thus the same 103 *Salinispora* species smBGCs were analyzed for the presence of P450s (Table S1). 

### 2.7. Data Analysis

All calculations were carried out following the procedure reported previously by our laboratory [68]. The average number of P450s was calculated using the formula: Average number of P450s = Number of P450s/Number of species. The P450 diversity percentage was calculated using the formula: P450 diversity percentage = 100 × Total number of P450 families/Total number of P450s × Number of species with P450s. The percentage of P450s that formed part of BGCs was calculated using the formula: Percentage of P450s part of BGCs = 100 × Number of P450s part of BGCs/Total number of P450s present in species.

### 2.8. Comparative Analysis of P450s and smBGCs Data

For comparative analysis of P450s and smBGCs, information for bacterial species belonging to different groups such as classes, *Alpha*- and *Gamma*-*proteobacteria* [64,68], phyla, *Firmicutes* [70] and *Cyanobacteria* [71], and the genera, *Streptomyces* [56,72], *Mycobacterium* [72,73], was resourced from published articles.

## 3. Results and Discussion

### 3.1. Salinispora Species P450 Profiles

**Figure 1:**
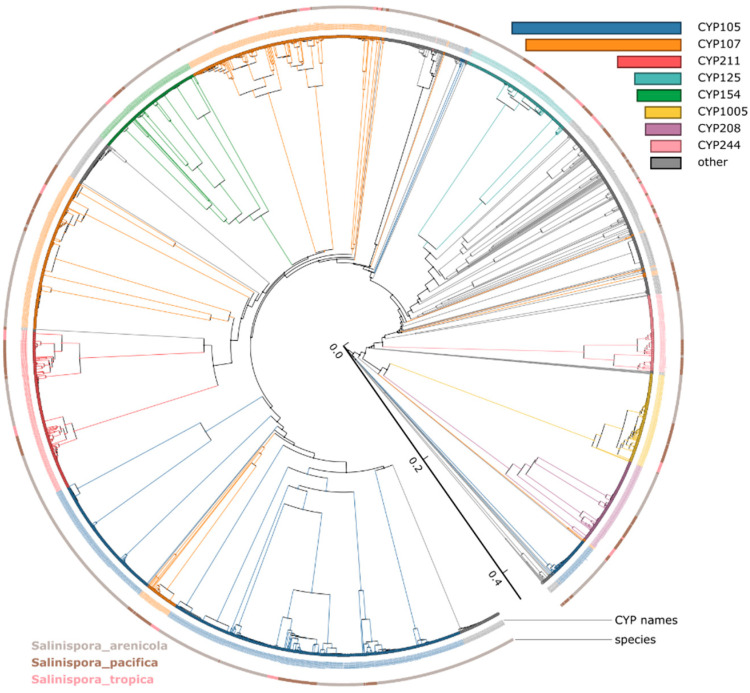
Phylogenetic analysis of *Salinispora* species P450s. 2643 P450s were used to construct the tree, and the members of the eight most abundant P450 families are highlighted in different colors and indicated in the figure. P450 protein sequences used to build the tree are listed in Table S2. A high-resolution phylogenetic tree is provided in Figure S1.

Genome-wide data mining and annotation of P450s in 126 *Salinispora* species revealed the presence of 2643 P450s in their genomes (Figure 1, Table 1 and Table 2). The P450 count in *Salinispora* species ranged from 10 to 35 P450s, with an average of 21 P450s (Table 1 and Table 2). Apart from the complete P450 sequences, 129 P450 fragments were also found in some *Salinispora* species (Table 2). P450 fragments in species are natural [58,70,74], and thus, these were excluded from further analysis. Among *Salinispora* species, *S. arenicola* CNY280 has the highest number of P450s (35 P450s), and *S. pacifica* CNS801 and *S. pacifica* CNT148 have the lowest number of P450s (10 P450s each) (Table 2). Comparative analysis revealed that *Salinispora* species have the highest average number of P450s than species belonging to *Cyanobacteria*, *Firmicutes*, *Alphaproteobacteria*, and *Gammaproteobacteria* (Table 1). However, *Salinispora* species had the lowest average number of P450s compared to species belonging to *Streptomyces* and *Mycobacterium* (Table 1). A point to be noted is that, among bacterial species, species belonging to the phylum *Actinobacteria* have the highest average number of P450s (Table 1). This indicates selective enrichment of P450s in these species due to their adaptation to ecological niches vis a vis P450s, helping them adapt to diverse ecological niches described elsewhere [58,74,75]. *Salinispora* species P450s, along with P450-fragments, are presented in Table S2.

### 3.2. CYP105 and CYP107 Families Are Bloomed in Salinispora Species

Based on the International P450 Nomenclature Committee Rules [60,61,62], all 2643 P450s can be grouped into 45 families and 103 subfamilies (Table 1 and Table 3). Phylogenetic analysis revealed that large P450 families CYP105 and CYP107 were scattered across the evolutionary tree (Figure 1). Previously, this phenomenon was observed for these P450 families [56,72]. Authors suggested that phylogenetic-based annotation of P450s could detect similarity cues beyond a simple percentage identity cutoff [56,72]. Except for CYP105 and CYP107, the rest of the P450s are grouped as per their families (Figure 1). A point to be noted is that most of the P450s are orthologs considering the *Salinispora* species analyzed in this study are different strains of three species. Comparative analysis revealed that *Salinispora* species have the lowest number of P450 families and subfamilies compared to other actinomycetes such as *Streptomyces* and *Mycobacterium* (Table 1). 

Among *Salinispora* species, *S. arenicola* CNY280 had the highest number of P450 families (18) and P450 subfamilies (32) in its genome (Table 1). This is quite an interesting observation where a species with the highest number of P450s also had the highest number of P450 families and subfamilies. This phenomenon was not found in other actinomycetes such as *Streptomyces* [56] and *Mycobacterium* [72,73]. For example, in *Streptomyces* species, *Streptomyces albulus* ZPM had the highest number of P450s, but *Streptomyces rimosus rimosus* ATCC 10970, and *Streptomyces clavuligerus* had the highest number of P450 families and subfamilies, respectively [56]. Among mycobacterial species, *Mycobacterium rhodesiae* NBB3 had the highest P450s and P450 families, but *M. marinum* had the highest P450 subfamilies [72,73]. 

Analysis of P450 families and subfamilies suggested that P450s in *Salinispora* species bloomed (presence of more copies of the same P450 family in a species by duplication of an ancestral gene) (Table 3). Among P450 families, the CYP105 was dominant with 600 members, followed by CYP107 with 551 members, CYP211 with 225 members, CYP125 with 164 members, CYP154 with 155 members, CYP1005 with 127 members, and CYP208 with 126 members (Table 3). These P450 families contributed more than 70% to the total P450s (Table 3). This indicates that P450 families such as CYP105, CYP107, CYP211, CYP125, and CYP154 are bloomed, whereas CYP1005 and CYP208 families are expanded in these species. Comparing the dominant P450 families revealed that CYP105 is prevalent only in *Salinispora* species (Table 1), where this family was second most dominant in *Streptomyces* species (Table 1). Interestingly, the second most dominant P450 family of *Salinispora* species, CYP107, was dominant in species belonging to bacterial groups *Streptomyces*, *Firmicutes* and *Gammaproteobacteria* (Table 1). The blooming was also observed at the subfamily level, indicating these P450s are preferred by *Salinispora* species for a particular reason. For example, subfamily AB was dominant with 124 members in CYP105; Subfamily AY was dominant with 116 members in CYP107, subfamily A was dominant with 128 members in CYP125, Subfamily M was dominant with 150 members, subfamily A was dominant with 126 members in CYP208, and Subfamily B dominant with 124 members in CYP211 (Table 3). Due to the blooming of specific P450s at the family level, *Salinispora* species had the lowest P450 diversity percentage, the same as *Firmicutes* species (Table 1). The blooming or expansion of P450s is a common phenomenon in organisms and is observed in other bacterial species (Table 2). It has been hypothesized that species enrich specific P450s in their genomes that are beneficial to them, particularly to adapt to ecological niches [56,72].

### 3.3. CYP107 and CYP125 Are Conserved in Salinispora Species

**Figure 2:**
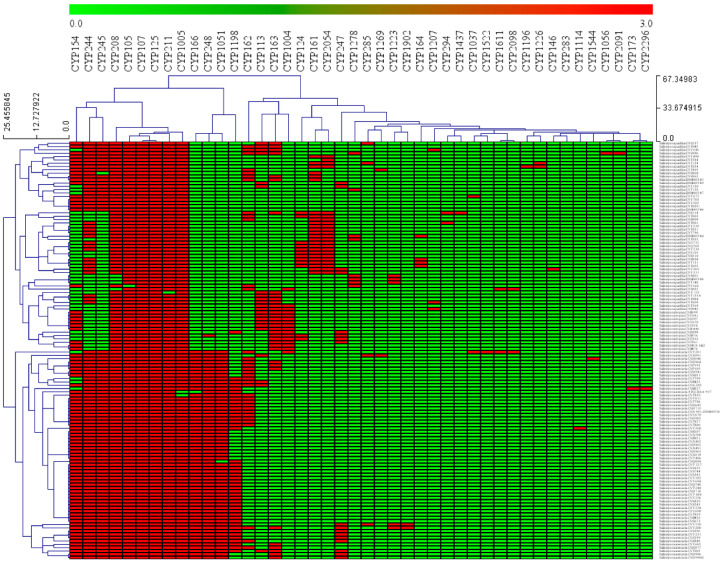
Heat-map of P450 family conservation or co-presence analysis in *Salinispora* species. In the heat-map, the presence and absence of P450 families are indicated in red and green colors. The horizontal axis represents P450 families, and the vertical axis represents *Salinispora* species.

P450 family conservation analysis revealed that CYP107 and CYP125 families are conserved in 126 *Salinispora* species (Figure 2). Except for a few species, CYP208 (4 species), CYP105 (one species), CYP211 (one species), and CYP1005 (2 species), the rest of the *Salinispora* species have these families (Figure 2). In addition to this, P450 families such as CYP154, CYP244, CYP245, CYP166, CYP248, and CYP1056 are co-present in many species (Figure 2). This suggests a prominent role of these P450 families in these species, possibly in secondary metabolism as observed in other bacterial species [58,72,74]. Conservation or co-presence of specific P450s in other bacterial species was also reported. The CYP107 family is conserved in all 203 *Streptomyces* species, and P450 families such as CYP156, CYP105, CYP154, and CYP157 are also present in the majority of the *Streptomyces* species [56]. Ten P450 families, CYP51, CYP123, CYP125, CYP130, CYP135, CYP136, CYP138, CYP140, CYP144, and CYP1128, were conserved in mycobacterial species [73]. Analysis of conservation of P450 families in 229 *Firmicutes* species and 114 cyanobacterial species revealed no conservation of the P450 family [70,71]. Still, some of the P450 families were co-present in most of the species. The P450 families CYP152, CYP107, CYP012, and CYP109, were found to be a co-presence in most *Firmicutes* species [70], and the P450 families CYP110 and CYP120 were found to be a co-presence in most cyanobacterial species [71]. 

If a P450 family is conserved or few P450 families are co-presence, these families play an important role in a species’s primary- or secondary-metabolism. Previous studies showed that this type of P450s prominently plays a role in secondary metabolism, helping species adapt to diverse ecological niches [58,59,72,74,75]. The importance of P450 families that are conserved and co-presence in *Salinispora* species is discussed in detail in the next section.

### 3.4. Unprecedented Number of P450s Involved in smBGCs

Analysis of the P450s part of smBGCs revealed that many P450s (47%) are part of these clusters, indicating their involvement in producing different secondary metabolites in *Salinispora* species (Table 4 and Table S1). The percentage of P450s part of smBGCs in *Salinispora* species was found to be unprecedented compared to other bacterial species, including other actinomycetes *Streptomyces* species and mycobacterial species that had 30% and 27% of P450s as part of smBGCs (Table 1). This suggests that *Salinispora* species dedicated half of their P450s to the production of secondary metabolites.

**Figure 3:**
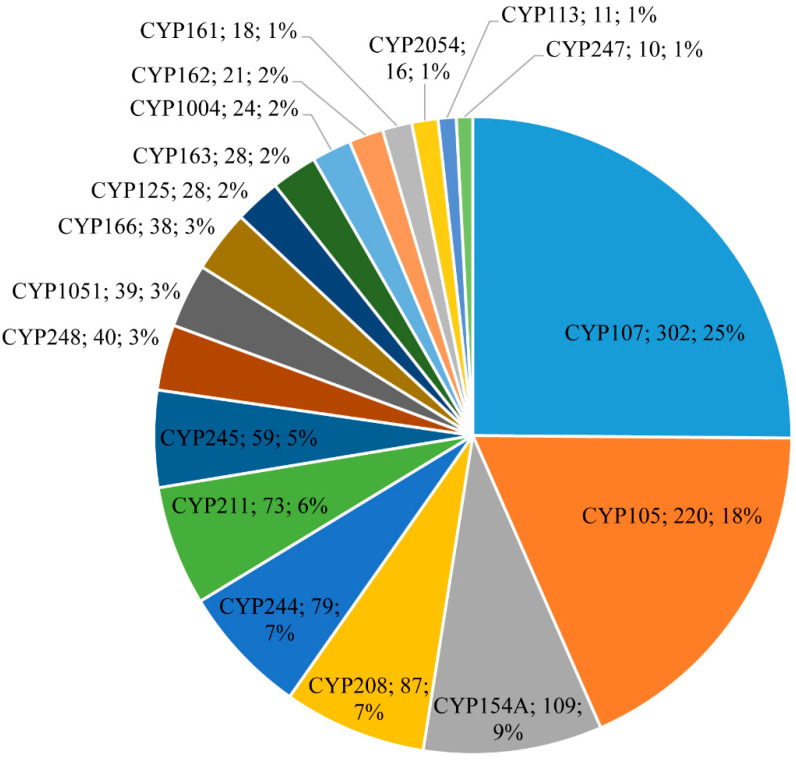
Comparative analysis of P450s associated with secondary metabolism in *Salinispora* species. The P450 family name, number of P450s, and the percentage of the total number of P450s that are part of secondary metabolite biosynthetic gene clusters (smBGCs) are presented in the figure. Detailed information on secondary metabolite clusters, species, and P450s are shown in Table S1.

Among 2643 P450s, 1236 P450s belonging to the 35 P450 families were part of smBGCs (Figure 3 and Table 4 and Table S1). This means almost 78% of P450 families of *Salinispora* species are involved in secondary metabolism. Among the families that are part of smBGCs, CYP107 is dominant with 302 members (25%), followed by CYP105 with 220 members (18%), CYP208 with 87 members (7%), CYP244 with 79 members (7%), and CYP211 with 73 members (6%) (Figure 3 and Table S1). Analysis of the P450s part of smBGCs revealed a strong correlation between the dominant P450 families (Table 3) being dominant in smBGCs (Figure 3). This suggests that *Salinispora* species are enriched by blooming or expanding these P450 families (as discussed in the previous section) in their genome to produce secondary metabolites.

Analysis of P450 smBGCs revealed the presence of 18 types (Table 4 and Table S2). Among the types, Type I PKS (Polyketide synthase) (T1PKS) was dominant with 223 clusters, followed by nonribosomal peptides (NRPS) (205 clusters) and Type II PKS (T2PKS) (76 clusters) (Table 4 and Table S1). This suggests that most of the secondary metabolites produced by P450 smBGCs are T1PKS. When the P450 smBGCs were further analyzed for the number of P450s and P450 families, the dominant BGC type was not found to be dominant concerning the number of P450s being part of that smBGC type (Table 4 and Table S1). NRPS had the highest number of P450s (395 P450s), followed by T1PKS (275 P450s), oligosaccharide (121 P450s), and indole (105 P450s) (Table 4 and Table S1). The difference being not having more P450s despite being dominant smBGCs such as T1PKS is that the other smBGCs have more P450s per se more than one P450 being part of that type (Table 4 and Table S1). This phenomenon of more than one P450 being part of smBGCs has been reported earlier in other bacterial species [75]. However, having up to 6 P450s as part of smBGCs is unprecedented (Table 4), suggesting these clusters produce diverse secondary metabolites. The P450s co-present in different *Salinispora* species were part of the same cluster (Table 4). Based on the arrangement of P450s concerning their family/subfamily and the number of P450s in smBGCs, it is clear that these smBGCs are orthologs (Table 4). These smBGCs are passed into different *Salinispora* species from a single ancestor before diverging into *S. arenicola*, *S. pacifica* and *S. tropica*. 

### 3.5. Functional Prediction of Salinispora Species P450s

Most of the *Salinispora* species P450s are orphans without an assigned biological function. Based on the homolog P450s from other organisms and being part of smBGCs, some P450 functions can be predicted. CYP105 and CYP107 members are involved in the degradation/biotransformation of xenobiotics and biosynthesis of secondary metabolites [76,77,78,79,80]. CYP107 from *S. arenicola* CNS-205 is involved in secondary metabolite biosynthesis [53]. It catalyzes multiple oxidative rearrangement reactions in the biosynthesis of saliniketal and rifampin [53]. CYP105 and CYP107 members’ enzymatic functions could help *Salinispora* species utilize diverse compounds as carbon sources, detoxify toxic compounds, or kill other bacterial species to thrive in the environment. It is no doubt that due to these beneficial properties, *Salinispora* species enriched these family members in their genomes. CYP125 members conserved in *Salinispora* species are cholesterol and cholest-4-en-3-one hydroxylases [81,82]. One can assume that CYP125 members possibly help *Salinispora* species utilize cholesterol or cholesterol-like molecules as carbon sources. Growth of *S. arenicola* CNS-205 on cholesterol where complete degradation of cholesterol was observed [83] strongly supports this assumption considering these species do have CYP125 in their genome.

Interestingly, the presence of CYP125 members as part of smBGCs as observed in *Salinispora* species (Table 4) is also observed in mycobacterial species [75], indicating CYP125 members do have other functions apart from cholesterol oxidation. CYP146 members are involved in β-hydroxytyrosine formation, a precursor for the biosynthesis of vancomycin antibiotics [84]. Interestingly, only a single member was found in *Salinispora* species (Table 3) and is not part of smBGCs, complicating predicting its role in these species.

CYP154 members are involved in regio- and stereo-selective hydroxylation of different steroids [85,86]. CYP154 from *Nocardia farcinica* IFM10152 is a bifunctional enzyme with *O*-dealkylation and *ortho*-hydroxylation activities [87]. This P450 converts formononetin, an isoflavone compound, into *ortho*-dihydroxy-isoflavone [87]. In *Salinispora* species, CYP154 members are dominant, indicating they may attribute the above-said activities to these species. However, the role of CYP154 in the generation of secondary metabolites and these compounds’ properties concerning *Salinispora* species is of future interest (Figure 3 and Table 4). 

CYP163A and CYP163B members produce novobiocin, aminocoumarin antibiotic [88], and skyllamycin, a potent inhibitor of the platelet-derived growth factor [89]. CYP162A members are involved in peptidyl nucleoside antibiotic nikkomycin synthesis [90,91]. CYP161A members are involved in the biosynthesis of antibiotics, pimaricin [92], and amphotericin [93]. CYP113 members are involved in the production a variety of antibiotics erythromycin [94,95], tylosin [96,97] and himastatin [98,99]. The presence of the CYP161-CYP163 and CYP113 members as part of smBGCs in *Salinispora* species (Figure 3 and Table 4) suggests that these members are certainly involved in the production of secondary metabolites in these species. 

CYP244 and CYP245 members are involved in the biosynthesis of antibiotic rapamycin [100,101]. These two P450s together as part of smBGCs clusters in *Salinispora* species (Table 4) indicate they are working together in producing secondary metabolite. CYP248A members are involved in the production of antibiotic aureothin [102]. *Salinispora* species have 63 CYP248A members (Table 3), and 40 of them are part of smBGC (Figure 3 and Table 4), indicating their prominent role in secondary metabolites production. CYP124 members are known for their terminal hydroxylation of methyl branched-lipids in *M. tuberculosis* [103]. None of these members were found as part of smBGCs in *Salinispora* species (Table 4), indicating their limited role possibly in the oxidation of different methylated-aliphatic lipids in these species. 

It is evident from the data presented in this article that close to half of *Salinispora* species P450s (1236 P450s) are part of smBGCs. Thus, we predict that these P450s play a role in producing different secondary metabolites characteristic of smBGC types (Table 4 and Table S2). The detailed information on species name, list of P450s part of smBGCs, their cluster information, and BGC type is presented in Table S1. 

## 4. Conclusions

*Salinispora* species being marine organisms within the phylum *Actinomycetes,* are considered model organisms for studying bacterial diversity and secondary metabolite production. Compared to the genera *Streptomyces* and *Mycobacterium*, the genus *Salinispora* has an unprecedented number of P450s as part of secondary metabolite biosynthetic gene clusters (smBGCs), indicating a great diversity of secondary metabolites produced by these species. The presence of up to six P450s as part of smBGCs is unusual and not observed in other bacterial species. Future functional characterization of P450s sheds lighter on the untapped secondary metabolite biotechnological potentials from *Salinispora* species. Based on the data presented in this article and the literature published on P450s function, we predict that *Salinispora* species enriched or expanded specific P450s in their genome to utilize diverse compounds as carbon sources to detoxify toxic compounds or kill other bacterial species to thrive in the environment. 

## Figures and Tables

**Table 1:** Comparative analysis of key features of P450s and their association with secondary metabolism between *Salinispora* species and different bacterial species. Abbreviation: No., number of; BGCs: biosynthetic gene clusters.

Category	*Salinispora* Species	*Streptomyces* Species	Mycobacterial Species	Cyanobacterial Species	*Firmicutes* Species	Alphaproteobacterial Species	Gammaproteobacterial Species
Species analysed	126	203	60	114	972	599	1261
Species without P450s	0	0	0	0	743	370	1091
Species with P450s	126	203	60	114	229	229	169
Percentage of species with P450s	100	100	100	100	24	38	13
No. of P450s	2643	5460	1784	341	712	873	277
No. of families	45	253	77	36	14	143	81
No. of subfamilies	103	698	132	79	53	214	102
Dominant P450 family	CYP105	CYP107	CYP125	CYP110	CYP107	CYP202	CYP133 & CYP107
Average No. of P450s	21	27	30	3	3	4	2
P450 diversity percentage	0.01	0.02	0.07	0.09	0.01	0.07	0.17
No. of P450s part of BGCs	1236	1231	204	27	126	21	49
No. of P450 families part of BGCs	35	135	31	6	10	16	22
Percentage of P450s part of BGCs	47	23	11	8	18	2	18
Reference(s)	This study	[56,72]	[72,73]	[71]	[70]	[64]	[68]

**Table 2:** Genome-wide data mining and annotation of P450s in 126 *Salinispora* species. Abbreviation, No. indicates the number in the table.

Species Name	No. of P450s	No. of P450 Fragments	No. of P450 Families	No. of Subfamilies
*Salinispora arenicola* CNH996	26	6	14	25
*Salinispora arenicola* CNH996B	27		14	25
*Salinispora arenicola* CNY280	35		18	32
*Salinispora arenicola* CNH877	34		15	30
*Salinispora arenicola* CNS848	32		16	29
*Salinispora arenicola* CNT798	31		14	27
*Salinispora arenicola* CNH643	31	1	14	28
*Salinispora arenicola* CNS-991	31		15	28
*Salinispora arenicola* CNT799	31		14	28
*Salinispora arenicola* CNY679	31	1	14	27
*Salinispora arenicola* CNT850	31		13	27
*Salinispora arenicola* CNT800	31		14	28
*Salinispora arenicola* CNY011	31		14	26
*Salinispora arenicola* CNY230	30		17	30
*Salinispora arenicola* CNH713	30		14	27
*Salinispora arenicola* CNH905	31	1	14	28
*Salinispora arenicola* CNT857	30		14	28
*Salinispora arenicola* CNY281	29	1	17	29
*Salinispora arenicola* CNH941	29		14	26
*Salinispora arenicola* CNB527	29	4	15	27
*Salinispora arenicola* CNT859	29		13	26
*Salinispora arenicola* CNT005	28		16	28
*Salinispora arenicola* CNH964	28	1	14	24
*Salinispora arenicola* CNP193	28		14	26
*Salinispora arenicola* CNP105	28	2	14	25
*Salinispora arenicola* CNH646	28		14	26
*Salinispora arenicola* CNR425	28		15	28
*Salinispora arenicola* CNS-205	28	1	15	28
*Salinispora arenicola* ATCC BAA-917	27	13	11	21
*Salinispora arenicola* CNY685	26	6	14	26
*Salinispora arenicola* CNS325	26		13	26
*Salinispora arenicola* CNS744	26		13	26
*Salinispora arenicola* CNY694	26	6	13	26
*Salinispora arenicola* CNY260	26		14	26
*Salinispora arenicola* CNT-088	26	1	13	24
*Salinispora arenicola* CNB458	26	4	13	26
*Salinispora arenicola* CNS296	25	1	14	25
*Salinispora arenicola* CNY231	25		14	26
*Salinispora arenicola* CNY282	25		13	25
*Salinispora arenicola* CNS299	25	1	14	25
*Salinispora arenicola* CNQ748	25		13	25
*Salinispora arenicola* CNY244	25		13	25
*Salinispora arenicola* CNS820	25		13	25
*Salinispora arenicola* CNS673	25		13	25
*Salinispora arenicola* CNY237	24		12	24
*Salinispora arenicola* CNS342	24	1	13	24
*Salinispora arenicola* CNH718	24	1	13	24
*Salinispora arenicola* CNX891	24	3	15	24
*Salinispora arenicola* CNY256	24		13	25
*Salinispora arenicola* CNS243	24	1	13	24
*Salinispora arenicola* CNY234	24	1	13	24
*Salinispora arenicola* CNY690	24	4	13	24
*Salinispora arenicola* CNQ884	23	1	13	25
*Salinispora arenicola* CNR107	22		12	22
*Salinispora arenicola* CNR921	22		12	22
*Salinispora arenicola* CNH962	22	1	12	22
*Salinispora arenicola* CNX481	22	2	12	22
*Salinispora arenicola* CNH963	22	1	12	22
*Salinispora arenicola* CNX814	22	1	12	21
*Salinispora arenicola* CNY486	22	1	13	24
*Salinispora arenicola* CNX508	21	1	12	21
*Salinispora arenicola* CNX482	21	1	12	21
*Salinispora pacifica* CNS996	21	1	15	21
*Salinispora pacifica* CNS237	20	1	12	19
*Salinispora pacifica* CNY646	20	1	13	19
*Salinispora tropica* CNT261	20	2	10	18
*Salinispora pacifica* DSM 45548	19		7	10
*Salinispora pacifica* CNT045	19	1	13	19
*Salinispora pacifica* CNT124	19		13	19
*Salinispora pacifica* DSM 45543	19		12	18
*Salinispora tropica* CNB536	19		11	19
*Salinispora tropica* CNH898	18	1	11	20
*Salinispora pacifica* CNT403	18	1	12	17
*Salinispora pacifica* CNS860	18	2	11	16
*Salinispora pacifica* CNS863	18	2	12	17
*Salinispora tropica* CNY012	18	2	10	18
*Salinispora pacifica* CNT584	17		11	17
*Salinispora pacifica* DSM 45549	17	1	11	16
*Salinispora pacifica* CNR114	17	1	13	17
*Salinispora tropica* CNR699	17	2	10	16
*Salinispora pacifica* CNT854	18		13	18
*Salinispora pacifica* CNT150	17	1	11	16
*Salinispora pacifica* CNT131	17	1	11	15
*Salinispora pacifica* DSM 45544	16		11	16
*Salinispora pacifica* CNT003	16	1	10	15
*Salinispora tropica* CNY681	16	1	10	16
*Salinispora tropica* CNS197	16	1	10	16
*Salinispora tropica* CNY678	16	1	10	16
*Salinispora tropica* CNT250	16	1	10	16
*Salinispora tropica* CNB-440	16	1	10	16
*Salinispora tropica* CNS416	15		9	15
*Salinispora pacifica* CNT001	15	1	11	15
*Salinispora pacifica* CNY498	15	1	11	15
*Salinispora pacifica* CNR909	15	1	10	15
*Salinispora tropica* CNB476	15	1	9	15
*Salinispora pacifica* CNR894	15		11	15
*Salinispora pacifica* CNY363	15		11	15
*Salinispora pacifica* CNS055	15		9	15
*Salinispora pacifica* CNT603	15	1	11	15
*Salinispora pacifica* CNT138	14		10	14
*Salinispora pacifica* DSM 45547	14	1	10	14
*Salinispora pacifica* CNH732	14	1	10	14
*Salinispora pacifica* CNY703	14		9	13
*Salinispora pacifica* CNQ768	14	1	10	14
*Salinispora pacifica* CNY673	14		10	14
*Salinispora pacifica* CNT855	14		9	14
*Salinispora pacifica* CNY239	14	1	10	14
*Salinispora pacifica* CNR942	14	1	10	14
*Salinispora pacifica* DSM 45546	14	1	10	14
*Salinispora pacifica* CNT609	14	1	10	14
*Salinispora pacifica* CNY331	14		10	14
*Salinispora tropica* CNR416	14		9	14
*Salinispora pacifica* CNY330	13		9	13
*Salinispora pacifica* CNT851	13	1	9	13
*Salinispora pacifica* CNT796	13	1	9	13
*Salinispora pacifica* CNS103	13		9	13
*Salinispora pacifica* CNY202	13	1	9	13
*Salinispora pacifica* CNT133A	13		9	13
*Salinispora arenicola* CNY666	13	5	8	13
*Salinispora pacifica* CNT029	13	1	9	13
*Salinispora pacifica* CNT084	13		9	13
*Salinispora pacifica* CNR510	13		9	13
*Salinispora pacifica* CNT569	12	1	9	13
*Salinispora pacifica* CNT-133	11	11	7	9
*Salinispora pacifica* CNS801	10		7	10
*Salinispora pacifica* CNT148	10		7	10

**Table 3:** Comparative analysis of P450 families and subfamilies in *Salinispora* species.

P450 Family	P450 Count	Percentage Count	Subfamily	Count	Percentage Count
CYP1004	34	1.29%	A	17	0.64
			B	17	0.64
CYP1005	127	4.81%	A	127	4.79
CYP1037	2	0.08%	B	2	0.08
CYP1051	60	2.27%	A	60	2.26
CYP1056	2	0.08%	B	2	0.08
CYP105	600	22.70%	AB	124	4.67
			AH	4	0.15
			B	1	0.04
			BL	78	2.94
			BN	1	0.04
			CH	44	1.66
			CN	62	2.34
			CP	62	2.34
			CT	41	1.55
			EJ	3	0.11
			G	62	2.34
			H	3	0.11
			J	52	1.96
			W	63	2.37
CYP107	551	20.85%	AW	65	2.45
			AX	75	2.83
			AY	116	4.37
			CL	3	0.11
			CT	6	0.23
			E	38	1.43
			EP	2	0.08
			EU	44	1.66
			FH	25	0.94
			FJ	20	0.75
			FS	61	2.30
			GU	1	0.04
			HF	2	0.08
			LA	6	0.23
			N	2	0.08
			NE	2	0.08
			NF	2	0.08
			NG	4	0.15
			NH	8	0.30
			Q	63	2.37
			Z	6	0.23
CYP1114	1	0.04%	C	1	0.04
CYP113	24	0.91%	B	6	0.23
			D	1	0.04
			E	10	0.38
			R	2	0.08
			S	2	0.08
			T	1	0.04
			X	2	0.08
CYP1197	1	0.04%	A	1	0.04
CYP1198	43	1.63%	B	43	1.62
CYP1207	4	0.15%	A	4	0.15
CYP1223	6	0.23%	D	2	0.08
			A	4	0.15
CYP1226	2	0.08%	A	2	0.08
CYP124	15	0.57%	M	15	0.57
CYP125	164	6.21%	A	128	4.82
			G	36	1.36
CYP1269	2	0.08%	A	2	0.08
CYP1278	11	0.42%	A	5	0.19
			B	6	0.23
CYP1437	1	0.04%	C	1	0.04
CYP146	1	0.04%	A	1	0.04
CYP1522	1	0.04%	A	1	0.04
CYP154	155	5.86%	AJ	4	0.15
			J	1	0.04
			M	150	5.65
CYP1611	1	0.04%	B	1	0.04
CYP161	28	1.06%	N	23	0.87
		0.00%	T	5	0.19
CYP162	39	1.48%	A	11	0.41
			B	2	0.08
			G	2	0.08
			H	1	0.04
			J	1	0.04
			K	1	0.04
			L	1	0.04
			M	1	0.04
			N	1	0.04
			P	18	0.68
CYP163	39	1.48%	A	2	0.08
			B	37	1.39
CYP164	4	0.15%	C	4	0.15
CYP166	62	2.35%	A	62	2.34
CYP173	1	0.04%	K	1	0.04
CYP1902	2	0.08%	A	2	0.08
CYP2054	22	0.83%	A	22	0.83
CYP205	1	0.04%	A	1	0.04
CYP208	126	4.77%	A	126	4.75
CYP2091	1	0.04%	A	1	0.04
CYP2098	2	0.08%	A	2	0.08
CYP211	225	8.51%	B	124	4.67
			C	101	3.81
CYP2296	1	0.04%	A	1	0.04
CYP244	107	4.05%	A	107	4.03
CYP245	83	3.14%	A	83	3.13
CYP247	21	0.79%	A	21	0.79
CYP248	63	2.38%	A	63	2.37
CYP2611	1	0.04%	B	1	0.04
CYP283	1	0.04%	A	1	0.04
CYP285	4	0.15%	A	2	0.08
			D	2	0.08
CYP294A4	2	0.08%	A	2	0.08

**Table 4:** Secondary metabolite biosynthetic gene cluster (smBGC) types and P450s are part of the cluster in *Salinispora* species. smBGC types were again classified into different varieties based on the P450s. The smBGCs type count and the total number of P450s in the cluster variety are also presented. The same smBGCs type names listed in the antibiotics and secondary metabolite analysis shell (anti-SMASH) database [74] were used in the table. Detailed information on secondary metabolite clusters, species, and P450s are shown in Table S1.

smBGC Type	smBGC Type Count	smBGC Type Variety	P450s	P450 Count
Bacteriocin	47	46	CYP107AW	46
		1	CYP283A	1
betalactone	2	1	CYP162A6,CYP107HF1	2
		1	CYP113S1	1
butyrolactone	1	1	CYP105CT1,CYP154M5	2
Indole	54	51	CYP244A,CYP245A	102
		3	CYP244A	3
ladderane	18	4	CYP154M15,CYP125G6,CYP107FS2,CYP105CN1,CYP105CP2	20
		8	CYP107AX-fragment	8
		6	CYP107AX	6
lanthipeptide	2	1	CYP1223A5	1
		1	CYP105CP2,CYP105CN1,CYP107FS2,CYP248A2,CYP105W2	5
LAP	1	1	CYP154AJ2	1
lipolanthine	2	2	CYP1223A5	2
NRPS	205	1	CYP1004B1,CYP1004A1	2
		8	CYP1004B,CYP1004A,CYP125G	24
		1	CYP105CH2-fragment,CYP105CH1-fragment	2
		1	CYP105CN1	1
		1	CYP105CN1,CYP105CP2	2
		1	CYP105CN1,CYP107FS2,CYP125G6,CYP154M15	4
		1	CYP105CN1,CYP107FS2,CYP247A7	3
		1	CYP105CP2	1
		7	CYP105CP2,CYP105CN1,CYP107FS2	21
		10	CYP105CP2,CYP105CN1,CYP107FS2,CYP125G6,CYP154M15	50
		2	CYP105CP2,CYP105CN1,CYP107FS2,CYP248A2	8
		1	CYP105CP2,CYP105CN1,CYP107FS2,CYP248A2,CYP105W2	5
		3	CYP105W	3
		38	CYP107AY	38
		1	CYP107AY14,CYP244A-fragment2	2
		6	CYP107AY2,CYP105CT1,CYP154M5	18
		1	CYP107AY2,CYP163B16	2
		1	CYP107AY7,CYP244A5,CYP245A11	3
		1	CYP107AY9,CYP162B3	2
		1	CYP107AY9,CYP244A10	2
		2	CYP107CL2,CYP1056B2	4
		5	CYP107CT3	5
		1	CYP107CT3,CYP107AY7	2
		1	CYP107FS2	1
		6	CYP107FS2,CYP105CN1,CYP105CP2	18
		2	CYP107NH1,CYP247A8,CYP107Z27	6
		1	CYP107Z27,CYP247A8,CYP107NH1	3
		1	CYP113D13,CYP163B22	2
		1	CYP1196A2	1
		1	CYP1198B1	1
		1	CYP1198B1,CYP107AY2	2
		2	CYP1207A12	2
		1	CYP125G1,CYP1004A1,CYP1004B1	3
		1	CYP125G6,CYP154M15	2
		4	CYP1278A4	4
		1	CYP1437C1	1
		1	CYP154AJ3	1
		1	CYP154J2,CYP244A5,CYP245A11	3
		5	CYP154M1,CYP208A4	10
		6	CYP154M	6
		3	CYP154M,CYP208A	6
		1	CYP154M16,CYP211C6	2
		1	CYP154M21,CYP154M13	2
		1	CYP154M21,CYP154M13,CYP105W2,CYP248A2	4
		3	CYP154M21,CYP154M13,CYP105W2,CYP248A2,CYP154M20	15
		1	CYP154M21,CYP154M13,CYP105W2,CYP248A2,CYP154M20,CYP162P1	6
		12	CYP162	12
		1	CYP163A10,CYP162K1	2
		15	CYP163B	15
		3	CYP164C2	3
		5	CYP208A21,CYP154M16	10
		2	CYP208A4,CYP154M1	4
		8	CYP244A,CYP107AY	16
		5	CYP244A5,CYP245A11	10
		2	CYP244A,CYP107AY	4
		2	CYP244A	2
		1	CYP245A11	1
		3	CYP247A	3
		1	CYP247A8,CYP107NH1	2
		1	CYP247A8,CYP107Z27	2
		1	CYP248A2	1
		1	CYP248A2,CYP105W2	2
		1	CYP285D2	1
NRPS-like	30	5	CYP107EU	5
		1	CYP107EU1,CYP1198B1,CYP105CH1	3
		1	CYP107FH3,CYP161N4,CYP107AY9	3
		7	CYP107FH3,CYP2054A3,CYP161N4	21
		2	CYP161N4,CYP2054A3,CYP107FH3	6
		6	CYP162A8	6
		6	CYP166A4	6
		1	CYP166A4,CYP107Q4,CYP105G5	3
		1	CYP285A9-fragment,CYP285A9-fragment	2
oligosaccharide	35	1	CYP105CP2	1
		1	CYP105W2,CYP107FS2,CYP105CN1,CYP105CP2	4
		1	CYP105W2,CYP107NH1	2
		1	CYP105W2,CYP154M20,CYP154M13,CYP154M21,CYP248A2	5
		5	CYP105W2,CYP248A2	10
		1	CYP105W2,CYP248A2,CYP107FS2	3
		9	CYP105W2/3,CYP248A2,CYP107FS2,CYP105CN1,CYP105CP2	45
		1	CYP1269A2	1
		8	CYP154M20,CYP248A2,CYP105W2,CYP154M13,CYP154M21	40
		1	CYP2091A1	1
		3	CYP248A2	3
		3	CYP248A2,CYP105W2/3	6
other	4	2	CYP247A7	2
		2	CYP105AH4	2
		1	CYP1004A3,CYP1004B4,CYP113E2,CYP163B18	4
T1PKS	223	1	CYP105AH4	1
		1	CYP105BN4	1
		17	CYP105CH1/2	17
		1	CYP105CN1	1
		4	CYP105G5	4
		16	CYP105G5,CYP107Q4	32
		2	CYP105H11	2
		1	CYP107AY13	1
		24	CYP107E	24
		1	CYP107E3,CYP125G1,CYP1004A1,CYP1004B1	4
		8	CYP107EU1	8
		2	CYP107FH4	2
		1	CYP107NE1	1
		3	CYP107Q4	3
		17	CYP107Q4,CYP105G5	34
		6	CYP113E1/2	6
		1	CYP113E2,CYP107EP2	2
		2	CYP1198B2	2
		1	CYP125G1	1
		1	CYP1278B-fragment2	1
		5	CYP154M5,CYP105CT1	10
		1	CYP154M5,CYP105CT1,CYP105G5,CYP105CP2	4
		1	CYP154M5,CYP105CT1,CYP107AY2-fragment	3
		1	CYP154M5,CYP105CT2	2
		1	CYP1611B1,CYP2098A1	2
		29	CYP166A4	29
		1	CYP166A4,CYP107Q4,CYP105G5	3
		70	CYP208A	70
		1	CYP208A28,CYP154M18	2
		1	CYP211C5	1
		2	CYP294A4	2
T2PKS	76	2	CYP107NG1	2
		1	CYP107NH1	1
		1	CYP125G4	1
		1	CYP161T1	1
		69	CYP211C	69
		1	CYP2296A2,CYP166A4,CYP173K1	3
		1	CYP244A5,CYP211C6	2
T3PKS	8	7	CYP161N4,CYP2054A3,CYP107FH3	21
		1	CYP107FH3	1
Terpene	61	39	CYP1051A	39
		2	CYP105CT1	2
		7	CYP105CT1,CYP154M5	14
		6	CYP107AY	6
		4	CYP107AY9,CYP244A10	8
		1	CYP107E37	1
		1	CYP154AJ2	1
		1	CYP154M5	1
transAT-PKS	1	1	CYP113 × 1	1
transAT-PKS-like	8	8	CYP163B	8

## Data Availability

The authors confirm that the data supporting the findings of this study are available within the article and its Supplementary Materials.

## Supplementary Materials

The following supporting information can be downloaded at: https://www.mdpi.com/article/10.3390/microorganisms10050871/s1. Figure S1: Phylogenetic analysis of *Salinispora* species P450s. 2643 P450s were used to construct the tree, and the members of the eight most abundant P450 families are highlighted in different colors and indicated in the figure. P450 protein sequences used to build the tree are listed in Table S2. Table S1: Identification of P450s that are part of secondary metabolite biosynthesis tic gene clusters (smBGCs) in *Salinispora* species. Cluster-ID and BGC type is retrieved from Integrated Microbial Genomes & Microbiomes (IMG/M) database [54,55]. BGC Type was indicated for consistency with the standard BGC Type name terminology available in the anti-SMASH database [74]. Table S2: P450 sequences identified and annotated in *Salinispora* species. Each P450 is presented with its assigned name followed by gene ID (in parenthesis) and species name.
