# Supplementary material for: An Unprecedented Number of Cytochrome P450s Are Involved in Secondary Metabolism in Salinispora Species
Source: Microorganisms. 2022 Apr 21;10(5):871. doi: 10.3390/microorganisms10050871 (PMC9143469; doi:10.3390/microorganisms10050871)
Supplement: Supplementary file 1 [file microorganisms-10-00871-s001.zip › Supplementary Information.docx]

Article

| **Citation:** Malinga, N.A.; Nzuza, N.; Padayachee, T.; Syed, P.R.;  Karpoormath, R.; Gront, D.;  Nelson, D.R.; Syed, K. An  Unprecedented Number of  Cytochrome P450s Are Involved in Secondary Metabolism in *Salinispora* Species. *Microorganisms* **2022**, *10*, 871. https://doi.org/10.3390/microorganisms10050871  Academic Editors: Mireille Fouillaud and Laurent Dufossé  Received: 4 April 2022  Accepted: 19 April 2022  Published: 21 April 2022  **Publisher’s Note:** MDPI stays neutral with regard to jurisdictional claims in published maps and institutional affiliations.  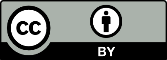  **Copyright:** © 2022 by the authors. Licensee MDPI, Basel, Switzerland. This article is an open access article distributed under the terms and conditions of the Creative Commons Attribution (CC BY) license (https://creativecommons.org/licenses/by/4.0/). |
| --- |

An Unprecedented Number of Cytochrome P450s Are Involved in Secondary Metabolism in *Salinispora* Species

^1^ Department of Biochemistry and Microbiology, Faculty of Science and Agriculture, University of Zululand, KwaDlangezwa 3886, South Africa; nsikelelo.malinga@gmail.com (N.A.M.); nomfundonzuza11@gmail.com (N.N.); teez07padayachee@gmail.com (T.P.)

^2^ Department of Pharmaceutical Chemistry, College of Health Sciences, University of KwaZulu-Natal, Durban 4000, South Africa; prosinah@gmail.com (P.R.S.); karpoormath@ukzn.ac.za (R.K.)

^3^ Faculty of Chemistry, Biological and Chemical Research Centre, University of Warsaw, Pasteura 1, 02-093 Warsaw, Poland; dgront@gmail.com

^4^ Department of Microbiology, Immunology and Biochemistry, University of Tennessee Health Science Center, Memphis, TN 38163, USA

***** Correspondence: drnelson1@gmail.com (D.R.N.); khajamohiddinsyed@gmail.com (K.S.); Tel.: +19-014-488-303 (D.R.N.); +27-035-902-6857 (K.S.)

Table of Contents

[Table S1: Identification of P450s that are part of secondary metabolite biosynthesis tic gene clusters (smBGCs) in Salinispora species. Cluster-ID and BGC type is retrieved from Integrated Microbial Genomes & Microbiomes (IMG/M) database [57,58]. smBGC Type was indicated for consistency with the standard BGC Type name terminology available in the anti-SMASH database [33]. 3](#_Toc101446248)

[Table S2: P450 sequences identified and annotated in Salinispora species. Each P450 is presented with its assigned name followed by gene ID (in parenthesis) and species name 116](#_Toc101446249)

[Full-length P450s 116](#_Toc101446250)

[P450-fragments 674](#_Toc101446251)

# **Table S1**: Identification of P450s that are part of secondary metabolite biosynthesis tic gene clusters (smBGCs) in Salinispora species. Cluster-ID and BGC type is retrieved from Integrated Microbial Genomes & Microbiomes (IMG/M) database [57,58]. smBGC Type was indicated for consistency with the standard BGC Type name terminology available in the anti-SMASH database [33].

| Sample Name | Genome ID | No of P450s part of clusters | P450 name | CLUSTER ID | BGC TYPE |
| --- | --- | --- | --- | --- | --- |
| *Salinispora arenicola* CNR107 | 2519103194 | 1 | CYP166A4 | 2519124058.c00001_F583DRA...region1 | T1PKS |
| *Salinispora pacifica* CNS801 | 2561511036 | 3 | CYP107AW9 | 2561515277.c00001_T435DRA...region3 | Bacteriocin |
|  |  |  | CYP1223A5 | 2561515279.c00003_T435DRA...region1 | lipolanthine |
|  |  |  | CYP211C4 | 2561515286.c00010_T435DRA...region1 | T2PKS |
| *Salinispora arenicola* CNY011 | 2517572153 | 12 | CYP208A12 | 2517596201.c00001_C576DRA...region2 | T1PKS |
|  |  |  | CYP244A4 | 2517596206.c00006_C576DRA...region1 | Indole |
|  |  |  | CYP245A7 | 2517596206.c00006_C576DRA...region1 | Indole |
|  |  |  | CYP1051A1 | 2517596207.c00007_C576DRA...region1 | Terpene |
|  |  |  | CYP105G5 | 2517596207.c00007_C576DRA...region2 | T1PKS |
|  |  |  | CYP107Q4 | 2517596207.c00007_C576DRA...region2 | T1PKS |
|  |  |  | CYP166A4 | 2517596209.c00009_C576DRA...region1 | T1PKS |
|  |  |  | CYP154M15 | 2517596211.c00011_C576DRA...region1 | ladderane |
|  |  |  | CYP125G6 | 2517596211.c00011_C576DRA...region1 | ladderane |
|  |  |  | CYP107FS2 | 2517596211.c00011_C576DRA...region1 | ladderane |
|  |  |  | CYP105CN1 | 2517596211.c00011_C576DRA...region1 | ladderane |
|  |  |  | CYP105CP2 | 2517596211.c00011_C576DRA...region1 | ladderane |
| *Salinispora arenicola* CNX508 | 2515154188 | 14 | CYP244A4 | 2515169292.c00005_B111DRA...region1 | Indole |
|  |  |  | CYP245A7 | 2515169292.c00005_B111DRA...region1 | Indole |
|  |  |  | CYP154M5 | 2515169296.c00009_B111DRA...region1 | T1PKS |
|  |  |  | CYP105CT1 | 2515169296.c00009_B111DRA...region1 | T1PKS |
|  |  |  | CYP1051A1 | 2515169297.c00010_B111DRA...region1 | Terpene |
|  |  |  | CYP105G5 | 2515169297.c00010_B111DRA...region2 | T1PKS |
|  |  |  | CYP107Q4 | 2515169297.c00010_B111DRA...region2 | T1PKS |
|  |  |  | CYP105CP2 | 2515169300.c00013_B111DRA...region2 | NRPS |
|  |  |  | CYP105CN1 | 2515169300.c00013_B111DRA...region2 | NRPS |
|  |  |  | CYP107FS2 | 2515169300.c00013_B111DRA...region2 | NRPS |
|  |  |  | CYP208A12 | 2515169301.c00014_B111DRA...region1 | T1PKS |
|  |  |  | CYP211C1 | 2515169303.c00016_B111DRA...region1 | T2PKS |
|  |  |  | CYP105W2 | 2515169305.c00018_B111DRA...region1 | NRPS |
|  |  |  | CYP248A2 | 2515169312.c00025_B111DRA...region1 | oligosaccharid |
| *Salinispora arenicola* CNY 237 | 2517572163 | 17 | CYP211C1 | 2517597048.c00002_C586DRA...region1 | T2PKS |
|  |  |  | CYP245A7 | 2517597050.c00004_C586DRA...region2 | Indole |
|  |  |  | CYP244A4 | 2517597050.c00004_C586DRA...region2 | Indole |
|  |  |  | CYP105CP2 | 2517597050.c00004_C586DRA...region4 | NRPS |
|  |  |  | CYP105CN1 | 2517597050.c00004_C586DRA...region4 | NRPS |
|  |  |  | CYP107FS2 | 2517597050.c00004_C586DRA...region4 | NRPS |
|  |  |  | CYP166A4 | 2517597054.c00008_C586DRA...region1 | T1PKS |
|  |  |  | CYP1051A1 | 2517597055.c00009_C586DRA...region1 | Terpene |
|  |  |  | CYP105G5 | 2517597055.c00009_C586DRA...region2 | T1PKS |
|  |  |  | CYP107Q4 | 2517597055.c00009_C586DRA...region2 | T1PKS |
|  |  |  | CYP208A12 | 2517597057.c00011_C586DRA...region1 | T1PKS |
|  |  |  | CYP107AY2 | 2517597063.c00017_C586DRA...region1 | NRPS |
|  |  |  | CYP105CT1 | 2517597063.c00017_C586DRA...region1 | NRPS |
|  |  |  | CYP154M5 | 2517597063.c00017_C586DRA...region1 | NRPS |
|  |  |  | CYP107EU1 | 2517597073.c00027_C586DRA...region1 | T1PKS |
|  |  |  | CYP248A2 | 2517597074.c00028_C586DRA...region1 | oligosaccharide |
|  |  |  | CYP105CH1 | 2517597075.c00029_C586DRA...region1 | T1PKS |
| *Salinispora arenicola* CNT005 | 2517572137 | 9 | CYP211C1 | 2517589297.c00005_B110DRA...region2 | T2PKS |
|  |  |  | CYP105W2 | 2517589298.c00006_B110DRA...region1 | oligosaccharide |
|  |  |  | CYP107FS2 | 2517589298.c00006_B110DRA...region1 | oligosaccharide |
|  |  |  | CYP105CN1 | 2517589298.c00006_B110DRA...region1 | oligosaccharide |
|  |  |  | CYP105CP2 | 2517589298.c00006_B110DRA...region1 | oligosaccharide |
|  |  |  | CYP244A4 | 2517589299.c00007_B110DRA...region1 | Indole |
|  |  |  | CYP245A7 | 2517589299.c00007_B110DRA...region1 | Indole |
|  |  |  | CYP113D13 | 2517589300.c00008_B110DRA...region1 | NRPS |
|  |  |  | CYP163B22 | 2517589300.c00008_B110DRA...region1 | NRPS |
| *Salinispora tropica* CNS416 | 2517572164 | 9 | CYP107AY1 | 2517597114.c00001_C590DRA...region2 | NRPS |
|  |  |  | CYP163B1 | 2517597115.c00002_C590DRA...region1 | NRPS |
|  |  |  | CYP211C1 | 2517597116.c00003_C590DRA...region1 | T2PKS |
|  |  |  | CYP208A4 | 2517597116.c00003_C590DRA...region3 | NRPS |
|  |  |  | CYP154M1 | 2517597116.c00003_C590DRA...region3 | NRPS |
|  |  |  | CYP107AW1 | 2517597117.c00004_C590DRA...region2 | Bacteriocin |
|  |  |  | CYP1004B1 | 2517597136.c00023_C590DRA...region1 | NRPS |
|  |  |  | CYP1004A1 | 2517597136.c00023_C590DRA...region1 | NRPS |
|  |  |  | CYP125G1 | 2517597148.c00035_C590DRA...region1 | T1PKS |
| *Salinispora arenicola* CNH996 | 2561511104 | 21 | CYP211C1 | 2571043777.c00002_T327DRA...region2 | T2PKS |
|  |  |  | CYP244A4 | 2571043781.c00006_T327DRA...region1 | Indole |
|  |  |  | CYP245A7 | 2571043781.c00006_T327DRA...region1 | Indole |
|  |  |  | CYP107AW7 | 2571043783.c00008_T327DRA...region1 | Bacteriocin |
|  |  |  | CYP166A4 | 2571043788.c00013_T327DRA...region1 | NRPS-like |
|  |  |  | CYP107AX-fragment9 | 2571043791.c00016_T327DRA...region1 | ladderane |
|  |  |  | CYP208A12 | 2571043796.c00021_T327DRA...region1 | T1PKS |
|  |  |  | CYP107AY2 | 2571043799.c00024_T327DRA...region1 | NRPS |
|  |  |  | CYP163B21 | 2571043804.c00029_T327DRA...region1 | NRPS |
|  |  |  | CYP154M5 | 2571043808.c00033_T327DRA...region1 | T1PKS |
|  |  |  | CYP105CT1 | 2571043808.c00033_T327DRA...region1 | T1PKS |
|  |  |  | CYP105G5 | 2571043810.c00035_T327DRA...region1 | T1PKS |
|  |  |  | CYP105CP2 | 2571043810.c00035_T327DRA...region1 | T1PKS |
|  |  |  | CYP105CN1 | 2571043815.c00040_T327DRA...region1 | NRPS |
|  |  |  | CYP107FS2 | 2571043815.c00040_T327DRA...region1 | NRPS |
|  |  |  | CYP247A7 | 2571043815.c00040_T327DRA...region1 | NRPS |
|  |  |  | CYP248A2 | 2571043818.c00043_T327DRA...region1 | NRPS |
|  |  |  | CYP105W2 | 2571043825.c00050_T327DRA...region1 | oligosaccharide |
|  |  |  | CYP107NH1 | 2571043825.c00050_T327DRA...region1 | oligosaccharide |
|  |  |  | CYP247A8 | 2571043840.c00065_T327DRA...region1 | NRPS |
|  |  |  | CYP1051A1 | 2571043857.c00082_T327DRA...region1 | Terpene |
| *Salinispora arenicola* CNH996B |  | 18 | CYP211C1 | 2561538853.c00002_CC96DRA...region2 | T2PKS |
|  |  |  | CYP107AX-fragment9 | 2561538858.c00007_CC96DRA...region1 | ladderane |
|  |  |  | CYP166A4 | 2561538858.c00007_CC96DRA...region2 | T1PKS |
|  |  |  | CYP244A4 | 2561538859.c00008_CC96DRA...region1 | Indole |
|  |  |  | CYP245A7 | 2561538859.c00008_CC96DRA...region1 | Indole |
|  |  |  | CYP107AW7 | 2561538861.c00010_CC96DRA...region1 | Bacteriocin |
|  |  |  | CYP208A12 | 2561538871.c00020_CC96DRA...region1 | T1PKS |
|  |  |  | CYP163B21 | 2561538876.c00025_CC96DRA...region1 | NRPS |
|  |  |  | CYP154M5 | 2561538881.c00030_CC96DRA...region1 | T1PKS |
|  |  |  | CYP105CT1 | 2561538881.c00030_CC96DRA...region1 | T1PKS |
|  |  |  | CYP105G5 | 2561538883.c00032_CC96DRA...region1 | T1PKS |
|  |  |  | CYP107Q4 | 2561538883.c00032_CC96DRA...region1 | T1PKS |
|  |  |  | CYP107FS2 | 2561538884.c00033_CC96DRA...region1 | NRPS |
|  |  |  | CYP105CN1 | 2561538887.c00036_CC96DRA...region1 | NRPS |
|  |  |  | CYP105CP2 | 2561538887.c00036_CC96DRA...region1 | NRPS |
|  |  |  | CYP247A7 | 2561538892.c00041_CC96DRA...region1 | other |
|  |  |  | CYP105W2 | 2561538896.c00045_CC96DRA...region1 | oligosaccharide |
|  |  |  | CYP248A2 | 2561538896.c00045_CC96DRA...region1 | oligosaccharide |
| *Salinispora pacifica* CNT138 | 2516143118 | 7 | CYP245A11 | 2516156462.c00001_B173DRA...region4 | NRPS |
|  |  |  | CYP107AW6 | 2516156462.c00001_B173DRA...region5 | Bacteriocin |
|  |  |  | CYP244A5 | 2516156462.c00001_B173DRA...region9 | T2PKS |
|  |  |  | CYP211C6 | 2516156462.c00001_B173DRA...region9 | T2PKS |
|  |  |  | CYP154M16 | 2516156462.c00001_B173DRA...region6 | NRPS |
|  |  |  | CYP107AY7 | 2516156476.c00015_B173DRA...region1 | terpene |
|  |  |  | CYP1278A4 | 2516156480.c00019_B173DRA...region1 | NRPS |
| *Salinispora pacifica* CNT001 | 2515154184 | 9 | CYP107AW7 | 2515168971.c00001_B169DRA...region2 | Bacteriocin |
|  |  |  | CYP208A22 | 2515168973.c00003_B169DRA...region2 | T1PKS |
|  |  |  | CYP161N4 | 2515168995.c00025_B169DRA...region1 | T3PKS |
|  |  |  | CYP2054A3 | 2515168995.c00025_B169DRA...region1 | T3PKS |
|  |  |  | CYP107FH3 | 2515168995.c00025_B169DRA...region1 | T3PKS |
|  |  |  | CYP107AY9 | 2515168997.c00027_B169DRA...region1 | terpene |
|  |  |  | CYP244A10 | 2515168997.c00027_B169DRA...region1 | terpene |
|  |  |  | CYP107E37 | 2515169026.c00056_B169DRA...region1 | T1PKS |
|  |  |  | CYP294A4 | 2515169033.c00063_B169DRA...region1 | T1PKS |
| *Salinispora pacifica* DSM 45547 | 2516493032 | 7 | CYP107AY7 | 2516493519.c00001_SALPAC4...region6 | NRPS |
|  |  |  | CYP244A5 | 2516493519.c00001_SALPAC4...region14 | NRPS |
|  |  |  | CYP245A11 | 2516493519.c00001_SALPAC4...region14 | NRPS |
|  |  |  | CYP107AW6 | 2516493519.c00001_SALPAC4...region15 | Bacteriocin |
|  |  |  | CYP154M16 | 2516493519.c00001_SALPAC4...region16 | NRPS |
|  |  |  | CYP211C6 | 2516493519.c00001_SALPAC4...region16 | NRPS |
|  |  |  | CYP1278A4 | 2516493519.c00001_SALPAC4...region22 | NRPS |
| *Salinispora pacifica* DSM 45548 | 2517287023 | 4 | CYP1278B4 | 2517287721.c00001_Salpac2...region1 | NRPS |
|  |  |  | CYP107AW9 | 2517287721.c00001_Salpac2...region3 | Bacteriocin |
|  |  |  | CYP211C4 | 2517287721.c00001_Salpac2...region6 | T2PKS |
|  |  |  | CYP1223A5 | 2517287721.c00001_Salpac2...region11 | lipolanthine |
| *Salinispora pacifica* CNH732 | 2571042006 | 9 | CYP107AW7 | 2571042892.c00004_T392DRA...region2 | Bacteriocin |
|  |  |  | CYP208A22 | 2571042893.c00005_T392DRA...region1 | T1PKS |
|  |  |  | CYP107FH3 | 2571042907.c00019_T392DRA...region1 | NRPS-like |
|  |  |  | CYP2054A3 | 2571042907.c00019_T392DRA...region1 | NRPS-like |
|  |  |  | CYP161N4 | 2571042907.c00019_T392DRA...region1 | NRPS-like |
|  |  |  | CYP107AY9 | 2571042909.c00021_T392DRA...region1 | terpene |
|  |  |  | CYP244A10 | 2571042909.c00021_T392DRA...region1 | terpene |
|  |  |  | CYP1278B-fragment2 | 2571042915.c00027_T392DRA...region1 | T1PKS |
|  |  |  | CYP107E37 | 2571042934.c00046_T392DRA...region1 | T1PKS |
| *Salinispora pacifica* CNT584 | 2517572160 | 11 | CYP154AJ2 | 2517596773.c00001_C582DRA...region1 | terpene |
|  |  |  | CYP211C9 | 2517596773.c00001_C582DRA...region2 | T2PKS |
|  |  |  | CYP244A5 | 2517596774.c00002_C582DRA...region1 | NRPS |
|  |  |  | CYP245A11 | 2517596774.c00002_C582DRA...region1 | NRPS |
|  |  |  | CYP107AW6 | 2517596774.c00002_C582DRA...region2 | Bacteriocin |
|  |  |  | CYP161T1 | 2517596782.c00010_C582DRA...region1 | T2PKS |
|  |  |  | CYP208A27 | 2517596785.c00013_C582DRA...region1 | T1PKS |
|  |  |  | CYP161N4 | 2517596793.c00021_C582DRA...region1 | NRPS-like |
|  |  |  | CYP2054A3 | 2517596793.c00021_C582DRA...region1 | NRPS-like |
|  |  |  | CYP107FH3 | 2517596793.c00021_C582DRA...region1 | NRPS-like |
|  |  |  | CYP107AY8 | 2517596807.c00035_C582DRA...region1 | NRPS |
| *Salinispora pacifica* CNS237 | 2524614807 | 14 | CYP211C7 | 2524706086.c00001_H303DRA...region1 | T2PKS |
|  |  |  | CYP285D2 | 2524706086.c00001_H303DRA...region3 | NRPS |
|  |  |  | CYP245A12 | 2524706086.c00001_H303DRA...region4 | indole |
|  |  |  | CYP244A5 | 2524706086.c00001_H303DRA...region4 | indole |
|  |  |  | CYP163B17 | 2524706086.c00001_H303DRA...region5 | transAT-PKS-like |
|  |  |  | CYP244A8 | 2524706087.c00002_H303DRA...region3 | NRPS |
|  |  |  | CYP107AY-fragment1 | 2524706087.c00002_H303DRA...region3 | NRPS |
|  |  |  | CYP107NE1 | 2524706091.c00006_H303DRA...region1 | T1PKS |
|  |  |  | CYP154M18 | 2524706091.c00006_H303DRA...region3 | NRPS |
|  |  |  | CYP208A28 | 2524706091.c00006_H303DRA...region3 | NRPS |
|  |  |  | CYP107AW4 | 2524706097.c00012_H303DRA...region2 | Bacteriocin |
|  |  |  | CYP113E2 | 2524706101.c00016_H303DRA...region1 | T1PKS |
|  |  |  | CYP107EP2 | 2524706101.c00016_H303DRA...region1 | T1PKS |
|  |  |  | CYP105AH4 | 2524706103.c00018_H303DRA...region1 | other |
| *Salinispora pacifica* CNT045 | 2517572158 | 12 | CYP154M16 | 2517596646.c00001_C554DRA...region1 | NRPS |
|  |  |  | CYP208A21 | 2517596646.c00001_C554DRA...region1 | NRPS |
|  |  |  | CYP107AW6 | 2517596646.c00001_C554DRA...region2 | Bacteriocin |
|  |  |  | CYP245A11 | 2517596646.c00001_C554DRA...region3 | indole |
|  |  |  | CYP244A5 | 2517596646.c00001_C554DRA...region3 | indole |
|  |  |  | CYP211C6 | 2517596647.c00002_C554DRA...region4 | T2PKS |
|  |  |  | CYP162A8 | 2517596652.c00007_C554DRA...region1 | NRPS-like |
|  |  |  | CYP107CL2 | 2517596656.c00011_C554DRA...region1 | NRPS |
|  |  |  | CYP1056B2 | 2517596656.c00011_C554DRA...region1 | NRPS |
|  |  |  | CYP163B20 | 2517596659.c00014_C554DRA...region1 | transAT-PKS-like |
|  |  |  | CYP107AY7 | 2517596665.c00020_C554DRA...region1 | NRPS |
|  |  |  | CYP107CT3 | 2517596667.c00022_C554DRA...region1 | NRPS |
| *Salinispora pacifica* CNY330 | 2518645626 | 9 | CYP107FH3 | 2518683698.c00002_C587DRA...region1 | NRPS-like |
|  |  |  | CYP2054A3 | 2518683698.c00002_C587DRA...region1 | NRPS-like |
|  |  |  | CYP161N4 | 2518683698.c00002_C587DRA...region1 | NRPS-like |
|  |  |  | CYP107AW7 | 2518683706.c00010_C587DRA...region2 | bacteriocin |
|  |  |  | CYP208A22 | 2518683716.c00020_C587DRA...region1 | T1PKS |
|  |  |  | CYP105H11 | 2518683723.c00027_C587DRA...region1 | T1PKS |
|  |  |  | CYP244A10 | 2518683730.c00034_C587DRA...region1 | NRPS |
|  |  |  | CYP107AY9 | 2518683730.c00034_C587DRA...region1 | NRPS |
|  |  |  | CYP107E37 | 2518683751.c00055_C587DRA...region1 | T1PKS |
| *Salinispora arenicola* CNH964 | 2515154125 | 1 | CYP208A12 | 2515164749.c00002_B164DRA...region2 | T1PKS |
| *Salinispora pacifica* CNT403 | 2561511034 | 12 | CYP244A5 | 2561515055.c00005_T427DRA...region1 | NRPS |
|  |  |  | CYP245A11 | 2561515055.c00005_T427DRA...region1 | NRPS |
|  |  |  | CYP107AW6 | 2561515055.c00005_T427DRA...region2 | Bacteriocin |
|  |  |  | CYP211C6 | 2561515058.c00008_T427DRA...region2 | T2PKS |
|  |  |  | CYP208A26 | 2561515061.c00011_T427DRA...region2 | T1PKS |
|  |  |  | CYP163B20 | 2561515063.c00013_T427DRA...region1 | transAT-PKS-like |
|  |  |  | CYP162A8 | 2561515066.c00016_T427DRA...region1 | NRPS-like |
|  |  |  | CYP1269A2 | 2561515068.c00018_T427DRA...region1 | oligosaccharide |
|  |  |  | CYP107AY7 | 2561515072.c00022_T427DRA...region1 | NRPS |
|  |  |  | CYP208A21 | 2561515087.c00037_T427DRA...region1 | NRPS |
|  |  |  | CYP154M16 | 2561515087.c00037_T427DRA...region1 | NRPS |
|  |  |  | CYP107CT3 | 2561515091.c00041_T427DRA...region1 | NRPS |
| *Salinispora pacifica* CNT-133 | 2548876971 | 1 | CYP163B20 | 2582697582.HQ215062.region1 | transAT-PKS-like |
| *Salinispora pacifica* CNQ768 | 2517572155 | 8 | CYP208A22 | 2517596374.c00003_C581DRA...region1 | T1PKS |
|  |  |  | CYP107AW7 | 2517596379.c00008_C581DRA...region3 | Bacteriocin |
|  |  |  | CYP107FH3 | 2517596392.c00021_C581DRA...region1 | NRPS-like |
|  |  |  | CYP2054A3 | 2517596392.c00021_C581DRA...region1 | NRPS-like |
|  |  |  | CYP161N4 | 2517596392.c00021_C581DRA...region1 | NRPS-like |
|  |  |  | CYP107AY9 | 2517596394.c00023_C581DRA...region1 | terpene |
|  |  |  | CYP244A10 | 2517596394.c00023_C581DRA...region1 | terpene |
|  |  |  | CYP107E37 | 2517596430.c00059_C581DRA...region1 | T1PKS |
| *Salinispora arenicola* CNS325 | 2571042009 | 19 | CYP208A12 | 2571043105.c00002_T371DRA...region2 | T1PKS |
|  |  |  | CYP107AY2 | 2571043107.c00004_T371DRA...region1 | NRPS |
|  |  |  | CYP105CT1 | 2571043107.c00004_T371DRA...region2 | terpene |
|  |  |  | CYP154M5 | 2571043107.c00004_T371DRA...region2 | terpene |
|  |  |  | CYP211C1 | 2571043108.c00005_T371DRA...region2 | T2PKS |
|  |  |  | CYP105CH1 | 2571043110.c00007_T371DRA...region2 | T1PKS |
|  |  |  | CYP244A4 | 2571043111.c00008_T371DRA...region1 | Indole |
|  |  |  | CYP245A7 | 2571043111.c00008_T371DRA...region1 | Indole |
|  |  |  | CYP166A4 | 2571043112.c00009_T371DRA...region2 | T1PKS |
|  |  |  | CYP105W2 | 2571043114.c00011_T371DRA...region1 | oligosaccharide |
|  |  |  | CYP248A2 | 2571043114.c00011_T371DRA...region1 | oligosaccharide |
|  |  |  | CYP107FS2 | 2571043114.c00011_T371DRA...region1 | oligosaccharide |
|  |  |  | CYP105CN1 | 2571043118.c00015_T371DRA...region1 | T1PKS |
|  |  |  | CYP105CP2 | 2571043114.c00011_T371DRA...region1 | oligosaccharide |
|  |  |  | CYP105BN4 | 2571043118.c00015_T371DRA...region1 | T1PKS |
|  |  |  | CYP107Q4 | 2571043120.c00017_T371DRA...region1 | T1PKS |
|  |  |  | CYP105G5 | 2571043120.c00017_T371DRA...region1 | T1PKS |
|  |  |  | CYP1051A1 | 2571043120.c00017_T371DRA...region2 | Terpene |
|  |  |  | CYP107EU1 | 2571043135.c00032_T371DRA...region1 | NRPS-like |
| *Salinispora arenicola* CNS296 | 2565956527 | 19 | CYP105CH1 | 2565959858.c00001_T319DRA...region2 | T1PKS |
|  |  |  | CYP107Q4 | 2565959861.c00004_T319DRA...region1 | T1PKS |
|  |  |  | CYP105G5 | 2565959861.c00004_T319DRA...region1 | T1PKS |
|  |  |  | CYP1051A1 | 2565959861.c00004_T319DRA...region2 | Terpene |
|  |  |  | CYP107AY2-fragment | 2565959862.c00005_T319DRA...region1 | NRPS |
|  |  |  | CYP105CT1 | 2565959862.c00005_T319DRA...region2 | butyrolactone |
|  |  |  | CYP154M5 | 2565959862.c00005_T319DRA...region2 | butyrolactone |
|  |  |  | CYP208A12 | 2565959863.c00006_T319DRA...region2 | T1PKS |
|  |  |  | CYP244A4 | 2565959865.c00008_T319DRA...region1 | Indole |
|  |  |  | CYP245A7 | 2565959865.c00008_T319DRA...region1 | Indole |
|  |  |  | CYP166A4 | 2565959867.c00010_T319DRA...region2 | T1PKS |
|  |  |  | CYP105CP2 | 2565959868.c00011_T319DRA...region2 | NRPS |
|  |  |  | CYP105CN1 | 2565959868.c00011_T319DRA...region2 | NRPS |
|  |  |  | CYP107FS2 | 2565959868.c00011_T319DRA...region2 | NRPS |
|  |  |  | CYP248A2 | 2565959868.c00011_T319DRA...region2 | NRPS |
|  |  |  | CYP105W2 | 2565959868.c00011_T319DRA...region2 | NRPS |
|  |  |  | CYP211C1 | 2565959873.c00016_T319DRA...region1 | T2PKS |
|  |  |  | CYP247A7 | 2565959874.c00017_T319DRA...region1 | other |
|  |  |  | CYP107EU1 | 2565959885.c00028_T319DRA...region1 | T1PKS |
| *Salinispora pacifica* DSM 45549 | 2517434008 | 4 | CYP105AH4 | 2517434628.c00001_Salpac6...region1 | other |
|  |  |  | CYP245A13 | 2517434628.c00001_Salpac6...region2 | Indole |
|  |  |  | CYP244A5 | 2517434628.c00001_Salpac6...region2 | Indole |
|  |  |  | CYP113E2 | 2517434628.c00001_Salpac6...region3 | T1PKS |
| *Salinispora arenicola* CNY231 | 2518285558 | 3 | CYP166A4 | 2518290717.c00001_C577DRA...region1 | T1PKS |
|  |  |  | CYP211C1 | 2518290720.c00004_C577DRA...region1 | T2PKS |
|  |  |  | CYP244A4 | 2518290721.c00005_C577DRA...region1 | Indole |
| *Salinispora arenicola* CNS744 | 2518285554 | 10 | CYP166A4 | 2518290334.c00002_C573DRA...region1 | T1PKS |
|  |  |  | CYP211C1 | 2518290335.c00003_C573DRA...region4 | T2PKS |
|  |  |  | CYP244A4 | 2518290338.c00006_C573DRA...region1 | Indole |
|  |  |  | CYP245A7 | 2518290338.c00006_C573DRA...region1 | Indole |
|  |  |  | CYP107AY2 | 2518290339.c00007_C573DRA...region1 | NRPS |
|  |  |  | CYP105CP2 | 2518290341.c00009_C573DRA...region2 | NRPS |
|  |  |  | CYP105CN1 | 2518290341.c00009_C573DRA...region2 | NRPS |
|  |  |  | CYP107FS2 | 2518290341.c00009_C573DRA...region2 | NRPS |
|  |  |  | CYP248A2 | 2518290341.c00009_C573DRA...region2 | NRPS |
|  |  |  | CYP105W2 | 2518290341.c00009_C573DRA...region2 | NRPS |
| *Salinispora tropica* CNB-476 | 651717007 | 1 | CYP163B1 | 651717006.EF397502.region1 | NRPS |
| *Salinispora arenicola* CNS342 | 2571042016 | 17 | CYP208A12 | 2571043981.c00002_T366DRA...region2 | T1PKS |
|  |  |  | CYP211C1 | 2571043982.c00003_T366DRA...region4 | T2PKS |
|  |  |  | CYP105W2 | 2571043986.c00007_T366DRA...region1 | oligosaccharide |
|  |  |  | CYP248A2 | 2571043986.c00007_T366DRA...region1 | oligosaccharide |
|  |  |  | CYP107FS2 | 2571043986.c00007_T366DRA...region1 | oligosaccharide |
|  |  |  | CYP105CN1 | 2571043986.c00007_T366DRA...region1 | oligosaccharide |
|  |  |  | CYP105CP2 | 2571043986.c00007_T366DRA...region1 | oligosaccharide |
|  |  |  | CYP244A4 | 2571043987.c00008_T366DRA...region1 | Indole |
|  |  |  | CYP245A7 | 2571043987.c00008_T366DRA...region1 | Indole |
|  |  |  | CYP166A4 | 2571043988.c00009_T366DRA...region1 | T1PKS |
|  |  |  | CYP154M5 | 2571043989.c00010_T366DRA...region1 | T1PKS |
|  |  |  | CYP105CT1 | 2571043989.c00010_T366DRA...region1 | T1PKS |
|  |  |  | CYP107AY2-fragment | 2571043989.c00010_T366DRA...region1 | T1PKS |
|  |  |  | CYP107Q4 | 2571043991.c00012_T366DRA...region1 | T1PKS |
|  |  |  | CYP105G5 | 2571043991.c00012_T366DRA...region1 | T1PKS |
|  |  |  | CYP1051A1 | 2571043991.c00012_T366DRA...region2 | Terpene |
|  |  |  | CYP105CH1 | 2571043997.c00018_T366DRA...region1 | T1PKS |
| *Salinispora pacifica* CNT796 | 2515154182 | 9 | CYP161N4 | 2515168768.c00002_B120DRA...region1 | T3PKS |
|  |  |  | CYP2054A3 | 2515168768.c00002_B120DRA...region1 | T3PKS |
|  |  |  | CYP107FH3 | 2515168768.c00002_B120DRA...region1 | T3PKS |
|  |  |  | CYP107AW7 | 2515168769.c00003_B120DRA...region2 | Bacteriocin |
|  |  |  | CYP208A22 | 2515168792.c00026_B120DRA...region1 | T1PKS |
|  |  |  | CYP244A13 | 2515168803.c00037_B120DRA...region1 | NRPS |
|  |  |  | CYP107AY9 | 2515168803.c00037_B120DRA...region1 | NRPS |
|  |  |  | CYP107NG1 | 2515168817.c00051_B120DRA...region1 | T2PKS |
|  |  |  | CYP107E37 | 2515168821.c00055_B120DRA...region1 | T1PKS |
| *Salinispora pacifica* CNR114 | 2515154178 | 11 | CYP107AW7 | 2515168496.c00002_B118DRA...region2 | Bacteriocin |
|  |  |  | CYP208A22 | 2515168497.c00003_B118DRA...region2 | T1PKS |
|  |  |  | CYP161N4 | 2515168517.c00023_B118DRA...region1 | NRPS-like |
|  |  |  | CYP2054A3 | 2515168517.c00023_B118DRA...region1 | NRPS-like |
|  |  |  | CYP107FH3 | 2515168517.c00023_B118DRA...region1 | NRPS-like |
|  |  |  | CYP1437C1 | 2515168521.c00027_B118DRA...region1 | NRPS |
|  |  |  | CYP163A10 | 2515168528.c00034_B118DRA...region1 | NRPS |
|  |  |  | CYP162K1 | 2515168528.c00034_B118DRA...region1 | NRPS |
|  |  |  | CYP107AY9 | 2515168530.c00036_B118DRA...region1 | NRPS |
|  |  |  | CYP294A4 | 2515168548.c00054_B118DRA...region1 | T1PKS |
|  |  |  | CYP107E37 | 2515168552.c00058_B118DRA...region1 | T1PKS |
| *Salinispora arenicola* CNY282 | 2521172655 | 7 | CYP211C1 | 2521193357.c00003_F585DRA...region1 | T2PKS |
|  |  |  | CYP107AY2 | 2521193358.c00004_F585DRA...region1 | NRPS |
|  |  |  | CYP105CT1 | 2521193358.c00004_F585DRA...region1 | NRPS |
|  |  |  | CYP154M5 | 2521193358.c00004_F585DRA...region1 | NRPS |
|  |  |  | CYP245A7 | 2521193362.c00008_F585DRA...region1 | Indole |
|  |  |  | CYP244A4 | 2521193362.c00008_F585DRA...region1 | Indole |
|  |  |  | CYP208A12 | 2521193364.c00010_F585DRA...region1 | T1PKS |
| *Salinispora arenicola* CNT798 | 2515154186 | 21 | CYP244A4 | 2515169136.c00007_B103DRA...region1 | Indole |
|  |  |  | CYP245A7 | 2515169136.c00007_B103DRA...region1 | Indole |
|  |  |  | CYP105CP2 | 2515169137.c00008_B103DRA...region2 | NRPS |
|  |  |  | CYP105CN1 | 2515169137.c00008_B103DRA...region2 | NRPS |
|  |  |  | CYP107FS2 | 2515169137.c00008_B103DRA...region2 | NRPS |
|  |  |  | CYP125G6 | 2515169137.c00008_B103DRA...region2 | NRPS |
|  |  |  | CYP154M15 | 2515169137.c00008_B103DRA...region2 | NRPS |
|  |  |  | CYP107AY2 | 2515169138.c00009_B103DRA...region1 | NRPS |
|  |  |  | CYP208A12 | 2515169139.c00010_B103DRA...region1 | T1PKS |
|  |  |  | CYP105CH1 | 2515169141.c00012_B103DRA...region2 | T1PKS |
|  |  |  | CYP107Q4 | 2515169143.c00014_B103DRA...region1 | T1PKS |
|  |  |  | CYP105G5 | 2515169143.c00014_B103DRA...region1 | T1PKS |
|  |  |  | CYP1051A1 | 2515169143.c00014_B103DRA...region2 | Terpene |
|  |  |  | CYP211C1 | 2515169144.c00015_B103DRA...region1 | T2PKS |
|  |  |  | CYP166A4 | 2515169145.c00016_B103DRA...region1 | T1PKS |
|  |  |  | CYP107AX13 | 2515169146.c00017_B103DRA...region1 | ladderane |
|  |  |  | CYP154M20 | 2515169150.c00021_B103DRA...region1 | oligosaccharide |
|  |  |  | CYP248A2 | 2515169150.c00021_B103DRA...region1 | oligosaccharide |
|  |  |  | CYP105W2 | 2515169150.c00021_B103DRA...region1 | oligosaccharide |
|  |  |  | CYP154M13 | 2515169150.c00021_B103DRA...region1 | oligosaccharide |
|  |  |  | CYP154M21 | 2515169150.c00021_B103DRA...region1 | oligosaccharide |
| *Salinispora pacifica* CNS860 | 2518285563 | 10 | CYP211C6 | 2518291047.c00006_C551DRA...region2 | T2PKS |
|  |  |  | CYP244A5 | 2518291050.c00009_C551DRA...region1 | indole |
|  |  |  | CYP107CT3 | 2518291051.c00010_C551DRA...region1 | NRPS |
|  |  |  | CYP163B20 | 2518291054.c00013_C551DRA...region1 | NRPS |
|  |  |  | CYP208A26 | 2518291055.c00014_C551DRA...region1 | T1PKS |
|  |  |  | CYP154M16 | 2518291056.c00015_C551DRA...region1 | NRPS |
|  |  |  | CYP208A21 | 2518291056.c00015_C551DRA...region1 | NRPS |
|  |  |  | CYP107AW6 | 2518291056.c00015_C551DRA...region2 | Bacteriocin |
|  |  |  | CYP107AY7 | 2518291057.c00016_C551DRA...region1 | terpene |
|  |  |  | CYP162A8 | 2518291067.c00026_C551DRA...region1 | NRPS-like |
| *Salinispora arenicola* CNY694 | 2561511114 | 23 | CYP154M5 | 2561539735.c00002_T378DRA...region1 | T1PKS |
|  |  |  | CYP105CT2 | 2561539735.c00002_T378DRA...region1 | T1PKS |
|  |  |  | CYP211C1 | 2561539736.c00003_T378DRA...region2 | T2PKS |
|  |  |  | CYP107AX-fragment8 | 2561539738.c00005_T378DRA...region1 | ladderane |
|  |  |  | CYP166A4 | 2561539738.c00005_T378DRA...region2 | NRPS-like |
|  |  |  | CYP208A12 | 2561539742.c00009_T378DRA...region1 | T1PKS |
|  |  |  | CYP105W3 | 2561539744.c00011_T378DRA...region1 | oligosaccharide |
|  |  |  | CYP248A2 | 2561539744.c00011_T378DRA...region1 | oligosaccharide |
|  |  |  | CYP107FS2 | 2561539744.c00011_T378DRA...region1 | oligosaccharide |
|  |  |  | CYP105CN1 | 2561539744.c00011_T378DRA...region1 | oligosaccharide |
|  |  |  | CYP105CP2 | 2561539744.c00011_T378DRA...region1 | oligosaccharide |
|  |  |  | CYP1051A4 | 2561539746.c00013_T378DRA...region1 | Terpene |
|  |  |  | CYP244A4 | 2561539748.c00015_T378DRA...region1 | Indole |
|  |  |  | CYP245A7 | 2561539748.c00015_T378DRA...region1 | Indole |
|  |  |  | CYP244A-fragment2 | 2561539752.c00019_T378DRA...region1 | NRPS |
|  |  |  | CYP107EU2 | 2561539763.c00030_T378DRA...region1 | NRPS-like |
|  |  |  | CYP107Q4 | 2561539765.c00032_T378DRA...region1 | T1PKS |
|  |  |  | CYP105G7 | 2561539765.c00032_T378DRA...region1 | T1PKS |
|  |  |  | CYP105CH2 | 2561539772.c00039_T378DRA...region1 | T1PKS |
|  |  |  | CYP285A9-fragment | 2561539788.c00055_T378DRA...region1 | NRPS-like |
|  |  |  | CYP285A9-fragment | 2561539788.c00055_T378DRA...region1 | NRPS-like |
|  |  |  | CYP107AW7 | 2561539796.c00063_T378DRA...region1 | Bacteriocin |
|  |  |  | CYP1198B2 | 2561539809.c00076_T378DRA...region1 | T1PKS |
| *Salinispora arenicola* CNP193 | 2518285552 | 19 | CYP105CP2 | 2518290163.c00003_C571DRA...region2 | NRPS |
|  |  |  | CYP105CN1 | 2518290163.c00003_C571DRA...region2 | NRPS |
|  |  |  | CYP107FS2 | 2518290163.c00003_C571DRA...region2 | NRPS |
|  |  |  | CYP125G6 | 2518290163.c00003_C571DRA...region2 | NRPS |
|  |  |  | CYP154M15 | 2518290163.c00003_C571DRA...region2 | NRPS |
|  |  |  | CYP154M20 | 2518290163.c00003_C571DRA...region3 | oligosaccharide |
|  |  |  | CYP248A2 | 2518290163.c00003_C571DRA...region3 | oligosaccharide |
|  |  |  | CYP105W2 | 2518290163.c00003_C571DRA...region3 | oligosaccharide |
|  |  |  | CYP154M13 | 2518290163.c00003_C571DRA...region3 | oligosaccharide |
|  |  |  | CYP154M21 | 2518290163.c00003_C571DRA...region3 | oligosaccharide |
|  |  |  | CYP208A12 | 2518290164.c00004_C571DRA...region1 | T1PKS |
|  |  |  | CYP245A7 | 2518290166.c00006_C571DRA...region1 | Indole |
|  |  |  | CYP244A4 | 2518290166.c00006_C571DRA...region1 | Indole |
|  |  |  | CYP166A4 | 2518290171.c00011_C571DRA...region1 | T1PKS |
|  |  |  | CYP1051A1 | 2518290172.c00012_C571DRA...region1 | Terpene |
|  |  |  | CYP105G5 | 2518290172.c00012_C571DRA...region2 | T1PKS |
|  |  |  | CYP107Q4 | 2518290172.c00012_C571DRA...region2 | T1PKS |
|  |  |  | CYP107AY2 | 2518290192.c00032_C571DRA...region1 | NRPS |
|  |  |  | CYP163B16 | 2518290193.c00033_C571DRA...region1 | NRPS |
| *Salinispora pacifica* CNS103 | 2515154129 | 5 | CYP107AW7 | 2515165134.c00004_B117DRA...region2 | Bacteriocin |
|  |  |  | CYP107FH3 | 2515165152.c00022_B117DRA...region1 | NRPS-like |
|  |  |  | CYP161N4 | 2515165152.c00022_B117DRA...region1 | NRPS-like |
|  |  |  | CYP107AY9 | 2515165152.c00022_B117DRA...region1 | NRPS-like |
|  |  |  | CYP107E37 | 2515165153.c00023_B117DRA...region1 | terpene |
| *Salinispora arenicola* CNP105 | 2518285551 | 1 | CYP208A12 | 2518290094.c00003_C572DRA...region2 | T1PKS |
| *Salinispora arenicola* CNX482 | 2515154137 | 16 | CYP245A7 | 2515165687.c00004_B168DRA...region2 | Indole |
|  |  |  | CYP244A4 | 2515165687.c00004_B168DRA...region2 | Indole |
|  |  |  | CYP208A12 | 2515165688.c00005_B168DRA...region1 | T1PKS |
|  |  |  | CYP154M5 | 2515165690.c00007_B168DRA...region1 | T1PKS |
|  |  |  | CYP105CT1 | 2515165690.c00007_B168DRA...region1 | T1PKS |
|  |  |  | CYP107Q4 | 2515165694.c00011_B168DRA...region1 | T1PKS |
|  |  |  | CYP105G5 | 2515165694.c00011_B168DRA...region1 | T1PKS |
|  |  |  | CYP1051A1 | 2515165694.c00011_B168DRA...region2 | Terpene |
|  |  |  | CYP211C1 | 2515165696.c00013_B168DRA...region1 | T2PKS |
|  |  |  | CYP107FS2 | 2515165698.c00015_B168DRA...region1 | NRPS |
|  |  |  | CYP105CN1 | 2515165698.c00015_B168DRA...region1 | NRPS |
|  |  |  | CYP105CP2 | 2515165698.c00015_B168DRA...region1 | NRPS |
|  |  |  | CYP105W2 | 2515165699.c00016_B168DRA...region1 | oligosaccharide |
|  |  |  | CYP248A2 | 2515165699.c00016_B168DRA...region1 | oligosaccharide |
|  |  |  | CYP166A4 | 2515165701.c00018_B168DRA...region1 | T1PKS |
|  |  |  | CYP107AY2-fragment | 2515165702.c00019_B168DRA...region1 | NRPS |
| *Salinispora arenicola* CNS299 | 2524614529 | 18 | CYP211C1 | 2524618012.c00001_H279DRA...region4 | T2PKS |
|  |  |  | CYP208A12 | 2524618014.c00003_H279DRA...region2 | T1PKS |
|  |  |  | CYP105W2 | 2524618017.c00006_H279DRA...region1 | oligosaccharide |
|  |  |  | CYP248A2 | 2524618017.c00006_H279DRA...region1 | oligosaccharide |
|  |  |  | CYP107FS2 | 2524618017.c00006_H279DRA...region1 | oligosaccharide |
|  |  |  | CYP105CN1 | 2524618017.c00006_H279DRA...region1 | oligosaccharide |
|  |  |  | CYP105CP2 | 2524618017.c00006_H279DRA...region1 | oligosaccharide |
|  |  |  | CYP244A4 | 2524618019.c00008_H279DRA...region1 | Indole |
|  |  |  | CYP245A7 | 2524618019.c00008_H279DRA...region1 | Indole |
|  |  |  | CYP166A4 | 2524618020.c00009_H279DRA...region1 | NRPS-like |
|  |  |  | CYP1051A1 | 2524618024.c00013_H279DRA...region1 | Terpene |
|  |  |  | CYP105G5 | 2524618024.c00013_H279DRA...region2 | T1PKS |
|  |  |  | CYP107Q4 | 2524618024.c00013_H279DRA...region2 | T1PKS |
|  |  |  | CYP105CT1 | 2524618025.c00014_H279DRA...region1 | terpene |
|  |  |  | CYP154M5 | 2524618025.c00014_H279DRA...region1 | terpene |
|  |  |  | CYP107AY2-fragment | 2524618030.c00019_H279DRA...region1 | NRPS |
|  |  |  | CYP247A7 | 2524618033.c00022_H279DRA...region1 | NRPS |
|  |  |  | CYP105CH1 | 2524618035.c00024_H279DRA...region1 | T1PKS |
| *Salinispora pacifica* CNT003 | 2515154126 | 10 | CYP208A22 | 2515164822.c00005_B167DRA...region1 | T1PKS |
|  |  |  | CYP107AW7 | 2515164823.c00006_B167DRA...region1 | Bacteriocin |
|  |  |  | CYP107FH3 | 2515164831.c00014_B167DRA...region1 | NRPS-like |
|  |  |  | CYP2054A3 | 2515164831.c00014_B167DRA...region1 | NRPS-like |
|  |  |  | CYP161N4 | 2515164831.c00014_B167DRA...region1 | NRPS-like |
|  |  |  | CYP107AY9 | 2515164850.c00033_B167DRA...region1 | NRPS |
|  |  |  | CYP162B3 | 2515164854.c00037_B167DRA...region1 | NRPS |
|  |  |  | CYP162G1 | 2515164867.c00050_B167DRA...region1 | NRPS |
|  |  |  | CYP107E37 | 2515164880.c00063_B167DRA...region1 | T1PKS |
|  |  |  | CYP107FH4 | 2515164904.c00087_B167DRA...region1 | T1PKS |
| *Salinispora pacifica* CNT569 | 2515154124 | 7 | CYP211C5 | 2515164709.c00001_B116DRA...region2 | T1PKS |
|  |  |  | CYP125G4 | 2515164709.c00001_B116DRA...region4 | T2PKS |
|  |  |  | CYP1004A3 | 2515164716.c00008_B116DRA...region1 | other |
|  |  |  | CYP1004B4 | 2515164716.c00008_B116DRA...region1 | other |
|  |  |  | CYP113E2 | 2515164716.c00008_B116DRA...region1 | other |
|  |  |  | CYP163B18 | 2515164716.c00008_B116DRA...region1 | other |
|  |  |  | CYP107AY3 | 2515164718.c00010_B116DRA...region2 | NRPS |
| *Salinispora arenicola* CNH643 | 2561511037 | 22 | CYP107Q4 | 2561515316.c00001_T339DRA...region1 | T1PKS |
|  |  |  | CYP105G5 | 2561515316.c00001_T339DRA...region1 | T1PKS |
|  |  |  | CYP1051A1 | 2561515316.c00001_T339DRA...region2 | Terpene |
|  |  |  | CYP211C1 | 2561515317.c00002_T339DRA...region5 | T2PKS |
|  |  |  | CYP208A12 | 2561515318.c00003_T339DRA...region1 | T1PKS |
|  |  |  | CYP244A4 | 2561515323.c00008_T339DRA...region1 | Indole |
|  |  |  | CYP245A7 | 2561515323.c00008_T339DRA...region1 | Indole |
|  |  |  | CYP105CP2 | 2561515324.c00009_T339DRA...region2 | NRPS |
|  |  |  | CYP105CN1 | 2561515324.c00009_T339DRA...region2 | NRPS |
|  |  |  | CYP107FS2 | 2561515324.c00009_T339DRA...region2 | NRPS |
|  |  |  | CYP125G6 | 2561515324.c00009_T339DRA...region2 | NRPS |
|  |  |  | CYP154M15 | 2561515324.c00009_T339DRA...region2 | NRPS |
|  |  |  | CYP166A4 | 2561515326.c00011_T339DRA...region1 | T1PKS |
|  |  |  | CYP107AX14P | 2561515329.c00014_T339DRA...region1 | ladderane |
|  |  |  | CYP107EU1 | 2561515333.c00018_T339DRA...region2 | NRPS-like |
|  |  |  | CYP162P1 | 2561515337.c00022_T339DRA...region1 | NRPS |
|  |  |  | CYP154M21 | 2561515338.c00023_T339DRA...region1 | NRPS |
|  |  |  | CYP154M13 | 2561515338.c00023_T339DRA...region1 | NRPS |
|  |  |  | CYP105W2 | 2561515338.c00023_T339DRA...region1 | NRPS |
|  |  |  | CYP248A2 | 2561515338.c00023_T339DRA...region1 | NRPS |
|  |  |  | CYP154M20 | 2561515338.c00023_T339DRA...region1 | NRPS |
|  |  |  | CYP107AY13 | 2561515348.c00033_T339DRA...region1 | NRPS |
| *Salinispora arenicola* CNH962 | 2519103193 | 17 | CYP211C1 | 2519123975.c00005_F582DRA...region2 | T2PKS |
|  |  |  | CYP244A4 | 2519123976.c00006_F582DRA...region1 | Indole |
|  |  |  | CYP245A7 | 2519123976.c00006_F582DRA...region1 | Indole |
|  |  |  | CYP107AX-fragment9 | 2519123978.c00008_F582DRA...region1 | ladderane |
|  |  |  | CYP166A4 | 2519123978.c00008_F582DRA...region2 | T1PKS |
|  |  |  | CYP154M14 | 2519123979.c00009_F582DRA...region2 | NRPS |
|  |  |  | CYP107FS2 | 2519123985.c00015_F582DRA...region1 | NRPS |
|  |  |  | CYP105CN1 | 2519123985.c00015_F582DRA...region1 | NRPS |
|  |  |  | CYP105CP2 | 2519123985.c00015_F582DRA...region1 | NRPS |
|  |  |  | CYP107AY2 | 2519123992.c00022_F582DRA...region1 | NRPS |
|  |  |  | CYP208A12 | 2519123993.c00023_F582DRA...region1 | T1PKS |
|  |  |  | CYP107Q4 | 2519123996.c00026_F582DRA...region1 | T1PKS |
|  |  |  | CYP105G5 | 2519123996.c00026_F582DRA...region1 | T1PKS |
|  |  |  | CYP1051A1 | 2519123996.c00026_F582DRA...region2 | Terpene |
|  |  |  | CYP105CT1 | 2519124007.c00037_F582DRA...region1 | Terpene |
|  |  |  | CYP105W2 | 2519124009.c00039_F582DRA...region1 | oligosaccharide |
|  |  |  | CYP248A2 | 2519124009.c00039_F582DRA...region1 | oligosaccharide |
| *Salinispora arenicola* CNX481 | 2518285555 | 18 | CYP211C1 | 2518290422.c00001_C574DRA...region1 | T2PKS |
|  |  |  | CYP245A7 | 2518290425.c00004_C574DRA...region2 | Indole |
|  |  |  | CYP244A4 | 2518290425.c00004_C574DRA...region2 | Indole |
|  |  |  | CYP166A4 | 2518290426.c00005_C574DRA...region1 | T1PKS |
|  |  |  | CYP105CP2 | 2518290430.c00009_C574DRA...region2 | NRPS |
|  |  |  | CYP105CN1 | 2518290430.c00009_C574DRA...region2 | NRPS |
|  |  |  | CYP107FS2 | 2518290430.c00009_C574DRA...region2 | NRPS |
|  |  |  | CYP1051A1 | 2518290431.c00010_C574DRA...region1 | Terpene |
|  |  |  | CYP105G5 | 2518290431.c00010_C574DRA...region2 | T1PKS |
|  |  |  | CYP107Q4 | 2518290431.c00010_C574DRA...region2 | T1PKS |
|  |  |  | CYP208A12 | 2518290433.c00012_C574DRA...region1 | T1PKS |
|  |  |  | CYP107AY2 | 2518290440.c00019_C574DRA...region1 | NRPS |
|  |  |  | CYP105CH2-fragment | 2518290443.c00022_C574DRA...region1 | NRPS |
|  |  |  | CYP105CH1-fragment | 2518290443.c00022_C574DRA...region1 | NRPS |
|  |  |  | CYP248A2 | 2518290449.c00028_C574DRA...region1 | oligosaccharide |
|  |  |  | CYP105W2 | 2518290449.c00028_C574DRA...region1 | oligosaccharide |
|  |  |  | CYP154M5 | 2518290452.c00031_C574DRA...region1 | T1PKS |
|  |  |  | CYP105CT1 | 2518290452.c00031_C574DRA...region1 | T1PKS |
| *Salinispora pacifica* CNT148 | 2517572145 | 2 | CYP107AW9 | 2517595798.c00002_B159DRA...region2 | Bacteriocin |
|  |  |  | CYP1223A5 | 2517595810.c00014_B159DRA...region1 | lanthipeptide |
| *Salinispora arenicola* CNH713 | 2571042007 | 22 | CYP211C1 | 2571042957.c00001_T341DRA...region3 | T2PKS |
|  |  |  | CYP166A4 | 2571042957.c00001_T341DRA...region7 | NRPS-like |
|  |  |  | CYP105CH1 | 2571042958.c00002_T341DRA...region1 | T1PKS |
|  |  |  | CYP162P1 | 2571042958.c00002_T341DRA...region3 | NRPS |
|  |  |  | CYP208A12 | 2571042962.c00006_T341DRA...region1 | T1PKS |
|  |  |  | CYP105CP2 | 2571042963.c00007_T341DRA...region2 | NRPS |
|  |  |  | CYP105CN1 | 2571042963.c00007_T341DRA...region2 | NRPS |
|  |  |  | CYP107FS2 | 2571042963.c00007_T341DRA...region2 | NRPS |
|  |  |  | CYP125G6 | 2571042963.c00007_T341DRA...region2 | NRPS |
|  |  |  | CYP154M15 | 2571042963.c00007_T341DRA...region2 | NRPS |
|  |  |  | CYP154M20 | 2571042963.c00007_T341DRA...region3 | oligosaccharide |
|  |  |  | CYP248A2 | 2571042963.c00007_T341DRA...region3 | oligosaccharide |
|  |  |  | CYP105W2 | 2571042963.c00007_T341DRA...region3 | oligosaccharide |
|  |  |  | CYP154M13 | 2571042963.c00007_T341DRA...region3 | oligosaccharide |
|  |  |  | CYP154M21 | 2571042963.c00007_T341DRA...region3 | oligosaccharide |
|  |  |  | CYP107Q4 | 2571042964.c00008_T341DRA...region1 | T1PKS |
|  |  |  | CYP105G5 | 2571042964.c00008_T341DRA...region1 | T1PKS |
|  |  |  | CYP1051A1 | 2571042964.c00008_T341DRA...region2 | Terpene |
|  |  |  | CYP244A4 | 2571042965.c00009_T341DRA...region1 | Indole |
|  |  |  | CYP245A7 | 2571042965.c00009_T341DRA...region1 | Indole |
|  |  |  | CYP107EU1 | 2571042966.c00010_T341DRA...region1 | T1PKS |
|  |  |  | CYP107AY2 | 2571042967.c00011_T341DRA...region1 | NRPS |
| *Salinispora arenicola* CNH963 | 2524023246 | 17 | CYP107AX-fragment9 | 2524041079.c00004_H278DRA...region1 | ladderane |
|  |  |  | CYP166A4 | 2524041079.c00004_H278DRA...region2 | NRPS-like |
|  |  |  | CYP107Q4 | 2524041079.c00004_H278DRA...region2 | NRPS-like |
|  |  |  | CYP105G5 | 2524041079.c00004_H278DRA...region2 | NRPS-like |
|  |  |  | CYP1051A1 | 2524041079.c00004_H278DRA...region3 | Terpene |
|  |  |  | CYP211C1 | 2524041081.c00006_H278DRA...region1 | T2PKS |
|  |  |  | CYP245A7 | 2524041083.c00008_H278DRA...region1 | Indole |
|  |  |  | CYP244A4 | 2524041083.c00008_H278DRA...region1 | Indole |
|  |  |  | CYP154M14 | 2524041084.c00009_H278DRA...region2 | NRPS |
|  |  |  | CYP105CP2 | 2524041089.c00014_H278DRA...region2 | NRPS |
|  |  |  | CYP105CN1 | 2524041089.c00014_H278DRA...region2 | NRPS |
|  |  |  | CYP107FS2 | 2524041089.c00014_H278DRA...region2 | NRPS |
|  |  |  | CYP208A12 | 2524041096.c00021_H278DRA...region1 | T1PKS |
|  |  |  | CYP107AY2 | 2524041100.c00025_H278DRA...region1 | NRPS |
|  |  |  | CYP105CT1 | 2524041114.c00039_H278DRA...region1 | Terpene |
|  |  |  | CYP248A2 | 2524041115.c00040_H278DRA...region1 | oligosaccharide |
|  |  |  | CYP105W2 | 2524041115.c00040_H278DRA...region1 | oligosaccharide |
| *Salinispora pacifica* CNS863 | 2517572156 | 11 | CYP162A8 | 2517596463.c00001_C552DRA...region2 | NRPS-like |
|  |  |  | CYP211C6 | 2517596468.c00006_C552DRA...region2 | T2PKS |
|  |  |  | CYP245A11 | 2517596470.c00008_C552DRA...region1 | indole |
|  |  |  | CYP244A5 | 2517596470.c00008_C552DRA...region1 | indole |
|  |  |  | CYP208A26 | 2517596472.c00010_C552DRA...region1 | T1PKS |
|  |  |  | CYP107AW6 | 2517596477.c00015_C552DRA...region1 | Bacteriocin |
|  |  |  | CYP163B20 | 2517596484.c00022_C552DRA...region1 | NRPS |
|  |  |  | CYP107AY7 | 2517596488.c00026_C552DRA...region1 | terpene |
|  |  |  | CYP208A21 | 2517596493.c00031_C552DRA...region1 | NRPS |
|  |  |  | CYP154M16 | 2517596493.c00031_C552DRA...region1 | NRPS |
|  |  |  | CYP107CT3 | 2517596497.c00035_C552DRA...region1 | NRPS |
| *Salinispora tropica* CNB476 | 2517572211 | 10 | CYP163B1 | 2517600244.c00001_B098DRA...region1 | NRPS |
|  |  |  | CYP154M1 | 2517600245.c00002_B098DRA...region1 | NRPS |
|  |  |  | CYP208A4 | 2517600245.c00002_B098DRA...region1 | NRPS |
|  |  |  | CYP211C1 | 2517600245.c00002_B098DRA...region3 | T2PKS |
|  |  |  | CYP107AW1 | 2517600246.c00003_B098DRA...region2 | Bacteriocin |
|  |  |  | CYP1004B1 | 2517600258.c00015_B098DRA...region1 | NRPS |
|  |  |  | CYP1004A1 | 2517600258.c00015_B098DRA...region1 | NRPS |
|  |  |  | CYP125G1 | 2517600258.c00015_B098DRA...region1 | NRPS |
|  |  |  | CYP107AY1 | 2517600260.c00017_B098DRA...region1 | terpene |
|  |  |  | CYP107E3 | 2517600285.c00042_B098DRA...region1 | T1PKS |
| *Salinispora tropica* CNT261 | 2524614530 | 13 | CYP163B1 | 2524618075.c00001_H301DRA...region1 | NRPS |
|  |  |  | CYP107NH1 | 2524618075.c00001_H301DRA...region3 | NRPS |
|  |  |  | CYP247A8 | 2524618075.c00001_H301DRA...region3 | NRPS |
|  |  |  | CYP107Z27 | 2524618075.c00001_H301DRA...region3 | NRPS |
|  |  |  | CYP154M1 | 2524618077.c00003_H301DRA...region1 | NRPS |
|  |  |  | CYP208A4 | 2524618077.c00003_H301DRA...region1 | NRPS |
|  |  |  | CYP211C1 | 2524618077.c00003_H301DRA...region3 | T2PKS |
|  |  |  | CYP107AW1 | 2524618079.c00005_H301DRA...region1 | Bacteriocin |
|  |  |  | CYP107AY1 | 2524618092.c00018_H301DRA...region1 | NRPS |
|  |  |  | CYP1004B1 | 2524618096.c00022_H301DRA...region1 | NRPS |
|  |  |  | CYP1004A1 | 2524618096.c00022_H301DRA...region1 | NRPS |
|  |  |  | CYP125G1 | 2524618096.c00022_H301DRA...region1 | NRPS |
|  |  |  | CYP107E3 | 2524618115.c00041_H301DRA...region1 | T1PKS |
| *Salinispora tropica* CNR699 | 2518645624 | 11 | CYP107AY1 | 2518683438.c00001_C589DRA...region1 | terpene |
|  |  |  | CYP163B1 | 2518683439.c00002_C589DRA...region1 | NRPS |
|  |  |  | CYP154M1 | 2518683440.c00003_C589DRA...region2 | NRPS |
|  |  |  | CYP208A4 | 2518683440.c00003_C589DRA...region2 | NRPS |
|  |  |  | CYP211C1 | 2518683440.c00003_C589DRA...region4 | T2PKS |
|  |  |  | CYP107AW1 | 2518683441.c00004_C589DRA...region2 | Bacteriocin |
|  |  |  | CYP113E1 | 2518683455.c00018_C589DRA...region1 | T1PKS |
|  |  |  | CYP1004B1 | 2518683459.c00022_C589DRA...region1 | NRPS |
|  |  |  | CYP1004A1 | 2518683459.c00022_C589DRA...region1 | NRPS |
|  |  |  | CYP125G1 | 2518683459.c00022_C589DRA...region1 | NRPS |
|  |  |  | CYP107E3 | 2518683471.c00034_C589DRA...region1 | T1PKS |
| *Salinispora arenicola* CNQ748 | 2515154180 | 15 | CYP107AY2 | 2515168620.c00002_B106DRA...region1 | NRPS |
|  |  |  | CYP105CT1 | 2515168620.c00002_B106DRA...region1 | NRPS |
|  |  |  | CYP154M5 | 2515168620.c00002_B106DRA...region1 | NRPS |
|  |  |  | CYP105CP2 | 2515168622.c00004_B106DRA...region2 | lanthipeptide |
|  |  |  | CYP105CN1 | 2515168622.c00004_B106DRA...region2 | lanthipeptide |
|  |  |  | CYP107FS2 | 2515168622.c00004_B106DRA...region2 | lanthipeptide |
|  |  |  | CYP248A2 | 2515168622.c00004_B106DRA...region2 | lanthipeptide |
|  |  |  | CYP105W2 | 2515168622.c00004_B106DRA...region2 | lanthipeptide |
|  |  |  | CYP211C1 | 2515168625.c00007_B106DRA...region1 | T2PKS |
|  |  |  | CYP245A7 | 2515168627.c00009_B106DRA...region1 | Indole |
|  |  |  | CYP244A4 | 2515168627.c00009_B106DRA...region1 | Indole |
|  |  |  | CYP208A12 | 2515168631.c00013_B106DRA...region1 | T1PKS |
|  |  |  | CYP107Q4 | 2515168634.c00016_B106DRA...region1 | T1PKS |
|  |  |  | CYP105G5 | 2515168634.c00016_B106DRA...region1 | T1PKS |
|  |  |  | CYP1051A1 | 2515168634.c00016_B106DRA...region2 | Terpene |
| *Salinispora arenicola* CNS-991, DSM 45545 | 2516143022 | 12 | CYP154M21 | 2516144862.scaffold1.region2 | NRPS |
|  |  |  | CYP154M13 | 2516144862.scaffold1.region2 | NRPS |
|  |  |  | CYP105W2 | 2516144862.scaffold1.region2 | NRPS |
|  |  |  | CYP248A2 | 2516144862.scaffold1.region2 | NRPS |
|  |  |  | CYP154M20 | 2516144862.scaffold1.region2 | NRPS |
|  |  |  | CYP154M15 | 2516144862.scaffold1.region3 | ladderane |
|  |  |  | CYP125G6 | 2516144862.scaffold1.region3 | ladderane |
|  |  |  | CYP107FS2 | 2516144862.scaffold1.region3 | ladderane |
|  |  |  | CYP105CN1 | 2516144862.scaffold1.region3 | ladderane |
|  |  |  | CYP105CP2 | 2516144862.scaffold1.region3 | ladderane |
|  |  |  | CYP244A4 | 2516144862.scaffold1.region5 | Indole |
|  |  |  | CYP245A7 | 2516144862.scaffold1.region5 | Indole |
| *Salinispora pacifica* CNT854 | 2515154170 | 10 | CYP154AJ2 | 2515167898.c00002_B115DRA...region2 | LAP |
|  |  |  | CYP211C9 | 2515167898.c00002_B115DRA...region3 | T2PKS |
|  |  |  | CYP107AW6 | 2515167899.c00003_B115DRA...region1 | Bacteriocin |
|  |  |  | CYP245A11 | 2515167899.c00003_B115DRA...region2 | indole |
|  |  |  | CYP244A5 | 2515167899.c00003_B115DRA...region2 | indole |
|  |  |  | CYP208A27 | 2515167909.c00013_B115DRA...region1 | T1PKS |
|  |  |  | CYP107FH3 | 2515167910.c00014_B115DRA...region1 | NRPS-like |
|  |  |  | CYP2054A3 | 2515167910.c00014_B115DRA...region1 | NRPS-like |
|  |  |  | CYP161N4 | 2515167910.c00014_B115DRA...region1 | NRPS-like |
|  |  |  | CYP1196A2 | 2515167921.c00025_B115DRA...region1 | NRPS |
| *Salinispora pacifica* DSM 45543 | 2517572194 | 11 | CYP162A8 | 2517598672.c00001_SALPACD...region2 | NRPS-like |
|  |  |  | CYP244A5 | 2517598672.c00001_SALPACD...region7 | NRPS |
|  |  |  | CYP245A11 | 2517598672.c00001_SALPACD...region7 | NRPS |
|  |  |  | CYP107AW6 | 2517598672.c00001_SALPACD...region8 | Bacteriocin |
|  |  |  | CYP208A21 | 2517598672.c00001_SALPACD...region9 | NRPS |
|  |  |  | CYP154M16 | 2517598672.c00001_SALPACD...region9 | NRPS |
|  |  |  | CYP211C6 | 2517598672.c00001_SALPACD...region13 | T2PKS |
|  |  |  | CYP208A26 | 2517598672.c00001_SALPACD...region15 | T1PKS |
|  |  |  | CYP163B20 | 2517598672.c00001_SALPACD...region20 | transAT-PKS-like |
|  |  |  | CYP107CT3 | 2517598672.c00001_SALPACD...region21 | NRPS |
|  |  |  | CYP107AY7 | 2517598672.c00001_SALPACD...region24 | NRPS |
| *Salinispora arenicola* CNY260 | 2518285560 | 17 | CYP107Q4 | 2518290865.c00001_C579DRA...region1 | T1PKS |
|  |  |  | CYP105G5 | 2518290865.c00001_C579DRA...region1 | T1PKS |
|  |  |  | CYP1051A1 | 2518290865.c00001_C579DRA...region2 | Terpene |
|  |  |  | CYP211C1 | 2518290867.c00003_C579DRA...region5 | T2PKS |
|  |  |  | CYP208A12 | 2518290870.c00006_C579DRA...region2 | T1PKS |
|  |  |  | CYP105W2 | 2518290872.c00008_C579DRA...region1 | oligosaccharide |
|  |  |  | CYP248A2 | 2518290872.c00008_C579DRA...region1 | oligosaccharide |
|  |  |  | CYP107FS2 | 2518290872.c00008_C579DRA...region1 | oligosaccharide |
|  |  |  | CYP105CN1 | 2518290872.c00008_C579DRA...region1 | oligosaccharide |
|  |  |  | CYP105CP2 | 2518290872.c00008_C579DRA...region1 | oligosaccharide |
|  |  |  | CYP107AY2 | 2518290873.c00009_C579DRA...region1 | NRPS |
|  |  |  | CYP105CT1 | 2518290873.c00009_C579DRA...region1 | NRPS |
|  |  |  | CYP154M5 | 2518290873.c00009_C579DRA...region1 | NRPS |
|  |  |  | CYP166A4 | 2518290874.c00010_C579DRA...region1 | T1PKS |
|  |  |  | CYP245A7 | 2518290875.c00011_C579DRA...region2 | Indole |
|  |  |  | CYP244A4 | 2518290875.c00011_C579DRA...region2 | Indole |
|  |  |  | CYP105CH1 | 2518290883.c00019_C579DRA...region1 | T1PKS |
| *Salinispora arenicola* CNT799 | 2526164509 | 23 | CYP208A12 | 2526165619.c00001_K389DRA...region2 | T1PKS |
|  |  |  | CYP105CH1 | 2526165620.c00002_K389DRA...region2 | T1PKS |
|  |  |  | CYP107AX13 | 2526165621.c00003_K389DRA...region4 | ladderane |
|  |  |  | CYP166A4 | 2526165621.c00003_K389DRA...region5 | NRPS-like |
|  |  |  | CYP105CP2 | 2526165625.c00007_K389DRA...region2 | NRPS |
|  |  |  | CYP105CN1 | 2526165625.c00007_K389DRA...region2 | NRPS |
|  |  |  | CYP107FS2 | 2526165625.c00007_K389DRA...region2 | NRPS |
|  |  |  | CYP125G6 | 2526165625.c00007_K389DRA...region2 | NRPS |
|  |  |  | CYP154M15 | 2526165625.c00007_K389DRA...region2 | NRPS |
|  |  |  | CYP107Q4 | 2526165628.c00010_K389DRA...region1 | T1PKS |
|  |  |  | CYP105G5 | 2526165628.c00010_K389DRA...region1 | T1PKS |
|  |  |  | CYP1051A1 | 2526165628.c00010_K389DRA...region2 | Terpene |
|  |  |  | CYP244A4 | 2526165630.c00012_K389DRA...region1 | Indole |
|  |  |  | CYP245A7 | 2526165630.c00012_K389DRA...region1 | Indole |
|  |  |  | CYP162P1 | 2526165633.c00015_K389DRA...region1 | NRPS |
|  |  |  | CYP211C1 | 2526165636.c00018_K389DRA...region1 | T2PKS |
|  |  |  | CYP154M20 | 2526165638.c00020_K389DRA...region1 | oligosaccharide |
|  |  |  | CYP248A2 | 2526165638.c00020_K389DRA...region1 | oligosaccharide |
|  |  |  | CYP105W2 | 2526165638.c00020_K389DRA...region1 | oligosaccharide |
|  |  |  | CYP154M13 | 2526165638.c00020_K389DRA...region1 | oligosaccharide |
|  |  |  | CYP154M21 | 2526165638.c00020_K389DRA...region1 | oligosaccharide |
|  |  |  | CYP107AY2 | 2526165640.c00022_K389DRA...region1 | NRPS |
|  |  |  | CYP1198B1 | 2526165640.c00022_K389DRA...region1 | NRPS |
| *Salinispora arenicola* CNH646 | 25154181 | 6 | CYP211C1 | 2515168709.c00001_B163DRA...region3 | T2PKS |
|  |  |  | CYP162P1 | 2515168711.c00003_B163DRA...region1 | NRPS |
|  |  |  | CYP208A12 | 2515168712.c00004_B163DRA...region2 | T1PKS |
|  |  |  | CYP107Q4 | 2515168715.c00007_B163DRA...region1 | T1PKS |
|  |  |  | CYP105G5 | 2515168715.c00007_B163DRA...region1 | T1PKS |
|  |  |  | CYP1051A1 | 2515168715.c00007_B163DRA...region2 | Terpene |
| *Salinispora arenicola* CNY280 | 2517572154 | 14 | CYP245A7 | 2517596283.c00001_C580DRA...region1 | Indole |
|  |  |  | CYP244A4 | 2517596283.c00001_C580DRA...region1 | Indole |
|  |  |  | CYP105CP2 | 2517596283.c00001_C580DRA...region3 | NRPS |
|  |  |  | CYP105CN1 | 2517596283.c00001_C580DRA...region3 | NRPS |
|  |  |  | CYP107FS2 | 2517596283.c00001_C580DRA...region3 | NRPS |
|  |  |  | CYP125G6 | 2517596283.c00001_C580DRA...region3 | NRPS |
|  |  |  | CYP154M15 | 2517596283.c00001_C580DRA...region3 | NRPS |
|  |  |  | CYP154M20 | 2517596283.c00001_C580DRA...region4 | oligosaccharide |
|  |  |  | CYP248A2 | 2517596283.c00001_C580DRA...region4 | oligosaccharide |
|  |  |  | CYP105W2 | 2517596283.c00001_C580DRA...region4 | oligosaccharide |
|  |  |  | CYP154M13 | 2517596283.c00001_C580DRA...region4 | oligosaccharide |
|  |  |  | CYP154M21 | 2517596283.c00001_C580DRA...region4 | oligosaccharide |
|  |  |  | CYP211C1 | 2517596285.c00003_C580DRA...region3 | T2PKS |
|  |  |  | CYP208A12 | 2517596287.c00005_C580DRA...region1 | T1PKS |
| *Salinispora pacifica* CNY202 | 2528311034 | 4 | CYP154M19 | 2528320063.c00001_K228DRA...region1 | NRPS |
|  |  |  | CYP154AJ3 | 2528320085.c00023_K228DRA...region1 | NRPS |
|  |  |  | CYP211C3 | 2528320095.c00033_K228DRA...region1 | T2PKS |
|  |  |  | CYP107AY4 | 2528320103.c00041_K228DRA...region1 | NRPS |
| *Salinispora arenicola* CNX891 | 2515154187 | 14 | CYP245A7 | 2515169201.c00006_B108DRA...region2 | Indole |
|  |  |  | CYP244A4 | 2515169201.c00006_B108DRA...region2 | Indole |
|  |  |  | CYP105CP2 | 2515169204.c00009_B108DRA...region2 | NRPS |
|  |  |  | CYP105CN1 | 2515169204.c00009_B108DRA...region2 | NRPS |
|  |  |  | CYP107FS2 | 2515169204.c00009_B108DRA...region2 | NRPS |
|  |  |  | CYP248A2 | 2515169204.c00009_B108DRA...region2 | NRPS |
|  |  |  | CYP105W2 | 2515169204.c00009_B108DRA...region2 | NRPS |
|  |  |  | CYP107Q4 | 2515169207.c00012_B108DRA...region1 | T1PKS |
|  |  |  | CYP105G5 | 2515169207.c00012_B108DRA...region1 | T1PKS |
|  |  |  | CYP1051A1 | 2515169207.c00012_B108DRA...region2 | Terpene |
|  |  |  | CYP211C1 | 2515169209.c00014_B108DRA...region1 | T2PKS |
|  |  |  | CYP107AY2-fragment | 2515169210.c00015_B108DRA...region1 | NRPS |
|  |  |  | CYP105CT1 | 2515169210.c00015_B108DRA...region1 | NRPS |
|  |  |  | CYP154M5 | 2515169210.c00015_B108DRA...region1 | NRPS |
| *Salinispora pacifica* CNT855 | 2515154128 | 7 | CYP107AW7 | 2515165046.c00001_B113DRA...region2 | Bacteriocin |
|  |  |  | CYP107FH3 | 2515165058.c00013_B113DRA...region1 | NRPS-like |
|  |  |  | CYP2054A3 | 2515165058.c00013_B113DRA...region1 | NRPS-like |
|  |  |  | CYP161N4 | 2515165058.c00013_B113DRA...region1 | NRPS-like |
|  |  |  | CYP208A22 | 2515165059.c00014_B113DRA...region1 | T1PKS |
|  |  |  | CYP107AY9 | 2515165080.c00035_B113DRA...region1 | NRPS |
|  |  |  | CYP107E37 | 2515165095.c00050_B113DRA...region1 | T1PKS |
| *Salinispora pacifica* CNS996 | 2517572157 | 13 | CYP244A5 | 2517596538.c00006_C553DRA...region1 | NRPS |
|  |  |  | CYP245A11 | 2517596538.c00006_C553DRA...region1 | NRPS |
|  |  |  | CYP211C6 | 2517596539.c00007_C553DRA...region1 | T2PKS |
|  |  |  | CYP107CL2 | 2517596540.c00008_C553DRA...region1 | NRPS |
|  |  |  | CYP1056B2 | 2517596540.c00008_C553DRA...region1 | NRPS |
|  |  |  | CYP107AW6 | 2517596542.c00010_C553DRA...region1 | Bacteriocin |
|  |  |  | CYP208A21 | 2517596542.c00010_C553DRA...region2 | NRPS |
|  |  |  | CYP154M16 | 2517596542.c00010_C553DRA...region2 | NRPS |
|  |  |  | CYP163B20 | 2517596543.c00011_C553DRA...region1 | NRPS |
|  |  |  | CYP162A8 | 2517596549.c00017_C553DRA...region1 | NRPS-like |
|  |  |  | CYP107AY7 | 2517596554.c00022_C553DRA...region1 | NRPS |
|  |  |  | CYP2091A1 | 2517596560.c00028_C553DRA...region1 | oligosaccharide |
|  |  |  | CYP107CT3 | 2517596564.c00032_C553DRA...region1 | NRPS |
| *Salinispora arenicola* CNT850 | 2515154135 | 22 | CYP208A12 | 2515165460.c00004_B165DRA...region1 | T1PKS |
|  |  |  | CYP166A4 | 2515165462.c00006_B165DRA...region1 | T1PKS |
|  |  |  | CYP105CP2 | 2515165463.c00007_B165DRA...region2 | NRPS |
|  |  |  | CYP105CN1 | 2515165463.c00007_B165DRA...region2 | NRPS |
|  |  |  | CYP107FS2 | 2515165463.c00007_B165DRA...region2 | NRPS |
|  |  |  | CYP125G6 | 2515165463.c00007_B165DRA...region2 | NRPS |
|  |  |  | CYP154M15 | 2515165463.c00007_B165DRA...region2 | NRPS |
|  |  |  | CYP1051A1 | 2515165467.c00011_B165DRA...region1 | Terpene |
|  |  |  | CYP105G5 | 2515165467.c00011_B165DRA...region2 | T1PKS |
|  |  |  | CYP107Q4 | 2515165467.c00011_B165DRA...region2 | T1PKS |
|  |  |  | CYP244A4 | 2515165468.c00012_B165DRA...region1 | Indole |
|  |  |  | CYP245A7 | 2515165468.c00012_B165DRA...region1 | Indole |
|  |  |  | CYP211C1 | 2515165472.c00016_B165DRA...region1 | T2PKS |
|  |  |  | CYP154M21 | 2515165474.c00018_B165DRA...region1 | NRPS |
|  |  |  | CYP154M13 | 2515165474.c00018_B165DRA...region1 | NRPS |
|  |  |  | CYP105W2 | 2515165474.c00018_B165DRA...region1 | NRPS |
|  |  |  | CYP248A2 | 2515165474.c00018_B165DRA...region1 | NRPS |
|  |  |  | CYP154M20 | 2515165474.c00018_B165DRA...region1 | NRPS |
|  |  |  | CYP162P1 | 2515165477.c00021_B165DRA...region1 | NRPS |
|  |  |  | CYP107AY2 | 2515165478.c00022_B165DRA...region1 | NRPS |
|  |  |  | CYP105CH1 | 2515165485.c00029_B165DRA...region1 | T1PKS |
|  |  |  | CYP107EU1 | 2515165489.c00033_B165DRA...region1 | T1PKS |
| *Salinispora pacifica* CNT133A | 2561511035 | 6 | CYP107AW10 | 2561515153.c00003_T436DRA...region2 | Bacteriocin |
|  |  |  | CYP244A12 | 2561515162.c00012_T436DRA...region2 | NRPS |
|  |  |  | CYP107AY8 | 2561515162.c00012_T436DRA...region2 | NRPS |
|  |  |  | CYP208A30 | 2561515165.c00015_T436DRA...region2 | T1PKS |
|  |  |  | CYP163B20 | 2561515195.c00045_T436DRA...region1 | NRPS |
|  |  |  | CYP107E37 | 2561515204.c00054_T436DRA...region1 | T1PKS |
| *Salinispora arenicola* CNH941 | 2515154193 | 16 | CYP105CP2 | 2515169666.c00003_B109DRA...region2 | NRPS |
|  |  |  | CYP105CN1 | 2515169666.c00003_B109DRA...region2 | NRPS |
|  |  |  | CYP107FS2 | 2515169666.c00003_B109DRA...region2 | NRPS |
|  |  |  | CYP125G6 | 2515169666.c00003_B109DRA...region2 | NRPS |
|  |  |  | CYP154M15 | 2515169666.c00003_B109DRA...region2 | NRPS |
|  |  |  | CYP154M20 | 2515169666.c00003_B109DRA...region3 | oligosaccharide |
|  |  |  | CYP248A2 | 2515169666.c00003_B109DRA...region3 | oligosaccharide |
|  |  |  | CYP105W2 | 2515169666.c00003_B109DRA...region3 | oligosaccharide |
|  |  |  | CYP154M13 | 2515169666.c00003_B109DRA...region3 | oligosaccharide |
|  |  |  | CYP154M21 | 2515169666.c00003_B109DRA...region3 | oligosaccharide |
|  |  |  | CYP208A12 | 2515169667.c00004_B109DRA...region1 | T1PKS |
|  |  |  | CYP245A7 | 2515169670.c00007_B109DRA...region1 | Indole |
|  |  |  | CYP244A4 | 2515169670.c00007_B109DRA...region1 | Indole |
|  |  |  | CYP107Q4 | 2515169674.c00011_B109DRA...region1 | T1PKS |
|  |  |  | CYP105G5 | 2515169674.c00011_B109DRA...region1 | T1PKS |
|  |  |  | CYP1051A1 | 2515169674.c00011_B109DRA...region2 | Terpene |
| *Salinispora pacifica* CNY239 | 2524614561 | 7 | CYP161N4 | 2524661751.c00016_H304DRA...region1 | T3PKS |
|  |  |  | CYP2054A3 | 2524661751.c00016_H304DRA...region1 | T3PKS |
|  |  |  | CYP107FH3 | 2524661751.c00016_H304DRA...region1 | T3PKS |
|  |  |  | CYP208A22 | 2524661756.c00021_H304DRA...region1 | T1PKS |
|  |  |  | CYP244A10 | 2524661759.c00024_H304DRA...region1 | NRPS |
|  |  |  | CYP107AY9 | 2524661759.c00024_H304DRA...region1 | NRPS |
|  |  |  | CYP107E37 | 2524661790.c00055_H304DRA...region1 | T1PKS |
| *Salinispora arenicola* CNR425 | 2528311033 | 1 | CYP211C1 | 2528319994.c00002_I008DRA...region4 | T2PKS |
| *Salinispora pacifica* CNR942 | 2518285561 | 9 | CYP1004B4 | 2518290928.c00001_C549DRA...region1 | NRPS |
|  |  |  | CYP1004A3 | 2518290928.c00001_C549DRA...region1 | NRPS |
|  |  |  | CYP125G4 | 2518290928.c00001_C549DRA...region1 | NRPS |
|  |  |  | CYP208A24 | 2518290928.c00001_C549DRA...region2 | T1PKS |
|  |  |  | CYP211C5 | 2518290928.c00001_C549DRA...region4 | T2PKS |
|  |  |  | CYP163B18 | 2518290934.c00007_C549DRA...region1 | NRPS |
|  |  |  | CYP1207A12 | 2518290936.c00009_C549DRA...region2 | NRPS |
|  |  |  | CYP107AY3 | 2518290951.c00024_C549DRA...region1 | NRPS |
|  |  |  | CYP113X1 | 2518290937.c00010_C549DRA...region1 | transAT-PKS |
| *Salinispora pacifica* CNT029 : | 2515154177 | 8 | CYP154J2 | 2515168458.c00001_B170DRA...region2 | NRPS |
|  |  |  | CYP244A5 | 2515168458.c00001_B170DRA...region3 | NRPS |
|  |  |  | CYP245A11 | 2515168458.c00001_B170DRA...region3 | NRPS |
|  |  |  | CYP107AW6 | 2515168458.c00001_B170DRA...region4 | Bacteriocin |
|  |  |  | CYP208A21 | 2515168458.c00001_B170DRA...region5 | NRPS |
|  |  |  | CYP154M16 | 2515168458.c00001_B170DRA...region5 | NRPS |
|  |  |  | CYP211C6 | 2515168458.c00001_B170DRA...region9 | T2PKS |
|  |  |  | CYP107AY7 | 2515168473.c00016_B170DRA...region1 | terpene |
| *Salinispora arenicola* CNX814 | 2517572152 | 16 | CYP208A12 | 2517596139.c00004_C575DRA...region1 | T1PKS |
|  |  |  | CYP245A7 | 2517596141.c00006_C575DRA...region2 | Indole |
|  |  |  | CYP244A4 | 2517596141.c00006_C575DRA...region2 | Indole |
|  |  |  | CYP166A4 | 2517596142.c00007_C575DRA...region2 | T1PKS |
|  |  |  | CYP107AY2-fragment | 2517596144.c00009_C575DRA...region1 | NRPS |
|  |  |  | CYP105CT1 | 2517596144.c00009_C575DRA...region1 | NRPS |
|  |  |  | CYP154M5 | 2517596144.c00009_C575DRA...region1 | NRPS |
|  |  |  | CYP107FS2 | 2517596146.c00011_C575DRA...region1 | NRPS |
|  |  |  | CYP105CN1 | 2517596146.c00011_C575DRA...region1 | NRPS |
|  |  |  | CYP105CP2 | 2517596146.c00011_C575DRA...region1 | NRPS |
|  |  |  | CYP1051A1 | 2517596147.c00012_C575DRA...region1 | Terpene |
|  |  |  | CYP105G5 | 2517596147.c00012_C575DRA...region2 | T1PKS |
|  |  |  | CYP107Q4 | 2517596147.c00012_C575DRA...region2 | T1PKS |
|  |  |  | CYP211C1 | 2517596151.c00016_C575DRA...region1 | T2PKS |
|  |  |  | CYP105W2 | 2517596157.c00022_C575DRA...region1 | oligosaccharide |
|  |  |  | CYP248A2 | 2517596157.c00022_C575DRA...region1 | oligosaccharide |
| *Salinispora pacifica* CNR894 | 2515154194 | 9 | CYP107AW7 | 2515169737.c00001_B119DRA...region2 | Bacteriocin |
|  |  |  | CYP164C2 | 2515169739.c00003_B119DRA...region1 | NRPS |
|  |  |  | CYP208A22 | 2515169754.c00018_B119DRA...region1 | T1PKS |
|  |  |  | CYP161N4 | 2515169756.c00020_B119DRA...region1 | T3PKS |
|  |  |  | CYP2054A3 | 2515169756.c00020_B119DRA...region1 | T3PKS |
|  |  |  | CYP107FH3 | 2515169756.c00020_B119DRA...region1 | T3PKS |
|  |  |  | CYP107AY9 | 2515169760.c00024_B119DRA...region1 | terpene |
|  |  |  | CYP244A10 | 2515169760.c00024_B119DRA...region1 | terpene |
|  |  |  | CYP107E37 | 2515169793.c00057_B119DRA...region1 | T1PKS |
| *Salinispora pacifica* CNT150 | 2515154136 | 12 | CYP211C8 | 2515165525.c00002_B174DRA...region2 | T2PKS |
|  |  |  | CYP244A5 | 2515165532.c00009_B174DRA...region1 | Indole |
|  |  |  | CYP245A13 | 2515165532.c00009_B174DRA...region1 | Indole |
|  |  |  | CYP208A28 | 2515165533.c00010_B174DRA...region1 | T1PKS |
|  |  |  | CYP154M18 | 2515165533.c00010_B174DRA...region1 | T1PKS |
|  |  |  | CYP107Z27 | 2515165536.c00013_B174DRA...region1 | NRPS |
|  |  |  | CYP247A8 | 2515165536.c00013_B174DRA...region1 | NRPS |
|  |  |  | CYP107NH1 | 2515165536.c00013_B174DRA...region1 | NRPS |
|  |  |  | CYP105AH4 | 2515165538.c00015_B174DRA...region1 | T1PKS |
|  |  |  | CYP113E2 | 2515165542.c00019_B174DRA...region1 | T1PKS |
|  |  |  | CYP244A9 | 2515165550.c00027_B174DRA...region1 | NRPS |
|  |  |  | CYP107NG1 | 2515165562.c00039_B174DRA...region1 | T2PKS |
| *Salinispora tropica* CNS197 | 2515154163 | 11 | CYP163B1 | 2515167485.c00002_B123DRA...region2 | transAT-PKS-like, |
|  |  |  | CYP154M1 | 2515167486.c00003_B123DRA...region2 | NRPS |
|  |  |  | CYP208A4 | 2515167486.c00003_B123DRA...region2 | NRPS |
|  |  |  | CYP211C1 | 2515167486.c00003_B123DRA...region4 | T2PKS |
|  |  |  | CYP107AW1 | 2515167488.c00005_B123DRA...region2 | Bacteriocin |
|  |  |  | CYP107AY1 | 2515167489.c00006_B123DRA...region2 | NRPS |
|  |  |  | CYP113E1 | 2515167500.c00017_B123DRA...region2 | T1PKS |
|  |  |  | CYP1004B1 | 2515167505.c00022_B123DRA...region1 | NRPS |
|  |  |  | CYP1004A1 | 2515167505.c00022_B123DRA...region1 | NRPS |
|  |  |  | CYP125G1 | 2515167505.c00022_B123DRA...region1 | NRPS |
|  |  |  | CYP107E3 | 2515167515.c00032_B123DRA...region1 | T1PKS |
| *Salinispora arenicola* CNS848 | 2571042345 | 16 | CYP162P1 | 2571067352.c00001_T422DRA...region1 | NRPS |
|  |  |  | CYP211C1 | 2571067353.c00002_T422DRA...region1 | T2PKS |
|  |  |  | CYP166A4 | 2571067354.c00003_T422DRA...region1 | T1PKS |
|  |  |  | CYP208A12 | 2571067355.c00004_T422DRA...region1 | T1PKS |
|  |  |  | CYP107AY2 | 2571067357.c00006_T422DRA...region1 | NRPS |
|  |  |  | CYP245A7 | 2571067358.c00007_T422DRA...region1 | Indole |
|  |  |  | CYP244A4 | 2571067358.c00007_T422DRA...region1 | Indole |
|  |  |  | CYP105CP2 | 2571067360.c00009_T422DRA...region2 | NRPS |
|  |  |  | CYP105CN1 | 2571067360.c00009_T422DRA...region2 | NRPS |
|  |  |  | CYP107FS2 | 2571067360.c00009_T422DRA...region2 | NRPS |
|  |  |  | CYP125G6 | 2571067360.c00009_T422DRA...region2 | NRPS, |
|  |  |  | CYP154M15 | 2571067360.c00009_T422DRA...region2 | NRPS |
|  |  |  | CYP154M21 | 2571067367.c00016_T422DRA...region1 | NRPS |
|  |  |  | CYP154M13 | 2571067367.c00016_T422DRA...region1 | NRPS |
|  |  |  | CYP105W2 | 2571067367.c00016_T422DRA...region1 | NRPS |
|  |  |  | CYP248A2 | 2571067367.c00016_T422DRA...region1 | NRPS |
| *Salinispora arenicola* CNH905 | 2515154183 | 22 | CYP1051A1 | 2515168866.c00001_B102DRA...region1 | Terpene |
|  |  |  | CYP105G5 | 2515168866.c00001_B102DRA...region2 | T1PKS |
|  |  |  | CYP107Q4 | 2515168866.c00001_B102DRA...region2 | T1PKS |
|  |  |  | CYP208A12 | 2515168867.c00002_B102DRA...region2 | T1PKS |
|  |  |  | CYP211C1 | 2515168868.c00003_B102DRA...region1 | T2PKS |
|  |  |  | CYP162P1 | 2515168871.c00006_B102DRA...region1 | NRPS |
|  |  |  | CYP244A4 | 2515168873.c00008_B102DRA...region1 | Indole |
|  |  |  | CYP245A7 | 2515168873.c00008_B102DRA...region1 | Indole |
|  |  |  | CYP107EU1 | 2515168877.c00012_B102DRA...region1 | NRPS-like |
|  |  |  | CYP166A4 | 2515168880.c00015_B102DRA...region1 | T1PKS |
|  |  |  | CYP154M20 | 2515168881.c00016_B102DRA...region1 | oligosaccharide |
|  |  |  | CYP248A2 | 2515168881.c00016_B102DRA...region1 | oligosaccharide |
|  |  |  | CYP105W2 | 2515168881.c00016_B102DRA...region1 | oligosaccharide |
|  |  |  | CYP154M13 | 2515168881.c00016_B102DRA...region1 | oligosaccharide |
|  |  |  | CYP154M21 | 2515168881.c00016_B102DRA...region1 | oligosaccharide |
|  |  |  | CYP105CP2 | 2515168882.c00017_B102DRA...region2 | NRPS |
|  |  |  | CYP105CN1 | 2515168882.c00017_B102DRA...region2 | NRPS |
|  |  |  | CYP107FS2 | 2515168882.c00017_B102DRA...region2 | NRPS |
|  |  |  | CYP125G6 | 2515168886.c00021_B102DRA...region1 | NRPS |
|  |  |  | CYP154M15 | 2515168886.c00021_B102DRA...region1 | NRPS |
|  |  |  | CYP107AY13 | 2515168892.c00027_B102DRA...region1 | T1PKS |
|  |  |  | CYP1198B1 | 2515168902.c00037_B102DRA...region1 | NRPS |
| *Salinispora arenicola* CNY256 | 2518285559 | 15 | CYP208A12 | 2518290787.c00002_C578DRA...region1 | T1PKS |
|  |  |  | CYP107AY2 | 2518290789.c00004_C578DRA...region1 | NRPS |
|  |  |  | CYP105CT1 | 2518290789.c00004_C578DRA...region1 | NRPS |
|  |  |  | CYP154M5 | 2518290789.c00004_C578DRA...region1 | NRPS |
|  |  |  | CYP211C1 | 2518290790.c00005_C578DRA...region1 | T2PKS |
|  |  |  | CYP1051A1 | 2518290791.c00006_C578DRA...region1 | Terpene |
|  |  |  | CYP105G5 | 2518290791.c00006_C578DRA...region2 | T1PKS |
|  |  |  | CYP107Q4 | 2518290791.c00006_C578DRA...region2 | T1PKS |
|  |  |  | CYP245A7 | 2518290795.c00010_C578DRA...region1 | Indole |
|  |  |  | CYP244A4 | 2518290795.c00010_C578DRA...region1 | Indole |
|  |  |  | CYP105W2 | 2518290796.c00011_C578DRA...region1 | oligosaccharide |
|  |  |  | CYP248A2 | 2518290796.c00011_C578DRA...region1 | oligosaccharide |
|  |  |  | CYP107FS2 | 2518290796.c00011_C578DRA...region1 | oligosaccharide |
|  |  |  | CYP105CN1 | 2518290796.c00011_C578DRA...region1 | oligosaccharide |
|  |  |  | CYP105CP2 | 2518290796.c00011_C578DRA...region1 | oligosaccharide |
| *Salinispora arenicola* CNS820 | 2565956528 | 14 | CYP211C1 | 2565959905.c00001_T365DRA...region5 | T2PKS |
|  |  |  | CYP208A12 | 2565959907.c00003_T365DRA...region2 | T1PKS |
|  |  |  | CYP105CT1 | 2565959908.c00004_T365DRA...region1 | terpene |
|  |  |  | CYP154M5 | 2565959908.c00004_T365DRA...region1 | terpene |
|  |  |  | CYP244A4 | 2565959911.c00007_T365DRA...region1 | Indole |
|  |  |  | CYP245A7 | 2565959911.c00007_T365DRA...region1 | Indole |
|  |  |  | CYP166A4 | 2565959913.c00009_T365DRA...region1 | T1PKS |
|  |  |  | CYP107FS2 | 2565959916.c00012_T365DRA...region1 | NRPS |
|  |  |  | CYP105CN1 | 2565959916.c00012_T365DRA...region1 | NRPS |
|  |  |  | CYP105CP2 | 2565959916.c00012_T365DRA...region1 | NRPS |
|  |  |  | CYP107Q4 | 2565959917.c00013_T365DRA...region1 | T1PKS |
|  |  |  | CYP105G5 | 2565959917.c00013_T365DRA...region1 | T1PKS |
|  |  |  | CYP1051A1 | 2565959917.c00013_T365DRA...region2 | Terpene |
|  |  |  | CYP107AY2 | 2565959922.c00018_T365DRA...region1 | NRPS |
| *Salinispora arenicola* CNS243 | 2524614515 | 16 | CYP211C1 | 2524617272.c00002_H302DRA...region5 | T2PKS |
|  |  |  | CYP166A4 | 2524617274.c00004_H302DRA...region2 | NRPS-like |
|  |  |  | CYP244A4 | 2524617277.c00007_H302DRA...region1 | Indole |
|  |  |  | CYP245A7 | 2524617277.c00007_H302DRA...region1 | Indole |
|  |  |  | CYP208A12 | 2524617278.c00008_H302DRA...region1 | T1PKS |
|  |  |  | CYP107AY2-fragment | 2524617279.c00009_H302DRA...region1 | NRPS |
|  |  |  | CYP105CT1 | 2524617279.c00009_H302DRA...region2 | terpene |
|  |  |  | CYP154M5 | 2524617279.c00009_H302DRA...region2 | terpene |
|  |  |  | CYP1051A1 | 2524617285.c00015_H302DRA...region1 | Terpene |
|  |  |  | CYP105G5 | 2524617285.c00015_H302DRA...region2 | T1PKS |
|  |  |  | CYP107Q4 | 2524617285.c00015_H302DRA...region2 | T1PKS |
|  |  |  | CYP248A2 | 2524617286.c00016_H302DRA...region1 | NRPS |
|  |  |  | CYP105W2 | 2524617286.c00016_H302DRA...region1 | NRPS |
|  |  |  | CYP105CP2 | 2524617288.c00018_H302DRA...region2 | NRPS |
|  |  |  | CYP105CN1 | 2524617288.c00018_H302DRA...region2 | NRPS |
|  |  |  | CYP107FS2 | 2524617288.c00018_H302DRA...region2 | NRPS |
| *Salinispora tropica* CNB536 | 251752212 | 13 | CYP163B1 | 2517600344.c00001_B099DRA...region1 | NRPS |
|  |  |  | CYP107NH1 | 2517600344.c00001_B099DRA...region3 | NRPS |
|  |  |  | CYP247A8 | 2517600344.c00001_B099DRA...region3 | NRPS |
|  |  |  | CYP107Z27 | 2517600344.c00001_B099DRA...region3 | NRPS |
|  |  |  | CYP211C1 | 2517600350.c00007_B099DRA...region1 | T2PKS |
|  |  |  | CYP125G1 | 2517600362.c00019_B099DRA...region1 | NRPS |
|  |  |  | CYP1004A1 | 2517600362.c00019_B099DRA...region1 | NRPS |
|  |  |  | CYP1004B1 | 2517600362.c00019_B099DRA...region1 | NRPS |
|  |  |  | CYP107AY1 | 2517600365.c00022_B099DRA...region1 | NRPS |
|  |  |  | CYP208A22 | 2517600379.c00036_B099DRA...region1 | T1PKS |
|  |  |  | CYP107AW1 | 2517600390.c00047_B099DRA...region1 | Bacteriocin |
|  |  |  | CYP107E3 | 2517600398.c00055_B099DRA...region1 | T1PKS |
|  |  |  | CYP283A2 | 2517600408.c00065_B099DRA...region1 | bacteriocin |
| *Salinispora pacifica* CNT084 | 2515154202 | 6 | CYP107AW10 | 2515170504.c00006_B171DRA...region2 | Bacteriocin |
|  |  |  | CYP208A30 | 2515170512.c00014_B171DRA...region1 | T1PKS |
|  |  |  | CYP244A12 | 2515170513.c00015_B171DRA...region1 | NRPS |
|  |  |  | CYP107AY8 | 2515170513.c00015_B171DRA...region1 | NRPS |
|  |  |  | CYP163B20 | 2515170545.c00047_B171DRA...region1 | NRPS |
|  |  |  | CYP107E37 | 2515170551.c00053_B171DRA...region1 | T1PKS |
| *Salinispora arenicola* CNT857 | 2515154127 | 21 | CYP208A12 | 2515164954.c00001_B107DRA...region2 | T1PKS |
|  |  |  | CYP105CP2 | 2515164957.c00004_B107DRA...region2 | NRPS |
|  |  |  | CYP105CN1 | 2515164957.c00004_B107DRA...region2 | NRPS |
|  |  |  | CYP107FS2 | 2515164957.c00004_B107DRA...region2 | NRPS |
|  |  |  | CYP125G6 | 2515164957.c00004_B107DRA...region2 | NRPS |
|  |  |  | CYP154M15 | 2515164957.c00004_B107DRA...region2 | NRPS |
|  |  |  | CYP154M20 | 2515164957.c00004_B107DRA...region3 | oligosaccharide |
|  |  |  | CYP248A2 | 2515164957.c00004_B107DRA...region3 | oligosaccharide |
|  |  |  | CYP105W2 | 2515164957.c00004_B107DRA...region3 | oligosaccharide |
|  |  |  | CYP154M13 | 2515164957.c00004_B107DRA...region3 | oligosaccharide |
|  |  |  | CYP154M21 | 2515164957.c00004_B107DRA...region3 | oligosaccharide |
|  |  |  | CYP211C1 | 2515164959.c00006_B107DRA...region2 | T2PKS |
|  |  |  | CYP105CH1 | 2515164962.c00009_B107DRA...region2 | T1PKS |
|  |  |  | CYP107Q4 | 2515164963.c00010_B107DRA...region1 | T1PKS |
|  |  |  | CYP105G5 | 2515164963.c00010_B107DRA...region1 | T1PKS |
|  |  |  | CYP1051A1 | 2515164963.c00010_B107DRA...region2 | Terpene |
|  |  |  | CYP245A7 | 2515164965.c00012_B107DRA...region1 | Indole |
|  |  |  | CYP244A4 | 2515164965.c00012_B107DRA...region1 | Indole |
|  |  |  | CYP166A4 | 2515164966.c00013_B107DRA...region1 | T1PKS |
|  |  |  | CYP107AX13 | 2515164968.c00015_B107DRA...region1 | ladderane |
|  |  |  | CYP162N1 | 2515164976.c00023_B107DRA...region1 | NRPS |
| *Salinispora pacifica* DSM 45546 | 2516653042 | 7 | CYP107AY7 | 2516665583.c00001_Salpac5...region6 | NRPS |
|  |  |  | CYP244A5 | 2516665583.c00001_Salpac5...region14 | NRPS |
|  |  |  | CYP245A11 | 2516665583.c00001_Salpac5...region14 | NRPS |
|  |  |  | CYP107AW6 | 2516665583.c00001_Salpac5...region15 | Bacteriocin |
|  |  |  | CYP154M16 | 2516665583.c00001_Salpac5...region16 | NRPS |
|  |  |  | CYP211C6 | 2516665583.c00001_Salpac5...region19 | T2PKS |
|  |  |  | CYP1278A4 | 2516665583.c00001_Salpac5...region22 | NRPS |
| *Salinispora tropica* CNY012 | 2540341192 | 12 | CYP163B1 | 2540348493.c00003_K262DRA...region1 | NRPS |
|  |  |  | CYP107NH1 | 2540348496.c00006_K262DRA...region1 | T2PKS |
|  |  |  | CYP247A8 | 2540348493.c00003_K262DRA...region3 | NRPS |
|  |  |  | CYP107Z27 | 2540348493.c00003_K262DRA...region3 | NRPS |
|  |  |  | CYP211C1 | 2540348494.c00004_K262DRA...region1 | T2PKS |
|  |  |  | CYP208A22 | 2540348494.c00004_K262DRA...region4 | T1PKS |
|  |  |  | CYP107AW1 | 2540348496.c00006_K262DRA...region2 | Bacteriocin |
|  |  |  | CYP107AY1 | 2540348497.c00007_K262DRA...region2 | NRPS |
|  |  |  | CYP1004B1 | 2540348506.c00016_K262DRA...region1 | NRPS |
|  |  |  | CYP1004A1 | 2540348506.c00016_K262DRA...region1 | NRPS |
|  |  |  | CYP125G1 | 2540348506.c00016_K262DRA...region1 | NRPS |
|  |  |  | CYP107E3 | 2540348528.c00038_K262DRA...region1 | T1PKS |
| *Salinispora arenicola* CNY234 | 2519103195 | 12 | CYP1051A1 | 2519124107.c00002_F584DRA...region1 | Terpene |
|  |  |  | CYP211C1 | 2519124108.c00003_F584DRA...region4 | T2PKS |
|  |  |  | CYP166A4 | 2519124110.c00005_F584DRA...region2 | T1PKS |
|  |  |  | CYP105W2 | 2519124112.c00007_F584DRA...region1 | oligosaccharide |
|  |  |  | CYP248A2 | 2519124112.c00007_F584DRA...region1 | oligosaccharide |
|  |  |  | CYP107FS2 | 2519124112.c00007_F584DRA...region1 | oligosaccharide |
|  |  |  | CYP105CN1 | 2519124112.c00007_F584DRA...region1 | oligosaccharide |
|  |  |  | CYP105CP2 | 2519124112.c00007_F584DRA...region1 | oligosaccharide |
|  |  |  | CYP245A7 | 2519124114.c00009_F584DRA...region1 | Indole |
|  |  |  | CYP244A4 | 2519124114.c00009_F584DRA...region1 | Indole |
|  |  |  | CYP107AY2-fragment | 2519124115.c00010_F584DRA...region1 | NRPS |
|  |  |  | CYP208A12 | 2519124117.c00012_F584DRA...region1 | T1PKS |
| *Salinispora tropica* CNT250 | 2540341193 | 11 | CYP163B1 | 2540348645.c00001_K261DRA...region1 | NRPS |
|  |  |  | CYP154M1 | 2540348646.c00002_K261DRA...region2 | NRPS |
|  |  |  | CYP208A4 | 2540348646.c00002_K261DRA...region2 | NRPS |
|  |  |  | CYP211C1 | 2540348646.c00002_K261DRA...region4 | T2PKS |
|  |  |  | CYP107AY1 | 2540348648.c00004_K261DRA...region2 | NRPS |
|  |  |  | CYP107AW1 | 2540348649.c00005_K261DRA...region1 | Bacteriocin |
|  |  |  | CYP1004B1 | 2540348659.c00015_K261DRA...region1 | NRPS |
|  |  |  | CYP1004A1 | 2540348659.c00015_K261DRA...region1 | NRPS |
|  |  |  | CYP125G1 | 2540348659.c00015_K261DRA...region1 | NRPS |
|  |  |  | CYP113E1 | 2540348662.c00018_K261DRA...region2 | T1PKS |
|  |  |  | CYP107E3 | 2540348677.c00033_K261DRA...region1 | T1PKS |
| *Salinispora pacifica* CNR510 | 2571042008 | 7 | CYP107AW7 | 2571043004.c00005_T377DRA...region2 | Bacteriocin |
|  |  |  | CYP107AY9 | 2571043021.c00022_T377DRA...region1 | terpene |
|  |  |  | CYP161N4 | 2571043023.c00024_T377DRA...region1 | T3PKS |
|  |  |  | CYP2054A3 | 2571043023.c00024_T377DRA...region1 | T3PKS |
|  |  |  | CYP107FH3 | 2571043023.c00024_T377DRA...region1 | T3PKS |
|  |  |  | CYP208A22 | 2571043030.c00031_T377DRA...region1 | T1PKS |
|  |  |  | CYP107E37 | 2571043063.c00064_T377DRA...region1 | T1PKS |
| *Salinispora arenicola* CNT800 | 2515154088 | 21 | CYP208A12 | 2515161955.c00001_B040DRA...region2 | T1PKS |
|  |  |  | CYP107AY2 | 2515161960.c00006_B040DRA...region1 | NRPS |
|  |  |  | CYP245A7 | 2515161961.c00007_B040DRA...region1 | Indole |
|  |  |  | CYP244A4 | 2515161961.c00007_B040DRA...region1 | Indole |
|  |  |  | CYP166A4 | 2515161962.c00008_B040DRA...region1 | T1PKS |
|  |  |  | CYP107AX13 | 2515161962.c00008_B040DRA...region2 | ladderane |
|  |  |  | CYP154M15 | 2515161964.c00010_B040DRA...region1 | ladderane |
|  |  |  | CYP125G6 | 2515161964.c00010_B040DRA...region1 | ladderane |
|  |  |  | CYP107FS2 | 2515161964.c00010_B040DRA...region1 | ladderane |
|  |  |  | CYP105CN1 | 2515161964.c00010_B040DRA...region1 | ladderane |
|  |  |  | CYP105CP2 | 2515161964.c00010_B040DRA...region1 | ladderane |
|  |  |  | CYP105CH1 | 2515161967.c00013_B040DRA...region2 | T1PKS |
|  |  |  | CYP1051A1 | 2515161968.c00014_B040DRA...region1 | Terpene |
|  |  |  | CYP105G5 | 2515161968.c00014_B040DRA...region2 | T1PKS |
|  |  |  | CYP107Q4 | 2515161968.c00014_B040DRA...region2 | T1PKS |
|  |  |  | CYP211C1 | 2515161972.c00018_B040DRA...region | T2PKS |
|  |  |  | CYP154M21 | 2515161974.c00020_B040DRA...region1 | NRPS |
|  |  |  | CYP154M13 | 2515161974.c00020_B040DRA...region1 | NRPS |
|  |  |  | CYP105W2 | 2515161974.c00020_B040DRA...region1 | NRPS |
|  |  |  | CYP248A2 | 2515161974.c00020_B040DRA...region1 | NRPS |
|  |  |  | CYP154M20 | 2515161974.c00020_B040DRA...region1 | NRPS |
| *Salinispora pacifica* CNS055 | 2518285562 | 10 | CYP107AW8 | 2518290980.c00001_C550DRA...region6 | Bacteriocin |
|  |  |  | CYP162H1 | 2518290980.c00001_C550DRA...region8 | NRPS |
|  |  |  | CYP1611B1 | 2518290987.c00008_C550DRA...region1 | T1PKS |
|  |  |  | CYP2098A1 | 2518290987.c00008_C550DRA...region1 | T1PKS |
|  |  |  | CYP1004B3 | 2518290997.c00018_C550DRA...region1 | NRPS |
|  |  |  | CYP1004A4 | 2518290997.c00018_C550DRA...region1 | NRPS |
|  |  |  | CYP125G4 | 2518290997.c00018_C550DRA...region1 | NRPS |
|  |  |  | CYP107AY12 | 2518290998.c00019_C550DRA...region1 | NRPS |
|  |  |  | CYP162J1 | 2518291009.c00030_C550DRA...region1 | NRPS |
|  |  |  | CYP107E38 | 2518291018.c00039_C550DRA...region1 | T1PKS |
| *Salinispora arenicola* CNS051 | 2518285553 | 10 | CYP211C1 | 2518290242.c00003_C570DRA...region1 | T2PKS |
|  |  |  | CYP245A7 | 2518290244.c00005_C570DRA...region1 | Indole |
|  |  |  | CYP244A4 | 2518290244.c00005_C570DRA...region1 | Indole |
|  |  |  | CYP166A4 | 2518290245.c00006_C570DRA...region2 | T1PKS |
|  |  |  | CYP105W2 | 2518290246.c00007_C570DRA...region1 | oligosaccharide |
|  |  |  | CYP248A2 | 2518290246.c00007_C570DRA...region1 | oligosaccharide |
|  |  |  | CYP107FS2 | 2518290246.c00007_C570DRA...region1 | oligosaccharide |
|  |  |  | CYP105CN1 | 2518290246.c00007_C570DRA...region1 | oligosaccharide |
|  |  |  | CYP105CP2 | 2518290246.c00007_C570DRA...region1 | oligosaccharide |
|  |  |  | CYP208A12 | 2518290248.c00009_C570DRA...region1 | T1PKS |
| *Salinispora arenicola* CNB527 | 2515154093 | 21 | CYP208A12 | 2515162172.c00001_B033DRA...region1 | T1PKS |
|  |  |  | CYP2296A2 | 2515162173.c00002_B033DRA...region1 | T2PKS |
|  |  |  | CYP166A4 | 2515162173.c00002_B033DRA...region1 | T2PKS |
|  |  |  | CYP173K1 | 2515162173.c00002_B033DRA...region1 | T2PKS |
|  |  |  | CYP245A7 | 2515162174.c00003_B033DRA...region1 | Indole |
|  |  |  | CYP244A4 | 2515162174.c00003_B033DRA...region1 | Indole |
|  |  |  | CYP166A4 | 2515162177.c00006_B033DRA...region1 | T1PKS |
|  |  |  | CYP107AX-fragment7 | 2515162177.c00006_B033DRA...region2 | ladderane |
|  |  |  | CYP154M5 | 2515162182.c00011_B033DRA...region1 | terpene |
|  |  |  | CYP211C1 | 2515162183.c00012_B033DRA...region1 | T2PKS |
|  |  |  | CYP105CP2 | 2515162187.c00016_B033DRA...region2 | NRPS |
|  |  |  | CYP105CN1 | 2515162187.c00016_B033DRA...region2 | NRPS |
|  |  |  | CYP107FS2 | 2515162187.c00016_B033DRA...region2 | NRPS |
|  |  |  | CYP244A-fragment2 | 2515162191.c00020_B033DRA...region1 | NRPS |
|  |  |  | CYP107AY14 | 2515162191.c00020_B033DRA...region1 | NRPS |
|  |  |  | CYP107EU2 | 2515162195.c00024_B033DRA...region1 | T1PKS |
|  |  |  | CYP248A2 | 2515162196.c00025_B033DRA...region1 | oligosaccharide |
|  |  |  | CYP105W3 | 2515162196.c00025_B033DRA...region1 | oligosaccharide |
|  |  |  | CYP107Q4 | 2515162200.c00029_B033DRA...region1 | T1PKS |
|  |  |  | CYP105G7 | 2515162200.c00029_B033DRA...region1 | T1PKS |
|  |  |  | CYP1051A4 | 2515162200.c00029_B033DRA...region2 | Terpene |
| *Salinispora arenicola* CNS-205 | 641228504 | 22 | CYP208A12 | 641228495.NC_009953.region2 | T1PKS |
|  |  |  | CYP166A4 | 641228495.NC_009953.region6 | T1PKS |
|  |  |  | CYP107Q4 | 641228495.NC_009953.region6 | T1PKS |
|  |  |  | CYP105G5 | 641228495.NC_009953.region6 | T1PKS |
|  |  |  | CYP1051A1 | 641228495.NC_009953.region7 | Terpene |
|  |  |  | CYP105W2 | 641228495.NC_009953.region9 | oligosaccharide |
|  |  |  | CYP248A2 | 641228495.NC_009953.region9 | oligosaccharide |
|  |  |  | CYP107FS2 | 641228495.NC_009953.region10 | NRPS |
|  |  |  | CYP105CN1 | 641228495.NC_009953.region10 | NRPS |
|  |  |  | CYP105CP2 | 641228495.NC_009953.region10 | NRPS |
|  |  |  | CYP244A4 | 641228495.NC_009953.region12 | Indole |
|  |  |  | CYP245A7 | 641228495.NC_009953.region12 | Indole |
|  |  |  | CYP211C1 | 641228495.NC_009953.region17 | T2PKS |
|  |  |  | CYP107EU1 | 641228495.NC_009953.region22 | NRPS-like |
|  |  |  | CYP1198B1 | 641228495.NC_009953.region22 | NRPS-like |
|  |  |  | CYP105CH1 | 641228495.NC_009953.region22 | NRPS-like |
|  |  |  | CYP162A6 | 641228495.NC_009953.region24 | betalactone |
|  |  |  | CYP107HF1 | 641228495.NC_009953.region24 | betalactone |
|  |  |  | CYP113S1 | 641228495.NC_009953.region24 | betalactone |
|  |  |  | CYP107AY2 | 641228495.NC_009953.region26 | NRPS |
|  |  |  | CYP105CT1 | 641228495.NC_009953.region27 | terpene |
|  |  |  | CYP154M5 | 641228495.NC_009953.region27 | terpene |
| *Salinispora pacifica* CNT131 | 2515154200 | 10 | CYP107AW7 | 2515170263.c00002_B172DRA...region2 | Bacteriocin |
|  |  |  | CYP164C2 | 2515170265.c00004_B172DRA...region1 | NRPS |
|  |  |  | CYP208A22 | 2515170281.c00020_B172DRA...region1 | T1PKS |
|  |  |  | CYP107FH3 | 2515170283.c00022_B172DRA...region1 | NRPS-like |
|  |  |  | CYP2054A3 | 2515170283.c00022_B172DRA...region1 | NRPS-like |
|  |  |  | CYP161N4 | 2515170283.c00022_B172DRA...region1 | NRPS-like |
|  |  |  | CYP107AY9 | 2515170296.c00035_B172DRA...region1 | NRPS |
|  |  |  | CYP244A10 | 2515170296.c00035_B172DRA...region1 | NRPS |
|  |  |  | CYP107E37 | 2515170317.c00056_B172DRA...region1 | T1PKS |
|  |  |  | CYP107FH4 | 2515170325.c00064_B172DRA...region1 | T1PKS |
| *Salinispora pacifica* CNT609 | 2517572161 | 7 | CYP244A12 | 2517596856.c00005_C584DRA...region2 | NRPS |
|  |  |  | CYP107AY8 | 2517596856.c00005_C584DRA...region2 | NRPS |
|  |  |  | CYP163B20 | 2517596858.c00007_C584DRA...region1 | transAT-PKS-like |
|  |  |  | CYP208A30 | 2517596869.c00018_C584DRA...region1 | T1PKS |
|  |  |  | CYP107AW10 | 2517596870.c00019_C584DRA...region2 | Bacteriocin |
|  |  |  | CYP1207A12 | 2517596888.c00037_C584DRA...region1 | NRPS |
|  |  |  | CYP107E37 | 2517596898.c00047_C584DRA...region1 | T1PKS |
| *Salinispora tropica* CNB-440 | 640427140 | 11 | CYP113E1 | 640427133.NC_009380.region1 | T1PKS |
|  |  |  | CYP163B1 | 640427133.NC_009380.region4 | transAT-PKS-like |
|  |  |  | CYP107AW1 | 640427133.NC_009380.region7 | Bacteriocin |
|  |  |  | CYP211C1 | 640427133.NC_009380.region10 | T2PKS |
|  |  |  | CYP208A4 | 640427133.NC_009380.region12 | NRPS |
|  |  |  | CYP154M1 | 640427133.NC_009380.region12 | NRPS |
|  |  |  | CYP107E3 | 640427133.NC_009380.region13 | T1PKS |
|  |  |  | CYP125G1 | 640427133.NC_009380.region13 | T1PKS |
|  |  |  | CYP1004A1 | 640427133.NC_009380.region13 | T1PKS |
|  |  |  | CYP1004B1 | 640427133.NC_009380.region13 | T1PKS |
|  |  |  | CYP107AY1 | 640427133.NC_009380.region17 | NRPS |
| *Salinispora arenicola* CNY690 | 2561511111 | 21 | CYP211C1 | 2561539429.c00003_T393DRA...region2 | T2PKS |
|  |  |  | CYP244A4 | 2561539430.c00004_T393DRA...region1 | Indole |
|  |  |  | CYP245A7 | 2561539430.c00004_T393DRA...region1 | Indole |
|  |  |  | CYP107AX-fragment8 | 2561539434.c00008_T393DRA...region1 | ladderane |
|  |  |  | CYP105W3 | 2561539435.c00009_T393DRA...region1 | oligosaccharide |
|  |  |  | CYP248A2 | 2561539435.c00009_T393DRA...region1 | oligosaccharide |
|  |  |  | CYP107FS2 | 2561539435.c00009_T393DRA...region1 | oligosaccharide |
|  |  |  | CYP105CN1 | 2561539435.c00009_T393DRA...region1 | oligosaccharide |
|  |  |  | CYP105CP2 | 2561539435.c00009_T393DRA...region1 | oligosaccharide |
|  |  |  | CYP105CT2 | 2561539436.c00010_T393DRA...region1 | terpene |
|  |  |  | CYP154M5 | 2561539436.c00010_T393DRA...region1 | terpene |
|  |  |  | CYP208A12 | 2561539442.c00016_T393DRA...region1 | T1PKS |
|  |  |  | CYP107EU2 | 2561539448.c00022_T393DRA...region1 | T1PKS |
|  |  |  | CYP107AY14 | 2561539454.c00028_T393DRA...region1 | NRPS |
|  |  |  | CYP244A-fragment2 | 2561539454.c00028_T393DRA...region1 | NRPS |
|  |  |  | CYP105CH2 | 2561539469.c00043_T393DRA...region1 | T1PKS |
|  |  |  | CYP105G7 | 2561539476.c00050_T393DRA...region1 | T1PKS |
|  |  |  | CYP107Q4 | 2561539476.c00050_T393DRA...region1 | T1PKS |
|  |  |  | CYP166A4 | 2561539482.c00056_T393DRA...region1 | T1PKS |
|  |  |  | CYP1051A4 | 2561539495.c00069_T393DRA...region1 | Terpene |
|  |  |  | CYP1198B2 | 2561539533.c00107_T393DRA...region1 | T1PKS |
| *Salinispora pacifica* CNT603 | 2515154185 | 9 | CYP107AW7 | 2515169054.c00002_B175DRA...region2 | Bacteriocin |
|  |  |  | CYP164C2 | 2515169055.c00003_B175DRA...region1 | NRPS |
|  |  |  | CYP208A22 | 2515169081.c00029_B175DRA...region1 | T1PKS |
|  |  |  | CYP244A10 | 2515169083.c00031_B175DRA...region1 | NRPS |
|  |  |  | CYP107AY9 | 2515169083.c00031_B175DRA...region1 | NRPS |
|  |  |  | CYP161N4 | 2515169086.c00034_B175DRA...region1 | T3PKS |
|  |  |  | CYP2054A3 | 2515169086.c00034_B175DRA...region1 | T3PKS |
|  |  |  | CYP107FH3 | 2515169086.c00034_B175DRA...region1 | T3PKS |
|  |  |  | CYP107E37 | 2515169101.c00049_B175DRA...region1 | T1PKS |
| *Salinispora pacifica* CNY331 | 2518645627 | 10 | CYP208A22 | 2518683836.c00002_C588DRA...region1 | T1PKS |
|  |  |  | CYP107AW7 | 2518683839.c00005_C588DRA...region2 | bacteriocin |
|  |  |  | CYP105H11 | 2518683861.c00027_C588DRA...region1 | T1PKS |
|  |  |  | CYP244A10 | 2518683868.c00034_C588DRA...region1 | NRPS |
|  |  |  | CYP107AY9 | 2518683868.c00034_C588DRA...region1 | NRPS |
|  |  |  | CYP161N4 | 2518683871.c00037_C588DRA...region1 | T3PKS |
|  |  |  | CYP2054A3 | 2518683871.c00037_C588DRA...region1 | T3PKS |
|  |  |  | CYP107FH3 | 2518683871.c00037_C588DRA...region1 | T3PKS |
|  |  |  | CYP247A7 | 2518683873.c00039_C588DRA...region1 | NRPS |
|  |  |  | CYP107E37 | 2518683908.c00074_C588DRA...region1 | T1PKS |
| *Salinispora arenicola* CNT859 | 2517572233 | 13 | CYP211C1 | 2517601328.c00003_D462DRA...region1 | T2PKS |
|  |  |  | CYP154M15 | 2517601333.c00008_D462DRA...region1 | ladderane |
|  |  |  | CYP125G6 | 2517601333.c00008_D462DRA...region1 | ladderane |
|  |  |  | CYP107FS2 | 2517601333.c00008_D462DRA...region1 | ladderane |
|  |  |  | CYP105CN1 | 2517601333.c00008_D462DRA...region1 | ladderane |
|  |  |  | CYP105CP2 | 2517601333.c00008_D462DRA...region1 | ladderane |
|  |  |  | CYP208A12 | 2517601335.c00010_D462DRA...region1 | T1PKS |
|  |  |  | CYP166A4 | 2517601338.c00013_D462DRA...region1 | T1PKS |
|  |  |  | CYP1051A1 | 2517601340.c00015_D462DRA...region1 | Terpene |
|  |  |  | CYP105G5 | 2517601340.c00015_D462DRA...region2 | T1PKS |
|  |  |  | CYP107Q4 | 2517601340.c00015_D462DRA...region2 | T1PKS |
|  |  |  | CYP154M21 | 2517601344.c00019_D462DRA...region1 | NRPS |
|  |  |  | CYP154M13 | 2517601344.c00019_D462DRA...region1 | NRPS |
| *Salinispora arenicola* CNB458 | 2517572210 | 15 | CYP208A12 | 2517600114.c00003_B162DRA...region1 | T1PKS |
|  |  |  | CYP211C1 | 2517600115.c00004_B162DRA...region1 | T2PKS |
|  |  |  | CYP245A7 | 2517600117.c00006_B162DRA...region1 | Indole |
|  |  |  | CYP244A4 | 2517600117.c00006_B162DRA...region1 | Indole |
|  |  |  | CYP1051A4 | 2517600118.c00007_B162DRA...region1 | Terpene |
|  |  |  | CYP107AX-fragment8 | 2517600119.c00008_B162DRA...region1 | ladderane |
|  |  |  | CYP107FS2 | 2517600120.c00009_B162DRA...region1 | NRPS |
|  |  |  | CYP105CN1 | 2517600120.c00009_B162DRA...region1 | NRPS |
|  |  |  | CYP105CP2 | 2517600120.c00009_B162DRA...region1 | NRPS |
|  |  |  | CYP105CT2 | 2517600121.c00010_B162DRA...region1 | terpene |
|  |  |  | CYP154M5 | 2517600121.c00010_B162DRA...region1 | terpene |
|  |  |  | CYP105CH2 | 2517600135.c00024_B162DRA...region1 | T1PKS |
|  |  |  | CYP105G7 | 2517600137.c00026_B162DRA...region1 | T1PKS |
|  |  |  | CYP107Q4 | 2517600137.c00026_B162DRA...region1 | T1PKS |
|  |  |  | CYP107EU2 | 2517600138.c00027_B162DRA...region1 | T1PKS |
| *Salinispora arenicola* CNH877 | 2519103192 | 23 | CYP208A12 | 2519123878.c00001_F581DRA...region2 | T1PKS |
|  |  |  | CYP211C1 | 2519123882.c00005_F581DRA...region1 | T2PKS |
|  |  |  | CYP245A7 | 2519123884.c00007_F581DRA...region1 | Indole |
|  |  |  | CYP244A4 | 2519123884.c00007_F581DRA...region1 | Indole |
|  |  |  | CYP1051A1 | 2519123885.c00008_F581DRA...region1 | Terpene |
|  |  |  | CYP105G5 | 2519123885.c00008_F581DRA...region2 | T1PKS |
|  |  |  | CYP107Q4 | 2519123885.c00008_F581DRA...region2 | T1PKS |
|  |  |  | CYP105CP2 | 2519123887.c00010_F581DRA...region2 | NRPS |
|  |  |  | CYP105CN1 | 2519123887.c00010_F581DRA...region2 | NRPS |
|  |  |  | CYP107FS2 | 2519123887.c00010_F581DRA...region2 | NRPS |
|  |  |  | CYP125G6 | 2519123887.c00010_F581DRA...region2 | NRPS |
|  |  |  | CYP154M15 | 2519123887.c00010_F581DRA...region2 | NRPS |
|  |  |  | CYP107AX14P | 2519123888.c00011_F581DRA...region1 | ladderane |
|  |  |  | CYP166A4 | 2519123889.c00012_F581DRA...region1 | T1PKS |
|  |  |  | CYP105CH1 | 2519123898.c00021_F581DRA...region1 | T1PKS |
|  |  |  | CYP154M20 | 2519123900.c00023_F581DRA...region1 | oligosaccharide |
|  |  |  | CYP248A2 | 2519123900.c00023_F581DRA...region1 | oligosaccharide |
|  |  |  | CYP105W2 | 2519123900.c00023_F581DRA...region1 | oligosaccharide |
|  |  |  | CYP154M13 | 2519123900.c00023_F581DRA...region1 | oligosaccharide |
|  |  |  | CYP154M21 | 2519123900.c00023_F581DRA...region1 | oligosaccharide |
|  |  |  | CYP107EU1 | 2519123905.c00028_F581DRA...region1 | NRPS-like |
|  |  |  | CYP162P1 | 2519123908.c00031_F581DRA...region1 | NRPS |
|  |  |  | CYP107AY2 | 2519123913.c00036_F581DRA...region1 | NRPS |
| *Salinispora arenicola* CNS673 | 2519103185 | 17 | CYP208A12 | 2519123544.c00003_B127DRA...region2 | T1PKS |
|  |  |  | CYP154M5 | 2519123545.c00004_B127DRA...region1 | T1PKS |
|  |  |  | CYP105CT1 | 2519123545.c00004_B127DRA...region1 | T1PKS |
|  |  |  | CYP107AY2 | 2519123545.c00004_B127DRA...region2 | NRPS |
|  |  |  | CYP211C1 | 2519123546.c00005_B127DRA...region1 | T2PKS |
|  |  |  | CYP245A7 | 2519123547.c00006_B127DRA...region1 | Indole |
|  |  |  | CYP244A4 | 2519123547.c00006_B127DRA...region1 | Indole |
|  |  |  | CYP105W2 | 2519123549.c00008_B127DRA...region1 | oligosaccharide |
|  |  |  | CYP248A2 | 2519123549.c00008_B127DRA...region1 | oligosaccharide |
|  |  |  | CYP107FS2 | 2519123549.c00008_B127DRA...region1 | oligosaccharide |
|  |  |  | CYP105CN1 | 2519123549.c00008_B127DRA...region1 | oligosaccharide |
|  |  |  | CYP105CP2 | 2519123549.c00008_B127DRA...region1 | oligosaccharide |
|  |  |  | CYP1051A1 | 2519123553.c00012_B127DRA...region1 | Terpene |
|  |  |  | CYP105G5 | 2519123553.c00012_B127DRA...region2 | T1PKS |
|  |  |  | CYP107Q4 | 2519123553.c00012_B127DRA...region2 | T1PKS |
|  |  |  | CYP105CH1 | 2519123561.c00020_B127DRA...region1 | T1PKS |
|  |  |  | CYP107EU1 | 2519123566.c00025_B127DRA...region1 | T1PKS |

At the JGI IMG/M database, two strains of *Salinispora arenicola* CNH996 are available. Thus we provided the genome ID available for one species. The second species is indicated with B at the end of its name.

Information on species with no smBGCs

| Genome Name / Sample Name | Genome ID |
| --- | --- |
| *Salinispora arenicola* CNY685 | 2563366734 |
| *Salinispora arenicola* CNY230 | 2561511115 |
| *Salinispora arenicola* CNQ884 | 2561511039 |
| *Salinispora pacifica* CNY498 | 2563366539 |
| *Salinispora pacifica* CNY703 | 2563366517 |
| *Salinispora arenicola* ATCC BAA-917 | 2548876908 |
| *Salinispora arenicola* CNR921 | 2515154203 |
| *Salinispora pacifica* CNT851 | 2517572162 |
| *Salinispora pacifica* CNT124 | 2517572159 |
| *Salinispora pacifica* DSM 45544 | 2517287019 |
| *Salinispora pacifica* CNY646 | 2563366531 |
| *Salinispora pacifica* CNY673 | 2563366533 |
| *Salinispora arenicola* CNY281 | 2561511103 |
| *Salinispora pacifica* CNR909 | 2561511038 |
| *Salinispora arenicola* CNY244 | 2561511110 |
| *Salinispora tropica* CNY681 | 2561511108 |
| *Salinispora arenicola* CNH718 | 2561511105 |
| *Salinispora arenicola* CNY679 | 2561511113 |
| *Salinispora arenicola* CNT-088 | 2548876909 |
| *Salinispora pacifica* CNY666 | 2563366532 |
| *Salinispora tropica* CNY678 | 2561511109 |
| *Salinispora pacifica* CNY363 | 2563366534 |
| *Salinispora arenicola* CNY486 | 2561511107 |

# **Table S2**: P450 sequences identified and annotated in Salinispora species. Each P450 is presented with its assigned name followed by gene ID (in parenthesis) and species name

## **Full-length P450s**

>CYP166A4(2519873113)*Salinispora arenicola* CNR107

MTDAISFELPWARTDKFDPPAVFDALREQRPLARMRYPDGHVGWIVSSYE

LVREVLGDPRFSHSCAVGHFPVTHQGQVIPTHPQIPGMFIHMDPPEHTRY

RRLLTGEFTVRRTSRLTGHVEGVATEQIEVMREHGAPADLVATFARPLVL

RVLSGLVGLPYGERDRYLHAVTLLHDAEADPAEAAAAYEQAGAYFDEVIE

RRRRQPEDDLISTLVGDGELTGEELRNIVTLLLFAGYETTESALAVGMFA

LLHHEDQLARLRADPTKIDAAIEELLRYLTVNQYHTYRTASEDIELHGEV

INKGDSVTVSLPAANRDPARFACPAELDIDRETSGHVAFGFGIHQCLGQN

LARVELRAGLSALLRAFPNLRLAVPADEVPLRLQGSVFAVKNLPVCW

>CYP105BL2(2519874224)*Salinispora arenicola* CNR107

MSSHSAAAPDPETATPLHTLAPELTFPQFERSTPFDPPQAYTELSGRCPV

APVSMADGKPSWLITSFEGVRTTLSDPRFSSDMSHPGFPNRTGKPVDDLL

KDTLGAMDGERHRYYRRMLTGELTVRRAKAMRPVITQITDEALDQLAAAG

PGADLVKHVAFVVPSRVACHLVGIPLSDYELFTGMAATLMDSTSSDDQFA

ALQNMVSYFDTLVTDREHHDRDDLLGHMVRRYLATGELTRDMLIRLAWTT

MAAGQETTAHMIGLGVAALLRHPDQLELLRREPHLLPGAVDELMRYLPLI

QFGIPRVAMDDVEVDGQTVTAGEGVVALPPLANRDPAVFERPDELDVRRN

ARQHLTFGYGPHQCPAHALARLELEVVYGRLLERFPTLRLADSDADLKVQ

DKDIMYRVSELAVTW

>CYP105AB8(2519874485)*Salinispora arenicola* CNR107

MTETASSRLTDTEFPVQRECPFAEPVEYEQIREQSSIAMVRLTGGGEAWW

ISGHEQGRAVLADRRFSSDRRKANFPFVSTDPAIRKRLHAQPLSLISMDG

AEHTQARRALIGEFTVRRLAALRPRIQQIVDQCIDEMLTTDQHRADLVKA

LSLPVPSLVICELLGVPYADHDFFQEHTATLVRRNTASEVRQHSIDELNA

YLGALIDRKLASPDDDLLGRQIARQHRDGTFDRSSMVSLAFLLLVAGHET

TANMISLGVVGLLQHPEQLAMIKDDPDKTPLAIEELLRFFTIVDSVTSRV

ATEDVRFGDTTINAGDGVVVSGLSADWDPTVFADPDRLDLERGARHHLAF

GFGPHQCLGQNLARLELQIVFDTLFHRIPTLRLAAPLDKIPFKTDAAIYG

ARELPVAW

>CYP211C1(2519874594)*Salinispora arenicola* CNR107

VVDVEELLTRLYSAQGRQDPFPVYADLHAQGPIAALPPEPERRRVAAVAV

GYDLVGAVLRDPEWSKAPPPGWTEQEILRTLQTSMMFINPPDHGRMRHVF

AGTFTPRRLGALEPVVNRVADELLDRMADAGAGGLDFVAEFAYPLPARVM

AEFIGIPETELDWYRERVDVIDAFLDVAGKTPQRLAAANAAGAELRAFYG

ELLARRRRTPGEDLISGLVEAVDAGGVELTEDELVSNLIVLFNASFVTTV

YMLSNGLPVLLAHPEVAAALATDPVLTAGAVDEILRLQAPVHLLARAAPR

DTVLGGVPIPQGQNVLLLIAAANRDPAHFPDPDRFDPWRSGPPSLAFGLG

LHYCLGAAVSRLEGRLALPRLLSRFPRLRIMEQPVYSGSLFLRGIDKLSV

SPGEGSTRE

>CYP1005A1(2519875045)*Salinispora arenicola* CNR107

VSAVLFRSWTKTAGTRWPDVTRVADQSGTEHLVVTRHALVRQVLTDQATY

RPDNALEAVTPVPVAALRVLAGHRFRLPPTLANNGGASHPAIRALVADAL

HPTKVAAQRPWLTGLVADRVASIRTTLDSGGPVDLYADLTADLPLLVLAR

LVELPDAPVNAVKQFARAALELFWAPLDADRQLALADEVGRFHQVLREFA

DTGGGLAAALRATGHSPDVLVGALFFLLVAGQETTSQFLTLLLHRLSGEP

TIRAALRAGSSSVADVVEEGLRLEPPIVTWRRVAAVDSTLGGTTVAAGTS

VLLWLARAGRDPAVVAAPDEFRPGQRGSRRHLAFGAGAHRCLGDQLARME

AAVVVEQATPLLDGVTVVRPPWYPDNLTFRMPDAFVVRR

>CYP208A12(2519875628)*Salinispora arenicola* CNR107

MTLDTITPRVPLGPPRTAALRMLLVMKRDRLGMLTSAAARYGDASRLPVG

HKALWFFNHPRYAKHVLADNSANYHKGIGLVHARRALGDGLLTSEGDLWR

KQRKVIQPAFQSRRIAQQAGMIAEEAFALVERLRARAGAGPVELTAELTG

LTLGVLGRSLLDADLAGFDSIGDSFATVQDQAMFELETLNAVPMWIPLPR

QIRFRRARRKLQAVVDTLVDGRAGNLADRVDVLSRLILSARGEADPRVGR

ERLRDELVTLLLAGHETTASTLGWTLSLIDRHPGVWERLHAEAVEVLGDR

LPEYDDLRRLRYTVMVVEEAMRLFPPVWLLPRRALAPDTIGEYRVPANAD

VVISPYTLHRHPEFWPNPERFDPERFAPGQAADRPRYAYLPFGAGPRFCV

GNNLGMMEAVFVIALLCRHLRLTGVPGYRLVPEPMLSLRIRGGLPLVVRP

VS

>CYP245A7(2519876214)*Salinispora arenicola* CNR107

MPSATLPRFALTGWSRENIVNPYPVYQRYREVASVHRGESGGDAPDTFYV

FSYDEVVQVLSSNCFGRGRSLDAAKASVPVPAEQKALRAIVENWLVFMDP

PRHTELRSLLNRSFSPRIVTELRPRIARIAQELLSRLGQQVDVDLVESFA

APLPILVISELLGIPEERRAWLRANALALQEASSSRAGRDVDGYARAEVA

AQEFTEYFREQVRLRRGRAGGDLITILANAQERGAPVSLDAIVGTCVHLL

TAGHETTTNSLAKAVLALREHPAVLDELRGAEGLTTDAVEEFLRYDPPVQ

AVTRWAHQDTTLGGCDIPRGSRVVALLGSANRDPARFPSPDVLDVRRPAD

RHLSFGLGIHYCLGATLARAELEIGLQALLDGVPTLGYGTQHVDYADDLV

FHGPSRLVLVNLGERCT

>CYP244A4(2519876218)*Salinispora arenicola* CNR107

MSTTTNTELTEAPETNMPVDPGLFDCMPDLIAAARVAPVVRIPYLGRHAW

VVCDRELVKQALTHPKMGKDIALVPEWMRQPGLMVTAQPDPEYARAMIMS

DGENHARIRRIHAPVLSPRNTERWGERVADKVEGFLDELSQAGSGGSTEV

NVVTNYTHKIPLAFISEMLGLPPEAEHRLRGITDIMLYSSDYAARREAIG

GLFGAVEDWVRNPADLRDGVITGLLAASDGPDAAVTEGEVIVWTLGMIIT

GYETTGSLISTSLYEAIRRPPHERPKTDEDITAWIEETLRVHPPFPHPTW

RFPLEDIELGGYLIPKGAPVQVSIAAANRKPGEGADSFDAERRGHGHLSF

GLGMHYCIGAPLVRLEAQIAVRGFLRRFPQARLSAETAVQWESEWMIRRM

SVLPAVLS

>CYP1051A1(2519876836)*Salinispora arenicola* CNR107

MATDAAITRARTVPAWKALPAAVRDTHRAFVDVGNWSDGDVVRVSLGVSR

PYLVTNPAHVQEVLHERAAIYPRGDDTALWRSVRKLVGDGILAEGDAWAA

SRRVLAPMFRPARINAMVDTMADAIAGAVDDLHGAATAGTPIDVGRELSR

IVCSAIMRVFFADRITVRDALRIMKAQETIVTAMAPRILAPLVPWWIPMP

GDRRFRAAVRSIDDILLPVLRQAQRQPDDGDDLLSRLVRARADDGRALSE

KRMRDDLVSMVAVTTETSTVVLTWLWPLLANHPDVANRLYDEIDRVVGGG

PVRGDHLAELTYTRMVLDELLRLYPAGWILPRRAATTDVLGGVRINKGAT

VILSPYVTQRMTAWWGPTAEAFDPERFAAGREAADGRHRYAYYPFGVGMH

RCLGEHLFNLEAILIVATLLSRFRFALTDTSMPGVKVAASTRPARTVEMI

LKPVAPVPAR

>CYP105G5(2519876858)*Salinispora arenicola* CNR107

LTIETTETPPADDSLRAPLPRQFMQRDDPSKLPPALAALAEQSPVGRSTL

PDGDPFWMVSGYDEARAVLSDPRFSSDRFRYHPRFKKLSGQLGERLRNDK

ARAGSFINMDPPEHTRYRKLLTGQFTVRRMRQLTVRIEQIVTEQVDVMLA

EGNSADLVSAFAVPVPSLMICELLGVRYEDRTEFQRRAAGLLQTDLPIKQ

AVENLEAQRAFMQRLVTDKRRTPADDMISGLVHHAGAEPPLTDDELVGIA

TLLLFAGLDTTASMLGLGMFMLLQRPEQMAVLRDDPSRIGDAVEELLRYL

TVVSTGLFRFAKEDVVLGDEHIPAGSTVVVSLMAANRDGRHWPEPETLDV

TRVRSSHLAFGHGVHQCLGQQLARIELTVGITELLRRLPNVRLAVPPADV

PLRNDMITYGVHRLPILWDTP

>CYP107Q4(2519876859)*Salinispora arenicola* CNR107

MTTTAETSAETIDLFSPEVVADPFGWYARLREETGPTTGTLNIGTMMGGP

EMWLVTRYEDVRQVLTDPRFLTNPPADSPLEDIRAGVFKRLDFPPDLIPW

MANLLNVSDGEDHTRLRKLVSYALTAHRIGKLRPRVEKITADLLDKLAED

GKDGSPVDLVEEYCYPLPVTVICELVGIDEPDRPHWRAWGDSMATMNGER

IPTTLVKCIELARELIAKRRAEPQDDLVTALVQAQAEDQNRVSDDEIIGI

LFSLVTAGHQTTTYLIGNSVILLLENPDQLARLKENPSMWPQAVRELQRL

GPIQFAQPRFPSEDIELGGVTIPRGAPVAPLLLAANTDPRRFPDPNKLII

DRLAVGSEGHLGFGKGIHRCLGQHLAYQEAEVALQGLFTRFPDLSLAVPR

EEIPWILRPGFTRTRTLPLKLV

>CYP154M5(2519876900)*Salinispora arenicola* CNR107

VEQSCPYKLDVTGRDVHAEGEAIRARGPVAQVELPGGVQGWSVTGYQAAR

QVLADPRFAKDPKKWPAYTSGAIPPNWPLIGWLLMDNMTTNDGADHQRLR

KLVSHGFTPRQVERTRPLIVKIVNDLLDGLSSAGPDEVVDLKGRFATPLP

ARVICDMFGVPEALRASVLRGAQVNVTSSISGEEAEANVEQWHRELLELV

EAKREKPDEDMASLLIAAKEEDGSTLTQEEVVGTLHLMLGAGSETLMNAL

SYAVLGMLSNPGQYEMVRNGTSSWDDVIEETLRAQAPVAQLPLRYATEDV

AVGGAVIKAGDPVLMGFTAIGRDPAVHGETAGDYDITREDKTHLSFGHGV

HFCLGAPLARLELKIALPALFERFPNMTLAVRPDQLEPQGTFIMNGHREL

PVRLGQPATVLA

>CYP105CP2(2519877182)*Salinispora arenicola* CNR107

MTKSMPVQDLPAFPIPRECPYRPSAQHVSLRSGGPMAKVRLYNGRTAWLV

TDSAHARAVLSDYRRVSIKPYHGNYPLLNEEFEKVVDSGYADVLFGVDPP

EHTRQRQMIMPSFTLRRTAVLRPDIQRIVDDKLDEMMRHGAPGDLVTEFA

QPVPSMVMSFLLGVPWEDHEEFETPAHKLFVPELAEEATTELGAYLERLI

QKKEQPGGTPGGTGLLDDLIRDHLRAGALSRDELVHIAMAMLVAGTDTTT

NVISLGTLALLDNPDQWAALRDNPDELIPGAVEEILRYTSLIEAFARVAV

SDIELNGAVIKEGEGILISSAGVNFDPALAPDPGRFDIRRPPRPSFSFSH

GIHRCPGDNLARLELEIAFRSLVTRMPNLRTAKPIDQIPSNNNDGTLQRL

YELPVVW

>CYP105W2(2519877313)*Salinispora arenicola* CNR107

MTGYQDRPTGDQPGAPVPSGSTDPGIGAFPLPRRCPFSPPAEYARLRAEH

PVVRLPMLGGDTAWVVSRHADVRQVLSDPRMSADRRRPGFPKFAPTTEGQ

RQASFANFRPPLNWLDPPEHAICRRQIVDEFSVRRVRQSRALVERVVDTH

LDALTAAAPGADLVSTFAYPVPSQVICEVLGVPYGEHEFFERRSTLMFRR

STPADERARCAREIRDFLDVVVTDKERRPGDDVLSRLLYRQRRAGGVDHE

AVVSMAFVLLVAGHVTTSNMIALSVLALLTHPARLARLRAEPERFPAAVE

ELLRYFTVVEAATARTTTAEVTIGGVTIAAGEGVVALGQAANRDPRVFEH

PDEFDPDRDARAHLAFGHGRHICPGQHLARLEMEVALSRLFRRLPGLRLT

MEVSDLPLKEDSNIFGLYALPVAW

>CYP105J3(2519877756)*Salinispora arenicola* CNR107

MTDSVAFPQGRVCPHQPAPGYRPLAVQRPLAQVTLYDGRRVWAVTTRDLA

RRLLVDPRISSDRTNPAWPAIVPIVAAAVNDAQQKVLKIATALVGTDGPE

HKAQRKMLIPSFTFRRMNALRPMIQEIVDQQLDEMIKSGAPTDLIPAFAS

AVPVTVLYRLMGIPDDDHGIFEKLSHQLLAGPNANEAYDQLMGYMSRLIA

ERRRNPGEGVLDDLLAQHGANDDADHDELVSTLVVQVAGNHGTTGSMIAL

GLFALLQHPEQLAELRADPSLMPTAVDELLRFLSVPDAVTRLAADDIEVE

GTIIRQGDGVFFITSLINRDTDVHDAPNSLGWHHASAADHLTFGFGAHQC

LGQSLARITMEIALGALIDRLPSLRLAVPAEEVPFLPAASLQVIAELPIT

W

>CYP211B2(2519877904)*Salinispora arenicola* CNR107

MDVSEAIAVLISPSGRLDPYPTYEQLRAHGPVSQTTAGLFVVTGYAEADM

VLRDPRFVVLDDDLRDDVFPHWQDSPAIKSIARSMLRTNPPDHSRIRRLA

AGAFTPRRVAAMREVVTAQADELVDEMIRAGRDGARVDFMDMFAYPLPVA

VICALLGVPAADRSRFRRWAGDLTGILEPEITPEELAGADAGADELRDYF

TGLIEQRRRAPADDLTTALVQAHDADGDRLSGEELLANLVVLLVAGFETT

TNLLGNGLVVLLTRPEAAAALRDEPDLAPGYVDELLRYDSPVQLTTRTVR

ESVSFAGTELPADSWLLVLLGAANRDPRRFPDPARFDPGRAQSQPLSFGA

GPHYCLGAGLARLEAQVAFPLLLRRLPELALAGRPSRRTRLTLRGYETLP

ITVGAVTADRGTPAGVAPGTP

>CYP125A41(2519874742)*Salinispora arenicola* CNR107

MTEPRIPAGFDFTDPEVLAHRVPREEFAELRRTAPVWWNAQPRGSAGFDD DGYWVVTRYADVMTVSRDSDTYSTRENTAIARLRPDTTREDIEMQRVIML NVDPPEHTKLRAIVSRGFTPRAINALRGSLAERAEHIVRDAAVRGVGDFV TDVACELPLQAIAELIGVPQHHRRKVFDWSNQLIGYDDPAYGTDPLTASA ELLAYAMEMAEERQRSPSDDLVTKLVNAQIDGEHLTTDEFGFFVMLLAVA GNETTRNAITHGMVAFLDNPEQWELFKAERPKSAVEEIIRWATPVNVFQR TALVDTVLGGQAISAGQRVALFYGSANFDEAVFEDPERFDITRSPNPHLG FGGSGAHFCLGANLARLEIELIFNSIADHLPDIRKVAAPQRLRSGWINGI

RQMPVRYR

>CYP105CT1(2519876883)*Salinispora arenicola* CNR107

MNSPNHMPADRSLTAPASGCPMALSRGRVGLDVADEISELRDGGRLGRIT TAFGQEATLITRYDEVRAQMADSVVFNVAGVPSPPALVDGGFDTESVRRR RTVGNLIMLDPPEHTRLRRMVAAWFTTRRVERLRPRVVEIIDAALDEMER SGPPVDLVAMFAKTVPITVICELIGVPEELRERYRRRAERAVSASAVSTP LDELRRLREAGWVSRELIEYHRENPSDDIIGMLLREHGTDSHDDGITDDE LVGLANALLIAGHETTTQMLSMGTLALLRHPDQLALLRDDPSIVAGAVEE LLRYVGVLHGGFVRVATRDTRLGGHRIHAGELVVPALTAANRDPRLLTDG

>CYP107FS2(2519877179)*Salinispora arenicola* CNR107

MPVPQGEQNLTTEVFADPKALFATLGSRQPLHRISLPDGMPAVLVTGNRE ARQALSDPRLVRSITAAAPELHKYHPLASDDYALSRHMLFADPPDHGRMR KLVSTAFTRRRVEQMRPRIQQITDDLIDVIAAKGEADLVETLALPLPIAV ISEMLGVPFADRSEFERHAEVLTGINASSGFDAIIAAGRWFDEYLAELVQ QRRREPQDDLISGMLAAQDKGDRLTDVELRSNALLLLSAGFETTVNLVAN GLLALLRHPEAMAALRSEPNLMTTAVDELLRYDSPVSCVTYHFAQEPVEI GGFKIRSGEHVVIAAAAANHDPTVFADPSRLDLRREGSGQILSFSHGIHF CLGAPLARLEGEIAFGTVLRRLAGLRLAVPADSLVWKASFVLHRLERLPV

TFTPDRAPNPIDSVHTV

>CYP105CN1(2519877180)*Salinispora arenicola* CNR107

MAAPAPQATQSTTPHPPSYPLPRECPYRPSAGTARLRDAGPVSTVRLYDG RTAWLVTGAAEARALLADSRVSNRADFPNYPVMDERHLSMRATREMAREE EGGFAAALFGMDPPEHTRQRQLLLPRFTVRQVAARRPAIQRIVDEHLDAM EANGSPADLVSAFATPVPTMVVCTHLGVPYQDRTRFEPAVAGLFEPDRAD AAMAELTAYLHQLIETKQSEPGDGVIDHLIANHLRPGAIDRAELVAIASA ILVAGTVTTSSAIALGTLALLTAPGQYAALVDNPDLVPGAVNEILRYLSL VEQLARVATEDIEIGGKLIRAGDGIIVSFAAGNLDPNVTTHPDRLDVALP PTNHLAFSHGIHHCIGQNLALLELDIAFRALVSRFPTLRLAVPAEQLPTYFAGDVPRLACLPVTW

>CYP248A2(2519877306)*Salinispora arenicola* CNR107

VLADAVTAFDPTAVDVRRDPYPSYHWLLRHDPVHRGAHQVWYVSRFADVR AVLGDERFARTGIRRFWTDLVGPGLLSQIVGDIILFQDEPDHGRLRGVVG PAFSPSALRRLEPTIEATVNDLLRPARALGAMDVVADLAYPLALRAVLEL LGLPAGDANAVGRWSRAVGRTLDRGATAEDMRRGHAAIAEFADYVERMLA ERREDGADLLALMLAAHRSQLMSRNEIVSTVVTFIFTGHETVASQLGNGL LSLLDHPEQLELMRRQPHLVPQAVEECLRFDPAVQSNTRQLAADVELHGR RLRRDDVVVVLAGAANRDPGRYDRPDELDIRRDPVPSMSFGAGMRYCLGS YLARLQLRTALGAMVALPDLRLACSPNELAYQPRTMFRGLTRLPVAFTPAG

>CYP107AY2(2519877799)*Salinispora arenicola* CNR107

MTAEPTPIPRSGARLGQEYDQLRKTGDVHQVLLPDASLAWLVTNPEVAAR ALADPRLALNRRNSRGGWSGFALPPALDANLLNLDAPDHTRLRRLVGPAF SPQRVAALRPGIRRAAEHLLDTLVATSGPTDLVTGYCNPLSVQVIADLMG VPEAGRTNLRAWTDTMLTSYPPDRDAIRQAVTELHGYVVDLIDIKQQQPG DDLLSALVTIEQDGDRLSRDELTSLAFLILFAGYENTANLIASAVLWLLD HGGLNVVPSSEAIEGTLRHEPPAPVAIRRFPTEDIIIGGVTIPAGDTVLL SIAAATRGADGNAARLAFGNGPHYCLGAALARVEAEEALTVLARRLPGLT LAVPPSQVRWRPTFRTHGPAELLVGW

>CYP107AW9(2561677780)*Salinispora pacifica* CNS801

VETVTGTSAPPPVPYIADPYPTLARIRANGPVSILHSDEGLPMWVIARYQ

DVRAALADPRFGQDSRRAQALADNRVAGVTLGGDIVHMLNSDPPDHTRLR

RHVQGAFTARRVAAMRPLVERITTSLLDGLAGRTTVDLVQDFAFPLPMLV

ICELLGFPAEERNVYRSWSTAILTHDDDPTAFATALREMTDYIAVQLRIR

QTRPGDDLLTELLAARDAGQLTDDEIIGMVFLLLIGGHETTVNLLGTATL

ALMRNPDQHRWLLANQPALPEAIDEFLRYESPVAMATLRFTTTPVAVDDV

VIPAGELVLVSLGGANRDPDRFPDADRLILNRHDTGHLAFGHGLHRCLGA

LLGKLEGEVALGALLRRYPRLALAAEVRQLRWRDTIMLRGLESLPVSLHG

>CYP107AX11(2561677791)*Salinispora pacifica* CNS801

MTSPPAPVFDQLLLRDPHRRYNALRDEAPVHHIRTPDGAPAWLVTRYDDV

RAAFTDPRLSVDKRLSSTDGEHGSSLPPELDAHLLNRDPPDHTRLRRFAA

AAFTPRRVADLRPAVERIVSTLLDGLAGNDHAELIGSLASPLPLQVMQEL

LGLPTQTSVDFRTWTNTLLSADANQPAQSRSAMASMRRFLIEQLAHKRAR

PGDDLLTGLLAPHADGDRLSDDELVAMLFLLMFAGYDNTAALIGNVAHAL

LTNAELAEAVRTGSLAVDELVDGVLRWNPSFPLAVRRFAREPVTIAGQLI

PAGDRIWLCLASANRDPAQFTEPDTLGTTGMRCPHLSFGHGIHYCLGAPL

ARLQTTVAITSLLDRFPGIRLAVPAQDIRWRESFRLRGLVALPVFLSDGC

GSGGTYG

>CYP1223A5(2561678741)*Salinispora pacifica* CNS801

MREVGRAVQALFGPAGCAEPYPHYEALRRAAPVCPLPDNIVLVSRYAEAE

RVIRDPRFLMEDAAWIGQSHPGWTEHPSFRSLMLEMVNHNPPDHARLRRL

VSRAFTPRRVAELRPAVEQLIDDLLDQMAAREGGTADFMADFALPLPVTV

IGELLGIPPADRIWFAPRVEAFTDAIEESMRGPSLAEADQAVKELWARLG

ELADERRRNPRNDMVSELVSVSDSGDDRLSHDELLANLVLLYAAGYETTS

NLLGNGLAALLERPALKERLRQNPTQASAFVEEMLRFDPPIQIATRYTAQ

ATTLGGLNIDAGTQIIVLIGSANRDPDRFADPDEFDPDRADGGSMVFGVG

AHYCLGAALARMEAEIAFPRLLDRFPAITGHAPGVRRRKSLRGFTQLPIS

LS

>CYP105AB33(2561678948)*Salinispora pacifica* CNS801

MTETASITTAGASSTATSGPGSGEVTDAEFPVERGCPFSTPAEYEQIREH

APLAKVRLTTGREAWWIAGHELARAVLADRRFSSDRRRDNFPFVSTDPET

RKQLQDQPTSMLGMDGAEHAQARRALMGEFTVRRMAGLRPRIQQIVDQHI

DEMLSSEQRSADLVEALSLPVPSLVICELLGVPYADHDFFQAHTGPLVRH

NTPSQVRVRIQEELNTYLGGLIDRKVADPTDDLLSRQIAKQRAAGTFDRT

SMVSMAFLLLIAGHETTANMISLGVVGLLQHPDQLAKIKEDPEKTPPAVE

ELLRYFSITDTVTARVATEDVQLGDTTINAGDGVVISGLAADHDPTVFTD

PNQIDLERGARHHVAFGFGPHQCIGQTLARLELQIVFDTLFRRIPTLRLA

APLDDIPFKSDAIIYGAEELPVAW

>CYP211B13(2561679662)*Salinispora pacifica* CNS801

MDASEAVALLMSPPGRLDPYPTYERLRAHGTVVPTAAGFFVVTGYTEADT

VLRNPRFGVMDDEEREGVFPHWQESPAMMSISQSMIRANPPDHSRMRRLA

AKAFTPRRVAALREVVAAQADGLVDGMIRAGRGGAPVDFLGSFAYPLPVA

VICALLGVPAADWAQFRRWASDLTGVLEPEITPQELAVADAGARELRDYF

TELVAQRRRTPADDLTTALVQAHDADGDRLSGEELLANLVLLLVAGFETT

TNLLGNGLVVLLNHPDSATALRDQPELAPGYVEELLRYDSPVQLTTRTVR

ESVPLAGVELPAGSWLLVLLGAANRDPARFTDPARFDPRRAQSPPLSFGA

GAHYCLGAGLARLEAQVAFPLLLRRLPELALAGEPTRRNRLTLRGYETLP

VTVGAVAADHGTPAGVALGTP

>CYP211C4(2561680802)*Salinispora pacifica* CNS801

VPDIEGLLTRLYSAPGRQDPYPVYADLHAQAAIAALAPRPERQRVAAVAV

GYDLVAAVLRDPEWGKQPPPGWTDQEILRTLQSSMMFINPPDHGRMRHVF

AGTFTPRRLGALAPVINRVTDELLDRMADAGPGEVDFVAEFAYPLPARVM

AEFIGIPATELTWYRERVDRIDAFLDVAGKTPERLAAANAAGAELRAFYR

ELLAHRRRMPGEDLISGLVEAVDAGGVELTEEELISNLIVLFNASFVTTV

YLLSNGLPVLLAEPKVAAALADDPELTAGAVDEILRLQTPVHLLARAAPR

DTVLGGVSIPQGQNVLLLIAAANRDPAHFPDPDRFDPRRSGPPSLAFGLG

LHYCLGAAVSRLEGRLALPRLLSRFPRLRILEQPVYSGSLFLRGIDRLSV

SPGGRSIRE

>CYP125A66(2561680927)*Salinispora pacifica* CNS801

MTEPRIPKGFDFTDPAVLERRVPREEFAELRRTAPVWWNAQPKGSAGFDD

DGYWVVTRYADVMAVSRDSETYSTRENTAIARFQPGTTQADREMQRVIMI

NVDPPEHTKLRAIVSRGFTPRAINALRGSLAERAERIVRDAAARDTGDFV

TDVACELPLQAIAELIGVPQHHRRKVFDWSNQLIGYDDPAYGVDPMAASA

ELLAYAMEMAHERQRNPSDDLVTQLVNAQIDGEHLTADEFGFFVMLLAVA

GNETTRNAITHGMLAFLENPEQWELFKAERPRSAVEEIIRWATPVNVFQR

TALVDTTLAGQAISAGQRVALFYGSANFDESVFEEPERFDITRSPNPHLG

FGGSGVHFCLGANLARLEIELIFNSIADHMPDIRQLADPQRLRSGWINGI

RELPVQYH

>CYP1005A6(2561681064)*Salinispora pacifica* CNS801

VSAVLFRSWTKTAGTRWPAVTRVADRQGTEHLVVTEHALVRQVLTDQVTY

RPDNALDAVTPIPVTALRVLAGHRFRLPPTLANNGGVNHPAIRALVADAL

HPAKVAAQRPWLTELVAERVAAIRATLDSGGSADLHAELSADLPLLVLAR

LVELPDAPVSAVKQFARAALELFWAPLDADRQLALADEVGRFHQVLREFA

DTGGGLAAALRATGHPPDVLVGALFFLLVAGQETTSQFLTLLLHRLAGEP

TVRAALRDGSVSVTSVVEEGLRLEPPIVTWRRVAAVDSTLGGTAVPAGTS

VLLWLARAGRDPAIVPAPDEFRPGQRGSRRHLAFGAGAHRCLGDQLARME

AAVVVEQATPLLDGVTVVRAPWYPDNLTFRMPDAFVIRR

>CYP107AY10(2561681613)*Salinispora pacifica* CNS801

MYQDQPTRAQLAPIPRSGALLGQEYDQLRKTGDVHQVLLPDTSMAWLVTS

PELVSRALADPRLALNRKHSRGGWSGFALPPALDANLLNLDAPDHTRLRR

LVGPAFSPQRVAALRPRIQRTAEELADTVLATGSPVDLVTGYCTPLSVQV

IADLLGVPEAGRTDLRAWTDTMLTSYPPDRDAIRRAVVELHGYVVNLIDA

KQQRPGDDLLSALVATEQEGDRLTRDELTSLAFLILFAGYENTANLIAST

VLRLLDHGSLRGVQASEAVEETLRLEPPAPAAVRRFPTEEMTIGGATIPA

GDTVLLSIAAATRGADGNSARLAFGNGPHFCLGAALARVEAEEAITVLAR

RLPSLALAVPGAPVRWRPTFRTHGPAELLVTW

>CYP1278B4(2561682054)*Salinispora pacifica* CNS801

MPSPVGAVVHPNPYPYYAAMVAERPFHFDEQLDTWVAASAAAAQAVLTAP

GCRVRPPHEPVPQGITGTPAGDVFGNLVRMTDGEPQHRLKAIVTQTLGAV

DRSTVAATAMRHARQVLNDSVRTPYEQLMFELPAQVVATLCGLDPAAGGE

ATRLVGHFVQCIPATASPEQQQRAARAAAGLQELLGSKLDDTQHGLLGDL

VRMATHVGWTDQAPLLANGVGFLSQTYDATAALMGNTLLALSQQECELPI

SEMALQRFVREVIRHDAPIQNTRRFTATPIRHGDVEVPAGQAVLVLLAAA

NRDPAANPDPHMFRADRTAPNVFTFSAGAHHCPGETLAVTIVTTVVEQLL

RVGFDPAKLSTQVSYRPSPNARIPVLTD

>CYP166A4(2563829763)*Salinispora arenicola* CNY685

MTDAISFELPWARTDKFDPPAVFDALREQRPLARMRYPDGHIGWIVSSYE

LVREVLGDPRFSHSCAVGHFPVTHQGQVIPTHPQIPGMFIHMDPPEHTRY

RRLLTGEFTVRRTSRLTGHVEGVAAEQIEVMREHGAPADLVATFARPLVL

RVLSGLVGLPYGERDRYLHAVTLLHDAEADPAEAAAAYEQAGAYFAEVIE

RRRRQPEDDLISTLVGDGELTGEELRNIVTLLLFAGYETTESALAVGMFA

LLHHEDQLARLRADPTKIDAAIEELLRYLTVNQYHTYRTASEDIELHGEV

INKGDSVTVSLPAANRDPARFACPADLDIDRETSGHVAFGFGIHQCLGQN

LARVELRAGLSALLRAFPNLRLAVPADEVPLRLQGSVFAVKNLPVCW

>CYP208A12(2563830041)*Salinispora arenicola* CNY685

MTLDTITPRVPLGPPRTAALRMLLVMKRDRLGMLTSAAARYGDASRLPVG

HKALWFFNHPRYAKHVLADNSANYHKGIGLVHARRALGDGLLTSEGDLWR

KQRKVIQPAFQSRRIAQQAGMIAEEAFALVERLRARAGAGPVELTAELTG

LTLGVLGRSLLDADLAGFDSIGDSFATVQDQAMFELETLNAVPMWIPLPR

QIRFRRARRKLQAVVDTLVDGRAGSLADRVDVLSRLILSARGEADPRVGR

ERLRDELVTLLLAGHETTASTLGWTLSLIDRHPGVWERLHAEAVEVLGDR

LPEYDDLRRLRYTVMVVEEAMRLFPPVWLLPRRALAPDTIGEYRVPANAD

VVISPYTLHRHPEFWPNPERFDPERFAPGQAADRPRYAYLPFGAGPRFCV

GNNLGMMEAVFVIALLCRHLRLTGVPGYRLVPEPMLSLRIRGGLPLVVRP

VS

>CYP211B2(2563830260)*Salinispora arenicola* CNY685

MDVSEAVAVLTSPSGRLDPYPTYEQLRAHGPVSRTTAGLFVVTGYAEADM

VLRDPRFVVLDDDLRDDVFPHWQDSPAIKSIARSMLRTNPPDHSRIRRLA

AGAFTPRRVAAMREVVTAQADELVDEMIRAGRDGARVDFMDMFAYPLPVA

VICALLGVPAADRSRFRRWAGDLTGILEPEITPEELAGADAGADELRDYF

TGLIEQRRRAPADDLTTALVQAHDADGDRLSGEELLANLVVLLVAGFETT

TNLLGNGLVVLLTRPEAAAALRDQPDLAPGYVDELLRYDSPVQLTTRTVR

ESVSFAGTELPAGSWLLVLLGAANRDPRRFPDPARFDPWRAQSQPLSFGA

GPHYCLGAGLARLEAQVAFPLLLRRLPELALAGRPSRRTRLTLRGYETLP

VTVGAVTADRGTPAGVAPGTP

>CYP1051A4(2563830543)*Salinispora arenicola* CNY685

MATDAAITRARTVPAWKALPAAVRDTHRALVDVGNWSDGDVVRVSLGVSR

PYLVTNPAHVQEVLHERAAIYPRGDDTALWRSVRKLVGDGILAEGDAWAA

SRRVLAPMFRPVRINAMVDTMADAIAGAVDDLHGAAVAGTPIDVGRELSR

IVCSAIMRVFFADRITVQDALRIMKAQETIVTAMAPRILAPLVPWWIPMP

GDRRFRTAVRSIDNILLPVLRQAQRQPDDGDDLLSRLVRARADDGQVLSE

KRMRDDLVSMVAVTTETSTVVLTWLWPLLANHPDVADRLYDEIDRVVGGG

PVRGDHLAELTYTRMVLDELLRLYPAGWILPRRAAATDVLGGVRINKGAT

VILSPYVTQRMTAWWGPTAEAFDPERFVAGQEAADGRHRYAYYPFGVGMH

RCLGEHLFNLEAILIVATLLSRFRFALTDTSMPGVKVAASTRPARTVEMI

LKPVAPIPAR

>CYP244A4(2563830921)*Salinispora arenicola* CNY685

MSTTTNTELTEAPETNMPVDPGLFDCMPDLIAAARVAPVVRIPYLGRHAW

VVCDRELVKQALTHPKMGKDITLVPEWMRQPGLMVTAQPDPEYARAMIMS

DGENHARIRRIHAPVLSPRNTERWGERVADKVEGFLDELSQAGSGSTEVN

VVTNYTHKIPLAFISEMLGLPPEAEHRLRGITDIMLYSSDYAARREAIGG

LFGAVEDWVRNPADLRDGVITGLLAASDGPDAAVTEGEVIVWTLGMIITG

YETTGSLISTSLYEAIRRPPHERPKTDEDITAWIEETLRVHPPFPHPTWR

FPLEDIELGGYLIPKGSPVQVSIAAANRKPGEGADSFEAERRGHGHLSFG

LGMHYCIGAPLVRLEAQIAVRGFLRRFPQARLSAESAVQWESEWMIRRMS

VLPAVLS

>CYP245A7(2563830925)*Salinispora arenicola* CNY685

MPSATLPRFALTGWNRENIVNPYPVYQRYREVAPVHRGEPGGDAPDTFYV

FSYDEVVQVLSSSCFGRGRSLDAAQASVPVPAEQKALRAIVENWLVFMDP

PRHTELRSLLNRSFSPRIVTELRPRIARIAQELLSRLGQQADVDLVESFA

APLPILVISELLGIPEERRAWLRANAMALQEASSSRAGRDVDGYARAEVA

AQEFTEYFREQVRLRRGSAGGDLITILANAQERGAPVSLDAIVGTCVHLL

TAGHETTTNSLAKAVLALREHPAVLDELRGADGLTTDAVEEFLRYDPPVQ

AVTRWAHQDTTLGGCDIPRGSRVVALLGSANRDPARFPLPDVLDVRRPAD

RHLSFGLGIHYCLGATLARAELEIGLQALLDGVPTLGYGTQHVDYADDLV

FHGPSRLVLINLGER

>CYP125A41(2563831037)*Salinispora arenicola* CNY685

MTEPRIPAGFDFTDPEVLAHRVPREEFAELRRTAPVWWNAQPRGSAGFDD

DGYWVVTRYADVMTVSRDSDTYSTRENTAIARLRPDTTREDIEMQRVIML

NVDPPEHTKLRAIVSRGFTPRAINALRGSLAERAEHIVRDAAVRGVGDFV

TDVACELPLQAIAELIGVPQHHRRKVFDWSNQLIGYDDPAYGTDPLTASA

ELLAYAMEMAEERQRSPSDDLVTKLVNAQIDGEHLTTDEFGFFVMLLAVA

GNETTRNAITHGMVAFLDNPEQWELFKAERPKSAVEEIIRWATPVNVFQR

TALVDTVLGGQAISAGQRVALFYGSANFDEAVFEDPERFDITRSPNPHLG

FGGSGAHFCLGANLARLEIELIFNSIADHLPDIRKVAAPQRLRSGWINGI

RQMPVRYR

>CYP211C1(2563831184)*Salinispora arenicola* CNY685

VVDVEGLLARLYSAQGRQDPFPVYADLHAQGPIAALPPEPERRRVAAVAV

GYDLVGAVLRDPEWSKAPPPGWTEQEILRTLQTSMMFINPPDHGRMRHVF

AGTFTPRRLGALEPVVNRVADELLDRMADAGAGGLDFVAEFAYPLPARVM

AEFIGIPETELDWYRERVDVIDAFLDVAGKTPQRLAAANAAGAELRAFYG

ELLAHRRRTPGEDLISGLVEAVDAGGVALTEDELISNLIVLFNASFVTTV

YMLSNGLPVLLAHPEVAAALATDPVLTAGAVDEILRLQAPVHLLARAAPR

DTVLGGVPIPQGQNVLLLIAAANRDPAHFPDPDRFDPRRSGPPSLAFGLG

LHYCLGAAVSRLEGRLALPRLLSRFPRLRIMEQPVYSGSLFLRGIDKLSV

SPGEGSTRE

>CYP154M5(2563831558)*Salinispora arenicola* CNY685

VEQSCPYKLDVTGRDIHAEGEAIRARGPVAQVELPGGVQGWSVTGYQAAR

QVLADPRFAKDPQKWPAYTSGAIPPTWPLIGWLLMDNMTTNDGADHQRLR

KLVSHGFTPRQVERTRPLIVKIVNDLLDGLSTAGPDEVVDLKGRFATPLP

ARVICDMFGVPEALRASVLRGAQVNVTSSISGEEAEANVEQWHRELLELV

EAKREKPDEDMASLLIAAKEEDGSTLSQEEVVGTLHLMLGAGSETLMNAL

SYAVLGMLSNPGQYEMVRNGTSSWDDVIEETLRAQAPVAQLPLRYATEDV

AVGGAVIKAGDPVLMGFTAIGRDPAVHGETAGDYDITRADKTHLSFGHGV

HFCLGAPLARLELKIALPALFERFPNMTLAVRPDQLEPQGTFIMNGHREL

PVRLGQPATVLA

>CYP105CT2(2563831574)*Salinispora arenicola* CNY685

MNSPNHMPADSSSTAPARGCPMALSRGRVGLDVTDEISELRDGGRLGRIT

TAFGQEATLITRYDEVRAQMADSAVFNVAGVPSPPVLVDGGFDTESVRRR

RTVGNLIMLDPPEHTRLRRMVAAWFTARRVERLRPRVVEIINAALDEMER

NGPPVDLVAMFAKTVPITVICELIGVPEELRERYRRRAERAASASAVSTP

LDELRRLREAGWVSRELIEYHRENPSDDIIGMLLREHGADSHDDGITDDE

LVGLANALLIAGHETTTQMLSMGTLALLRHPDQLALLRDDPSIVAGAVEE

LLRYVGVLHGGFVRVATRDTRLGGHRIHAGELVVPALTAANRDPRLLTDG

DRLDITRPPTSHVAFGHGVHFCIGAPLARMELREAFPALLRRFPGLRLAV

PDSELEFTQGTTVYSLRGLPVTW

>CYP107FS2(2563832156)*Salinispora arenicola* CNY685

MPVPQGEQNLTTEVFADPKALFATLGSRQPLHRISLPDGMPAVLVTGYRE

ARQALSDPRLVRSITAAAPELHKYHPLASDDYALSRHMLFADPPDHGRMR

KLVSTAFTRRRVEQMRPRIQQITDDLIDVVAAKGEADLVEALALPLPIAV

ISEMLGVPFADRSEFERHAEVLTGINASSGFDAIIAAGRWFDEYLAGLVQ

QRRREPQDDLISGMLAAQDKGDRLTDVELRSNALLLLSAGFETTVNLVAN

GLLALLRHPEAMAALRSEPNLMTSAVDELLRYDSPVSCVTYHFAQEPVEI

GGFEIRSGEHVVIAAAAANHDPTVFADPSRLDLRREGSGQILSFSHGIHF

CLGAPLARLEGEIAFGTVLRRLAGLRLAVPTDSLVWKASFVLHRLERLPV

TFTPDRDPNPIDSVHTV

>CYP105CN1(2563832157)*Salinispora arenicola* CNY685

MAAPAPQATQSTTPHPPSYPLPRECPYRPSAGTARLRDAGPVSTVRLYDG

RTAWLVTGAAEARALLADSRVSNRADFPNYPVMDERHLSMRATREMAREE

EGGFAAALFGMDPPEHTRQRQLLLPRFTVRQVAARRPAIQRIVDEHLDAM

EANGSPADLVSAFATPVPTMVVCTHLGVPYQDRTRFEPAVAGLFEPDRAD

AAMAELTAYLRQLIETKQSEPGDGVIDHLIANHLRPGAIDRAELVAIASA

ILVAGTVTTSSAIALGTLALLTAPGQYAALVDNPDLVPGAVNEILRYLSL

VEQLARVATEDIEIGGKLIRAGDGIIVSFAAGNLDPNVTTHPDRLDVALP

PTNHLAFSHGIHHCIGQNLALLELDIAFRALVSRFPTLRLAVPAEQLPTY

FAGDVPRLACLPVTW

>CYP105CP2(2563832159)*Salinispora arenicola* CNY685

MTKSMPVQDLPAFPIPRECPYRPSAQHVSLRSGGPMARVRLYNGRTAWLV

TDSAHARAVLSDYRRVSIKPYHGNYPLLNEEFEKVVDSGYADVLFGVDPP

EHTRQRQMIMPSFTLRRTAVLRPDIQRIVDDKLDEMTRHGAPGDLVTEFA

QPVPSMVMSFLLGVPWEDHEEFETPAHKLFVPELAEEATTELGAYLERLI

QKKEQPGGTPGGTGLLDDLIRDHLRAGALSRDELVHIAMAMLVAGTDTTT

NVISLGTLALLDNPDQWEALRDNPDELIPGAVEEILRYTSLIEAFARVAV

SDIELNGAVIKEGEGILISSAGVNFDPALAPDPGRFDIRRPPRPSFSFSH

GIHRCPGDNLARLELEIAFRSLVTRMPNLRTAKPIDQIPSNNNDGTLQRL

YELPVVW

>CYP105CH2(2563832507)*Salinispora arenicola* CNY685

VSSLPLPTYPKLRDPADPLLPPADYLAIQSEKPIAKVLLPSGRPTWLITG

HALARQVLTEPCVSVDRKHPNFPYPVPNPDAVVAQVARWTYILLGDDPPL

HTERRRLLISEFSVRQAQAMRPRIQQLVDFHLDQLIAAGPGADFSKHFAM

KVPSAVICEMLGVPFADHDYFQERTALQLRRDVAVADQKQAIDELLAYFE

QLIQRKSSHPGDDLLSRLIVSNRETEAFDHEALVALGLLLLVGGHETTAN

TLTLATATMLERPEVAEQLRTDPSLMPSAVEEFLRYFSVAVAVSRIATAD

LQVGDQLVRAGESMLLVLNTIARDGTVFPEPHRLDIHRNARNHLAFSHGI

HQCMGQNLARVEMQIALDTVLRRLPGLHLVTPFEELPFKYRHLVWGIEEL

KVAW

>CYP107Q4(2563832868)*Salinispora arenicola* CNY685

MTTTAETSAETIDLFSPEVVADPFGWYARLREETGPTTGTLNIGTMMGGP

EMWLVTRYEDVRQVLTDPRFLTNPPADSPLEDIRAGVFKRLDFPPDLIPW

MANLLNVSDGEDHTRLRKLVSYALTAHRIGKLRPRVEKITADLLDKLAED

GKDGSPVDLVEEYCYPLPVTVICELVGIDEPDRAHWRAWGDSMATMNGER

IPTTLVKCIELARELIEKRRAEPQDDLVTALVQAQAEDQNRVSDDEIIGI

LFSLVTAGHQTTTYLIGNSVILLLENPDQLARLKENPSMWPQAVRELQRL

GPIQFAQPRFPSEDIELGGVTIPRGAPVAPLLLAANTDPRRFPDPNKLII

DRLAVGSESHLGFGKGIHRCLGQHLAYQEAEVALQGLFTRFPDLSLAVPR

EEIPWILRPGFTRTRTLPLKLV

>CYP105G7(2563832869)*Salinispora arenicola* CNY685

LTIETTETPPADDTLRAPLPRQFMQRDDPSKLPPALAALAEQAPVGRSTL

PDGDPFWMVSGYDEARAVLSDPRFSSDRFRYHPRFKKLSGQLGERLRNDK

ARAGSFINMDPPEHTRYRKLLTGQFTVRRMRQLTVRIEQIVTEQLDVMLA

KGNSADLVSAFAVPVPSLMICELLGVRYEDRTEFQRRAAGLLQTDLPIKQ

AVENLEAQRAFMQRLVTDKRRNPADDMISGLVHHAGADPPLTDDELVGIA

TLLLFAGLDTTASMLGLGMFMLLQRPEQMAVLRDDPPRIGDAVEELLRYL

TVVSAGLFRFAKEDVVLGDEHIPAGSTVVVSLMAANRDGRHWPEPETLDV

TRVRSSHLAFGHGVHQCLGQQLARIELTVGITELLRRLPNVRLAVPPADV

PLRNDMITYGVHRLPIRWDTP

>CYP105BL2(2563832960)*Salinispora arenicola* CNY685

MSSHAAAAPDPETATPLHTLAPELTFPQFERSTPFDPPQAYTELSGRCPV

APVSMSDGKPSWLITSFEGVRTTLSDPRFSSDMSHPGFPNRTGKPVDDLL

KDTLGAMDGERHRYYRRMLTGELTVRRAKAMRPVITQITDEALDQLAAAG

PGADLVKHVALVVPSRVAYHLVGIPSSDYELFTGMATTLMESTSSADQFA

ALQNMVSYFDTLVTDREHHDRDDLLGRMVRRYLATGELTRDMLITLVWTT

LAAGQETTAHMIGLGVAALLRHPDQLELLRREPHLLPGAVDELMRYLPMI

QFGIPRVAMDDVKVDGQTVTAGEGVVALPPLANRDPAVFERPDELDVRRN

ARQHLTFGYGPHQCPAHALARLELEVVYGRLLERFPTLRLADSDADLKVR

DEDIMYRVSELAVTW

>CYP107EU2(2563833241)*Salinispora arenicola* CNY685

VTVGQTLPDLVYSPEFTRDPYAVFARLREQAPVCRVTTHRGMSAWMVTRY

ADVRALLADSRLAKDGNRIGELMPRHSKLTGAATGFPPGLTTNMVNSDPP

DHTRLRHLVGREFTGHRVESLRPRIEEIVDDLLDGVAACGDEADLAEILS

RRLPIAVIGELLGVPEADRAGFFRWADTLYGGTASPEALGQAYNAIVDYL

GRLCDAKRDVPADDLLTALVQVSADEDRLSREELVSMALLLLVAGHETTS

KQISNGVLALLLNPEQLKLLKAQPALAAGAVEELLRFEGPSLSASLRFTT

EPVEVAGVVIPEGEFVLLSLASGNRDPQKFPDPDRLDITRSTQGNLAMGH

GIHHCVGAALARLELEIVLSRLVARFPQLQLAVAADDLEWLVNSFFRAPL

HLPVSLGR

>CYP1005A1(2563833337)*Salinispora arenicola* CNY685

VSAVLFRSWTKTAGTRWPDVTRVADQSGTEHLVVTRHALVRQVLTDQATY

RPDNALEAVTPVPVAALRVLAGHRFRLPPTLANNGGVSHPAIRALVADAL

HPTKVAAQRPWLTELVADRVATIRTTLDSGGPVDLYADLTADLPLLVLAR

LVELPDAPVNAVKQFARAALELFWAPLDADRQLALADEVGRFHQVLREFA

DTGGGLAAALRATGHPPDVLVGALFFLLVAGQETTSQFLTLLLHRLTDEP

TIRAALRAGSTSVADVVEEGLRLEPPIVTWRRVAAVDSTLGGTTVAAGTS

VLLWLARAGRDPAVVAAPDEFRPGQRGSRRHLAFGAGAHRCLGDQLARME

AAVVVEQATPLLDGVTVVRPPWYPDNLTFRMPDAFVVRR

>CYP107AY14(2563833425)*Salinispora arenicola* CNY685

MKAELTPIPRSGARLGQEYDQLRKTGDVHQVLLPDASLAWLITNPEVAAR

ALADPRLALNRRNSRGGWSGFALPPALDANLLNLDAPDHTRLRRLVGPAF

SPQRVAGLRPGIRRAAEHLLDTLVATSRPTDLVTGYCNPLSVQVIADLMG

VPDAGRTNLRAWTDTMLTSYPPDRDAIRQAVTELHGYVVDLIDLKQQQPG

DDLLSVLVTIEQDGDRLTRDELTSLAFLILFAGYENTANLIASAVLWLLD

HGGLNEVPIAEAIEGTLRDEPPAPVAIRRFPTEDIIIRGLTIPAGDTVLL

SIAATRGADGNAARLAFGNGPHYCLGAALARVEAEEALTVLARRLPGLTL

AVPPAQVRWRPTFRTHGPAELLVGW

>CYP105W3(2563833538)*Salinispora arenicola* CNY685

MTGYQDPPTGDQPGAPVPSGSTDPGLDAFPLPRRCPFSPPAEYARLRAEH

PVVRLPMLGGDTAWVVSRHADVRQVLGDPRMSADRRRPGFPKFAPTTEGQ

RQASFANFRPPLNWMDPPEHAICRRQIVDEFSARRVRQLRTLVERVVDAH

LDALAAAAPGADLVSTFAYPVPSQVICEVLGVPYGEHGFFERRSTLMFRR

STPADERARCAREIREFLDVVVTDKERRPGDDVLSRLLYRQRSAGGVDHE

AVVSMAFVLLVAGHVTTSNMLALSVLALLTHPARLARLRAEPKRFPAAVE

ELLRYFTVVEAATARTATAEVTIGGVTIAAGEGVVALGQAANRDPRVFEH

PDEFDPDRDARAHLAFGHGRHICPGQHLARLEMEVALSRLFRRLPGLRLT

MKVSDLPLKEDSNIFGLYALPVAW

>CYP248A2(2563833546)*Salinispora arenicola* CNY685

VLADAVTAFDPTAVDVRRDPYPSYHWLLRHDPVHRGAHQVWYVSRFADVR

AVLGDERFARTGIRRFWTDLVGPGLLSQIVGDIILFQDEPDHGRLRGVVG

PAFSPSALRRLEPTIEATVNDLLRPARALGAMDVVADLAYPLALRAVLGL

LGLPAGDADAVGRWSRAVGRTLDRGATAEDMRRGHAAIAEFADYVERALA

ERREDGADLLALMLAAHRSQLMSRNEIVSTVVTFIFTGHETVASQLGNGL

LSLLDHPEQSELVRRQPHLVSQAVEECLRFDPAVQSNTRQLAADVELHGR

RLRRDDVVVVLAGAANRDPGRYDRPDEFDIRRDPVPSMSFGAGMRYCLGS

YLARLQLRTALGAMVALPDLRLVCSPNELAYQPRTMFRGLTRLPVAFTPA

G

>CYP105AB35(2563833710)*Salinispora arenicola* CNY685

MTETASSRLTDTEFPVQRQCPFAEPVEYEQIREQSSIAMVRLTGGGEAWW

ISGHEQGRAVLADRRFSSDRRKANFPFISTDPATRKRLYAQPLSMISMDG

AEHAQARRALIGEFTVRRLAALRPRIQQIVDQCIDEMLTTDQHCVDLVKA

LSLPVPSLVICELLGVPYDDHDFFQEHTATLVRRNTAPEVRQHSIDELNA

YLGALIDRKLANPDDDLLGRQIARQHQDGTFDRSSIVSLAFLLLVAGHET

TANMISLGVVGLLQHPDQLTMIKNDPDKMPLAIEELLRFFTIVDSVTSRV

ATEDVRFGDTTINAGDGVVVSGLSADWDPTVFADPDRLDLERGARHHIAF

GFGPHQCIGQNLARLELQIVFDTLFRRIPTLRLAASLDKIPFKTDAAIYG

ARELPVAW

>CYP107AX1(2563834207)*Salinispora arenicola* CNY685

MTSRPTAVFDQCLLRDPHSRYNALRDQAPVHHVLTPDGAPAWLVTRYNDV

RAAFTDPRLSVDKRFSGTDGEHGSSLPPELDAHLLNRDPPDHTRLRRLAA

AAFTPRRVADLHPAVERIVSTLLDGLAGHDRAELIGSLASPLPLQVMHKL

LGLPTQANIDFRTWTNTLLSADANQPAQSRAAMANMRRFLIEQLAHKRAQ

PGDDLLTGLLAAREDDDRLTDDELVAMVFLLMFAGYDNTAALIGTVTHSL

LTNAELHEAVRGGSLALDELIDEVLRWNPAFPLAVRRFAREPITIAGQTI

PAGDRIWLCLASANRDPAQFTQPDELGIVGLRRPHLSFGHGIHYCLGAPL

ARLQTTIAVTSLLNRFPEMRLAVPAHDIRWRESFRLRGLIALPVYL

>CYP163B23(2563834754)*Salinispora arenicola* CNY685

MTEAWYDLSELDLSDPDTYSRYDQHQIWRQLRSEGGIHRQPGTADKPSFW

VVTKYNDVKAVLGDTERFTSERGNVFATLLAGGDTASGQMLAVTDGQRHK

DIRTILTKAFSPRALNYVAERVRGNASRLVSEAVGKESCDFATEVAERIP

ISTICDLLGVPSGDHDILLGLTKSTLGSDRPGYDELEARLARNEILLYFG

DLVERRRKDPQEDVISVLATAVVDGARLPEDTVVLNCYSLLLGGDETSRL

SMIGAVQAFIDSPTEWRRLKNEEVTLETATEEILRWTSPAMHFGRRAVVK

SVIREHVIEAGDIVTVWLSSANRDEEVFDRADEMDLARSPNKHITFGHGR

HFCIGSYLGRAEVSAMIEALRSQVVNIERVGEPRPLHSNFFSGLRSLPVK

LAAS

>CYP1198B2(2563834806)*Salinispora arenicola* CNY685

MSGELTDQRAAPDVGGNPLRSLLDHGIRANPYPLFGELREAGPTAVEDGS

VVLFGEYEHCSQILRHRDMGSDTSEAPSIKGFVVDDAERAGSSIFFMDQP

GHGRQRKLVSKSFTPRIVQSFGPQITRIVDGLFEDFQDKGELDVVTDLAY

PVSIGIICDLFGIPDDERDMLKEWSDDLALSTELPTLGAAIGVLNVFTRD

EINRFGSVAMAAHAYFADLIHRRRKNPGDDLVSSLLATESNGERLTRFEV

TSVLATLFVAAHESTTNLISGGILALLRNQDQMTVLRENPGVITNVVDES

LRYDPPVHLAARMARARTTIGGYDLDPGSIVVVLMAAGNRDPRAYENPDV

FDVNRKIKNVSLAFGAGAHFCIGSGLAKLEAEIAISAFAQRLKNPEVDES

SLEYRRHIVVRGLEHMKVSFQP

>CYP105BL6(2516634423)*Salinispora pacifica* DSM 45547

MSSHPAAAPGPETATPLHTLAPELTFPQFERATPFDPPEAYTELSGRCPV

APVRMADGKPSWLITSFEGVRAALSDPRLSSDMSHPGFPNRTGKPVDDLL

KDTLGAMDGERHRYYRRMLTGELTVRRAKAMRPVITQITDEALDQLAAAG

PGADLVKHVALVVPSRVACHLVGIPLSEYELFTGMAAKLMEATSSADQIA

ALQDMVSYFDKLVTDREHHDRDDLLGHMVRRYLSTGELTREMLIRLAWTT

MAAGQETTAHMIGLGVAALLRHPDQLELLRREPHLMPGAVDELMRYLPMI

QFGIPRVAMDDVEVDGQTVTAGEGVVALPPLANRDPAVFERPDELDVRRN

ARQHLSFGYGPHQCPAHALARLELEVVYSRLLERFPTLRLADGDADLKVQ

DEDIMYNVSELAVAW

>CYP107AY7(2516636062)*Salinispora pacifica* DSM 45547

MRAEPAPIPRSGARLGQEYDQLRKTGDVHQVLLPDTSLAWLVTSPELVSR

ALADPRLALNRKHSRGGWSGFALPPALDANLLNLDAPDHTRLRRLVGPAF

SPQRVAALRPRIERTAEELVDTVVATGSPVDLVTGYCTPLSVQVIADLLG

VPEARRTDLRAWTDTMLTSYPPDRDAIRQAVVELHGYVVNLIEAKRQRPG

DDLLSALVATEQEGDRLTRDELTSLAFLILFAGYENTANLIASTVLRLLG

HGSLRGARASEAIEETLRLEPPAPAAIRRFPIEEMTIGGATIPAGDTVLL

SIAAATRGTDGNSARLAFGNGPHFCLGAALARVEAEEAITVLARRLPSLA

LAAPGAPVRWRPTFRTHGPAELLVTW

>CYP105AB34(2516637484)*Salinispora pacifica* DSM 45547

MTETASITTPGTSSTATSGPGSGEVTDTEFPVERGCPFSTPAEYEQIREH

SPLTKVRLTTGREAWWIAGHELARAVLADRRFSSDRRRDNFPFVSTDPET

RKQLQDQPTSMLGMDGAEHAQTRRALMGEFTVRRMAGLRPRIQQIVDQHI

DEMLSSEQRSADLVEALSLPVPSLVICELLGVPYADHDFFQARSGPLVRH

HTPSKVRVRIQEELNTYLGGLIDRKVADPTDDLLSRQIAKQHAAGTFDRT

SLVSMAFLLLIAGHETTANMISLGVVGLLQHPDQLAMIKDDPEKTPPAVE

ELLRYFTITDTVTARVATEDVQLGDTTITAGDGVVISGLAADHDPTVFTD

PNQIDLERGARHHVAFGFGPHQCIGQTLARLELQIVFDTLFRRIPTLRLA

APLDDIPFKSDAIIYGAEELPVAW

>CYP1005A8(2516637726)*Salinispora pacifica* DSM 45547

MSAVLFRSWTKTAGPHWPAVTRVADQQGTEHLVVTEHELVRQVLTDQVTY

RPDNALDAVTPIPVPALRVLAGHGFRLPPTLANNGGVSHPAIRALVADAL

HPAKVAAQRPWLTKLVAERVAAIGATLDSGGSADLHAELNADLPLLVLAR

LVELPDAPVSAVKQFARAALELFWAPLDADRQLALADEVGRFHQVLREFA

DTGGGLAAALRTTGHPPDVLVGALFFLLVAGQETTSQFLTLLLHRLAGEP

TVRAALRDGGVSVANVVEEGLRLEPPIVTWRRVAAVDSTLGGTAVPAGTS

VLLWLARAGRDPAIVPAPDEFRPGQRGSRRHLAFGAGAHRCLGDQLARME

AAVVVEQAAPLLDGISVVRAPWYPDNLTFRMPDAFVIRR

>CYP211B14(2516638071)*Salinispora pacifica* DSM 45547

MDASEAVALLMSPPGRLDPYPTYERLRAHGPVVPTAAGFFVVTGYTEADA

VLRNPRFGVMDDEERDGVFPHWQDSPAMMSISQSMIRANPPDHSRMRRLA

AGVFTPRRVAALREVVAAQADELIDEMIRAGRGGAPVDFLGSFAYPLPVT

VICALLGVPAADWAQFRRWASDLTGVLEPEITPQELAIADAGATELRDYF

TELIAQRRRDPTDDLTTALVQTHDADGDRLSGEELLANLVLLLVAGFETT

TNLLGNGLFVLLTHPESATALRDQPELAPGYVDELLRYDSPVQLTTRTVR

ESVPLAGVELPAGSWLLVLLGAANRDPARFTDPARFDPGRAQSPPLSFGA

GAHYCLGAGLARLEAQVAFPLLLRRLPELALAGEPIRRNRLTLRGYETLP

VTVGAVAVDHGTPAGAALSTP

> CYP244A5(2516638466)*Salinispora pacifica* DSM 45547

MSATTNAELGEAPETSMPVDPGLFDCMPDLIAAARIAPVVRIPYLGRHAW

VVCDRELVKQALTHPKMGKDITLVPEWMRQPGLMVTAQPPPEYARAMIMS

DGENHARIRRIHAPVLSPRNTERWGEQVATKVEGFLDELSKAAAGSNAEV

DVVTNYTHKIPLAFISEMLGLPPAAEHRLRSITDIMLYSSDYAARREAIG

GLFGAVEEWVRNPDGLRDGVITGLLAGSDGPGAAVTEGEVIVWTLGMIIT

GYETTGSLISTSLYEALRRPPHERPRTDEDITAWIEETLRVHPPFPHPTW

RFPLEDIELGGYLIPKGAPVQVSIAAANRQPGEGADSFDTERRGHGHLSF

GLGMHYCIGAPLVRLEAKIAVRGFLRRFPQARLSADAAVQWESEWMIRRM

SFLPAVLS

>CYP245A11(2516638470)*Salinispora pacifica* DSM 45547

MSSTTLPRFTLTGWNREDIVNPYPVYRRYREVAAVHRGEAGGDAPETFYV

FSYDQVAQVLSSSCFGRGRSLDATAASVPVPADQKALRAVVENWLVFMDP

PRHTELRSLLNRSFSPRIVTGLRPRIARIAQELLSRLGRQVETDLVEGFA

APLPILVIAELLGIPAERHGWLRTNALALQEASSSRARRDTAGYARAEAA

AQEFTEYFREQVRLRRGSAGDDLLTILANAQLRGAPVSLDAVVGTGVHLL

TAGHETTTNSLAKAVLALQAHPAVLEELRGADGLTADSIEEFLRYDPPVQ

AVTRWTHQDTTLGGWEVPRGSRVVALLGSANRDPARFPLPDALDVHRPAD

RQLGFGLGIHYCLGATLARAELEIGLQTLLNGLPTLGYPAQYVDYADDMV

FHGPSRLILVNPGERFCQ

>CYP107AW6(2516638559)*Salinispora pacifica* DSM 45547

METVTGTSAPPPVPYIADPYPTLARIRANGPVSILHSDEGVPMWVIARYR

EVRAALADPRFGQDARRAQALADNRVAGVTLGGDIVHMLNSDPPDHTRLR

RHVQGAFTARRVAAMRPLVERITTSLLDGLAGRKTVDLVQDLAFPLPMLV

ICELLGFPAEERNAYRSWSTAILTHDDDPAVFATALREMTDYIAVQLRIR

RSRPGDDLLTELLAARDAGQLTDDEIVGMVFLLLIGGHETTVNLLGTATL

ALLRNPDQHRWLLANRHALPEAIDEFLRYESPVAMATLRFTTTPVTVDDV

VIPAGELVLVSLGGANRDPDRFPDADRLILDRRDTGHLAFGHGLHRCLGA

FLGKLEGEVALGALLRRHPKLALATEVRQLQWRDTIMLRGLESLPVSLHG

>CYP107AX9(2516638575)*Salinispora pacifica* DSM 45547

MTAQPAPVFDQRLLRDPHRRYNALRDQAPVHRVRTPDGAPAWLVTRYDDV

RAAFTDPRLSVDKRFSGTDGEHGSSLPPELDAHLLNRDPPDHTRLRRLAA

AAFTPRRVADLRPAVEKTVSTLLDGLAGNDHAELIGSLASPLPLQVMHEL

LGLPTQTSVDFRTWTNTLLSADANQPAQSRSAMANMRRFLIEQVAHKRAQ

PGDDLLTGLLCVREDDDGLTDDELVAMLFLLMFAGYDNTAALIGNAIHAL

LTNVELAEAVRTGSLAVDELVDGVLRWNPSFPLAVRRFAREPITIAGQTI

PAGDRIWLCLASANRDPAHFTEPDEIGIADMRRPHLSFGHGIHYCLGAPL

ARLQTTVAVASLFDRFPGIRLAVPVQDIQWRESFRLRGLVALPVSL

>CYP208A21(2516638588)*Salinispora pacifica* DSM 45547

MTTTSIDRRRPPGPPRAAALSMLLTMSRDRLGMMTAAARAYGDAAWLPVG

HKALYFFNHPDYAKHVLTDNSDNYTKGIGLVHARRALGDGLLTSEGELWR

EQRRVIRPSFRSGRAPDQASVIAEEVASLVERLRARAGGPPVNVVTEFTG

LTLGVLGRTLLDVDLTALATVGDAFAAVQDQAMFELVTLSAVPTWIPLTR

QRRFRRARAELERIVDDLVARRGDVSGRDDVLSRLILSTGAEPDARVRRQ

RLRDELVTLLLAGHETTASTLGWTLYLIDRHPPVRERLRAEAAEVLGDRL

PAYRDLPDLRFTTMVVQEAMRLYPPVWLLPRRSRRADRVGPYWVPAGSDV

VVSPYTMHRHPGFWPEPDRFDPLRFDPRNAADRPRYAYLPFGAGPRVCVG

SNLGMTEAVIAVAMLCRELRLVRVPTHAAVPEPMLSLRIRGGLPMSVHLA

D

>CYP154M16(2516638605)*Salinispora pacifica* DSM 45547

MPDRCPVLDPSGRDIHAEADRLRAQAPAVKVELPGGVHAWSITSYDVVRR

LLLDRNVTKNARNHWPKFINDEIPPDWEMISWVAMDNMVTAYGKHLVRLR

RLIAKAFTAQRVETVRPQVEKLVDELLDGLAAETGEVVDLREKFCYPLPA

LLIADLIGMTEQQRAQTAKAMDLMVDTTVSPEQAQAILTGWRTAMDELIA

AKRREPGKDIASDLIAARDDENGGQLTDSELTDTIFAILGAGSETTINFL

DNAVTALVTHPGQLELVRSGRAGWDDVIDEVLRVQCPLASLPLRYAVTDI

ELDGVTIPQGDPILINYAAAGRDPALHGDTAGEFDVTRENKEHVSFGHGP

HYCLGAGIARLVATIGLSRLFERFPDLRLAVPAEELQPLPTFIMNGHRAL

PVRLVPAPAAATAV

>CYP211C6(2516638846)*Salinispora pacifica* DSM 45547

MPDIEGLLARLYSAQGRQDPYPVYADLHAKAAIAALEPRPERQRVAAVAV

GYDLVAAVLRDPEWFKQPPPGWRDQEILRILQSSMMFINPPDHGRMRHVF

AGTFTPRRLGALEPVINRVTDELLDRMADAGPGEVDFVAEFAYPLPARVM

AEFIGIPATELAWYRERVDRVDAFLDVAGKTPERLAAANAAGAELRFFYR

ELLAHRRRTPGEDLISGLVEAVDAGGVELTEDELISNLIVLFNASFVTTV

YLLSNGLPVLLAHPEVAAALTSSPELAAGAVDEILRLQTPVHLLARAAPR

DTVLGGVSIPQGQNVLLLIAAANRDPAHFPDPDRFDPRRSGPPSLAFGLG

LHYCLGAAVSRLEGRLALPRLLSRFPRLRILEQPVYSGSLFLRGIDKLSV

SPGGREHP

>CYP125A66(2516638982)*Salinispora pacifica* DSM 45547

MTEPRIPVGFDFTDPAVLERRVPREEFAELRRTAPVWWNAQPKGSAGFDD

DGYWVVTRYADVMAVSRDSETYSTRENTAIARFQPGTTQADREMQRVIMI

NVDPPEHTKLRAIVSRGFTPRAINALRGSLGERAERIVRDAAGRGAGDFV

TDVACELPLQAIAELIGVPQHHRRKVFDWSNQLIGYDDPAYGVDPLAASA

ELLAYAMEMAHERQRNPSDDLVTKLVNAQIDGEHLTADEFGFFVMLLAVA

GNETTRNAITHGMLAFLENPEQWELFKAERPRSAVEEIIRWATPVNVFQR

TALVDTTLAGQAISAGQRVALFYGSANFDESVFEEPERFDITRSPNPHLG

FGGSGVHFCLGANLARLEIELIFNSIADHMPDIRKVADPQRLRSGWINGI

RELPVQYH

>CYP1278A4(2516639204)*Salinispora pacifica* DSM 45547

MPSPVGAVVHPNPYPYYAAMVAERPFHFDEQLDTWVAASAAAAQAVLAAP

GCRVRPPHEPVPQGITGTPAGDVFGNLVRMTDGEPQHRLKAIVTQTLGAI

DRSAVAATAMQRARQVLNDSVRTPYEQLMFELPAQVVATLCGLDPAAGGE

ATRLVGHFVQCIPATASPEQQQRAAQAAAGLQELLGPKLDDTQHGLLGEL

VRMATHVGWTDRAPLLANGIGFLSQTYDATAALMGNTLLALSQQECELPT

SEMALQRFVREVIRHDAPIQNTRRFTATPIRHGDVEVPAGQAVLVLLAAA

NRDPAANPDPHMFRADRTTPNVFTFSAGAHHCPGETLAVTIVTTVVEQLL

RVGFDPAKLSTRVTYRPSPNARIPVLTE

>CYP105BL2(2562134718)*Salinispora arenicola* CNY230

MSSHSAAAPDPETATPLHTLAPELTFPQFERSTPFDPPQAYTELSGRCPV

APVSMADGKPSWLITSFEGVRTTLSDPRFSSDMSHPGFPNRTGKPVDDLL

KDTLGAMDGERHRYYRRMLTGELTVRRAKAMRPVITQITDEALDQLAAAG

PGADLVKHVAFVVPSRVACHLVGIPLSDYELFTGMAATLMDSTSSDDQFA

ALQNMVSYFDTLVTDREHHDRDDLLGHMVRRYLATGELTRDMLIRLAWTT

MAAGQETTAHMIGLGVAALLRHPDQLELLRREPHLLPGAVDELMRYLPLI

QFGIPRLAMDDVEVDGQTVTAGEGVVALPPLANRDPAVFERPDELDVRRN

ARQHLTFGYGPHQCPAHALARLELEVVYGRLLERFPTLRLADSDADLKVQ

DKDIMYRVSELAVTW

>CYP105CH1(2562134793)*Salinispora arenicola* CNY230

VSSLPLPTYPKLRDPADPLLPPAEYLAIQSEKPIAKVLLPSGRPTWLITG

HALARQVLTEPCVSVDRRHPNFPYPVPNPDAVVAQVARWTYILLGDDPPL

HTERRRLLISEFTVRQAQAMRPRIQQLVDFHLEQLIAAGPGADFSKHFAM

KVPSAVICEMLGVPFADHDYFQERTALQLRRDVPVAAQKQAIDELLAYFE

QLIQEKSSHPGDDVLSRLIVSNRETEAFDHEALVALGLLLLVGGHETTAN

TLTLATATMLERPEIAEQLRTDPSLMPSAVEEFLRYFSVAVAVSRIATAD

LQVGGQLVRAGESMLLVLNTIARDGTVFPEPHRLDIRRNARNHLAFSHGI

HQCMGQNLARVEMQIALDTVLRRLPGLHLVAPFEELPFKYRHLVWGIEEL

RVAW

>CYP125A41(2562134950)*Salinispora arenicola* CNY230

MTEPRIPAGFDFTDPEVLAHRVPREEFAELRRTAPVWWNAQPRGSAGFDD

DGYWVVTRYADVMTVSRDSDTYSTRENTAIARLRPDTTREDIEMQRVIML

NVDPPEHTKLRAIVSRGFTPRAINALRGSLAERAEHIVRDAAVRGVGDFV

TDVACELPLQAIAELIGVPQHHRRKVFDWSNQLIGYDDPAYGTDPLTASA

ELLAYAMEMAEERQRSPSDDLVTKLVNAQIDGEHLTTDEFGFFVMLLAVA

GNETTRNAITHGMVAFLDNPEQWELFKAERPKSAVEEIIRWATPVNVFQR

TALVDTVLGGQAISAGQRVALFYGSANFDEAVFEDPERFDITRSPNPHLG

FGGSGAHFCLGANLARLEIELIFNSIADHLPDIRKVAAPQRLRSGWINGI

RQMPVRYR

>CYP211C1(2562135099)*Salinispora arenicola* CNY230

VVDVEELLTRLYSAQGRQDPFPVYADLHAQGPIAALPPEPERRRVAAVAV

GYDLVGAVLRDPEWSKAPPPGWTEQEILRTLQTSMMFINPPDHGRMRHVF

AGTFTPRRLGALEPVVNRVADELLDRMADAGAGGLDFVAEFAYPLPARVM

AEFIGIPETELDWYRERVDVIDAFLDVAGKTPQRLAAANAAGAELRAFYG

ELLARRRRTPGEDLISGLVEAVDAGGVELTEDELVSNLIVLFNASFVTTV

YMLSNGLPVLLAHPEVAAALATDPVLTAGAVDEILRLQAPVHLLARAAPR

DTVLGGVPIPQGQNVLLLVAAANRDPAHFPDPDRFDPWRSGPPSLAFGLG

LHYCLGAAVSRLEGRLALPRLLSRFPRLRIMEQPVYSGSLFLRGIDKLSV

SPGEGSTRE

>CYP105AB8(2562136149)*Salinispora arenicola* CNY230

MTETASSRLTDTEFPVQRECPFAEPVEYEQIREQSSIAMVRLTGGGEAWW

ISGHEQGRAVLADRRFSSDRRKANFPFVSTDPAIRKRLHAQPLSLISMDG

AEHTQARRALIGEFTVRRLAALRPRIQQIVDQCIDEMLTTDQRRADLVKT

LSLPVPSLVICELLGVPYADHDFFQEHTATLVRRNTASEVRQHSIDELNA

YLGALIDRKLASPDDDLLGRQIARQHRDGTFDRSSMVSLAFLLLVAGHET

TANMISLGVVGLLQHPEQLAMIKDDPDKTPLAIEELLRFFTIVDSVTSRV

ATEDVRFGDTTINAGDGVVVSGLSADWDPTVFADPDRLDLERGARHHLAF

GFGPHQCLGQNLARLELQIVFDTLFHRIPTLRLAAPLDKIPFKTDAAIYG

ARELPVAW

>CYP1005A1(2562136417)*Salinispora arenicola* CNY230

VSAVLFRSWTKTAGTRWPDVTRVADQSGTEHLVVTRHALVRQVLTDQATY

RPDNALEAVTPIPVAALRVLAGHRFRLPPTLANNGGVSHPAIRALVADAL

HPTKVAAQRPWLTGLVADRVASIRTTLDSGGPVDLYADLTADLPLLVLAR

LVELPDAPVNAVKQFARAALELFWAPLDADRQLALADEVGRFHQVLREFA

DTGGGLAAALRATGHSPDVLVGALFFLLVAGQETTSQFLTLLLHRLSGEP

TIRAALRAGSSSVADVVEEGLRLEPPIVTWRRVAAVDSTLGGTTVAAGTS

VLLWLARAGRDPAVVAAPDEFRPGQRGSRRHLAFGAGAHRCLGDQLARME

AAVVVEQATPLLDGVTVVRPPWYPDNLTFRMPDAFVVRR

>CYP245A7(2562136635)*Salinispora arenicola* CNY230

MPSATLPRFALTGWSRENIVNPYPVYQRYREVASVHRGEPGGDAPDTFYV

FSYDEVVQVLSSNCFGRGRSLDAAKASVPVPAEQKALRAIVENWLVFMDP

PRHTDLRSLLNRSFSPRIVTELRPRIARIAQELLSRLGQQVDVDLVESFA

APLPILVISELLGIPEERRAWLRANALALQEASSSRAGRDVDGYARAEVA

AQEFTEYFREQVRLRRGRAGGDLITILANAQQRGAPVSLDAIVGTCVHLL

TAGHETTTNSLAKAVLALREHPAVLDELRGAEGLTTDAVEEFLRYDPPVQ

AVTRWAHQDTTLGGCDIPRGSRVVALLGSANRDPARFPSPDVLDVRRPAD

RHLSFGLGIHYCLGATLARAELEIGLQALLDGVPTLGYGTQHVDYADDLV

FHGPSRLVLVNLGERCK

>CYP244A4(2562136639)*Salinispora arenicola* CNY230

MSTTTNTELTEAPETNMPVDPGLFDCMPDLIAAARVAPVVRIPYLGRHAW

VVCDRELVKQALTHPKMGKDIALVPEWMRQPGLMVTAQPDPEYARAMIMS

DGENHARIRRIHAPVLSPRNTERWGERVADKVEGFLDELSQAGSGGSTEV

NVVTNYTHKIPLAFISEMLGLPPEAEHRLRGITDIMLYSSDYAARREAIG

GLFGAVEDWVRNPADLRDGVITGLLAASDGPDAAVTEGEVIVWTLGMIIT

GYETTGSLISTSLYEAIRRPPHERPKTDEDITAWIEETLRVHPPFPHPTW

RFPLEDIELGGYLIPKGAPVQVSIAAANRKPGEGADSFDAERRGHGHLSF

GLGMHYCIGAPLVRLEAQIAVRGFLRRFPQARLSAETAVQWESEWMIRRM

SVLPAVLS

>CYP166A4(2562137334)*Salinispora arenicola* CNY230

MTDAISFELPWARTDKFDPPAVFDALREQRPLARMRYPDGHVGWIVSSYE

LVREVLGDPRFSHSCAVGHFPVTHQGQVIPTHPQIPGMFIHMDPPEHTRY

RRLLTGEFTVRRTSRLTGHVEGVATEQIEVMREHGAPADLVATFARPLVL

RVLSGLVGLPYGERDRYLHAVTLLHDAEADPAEAAAAYEQAGAYFDEVIE

RRRRQPEDDLISTLVGDGELTGEELRNIVTLLLFAGYETTESALAVGMFA

LLHHEDQLARLRADPTKIDAAIEELLRYLTVNQYHTYRTASEDIELHGEV

INKGDSVTVSLPAANRDPARFACPAELDIDRETSGHVAFGFGIHQCLGQN

LARVELRAGLSALLRAFPNLRLAVPADEVPLRLQGSVFAVKNLPVCW

>CYP208A12(2562137442)*Salinispora arenicola* CNY230

MTLDTITPRVPLGPPRTAALRMLLVMKRDRLGMLTSAAARYGDASRLPVG

HKALWFFNHPRYAKHVLADNSANYHKGIGLVHARRALGDGLLTSEGDLWR

KQRKVIQPAFQSRRIAQQAGMIAEEAFALVERLRARAGAGPVELTAELTG

LTLGVLGRSLLDADLAGFDSIGDSFATVQDQAMFELETLNAVPMWIPLPR

QIRFRRARRKLQAVVDTLVDGRAGNLADRVDVLSRLILSARGEADPRVGR

ERLRDELVTLLLAGHETTASTLGWTLSLIDRHPGVWERLHAEAVEVLGDR

LPEYDDLRRLRYTVMVVEEAMRLFPPVWLLPRRALAPDTIGEYRVPANAD

VVISPYTLHRHPEFWPNPERFDPERFAPGQAADRPRYAYLPFGAGPRFCV

GNNLGMMEAVFVIALLCRHLRLTGVPGYRLVPEPMLSLRIRGGLPLVVRP

VS

>CYP285A9(2562137483)*Salinispora arenicola* CNY230

MKLTKPAEHEPVDLEKADLVDPAVHGEGDPHAIWHAMRERDPVHWQQVDE

RLGYWSATRYEDVARVLRDHRVFTSEHGTLLNLLGKKDPASRKQLPVTDP

PRHTKMRSPVQRALNSKVLERHREQIREEAQRLLASVPDGEEFDFAEMVG

KLPMAVTGTLMGLDREDWPQLTYLTSQAIAPDDPEFVLPEGGEAALARAH

RELFAAFEQSLSRRKGKGNGDLIDVLRTMEMDDGRKLRPGEISNSYSLLL

GANVTTPHVPNAAMGELAKDGKYADWASHPELFESGINEALRWSSPASHF

LRYAKTDVELSGVTIRQGEPVAAWIGSANRDATVFPDPYEFDIRRDPRNH

LAFGELPCRATSEAIFSLRPPDGRRRRPAEEEAFTQSRRLASAPFRSNHA

DVKDRSAGDTLSTTSEPVSPPIREGNPR

>CYP107FS2(2562137536)*Salinispora arenicola* CNY230

MPVPQGEQNLTTEVFADPKALFATLGSRQPLHRISLPDGMPAVLVTGNRE

ARQALSDPRLVRSITAAAPELHKYHPLASDDYALSRHMLFADPPDHGRMR

KLVSTAFTRRRVEQMRPRIQQITDDLIDVIAAKGEADLVETLALPLPIAV

ISEMLGVPFADRSEFERHAEVLTGINASSGFDAIIAAGRWFDEYLAELVQ

QRRREPQDDLISGMLAAQDKGDRLTDVELRSNALLLLSAGFETTVNLVAN

GLLALLRHPEAMAALRSEPNLMTTAVDELLRYDSPVSCVTYHFAQEPVEI

GGFEIRSGEHVVIAAAAANHDPTVFADPSRLDLRREGSGQILSFSHGIHF

CLGAPLARLEGEIAFGTVLRRLAGLRLAVPTDSLVWKASFVLHRLERLPV

TFTPDRAPNPIDSVHTV

>CYP105CN1(2562137537)*Salinispora arenicola* CNY230

MAAPAPQATQSTTPHAPSYPLPRECPYRPSAGTARLRDAGPVSTVRLYDG

RTAWLVTGAAEARALLADSRVSNRADFPNYPVMDERHLSMRATREMAREE

EGGFAAALFGMDPPEHTRQRQLLLPRFTVRQVAARRPAIQRIVDEHLDAM

EANGSPADLVSAFATPVPTMVVCTHLGVPYQDRTRFEPAVAGLFEPDRAD

AAMAELTAYLHQLIETKQSEPGDGVIDHLIANHLRPGAIDRAELVAIASA

ILVAGTVTTSSAIALGTLALLTAPGQYAALVDNPDLVPGAVNEILRYLSL

VEQLARVATEDIEIGGKLIRAGDGIIVSFAAGNLDPNVTTHPDRLDVALP

PTNHLAFSHGIHHCIGQNLALLELDIAFRALVSRFPTLRLAVPAEQLPTY

FAGDVPRLACLPVTW

>CYP105CP2(2562137539)*Salinispora arenicola* CNY230

MTKSMPVQDLPAFPIPRECPYRPSAQHVSLRSGGPMAKVRLYNGRTAWLV

TDSAHARAVLSDYRRVSIKPYHGNYPLLNEEFEKVVDSGYADVLFGVDPP

EHTRQRQMIMPSFTLRRTAVLRPDIQRIVDDKLDEMMRHGAPGDLVTEFA

QPVPSMVMSFLLGVPWEDHEEFETPAHKLFVPELAEEATTELGAYLERLI

QKKEQPGGTPGGTGLLDDLIRDHLRAGALSRDELVHIAMAMLVAGTDTTT

NVISLGTLALLDNPDQWAALRDNPDELIPGAVEEILRYTSLIEAFARVAV

SDIELNGAVIKEGEGILISSAGVNFDPALAPDPGRFDIRRPPRPSFSFSH

GIHRCPGDNLARLELEIAFRSLVTRMPNLRTAKPIDQIPSNNNDGTLQRL

YELPVVW

>CYP107Q4(2562137639)*Salinispora arenicola* CNY230

MTTTAETSAETIDLFSPEVVADPFGWYARLREETGPTTGTLNIGTMMGGP

EMWLVTRYEDVRQVLTDPRFLTNPPADSPLEDIRAGVFKRLDFPPDLIPW

MANLLNVSDGEDHTRLRKLVSYALTAHRIGKLRPRVEKITADLLDKLAED

GKDGSPVDLVEEYCYPLPVTVICELVGIDEPDRPHWRAWGDSMATMNGER

IPTTLVKCIELARELIAKRRAEPQDDLVTALVQAQAEDQNRVSDDEIIGI

LFSLVTAGHQTTTYLIGNSVILLLENPDQLARLKENPSMWPQAVRELQRL

GPIQFAQPRFPSEDIELGGVTIPRGAPVAPLLLAANTDPRRFPDPNKLII

DRLAVGSEGHLGFGKGIHRCLGQHLAYQEAEVALQGLFTRFPDLSLAVPR

EEIPWILRPGFTRTRTLPLKLV

>CYP105G5(2562137640)*Salinispora arenicola* CNY230

LTIETTETPPADDSLRAPLPRQFMQRDDPSKLPPALAALAEQSPVGRSTL

PDGDPFWMVSGYDEARAVLSDPRFSSDRFRYHPRFKKLSGQLGERLRNDK

ARAGSFINMDPPEHTRYRKLLTGQFTVRRMRQLTVRIEQIVTEQVDVMLA

EGNSADLVSAFAVPVPSLMICELLGVRYEDRTEFQRRAAGLLQTDLPIKQ

AVENLEAQRAFMQRLVTDKRRTPADDMISGLVHHAGAEPPLTDDELVGIA

TLLLFAGLDTTASMLGLGMFMLLQRPEQMAVLRDDPSRIGDAVEELLRYL

TVVSTGLFRFAKEDVVLGDEHIPAGSTVVVSLMAANRDGRHWPEPETLDV

TRVRSSHLAFGHGVHQCLGQQLARIELTVGITELLRRLPNVRLAVPPADV

PLRNDMITYGVHRLPILWDTP

>CYP1051A1(2562137666)*Salinispora arenicola* CNY230

MATDAAITRARTVPAWKALPAAVRDTHRAFVDVGNWSDGDVVRVSLGVSR

PYLVTNPAHVQEVLHERAAIYPRGDDTALWRSVRKLVGDGILAEGDAWAA

SRRVLAPMFRPARINAMVDTMADAIAGAVDDLHGAGTAGTPIDVGRELSR

IVCSAIMRVFFADRITVQDALRIMKAQETIVTAMAPRILAPLVPWWIPMP

GDRRFRAAVRSIDNILLPVLRQAQRQPDDGDDLLSRLVRARADDGRALSE

KRMRDDLVSMVAVTTETSTVVLTWLWPLLANHPDVANRLYDEIDRVVGGG

PVRGDHLAELTYTRMVLDELLRLYPAGWILPRRAAATDVLGGVRINKGAT

VILSPYVTQRMTAWWGPTAEAFDPERFAAGREAADGRHRYAYYPFGVGMH

RCLGEHLFNLEAILIVATLLSRFRFALTDTSMPGVKVAASTRPARTVEMI

LKPVAPVPAR

>CYP211B2(2562138554)*Salinispora arenicola* CNY230

MDVSEAIAVLISPSGRLDPYPTYEQLRAHGPVSRTTAGLFVVTGYAEADM

VLRDPRFVVLDDDLRDDVFPHWQDSPAIKSIARSMLRTNPPDHSRIRRLA

AGAFTPRRVAAMREVVTAQADELVDEMIRAGRDGARVDFMDMFAYPLPVA

VICALLGVPAADRSRFRRWAGDLTGILEPEITPEELAGADAGADELRDYF

TGLIEQRRRAPADDLTTALVQAHDADGDRLSGEELLANLVVLLVAGFETT

TNLLGNGLVVLLTRPEAAAALRDEPDLAPGYVDELLRYDSPVQLTTRTVR

ESVSFAGTELPADSWLLVLLGAANRDPRRFPDPARFDPGRAQSQPLSFGA

GPHYCLGAGLARLEAQVAFPLLLRRLPELALAGRPSRRTRLTLRGYETLP

ITVGAVTADRGTPAGVAPGTP

>CYP105CT1(2562138893)*Salinispora arenicola* CNY230

MNSPNHMPADRSLTAPTSGCPMALSRGRVGLDVADEISELRDGGRLGRIT

TAFGQEATLITRYDEVRAQMADSVVFNVAGVPSPPALVDGGFDTESVRRR

RTVGNLIMLDPPEHTRLRRMVAAWFTTRRVERLRPRVVEIIDAALDEMER

SGPPVDLVAMFAKTVPITVICELIGVPEELRERYRRRAERAVSASAVSTP

LDELRRLREAGWVSRELIEYHRENPSDDIIGMLLREHGTDSHDDGITDDE

LVGLANALLIAGHETTTQMLSMGTLALLRHPDQLALLRDDPSIVAGAVEE

LLRYVGVLHGGFVRVATRDTRLGGHRIHAGELVVPALTAANRDPRLLTDG

DRLDITRPPTSHVAFGHGVHFCIGAPLARMELREAFPALLRRFPGLRLAV

PDSELEFTQGTTVYSLRGLPVTW

>CYP154M5(2562138910)*Salinispora arenicola* CNY230

VEQSCPYKLDVTGRDVHAEGEAIRARGPVAQVELPGGVQGWSVTGYQAAR

QVLADPRFAKDPKKWPAYTSGAIPPNWPLIGWLLMDNMTTNDGADHQRLR

KLVSHGFTPRQVERTRPLIVKIVNDLLDGLSSAGPDEVVDLKGRFATPLP

ARVICDMFGVPEALRASVLRGAQVNVTSSISGEEAEANVEQWHRELLELV

EAKREKPDEDMASLLIAAKEEDGSTLTQEEVVGTLHLMLGAGSETLMNAL

SYAVLGMLSNPGQYEMVRNGTSSWDDVIEETLRAQAPVAQLPLRYATEDV

AVGGAVIKAGDPVLMGFTAIGRDPAVHGETAGDYDITREDKTHLSFGHGV

HFCLGAPLARLELKIALPALFERFPNMTLAVRPDQLEPQGTFIMNGHREL

PVRLGQPATVLA

>CYP105J3(2562139009)*Salinispora arenicola* CNY230

MTDSVAFPQGRVCPHQPAPGYRPLAVQRPLAQVTLYDGRRVWAVTTRDLA

RRLLVDPRISSDRTNPAWPAIVPIVAAAVNDAQQKVLKIATALVGTDGPE

HKAQRKMLIPSFTFRRMNALRPMIQEIVDQQLDEMIKSGAPTDLIPAFAS

AVPVTVLYRLMGIPDDDHGIFEKLSHQLLAGPNANEAYDQLMGYMSRLIA

ERRRNPGEGVLDDLLAQHGANDDADHDELVSTLVVQVAGNHGTTGSMIAL

GLFALLQHPEQLAELRADPSLMPTAVDELLRFLSVPDAVTRLAADDIEVE

GTIIRKGDGVFFITSLINRDTDVHDAPNSLGWHHASAADHLTFGFGAHQC

LGQSLARITMEIALGALIDRLPSLRLAVPAEEVPFLPAASLQVIAELPIT

W

>CYP1902A1(2562139172)*Salinispora arenicola* CNY230

MVAIGRITYNPVGPAFFIDPYPHYAQLRAENPVHHTKYGFVVLTQRKQIV

EAYRHPALSRNTMLWEDFATWRRGSTDGPLERMMANWLVMIDPPRHTPLR

AIHEQVFTRRLLDAATSVVESIVSELLAPGRAAGGMDLVGEFADRIPVYL

INHLLDLPRADWDRIVEWSRAIALTNEPMLTSKVLRAGEDARTAMGEYFD

PLIACRRATPGDDVLSGLAVTEVDGVRLTSEELQDSLAFLYQAGHPTSTH

LISLAVHSLLRHPDQLDRLRADPSLIPDAVEELHRYDGPVQMNDRVAVED

VHLFGEHLHRGQLVRLCVASANRDEDHHPQAARLDVTRTVTDQLGYGHGL

HHCVGAHLGRVQAQAALRALLAAAPGLRLTGDRLRFLPSASNRGLVALPV

AF

>CYP107NF1(2562139188)*Salinispora arenicola* CNY230

MPGYSLDSPARLDTDDFVADAHGVFATLREVAPAVPVIINGGIHAWLITR

YDVAKQAFTDDRMAKDVAHWRAFHAGEVPFTGDVAVAARRNILSTDPPDH

TRLRSALAAAFTPRRVEALRGWIGRIVADLLNSIASAGSADLVADFALPI

PMTVICELFGVPEEHRPHIRSWTETLFHGAEPDRMRQASDDIDSLLAEVI

ASRRARPGDDFTSTLVRAQDEGQLSTVELVMLLRAMLAGGNETTINLLGN

AAAALLRWPDQRATLTADPQRWPDAIEEVLRRDGPIQNSIWRFATTDIKI

GEVTIPCGDAIIIALAAAGRDGRRFADPDRFDIGRTDQAHLAFGRGIHHC

IGAPLARLEAAVALPALFARLPDLRLATSWEQLRYRRSTMSRSLVSLPVK

FTPASPDHPAHP

>CYP1223D1(2562139189)*Salinispora arenicola* CNY230

MSTVVSAMQTLRSEAGRADPFPIYAELHKLGAVCAVDDPAERYQFVVHGY

DAVNHVLRDPAYRVTDAEMLERGGLRWREHPSLVAVLTSMFFTNEPRHAR

LRGMYSRIVTSRRVNALRGQIVAIVDDLLARMAERGAGGEPLDFMAEFAF

PMPGNVICEMMGVPDGDRSWFIPRAHIFGDLLDLGKSSAELLRTADEATV

ELTEYFGKLIALRQKDPRDDMISGFAQLQATAERIPEPELLASLLTFFNA

GFASTSHLLGNGVPLLAGQPRTIEAIQRDDVAARHVEEILRVAPPTHMAV

RVTGEDRTVAGLTMPAGSLLLVLLAAANHDPARFPDPGRFDPNRPDNRPL

TFGAGAHFCLGAPLTRLEGQVAFPMLFDRFPKLAPVGEATKTNRLTLSGY

QTLPVALS

>CYP248A2(2562139215)*Salinispora arenicola* CNY230

VLADAVTAFDPTAVDVRRDPYPSYHWLLRHDPVHRGAHQVWYVSRFADVR

AVLGDERFARTGIRRFWTDLVGPGLLSQIVGDIILFQDEPDHGRLRGVVG

PAFSPSALRRLEPTIEATVNDLLRPARALGAMDVVADLAYPLALRAVLEL

LGLPAGDANAVGRWSRAVGRTLDRGATAEDMRRGHAAIAEFADYVERALA

ERREDGADLLALMLAAHRSQLMSRNEIVSTVVTFIFTGHETVASQLGNGL

LSLLDHPEQMELMRRQPHLVPQAVEECLRFDPAVQSNTRQLVADVELHGR

RLRRDDVVVVLAGAANRDPGRYDRPDELDIRRDPVPSMSFGAGMRYCLGS

YLARLQLRTALGAMVALPDLRLVCSPNELAYQPRTMFRGLTRLPVAFTPA

G

>CYP105W2(2562139223)*Salinispora arenicola* CNY230

MTGYQDRPTGDQPGAPVPSGSTDPGIGAFPLPRRCPFSPPAEYARLRAEH

PVVRLPMLGGDTAWVVSRHADVRQVLSDPRMSADRRRPGFPKFAPTTEGQ

RQASFANFRPPLNWLDPPEHAICRRQIVDEFSVRRVRQSRALVERVVDTH

LDALTAAAPGADLVSTFAYPVPSQVICEVLGVPYGEHEFFERRSTLMFRR

STPADERARCAREIRDFLDMVVTDKERRPGDDVLSRLLYRQRRAGGMDHE

AVVSMAFVLLVAGHVTTSNMIALSVLALLTHPARLARLRAEPERFPAAVE

ELLRYFTVVEAATARTTTAEVTIGGVTIAAGEGVVALGQAANRDPRVFEH

PDEFDPDRDARAHLAFGHGRHICPGQHLARLEMEVALSRLFRRLPGLRLT

MEVSDLPLKEDSNIFGLYALPVAW

>CYP107AY2(2562139297)*Salinispora arenicola* CNY230

MTAEPTPIPRSGARLGQEYDQLRKTGDVHQVLLPDASLAWLVTNPEVAAR

ALADPRLALNRRNSRGGWSGFALPPALDANLLNLDAPDHTRLRRLVGPAF

SPQRVAALRPGIRRAAEHLLDTLVATSGPTDLVTGYCNPLSVQVIADLMG

VPEAGRTNLRAWTDTMLTSYPPDRDAIRRAVTELHGYVVDLIDIKQQQPG

DDLLSTLVTIEQDGDRLSRDELTSLAFLILFAGYENTANLIASAVLWLLD

HGGLNVVPISEAIEGTLRHEPPAPVAIRRFPTEDIIIGGVTIPAGDTVLL

SVAAATRGADGKAARLAFGNGPHYCLGAALARVEAEEALTVLARRLPGLT

LAVPPSQVRWRPTFRTHGPAELLVGW

>CYP247A7(2562139379)*Salinispora arenicola* CNY230

VRLTPGAARDIDLDSVNLFDLDLYTSGDPHPIWDVMRAQSPLHHQVLADG

REFWSVTRYDDVCRVLGDYREFTSERGTVVTHLGEDDIAAGKLLTSTDPP

RHTQVRRAIGAKLTARAVASWQDRIRDAIVRFLEPALDGDTFDLAEQALL

LPAIVTGPLLGIPERDWQELVQLTAMVTAPSDPHFQHGSEAATLAISHHE

LVTYVTEWVKQRRSAGGGDGSLLDHLMSVRVGGAPLTDEEIALDGYSILL

GANVTTPHTVSGTVLALIERPEQFEKAQADPSLLANLVEEGLRWTSAACN

FMRYALNDTRIGGGTVPAGGAVVAWIGSANRDESYFPDPHQFDITRSGAN

RQVAFGFGPHYCIGAPLARMTLGIFFEELVQRFGSIELAGEPQHLRSYFI

AGMTHLPIVAQKRKTP

>CYP107EU1(2562139582)*Salinispora arenicola* CNY230

VTIGQTLPDLVYSPEFTRDPYAIFARLREQAPVCRVTTHRGMSAWMVTRH

ADVRALLADNRLAKDGNRIGELMPRHSTLTGAATGFPPGLTTNMVNSDPP

DHTRLRHLVGREFTGHRVEGLRPRIEEIVDDLLDGVAACGDEADLAETLA

RRLPIAVIGELLGVPEADRAEFFRWADTLYGGTASPEALGQAYNAIVDYL

GRLCDAKRDVPADDLLTALVQVSADEDRLSREELVSMALLLLVAGHETTS

KQISNGVLALLLNPEQLKLLKAQPARTAGAVEELLRFEGPSLSASLRFTT

EPVEVAGVVIPEGEFVLLSLASGNRDPEKFPDPDRLDITRSTQGNLAMGH

GIHHCVGAALARLELEIVLSRLVARFPQMQLAVEADDLEWLVNSFFRAPL

HLPVSLRR

>CYP1198B1(2562139836)*Salinispora arenicola* CNY230

MSGELTDQRTAPGAGGNPLRSLMDHGIRANPYPLFGELREAGPTAVEDGS

VVLFGEYEHCSQILRHRDMGSDTSEAPSIKGFVVDDAERAGSSIFFMDQP

GHGRQRKLVSKSFTPRIVKSFGPQITHIVDGLFEDFRDRGELDVVTDLAY

PVSIGIICDLFGIPDDERDMLKEWSDDLALSTELPTLGAAIGVLNVFTRD

EINRFGSVAMAAHAYFADLIHRRRKNPGDDLVSSLLATESNGERLTRFEV

TSVLATLFVAAHESTTNLISGGILALLRNQDQMAVLRENPGLITNVVDES

LRYDPPVHLAARMARARTTIGGYDLDPGTIVVVLMAAGNRDPRAYENPDV

FDVNRKIRNVSLAFGAGAHFCIGSGLAKLEAEIAISAFAQRLKHPEVDES

SLEYRRHIVVRGLEHMKVSFQP

>CYP105BL2(2561692682)*Salinispora arenicola* CNQ884

MSSHSAAAPDPETATPLHTLAPELTFPQFERSTPFDPPQAYTELSGRCPV

APVSMADGKPSWLITSFEGVRTTLSDPRFSSDMSHPGFPNRTGKPVDDLL

KDTLGAMDGERHRYYRRMLTGELTVRRAKAMRPVITQITDEALDQLAAAG

PGADLVKHVAFVVPSRVACHLVGIPLSDYELFTGMAATLMDSTSSDDQFA

ALQNMVSYFDTLVTDREHHDRDDLLGHMVRRYLATGELTRDMLIRLAWTT

MAAGQETTAHMIGLGVAALLRHPDQLELLRREPHLLPGAVDELMRYLPLI

QFGIPRVAMDDVEVDGQTVTAGEGVVALPPLANRDPAVFERPDELDVRRN

ARQHLTFGYGPHQCPAHALARLELEVVYGRLLERFPTLRLADSDADLKVQ

DKDIMYRVSELAVTW

>CYP105CT1(2561693279)*Salinispora arenicola* CNQ884

MNSPNHMPADRSLTAPTSGCPMALSRGRVGLDVADEISELRDGGRLGRIT

TAFGQEATLITRYDEVRAQMADSVVFNVAGVPSPPALVDGGFDTESVRRR

RTVGNLIMLDPPEHTRLRRMVAAWFTTRRVERLRPRVVEIIDAALDEMER

SGPPVDLVAMFAKTVPITVICELIGVPEELRERYRRRAERAVSASAVSTP

LDELRRLREAGWVSRELIEYHRENPSDDIIGMLLREHGTDSHDDGITDDE

LVGLANALLIAGHETTTQMLSMGTLALLRHPDQLALLRDDPSIVAGAVEE

LLRYVGVLHGGFVRVATRDTRLGGHRIHAGELVVPALTAANRDPRLLTDG

DRLDITRPPTSHVAFGHGVHFCIGAPLARMELREAFPALLRRFPGLRLAV

PDSELEFTQGTTVYSLRGLPVTW

>CYP166A4(2561693433)*Salinispora arenicola* CNQ884

MTDAISFELPWARTDKFDPPAVFDALREQRPLARMRYPDGHVGWIVSSYE

LVREVLGDPRFSHSCAVGHFPVTHQGQVIPTHPQIPGMFIHMDPPEHTRY

RRLLTGEFTVRRTSRLTGHVEGVATEQIEVMREHGAPADLVATFARPLVL

RVLSGLVGLPYGERDRYLHAVTLLHDAEADPAEAAAAYEQAGAYFDEVIE

RRRRQPEDDLISTLVGDGELTGEELRNIVTLLLFAGYETTESALAVGMFA

LLHHEDQLARLRADPTKIDAAIEELLRYLTVNQYHTYRTASEDIELHGEV

INKGDSVTVSLPAANRDPARFACPAELDIDRETSGHVAFGFGIHQCLGQN

LARVELRAGLSALLRAFPNLRLAVPADEVPLRLQGSVFAVKNLPVCW

>CYP125A41(2561693850)*Salinispora arenicola* CNQ884

MTEPRIPAGFDFTDPEVLAHRVPREEFAELRRTAPVWWNAQPRGSAGFDD

DGYWVVTRYADVMTVSRDSDTYSTRENTAIARLRPDTTREDIEMQRVIML

NVDPPEHTKLRAIVSRGFTPRAINALRGSLAERAEHIVRDAAVRGVGDFV

TDVACELPLQAIAELIGVPQHHRRKVFDWSNQLIGYDDPAYGTDPLTASA

ELLAYAMEMAEERQRSPSDDLVTKLVNAQIDGEHLTTDEFGFFVMLLAVA

GNETTRNAITHGMVAFLDNPEQWELFKAERPKSAVEEIIRWATPVNVFQR

TALVDTVLGGQAISAGQRVALFYGSANFDEAVFEDPERFDITRSPNPHLG

FGGSGAHFCLGANLARLEIELIFNSIADHLPDIRKVAAPQRLRSGWINGI

RQMPVRYR

>CYP211C1(2561693998)*Salinispora arenicola* CNQ884

VVDVEELLTRLYSAQGRQDPFPVYADLHAQGPIAALPPEPERRRVAAVAV

GYDLVGAVLRDPEWSKAPPPGWTEQEILRTLQTSMMFINPPDHGRMRHVF

AGTFTPRRLGALEPVVNRVADELLDRMADAGAGGLDFVAEFAYPLPARVM

AEFIGIPETELDWYRERVDVIDAFLDVAGKTPQRLAAANAAGAELRAFYG

ELLARRRRTPGEDLISGLVEAVDAGGVELTEDELVSNLIVLFNASFVTTV

YMLSNGLPVLLAHPEVAAALATDPVLTAGAVDEILRLQAPVHLLARAAPR

DTVLGGVPIPQGQNVLLLIAAANRDPAHFPDPDRFDPWRSGPPSLAFGLG

LHYCLGAAVSRLEGRLALPRLLSRFPRLRIMEQPVYSGSLFLRGIDKLSV

SPGGREYP

>CYP211B2(2561694498)*Salinispora arenicola* CNQ884

MDVSEAIAVLISPSGRLDPYPTYEQLRAHGPVSRTTAGLFVVTGYAEADM

VLRDPRFVVLDDDLRDDVFPHWQDSPAIKSIARSMLRTNPPDHSRIRRLA

AGAFTPRRVAAMREVVTAQADELVDEMIRAGRDGARVDFMDMFAYPLPVA

VICALLGVPAADRSRFRRWAGDLTGILEPEITPEELAGADAGADELRDYF

TGLIEQRRRAPADDLTTALVQAHDADGDRLSGEELLANLVVLLVAGFETT

TNLLGNGLVVLLTRPEAAAALRDEPDLAPGYVDELLRYDSPVQLTTRTVR

ESVSFAGTELPADSWLLVLLGAANRDPRRFPDPARFDPGRAQSQPLSFGA

GPHYCLGAGLARLEAQVAFPLLLRRLPELALAGRPSRRTRLTLRGYETLP

ITVGAVTADRGTPAGVAPGTP

>CYP208A12(2561694824)*Salinispora arenicola* CNQ884

MTLDTITPRVPLGPPRTAALRMLLVMKRDRLGMLTSAAARYGDASRLPVG

HKALWFFNHPRYAKHVLADNSANYHKGIGLVHARRALGDGLLTSEGDLWR

KQRKVIQPAFQSRRIAQQAGMIAEEAFALVERLRARAGAGPVELTAELTG

LTLGVLGRSLLDADLAGFDSIGDSFATVQDQAMFELETLNAVPMWIPLPR

QIRFRRARRKLQAVVDTLVDGRAGNLADRVDVLSRLILSARGEADPRVGR

ERLRDELVTLLLAGHETTASTLGWTLSLIDRHPGVWERLHAEAVEVLGDR

LPEYDDLRRLRYTVMVVEEAMRLFPPVWLLPRRALAPDTIGEYRVPANAD

VVISPYTLHRHPEFWPNPERFDPERFAPGQAADRPRYAYLPFGAGPRFCV

GNNLGMMEAVFVIALLCRHLRLTGVPGYRLVPEPMLSLRIRGGLPLVVRP

VS

>CYP107FS2(2561694931)*Salinispora arenicola* CNQ884

MPVPQGEQNLTTEVFADPKALFATLGSRQPLHRISLPDGMPAVLVTGNRE

ARQALSDPRLVRSITAAAPELHKYHPLASDDYALSRHMLFADPPDHGRMR

KLVSTAFTRRRVEQMRPRIQQITDDLIDVIAAKGEADLVETLALPLPIAV

ISEMLGVPFADRSEFERHAEVLTGINASSGFDTIIAAGRWFDEYLAELVQ

QRRREPQDDLISGMLAAQDKGDRLTDVELRSNALLLLSAGFETTVNLVAN

GLLALLRHPEAMAALRSEPNLMTTAVDELLRYDSPVSCVTYHFAQEPVEI

GGFEIRSGEHVVIAAAAANHDPTVFADPSRLDLRREGSGQILSFSHGIHF

CLGAPLARLEGEIAFGTVLRRLAGLRLAVPTDSLVWKASFVLHRLERLPV

TFTPDRAPNPIDSVHTV

>CYP105CN1(2561694932)*Salinispora arenicola* CNQ884

MAAPAPQATQSTTPHPPSYPLPRECPYRPSAGTARLRDAGPVSTVRLYDG

RTAWLVTGAAEARALLADSRVSNRADFPNYPVMDERHLSMRATREMAREE

EGGFAAALFGMDPPEHTRQRQLLLPRFTVRQVAARRPAIQRIVDEHLDAM

EANGSPADLVSAFATPVPTMVVCTHLGVPYQDRTRFEPAVAGLFEPDRAD

AAMAELTAYLHQLIETKQSEPGDGVIDHLIANHLRPGAIDRAELVAIASA

ILVAGTVTTSSAIALGTLALLTAPGQYAALVDNPDLVPGAVNEILRYLSL

VEQLARVATEDIEIGGKLIRAGDGIIVSFAAGNLDPNVTTHPDRLDVALP

PTNHLAFSHGIHHCIGQNLALLELDIAFRALVSRFPTLRLAVPAEQLPTY

FAGDVPRLACLPVTW

>CYP105CP2(2561694934)*Salinispora arenicola* CNQ884

MTKSMPVQDLPAFPIPRECPYRPSAQHVSLRSGGPMAKVRLYNGRTAWLV

TDSAHARAVLSDYRRVSIKPYHGNYPLLNEEFEKVVDSGYADVLFGVDPP

EHTRQRQMIMPSFTLRRTAVLRPDIQRIVDDKLDEMMRHGAPGDLVTEFA

QPVPSMVMSFLLGVPWEDHEEFETPAHKLFVPELAEEATTELGAYLERLI

QKKEQPGGTPGGTGLLDDLIRDHLRAGALSRDELVHIAMAMLVAGTDTTT

NVISLGTLALLDNPDQWAALRDNPDELIPGAVEEILRYTSLIEAFARVAV

SDIELNGAVIKEGEGILISSAGVNFDPALAPDPGRFDIRRPPRPSFSFSH

GIHRCPGDNLARLELEIAFRSLVTRMPNLRTAKPIDQIPSNNNDGTLQRL

YELPVVW

>CYP245A7(2561695815)*Salinispora arenicola* CNQ884

MPSATLPRFALTGWSRENIVNPYPVYQRYREVASVHRGEPGGDAPDTFYV

FSYDEVVQVLSSNCFGRGRSLDAAKASVPVPAEQKALRAIVENWLVFMDP

PRHTELRSLLNRSFSPRIVTELRPRIARIAQELLSRLGQQVDVDLVESFA

APLPILVISELLGIPEERRAWLRANALALQEASSSRAGRDVDGYAQAEVA

AQEFTEYFREQVRLRRGRAGGDLITILANAQERGAPVSLDAIVGTCVHLL

TAGHETTTNSLAKAVLALREHPAVLDELRGAEGLTTDAVEEFLRYDPPVQ

AVTRWAHQDTTLGGCDIPRGSRVVALLGSANRDPARFPSPDVLDVRRPAD

RHLSFGLGIHYCLGATLARAELEIGLQALLDGVPTLGYGTQHVDYADDLV

FHGPSRLVLVNLGERCK

>CYP244A4(2561695819)*Salinispora arenicola* CNQ884

MSTTTNTELTEAPETNMPVDPGLFDCMPDLIAAARVAPVVRIPYLGRHAW

VVCDRELVKQALTHPKMGKDIALVPEWMRQPGLMVTAQPDPEYARAMIMS

DGENHARIRRIHAPVLSPRNTERWGERVADKVEGFLDELSQAGSGGSAEV

NVVTNYTHKIPLAFISEMLGLPPEAEHRLRGITDIMLYSSDYAARREAIG

GLFGAVEDWVRNPADLRDGVITGLLAASDGPDAAVTEGEVIVWTLGMIIT

GYETTGSLISTSLYEAIRRPPHERPKTDEDITAWIEETLRVHPPFPHPTW

RFPLEDIELGGYLIPKGAPVQVSIAAANRKPGEGADSFDAERRGHGHLSF

GLGMHYCIGAPLVRLEAQIAVRGFLRRFPQARLSAETAVQWESEWMIRRM

SVLPAVLS

>CYP105AB8(2561696028)*Salinispora arenicola* CNQ884

MTETASSRLTDTEFPVQRECPFAEPVEYEQIREQSSIAMVRLTGGGEAWW

ISGHEQGRAVLADRRFSSDRRKANFPFVSTDPAIRKRLHAQPLSLISMDG

AEHTQARRALIGEFTVRRLAALRPRIQQIVDQCIDEMLTTDQHRADLVKT

LSLPVPSLVICELLGVPYADHDFFQEHTATLVRRNTASEVRQHSIDELNA

YLGALIDRKLASPDDDLLGRQIARQHRDGTFDRSSMVSLAFLLLVAGHET

TANMISLGVVGLLQHPEQLAMIKDDPDKTPLAIEELLRFFTIVDSVTSRV

ATEDVRLGDTTINAGDGVVVSGLSADWDPTVFADPDRLDLERGARHHLAF

GFGPHQCLGQNLARLELQIVFDTLFHRIPTLRLAAPLDKIPFKTDAAIYG

ARELPVAW

>CYP1005A1(2561696065)*Salinispora arenicola* CNQ884

VSAVLFRSWTKTAGTRWPDVTRVADQSGTEHLVVTRHALVRQVLTDQATY

RPDNALEAVTPIPVAALRVLAGHRFRLPPTLANNGGVSHPAIRALVADAL

HPTKVAAQRPWLTGLVADRVASIRTTLDSGGPVDLYADLTADLPLLVLAR

LVELPDAPVNAVKQFARAALELFWAPLDADRQLALADEVGRFHQVLREFA

DTGGGLAAALRATGHSPDVLVGALFFLLVAGQETTSQFLTLLLHRLSGEP

TIRAALRAGSISVADVVEEGLRLEPPIVTWRRVAAVDSTLGGTTVAAGTS

VLLWLARAGRDPAVVAAPDEFRPGQRGSRRHLAFGAGAHRCLGDQLARME

AAVVVEQATPLLDGVTVVRPPWYPDNLTFRMPDAFVVRR

>CYP105CH1(2561696407)*Salinispora arenicola* CNQ884

VSSLPLPTYPKLRDPADPLLPPAEYLAIQSEKPIAKVLLPSGRPTWLITG

HALARQVLTEPCVSVDRRHPNFPYPVPNPDAVVAQVARWTYILLGDDPPL

HTERRRLLISEFTVRQAQAMRPRIQQLVDFHLEQLIAAGPGADFSKHFAM

QVPSAVICEMLGVPFADHDYFQERTALQLRRDVPVAAQKQAIDELLAYFE

QLIQEKSSHPGDDVLSRLIVSNRETEAFDHEALVALGLLLLVGGHETTAN

TLTLATATMLERPEIAEQLRTDPSLMPSAVEEFLRYFSVAVAVSRIATAD

LQVGGQLVRAGESMLLVLNTIARDGTVFPEPHRLDIRRNARNHLAFSHGI

HQCMGQNLARVEMQIALDTVLRRLPGLHLVAPFEELPFKYRHLVWGIEEL

RVAW

>CYP105G5(2561696678)*Salinispora arenicola* CNQ884

LTIETTETPPADDSLRAPLPRQFMQRDDPSKLPPALAALAEQSPVGRSTL

PDGDPFWMVSGYDEARAVLSDPRFSSDRFRYHPRFKKLSGQLGERLRNDK

ARAGSFINMDPPEHTRYRKLLTGQFTVRRMRQLTVRIEQIVTEQVDVMLA

EGNSADLVSAFAVPVPSLMICELLGVRYEDRTEFQRRAAGLLQTDLPIKQ

AVENLEAQRAFMQRLVTDKRRTPADDMISGLVHHAGAEPPLTDDELVGIA

TLLLFAGLDTTASMLGLGMFMLLQRPEQMAVLRDDPSRIGDAVEELLRYL

TVVSTGLFRFAKEDVVLGDEHIPAGSTVVVSLMAANRDGRHWPEPETLDV

TRVRSSHLAFGHGVHQCLGQQLARIELTVGITELLRRLPNVRLAVPPADV

PLRNDMITYGVHRLPILWDTP

>CYP105W2(2561696713)*Salinispora arenicola* CNQ884

MTGYQDRPTGDQPGAPVPSGSTDPGIGAFPLPRRCPFSPPAEYARLRAEH

PVVRLPMLGGDTAWVVSRHADVRQVLSDPRMSADRRRPGFPKFAPTTEGQ

RQASFANFRPPLNWLDPPEHAICRRQIVDEFSVRRVRQSRALVERVVDTH

LDALTAAAPGADLVSTFAYPVPSQVICEVLGVPYGEHEFFERRSTLMFRR

STPADERARCAREIRDFLDVVVTDKERRPGDDVLSRLLYRQRRAGGVDHE

AVVSMAFVLLVAGHVTTSNMLALSVLALLTHPARLARLRAEPERFPAAVE

ELLRYFTVVEAATARTTTAEVTIGGVTIAAGEGVVALGQAANRDPRVFEH

PDEFDPDRDARAHLAFGHGRHICPGQHLARLEMEVALSRLFRRLPGLRLT

MEVSDLPLKEDSNIFGLYALPVAW

>CYP248A2(2561696721)*Salinispora arenicola* CNQ884

VLADAVTAFDPTAVDVRRDPYPSYHWLLRHDPVHRGAHQVWYVSRFADVR

AVLGDERFARTGIRRFWTDLVGPGLLSQIVGDIILFQDEPDHGRLRGVVG

PAFSPSALRRLEPTIEATVNDLLRPARALGAMDVVADLAYPLALRAVLEL

LGLPAGDANAVGRWSRAVGRTLDRGATAEDMRRGHAAIAEFADYVERALA

ERREDGADLLALMLAAHGSQLMSRNEIVSTVVTFIFTGHETVASQLGNGL

LSLLDHPEQLELMRRQPHLVPQAVEECLRFDPAVQSNTRQLAADVELHGR

RLRRDDVVVVLAGAANRDPGRYDRPDELDIRRDPVPSMSFGAGMRYCLGS

YLARLQLRTALGAMVALPDLRLACNPNELAYQPRTMFRGLTRLPVAFTPA

G

>CYP105J3(2561696873)*Salinispora arenicola* CNQ884

MTDSVAFPQGRVCPHQPAPGYRPLAVQRPLAQVTLYDGRRVWAVTTRDLA

RRLLVGPRISSDRTNPAWPAIVPIVAAAVNDAQQKVLKIATALVGTDGPE

HKAQRKMLIPSFTFRRMNALRPMIQEIVDQQLDEMIQSGAPTDLIPAFAS

AVPVTVLYRLMGIPDDDHGIFEKLSHQLLAGPNANEAYDQLMGYMSRLIA

ERRRNPGEGVLDDLLAQHGANDDADHDELVSTLVVQVAGNHGTTGSMIAL

GLFALLQHPEQLAELRADPSLMPTAVDELLRFLSVPDAVTRLAADDIEVE

GTIIRKGDGVFFITSLINRDTDVHDAPNSLGWHHASAADHLTFGFGAHQC

LGQSLARITMEIALGALIDRLPSLRLAVPAEEVPFLPAASLQVIAELPIT

W

>CYP107EU1(2561696954)*Salinispora arenicola* CNQ884

VTIGQTLPDLVYSPEFTRDPYAIFARLREQAPVCRVTTHRGMSAWMVTRH

ADVRALLADNRLAKDGNRIGELMPRHSTLTGAATGFPPGLTTNMVNSDPP

DHTRLRHLVGREFTGHRVEGLRPRIEEIVDDLLDGVAACGDEADLAETLA

RRLPIAVIGELLGVPEADRAEFFRWADTLYGGTASPEALGQAYNAIVDYL

GRLCDAKRDVPADDLLTALVQVSADEDRLSREELVSMALLLLVAGHETTS

KQISNGVLALLLNPEQLKLLKAQPARTAGAVEELLRFEGPSLSASLRFTT

EPVEVAGVVIPEGEFVLLSLASGNRDPEKFPDPDRLDITRSTQGNLAMGH

GIHHCVGAALARLELEIVLSRLVARFPQMQLAVEADDLEWLVNSFFRAPL

HLPVSLRR

>CYP1051A1(2561697067)*Salinispora arenicola* CNQ884

MATDAAITRARTVPAWKALPAAVRDTHRAFVDVGNWSDGDVVRVSLGVSR

PYLVTNPAHVQEVLHERAAIYPRGDDTALWRSVRKLVGDGILAEGDAWAA

SRRVLAPMFRPARINAMVDTMADAIAGAVDDLHGAATAGTPIDVGRELSR

IVCSAIMRVFFADRITVRDALRIMKAQETIVTAMAPRILAPLVPWWIPMP

GDRRFRAAVRSIDDILLPVLRQAQRQPDDGDDLLSRLVRARADDGRALSE

KRMRDDLVSMVAVTTETSTVVLTWLWPLLANHPDVANRLYDEIDRVVGGG

PVRGDHLAELTYTRMVLDELLRLYPAGWILPRRAATTDVLGGVRINKGAT

VILSPYVTQRMTAWWGPTAEAFDPERFAAGREAADGRHRYAYYPFGVGMH

RCLGEHLFNLEAILIVATLLSRFRFALTDTSMPGVKVAASTRPARTVEMI

LKPVAPVPAR

>CYP105EJ1(2561697271)*Salinispora arenicola* CNQ884

MAGGNSDLPSFPMARRCPHHPPPEYAEMRARGAAVRVNFLGTPVWAITRH

AEARDLLTDSRVSTDPASPGHPFGALAATAERMAGQLTDMDPPEHSAHRR

MLISEFGVRRVREMRPAIERSVDDLLDGMAAAGAADLVEAYGLPLASAIM

CQLLGVPERDHAFFHDRACAMLGGVFSSRDADVARMEMLAYLDDLVRGHK

PGQDGVIDRLLTIRHDTGEPTHEAIVGMCFMLLLAGHHTTATMIPLGVHT

LLDHPDQLAQLRAEPALWPVAVDELLRFHSIVDWMSFDRVATADIDVGDE

RIHAGEGIFVLGAAANRDERAFERPDDFNIHRRSRHHLAFGVGIHQCLGQ

SLARTELEISYRRLFDRFPTIRIQPPAAGLSFKYDAAMFGLQELPVTW

>CYP1198B1(2561697425)*Salinispora arenicola* CNQ884

MSGELTDQRTAPGAGGNPLRSLMDHGIRANPYPLFGELREAGPTAVEDGS

VVLFGEYEHCSQILRHRDMGSDTSEAPSIKGFVVDDAERAGSSIFFMDQP

GHGRQRKLVSKSFTPRIVKSFGPQITHIVDGLFEDFRDKGELDVVTDLAY

PVSIGIICDLFGIPDDERDMLKEWSDDLALSTELPTLGAAIGVLNVFTRD

EINRFGSVAMAAHAYFADLIHRRRKNPGDDLVSSLLATESNGERLTRFEV

TSVLATLFVAAHESTTNLISGGILALLRNQDQMAVLRENPGLITNVVDES

LRYDPPVHLAARMARARTTIGGYDLDPGTIVVVLMAAGNRDPRAYESPDV

FDVNRKIKNVSLAFGAGAHFCIGSGLAKLEAEIAISAFAQRLKNPEVDES

SLEYRRHIVVRGLEHMKVSFQP

>CYP208A12(2517938111)*Salinispora arenicola* CNY011

MTLDTITPRVPLGPPRTAALRMLLVMKRDRLGMLTSAAARYGDASRLPVG

HKALWFFNHPRYAKHVLADNSANYHKGIGLVHARRALGDGLLTSEGDLWR

KQRKVIQPAFQSRRIAQQAGMIAEEAFALVERLRARAGAGPVELTAELTG

LTLGVLGRSLLDADLAGFDSIGDSFATVQDQAMFELETLNAVPMWIPLPR

QIRFRRARRKLQAVVDTLVDGRAGNLADRVDVLSRLILSARGEADPRVGR

ERLRDELVTLLLAGHETTASTLGWTLSLIDRHPGVWERLHAEAVEVLGDR

LPEYDDLRRLRYTVMVVEEAMRLFPPVWLLPRRALAPDTIGEYRVPANAD

VVISPYTLHRHPEFWPNPERFDPERFAPGQAADRPRYAYLPFGAGPRFCV

GNNLGMMEAVFVIALLCRHLRLTGVPGHRLVPEPMLSLRIRGGLPLVVRP

VS

>CYP245A7(2517939270)*Salinispora arenicola* CNY011

MPSATLPRFALTGWSRENIVNPYPVYQRYREVASVHRGEPGDDAPDTFYV

FSYDEVVQVLSSNCFGRGRSLDAAKASVPVPAEQKALRAIVENWLVFMDP

PRHTELRSLLNRSFSPRIVTELRPRIARIAQELLSRLGQQVDVDLVESFA

APLPILVISELLGIPEERRAWLRANALALQEASSSRAGRDVDGYARAEVA

AQEFTEYFREQVRLRRGRAGGDLITILANAQQRGAPVSLDAIVGTCVHLL

TAGHETTTNSLAKAVLALREHPAVLDELRGAEGLTTDAVEEFLRYDPPVQ

AVTRWAHQDTTLGGCDIPRGSRVVALLGSANRDPARFPSPDVLDVRRPAD

RHLSFGLGIHYCLGATLARAELEIGLQALLDGVPTLGYGTQHVDYADDLV

FHGPSRLVLVNLGERCT

>CYP244A4(2517939266)*Salinispora arenicola* CNY011

MSTTTNTELTEAPETNMPVDPGLFDCMPDLIAAARVAPVVRIPYLGRHAW

VVCDRELVKQALTHPKMGKDIALVPEWMRQPGLMVTAQPDPEYARAMIMS

DGENHARIRRIHAPVLSPRNTERWGERVADKVEGFLDELSRAGSGGSTEV

NVVTNYTHKIPLAFISEMLGLPPEAEHRLRGITDIMLYSSDYAARREAIG

GLFGAVEDWVRNPADLRDGVITGLLAASDGPDAAVTEGEVIVWTLGMIIT

GYETTGSLISTSLYEAIRRPPHERPKTDEDITAWIEETLRVHPPFPHPTW

RFPLEDIELGGYLIPKGAPVQVSIAAANRKPGEGADSFDAERRGHGHLSF

GLGMHYCIGAPLVRLEAQIAVRGFLRRFPQARLSAETAVQWESEWMIRRM

SVLPAVLS

>CYP1051A1(2517939531)*Salinispora arenicola* CNY011

MATDAAITRARTVPAWKALPAAVRDTHRALVDVGNWSDGDVVRVSLGVSR

PYLVTNPAHVQEVLHERAAIYPRGDDTALWRSVRKLVGDGILAEGDAWAA

SRRVLAPMFRPARINAMVDTMADAIAGAVDGLHGAATAGTPIDVGRELSR

IVCSAIMRVFFADRITVRDALRIMKAQETIVTAMAPRILAPLVPWWIPMP

GDRRFRAAVRSIDDILLPVLRQAQRQPDDGDDLLSRLVRARADDGQALSE

KRMRDDLVSMVAVTTETSTVVLTWLWPLLANHPDVANRLYDEIDRVVGGG

PVRGDHLAELTYTRMVLDELLRLYPAGWILPRRAATTDVLGGVRINKGAT

VILSPYVTQRMTAWWGPTAEAFDPERFAAGREAADGRHRYAYYPFGVGMH

RCLGEHLFNLEAILIVATLLSRFRFALTDTSMPGVKVAASTRPARTVEMV

LKPVAPVPAR

>CYP105G5(2517939551)*Salinispora arenicola* CNY011

LTIETTETPPADDSLRAPLPRQFMQRDDPSKLPPALAALAEQSPVGRSTL

PDGDPFWMVSGYDEARAVLSDPRFSSDRFRYHPRFKKLSGQLGERLRNDK

ARAGSFINMDPPEHTRYRKLLTGQFTVRRMRQLTVRIEQIVTEQVDVMLA

EGNSADLVSAFAVPVPSLMICELLGVRYEDRTEFQRRAAGLLQTDLPIKQ

AVENLEAQRAFMQRLVTDKRRTPADDMISGLVHHAGAEPPLTDDELVGIA

TLLLFAGLDTTASMLGLGMFMLLQRPEQMAVLRDDPSRIGDAVEELLRYL

TVVSTGLFRFAKEDVVLGDEHIPAGSTVVVSLMAANRDGRHWPEPETLDV

TRVRSSHLAFGHGVHQCLGQQLARIELTVGITELLRRLPNVRLAVPPADV

PLRNDMITYGVHRLPILWDTP

>CYP107Q4(2517939552)*Salinispora arenicola* CNY011

MTTTAETSAETIDLFSPEVVADPFGWYARLREETGPTTGTLNIGTMMGGP

EMWLVTRYEDVRQVLTDPRFLTNPPADSPLEDIRAGVFKRLDFPPDLIPW

MANLLNVSDGEDHTRLRKLVSYALTAHRIGKLRPRVEKITADLLDKLAED

GKDGSPVDLVEEYCYPLPVTVICELVGIDEPDRPHWRAWGDSMATMNGER

IPTTLVKCIELARELIAKRRAEPQDDLVTALVQAQAEDQNRVSDDEIIGI

LFSLVTAGHQTTTYLIGNSVILLLENPDQLARLKENPSMWPQAVRELQRL

GPIQFAQPRFPSEDIELGGVTIPRGAPVAPLLLAANTDPRRFPDPNKLII

DRLAVGSEGHLGFGKGIHRCLGQHLAYQEAEVALQGLFTRFPDLSLAVPR

EEIPWILRPGFTRTRTLPLKLV

>CYP125A41(2517939652)*Salinispora arenicola* CNY011

MTEPRIPAGFDFTDPEVLAHRVPREEFAELRRTAPVWWNAQPRGSAGFDD

DGYWVVTRYADVMTVSRDSDTYSTRENTAIARLRPDTTREDIEMQRVIML

NVDPPEHTKLRAIVSRGFTPRAINALRGSLAERAEHIVRDAAVRGVGDFV

TDVACELPLQAIAELIGVPQHHRRKVFDWSNQLIGYDDPAYGTDPLTASA

ELLAYAMEMAEERQRSPSDDLVTKLVNAQIDGEHLTTDEFGFFVMLLAVA

GNETTRNAITHGMVAFLDNPEQWELFKAERPKSAVEEIIRWATPVNVFQR

TALVDTVLGGQAISAGQRVALFYGSANFDEAVFEDPERFDITRSPNPHLG

FGGSGAHFCLGANLARLEIELIFNSIADHLPDIRKVAAPQRLRSGWINGI

RQMPVRYR

>CYP166A4(2517940000)*Salinispora arenicola* CNY011

MTDAISFELPWARTDKFDPPAVFDALREQRPLARMRYPDGHVGWIVSSYE

LVREVLGDPRFSHSCAVGHFPVTHQGQVIPTHPQIPGMFIHMDPPEHTRY

RRLLTGEFTVRRTSRLTGHVEGVATEQIEVMREHGAPADLVATFARPLVL

RVLSGLVGLPYGERDRYLHAVTLLHDAEADPAEAAAAYEQAGAYFDEVIE

RRRRQPEDDLISTLVGDGELTGEELRNIVTLLLFAGYETTESALAVGMFA

LLHHEDQLARLRADPTKIDAAIEELLRYLTVNQYHTYRTASEDIELHGEV

INKGDSVTVSLPAANRDPARFACPAELDIDRETSGHVAFGFGIHQCLGQN

LARVELRAGLSALLRAFPNLRLAVPADEVPLRLQGSVFAVKNLPVCW

>CYP154M15(2517940234)*Salinispora arenicola* CNY011

MNDKCPFAALDVTGQDLHGEAARLREQGPAVLVELPGGVKAWSINRYEVI

RELLMDRRVTKNARKHWPAFIKGEIPPDWEMISWVAMDNMVTAYGKDHVR

LRKLVGRAFTQRRADALRPQVVALSTKLLDDLGATPPGEVVDLRERFAYP

LPAMLVASLIGMSEAALAACSKVIDMMVHTNVSPEEAQAVLRGWRAAMAD

LIESKRRTPGEDITTDLIAAREEDGSRLSEAELADTIFAILGAGSETTIN

FFDNAITALLSRPGQLQLLRTGAVTWDDVIDEVLRVESPLAHLPLRYAVE

DIELDGVTIPQGDPILVNYAAAGRDPALHGGTADEFDLARGDKTHLSFGY

GPHYCLGAGIARMVATIGLSMLFERFPDLSLAVPVTELKRLPTFIMNGHQ

TLPVRLTAHAR

>CYP125G6(2517940259)*Salinispora arenicola* CNY011

VPTLLNEHVSYDGAAIAIVDSDGATSWIRLAERVNRWVHLLRAHGLDTGD

RLACVTGNRRETFEVLLAALHTGVTVVPVNWHLTVTEIGHILSDSGSRVV

ITEELHVKAVAAAADGTAGPVAGLVLGDREVEGFAAVEPLLAAASPAEPE

GQVCGATMLYTSGTTGRPKGVVNNLFVTGAPYARVGRLCDYARSVLGVPR

RERMLLDGPWYHSSQLFFALLSLLQGSRLVIRPYFDPAATLKTIDDHRIT

VTHLVPTQLVRLLRVDALTRQMFSGASLRRVWHGGGPCPPEVKRSMIDWW

GPVLVEYYGATEGGVVTLIDSAEWLARPGSVGRAVPPSEVVVVDDGGQPV

AAGQTGQVFFRRRTGNRFHYHNAPEKTQAAYLAPDTFTYGEVGHVDEDGY

LFLTGRAQDMIVSGGVNVYPAEVEAALLRHPVVRDAAVIGVADDEFGERV

VGIVVPETAVDPDDLATHLDAHCRVSLAGFKVPRTYRVVESLPRDETGKL

RKDALRSKFGWLSGAAMTVPRPATGQPTAHRPDIAHPTTYVSGVPHDEFA

RRRRDEPVGWVAEPVLTRHTAAGRTATRGSGFWAVTRYEDVVAASRRVTD

FSSAAKGAFLTDPRTPADLQQARQLLVNMDDPHHARLRKLVTSVFTPRAV

RGLLASIDAHAAALVAKVVAAGEFDVVTDLAAELPLLVLADLLGVPKQDR

ALLYGWSNHLVGFDDPDFGGGDIDAYRTAMAEAFQYALNLGVERRARPTD

DLVSLLANAEVDGTRLTDREFCNFWLLLVVAGNETTRHLIAGTMQALTEH

PGECARLVEGRVPMESAVEELLRWVTPIMQFRRTATRDTEIGGQAVTAGE

KVVLYYTSANRDATVFAEPDRLDLGRTPNRQLSFGIGPHYCLGAHLARAE

L

>CYP105CN1(2517940298)*Salinispora arenicola* CNY011

MAAPAPQATPSTTPHPPSYPLPRECPYRPSAGTAHLRDAGPVSTVRLYDG

RTAWLVTGAAEARALLADSRVSNRADFPNYPVMDERHLSMRATREMAREE

EGGFAAALFGMDPPEHTRQRQLLLPRFTVRQVAARRPAIQRIVDEHLDAM

EANGSPADLVSAFATPVPTMVVCTHLGVPYQDRTRFEPAVAGLFEPDRAD

AAMAELTAYLHQLIETKQSEPGDGVIDHLIANHLRPGAIDRAELVAIASA

ILVAGTVTTSSAIALGTLALLTAPGQYTALVDNPDLVPGAVNEILRYLSL

VEQLARVATEDIEIGGKLIRAGDGIIVSFAAGNLDPNVTTHPDRLDVALP

PTNHLAFSHGIHHCIGQNLALLELDIAFRALVSRFPTLRLAVPAEQLPTY

FAGDVPRLACLPVTW

TALLRVMSPHLGSLQLTGPVSRLASNFVNGVKAMPAVIGSR

>CYP105CP2(2517940300)*Salinispora arenicola*_CNY011

MTKSMPVQDLPAFPIPRECPYRPSAQHVSLRSGGPMAKVRLYNGRTAWLV

TDSAHARAVLSDYRRVSIKPYHGNYPLLNEEFEKVVDSGYADVLFGVDPP

EHTRQRQMIMPSFTLRRTAVLRPDIQRIVDDKLDEMMRHGAPGDLVTEFA

QPVPSMVISFLLGVPWEDHEEFETPAHKLFVPELAEEATTELGAYLERLI

QKKEQPGGTPGGTGLLDDLIRDHLRAGALSRDELVHIAMAMLVAGTDTTT

NVISLGTLALLDNPDQWAALRDNPDELIPGAVEEILRYTSLIEAFARVAV

SDIELNGAVIKEGEGILISSAGVNFDPALAPDPGRFDIRRPPRPSFSFSH

GIHRCPGDNLARLELEIAFRSLVTRMPNLRTAKPIDQIPSNNNDGTLQRL

YELPVVW

>CYP105BL2(2517940822)*Salinispora arenicola* CNY011

MSSHSAAAPDPETATPLHTLAPELAFPQFERSAPFDPPQAYTELSGRCPV

APVSMADGKPSWLITSFEGVRTTLSDPHFSSDMSHPGFPNRTGKPVDDLL

KDTLGAMDGERHRYYRRMLTGELTVRRAKAMRPVITQITDEALDQLAAAG

PGADLVKHVAFVVPSRVACHLVGIPLSDYELFTGMAATLMDSTSSDDQFA

ALQNMVSYFDTLVTDREHHDRDDLLGHMVRRYLATGELTRDMLIRLAWTT

MAAGQETTAHMIGLGVAALLRHPDQLELLRREPHLLPGAVDELMRYLPLI

QFGIPRVAMDDVEVDGQTVTAGEGVVALPPLANRDPAVFERPDELDVRRN

ARQHLTFGYGPHQCPAHALARLELEVVYGRLLERFPTLRLADSDADLKVQ

DKDIMYRVSELAVTW

>CYP105AB8(2517940873)*Salinispora arenicola* CNY011

MTETASSRLTDTEFPVQRECPFAEPVEYEQIREQSSIAMVRLTGGGEAWW

ISGHEQGRAVLADRRFSSDRRKANFPFVSTDPAIRKRLHAQPLSLISMDG

AEHTQARRALIGEFTVRRLAALRPRIQQIVDQCIDEMLTTDQHCADLVKT

LSLPVPSLVICELLGVPYADHDFFQEHTATLVRRNTASEVRQHSIDELNA

YLGALIDRKLASPDDDLLGRQIARQHRDGTFDRSSMVSLAFLLLVAGHET

TANMISLGVVGLLQHPEQLAMIKDDPDKTPLAIEELLRFFTIVDSVTSRV

ATEDVRFGDTTINAGDGVVVSGLSADWDPTVFADPDRLDLERGARHHLAF

GFGPHQCLGQNLARLELQIVFDTLFHRIPTLRLAAPLDKIPFKTDAAIYG

ARELPVAW

>CYP154M21(2517941061)*Salinispora arenicola* CNY011

MEKCPYVLDRAGSDIHKEASNLRARGPVTLVELHGGYTAWSVTSYEVAKQ

LLMDPRISKKTKAHWPEFRDGNVPQDWELYTWVAMDNMQTRDGKEHDRLR

KLVAPAFTGRQAVKSRPIIEEIVNRLLDDLETAPRGQAVDIKARYFYPLS

TILVCDLLGIAEEDRDVILHGNVVNSKTTNTAEESEANLHQWQTALGRLV

ETKRRDPGDDLTTVIIKAGEDEQTPLTDDEVIGSLHLLIGGGTETTANVL

CHTVVDMLTHPDQLAMVRSGAVSWESAWEEEVRKDGAVGSMPFRCATDDV

EIGGVTIAKGDLVLINYAAAGRDPERYGDAAAEFDITRADKANLSFGYGR

HRCLGPALATMEAMVALPALFDRFPNLALAVPPNELKPQGTFIFNGYAEV

PLLLRS

>CYP154M13(2517941062)*Salinispora arenicola* CNY011

MSTGRPVVLDPTGRDIHGEADHLRALGPATLVELPGGVLAWSINSYEVGK

ALLSAPNVSKSARRHWPAFYNGEIPPDWEMISWVAMDNISTTFGGDHRRL

RRLTAKAFGSRRAEQVRPMATHMVNTLLDRMADAADAGEVVDLKAAFAYP

LPGMLVAELIGMSEEARVAAAKVIDMMTATNITPEQAQGVLLGWRDAITD

LIALKRAQPGDDITSDLIAARDEDGSLLTEQELVDTIFAILGAGSETTIN

FFDNAITQLLTHPEQLELVKSGQVSWDDVIEEVLRLESPLASLPMRFAVE

DIQLDGVTIHRGDPILINYAALGRDPALHGESAGVFDVTRQHKEHLSFGH

GAHYCLGAGIARMVAKTGLSALFERFPRMTLAVSATDLVPYPTFIMNGNR

QLPVHLSGALTWREGEQSALGRA

>CYP105W2(2517941075)*Salinispora arenicola* CNY011

MTGYQDRPTGDQPGAPVPSGSTDPGIGAFPLPRRCPFSPPAEYARLRAEH

PVVRLPMLGGDTAWVVSRHADVRQVLSDPRMSADRRRPGFPKFAPTTEGQ

RQASFANFRPPLNWLDPPEHAICRRQIVDEFSVRRVRQSRALVERVVDTH

LDALTAAAPGADLVSTFAYPVPSQVICEVLGVPYGEHEFFERRSTLMFRR

STPADERARCAREIRDFLDVVVTDKEHRPGDDVLSRLLYRQRRAGGVDHE

AVVSMAFVLLVAGHVTTSNMLALSVLALLTHPARLARLRAEPERFPAAVE

ELLRYFTVVEAATARTATAEVTIGGVTIAAGEGVVAVGQAANRDPRVFEH

PDEFDPDRDARAHLAFGHGRHICPGQHLARLEMEVALSRLFRRLPGLRLT

MEVSDLPLKEDSNIFGLYALPVAW

>CYP248A2(2517941083)*Salinispora arenicola* CNY011

VLADAVTAFDPTAVDVRRDPYPSYHWLLRHDPVHRGAHQVWYVSRFADVR

AVLGDERFARTGIRRFWTDLVGPGLLSQIVGDIILFQDEPDHGRLRGVVG

PAFSPSALRRLEPTIEATVNDLLRPARALGAMDVVADLAYPLALRAVLEL

LGLPAGDANAVGRWSRAVGRTLDRGATAEDMRRGHAAIAEFADYVERALA

ERREDGADLLALMLAAHRSQLMSRNEIVSTVVTFIFTGHETVASQLGNGL

LSLLDHPEQLELVRRQPHLVPQAVEECLRFDPAVQSNTRQLAADVELHGR

RLRRDDVVVVLAGAANRDPGRYDRPDELDIRRDPVPSMSFGAGMRYCLGS

YLARLQLRTALGAMVALPDLRLVCNPNELAYQPRTMFRGLTRLPVAFTPA

G

>CYP154M20(2517941106)*Salinispora arenicola* CNY011

MERCPYVLDRAGRDLHGEAKALRARGPVTLVELHGGYTAWSVTSYEIAKQ

LLVDPRISKNTKETWPEFREGKVPQDWELYTWVAMDNMQTRDGEEHDRLR

KLVAQAFTTRQVAKVRPMIEDIVDRLLDDLEKVPAGEVVDIKGRYFYPLS

TILVCDLLGIPEADRAEALHGTVVNARTTNSAEESEANLHQWQSALSKLV

ETKRREPGNDITTLIIKAREDEQAPLTDDEVIGSLHLLIGGGTETTSNVL

CNTLIDLLTHPDQMAMIRSGAVGWEAAWEEEVRKDGAVGSMPFRCANADI

EIGGVTIAKGDLVLINYAAAGRDPERYGDTTAEFDITRADKTNLSFGYGR

HRCLGPALATMEAMISLPALFERFPDLVLAVPRDELKPQGTFVFNGYAEV

PMLLRS

>CYP107FJ2(2517941113)*Salinispora arenicola* CNY011

MSETLSTSVLLADAAEQRAWRAKLRGAGPVHRITTQSGETGWLIVGHEEA

RNALVDLRLQGRTATVGHGRRMPEDLERALNSHMLNVGPPDHTRLRRLVS

AAFTRRRIEQMRPRIQELTDELLDGLAGADEADLVAGLALPLPMRVLVDL

FGIPAEDCADFNVWTKVLTSAGAVDLDRLTTAAGEMVAYLRGLLDRKRQV

PESDLLSALVAVRDGADRLSDDELTSMVYLLLTAGYETTVNLIGNGLLNL

LANPEQLVAFKADPDLLPQVVEEAMRFDSPVQIAVRHSTEPVEIAGQAIP

SGALILVSLLWANRDPDRFTEPEVFRVDRQDNPQLGFGYGFHHCIGAPLA

RMEGTVAIGTVIRRFPALRLAHPAGSLTWRASMVMHGLTALPVHLR

>CYP211C1(2517941320)*Salinispora arenicola* CNY011

VVDVEELLTRLYSAQGRQDPFPVYADLHAQGPIAALPPEPERRRVAAVAV

GYDLVGAVLRDPEWSKAPSPGWTEQEILRTLQTSMMFINPPDHGRMRHVF

AGTFTPRRLGALEPVVNRVADELLDRMADAGAGGLDFVAEFAYPLPARVM

AEFIGIPETELDWYRERVDVIDAFLDVAGKTPQRLAAANAAGAELRAFYG

ELLARRRRTPGEDLITGLVEAVDAGGVELTEDELVSNLIVLFNASFVTTV

YMLSNGLPVLLAHPEVAAALATDPVLTAGAVDEILRLQAPVHLLARAAPR

DTVLGGVPIPQGQNVLLLIAAANRDPAHFPDPDRFDPWRSGPPSLAFGLG

LHYCLGAAVSRLEGRLALPRLLSRFPRLRIMEQPVYSGSLFLRGIDKLSV

SPGEGSTRE

>CYP162P1(2517941387)*Salinispora arenicola* CNY011

MGLPSEVDLADPDLYAEGDPDAEWAWLRAHRPVYRNPAGATAEFWALTRY

RDALQVYRDPSTFSSERGMVLGVDPVAGDPAAGRMLVVTDPPRHPKLRRI

VSGIFVPRTMHRLEGRVRSLVDQLLHRVVDGAGRCDFVNDVAARLPVAII

CELLGVPADEQDWMYHLTSTAFGGGDPAGSAEVSAVERAEAYGDILLYYG

ELAAERRRRPGDDLVTLLVHADLDGEPLDVEDVLVNCTNLIIGGNETTRH

AASGGLLALAQRPELWRRLRETPTAVPTAVEEVLRWTTPGMHVLRTATRD

TEIGGVPIRAGERVVVWNAAANRDEDVFADPQRFDIDRSPNRHIAFGQGG

HHCLGAALARLELTILFEEMAKQVTNVRLTGPVRRVRSCVLRGIRALPVE

LVT

>CYP1005A1(2517941816)*Salinispora arenicola* CNY011

VSAVLFRSWTKTAGTRWPDVTRVADQSGTEHLVVTRHALVRQVLTDQATY

RPDNALEAVTPVPVAALRVLAGHRFRLPPTLANNGGASHPAIRALVADAL

HPTKVAAQRPWLTGLVADRVATIRTTLDSGGPVDLYADLTADLPLLVLAR

LVELPDAPVNAVKQFARAALELFWAPLDADRQLALADEVGRFHQVLREFA

DTGGGLAAALRATGHSPDVLVGALFFLLVAGQETTSQFLTLLLHRLSGEP

TIRAALRAGSSSVADVVEEGLRLEPPIVTWRRVAAVDSTLGGTTVAAGTS

VLLWLARAGRDPAVVAAPDEFRPGQRGSRRHLAFGAGAHRCLGDQLARME

AAVVVEQATPLLDGVTVVRPPWYPDNLTFRMPDAFVVRR

>CYP107EU1(2517941892)*Salinispora arenicola* CNY011

VTIGQTLPDLVYSPEFTRDPYAIFARLREQAPVCRVTTHRGMSAWMVTRH

ADVRALLADNRLAKDGNRIGELMPRHSTLTGAATGFPPGLTTNMVNSDPP

DHTRLRHLVGREFTGHRVEGLRPRIEEIVDDLLDGVAACGDEADLAETLA

RRLPIAVIGELLGVPEADRAEFFRWADTLYGGTASPEALGQAYNAIVDYL

GRLCDAKRDVPADDLLTALVQVSADEDRLSREELVSMALLLLVAGHETTS

KQISNGVLALLLNPEQLKLLKAQPARTAGAVEELLRFEGPSLSASLRFTT

EPVEVAGVVIPEGEFVLLSLASGNRDPEKFPDPDRLDITRYTQGNLAMGH

GIHHCVGAALARLELEIVLSRLVARFPQMQLAVEADDLEWLVNSFFRAPL

HLPVSLRR

>CYP211B2(2517942066)*Salinispora arenicola* CNY011

MDVSEAIAVLISPSGRLDPYPTYEQLRAHGPVSQTTAGLFVVTGYAEADM

VLRDPRFVVLDDDLRDDVFPHWQDSPAIKSIARSMLRTNPPDHSRIRRLA

AGAFTPRRVAAMREVVTAQADELVDEMIRAGRDGARVDFMDMFAYPLPVA

VICALLGVPAADRSRFRRWAGDLTGILEPEITPEELAGADAGADELRDYF

TGLIEQRRRAPADDLTTALVQAHDADGDRLSGEELLANLVVLLVAGFETT

TNLLGNGLVVLLTRPEAAAALRDEPDLAPGYVDELLRYDSPVQLTTRTVR

ESVSFAGTELPAGSWLLVLLGAANRDPRRFPDPARFDPGRAQSQPLSFGA

GPHYCLGAGLARLEAQVAFPLLLRRLPELALAGRPSRRTRLTLRGYETLP

ITVGAVTADRGTPAGVAPGTP

>CYP105CH1(2517942153)*Salinispora arenicola* CNY011

VSSLPLPTYPKLRDPADPLLPPAEYLAIQSEKPIAKVLLPSGRPTWLITG

HALARQVLTEPCVSVDRRHPNFPYPVPNPDAVVAQVARWTYILLGDDPPL

HTERRRLLISEFTVRQAQAMRPRIQQLVDFHLEQLIAAGPGADFSKHFAM

KVPSAVICEMLGVPFADHDYFQERTALQLRRDVPVAAQKQAIDELLAYFE

QLIQEKSSHPGDDVLSRLIVSNRETEAFDHEALVALGLLLLVGGHETTAN

TLTLATATMLERPEIAEQLRTDPSLMPSAVEEFLRYFSVAVAVSRIATAD

LQVGGQLVRAGESMLLVLNTIARDGTVFPEPHRLDIRRNARNHLAFSHGI

HQCMGQNLARVEMQIALDTVLRRLPGLHLVTPFEELPFKYRHLVWGIEEL

RVAW

>CYP107AX14P(2517942162)*Salinispora arenicola* CNY011

VTSPPHVAFGHHLLRDPHGQYNALRGQAPVHHIRTPDGAPAWLVMRYHDV

QAALTDLRLSVDKRFSDTDGEHGSSLPPELDAHLLNRDPPDHTRLRRLAA

AAFTPRRVADQAPAVATIVDALLDGIADQDQAELIGALASPLPLQVMHEL

LGLPTQTSVDFRNWTNTLLSADANQPAQSRSAMATMRRFLMGQLARKRAQ

PGDDLVTGLLAARDDDDRFTDDELLAMVFLLMFAGYDNTAALIGNVTHAL

LTDAGLHDAVRAGSVDLDSVVDEVLRWNPSFPLAVRRFAREPVTPSRQAI

ASGSAWPARTATPHSSPNPTN

>CYP105J3(2517942370)*Salinispora arenicola* CNY011

MTDSVAFPQGRVCPHQPAPGYRPLAVQRPLAQVTLYDGRRVWAVTTRDLA

RRLLVDPRISSDRTNPAWPAIVPIVAAAVNDAQQKVLKIATALVGTDGPE

HKAQRKMLIPSFTFRRMNALRPMIQEIVDQQLDEMIKSGAPTDLIPAFAS

AVPVTVLYRLMGIPDDDHGIFEKLSHQLLAGPNANEAYDQLMGYMSRLIA

ERRRNPGEGVLDDLLAQHGANDDADHDELVSTLVVQVAGNHGTTGSMIAL

GLFALLQHPEQLAELRADPSLMPTAVDELLRFLSVPDAVTRLAADDIEVE

GTVIRKGDGVFFITSLINRDTDVHDAPNSLGWHHASAADHLTFGFGAHQC

LGQSLARITMEIALGALIDRLPSLRLAVPAEEVPFLPAASLQVIAELPIT

W

>CYP107AY2(2517942663)*Salinispora arenicola* CNY011

MTAEPTPIPRSGARLGQEYDQLRKTGDVHQVLLPDASLAWLVTNPEVAAR

ALADPRLALNRRNSRGGWSGFALPPALDANLLNLDAPDHTRLRRLVGPAF

SPQRVAALRPGIRRAAEHLLDTLVATSGPTDLVTGYCNPLSVQVIADLMG

VPEAGRTNLRAWTDTMLTSYPPDRDAIRRAVTELHGYVVDLIDLKQQQPG

DDLLSALVTIEQDGDRLSRDELTSLAFLILFAGYENTANLIASAVLWLLD

HGGLNVVPISEAIEATLRHEPPAPVAIRRFPTEDIIIGGVTIPAGDTVLL

SIAAATRGADGNAARLAFGNGPHYCLGAALARVEAEEALTVLARRLPGLT

LAVPPSQVRWRPTFRTHGPAELLVGW

>CYP244A7(2517942816)*Salinispora arenicola* CNY011

VRVTTSSNLAEAPEVTMPVDLRPTDCLPELLAAARVAPVVRTRYLDQHAW

IVCDRELVKQALTHPKLGKDITLAPDWMRKPGQMVTAMPPPEYARMMITS

DGENHSRIRRLHAPVLSPRNTERWGKRVATLADELLDELDSGDNAEVNVV

TDYTHKVPLAFTSEMLGLPPGAERRLHDITEVMLYSADYALRQQAVEELF

EAVQDWVRNPAGLYDGVITGLLASSDEEDVTVTEGEVIVWTLSLVINGYE

TTGSLISAALYEALRRPVRERPHTDEDIAAWIEEALRVQPPVPHTTWRFA

LEDLSLGGYLIPKGAPVQVSIAAANRDPNEDTDGFDAERRGRGHLSFGRG

VHYCIGAPLARLQAQIALHGFLQRFPRARLSADTAPQWESEWMIRRMSVL

PALLA

>CYP1198B1(2517942937)*Salinispora arenicola* CNY011

MSGELTDQRTAPGAGGNPLRSLMDHGIRANPYPLFGELREAGPTAVEDGS

VVLFGEYEHCSQILRHRDMGSDTSEAPSIKGFVVDDAERAGSSIFFMDQP

GHGRQRKLVSKSFTPRIVKSFGPQITHIVDGLFEDFRDKGELDVVTDLAY

PVSIGIICDLFGIPDDERDMLKEWSDDLALSTELPTLGAAIGVLNVFTRD

EINRFGSVAMAAHAYFADLIHRRRKNPGDDLVSSLLATESNGERLTRFEV

TSVLATLFVAAHESTTNLISGGILALLRNQDQMAVLRENPGLITNVVDES

LRYDPPVHLAARMARARTTIGGYDLDPGTIVVVLMAAGNRDPRAYENPDV

FDVNRKIRNVSLAFGAGAHFCIGSGLAKLEAEIAISAFAQRLKHPEVDES

SLEYRRHIVVRGLEHMKVSFQP

>CYP105BL2(2516011863)*Salinispora arenicola* CNX508

MSSHSAAAPDPETATPLHTLAPELTFPQFERSTPFDPPQAYTELSGRCPV

APVSMADGKPSWLITSFEGVRTTLSDPRFSSDMSHPGFPNRTGKPVDDLL

KDTLGAMDGERHRYYRRMLTGELTVRRAKAMRPVITQITDEALDQLAAAG

PGADLVKHVAFVVPSRVACHLVGIPFSDYERFTGMAATLMDSTSSADQFA

ALQNMVSYFDTLVTDREHHDRDDLLGHMVRRYLASGELTRDMLIRLAWTT

MAAGQETTAHMIGLGVAALLRHPDQLELLRREPHLLPGAVDELMRYLPLI

QFGIPRVAMDDVEVDGQTVTAGEGVVTLPPLANRDPAVFERPDELDVRRN

ARQHLTFGYGPHQCPAHALARLELEVVYGRLLERFPTLRLADSDADLKVQ

DKDIMYRVSELAVTW

>CYP125A41(2516012414)*Salinispora arenicola* CNX508

MTEPRIPAGFDFTDPEVLAHRVPREEFAELRRTAPVWWNAQPRGSAGFDD

DGYWVVTRYADVMTVSRDSDTYSTRENTAIARLRPDTTREDIEMQRVIML

NVDPPEHTKLRAIVSRGFTPRAINALRGSLAERAEHIVRDAAVRGVGDFV

TDVACELPLQAIAELIGVPQHHRRKVFDWSNQLIGYDDPAYGTDPLTASA

ELLAYAMEMAEERQRSPSDDLVTKLVNAQIDGEHLTTDEFGFFVMLLAVA

GNETTRNAITHGMVAFLDNPEQWELFKAERPKSAVEEIIRWATPVNVFQR

TALVDTVLGGQAISAGQRVALFYGSANFDEAVFEDPERFDITRSPNPHLG

FGGSGAHFCLGANLARLEIELIFNSIADHLPDIRKVAAPQRLRSGWINGI

RQMPVRYR

>CYP244A4(2516012801)*Salinispora arenicola* CNX508

MSTTTNTELTEAPETNMPVDPGLFDCMPDLIAAARVAPVVRIPYLGRHAW

VVCDRELVKQALTHPKMGKDIALVPEWMRQPGLMVTAQPDPEYARAMIMS

DGENHARIRRIHAPVLSPRNTERWGDRVADKVEGFLDELSQAGSGGSTEV

NVVTNYTHKIPLAFISEMLGLPPEAEHRLRGITDIMLYSSDYAARREAIG

GLFGAVEDWVRNPADLRDGVITGLLAASDGPDAAVTEGEVIVWTLGMIIT

GYETTGSLISTSLYEAIRRPPHERPKTDEDITAWIEETLRVHPPFPHPTW

RFPLEDIELGGYLIPKGAPVQVSIAAANRKPGEGADSFDAERRGHGHLSF

GLGMHYCIGAPLVRLEAQIAVRGFLRRFPQARLSAETAVQWESEWMIRRM

SVLPAVLS

>CYP245A7(2516012805)*Salinispora arenicola* CNX508

MPSATLPRFALTGWSRENIVNPYPVYQRYREVASVHRGEPGGDAPDTFYV

FSYDEVVQVLSSNCFGRGRSLDAAKASVPVPAEQKALRAIVENWLVFMDP

PRHTELRSLLNRSFSPRIVTELRPRIARIAQELLSRLGQQVDVDLVESFA

APLPILVISELLGIPEERRAWLRANALALQEASSSRAGRDVDGYARADVA

AQEFTEYFREQVRLRRGRAGGDLITILANAQQRGAPVSLDAIVGTCVHLL

TAGHETTTNSLAKAVLALREHPAVLDELRGADGLTTDAVEEFLRYDPPVQ

AVTRWAHQDATLGGCDIPRGSRVVALLGSANRDPARFPSPDVLDVRRPAD

RHLSFGLGIHYCLGATLARAELEIGLQALLDGVPTLGYGTQHVDYADDLV

FHGPSRLVLVNLGERCK

>CYP154M5(2516013607)*Salinispora arenicola* CNX508

VEQSCPYKLDVTGRDVHAEGEAIRARGPVAQVELPGGVQGWSVTGYQAAR

QVLADPRFAKDPKKWPAYTSGAIPPNWPLIGWLLMDNMTTNDGADHQRLR

KLVSHGFTPRQVERTRPLIVKIVNDLLDGLSSAGPDEVVDLKGRFATPLP

ARVICDMFGVPEALRASVLRGAQVNVTSSISGEEAEANVEQWHRELLELV

EAKREKPDEDMASLLIAAKEEDGSTLTQEEVVGTLHLMLGAGSETLMNAL

SYAVLGMLSNPGQYEMVRNGTSSWDDVIEETLRAQAPVAQLPLRYATEDV

AVGGAVIKAGDPVLMGFTAIGRDPAVHGETAGDYDITREDKTHLSFGHGV

HFCLGAPLARLELKIALPALFERFPNMTLAVRPAQLEPQGTFIMNGHREL

PVRLGQPATVLA

>CYP105CT1(2516013624)*Salinispora arenicola* CNX508

MNSPNHMPADRSLTAPTSGCPMALSRGRVGLDVADEIGELRDGGRLGRIT

TAFGQEATLITRYDEVRAQMADSVVFNVAGVPSPPALVDGGFDTESVRRR

RTVGNLIMLDPPEHTRLRRMVAAWFTTRRVERLRPRVVEIIDAALDEMER

SGPPVDLVAMFAKTVPITVICELIGVPEELRERYRRRAERAVSASAVSTP

LDELRRLREAGWVSRELIEYHRENPSDDIIGMLLREHGTDSHDDGITDDE

LVGLANALLIAGHETTTQMLSMGTLALLRHPDQLALLRDDPSIVAGAVEE

LLRYVGVLHGGFVRVATRDTRLGGHRIHAGELVVPALTAANRDPRLLTDG

DRLDITRPPTSHVAFGHGVHFCIGAPLARMELREAFPALLRRFPGLRLAV

PDSELEFTQGTTVYSLRGLPVTW

>CYP1051A1(2516013722)*Salinispora arenicola* CNX508

MATDAAITRARTVPAWKALPAAVRGAHRALVDVGNWSDGDVVRVSLGVSR

PYLVTNPAHVQEVLHERAAIYPRGDDTTLWRSVRKLVGDGILAEGDAWAA

SRRVLAPMFRPARINAMVDTMADAIAGAVDDLHGAATAGTPIDVGRELSR

IVCSAIMRVFFADRITVRDALRIMKAQETIVTAMAPRILAPLVPWWIPMP

GDRRFRAAVRSIDDILLPVLRQAQRQPDDGDDLLSRLVRARADDGRTLSE

KRMRDDLVSMVAVTTETSTVVLTWLWPLLANHPDVANRLYDEIDRVVGGG

PVRGDHLAELTYTRMVLDELLRLYPAGWILPRRAATTDVLGGVRIDKGAT

VILSPYVTQRMTAWWGPTAEAFDPERFAAGREAADGRHRYAYYPFGVGMH

RCLGEHLFNLEAILIVATLLSRFRFALTDTSMPGVKVAASTRPARTVEMV

LKPVAPVPAR

>CYP105G5(2516013755)*Salinispora arenicola* CNX508

LTIETTETPPADDSLRAPLPRQFMQRDDPSKLPPALAALAEQSPVGRSTL

PDGDPFWMVSGYDEARAVLSDPRFSSDRFRYHPRFKKLSGELGERLRNDK

ARAGSFINMDPPEHTRYRKLLTGQFTVRRMRQLTVRIEQIVTEQVDVMLA

EGNSADLVSAFAVPVPSLMICELLGVRYEDRTEFQRRAAGLLQTDLPIKQ

AVENLEAQRAFMQRLVTDKRRTPADDMISGLVHHAGAEPPLTDDELVGIA

TLLLFAGLDTTASMLGLGMFMLLQRPEQMAVLRDDPSRIGDAVEELLRYL

TVVSTGLFRFAKEDVVLGDEHIPAGSTVVVSLMAANRDGRHWPEPETLDV

TRVRSSHLAFGHGVHQCLGQQLARIELTVGITELLRRLPNVRLAVPPADV

PLRNDMITYGVHRLPILWDTP

>CYP107Q4(2516013756)*Salinispora arenicola* CNX508

MTTTAETSAETIDLFSPEVVADPFGWYARLREETGPTTGTLNIGTMMGGP

EMWLVTRYEDVRQVLTDPRFLTNPPADSPLEDIRAGVFKRLDFPPDLIPW

MANLLNVSDGEDHTRLRKLVSYALTAHRIGKLRPRVEKITADLLDKLAED

GKDGSPVDLVEEYCYPLPVTVICELVGIDEPDRPHWRAWGDSMATMNGER

IPTTLVKCIELARELIAKRRAEPQDDLVTALVQAQAEDQNRVSDDEIIGI

LFSLVTAGHQTTTYLIGNSVILLLENPDQLARLKENPSMWPQAVRELQRL

GPIQFAQPRFPSEDIELGGVTIPRGAPVAPLLLAANTDPRRFPDPNKLII

DRLAVGSEGHLGFGKGIHRCLGQHLAYQEAEVALQGLFTRFPDLSLAVPR

EEIPWILRPGFTRTRTLPLKLV

>CYP105CP2(2516014123)*Salinispora arenicola* CNX508

MTKSMPVQDLPAFPIPRECPYRPSAQHVSLRSGGPMAKVRLYNGRTAWLV

TDSAHARAVLSDYRRVSIKPYHGNYPLLNEEFEKVVDSGYADVLFGVDPP

EHTRQRQMIMPSFTLRRTAVLRPDVQRIVDDKLDEMMRHGAPGDLVTEFA

QPVPSMVMSFLLGVPWEDHEEFETPAHKLFVPELAEEATTELGAYLERLI

QKKEQPGGTPGGTGLLDDLIRDHLRAGALSRDELVHIAMAMLVAGTDTTT

NVISLGTLALLDNPDQWAALRDDPDELIPGAVEEILRYTSLIEAFARVAV

SDIELNGAVIKEGEGILISSAGVNFDPALAPDPGRFDIRRPPRPSFSFSH

GIHRCPGDNLARLELEIAFRSLVTRMPNLRTAKPIDQIPSNNNDGTLQRL

YELPVVW

>CYP105CN1(2516014125)*Salinispora arenicola* CNX508

MAAPAPQATQSTTPHPPSYPLPRECPYRPSAGTARLRDAGPVSTVRLYDG

RTAWLVTGAAEARALLADSRVSNRADFPNYPVMDERHLSMRATREMAREE

EGGFAAALFGMDPPEHTRQRQLLLPRFTVRQVAARRPAIQRIVDEHLDAM

EANGSPADLVSAFATPVPTMVVCTHLGVPYQDRTRFEPAVAGLFEPDRAD

AAMAELTAYLHQLIETKQSEPGDGVIDHLIANHLRPGAIDRAELVAIASA

ILVAGTVTTSSAIALGTLALLTAPGQYAALVDNPDLVPGAVNEILRYLSL

VEQLARVATEDIEIGGKLIRAGDGIIVSFAAGNLDPNVTTHPDRLDVALP

PTNHLAFSHGIHHCIGQNLALLELDIAFRALVSRFPTLRLAVPAEQLPTY

FAGDVPRLACLPVTW

>CYP107FS2(2516014126)*Salinispora arenicola* CNX508

MPVPQGEQNLTTEVFADPKALFATLGSRQPLHRISLPDGMPAVLVTGNRE

ARQALSDPRLVRSITAAAPELHKYHPLASDDYALSRHMLFADPPDHGRMR

KLVSTAFTRRRVEQMRPRIQQITDDLIDVIAAKGEADLVETLALPLPIAV

ISEMLGVPFADRSEFERHAEVLTGINASSGFDAIIAAGRWFDEYLAELVQ

QRRREPQDDLISGMLAAQDKGDRLTDVELRSNALLLLSAGFETTVNLVAN

GLLALLRHPEAMAALRSEPNLMTTAVDELLRYDSPVSCVTYHFAQEPVEI

GGFEIRSGEHVVIAAAAANHDPTVFADPSRLDLRREGSGQILSFSHGIHF

CLGAPLARLEGEIAFGTVLRRLAGLTLAVPTDSLVWKASFVLHRLERLPV

TFTPDRAPNPIDSVHTV

>CYP208A12(2516014158)*Salinispora arenicola* CNX508

MTLDTITPRVPLGPPRTAALRMLLVMKRDRLGMLTSAAARYGDASRLPVG

HKALWFFNHPRYAKHVLADNSANYHKGIGLVHARRALGDGLLTSEGDLWR

KQRKVIQPAFQSRRIAQQAGMIAEEAFALVERLRARAGAGPVELTAELTG

LTLGVLGRSLLDADLAGFDSIGDSFATVQDQAMFELETLNAVPMWIPLPR

QIRFRRARRKLQAVVDTLVDGRAGNLADRVDVLSRLILSARGEADPRVGR

ERLRDELVTLLLAGHETTASTLGWTLSLIDRHPGVWERLHAEAVEVLGDR

LPEYDDLRRLRYTVMVVEEAMRLFPPVWLLPRRALAPDTIGEYRVPANAD

VVISPYTLHRHPEFWPNPERFDPERFAPGQAADRPRYAYLPFGAGPRFCV

GNNLGMMEAVFVIALLCRHLRLTGVPGYRLVPEPMLSLRIRGGLPLVVRP

VS

>CYP1005A1(2516014320)*Salinispora arenicola* CNX508

VSAVLFRSWTKTAGTRWPDVTRVADQSGTEHLVVTRHALVRQVLTDQATY

RPDNALEAVTPIPVAALRVLAGHRFRLPPTLANNGGVSHPAIRALVADAL

HPTKVAAQRPWLTGLVADRVASIRTTLDSGGPVDLYADLTADLPLLVLAR

LVELPDAPVNAVKQFARAALELFWAPLDADRQLALADEVGRFHQVLREFA

DTGGGLAAALRATGHSPDVLVGALFFLLVAGQETTSQFLTLLLHRLSGEP

TIRAALRAGSISVADVVEEGLRLEPPIVTWRRVAAVDSTLGGTTVAAGTS

VLLWLARAGRDPAVVAAPDEFRPGQRGSRRHLAFGAGAHRCLGDQLARME

AAVVVEQATPLLDGVTVVRPPWYPDNLTFRMPDAFVVRR

>CYP211C1(2516014419)*Salinispora arenicola* CNX508

VVDVEELLTRLYSAQGRQDPFPVYADLHAQGPIAALPPEPERRRVAAVAV

GYDLVGAVLRDPEWSKAPPPGWTEQEILRTLQTSMMFINPPDHGRMRHVF

AGTFTPRRLGALEPVVNRVADELLDRMADAGAGGLDFVAEFAYPLPARVM

AEFIGIPETELDWYRERVDVIDAFLDVAGKTPQRLAAANAAGAELRAFYG

ELLARRRRTPGEDLISGLVEAVDAGGVELTEDELVSNLIVLFNASFVTTV

YMLSNGLPVLLAHPEVAAALATDPVLTAGAVDEILRLQAPVHLLARAAPR

DTVLGGVPIPQGQNVLLLIAAANRDPAHFPDPDRFDPWRSGPPSLAFGLG

LHYCLGAAVSRLEGRLALPRLLSRFPRLRIMEQPVYSGSLFLRGIDKLSV

SPGEGSTRE

>CYP107AY2(2516014671)*Salinispora arenicola* CNX508

MTAEPTPIPRSGARLGQEYDQLRKTGDVHQVLLPDASLAWLVTNPEVAAR

ALADPRLALNRRNSRGGWSGFALPPALDANLLNLDAPDHTRLRRLVGPAF

SPQRVAALRPGIRRAAEHLLDTLVATSGPTDLVTGYCNPLSVRVIADLMG

VPEAGRTNLRAWTDTMLTSYPPDRDAIRQAVTELHGYVVDLIDIKQQQPG

DDLLSALVTIEQDGDRLSRDELTSLAFLILFAGYENTANLIASAVLWLLD

HGGLNVVPISEAIEATLRHEPPAPVAIRRFPTEDIIIGGLTIPAGDTVLL

SVAAATRGADGNAARLAFGNGPHYCLGAALARVEAEEALTVLARRLPGLT

LAVPPSQVRWRPTFRTHGPAELLVGW

>CYP105AB8(2516014717)*Salinispora arenicola* CNX508

MTETASSRLTDTEFPVQRECPFAEPVEYEQIREQSSIAMVRLTGGGEAWW

ISGHEQGRAVLADRRFSSDRRKANFPFVSTDPAIRKRLHAQPLSLISMDG

AEHTQARRALIGEFTVRRLAALRPRIQQIVDQCIDEMLTTDQHCADLVKA

LSLPVPSLVICELLGVPYADHDFFQEHTATLVRRNTASEVRQHSIDELNA

YLGALIDRKLASPDDDLLGRQIARQHRDGTFDRSSMVSLAFLLLVAGHET

TANMISLGVVGLLQHPEQLAMIKNDPDKTPLAIEELLRFFTIVDSVTSRV

ATEDVRFGDTTINAGDGVVVSGLSADWDPTVFADPDRLDLERGARHHLAF

GFGPHQCLGQNLARLELQIVFDTLFHRIPTLRLAAPLDKIPFKTDAAIYG

ARELPVAW

>CYP105W2(2516015234)*Salinispora arenicola* CNX508

MTGYQDRPTGDQPGAPVPSGSTDPGIGAFPLPRRCPFSPPAEYARLRAEH

PVVRLPMLGGDTAWVVSRHADVRQVLSDPRMSADRRRPGFPKFAPTTEGQ

RQASFANFRPPLNWLDPPEHAICRRQIVDEFSVRRVRQSRALVERVVDTH

LDALTAAAPGADLVSTFAYPVPSQVICEVLGVPYGEHEFFERRSTLMFRR

STPADERARCAREIRDFLDVVVTDKERRPGDDVLSRLLYRQRSAGGMDHE

AVVSMAFVLLVAGHVTTSNMLALSVLALLTHPARLARLRAEPERFPAAVE

ELLRYFTVVEAATARTTTAEVTIGGVTIAAGEGVVALGQAANRDPRVFEH

PDEFDPDRDARAHLAFGHGRHICPGQHLARLEMEVALSRLFRRLPGLRLT

MEVSDLPLKEDSNIFGLYALPVAW

>CYP248A2(2516015241)*Salinispora arenicola* CNX508

VLADAVTAFDPTAVDVRRDPYPSYHWLLRHDPVHRGAHQVWYVSRFADVR

AVLGDERFARTGIRRFWTDLVGPGLLSQIVGDIILFQDEPDHGRLRGVVG

PAFSPSALRRLEPTIEATVNDLLRPARALGAMDVVADLAYPLALRAVLEL

LGLPAGDANAVGRWSRAVGRTLDRGATAEDMRRGHAAIAEFADYVERALA

ERREDGADLLALMLAAHRSQLMSRNEIVSTVVTFIFTGHETVASQLGNGL

LSLLDHPEQLELVRRQPHLVPQAVEECLRFDPAVQSNTRQLAADVELHGR

RLRRDDVVVVLAGAANRDPGRYDRPDELDIRRDPVPSMSFGAGMRYCLGS

YLARLQLRTALGAMVALPDLRVVCSPNELAYQPRTMFRGLTRLPVAFTPA

G

>CYP211B2(2516015494)*Salinispora arenicola* CNX508

MDVSEAIAVLISPSGRLDPYPTYEQLRAHGPVSRTTAGLFVVTGYAEADM

VLRDPRFVVLDDDLRDDVFPHWQDSPAIKSIARSMLRTNPPDHSRIRRLA

AGAFTPRRVAAMREVVTAQADELVDEMIRAGRDGARVDFMDMFAYPLPVA

VICALLGVPAADRSRFRRWAGDLTGILEPEITPEDLAGADAGADELRDYF

TGLIEQRRRAPADDLTTALVQAHDADGDRLSGEELLANLVVLLVAGFETT

TNLLGNGLVVLLTRPEAAAALRDEPDLAPGYVDELLRYDSPVQLTTRTVR

ESVSFAGTELPAGSWLLVLLGAANRDPRRFPDPARFDPWRAQSQPLSFGA

GPHYCLGAGLARLEAQVAFPLLLRRLPELALAGRPSRRTRLTLRGYETLP

ITVGAVTADRGTPAGVAPGTP

>CYP166A4(2516016425)*Salinispora arenicola* CNX508

MTDAISFELPWARTDKFDPPAVFDALREQRPLARMRYPDGHVGWIVSSYE

LVREVLGDPRFSHSCAVGHFPVTHQGQVIPTHPQIPGMFIHMDPPEHTRY

RRLLTGEFTVRRTSRLTGHVEGVATEQIEVMREHGAPADLVATFARPLVL

RVLSGLVGLPYGERDRYLHAVTLLHDAEADPAEAAAAYEQAGAYFDEVIE

RRRRQPEDDLISTLVGGGELTGEELRNIVTLLLFAGYETTESALAVGMFA

LLHHEDQLARLRADPTKIDAAIEELLRYLTVNQYHTYRTASEDIELHGEV

INKGDSVTVSLPAANRDPARFACPAELDIDRETSGHVAFGFGIHQCLGQN

LARVELRAGLSALLRAFPNLRLAVPADEVPLRLQGSVFAVKNLPVCW

>CYP105BL2(2517989361)*Salinispora arenicola* CNY237

MSSHSAAAPDPETATPLHTLAPELTFPQFERSTPFDPPQAYTELSGRCPV

APVSMADGKPSWLITSFEGVRTTLSDPRFSSDMSHPGFPNRTGKPVDDLL

KDTLGAMDGERHRYYRRMLTGELTVRRAKAMRPVITQITDEALDQLAAAG

PGADLVKHVAFVVPSRVACHLVGIPLSDYELFTGMAATLMDSTSSDDQFA

ALQNMVSYFDTLVTDREHHDRDDLLGHMVRRYLATGELTRDMLIRLAWTT

MAAGQETTAHMIGLGVAALLRHPDQLELLRREPHLLPGAVDELMRYLPLI

QFGIPRVAMDDVEVDGQTVTAGEGVVALPPLANRDPAVFERPDELDVRRN

ARQHLTFGYGPHQCPAHALARLELEVVYGRLLERFPTLRLADSDADLKVQ

DKDIMYRVSELAVTW

>CYP211C1(2517989908)*Salinispora arenicola* CNY237

VVDVEELLTRLYSAQGRQDPFPVYADLHAQGPIAALPPEPERRRVAAVAV

GYDLVGAVLRDPEWSKAPPPGWTEQEILRTLQTSMMFINPPDHGRMRHVF

AGTFTPRRLGALEPVVNRVADELLDRMADAGAGGLDFVAEFAYPLPARVM

AEFIGIPATELDWYRERVDVIDAFLDVAGKTPQRLAAANAAGAELRAFYG

ELLARRRRTPGEDLISGLVEAVDAGGVELTEDELVSNLIVLFNASFVTTV

YMLSNGLPVLLAHPEVAAALATDPVLTAGAVDEILRLQAPVHLLARAAPR

DTVLGGVPIPQGQNVLLLIAAANRDPAHFPDPDRFDPWRSGPPSLAFGLG

LHYCLGAAVSRLEGRLALPRLLSRFPRLRIMEQPVYSGSLFLRGIDKLSV

SPGEGSTRE

>CYP125A41(2517990056)*Salinispora arenicola* CNY237

MTEPRIPAGFDFTDPEVLAHRVPREEFAELRRTAPVWWNAQPRGSAGFDD

DGYWVVTRYADVMTVSRDSDTYSTRENTAIARLRPDTTREDIEMQRVIML

NVDPPEHTKLRAIVSRGFTPRAINALRGSLAERAEHIVRDAAVRGVGDFV

SDVACELPLQAIAELIGVPQHHRRKVFDWSNQLIGYDDPAYGTDPLTASA

ELLAYAMEMAEERQRSPSDDLVTKLVNAQIDGEHLTTDEFGFFVMLLAVA

GNETTRNAITHGMVAFLDNPEQWELFKAERPKSAVEEIIRWATPVNVFQR

TALVDTVLGGQAISAGQRVALFYGSANFDEAVFEDPERFDITRSPNPHLG

FGGSGAHFCLGANLARLEIELIFNSIADHLPDIRKVAAPQRLRSGWINGI

RQMPVRYR

>CYP105J3(2517990232)*Salinispora arenicola* CNY237

MTDSVAFPQGRVCPHQPAPGYRPLAVQRPLAQVTLYDGRRVWAVTTRDLA

RRLLVDPRISSDRTNPAWPAIVPIVAAAVNDAQQKVLKIATALVGTDGPE

HKAQRKMLIPSFTFRRMNALRPMIQEIVDQQLDEMIKSGAPTDLIPAFAS

AVPVTVLYRLMGIPDDDHGIFEKLSHQLLAGPNANEAYDQLMGYMSRLIA

ERRRNPGEGVLDDLLAQHGANDDADHDELVSTLVVQVAGNHGTTGSMIAL

GLFALLQHPEQLAELRADPSLMPTAVDELLRFLSVPDAVTRLAADDIEVE

GTIIRKGDGVFFITSLINRDTDVHDAPNSLGWHHASAADHLTFGFGAHQC

LGQSLARITMEIALGALIDRLPSLRLAVPAEEVPFLPAASLQVIAELPIT

W

>CYP245A7(2517990711)*Salinispora arenicola* CNY237

MPSATLPRFALTGWSRENIVNPYPVYQRYREVASVHRGEPGGDAPDTFYV

FSYDEVVQVLSSNCFGRGRSLDAAKASVPVPAEQKALRAIVENWLVFMDP

PRHTELRSLLNRSFSPRIVTELRPRIARIAQELLSRLGQQVDVDLVESFA

APLPILVISELLGIPEERRAWLRANALALQEASSSRAGRDVDGYARAEVA

AQEFTEYFREQVRLRRGRAGGDLITILANAQQRGAPVSLDAIVGTCVHLL

TAGHETTTNSLAKAVLALREHPAVLDELRGAEGLTTDAVEEFLRYDPPVQ

AVTRWAHQDTTLGGCDIPRGSRVVALLGSANRDPARFPSPDVLDVRRPAD

RHLSFGLGIHYCLGATLARAELEIGLQALLDGVPTLGYGTQHVDYADDLV

FHGPSRLVLVNLGERCK

>CYP244A4(2517990715)*Salinispora arenicola* CNY237

MSTTTNTELTEAPETNMPVDPGLFDCMPDLIAAARVAPVVRIPYLGRHAW

VVCDRELVKQALTHPKMGKDIALVPEWMRQPGLMVTAQPDPEYARAMIMS

DGENHARIRRIHAPVLSPRNTERWGERVADKVEGFLDELSQAGSGGSAEV

NVVTNYTHKIPLAFISEMLGLPPEAEHRLRGITDIMLYSSDYAARREAIG

GLFGAVEDWVRNPADLRDGVITGLLAASDGPDAAVTEGEVIVWTLGMIIT

GYETTGSLISTSLYEAIRRPPHERPKTDEDITAWIEETLRVHPPFPHPTW

RFPLEDIELGGYLIPKGAPVQVSIAAANRKPGEGADSFDAERRGHGHLSF

GLGMHYCIGAPLVRLEAQIAVRGFLRRFPQARLSAETAVQWESEWMIRRM

SVLPAVLS

>CYP105CP2(2517990935)*Salinispora arenicola* CNY237

MTKSMPVQDLPAFPIPRECPYRPSAQHVSLRSGGPMAKVRLYNGRTAWLV

TDSAHARAVLSDYRRVSIKPYHGNYPLLNEEFEKVVDSGYADVLFGVDPP

EHTRQRQMIMPSFTLRRTAVLRPDIQRIVDDKLDEMMRHGAPGDLVTEFA

QPVPSMVMSFLLGVPWEDHEEFETPAHKLFVPELAEEATTELGAYLERLI

QKKEQPGGTPGGTGLLDDLIRDHLRAGALSRDELVHIAMAMLVAGTDTTT

NVISLGTLALLDNPDQWAALRDNPDELIPGAVEEILRYTSLIEAFARVAV

SDIELNGAVIKEGEGILISSAGVNFDPALAPDPGRFDIRRPPRPSFSFSH

GIHRCPGDNLARLELEIAFRSLVTRMPNLRTAKPIDQIPSNNNDGTLQRL

YELPVVW

>CYP105CN1(2517990937)*Salinispora arenicola* CNY237

MAAPAPQATQSTTPHPPSYPLPRECPYRPSAGTARLRDAGPVSTVRLYDG

RTAWLVTGAAEARALLADSRVSNRADFPNYPVMDERHLSMRATREMAREE

EGGFAAALFGMDPPEHTRQRQLLLPRFTVRQVAARRPAIQRIVDEHLDAM

EANGSPADLVSAFATPVPTMVVCTHLGVPYQDRTRFEPAVAGLFEPDRAD

AAMAELTAYLHQLIETKQSEPGDGVIDHLIANHLRPGAIDRAELVAIASA

ILVAGTVTTSSAIALGTLALLTAPGQYAALVDNPDLVPGAVNEILRYLSL

VEQLARVATEDIEIGGKLIRAGDGIIVSFAAGNLDPNVTTHPDRLDVALP

PTNHLAFSHGIHHCIGQNLALLELDIAFRALVSRFPTLRLAVPAEQLPTY

FAGDVPRLACLPVTW

>CYP107FS2(2517990938)*Salinispora arenicola* CNY237

MPVPQGEQNLTTEVFADPKALFATLGSRQPLHRISLPDGMPAVLVTGNRE

ARQALSDPRLVRSITAAAPELHKYHPLASDDYALSRHMLFADPPDHGRMR

KLVSTAFTRRRVEQMRPRIQQITDDLIDVIAAKGEADLVETLALPLPIAV

ISEMLGVPFADRSEFERHAEVLTGINASSGFDAIIAAGRWFDEYLAELVQ

QRRREPQDDLISGMLAAQDKGDRLTDVELRSNALLLLSAGFETTVNLVAN

GLLALLRHPEAMAALRSEPNLMTTAVDELLRYDSPVSCVTYHFAQEPVEI

GGFEIRSGEHVVIAAAAANHDPTVFADPSRLDLRREGSGQILSFSHGIHF

CLGAPLARLEGEIAFGTVLRRLAGLRLAVPTDSLVWKASFVLHRLERLPV

TFTPDRAPNPIDSVHTV

>CYP166A4(2517991631)*Salinispora arenicola* CNY237

MTDAISFELPWARTDKFDPPAVFDALREQRPLARMRYPDGHVGWIVSSYE

LVREVLGDPRFSHSCAVGHFPVTHQGQVIPTHPQIPGMFIHMDPPEHTRY

RRLLTGEFTVRRTSRLTGHVEGVATEQIEVMREHGAPADLVATFARPLVL

RVLSGLVGLPYGERDRYLHAVTLLHDAEADPAEAAAAYEQAGAYFDEVIE

RRRRQPEDDLISTLVGDGELTGEELRNIVTLLLFAGYETTESALAVGMFA

LLHHEDQLARLRADPTKIDAAIEELLRYLTVNQYHTYRTASEDIELHGEV

INKGDSVTVSLPAANRDPARFACPAELDIDRETSGHVAFGFGIHQCLGQN

LARVELRAGLSALLRAFPNLRLAVPADEVPLRLQGSVFAVKNLPVCW

>CYP1051A1(2517991881)*Salinispora arenicola* CNY237

MATDAAITRARTVPAWKALPAAVRDTHRAFVDVGNWSDGDVVRVSLGVSR

PYLVTNPAHVQEVLHERAAIYPRGDDTALWRSVRKLVGDGILAEGDAWAA

SRRVLAPMFRPARINAMVDTMADAIAGAVDDLHGAATAGTPIDVGRELSR

IVCSAIMRVFFADRITVRDALRIMKAQETIVTAMAPRILAPLVPWWIPMP

GDRRFRAAVRSIDDILLPVLRQAQRQPDDGDDLLSRLVRARADDGRALSE

KRMRDDLVSMVAVTTETSTVVLTWLWPLLANHPDVANRLYDEIDRVVGGG

PVRGDHLAELTYTRMVLDELLRLYPAGWILPRRAATTDVLGGVRINKGAT

VILSPYVTQRMTAWWGPTAEAFDPERFAAGREAADGRHRYAYYPFGVGMH

RCLGEHLFNLEAILIVATLLSRFRFALTDTSMPGVKVAASTRPARTVEMI

LKPVAPVPAR

>CYP105G5(2517991905)*Salinispora arenicola* CNY237

LTIETTETPPADDSLRAPLPRQFMQRDDPSKLPPALAALAEQSPVGRSTL

PDGDPFWMVSGYDEARAVLSDPRFSSDRFRYHPRFKKLSGQLGERLRNDK

ARAGSFINMDPPEHTRYRKLLTGQFTVRRMRQLTVRIEQIVTEQVDVMLA

EGNSADLVSAFAVPVPSLMICELLGVRYEDRTEFQRRAAGLLQTDLPIKQ

AVENLEAQRAFMQRLVTDKRRTPADDMISGLVHHAGAEPPLTDDELVGIA

TLLLFAGLDTTASMLGLGMFMLLQRPEQMAVLRDDPSRIGDAVEELLRYL

TVVSTGLFRFAKEDVVLGDEHIPAGSTVVVSLMAANRDGRHWPEPETLDV

TRVRSSHLAFGHGVHQCLGQQLARIELTVGITELLRRLPNVRLAVPPADV

PLRNDMITYGVHRLPILWDTL

>CYP107Q4(2517991906)*Salinispora arenicola* CNY237

MTTTAETSAETIDLFSPEVVADPFGWYARLREETGPTTGTLNIGTMMGGP

EMWLVTRYEDVRQVLTDPRFLTNPPADSPLEDIRAGVFKRLDFPPDLIPW

MANLLNVSDGEDHTRLRKLVSYALTAHRIGKLRPRVEKITADLLDKLAED

GKDGSPVDLVEEYCYPLPVTVICELVGIDEPDRPHWRAWGDSMATMNGER

IPTTLVKCIELARELIAKRRAEPQDDLVTALVQAQAEDQNRVSDDEIIGI

LFSLVTAGHQTTTYLIGNSVILLLENPDQLARLKENPSMWPQAVRELQRL

GPIQFAQPRFPSEDIELGGVTIPRGAPVAPLLLAANTDPRRFPDPNKLII

DRLAVGSEGHLGFGKGIHRCLGQHLAYQEAEVALQGLFTRFPDLSLAVPR

EEIPWILRPGFTRTRTLPLKLV

>CYP1005A1(2517992030)*Salinispora arenicola* CNY237

VSAVLFRSWTKTAGTRWPDVTRVADQSGTEHLVVTRHALVRQVLTDQATY

RPDNALEAVTPVPVAALRVLAGHRFRLPPTLANNGGASHPAIRALVADAL

HPTKVAAQRPWLTGLVADRVASIRTTLDSGGPVDLYADLTADLPLLVLAR

LVELPDAPVNAVKQFARAALELFWAPLDADRQLALADEVGRFHQVLREFA

DTGGGLAAALRATGHSPDVLVGALFFLLVAGQETTSQFLTLLLHRLSGEP

TIRAALRAGSISVADVVEEGLRLEPPIVTWRRVAAVDSTLGGTTVAAGTS

VLLWLARAGRDPAVVAAPDEFRPGQRGSRRHLAFGAGAHRCLGDQLARME

AAVVVEQATPLLDGVTVVRPPWYPDNLTFRMPDAFVVRR

>CYP208A12(2517992137)*Salinispora arenicola* CNY237

MTLDTITPRVPLGPPRTAALRMLLVMKRDRLGMLSSAAARYGDASRLPVG

HKALWFFNHPRYAKHVLADNSANYHKGIGLVHARRALGDGLLTSEGDLWR

KQRKVIQPAFQSRRIAQQAGMIAEEAFALVERLRARAGAGPVELTAELTG

LTLGVLGRSLLDADLAGFDSIGDSFARVQDQAMFELETLNAVPMWIPLPR

QIRFRRARRKLQAVVDTLVDGRAGNLAGRVDVLSRLILSARGEADPRVGH

ERLRDELVTLLLAGHETTASTLGWTLSLIDRHPGVWERLHAEAVEVLGDR

LPEYDDLRRLRYTVMVVEEAMRLFPPVWLLPRRALAPDTIGEYRVPANAD

VVISPYTLHRHPEFWPNPERFDPERFAPGQAADRPRYAYLPFGAGPRFCV

GNNLGMMEAVFVIALLCRHLRLTGVPGYRLVPEPMLSLRIRGGLPLVVRP

VS

>CYP105AB8(2517992846)*Salinispora arenicola* CNY237

MTETASSRLTDTEFPVQRECPFAEPVEYEQIREQSSIAMVRLTGGGEAWW

ISGHEQGRAVLADRRFSSDRRKANFPFVSTDPAIRKRLHAQPLSLISMDG

AEHTQARRALIGEFTVRRLAALRPRIQQIVDQCIDEMLTTDQHRADLVKT

LSLPVPSLVICELLGVPYADHDFFQEHTATLVRRNTASEVRQHSIDELNA

YLGALIDRKLASPDDDLLGRQIARQHRDGTFDRSSMVSLAFLLLVAGHET

TANMISLGVVGLLQHPEQLAMIKDDPDKTPLAIEELLRFFTIVDSVTSRV

ATEDVRFGDTTINAGDGVVVSGLSADWDPTVFADPDRLDLERGARHHLAF

GFGPHQCLGQNLARLELQIVFDTLFHRIPTLRLAAPLDKIPFKTDAAIYG

ARELPVAW

>CYP107AY2(2517992949)*Salinispora arenicola* CNY237

MTAEPTPIPRSGARLGQEYDQLRKTGDVHQVLLPDASLAWLVTNPEVAAR

ALADPRLALNRRNSRGGWSGFALPPALDANLLNLDAPDHTRLRRLVGPAF

SPQRVAALRPGIRRAAEHLLDTLVATSGPTDLVTGYCNPLSVQVIADLMG

VPEAGRTNLRAWTDTMLTSYPPDRDAIRRAVTELHGYVVDLIDIKQQQPG

DDLLSTLVTIEQDGDRLSRDELTSLAFLILFAGYENTANLIASAVLWLLD

HGGLNVVPISEAIEATLRHEPPAPVAIRRFPTEDIIIGGVTIPAGDTVLL

SVAAATRGADGNAARLAFGNGPHYCLGAALARVEAEEALTVLARRLPGLT

LAVPPSQVRWRPTFRTHGPAELLVGW

>CYP105CT1(2517992980)*Salinispora arenicola* CNY237

MNSPNHMPADRSLTAPTSGCPMALSRGRVGLDVADEISELRDGGRLGRIT

TAFGQEATLITRYDEVRAQMADSVVFNVAGVPSPPALVDGGFDTESVRRR

RTVGNLIMLDPPEHTRLRRMVAAWFTTRRVERLRPRVVEIIDAALDEMER

SGPPVDLVAMFAKTVPITVICELIGVPEELRERYRRRAERAVSASAVSTP

LDELRRLREAGWVSRELIEYHRENPSDDIIGMLLREHGTDSHDDGITDDE

LVGLANALLIAGHETTTQMLSMGTLALLRHPDQLALLRDDPSIVARAVEE

LLRYVGVLHGGFVRVATRDTRLGGHRIHAGELVVPALTAANRDPRLLTDG

DRLDITRPPTSHVAFGHGVHFCIGAPLARMELREAFPALLRRFPGLRLAV

PDSELEFTQGTTVYSLRGLPVTW

>CYP154M5(2517992997)*Salinispora arenicola* CNY237

VEQSCPYKLDVTGRDVHAEGEAIRARGPVAQVELPGGVQGWSVTGYQAAR

QVLADPRFAKDPKKWPAYTSGAIPPNWPLIGWLLMDNMTTNDGADHQRLR

KLVSHGFTPRQVERTRPLIVKIVNDLLDGLSSAGPDEVVDLKGRFATPLP

ARVICDMFGVPEALRASVLRGAQVNVTSSISGEEAEANVEQWHRELLELV

EAKREKPDEDMASLLIAAKEEDGSTLTQEEVVGTLHLMLGAGSETLMNAL

SYAVLGMLSNPGQYEMVRNGTSSWDDVIEETLRAQAPVAQLPLRYATEDV

AVGGAVIKAGDPVLMGFTAIGRDPAVHGETAGDYDITREDKTHLSFGHGV

HFCLGAPLARLELKIALPALFERFPNMTLAVRPDQLEPQGTFIMNGHREL

PVRLGQPATVLA

>CYP211B2(2517993465)*Salinispora arenicola* CNY237

MDVSEAIAVLISPSGRLDPYPTYEQLRAHGPVSRTTAGLFVVTGYAEADM

VLRDPRFVVLDDDLRDDVFPHWQDSPAIKSIARSMLRTNPPDHSRIRRLA

AGAFTPRRVAAMREVVTAQADELVDEMIRAGRDGARVDFMDMFAYPLPVA

VICALLGVPAADRSRFRRWAGDLTGILEPEITPEELAGADAGADELRDYF

TGLIEQRRRAPADDLTTALVQAHDADGDRLSGEELLANLVVLLVAGFETT

TNLLGNGLVVLLTRPEAAAALRDEPDLAPGYVDELLRYDSPVQLTTRTVR

ESVSFAGTELPADSWLLVLLGAANRDPRRFPDPARFDPGRAQSQPLSFGA

GPHYCLGAGLARLEAQVAFPLLLRRLPELALAGRPSRRTRLTLRGYETLP

ITVGAVTADRGTPAGVAPGTP

>CYP107EU1(2517993823)*Salinispora arenicola* CNY237

VTIGQTLPDLVYSPEFTRDPYAIFARLREQAPVCRVTTHRGMSAWMVTRH

ADVRALLADNRLAKDGNRIGELMPRHSTLTGAATGFPPGLTTNMVNSDPP

DHTRLRHLVGREFTGHRVEGLRPRIEEIVDDLLDGVAACGDEADLAETLA

RRLPIAVIGELLGVPEADRAEFFRWADTLYGGTASPEALGQAYNAIVDYL

GRLCDAKRDVPADDLLTALVQVSADEDRLSREELVSMALLLLVAGHETTS

KQISNGVLALLLNPEQLKLLKAQPARTAGAVEELLRFEGPSLSASLRFTT

EPVEVAGVVIPEGEFVLLSLASGNRDPEKFPDPDRLDITRSTQGNLAMGH

GIHHCVGAALARLELEIVLSRLVARFPQMQLAVEADDLEWLVNSFFRAPL

HLPVSLRR

>CYP105W2(2517993866)*Salinispora arenicola* CNY237

MTGYQDRPTGDQPGAPVPSGSTDPGIGAFPLPRRCPFSPPAEYARLRAEH

PVVRLPMLGGDTAWVVSRHADVRQVLSDPRMSADRRRPGFPKFAPTTEGQ

RQASFANFRPPLNWLDPPEHAICRRQIVDEFSVRRVRQSRALVERVVDTH

LDALTAAAPGADLVSTFAYPVPSQVICEVLGVPYGEHEFFERRSTLMFRR

STPADERARCAREIRDFLDMVVTDKEHRPGDDVLSRLLYRQRRAGGVDHE

AVVSMAFVLLVAGHVTTSNMLALSVLALLTHPARLARLRAEPERFPAAVE

ELLRYFTVVEAATARTATAEVTIGGVTIAAGEGVVALGQAANRDPRVFEH

PDEFDPDRDARAHLAFGYGRHICPGQHLARLEMEVALSRLFRRLPGLRLT

MEVSDLPLKEDSNIFGLYALPVAW

>CYP248A2(2517993874)*Salinispora arenicola* CNY237

VLADAVTAFDPTAVDVRRDPYPSYHWLLRHDPVHRGAHQVWYVSRFADVR

AVLGDERFARTGIRRFWTDLVGPGLLSQIVGDIILFQDEPDHGRLRGVVG

PAFSPSALRRLEPTIEATVNDLLRPARALGAMDVVADLAYPLALRAVLEL

LGLPAGDANAVGRWSRAVGRTLDRGATAEDMRRGHAAIAEFADYVERALA

ERREDGADLLALMLAAHRSQLMSRNEIVSTVVTFIFTGHETVASQLGNGL

LSLLDHPEQMELMRRQPHLLPHAVEECLRFDPAVQSNTRQLAADVELHGR

RLRRDDVVVVLAGAANRDPGRYDRPDELDIRRDPVPSMSFGAGMRYCLGS

YLARLQLRTALGAMVALPDLRLVCSPNELAYQPRTMFRGLTRLPVAFTPA

G

>CYP105CH1(2517993920)*Salinispora arenicola* CNY237

VSSLPLPTYPKLRDPADPLLPPAEYLAIQSEKPIAKVLLPSGRPTWLITG

HALARQVLTEPCVSVDRRHPNFPYPVPNPDAVVAQVARWTYILLGDDPPL

HTERRRLLISEFTVRQAQAMRPRIQQLVDFHLEQLIAAGPGADFSKHFAM

QVPSAVICEMLGVPFADHDYFQERTALQLRRDVPVAAQKQAIDELLAYFE

QLIQEKSSHPGDDVLSRLIVSNRETEAFDHEALVALGLLLLVGGHETTAN

TLTLATATMLERPEIAEQLRTDPSLMPSAVEEFLRYFSVAVAVSRIATAD

LQVGGQLVRAGESMLLVLNTIARDGTVFPEPHRLDIRRNARNHLAFSHGI

HQCMGQNLARVEMQIALDTVLRRLPGLHLVTPFEELPFKYRHLVWGIEEL

RVAW

>CYP1005A1(2517867720)*Salinispora arenicola* CNT005

VSAVLFRSWTKTAGTRWPDVTRVADQNGTEHLVVTRHALVRQVLTDQATY

RPDNALEAVTPIPVAALRVLAGHRFRLPPTLANNGGVSHPAIRALVADAL

HPTKVAAQRPWLTGLVADRVASIRTTLDSGGPVDLYADLTADLPLLVLAR

LVELPDAPVNAVKQFARAALELFWAPLDADRQLALADEVGRFHQVLREFA

DTGGGLAAALRATGHSPDVLVGALFFLLVAGQETTSQFLTLLLHRLSGEP

TIRAALRAGSSSVADVVEEGLRLEPPIVTWRRVAAVDSTLGGTTVAAGTS

VLLWLARAGRDPAVVAAPDEFRPGQRGSRRHLAFGAGAHRCLGDQLARME

AAVVVEQATPLLDGVTVVRPPWYPDNLTFRMPDAFVVRR

>CYP125A41(2517869090)*Salinispora arenicola* CNT005

MTEPRIPAGFDFTDPEVLAHRVPREEFAELRRTAPVWWNAQPRGSAGFDD

DGYWVVTRYADVMTVSRDSDTYSTRENTAIARLRPDTTREDIEMQRVIML

NVDPPEHTKLRAIVSRGFTPRAINALRGSLAERAEHIVRDAAVRGVGDFV

TDVACELPLQAIAELIGVPQHHRRKVFDWSNQLIGYDDPAYGTDPLTASA

ELLAYAMEMAEERQRSPSDDLVTKLVNAQIDGEHLTTDEFGFFVMLLAVA

GNETTRNAITHGMVAFLDNPEQWELFKAERPKSAVEEIIRWATPVNVFQR

TALVDTVLGGQAISAGQRVALFYGSANFDEAVFEDPERFDITRSPNPHLG

FGGSGAHFCLGANLARLEIELIFNSIADHLPDIRKVAAPQRLRSGWINGI

RQMPVRYR

>CYP211C1(2517869238)*Salinispora arenicola* CNT005

VVDVEELLTRLYSAQGRQDPFPVYADLHAQGPIAALPPEPERRRVAAVAV

GYDLVGAVLRDPEWSKAPPPGWTEQEILRTLQTSMMFINPPDHGRMRHVF

AGTFTPRRLGALEPVVNRVADELLDRMADAGAGGLDFVAEFAYPLPARVM

AEFIGIPETELDWYRERVDVIDAFLDVAGKTPQRLAAANAAGAELRAFYG

ELLARRRRTPGEDLISGLVEAVDAGGVELTEDELVSNLIVLFNASFVTTV

YMLSNGLPVLLAHPEVAAALATDPVLTAGAVDEILRLQAPVHLLARAAPR

DTVLGGVPIPQGQNVLLLIAAANRDPAHFPDPDRFDPWRSGPPSLAFGLG

LHYCLGAAVSRLEGRLALPRLLSRFPRLRIMEQPVYSGSLFLRGIDKLSV

SPGGREYP

>CYP105W2(2517869353)*Salinispora arenicola* CNT005

MTGYQDRPTGDQPGAPVPSGSTDPGIGAFPLPRRCPFSPPAEYARLRAEH

PVVRLPMLGGDTAWVVSRHADVRQVLSDPRMSADRRRPGFPKFAPTTEGQ

RQASFANFRPPLNWLDPPEHAICRRQIVDEFSVRRVRQSRALVERVVDTH

LDALTAAAPGADLVSTFAYPVPSQVICEVLGVPYGEHEFFERRSTLMFRR

STPADERARCAREIRDFLDMVVTDKEHRPGDDVLSRLLYQQRSAGGVDHE

AVVSMAFVLLVAGHVTTSNMLALSVLALLTHPARLARLRAEPERFPAAVE

ELLRYFTVVEAATARTATAEVTIGGVTIAAGEGVVALGQAANRDPRVFEH

PDEFDPDRDARAHLAFGYGRHVCPGQHLARLEMEVALSRLFRRLPGLRLT

MEVSDLPLKEDSNIFGLYALPVAW

>CYP248A2(2517869361)*Salinispora arenicola* CNT005

VLADAVTAFDPTAVDVRRDPYPSYHWLLRHDPVHRGAHQVWYVSRFADVR

AVLGDERFARTGIRRFWTDLVGPGLLSQIVGDIILFQDEPDHGRLRGVVG

PAFSPSALRRLEPTIEATVNDLLRPARALGAMDVVADLAYPLALRAVLEL

LGLPAGDANAVGRWSRAVGRTLDRGATAEDMRRGHAAIAEFADYVERALA

ERREDGADLLALMLAAHRSQLMSRNEIVSTVVTFIFTGHETVASQLGNGL

LSLLDHPEQMELVRRQPHLVPQAVEECLRFDPAVQSNTRQLAADVELHGR

RLRRDDVVVVLAGAANRDPGRYDRPDELDIRRDPVPSMSFGAGMRYCLGS

YLARLQLRTALGAMVALPDLRLVCSPNELAYQPRTMFRGLTRLPVAFTPA

G

>CYP107FS2(2517869424)*Salinispora arenicola* CNT005

MPVPQGEQNLTTEVFADPKALFATLGSRQPLHRISLPDGMPAVLVTGNRE

ARQALSDPRLVRSITAAAPELHKYHPLASDDYALSRHMLFADPPDHGRMR

KLVSTAFTRRRVEQMRPRIQQITDDLIDVIAAKGEADLVETLALPLPIAV

ISEMLGVPFADRSEFERHAEVLTGINASSGFDAIIAAGRWFDEYLAELVQ

QRRREPQDDLISGMLAAQDKGDRLTDVELRSNALLLLSAGFETTVNLVAN

GLLALLRHPEAMAALRSEPNLMTTAVDELLRYDSPVSCVTYHFAQEPVEI

GGFEIRSGEHVVIAAAAANHDPTVFADPSRLDLRREGSGQILSFSHGIHF

CLGAPLARLEGEIAFGTVLRRLAGLRLAVPTDSLVWKASFVLHRLERLPV

TFTPDRAPNPIDSVHTV

>CYP105CN1(2517869425)*Salinispora arenicola* CNT005

MAAPAPQATQSTTPHPPSYPLPRECPYRPSAGTARLRDAGPVSTVRLYDG

RTAWLVTGAAEARALLADSRVSNRADFPNYPVMDERHLSMRATREMAREE

EGGFAAALFGMDPPEHTRQRQLLLPRFTVRQVAARRPAIQRIVDEHLDAM

EANGSPADLVSAFATPVPTMVVCTHLGVPYQDRTRFEPAVAGLFEPDRAD

AAMAELTAYLHQLIETKQSEPGDGVIDHLIANHLRPGAIDRAELVAIASA

ILVAGTVTTSSAIALGTLALLTAPGQYAALVDNPDLVPGAVNEILRYLSL

VEQLARVATEDIEIGGKLIRAGDGIIVSFAAGNLDPNVTTHPDRLDVALP

PTNHLAFSHGIHHCIGQNLALLELDIAFRALVSRFPTLRLAVPAEQLPTY

FAGDVPRLACLPVTW

>CYP105CP2(2517869427)*Salinispora arenicola* CNT005

MTKSMPVQDLPAFPIPRECPYRPSAQHVSLRSGGPMAKVRLYNGRTAWLV

TDSAHARAVLSDYRRVSIKPYHGNYPLLNEEFEKVVDSGYADVLFGVDPP

EHTRQRQMIMPSFTLRRTAVLRPDIQRIVDDKLDEMMRHGAPGDLVTEFA

QPVPSMVMSFLLGVPWEDHEEFETPAHKLFVPELAEEATTELGAYLERLI

QKKEQPGGTPGGTGLLDDLIRDHLRAGALSRDELVHIAMAMLVAGTDTTT

NVISLGTLALLDNPDQWAALRDNPDELIPGAVEEILRYTSLIEAFARVAV

SDIELNGAVIKEGEGILISSAGVNFDPALAPDPGRFDIRRPPRPSFSFSH

GIHRCPGDNLARLELEIAFRSLVTRMPNLRTAKPIDQIPSNNNDGTLQRL

YELPVVW

>CYP244A4(2517869650)*Salinispora arenicola* CNT005

MSTTTNTELTEAPETNMPVDPGLFDCMPDLIAAARVAPVVRIPYLGRHAW

VVCDRELVKQALTHPKMGKDIALVPEWMRQPGLMVTAQPDPEYARAMIMS

DGENHARIRRIHAPVLSPRNTERWGERVADKVEGFLDELSQAGSGGSTEV

NVVTNYTHKIPLAFISEMLGLPPEAEHRLRGITDIMLYSSDYAARREAIG

GLFGAVEDWVRNPADLRDGVITGLLAASDGPDAAVTEGEVIVWTLGMIIT

GYETTGSLISTSLYEAIRRPPHERPKTDEDITAWIEETLRVHPPFPHPTW

RFPLEDIELGGYLIPKGAPVQVSIAAANRKPGEGADSFDAERRGHGHLSF

GLGMHYCIGAPLVRLEAQIAVRGFLRRFPQARLSAETAVQWESEWMIRRM

SVLPAVLS

>CYP245A7(2517869654)*Salinispora arenicola* CNT005

MPSATLPRFALTGWSRENIVNPYPVYQRYREVASVHRGESGGDAPDTFYV

FSYDEVVQVLSSNCFGRGRSLDAAKASVPVPAEQKALRAIVENWLVFMDP

PRHTELRSLLNRSFSPRIVTELRPRIARIAQELLSRLGQQVDVDLVESFA

APLPILVISELLGIPEERRAWLRANALALQEASSSRAGRDVDGYAQAEVA

AQEFTEYFREQVRLRRGRAGGDLITILANAQERGAPVSLDAIVGTCVHLL

TAGHETTTNSLAKAVLALREHPAVLDELRGAEGLTTDAVEEFLRYDPPVQ

AVTRWAHQDTTLGGCDIPRGSRVVALLGSANRDPARFPSPDVLDVRRPAD

RHLSFGLGIHYCLGATLARAELEIGLQALLDGVPTLGYGTQHVDYADDLV

FHGPSRLVLVNLGERCK

>CYP113D13(2517869917)*Salinispora arenicola* CNT005

VNLADKLGFNQAQFWLRGVRPEELVSYQEQAGLWNVYGYPEIAEALSDPV

AFSSETQRLAGEWESFTEGNIVAMDPPAHKKLRKLVSQAFTPKVVADLEP

RIAVVTGDLLDKVSGRMELVADLAYPLPVIVIADLLGVPSSDHPLFKQWV

EAMFSTGTQISLKDPSPEQQEHFEQGMAQVENLTGYLKVHIRERRVQPRD

DLLTKLVEAEVDGQRLTDVEASNFAMALLVAGHLTTTMLLGNTILCLDAF

PDQRAKVQADRTLVPGAIEEAMRFLSPSGAVARVTTREVKLGGRTIPANQ

MVIMWVAAANRDPRQFTDPDVFDLTRDPNTQMSFGRGIHFCLGAPLARLE

SRVALNILLDRFPDLRTDSDDPPEVMPHPTMAGAKRLPLLL

>CYP163B22(2517869920)*Salinispora arenicola* CNT005

MPVTMQEGFRRTERREDIVVSAFATGHTVLDLTDPATFVNHEPGEFWAGV

RDHDPVFWHESGAGRPGFWVVSRHADVLACYADPVRLSSARGTVLDVLLR

GPDSAGGRMLAVSDRPRHRDLRAVMLRAFSPRVLGEVSDLVHRRAGELIS

RVTQGGAFDFAAAVAEHIPMGTICDLLSIPHADRPAMLGWNKEALSSDDA

EADPFAALAARNEILLYFTDLAAWRRARPGDDVISLIATAVVGDELLSLE

DVALNCYSLILGGDESSRVSAICAVEAFADFPDQWRAVRDGTVSIDTAVE

EVLRWATPALHFARTATTDLDLAGHRIRAGEIVTLWNISANFDERSFASP

NRFDVGRRPNKHVSFGHGPHFCLGAYLGRAELHALLTALAGHVTHIERAG

PARRIYSNFLNGHSSLPVSFTGR

>CYP105AB8(2517870058)*Salinispora arenicola* CNT005

MTETASSRLTDTEFPVQRECPFAEPVEYEQIREQSSIAMVRLTGGGEAWW

ISGHEQGRAVLADRRFSSDRRKANFPFVSTDPAIRKRLHAQPLSLISMDG

AEHTQARRALIGEFTVRRLAALRPRIQQIVDQCIDEMLTTDQHHADLVKT

LSLPVPSLVICELLGVPYADHDFFQEHTATLVRRNTASEVRQHSIDELNA

YLGALIDRKLASPDDDLLGRQIARQHRDGTFDRSSMVSLAFLLLVAGHET

TANMISLGVVGLLQHPEQLAMIKDDPDKTPLAIEELLRFFTIVDSVTSRV

ATEDVRFGDTTINAGDGVVVSGLSADWDPTVFADPDRLDLERGARHHLAF

GFGPHQCLGQNLARLELQIVFDTLFHRIPTLRLAAPLDKIPFKTDAAIYG

ARELPVAW

>CYP208A12(2517870310)*Salinispora arenicola* CNT005

MTLDTITPRVPLGPPRTAALRMLLVMKRDRLGMLSSAAARYGDASRLPVG

HKALWFFNHPRYAKHVLADNSANYHKGIGLVHARRALGDGLLTSEGDLWR

KQRKVIQPAFQSRRIAQQAGMIAEEAFALVERLRARAGAGPVELTAELTG

LTLGVLGRSLLDADLAGFDSIGDSFATVQDQAMFELETLNAVPMWIPLPR

QIRFRRARRKLQAVVDTLVDGRAGNLADRVDVLSRLILSARGEADPRVGR

ERLRDELVTLLLAGHETTASTLGWTLSLIDRHPGVWERLHAEAVEVLGDR

LPEYDDLRRLRYTVMVVEEAMRLFPPVWLLPRRALAPDTIGEYRVPANAD

VVISPYTLHRHPEFWPNPERFDPERFAPGQAADRPRYAYLPFGAGPRFCV

GNNLGMMEAVFVIALLCRHLRLTGVPGYRLVPEPMLSLRIRGGLPLVVRP

VS

>CYP105BL2(2517870542)*Salinispora arenicola* CNT005

MSSHSAAAPDPETATPLHTLAPELTFPQFERSTPFDPPQAYTELSGRCPV

APVSMADGKPSWLITSFEGVRTTLSDPRFSSDMSHPGFPNRTGKPVDDLL

KDTLGAMDGERHRYYRRMLTGELTVRRAKAMRPVITQITDEALDQLAAAG

PGADLVKHVAFVVPSRVACHLVGIPLSDYELFTGMAATLMDSTSSDDQFA

ALQNMVSYFDTLVTDREHHDRDDLLGHMVRRYLATGELTRDMLIRLAWTT

MAAGQETTAHMIGLGVAALLRHPDQLELLRREPHLLPGAVDELMRYLPLI

QFGIPRVAMDDVEVDGQTVTAGEGVVALPPLANRDPAVFERPDELDVRRN

ARQHLTFGYGPHQCPAHALARLELEVVYGRLLERFPTLRLADSDADLKVQ

DKDIMYRVSELAVTW

>CYP166A4(2517870718)*Salinispora arenicola* CNT005

MTDAISFELPWARTDKFDPPAVFDALREQRPLARMRYPDGHVGWIVSSYE

LVREVLGDPRFSHSCAVGHFPVTHQGQVIPTHPQIPGMFIHMDPPEHTRY

RRLLTGEFTVRRTSRLTGHVEGVATEQIEVMREHGAPADLVATFARPLVL

RVLSGLVGLPYGERDRYLHAVTLLHDAEADPAEAAAAYEQAGAYFDEVIE

RRRRQPEDDLISTLVGDGELTGEELRNIVTLLLFAGYETTESALAVGMFA

LLHHEDQLARLRADPTKIDAAIEELLRYLTVNQYHTYRTASEDIELHGEV

INKGDSVTVSLPAANRDPARFACPAELDIDRETSGHVAFGFGIHQCLGQN

LARVELRAGLSALLQAFPNLRLAVPADEVPLRLQGSVFAVKNLPVCW

>CYP1051A1(2517870815)*Salinispora arenicola* CNT005

MATDAAITRARTVPAWKALPAAVRDTHRAFVDVGNWSDGDVVRVSLGVSR

PYLVTNPAHVQEVLHERAAIYPRGDDTALWRSVRKLVGDGILAEGDAWAA

SRRVLAPMFRPARINAMVDTMADAIAGAIDDLHGAATAGTPIDVGRELSR

IVCSAIMRVFFADRITVRDALRIMKAQETIVTAMAPRILAPLVPWWIPMP

GDRRFRAAVRSIDDILLPVLRQAQRQPDDGDDLLSRLVHARADDGRALSE

KRMRDDLVSMVAVTTETSTVVLTWLWPLLANHPDVANRLYDEIDRVVGGG

PVRGDHLAELTYTRMVLDELLRLYPAGWILPRRAATTDILGGVRINKGAT

VILSPYVTQRMTAWWGPTAEAFDPERFAAGREAADGRHRYAYYPFGVGMH

RCLGEHLFNLEAILIVATLLSRFRFALTDTSMPGVKVAASTRPARTVEVI

LKPVAPVPAR

>CYP105G5(2517870841)*Salinispora arenicola* CNT005

LTIETTETPPADDSLRAPLPRQFMQRDDPSKLPPALAALAEQSPVGRSTL

PDGDPFWMVSGYDEARAVLSDPRFSSDRFRYHPRFKKLSGQLGERLRNDK

ARAGSFINMDPPEHTRYRKLLTGQFTVRRMRQLTVRIEQIVTEQVDVMLA

EGNSADLVSAFAVPVPSLMICELLGVRYEDRTEFQRRAAGLLQTDLPIKQ

AVENLEAQRAFMQRLVTDKRRTPADDMISGLVHHAGAEPPLTDDELVGIA

TLLLFAGLDTTASMLGLGMFMLLQRPEQMAVLRDDPSRIGDAVEELLRYL

TVVSTGLFRFAKEDVVLGDEHIPAGSTVVVSLMAANRDGRHWPEPETLDV

TRVRSSHLAFGHGVHQCLGQQLARIELTVGITELLRRLPNVRLAVPPADV

PLRNDMITYGVHRLPILWDTP

>CYP107Q4(2517870842)*Salinispora arenicola* CNT005

MTTTAETSAETIDLFSPEVVADPFGWYARLREETGPTTGTLNIGTMMGGP

EMWLVTRYEDVRQVLTDPRFLTNPPADSPLEDIRAGVFKRLDFPPDLIPW

MANLLNVSDGEDHTRLRKLVSYALTAHRIGKLRPRVEKITADLLDKLAED

GKDGSPVDLVEEYCYPLPVTVICELVGIDEPDRPHWRAWGDSMATMNGER

IPTTLVKCIELARELIAKRRAEPQDDLVTALVQAQAEDQNRVSDDEIIGI

LFSLVTAGHQTTTYLIGNSVILLLENPDQLARLKENPSMWPQAVRELQRL

GPIQFAQPRFPSEDIELGGVTIPRGAPVAPLLLAANTDPRRFPDPNKLII

DRLAVGSEGHLGFGKGIHRCLGQHLAYQEAEVALQGLFTRFPDLSLAVPR

EEIPWILRPGFTRTRTLPLKLV

>CYP107AY2(2517871581)*Salinispora arenicola* CNT005

MTAEPTPIPRSGARLGQEYDQLRKTGDVHQVLLPDASLAWLVTNPEVAAR

ALADPRLALNRRNSRGGWSGFALPPALDANLLNLDAPDHTRLRRLVGPAF

SPQRVAALRPGIRRAAEHLLDTLVATSGPTDLVTGYCNPLSVQVIADLMG

VPEAGRTNLRAWTDTMLTSYPPDRDAIRRAVTELHGYVVDLIDIKQQQPG

DDLLSTLVTIEQDGDRLSRDELTSLAFLILFAGYENTANLIASAVLWLLD

HGGLNVVPISEAIEATLRHEPPAPVAIRRFPTEDIIIGGVTIPAGDTVLL

SVAAATRGADGNAARLAFGNGPHYCLGAALARVEAEEALTVLARRLPGLT

LAVPPSQVRWRPTFRTHGPAELLVGW

>CYP154M5(2517871690)*Salinispora arenicola* CNT005

VEQSCPYKLDVTGRDVHAEGEAIRARGPVAQVELPGGVQGWSVTGYQAAR

QVLADPRFAKDPKKWPAYTSGAIPPNWPLIGWLLMDNMTTNDGADHQRLR

KLVSHGFTPRQVERTRPLIVKIVNDLLDGLSSAGPDEVVDLKGRFATPLP

ARVICDMFGVPEALRASVLRGAQVNVTSSISGEEAEANVEQWHRELLELV

EAKREKPDEDMASLLIAAKEEDGSTLTQEEVVGTLHLMLGAGSETLMNAL

SYAVLGMLSNPGQYEMVRNGTSSWDDVIEETLRAQAPVAQLPLRYATEDV

AVGGAVIKAGDPVLMGFTAIGRDPAVHGETAGDYDITREDKTHLSFGHGV

HFCLGAPLARLELKIALPALFERFPNMTLAVRPDQLEPQGTFIMNGHREL

PVRLGQPATVLA

>CYP105CT1(2517871707)*Salinispora arenicola* CNT005

MNSPNHMPADRSLTAPTSGCPMALSRGRVGLDVADEISELRDGGRLGRIT

TAFGQEAMLITRYDEVRAQMADSVVFNVAGVPSPPALVDGGFDTESVRRR

RTVGNLIMLDPPEHTRLRRMVAAWFTTRRVERLRPRVVEIIDAALDEMER

SGPPVDLVAMFAKTVPITVICELIGVPEELRERYRRRAERAVSASAVSTP

LDELRRLREAGWVSRELIEYHRENPSDDIIGMLLREHGTDSHDDGITDDE

LVGLANALLIAGHETTTQMLSMGTLALLRHPDQLALLRDDPSIVAGAVEE

LLRYVGVLHGGFVRVATRDTRLGGHRIHAGELVVPALTAANRDPRLLTDG

DRLDITRPPTSHVAFGHGVHFCIGAPLARMELREAFPALLRRFPGLRLAV

PDSELEFTQGTTVYSLRGLPVTW

>CYP105CH1(2517871762)*Salinispora arenicola* CNT005

VSSLPLPTYPKLRDPADPLLPPAEYLAIQSEKPIAKVLLPSGRPTWLITG

HALARQVLTEPCVSVDRRHPNFPYPVPNPDAVVAQVARWTYILLGDDPPL

HTERRRLLISEFTVRQAQAMRPRIQQLVDFHLEQLIAAGPGADFSKHFAM

KVPSAVICEMLGVPFADHDYFQERTALQLRRDVPVAAQKQAIDELLAYFE

QLIQEKSSHPGDDVLSRLIVSNRETEAFDHEALVALGLLLLVGGHETTAN

TLTLATATMLERPEIAEQLRTDPSLMPSAVEEFLRYFSVAVAVSRIATAD

LQVGGQLVRAGESMLLVLNTIARDGTVFPEPHRLDIRRNARNHLAFSHGI

HQCMGQNLARVEMQIALDTVLRRLPGLHLVAPFEELPFKYRHLVWGIEEL

RVAW

>CYP211B2(2517871999)*Salinispora arenicola* CNT005

MDVSEAIAVLISPSGRLDPYPTYEQLRAHGPVSRTTAGLFVVTGYAEADM

VLRDPRFVVLDDDLRDDVFPHWQDSPAIKSIARSMLRTNPPDHSRIRRLA

AGAFTPRRVAAMREVVTAQADELVDEMIRAGRDGARVDFMDMFAYPLPVA

VICALLGVPAADRSRFRRWAGDLTGILEPEITPEELAGADAGADELRDYF

TGLIEQRRRAPADDLTTALVQAHDADGDRLSGEELLANLVVLLVAGFETT

TNLLGNGLVVLLTRPEAAAALRDEPDLAPGYVDELLRYDSPVQLTTRTVR

ESVSFAGTELPADSWLLVLLGAANRDPRRFPDPARFDPGRAQSQPLSFGA

GPHYCLGAGLARLEAQVAFPLLLRRLPELALAGRPSRRTRLTLRGYETLP

ITVGAVTADRGTPAGVAPGTP

>CYP247A7(2517872040)*Salinispora arenicola* CNT005

VRLTPGAARDIDLDSVNLFDLDLYTSGDPHPIWDVMRAQSPLHHQVLADG

REFWSVTRYDDVCRVLGDYREFTSERGTVVTHLGEDDIAAGKLLTSTDPP

RHTQVRRAIGAKLTARAVASWQDRIRDAIVRFLEPALDGDTFDLAEQALL

LPAIVTGPLLGIPERDWQELVQLTAMVTAPSDPHFQHGSEAATLAISHHE

LVTYVTEWVKQRRSAGGGDGSLLDHLMSVRVGGAPLTDEEIALDGYSILL

GANVTTPHTVSGTVLALIERPEQFEKAQADPSLLANLVEEGLRWASAACN

FMRYAVNDTRIGGGTVPAGGAVVAWIGSANRDESYFPDPHQFDITRSGAN

RQVAFGFGPHYCIGAPLARMTLGIFFEELVQRFGSIELAGEPQHLRSYFI

AGMTHLPIVAQKRKTP

>CYP105J3(2517872124)*Salinispora arenicola* CNT005

MTDSVAFPQGRVCPHQPAPGYRPLAVQRPLAQVTLYDGRRVWAVTTRDLA

RRLLVDPRISSDRTNPAWPAIVPIVAAAVNDAQQKVLKIATALVGTDGPE

HKAQRKMLIPSFTFRRMNALRPMIQEIVDQQLDEMIKSGAPTDLIPAFAS

AVPVTVLYRLMGIPDDDHGIFEKLSHQLLAGPNANEAYDQLMGYMSRLIA

ERRRNPGEGVLDDLLAQHGANDDADHDELVSTLVVQVAGNHGTTGSMIAL

GLFALLQHPEQLAELRADPSLMPTAVDELLRFLSVPDAVTRLAADDIEVE

GTIIRKGDGVFFITSLINRDTDVHDAPNSLGWHHASAADHLTFGFGAHQC

LGQSLARITMEIALGALIDRLPSLRLAVPAEEVPFLPAASLQVIAELPIT

W

>CYP107EU1(2517872439)*Salinispora arenicola* CNT005

VTIGQTLPDLVYSPEFTRDPYAIFARLREQAPVCRVTTHRGMSAWMVTRH

ADVRALLADNRLAKDGNRIGELMPRHSTLTGAATGFPPGLTTNMVNSDPP

DHTRLRHLVGREFTGHRVEGLRPRIEEIVDDLLDGVAACGDEADLAETLA

RRLPIAVIGELLGVPEADRAEFFRWADTLYGGTASPEALGQAYNAIVDYL

GRLCDAKRDVPADDLLTALVQVSADEDRLSREELVSMALLLLVAGHETTS

KQISNGVLALLLNPEQLKLLKAQPARTAGAVEELLRFEGPSLSASLRFTT

EPVEVAGVVIPEGEFVLLSLASGNRDPEKFPDPDRLDITRSTQGNLAMGH

GIHHCVGAALARLELEIVLSRLVARFPQMQLAVEADDLEWLVNSFFRAPL

HLPVSLRR

>CYP1198B1(2517872723)*Salinispora arenicola* CNT005

MSGELTDQRTAPGAGGNPLRSLMDHGIRANPYPLFGELREAGPTAVEDGS

VVLFGEYEHCSQILRHRDMGSDTSEAPSIKGFVVDDAERAGSSIFFMDQP

GHGRQRKLVSKSFTPRIVKSFGPQITHIVDGLFEDFRDKGELDVVTDLAY

PVSIGIICDLFGIPDDERDMLKEWSDDLALSTELPTLGAAIGVLNVFTRD

EINRFGSVAMAAHAYFADLIHRRRKNPGDDLVSSLLATESNGERLTRFEV

TSVLATLFVAAHESTTNLISGGILALLRNQDQMAVLRENPGLITNVVDES

LRYDPPVHLAARMARARTTIGGYDLDPGTIVVVLMAAGNRDPRAYESPDV

FDVNRKIKNVSLAFGAGAHFCIGSGLAKLEAEIAISAFAQRLKNPEVDES

SLEYRRHIVVRGLEHMKVSFQP

>CYP107AY1(2517994832)*Salinispora tropica* CNS416

MRAELAPIPRSGARLGQEYDQLRNAGDVHQVLLPDSSLAWLVTNPKLVSR

ALTDPRLALNRRHSRGSWSGFALPPALDANLLNLDAPDHTRLRRLVGPAF

SPQRVSALRPRIRRTAEHLLDTLVATSGPVDLVTGYCTPLSVQVIADLMG

VPEAGRADLRTWTDTMLTSYPPDRDAIRQAVVELHGYVVDLIDTKRQQPG

DDLLSALVTIEQDGDRLTRDELTSLAFLILFAGYENTANLIASTVLRLLD

HGGLRGVQLPEAIEETLRLEPPAPAAVRRFPTEEMTIGGATIPAGDIVLL

SIAAATRGTAGNAARLAFGNGPHFCLGAALARVEAEEALTVLARRLPDLA

LALPVAQVRWRPTFRTHGPAELLVTW

>CYP163B1(2517995108)*Salinispora tropica* CNS416

VTAADTGRVGSGESQTPAHTVDLADPATFANHDLTGFWQQLRDEEPIHWN

PPTAGRRGFWVVSRYADILDVYRDDVTFTSERGNVLVTLLAGGDAGAGRM

LAVTDGPRHAELRKLLLRALGPRVLAPVCAAVRTNTRQMIREAVTKGECD

FASDIASRIPMMTISNLLGVPDADRAFLLSLTKTALSADDESISETESAM

ARNEILLYFQDLMEFRRDHPGEDVVSMLVNSSIDGAPLSDDDIVLNCYSL

IIGGDETSRLTMIDSVNTLAAHPQQWRRLKDGQCEIDKAVDEVLRWASPS

MHFGRVAARDTILHGVRIRADDIVTLWHASGNRDERVFHRPEVFDLGRTP

NRHLSFGHGPHYCIGSYLAKVEISELLIALRDLTSGFETTGEPQRIRSNL

LTGFATMPVRFVPDRAGLARDALDG

>CYP211C1(2517995453)*Salinispora tropica* CNS416

VLDVEGLLTRLYSEQGRQDPYPVYADLHAQGAIAALAPRPEGQRVAAVAV

GYDLVGAVLRDPEWSKQPPPGWMEQEILRTLQSSMMFINPPDHGRMRKVF

AGTFTPRRLGTLEPVINRVADELLDRMADAGPGEVDFVAEFAYPLPARVM

AEFIGIPATELAWYRDRVDRIDAFLDVAGKTPERLAAANAAAAELRVFYA

DLLARRRRTPGEDLISGLVEAVDAGGVQLTEDELINNLIVLFNASFVTTV

YMLSNGLPVLLEHPEVAAALADDPELTAGAIDEILRLQTPVHLLARAAPR

DTVLGGVPIPQGQNVLLLIAAANRDPAHFPDPDRFDPRRPGPPSLAFGLG

LHYCLGAAVSRLEGRLALPRLLSRFPRLRIMEQPVYSGSLFLRGIDKLSV

SPGGRMHP

>CYP125A15(2517995586)*Salinispora tropica* CNS416

MTEPRIPVGFDFTDPAVLERRVPREEFAELRRTAPVWWNVQPRGSAGFDD

DGYWVVTRYADVMAVSRDSDTYSTRENTAIARFQPGTTRADLEMQRVIML

NVDPPEHTKLRAIVSRGFTPRAINALRGSLAERAERIVRDAAVRGTGDFV

ADVACELPLQAIAELIGVPQHHRRKVFDWSNQLIGYDDPAYGVDPLTAAA

ELLAYAMEMANERQLNPSDDLVTKLVNAQIDGEHLTTDEFGFFVMLLAVA

GNETTRNAITHGMLAFLEHPEQWELFKAERPRSAVEEIIRWATPVNVFQR

TALVDTTLGGQAISAGQRVALFYGSANFDESVFEEPERFDITRSPNPHLG

FGGSGAHFCLGANLARLEIELIFHSIADHMPDIRKVAEPRRLRSGWINGI

REMPVRYR

>CYP208A4(2517995664)*Salinispora tropica* CNS416

VTVAAAGRTFSGPTGAALLRSLWQLGQDRLGLMTSAARYGDAVRLGVGSR

SLYFFNHPDHAKHVLADNSGNYTKGLGLVHARRALGDGLLTSEGELWREQ

RRVIQPVFQAKRVAGQAHAVAEEADRLIARLRARRGRGPVNLTDEFTALT

LGVLGRTLLDANLDAFTTVGAAFEEMQNQAMFEMASMSMVPMWVPLPQQL

RFRRARRELERIVGRLVADRTARGEGTGADDALSRLIASTRDEPDPGVAR

RRMRDELVTLLLAGHETTASTLGWTFHLINQDPRVRVRLREEAIDVLGGR

LPEYADLARLTYTKMVVSEAMRLYPPVWMLSRLARDADVVDGYPVPARAD

VLICPYTLHRHPAFWPEPERFDPERFDPEVTTDRPRYAYVPFGAGPRFCV

GNHLGLMEAVFVVAMVSREFDLVAPVGQPVVAEPMLSLRVRGGLSMTVEP

VS

>CYP154M1(2517995692)*Salinispora tropica* CNS416

MRRRCPVVIDPAGTDIHAEGARIRANGSVSQVELPGGVLAWSVTGQQVAR

KVLSDQRFSKDPRKHWTDYLEGRIGQDFPLIGWVLMDNLTTAYGSDHSRL

RKPCANAFTPRRVEALRPAVERAAVELLGELATVSPTESVDLKARYAHPL

PSRVICDLFGVPEEDREEMLRGGEVNVDTRVSAEEAAANVERWHQQMLDF

IEEKRRNPGPDLTSDLIAAQQAEGSRLTDSEMVGTLHIMLATGTEPVKNL

IGNAVFALLTHPEQLDLVRSGRAGWDDVIQETLRMQAPVAHLPFRFAVED

VDIDGVTIRRGDPLLVNFAAIGRDPDVHGDTAAEFDITRADKEHLSFGHG

VYRCIGQPLALREAEIALRMLFQRFPNLVLAVPPEEVTPQPTFIMNGLDT

LPVLLKGRA

>CYP107AW1(2517995954)*Salinispora tropica* CNS416

MESVTSTSAPPPVPYIADPYPALARIRANGPVSILHSDEGIPMWVIARYR

NVRAALADPRFGQDARRAQTLADNRVAGVTLGGDVIHMLNSDPPDHTRLR

HLVQGAFTARRVAAMRPLVERITTSLLDGVGGRQTVDLVQDFAFPLPMLV

ICELLGFPAEERDAYRSWSTAILTHNDDPAAFATALRDMTDYIEVQLRHR

RARPGEDLLTELLAARDAGQLTDDEIVGMVFLLLIGGHETTVNLLGTATL

ALVRNPDQHRWLLANPHALSEAIDEFLRYESPVAMATLRFTTAPVTVDDV

VIPAGELVLVSLGGANRDPDRFPDADRLILDRRDTGHLAFGHGLHRCLGA

FLGKLEGEVALGALLGRYPGLTLAAEVRQLRWRDTIMLRGLESLPVSLHG

>CYP211B1(2517996017)*Salinispora tropica* CNS416

MDASEAVALLMSPLGRIDPYPTYERLRAHGPVVQTAAGFFVVTGYTEADT

VLRNARLAFEVMDDELRDDVFPHWQDSPAMKSIARSMIRANPPNHGRMRR

LAAGAFTPRRIAALREVVTAQADELADEMIRAGRDGAPVDFMGSFAYPLP

VAVICALLGVPAADWARFRGWASDLTAVLEPEITPQELTVADAGASELRD

YFTELIAQRRRAPADDLTTALVQTHDADGDRLSGEELLANLVLLLVAGFE

TTTNLLGNGLVVLLAHPDSATALRDQPELAPGYVDELLRYDSPVQLTTRT

VRESVLLAGVELPAGSWVLVLLGAANRDPERFTDPTRFDPGRAQSPPLSF

GAGAHYCLGAGLARLEAQVAFPLLLRRLPELALAGEPTRRNRLTLRGYET

LPVTVSAIAADHGTPAGVARGTP

>CYP107AX1(2517997454)*Salinispora tropica* CNS416

MTSRPTAVFDQCLLRDPHSRYNALRDQAPVHHVLTPDGAPAWLVTRYNDV

RAAFTDPRLSVDKRFSGTDGEHGSSLPPELDAHLLNRDPPDHTRLRRLAA

AACTPRRVADLHPAVERIVSTLLDGLAGHDRAELIGSLASPLPLQVMHEL

LGLPTQANIDFRTWTNTLLSADANQPAQSRAAMANMRRFLIEQLAHKRAQ

PGDDLLTGLLAAREDDDRLTDDELVAMVFLLMFAGYDNTAALIGTVTHAL

LTNAELHEAVRGGSLALDELIDEVLRWNPAFPLAVRRFAREPITIAGQTI

PAGDRIWLCLASANRDPAQFTQPDELGIIGLRRSHLSFGHGIHYCLGAPL

ARLQTTIAVTSLLNRFPEMRLAVPAHDIRWRESFRLRGLIALPVYL

>CYP1005A1(2517997727)*Salinispora tropica* CNS416

VSAVLFRSWTKTAGTHWPAITRVADQQGTEHLVVTQHALVRQVLTDQLTY

RPDNALDAVTPIPVAALRVLAGHRFRLPPTLANNGGVSHPAIRALVADAL

HPAKVAAQRPWLTGLVAERVAAIRRTLDSGGSTDLHAELNADLPLLVLAR

LVELPDAPVSAVKQFARAALELFWAPLDADRQLALADEVGRFHQVLREFA

DTGGGLAAALRATGHPPDVLVGALFFLLVAGQETTSQFLTLLLHRLADEP

TVRAALRADSVSVADVVEEGLRLEPPIVTWRRVAAVDSTLGGSTVAAGTS

VLLWLARAGRDPAVVPAPDEFRPGQRGSRRHLAFGAGAHRCLGDQLARME

AAVVVEQVTPLLDGVTVVRPPWYPDNLTFRMPDAFVVRR

>CYP105AB2(2517997972)*Salinispora tropica* CNS416

MTETASIATTRTASGQLTDAEFPVQRGCPFTTPTEYEQIREESSIAKVRL

KNGGEAWWIAGHELGRSVLADRRFSSDRRRDNFPFVSTDPETRAQLQSQP

TSMLGMDGAEHAQTRRALMGEFTVRRMAGLRPRIQQIVDQHIDEMLATPQ

RSVDLVEALSLPVPSLVICELLGVPYADHDFFQGLTGPLLRHTTPPEVRL

RIQEELNTYLGTLIDHKLTDPTDDLLSRQIAKHRDNGTFDRASMVSLAFL

LLVAGHETTANMISLGVVGLLQHPDQLVIIKDDPDKTPLAVEELLRYFTI

ADSVTARVATEDVQLGDTTINAGDGVVISGLAADRDPTVFAEPDRLDLER

GARHHVAFGFGPHQCIGQTLARMELRIVFDTLFHRIPTLRLAAPLDDIPF

KSDAFVYGIEELPVAW

>CYP1004B1(2517998671)*Salinispora tropica* CNS416

MTTSALAPRFDALDPNVVEDPYPEYARLRAAGPLCRLGPGSWGVTRFADV

TNLQHDPRLGSEFPAGYHEISVGDGPASAFFQRVMLYRDPPDHIRLRRLM

SGAFTPAVVRRLRSHIEDLVDELLAPALAAGRMDLVPELAYPLPVRVVCR

LMGIPPESTEDVRHHATNIGRAFTAVVPEQARTEADEAVSWLREHLGALL

EQRRSHRGDDLLSRLLDAEESGDNLSADEIVDNTVFSFFAGFETTVHMIT

TGTAALLAHPDQLARLRADPSLVTTAVDEFLRWDAPIQGTARYVREPIEI

GGRTIRRGRVLVLMIGSANHDERRFAQPDRLDVGRQDNPHVAFGGGAHLC

LGAFLARMEGAVVFDRLARLAVLEPDGPTVREPNTPFRAYASVPVRIGDR

>CYP1004A1(2517998675)*Salinispora tropica* CNS416

MRANTARPHLSAAFLATKDDPYPAYAELRARGPLTRAELGQWLVTGHGAV

SALLRDGRLESRMPAEYTRLTLGDSPGVDFLHRIVLTRTPPEHTRLRRFI

GRALGTPVVRRLHDRIAAATDALLEPALDRGRLDVVTELAVPLPVGVVCD

LIGIPTGDRPAVLTRVTALAKVFDAANLSPADLADINTALPWLHDYFGDL

LAVRRAGSGGPTLTEMYWEESASDRLAVADFVDNMLFLFHAGFETTMGLV

SNGVAALLNNPEQLGRLRADPALVPSAVEEFLRYDAPIQNVIRVARKPVE

VAGQKIRAGRTVLLLLGAANRDEEVFADAERLDVGRDPNPHLGFGGGLHH

CVGTALARLMAVVVFERLVDRVTVLGPAAPAVRRRHASLRSYDHLPLAVA

AR

>CYP125G1(2517998714)*Salinispora tropica* CNS416

MSTEVVSTGNRADIAHPATYAAGVPYAEFARLRRDKPVSWVPEAALWRRS

GEGRILSQGPGFWAVTTHEGVVAASRQPEVFSSGRQGAFLADPRTTADLE

QARQLLVNMDAPQHARVRKLVTAVFTPRAIRALGDSVTAHARNLVERAVR

QEECDVVADLAAELPLLVLADLLGLPREDRHLLYQWSNNLVGFDDPEYGG

GDVEAYRKTFFEAFQYALSVAGERRRAPREDLMTLLATSEVDGRRLTDRE

FCNFWLLLVVAGNETTRHLITGSVLALVDNPMQRERLVADDTLLPSAVDE

LLRWVSPIMQFRRTAIVDTELCGTPIAAGDKVVLWYTSANRDAAVFEAPD

ELRLHRNPNPHLSFGMGPHFCLGAHLARLEARTMLRELAPHLSRFRLTGP

VVRLESNFVNGVKSLPGSFTGQ

>CYP107E3(2517999187)*Salinispora tropica* CNS416

VTIDQEIRKYPFCESPGIGIDPTYGLLRSTEPLARVQLPYGEVSWLATRY

EDVKTVLTDPRFSRAAAQGKDQPRTREEMTYEGIIGLDPPDHTRLRKLAG

KALTARRVNAIRADAQRIANEYVDEMIAKGSPGDLVELFALPYPVTVICE

LLGVPFEDRAQFRIWTEGLTSTSEQLMVYAEQLFGYMGKLVAQRREEPTD

DLLGALVKARDEGDRLTEQELLSIAGVGLLLTGVETVSTHIPNFVYALLT

HPELMAQLRADRSLVPAAVEELLRMIPLNPAAMFPRYAVEDVTLSGITVR

AGEPVLVSLPGANRDPEVFENPETFDFTREQNPHVAFGHGPHHCLGAQLA

RMELQVALHTVLDRFPDLSLADGDEGVSWKSGLLVRGPSRLLVAW

>CYP163B1(2527070068)*Salinispora tropica* CNR416

VTAADTGRVGSGESQTPAHTVDLADPATFANHDLTGFWQQLRDEEPIHWN

PPTAGRRGFWVVSRYADILDVYRDDVTFTSERGNVLVTLLAGGDAGAGRM

LAVTDGPRHAELRKLLLRALGPRVLAPVCAAVRTNTRQMIREAVTKGECD

FASDIASRIPMMTISNLLGVPDADRAFLLSLTKTALSADDESISETESAM

ARNEILLYFQDLMEFRRDHPGEDVVSMLVNSSIDGAPLSDDDIVLNCYSL

IIGGDETSRLTMIDSVNTLAAHPQQWRRLKDGQCEIDKAVDEVLRWASPS

MHFGRVAARDTILHGVRIRADDIVTLWHASGNRDERVFHRPEVFDLGRTP

NRHLSFGHGPHYCIGSYLAKVEISELLIALRDLTSGFETTGEPQRIRSNL

LTGFATMPVRFVPDRAGLARDALDG

>CYP154M1(2527070702)*Salinispora tropica* CNR416

MRRRCPVVIDPAGTDIHAEGARIRANGSVSQVELPGGVLAWSVTGQQVAR

KVLSDQRFSKDPRKHWTDYLEGRIGQDFPLIGWVLMDNLTTAYGSDHSRL

RKPCANAFTPRRVEALRPAVERAAVELLGELATVSPTESVDLKARYAHPL

PSRVICDLFGVPEEDREEMLRGGEVNVDTRVSAEEAAANVERWHQQMLDF

IEEKRRNPGPDLTSDLIAAQQAEGSRLTDSEMVGTLHIMLATGTEPVKNL

IGNAVFALLTHPEQLDLVRSGRAGWDDVIQETLRMQAPVAHLPFRFAVED

VDIDGVTIRRGDPLLVNFAAIGRDPDVHGDTAAEFDITRADKEHLSFGHG

VYRCIGQPLALREAEIALRMLFQRFPNLVLAVPPEEVTPQPTFIMNGLDT

LPVLLKGRA

>CYP208A4(2527070730)*Salinispora tropica* CNR416

VTVAAAGRTFSGPTGAALLRSLWQLGQDRLGLMTSAARYGDAVRLGVGSR

SLYFFNHPDHAKHVLADNSGNYTKGLGLVHARRALGDGLLTSEGELWREQ

RRVIQPVFQAKRVAGQAHAVAEEADRLIARLRARRGRGPVNLTDEFTALT

LGVLGRTLLDANLDAFTTVGAAFEEMQNQAMFEMASMSMVPMWVPLPQQL

RFRRARRELERIVGRLVADRTARGEGTGADDALSRLIASTRDEPDPGVAR

RRMRDELVTLLLAGHETTASTLGWTFHLINQDPRVRVRLREEAIDVLGGR

LPEYADLARLTYTKMVVSEAMRLYPPVWMLSRLARDADVVDGYPVPARAD

VLICPYTLHRHPAFWPEPERFDPERFDPEVTTDRPRYAYVPFGAGPRFCV

GNHLGLMEAVFVVAMVSREFDLVAPVGQPVVAEPMLSLRVRGGLSMTVEP

VS

>CYP125A15(2527070808)*Salinispora tropica* CNR416

MTEPRIPVGFDFTDPAVLERRVPREEFAELRRTAPVWWNVQPRGSAGFDD

DGYWVVTRYADVMAVSRDSDTYSTRENTAIARFQPGTTRADLEMQRVIML

NVDPPEHTKLRAIVSRGFTPRAINALRGSLAERAERIVRDAAVRGTGDFV

ADVACELPLQAIAELIGVPQHHRRKVFDWSNQLIGYDDPAYGVDPLTAAA

ELLAYAMEMANERQLNPSDDLVTKLVNAQIDGEHLTTDEFGFFVMLLAVA

GNETTRNAITHGMLAFLEHPEQWELFKAERPRSAVEEIIRWATPVNVFQR

TALVDTTLGGQAISAGQRVALFYGSANFDESVFEEPERFDITRSPNPHLG

FGGSGAHFCLGANLARLEIELIFHSIADHMPDIRKVAEPRRLRSGWINGI

REMPVRYR

>CYP211C1(2527070941)*Salinispora tropica* CNR416

VLDVEGLLTRLYSEQGRQDPYPVYADLHAQGAIAALAPRPEGQRVAAVAV

GYDLVGAVLRDPEWSKQPPPGWMEQEILRTLQSSMMFINPPDHGRMRKVF

AGTFTPRRLGTLEPVINRVADELLDRMADAGPGEVDFVAEFAYPLPARVM

AEFIGIPATELAWYRDRVDRIDAFLDVAGKTPERLAAANAAAAELRVFYA

DLLARRRRTPGEDLISGLVEAVDAGGVQLTEDELINNLIVLFNASFVTTV

YMLSNGLPVLLEHPEVAAALADDPELTAGAIDEILRLQTPVHLLARAAPR

DTVLGGVPIPQGQNVLLLIAAANRDPAHFPDPDRFDPRRPGPPSLAFGLG

LHYCLGAAVSRLEGRLALPRLLSRFPRLRIMEQPVYSGSLFLRGIDKLSV

SPGGRMHP

>CYP107AY1(2527071010)*Salinispora tropica* CNR416

MRAELAPIPRSGARLGQEYDQLRNAGDVHQVLLPDSSLAWLVTNPKLVSR

ALTDPRLALNRRHSRGSWSGFALPPALDANLLNLDAPDHTRLRRLVGPAF

SPQRVSALRPRIRRTAEHLLDTLVATSGPVDLVTGYCTPLSVQVIADLMG

VPEAGRADLRTWTDTMLTSYPPDRDAIRQAVVELHGYVVDLIDTKRQQPG

DDLLSALVTIEQDGDRLTRDELTSLAFLILFAGYENTANLIASTVLRLLD

HGGLRGVQLPEAIEETLRLEPPAPAAVRRFPTEEMTIGGATIPAGDIVLL

SIAAATRGTAGNAARLAFGNGPHFCLGAALARVEAEEALTVLARRLPDLA

LALPVAQVRWRPTFRTHGPAELLVTW

>CYP107AW1(2527071254)*Salinispora tropica* CNR416

MESVTSTSAPPPVPYIADPYPALARIRANGPVSILHSDEGIPMWVIARYR

NVRAALADPRFGQDARRAQTLADNRVAGVTLGGDVIHMLNSDPPDHTRLR

HLVQGAFTARRVAAMRPLVERITTSLLDGVGGRQTVDLVQDFAFPLPMLV

ICELLGFPAEERDAYRSWSTAILTHNDDPAAFATALRDMTDYIEVQLRHR

RARPGEDLLTELLAARDAGQLTDDEIVGMVFLLLIGGHETTVNLLGTATL

ALVRNPDQHRWLLANPHALSEAIDEFLRYESPVAMATLRFTTAPVTVDDV

VIPAGELVLVSLGGANRDPDRFPDADRLILDRRDTGHLAFGHGLHRCLGA

FLGKLEGEVALGALLGRYPGLTLAAEVRQLRWRDTIMLRGLESLPVSLHG

>CYP211B1(2527071555)*Salinispora tropica* CNR416

MDASEAVALLMSPLGRIDPYPTYERLRAHGPVVQTAAGFFVVTGYTEADT

VLRNARLAFEVMDDELRDDVFPHWQDSPAMKSIARSMIRANPPNHGRMRR

LAAGAFTPRRIAALREVVTAQADELADEMIRAGRDGAPVDFMGSFAYPLP

VAVICALLGVPAADWARFRGWASDLTAVLEPEITPQELTVADAGASELRD

YFTELIAQRRRAPADDLTTALVQTHDADGDRLSGEELLANLVLLLVAGFE

TTTNLLGNGLVVLLAHPDSATALRDQPELAPGYVDELLRYDSPVQLTTRT

VRESVLLAGVELPAGSWVLVLLGAANRDPERFTDPTRFDPGRAQSPPLSF

GAGAHYCLGAGLARLEAQVAFPLLLRRLPELALAGEPTRRNRLTLRGYET

LPVTVSAIAADHGTPAGVARGTP

>CYP1005A1(2527071860)*Salinispora tropica* CNR416

VSAVLFRSWTKTAGTHWPAITRVADQQGTEHLVVTQHALVRQVLTDQLTY

RPDNALDAVTPIPVAALRVLAGHRFRLPPTLANNGGVSHPAIRALVADAL

HPAKVAAQRPWLTGLVAERVAAIRRTLDSGGSTDLHAELNADLPLLVLAR

LVELPDAPVSAVKQFARAALELFWAPLDADRQLALADEVGRFHQVLREFA

DTGGGLAAALRATGHPPDVLVGALFFLLVAGQETTSQFLTLLLHRLADEP

TVRAALRADSVSVADVVEEGLRLEPPIVTWRRVAAVDSTLGGSTVAAGTS

VLLWLARAGRDPAVVPAPDEFRPGQRGSRRHLAFGAGAHRCLGDQLARME

AAVVVEQVTPLLDGVTVVRPPWYPDNLTFRMPDAFVVRR

>CYP107AX1(2527073163)*Salinispora tropica* CNR416

MTSRPTAVFDQCLLRDPHSRYNALRDQAPVHHVLTPDGAPAWLVTRYNDV

RAAFTDPRLSVDKRFSGTDGEHGSSLPPELDAHLLNRDPPDHTRLRRLAA

AACTPRRVADLHPAVERIVSTLLDGLAGHDRAELIGSLASPLPLQVMHEL

LGLPTQANIDFRTWTNTLLSADANQPAQSRAAMANMRRFLIEQLAHKRAQ

PGDDLLTGLLAAREDDDRLTDDELVAMVFLLMFAGYDNTAALIGTVTHAL

LTNAELHEAVRGGSLALDELIDEVLRWNPAFPLAVRRFAREPITIAGQTI

PAGDRIWLCLASANRDPAQFTQPDELGIIGLRRSHLSFGHGIHYCLGAPL

ARLQTTIAVTSLLNRFPEMRLAVPAHDIRWRESFRLRGLIALPVYL

>CYP105AB2(2527073541)*Salinispora tropica* CNR416

MTETASIATTRTASGQLTDAEFPVQRGCPFTTPTEYEQIREESSIAKVRL

KNGGEAWWIAGHELGRSVLADRRFSSDRRRDNFPFVSTDPETRAQLQSQP

TSMLGMDGAEHAQTRRALMGEFTVRRMAGLRPRIQQIVDQHIDEMLATPQ

RSVDLVEALSLPVPSLVICELLGVPYADHDFFQGLTGPLLRHTTPPEVRL

RIQEELNTYLGTLIDHKLTDPTDDLLSRQIAKHRDNGTFDRASMVSLAFL

LLVAGHETTANMISLGVVGLLQHPDQLVIIKDDPDKTPLAVEELLRYFTI

ADSVTARVATEDVQLGDTTINAGDGVVISGLAADRDPTVFAEPDRLDLER

GARHHVAFGFGPHQCIGQTLARMELRIVFDTLFHRIPTLRLAAPLDDIPF

KSDAFVYGIEELPVAW

>CYP125G1(2527074094)*Salinispora tropica* CNR416

FCNFWLLLVVAGNETTRHLITGSVLALVDNPMQRERLVADDTLLPSAVDE

LLRWVSPIMQFRRTAIVDTELCGTPIAAGDKVVLWYTSANRDAAVFEAPD

ELRLHRNPNPHLSFGMGPHFCLGAHLARLEARTMLRELAPHLSRFRLTGP

VVRLESNFVNGVKSLPGSFTGQ

>CYP1004A1(2527074133)*Salinispora tropica* CNR416

MRANTARPHLSAAFLATKDDPYPAYAELRARGPLTRAELGQWLVTGHGAV

SALLRDGRLESRMPAEYTRLTLGDSPGVDFLHRIVLTRTPPEHTRLRRFI

GRALGTPVVRRLHDRIAAATDALLEPALDRGRLDVVTELAVPLPVGVVCD

LIGIPTGDRPAVLTRVTALAKVFDAANLSPADLADINTALPWLHDYFGDL

LAVRRAGSGGPTLTEMYWEESASDRLAVADFVDNMLFLFHAGFETTMGLV

SNGVAALLNNPEQLGRLRADPALVPSAVEEFLRYDAPIQNVIRVARKPVE

VAGQKIRAGRTVLLLLGAANRDEEVFADAERLDVGRDPNPHLGFGGGLHH

CVGTALARLMAVVVFERLVDRVTVLGPAAPAVRRRHASLRSYDHLPLAVA

AR

>CYP1004B1(2527074137)*Salinispora tropica* CNR416

MTTSALAPRFDALDPNVVEDPYPEYARLRAAGPLCRLGPGSWGVTRFADV

TNLQHDPRLGSEFPAGYHEISVGDGPASAFFQRVMLYRDPPDHIRLRRLM

SGAFTPAVVRRLRSHIEDLVDELLAPALAAGRMDLVPELAYPLPVRVVCR

LMGIPPESTEDVRHHATNIGRAFTAVVPEQARTEADEAVSWLREHLGALL

EQRRSHRGDDLLSRLLDAEESGDNLSADEIVDNTVFSFFAGFETTVHMIT

TGTAALLAHPDQLARLRADPSLVTTAVDEFLRWDAPIQGTARYVREPIEI

GGRTIRRGRVLVLMIGSANHDERRFAQPDRLDVGRQDNPHVAFGGGAHLC

LGAFLARMEGAVVFDRLARLAVLEPDGPTVREPNTPFRAYASVPVRIGDR

>CYP125A41(2571107809)*Salinispora arenicola* CNH996B

MTEPRIPAGFDFTDPEVLAHRVPREEFAELRRTAPVWWNAQPRGSAGFDD

DGYWVVTRYADVMTVSRDSDTYSTRENTAIARLRPDTTREDIEMQRVIML

NVDPPEHTKLRAIVSRGFTPRAINALRGSLAERAEHIVRDAAVRGVGDFV

TDVACELPLQAIAELIGVPQHHRRKVFDWSNQLIGYDDPAYGTDPLTASA

ELLAYAMEMAEERQRSPSDDLVTKLVNAQIDGEHLTTDEFGFFVMLLAVA

GNETTRNAITHGMVAFLDNPEQWELFKAERPKSAVEEIIRWATPVNVFQR

TALVDTVLGGQAISAGQRVALFYGSANFDEAVFEDPERFDITRSPNPHLG

FGGSGAHFCLGANLARLEIELIFNSIADHLPDIRKVAAPQRLRSGWINGI

RQMPVRYR

>CYP211C1(2571107958)*Salinispora arenicola* CNH996B

VVDVEELLTRLYSAQGRQDPFPVYADLHAQGPIAALPPEPERRRVAAVAV

GYDLVGAVLRDPEWSKAPPPGWTEQEILRTLQTSMMFINPPDHGRMRHVF

AGTFTPRRLGALEPVVNRVADELLDRMADAGAGGLDFVAEFAYPLPARVM

AEFIGIPETELDWYRERVDVIDAFLDVAGKTPQRLAAANAAGAELRAFYG

ELLAHRRRTPGEDLISGLLEAVDAGGVELTEDELVSNLIVLFNASFVTTV

YMLSNGLPVLLAHPEVAAALATDPVLTAGAVDEILRLQAPVHLLARAAPR

DTVLGGVPIPQGQNVLLLIAAANRDPAHFPDPDRFDPRRSGPPSLAFGLG

LHYCLGAAVSRLEGRLALPRLLSRFPRLRIMEQPVYSGSLFLRGIDKLSV

SPGEGSTRE

>CYP211B2(2571108288)*Salinispora arenicola* CNH996B

MDVSEAVAVLISPSGRLDPYPTYEQLRAHGPVSRTTAGLFVVTGYAEADM

VLRDPRFVVLDDDLRDDVFPHWQDSPAIKSIARSMLRTNPPDHSRIRRLA

AGAFTPRRVAAMREVVTAQADELVDEMIRAGRDGARVDFMDMFAYPLPVA

VICALLGVPAADRSRFRRWAGDLTGILEPEITPEELAGADAGADELRDYF

TGLIEQRRRAPADDLTTALVQAHDADGDRLSGEELLANLVVLLVAGFETT

TNLLGNGLVVLLTRPEAAAALRDEPDLAPGYVDELLRYDSPVQLTTRTVR

ESVSFAGTELPAGSWLLVLLGAANRDPRRFPDPARFDPWRAQSQPLSFGA

GPHYCLGAGLARLEAQVAFPLLLRRLPELALAGRPSRRTRLTLRGYETLP

ITVGAVTADRGTPAGVAPGTP

>CYP244A4(2571108768)*Salinispora arenicola* CNH996B

MSTTTNTELTEAPETNMPVDPGLFDCMPDLIAAARVAPVVRIPYLGRHAW

VVCDRELVKQALTHPKMGKDIALVPEWMRQPGLMVTAQPDPEYARAMIMS

DGENHARIRRIHAPVLSPRNTERWGERVADKVEGFLDELSQAGSGGSTEV

NVVTNYTHKIPLAFISEMLGLPPEAEHRLRGITDIMLYSSDYAARREAIG

GLFGAVEDWVRNPADLRDGVITGLLAASDGPDAAVTEGEVIVWTLGMIIT

GYETTGSLISTSLYEAIRRPPHERPKTDEDITAWIEETLRVHPPFPHPTW

RFPLEDIELGGYLIPKGAPVQVSIAAANRKPGEGADSFDAERRGHGHLSF

GLGMHYCIGAPLVRLEAQIAVRGFLRRFPQARLSAETAVQWESEWMIRRM

SVLPAVLS

>CYP245A7(2571108772)*Salinispora arenicola* CNH996B

MPSATLPRFALTGWSRENIVNPYPVYQRYREVASVHRGEPGGDAPDTFYV

FSYDEVVQVLSSNCFGRGRSLDAAKASVPVPAEQKALRAIVENWLVFMDP

PRHTELRSLLNRSFSPRIVTELRPRIARIAQELLSRLGQQVDVDLVESFA

APLPILVISELLGIPEERRAWLRANAMALQEASSSRAGRDVDGYARAEVA

AQEFTEYFREQVRLRRGRAGGDLITILANAQQRGAPVSLDAIVGTCVHLL

TAGHETTTNSLAKAVLALREHPAVLDELRGADGLTTDAVEEFLRYDPPVQ

AVTRWAHQDTTLGGYDIPRGSRVVALLGSANRDPARFPSPDVLDVRRPAD

RHLSFGLGIHYCLGATLARAELEIGLQALLDGVPTLGYGTQHVDYADDLV

FHGPSRLVLVNLGERCK

>CYP107AW7(2571109023)*Salinispora arenicola* CNH996B

VETVTGTSTPPPVPYIADPYPTLARIRANGPVSILHSDEGIPMWVIARYR

DVRAALADPRFGQDARRAQALADNRVAGVTLGGDIVHMLNSDPPDHTRLR

RHVQGAFTARRVAAMRPLVERITTSLLDGLTGRTTVDLVQDFAFPLPMLV

ICELLGFPAEERNAYRSWSTAILTHDDDPAAFATALREMTDYIAVQLRIR

QTQPGDDILTELLAARDAGQLTDDEIIGMVFLLLIGGHETTVNLLGTATL

ALMRNPDQHRWLLANQHALPEAIDEFLRYESPVAMATLRFTTTPVAVDDV

VIPAGELVLVSLGGANRDPDRFPDADRLILDRRDTGHLAFGHGLHRCLGA

FLGKLEGEVALGALLRRHPRLALAAEVRQLRWRDTIMLRGLESLPVSLHG

>CYP166A4(2571109828)*Salinispora arenicola* CNH996B

MTYAISFELPWARTDKFDPPAVFDALREQRPLARMRYPDGHVGWIVSSYE

LVREVLGDPRFSHSCAVGHFPVTHQGQVIPTHPQIPGMFIHMDPPEHTRY

RRLLTGEFTVRRTSRLTGHVEGVATEQIEVMREHGAPADLVATFARPLVL

RVLSGLVGLPYGERDRYLHAVTLLHDAEADPAEAAAAYEQAGAYFDEVIE

RRRRQPEDDLISTLVGDGELTGEELRNIVTLLLFAGYETTESALAVGMFA

LLYHEDQLARLRADPTKIDAAIEELLRYLTVNQYHTYRTASEDIELHGEV

INKGDSVTVSLPAANRDPARFACPADLDIDRETSGHVAFGFGIHQCLGQN

LARVELRAGLSALLRAFPNLRLAVPADEVPLRLQGSVFAVKNLPVCW

>CYP125A41(2571107809)*Salinispora arenicola* CNH996B

MTEPRIPAGFDFTDPEVLAHRVPREEFAELRRTAPVWWNAQPRGSAGFDD

DGYWVVTRYADVMTVSRDSDTYSTRENTAIARLRPDTTREDIEMQRVIML

NVDPPEHTKLRAIVSRGFTPRAINALRGSLAERAEHIVRDAAVRGVGDFV

TDVACELPLQAIAELIGVPQHHRRKVFDWSNQLIGYDDPAYGTDPLTASA

ELLAYAMEMAEERQRSPSDDLVTKLVNAQIDGEHLTTDEFGFFVMLLAVA

GNETTRNAITHGMVAFLDNPEQWELFKAERPKSAVEEIIRWATPVNVFQR

TALVDTVLGGQAISAGQRVALFYGSANFDEAVFEDPERFDITRSPNPHLG

FGGSGAHFCLGANLARLEIELIFNSIADHLPDIRKVAAPQRLRSGWINGI

RQMPVRYR

>CYP208A12(2571110450)*Salinispora arenicola* CNH996B

MTLDTITPRVPLGPPRTAALRMLLVMKRDRLGMLTSAAARYGDASRLPVG

HKALWFFNHPRYAKHVLADNSANYHKGIGLVHARRALGDGLLTSEGDLWR

KQRKVIQPAFQSRRIAQQAGMIAEEAFALVERLRARAGAGPVELTAELTG

LTLGVLGRSLLDADLAGFDSIGDSFATVQDQAMFELETLNAVPMWIPLPR

QIRFRRARRKLQAVVDTLVDGRAGNLANRVDVLSRLILSARGEADPRVGR

ERLRDELVTLLLAGHETTASTLGWTLSLIDRHPGVWERLHAEAVEVLGDR

LPEYDDLRRLRYTVMVVEEAMRLFPPVWLLPRRALAPDTIGEYRVPANAD

VVISPYTLHRHPEFWPNPERFDPERFAPGQAADRPRYAYLPFGAGPRFCV

GNNLGMMEAVFVIALLCRHLRLTGVPGYRLVPEPMLSLRIRGGLPLVVRP

VS

>CYP105BL2(2571110549)*Salinispora arenicola* CNH996B

MSSHSAAAPDPVTATPLHTLAPELTFPQFERSTPFDPPQAYTELSGRCPV

APVSMADGKPSWLITSFEGVRTTLSDPRFSSDMSHPGFPNRTGKPVDDLL

KDTLGAMDGERHRYYRRMLTGELTVRRAKAMRPVITRITDEALDQLAAAG

PGADLVKHVAFVVPSRVACHLVGIPLSDYELFTGMAATLMDSTSSDDQFA

ALQNMVSYFDTLVTDREHHDRDDLLGHMVRRYLATGELTRDMLIRLAWTT

MAAGQETTAHMIGLGVAALLRHPDQLELLRREPHLLPGAVDELMRYLPLI

QFGIPRVATDDVEVDGQTVTAGEGVVALPPLANRDPAVFERPDELDVRRN

ARQHLTFGYGPHQCPAHALARLELEVVYGRLLERFPTLRLADSDADLKVQ

DKDIMYRVSELAVTW

>CYP1005A1(2571110589)*Salinispora arenicola* CNH996B

MTAGTRWPDVTRVADQSGTEHLVVTRHALVRQVLTDQATYRPDNALEAVT

PVPVAALRVLAGHRFRLPPTLANNGGVSHPAIRALVADALHPTKVAAQRP

WLTGLVADRVATIRTTLDSGGPVDLYADLTADLPLLVLARLVELPDAPVN

AVKQFARAALELFWAPLDADRQLALADEVGRFHQVLREFADTGGGLAAAL

RATGHSPDVLVGALFFLLVAGQETTSQFLTLLLHRLSGEPTIRAALRAGS

ISVADVVEEGLRLEPPIVTWRRVAAVDSTLGGTTVAAGTSVLLWLARAGR

DPAVVAAPDEFRPGQRGSRRHLAFGAGAHRCLGDQLARMEAAVVVEQATP

LLDGVTVVRPPWYPDNLTFRMPDAFVVRR

>CYP107AY2(2571110692)*Salinispora arenicola* CNH996B

MTAEPTPIPRSGTRLGPEYDQLRKTGDVHQVLLPDASLAWLVTNPEVAAR

ALADPRLALNRRNSRGGWSGFALPPALDANLLNLDAPDHTRLRRLVGPAF

SRQRVAALRPGIRRAAEHLLDTLVATSGPTDLVTGYCNPLSVQVIADLMG

VPEAGRTNLRAWTDTMLTSYPPDRDAIRQAVTELHGYVVDLIDIKQQQPG

DDLLSALVTIEQDGDRLSRDELTSLAFLILFAGYENTANLIASAVRWLLD

HGGLNVVPISEAIEGTLRHEPPAPVAIRRFPTEDIIIGGVTIPAGDTVLL

SIAAATRGADGNAARLAFGNGPHYCLGAALARVEAEEALTVLARRLPGLT

LAVPPAQVRWRPTFRTHGPAELLVGW

>CYP163B21(2571110985)*Salinispora arenicola* CNH996B

MNLSLDLTDPATFVENDPHEFWRQVRREQPTYWHPSTAGRPGFWVVAGYE

EVRACYGNWKQLSSARGNVLDVLLHGGDSAGGKMLAVTDRPRHRHLRNLM

LRAFSPRVMGEVVRKVEERAAELIRTVTERGSFDFATEVAEHIPMNTICD

LLSIPPNDRKQLLVWTKAALSSADPQSDTLDALEARNEIVLYLMDLAFER

RSTPGNDVISMIAGAEVDGRPLTPEEVALNCYSLILGGDESSRITAISSV

LAFTEYPDQWHALREGTVSVETTTEELLRWATPGMHFARTALCDLSIQGQ

RIQTGDIVTFWNTSANNDETVFAAPRRLDLSRDPNKHLTLGHGPHFCVGA

ALGRAELGALLRALIASVGEIEIHGAPQRIYSNLLHGYSSLPVTFHPLST

GKAT

>CYP154M5(2571111264)*Salinispora arenicola* CNH996B

VEQSCPYKLDVTGRDVHAEGEAIRARGPVAQVELPGGVQGWSVTGYQAAR

QVLADPRFAKDPKKWPAYTSGAIPPNWPLIGWLLMDNMTTNDGADHQRLR

KLVSHGFTPRQVERTRPLIVKIVNDLLDGLSSAGPDEVVDLKGRFATPLP

ARVICDMFGVPEALRASVLRGAQVNVTSSISGEEAEANVEQWHRELLELV

EAKREKPDEDMASLLIAAKEEDGSTLTQEEVVGTLHLMLGAGSETLMNAL

SYAVLGMLSNPGQYEMVRNGTSSWDDVIEETLRAQAPVAQLPLRYATEDV

AVGGAVIKAGDPVLMGFTAIGRDPAVHGETAGDYDITREDKTHLSFGHGV

HFCLGAPLARLELKIALPALFERFPNMTLAVRPDQLEPQGTFIMNGHREL

PVRLGQPATVLA

>CYP105CT1(2571111281)*Salinispora arenicola* CNH996B

MNSPNHMPADRSLTAPASGCPMALSRGRVGLDVADEISELRDGGRLGRIT

TAFGQEATLITRYDEVRAQMADSVVFNVAGVPSPPALVDGGFDTESVRRR

RTVGNLIMLDPPEHTRLRRMVAAWFTTRRVERLRPRVVEIIDAALDEMER

SGPPVDLVAMFAKTVPITVICELIGVPEELRERYRRRAERAVSASAVSTP

LDELRRLREAGWVSRELIEYHRENPSDDIIGMLLREHGTDSHDDGITDDE

LVGLANALLIAGHETTTQMLSMGTLALLRHPDQLALLRDDPSIVAGAVEE

LLRYVGVLHGGFVRVATRDTRLGGHRIHAGELVVPALTAANRDPRLLTDG

DRLDITRPPTSHVAFGHGVHFCIGAPLARMELREAFPALLRRFPGLRLAV

PDSELEFTQGTTVYSLRGLPVTW

>CYP105G5(2571111362)*Salinispora arenicola* CNH996B

LTIETTETPAADDSLRAPLPRQFMQRDDPSKLPPALAALAEQSPVGRSTL

PDGDPFWMVSGYDEARAVLSDPRFSSDRFRYHPRFKKLSGQLGERLRNDK

ARAGSFINMDPPEHTRYRKLLTGQFTVRRMRQLTVRIEQIVTEQLDVMLA

EGNSADLVSAFAVPVPSLMICELLGVRYEDRTEFQRRAAGLLQTDLPIKQ

AVENLEAQRAFMQRLVTDKRTTPADDMISGLVHHAGAEPPLTDDELVGIA

TLLLFAGLDTTASMLGLGMFMLLQRPEQMAVLRDDPSRIGDAVEELLRYL

TVVSTGLFRFAKEDVVLGDEHIPAGSTVVVSLMAANRDGRHWPEPETLDV

TRVRSSHLAFGHGVHQCLGQQLARIELTVGITELLCRLPNVRLAVPPADV

PLRNDMITYGVHRLPILWGTP

>CYP107Q4(2571111363)*Salinispora arenicola* CNH996B

MTTTAETSAETIDLFSPEVVADPFGWYARLREETGPNTGTLNIGTMMGGP

EMWLVTRYEDVRQVLTDPRFLTNPPADSPLEDIRAGVFRRLDFPPDLIPW

MANLLNVSDGEDHTRLRKLVSYALTAHRIGKLRPRVEKITADLLDKLAED

GKDGSPVDLVEEYCYPLPVTVICELVGIDEPDRPHWRAWGDSMATMNGER

IPTTLVKCIELARELIAKRRAEPQDDLVTALVQAQAEDQNRVSDDEIIGI

LFSLVTAGHQTTTYLIGNSVILLLENPDQLARLKENPSMWPQAVRELQRL

GPIQFAQPRFPSEDIELGGVTIPRGAPVAPLLLAANTDPRRFPDPNKLII

DRLAVGSEGHLGFGKGIHRCLGQHLAYQEAEVALQGLFTRFPDLTLAVPR

EEIPWILRPGFTRTRTLPLKLV

>CYP105AB36(2571111465)*Salinispora arenicola* CNH996B

MTEAASSRLTDTEFPVQRQCPFAEPVEYEQIREQSSIAMVRLTGGGEAWW

ISGHEQGRAVLADRRFSSDRRKANFPFVSTDPATRKRLHAQPLSMISMDG

AEHAQARRALIGEFTVRRLAALRPRIQQIVDQCIDEMLTTDQHCVDLVKA

LSLPVPSLVICELLGVPYDDHDFFQEHTATLVRRNTAPEVRQHCVDELNA

YLGALIDRKLANPDDDLLGRQIARQHQDGTFDRASIVSLAFLLLVAGHET

TANMISLGVVGLLQHPDQLTMIKKDPDKVPLAIEELLRFFTIVDSVTSRV

ATEDVRFGDTTINAGDGVVVSGLSADWDPTVFADPDRLDLERGARHHIAF

GFGPHQCIGQNLARLELQIVFDTLFRRIPTLRLAAPLDKIPFKTDAAIYG

ARELPVAW

>CYP105CP2(2571111611)*Salinispora arenicola* CNH996B

MTKSMPVQDLPAFPIPRECPYRPSAQHVSLRSGGPMAKVRLYNGRTAWLV

TDSAHARAVLSDYRRVSIKPYHGNYPLLNEEFEKVVDSGYADVLFGVDPP

EHTRQRQMIMPSFTLRRTAVLRPDIQRIVDDKLDEMMRHGAPGDLVTEFA

QPVPSMVMSFLLGVPWEDHEEFETPAHKLFVPELAEEATTELGAYLERLI

QKKEQPGGTPGGTGLLDDLIRDHLRAGALSRDELVHIAMAMLVAGTDTTT

NVISLGTLALLDNPDQWAALRDNPDELIPGAVEEILRYTSLIEAFARVAV

SDIELNGAVIKEGEGILISSAGVNFDPALAPDPGRFDIRRPPRPSFSFSH

GIHRCPGDNLARLELEIAFRSLVTRMPNLRTATPIDQIPSNNNDGTLQRL

YELPVVW

>CYP105CN1(2571111613)*Salinispora arenicola* CNH996B

MAAPAPQATQSTTPHPPSYPLPRECPYRPSAGTARLRDAGPVSTVRLYDG

RTAWLVTGAAEARALLADSRVSNRADFPNYPVMDERHLSMRATREMAREE

EGGFAAALFGMDPPEHTRQRQLLLPRFTVRQVAARRPAIQRIVDEHLDAM

EANGSPADLVSAFATPVPTMVVCTHLGVPYQDRTRFEPAVAGLFEPDRAD

AAMAELTAYLHQLIETRQSEPGDGVIDHLIANHLRPGAIDRAELVAIASA

ILVAGTVTTSSAIALGTLALLTAPGQYAALVDNPDLVPGAVNEILRYLSL

VEQLARVATEDIEIGGKLIRAGDGIIVSFAAGNLDPNVTTHPDRLDVALP

PTNHLAFSHGIHHCIGQNLALLELDIAFRALVSRFPTLRLAVPAEQLPTY

FAGDVPRLACLPVTW

>CYP107FS2(2571111614)*Salinispora arenicola* CNH996B

MPVPQGEQNLTTEVFADPKALFATLGSRQPLHRISLPDGMPAVLVTGNRE

ARQALSDPRLVRSITAAAPELHKYHPLASDDYALSRHMLFADPPDHGRMR

KLVSTAFTRRRVEQMRPRIQQITDDLIDVIAAKGEADLVETLALPLPIAV

ISEMLGVPFADRSEFERHAEVLTGINASSGFDAIIAAGRWFDEYLAGLVQ

QRRREPQDDLISGMLAAQDKGDRLTDVELRSNALLLLSAGFETTVNLVAN

GLLALLRHPEAMAALRSEPNLMTTAVDELLRYDSPVSCVTYHFAQEPVEI

GGFEIRSGEHVVIAAAAANHDPTVFADPSRLDLRREGSGQILSFSHGIHF

CLGAPLARLEGEIAFGTVLRRLAGLRLAVPTDSLVWKASFVLHRLERLPV

TFTPDRAPNPIDSVHTV

>CYP247A7(2571111746)*Salinispora arenicola* CNH996B

VRLTPGAARDIDLDSVNLFDLDLYTSGDPHPIWDVMRAQSPLHHQVLADG

REFWSVTRYDDVCRVLGDYREFTSERGTVVTHLGEDDIAAGKLLTSTDPP

RHTQVRRAIGAKLTARAVASWQDRIRDAIVRFLEPALDGDTFDLAEQALL

LPAIVTGPLLGIPERDWQELVQLTAMVTAPSDPHFQHGSEAATLAISHHE

LVTYVTEWVKQRRSAGGGDGSLLDHLMSVRVGGAPLTDEEIALDGYSILL

GANVTTPHTVSGTVLALIERPEQFEKAQADPSLLANLVEEGLRWTSAACN

FMRYALNDTRIGGGTVPAGGAVVAWIGSANRDESYFPDPHQFDITRSGAN

RQVAFGFGPHYCIGAPLARMTLGIFFEELVQRFGSIELAGEPQHLRSYFI

AGMTHLPIVAQKRKTP

>CYP248A2(2571112055)*Salinispora arenicola* CNH996B

VLADAVTAFDPTAVDVRRDPYPSYHWLLRHDPVHRGAHQVWYVSRFADVR

AVLGDERFARTGIRRFWTDLVGPGLLSQIVGDIILFQDEPDHGRLRGVVG

PAFSPSALRRLEPTIEATVNDLLRPARALGAMDVVADLAYPLALRAVLEL

LGLPAGDANAVGRWSRAVGRTLDRGATAEDMRRGHAAITEFADYVERALA

ERREDGADLLALMLAAHRSQLMSRNEIVSTVVTFIFTGHETVASQLGNGL

LSLLDHPEQLELVRRQPHLVPQAVEECLRFDPAVQSNTRQLAADVELHGR

RLRRDDVVVVLAGAANRDPGRYDRPDELDIRRDPVPSMSFGAGMRYCLGS

YLARLQLRTALGAMVALPDLRLVCNPNELAYQPRTMFRGLTRLPVAFTPA

G

>CYP105W2(2571112063)*Salinispora arenicola* CNH996B

MTGYQDRPTGDQPGAPVPSGSTDPGIGAFPLPRRCPFSPPAEYARLRAEH

PVVRLPMLGGDTAWVVSRHADVRQVLSDPRMSADRRRPGFPKFAPTTEGQ

RQASFANFRPPLNWLDPPEHAICRRQIVDEFSVRRVRQSRALVERVVDTH

LDALTAGAPGADLVSTFAYPVPSQVICEVLGVPYGEHEFFERRSTLMFRR

STPADERARYAREIRDFLDMVVTDKERRPGDDVLSRLLYRQRSAGGMDHE

AVVSMAFVLLVAGHVTTSNMLALSVLALLTHPARLARLRAEPERFPAAVE

ELLRYFTVVEAATARTTTAEIMIGGVTIAAGEGVVALGQAANRDPRVFEH

PDEFDPDRDARAHLAFGHGRHICPGQHLARLEMEVALSRLFRRLPGLRLT

MEVSDLPLKEDSNIFGLYALPVAW

>CYP107NH1(2571112462)*Salinispora arenicola* CNH996B

MTEPVVSLMDPEFWNDPVSAYERLRGSGPLIRMGLPGVPPVWLVTSCEHV

KSALSDPRFVVDAANVPGHHGPGIVDQMMAASGMPDEFRDYMTNMMFTDG

KDHSRLRRLVTPGFSARRIRAMRPRVDQIAEELVESLAEKGSGELIADFS

TPLTTTVICELIGVDRADQAQMGAWMHDYTTGERVVSGRAMVNYTRDLIE

RRRAEPADDMISAMIRSGDEAGDRLSDAEIIAMALLLINAGHHSTAQFIP

NAVLVLLDHPEQLARLRAEPGQLPGAMDELMRLANPVPIATPRYATEDME

FAGVAVRRGEALTGSLEAANFDPERFPAPRQLDTGRDLGRGDGHLSFGAG

PHYCPGAALARLEGEIALDHLLLRRDSLRLAVERDEVDYVDVSLGLRMLS

SLPVRL

>CYP247A8(2571112467)*Salinispora arenicola* CNH996B

VRLTPEAGRPVALGSIDLFDPDLFASGDPHSVWDVMRSEAPLHRQVLPDG

RVFWSVTRYEDVCRVLGDHRAFTSERGTVFTQLGADDIAAGKLLTSTDPP

RHTEVRRAIGGRLTARAVAHWQDLVRRAVVRFLEPALDGGCWDLAERAQQ

LPLIIAGSLLGIPDSDWEKLVQLTGMVTAPSDSMFGLGSEAATLAIAHHE

IFDYLSDLVRRRRSAGTGGDSLLDHLMTVRAGAGPLPDEEVVYDSYSLIL

GANATTPHTLSGIVLTLVERPEQCDKVRADPSLIPSMVEEGLRWTSAACN

FMRYATVDTRLTGGTVPAGEAVVAWIASANRDESQFADPHTFDVTRGENR

HVAFGFGPHYCIGAPLARMTARIFFEELFRRFWSIEIDGEPQHLRSNFIA

GMTHLPVVTRKRAQV

>CYP1051A1(2571112745)*Salinispora arenicola* CNH996B

MATDAPITRARTVPAWKALPAAVRDTHRALVDVGNWSDGDVVRVSLGVSR

PYLVTNPAHVQEVLHERAAIYPRGDDTALWRSVRKLVGDGILAEGDAWAA

SRRVLAPMFRPARINAMVDTMADAIAGAVDDLHEAATAGTPIDVGRELSR

IVCSAIMRVFFADRITVRDALRIMKAQETIVTAMAPRILAPLVPWWIPMP

GDRRFRAAVRSIDDILLPVLRQAQRQPDDGDDLLSRLVRARADDGRALSE

KRMRDDLVSMVAVTTETSTVVLTWLWPLLANHPDVANRLYDEIDRVVGGG

PVRGDHLAELTYTRMVLDELLRLYPAGWILPRRAATTDVLGGVRINKGAT

VILSPYVTQRMTAWWGPTAEAFDPERFAAGREAADGRHRYAYYPFGVGMH

RCLGEHLFNLEAILIVATLLSRFRFALTDTSMPGVKVAASTRPARTVEMI

LKPVAPVPAR

>CYP211C1(2562074143)*Salinispora arenicola* CNH996

VVDVEELLTRLYSAQGRQDPFPVYADLHAQGPIAALPPEPERRRVAAVAV

GYDLVGAVLRDPEWSKAPPPGWTEQEILRTLQTSMMFINPPDHGRMRHVF

AGTFTPRRLGALEPVVNRVADELLDRMADAGAGGLDFVAEFAYPLPARVM

AEFIGIPETELDWYRERVDVIDAFLDVAGKTPQRLAAANAAGAELRAFYG

ELLAHRRRTPGEDLISGLLEAVDAGGVELTEDELVSNLIVLFNASFVTTV

YMLSNGLPVLLAHPEVAAALATDPVLTAGAVDEILRLQAPVHLLARAAPR

DTVLGGVPIPQGQNVLLLIAAANRDPAHFPDPDRFDPRRSGPPSLAFGLG

LHYCLGAAVSRLEGRLALPRLLSRFPRLRIMEQPVYSGSLFLRGIDKLSV

SPGEGSTRE

>CYP125A41(2562074292)*Salinispora arenicola* CNH996

MTEPRIPAGFDFTDPEVLAHRVPREEFAELRRTAPVWWNAQPRGSAGFDD

DGYWVVTRYADVMTVSRDSDTYSTRENTAIARLRPDTTREDIEMQRVIML

NVDPPEHTKLRAIVSRGFTPRAINALRGSLAERAEHIVRDAAVRGVGDFV

TDVACELPLQAIAELIGVPQHHRRKVFDWSNQLIGYDDPAYGTDPLTASA

ELLAYAMEMAEERQRSPSDDLVTKLVNAQIDGEHLTTDEFGFFVMLLAVA

GNETTRNAITHGMVAFLDNPEQWELFKAERPKSAVEEIIRWATPVNVFQR

TALVDTVLGGQAISAGQRVALFYGSANFDEAVFEDPERFDITRSPNPHLG

FGGSGAHFCLGANLARLEIELIFNSIADHLPDIRKVAAPQRLRSGWINGI

RQMPVRYR

>CYP211B2(2562074960)*Salinispora arenicola* CNH996

MDVSEAVAVLISPSGRLDPYPTYEQLRAHGPVSRTTAGLFVVTGYAEADM

VLRDPRFVVLDDDLRDDVFPHWQDSPAIKSIARSMLRTNPPDHSRIRRLA

AGAFTPRRVAAMREVVTAQADELVDEMIRAGRDGARVDFMDMFAYPLPVA

VICALLGVPAADRSRFRRWAGDLTGILEPEITPEELAGADAGADELRDYF

TGLIEQRRRAPADDLTTALVQAHDADGDRLSGEELLANLVVLLVAGFETT

TNLLGNGLVVLLTRPEAAAALRDEPDLAPGYVDELLRYDSPVQLTTRTVR

ESVSFAGTELPAGSWLLVLLGAANRDPRRFPDPARFDPWRAQSQPLSFGA

GPHYCLGAGLARLEAQVAFPLLLRRLPELALAGRPSRRTRLTLRGYETLP

ITVGAVTADRGTPAGVAPGTP

>CYP166A4(2562075386)*Salinispora arenicola* CNH996

MTYAISFELPWARTDKFDPPAVFDALREQRPLARMRYPDGHVGWIVSSYE

LVREVLGDPRFSHSCAVGHFPVTHQGQVIPTHPQIPGMFIHMDPPEHTRY

RRLLTGEFTVRRTSRLTGHVEGVATEQIEVMREHGAPADLVATFARPLVL

RVLSGLVGLPYGERDRYLHAVTLLHDAEADPAEAAAAYEQAGAYFDEVIE

RRRRQPEDDLISTLVGDGELTGEELRNIVTLLLFAGYETTESALAVGMFA

LLYHEDQLARLRADPTKIDAAIEELLRYLTVNQYHTYRTASEDIELHGEV

INKGDSVTVSLPAANRDPARFACPADLDIDRETSGHVAFGFGIHQCLGQN

LARVELRAGLSALLRAFPNLRLAVPADEVPLRLQGSVFAVKNLPVCW

>CYP244A4(2562075503)*Salinispora arenicola* CNH996

MSTTTNTELTEAPETNMPVDPGLFDCMPDLIAAARVAPVVRIPYLGRHAW

VVCDRELVKQALTHPKMGKDIALVPEWMRQPGLMVTAQPDPEYARAMIMS

DGENHARIRRIHAPVLSPRNTERWGERVADKVEGFLDELSQAGSGGSTEV

NVVTNYTHKIPLAFISEMLGLPPEAEHRLRGITDIMLYSSDYAARREAIG

GLFGAVEDWVRNPADLRDGVITGLLAASDGPDAAVTEGEVIVWTLGMIIT

GYETTGSLISTSLYEAIRRPPHERPKTDEDITAWIEETLRVHPPFPHPTW

RFPLEDIELGGYLIPKGAPVQVSIAAANRKPGEGADSFDAERRGHGHLSF

GLGMHYCIGAPLVRLEAQIAVRGFLRRFPQARLSAETAVQWESEWMIRRM

SVLPAVLS

>CYP245A7(2562075507)*Salinispora arenicola* CNH996

MPSATLPRFALTGWSRENIVNPYPVYQRYREVASVHRGEPGGDAPDTFYV

FSYDEVVQVLSSNCFGRGRSLDAAKASVPVPAEQKALRAIVENWLVFMDP

PRHTELRSLLNRSFSPRIVTELRPRIARIAQELLSRLGQQVDVDLVESFA

APLPILVISELLGIPEERRAWLRANAMALQEASSSRAGRDVDGYARAEVA

AQEFTEYFREQVRLRRGRAGGDLITILANAQQRGAPVSLDAIVGTCVHLL

TAGHETTTNSLAKAVLALREHPAVLDELRGADGLTTDAVEEFLRYDPPVQ

AVTRWAHQDTTLGGYDIPRGSRVVALLGSANRDPARFPSPDVLDVRRPAD

RHLSFGLGIHYCLGATLARAELEIGLQALLDGVPTLGYGTQHVDYADDLV

FHGPSRLVLVNLGERCK

>CYP107AW7(2562075735)*Salinispora arenicola* CNH996

VETVTGTSTPPPVPYIADPYPTLARIRANGPVSILHSDEGIPMWVIARYR

DVRAALADPRFGQDARRAQALADNRVAGVTLGGDIVHMLNSDPPDHTRLR

RHVQGAFTARRVAAMRPLVERITTSLLDGLTGRTTVDLVQDFAFPLPMLV

ICELLGFPAEERNAYRSWSTAILTHDDDPAAFATALREMTDYIAVQLRIR

QTQPGDDILTELLAARDAGQLTDDEIIGMVFLLLIGGHETTVNLLGTATL

ALMRNPDQHRWLLANQHALPEAIDEFLRYESPVAMATLRFTTTPVAVDDV

VIPAGELVLVSLGGANRDPDRFPDADRLILDRRDTGHLAFGHGLHRCLGA

FLGKLEGEVALGALLRRHPRLALAAEVRQLRWRDTIMLRGLESLPVSLHG

>CYP105BL2(2562076171)*Salinispora arenicola* CNH996

MSSHSAAAPDPVTATPLHTLAPELTFPQFERSTPFDPPQAYTELSGRCPV

APVSMADGKPSWLITSFEGVRTTLSDPRFSSDMSHPGFPNRTGKPVDDLL

KDTLGAMDGERHRYYRRMLTGELTVRRAKAMRPVITRITDEALDQLAAAG

PGADLVKHVAFVVPSRVACHLVGIPLSDYELFTGMAATLMDSTSSDDQFA

ALQNMVSYFDTLVTDREHHDRDDLLGHMVRRYLATGELTRDMLIRLAWTT

MAAGQETTAHMIGLGVAALLRHPDQLELLRREPHLLPGAVDELMRYLPLI

QFGIPRVATDDVEVDGQTVTAGEGVVALPPLANRDPAVFERPDELDVRRN

ARQHLTFGYGPHQCPAHALARLELEVVYGRLLERFPTLRLADSDADLKVQ

DKDIMYRVSELAVTW

>CYP208A12(2562076896)*Salinispora arenicola* CNH996

MTLDTITPRVPLGPPRTAALRMLLVMKRDRLGMLTSAAARYGDASRLPVG

HKALWFFNHPRYAKHVLADNSANYHKGIGLVHARRALGDGLLTSEGDLWR

KQRKVIQPAFQSRRIAQQAGMIAEEAFALVERLRARAGAGPVELTAELTG

LTLGVLGRSLLDADLAGFDSIGDSFATVQDQAMFELETLNAVPMWIPLPR

QIRFRRARRKLQAVVDTLVDGRAGNLANRVDVLSRLILSARGEADPRVGR

ERLRDELVTLLLAGHETTASTLGWTLSLIDRHPGVWERLHAEAVEVLGDR

LPEYDDLRRLRYTVMVVEEAMRLFPPVWLLPRRALAPDTIGEYRVPANAD

VVISPYTLHRHPEFWPNPERFDPERFAPGQAADRPRYAYLPFGAGPRFCV

GNNLGMMEAVFVIALLCRHLRLTGVPGYRLVPEPMLSLRIRGGLPLVVRP

VS

>CYP1005A1(2562076978)*Salinispora arenicola* CNH996

MTAGTRWPDVTRVADQSGTEHLVVTRHALVRQVLTDQATYRPDNALEAVT

PVPVAALRVLAGHRFRLPPTLANNGGVSHPAIRALVADALHPTKVAAQRP

WLTGLVADRVATIRTTLDSGGPVDLYADLTADLPLLVLARLVELPDAPVN

AVKQFARAALELFWAPLDADRQLALADEVGRFHQVLREFADTGGGLAAAL

RATGHSPDVLVGALFFLLVAGQETTSQFLTLLLHRLSGEPTIRAALRAGS

ISVADVVEEGLRLEPPIVTWRRVAAVDSTLGGTTVAAGTSVLLWLARAGR

DPAVVAAPDEFRPGQRGSRRHLAFGAGAHRCLGDQLARMEAAVVVEQATP

LLDGVTVVRPPWYPDNLTFRMPDAFVVRR

>CYP163B21(2562077256)*Salinispora arenicola* CNH996

MNLSLDLTDPATFVENDPHEFWRQVRREQPTYWHPSTAGRPGFWVVAGYE

EVRACYGNWKQLSSARGNVLDVLLHGGDSAGGKMLAVTDRPRHRHLRNLM

LRAFSPRVMGEVVRKVEERAAELIRTVTERGSFDFATEVAEHIPMNTICD

LLSIPPNDRKQLLVWTKAALSSADPQSDTLDALEARNEIVLYLMDLAFER

RSTPGNDVISMIAGAEVDGRPLTPEEVALNCYSLILGGDESSRITAISSV

LAFTEYPDQWHALREGTVSVETTTEELLRWATPGMHFARTALCDLSIQGQ

RIQTGDIVTFWNTSANNDETVFAAPRRLDLSRDPNKHLTLGHGPHFCVGA

ALGRAELGALLRALIASVGEIEIHGAPQRIYSNLLHGYSSLPVTFHPLST

GKAT

>CYP154M5(2562077577)*Salinispora arenicola* CNH996

VEQSCPYKLDVTGRDVHAEGEAIRARGPVAQVELPGGVQGWSVTGYQAAR

QVLADPRFAKDPKKWPAYTSGAIPPNWPLIGWLLMDNMTTNDGADHQRLR

KLVSHGFTPRQVERTRPLIVKIVNDLLDGLSSAGPDEVVDLKGRFATPLP

ARVICDMFGVPEALRASVLRGAQVNVTSSISGEEAEANVEQWHRELLELV

EAKREKPDEDMASLLIAAKEEDGSTLTQEEVVGTLHLMLGAGSETLMNAL

SYAVLGMLSNPGQYEMVRNGTSSWDDVIEETLRAQAPVAQLPLRYATEDV

AVGGAVIKAGDPVLMGFTAIGRDPAVHGETAGDYDITREDKTHLSFGHGV

HFCLGAPLARLELKIALPALFERFPNMTLAVRPDQLEPQGTFIMNGHREL

PVRLGQPATVLA

>CYP105CT1(2562077594)*Salinispora arenicola* CNH996

MNSPNHMPADRSLTAPASGCPMALSRGRVGLDVADEISELRDGGRLGRIT

TAFGQEATLITRYDEVRAQMADSVVFNVAGVPSPPALVDGGFDTESVRRR

RTVGNLIMLDPPEHTRLRRMVAAWFTTRRVERLRPRVVEIIDAALDEMER

SGPPVDLVAMFAKTVPITVICELIGVPEELRERYRRRAERAVSASAVSTP

LDELRRLREAGWVSRELIEYHRENPSDDIIGMLLREHGTDSHDDGITDDE

LVGLANALLIAGHETTTQMLSMGTLALLRHPDQLALLRDDPSIVAGAVEE

LLRYVGVLHGGFVRVATRDTRLGGHRIHAGELVVPALTAANRDPRLLTDG

DRLDITRPPTSHVAFGHGVHFCIGAPLARMELREAFPALLRRFPGLRLAV

PDSELEFTQGTTVYSLRGLPVTW

>CYP105G5(2562077675)*Salinispora arenicola* CNH996

LTIETTETPAADDSLRAPLPRQFMQRDDPSKLPPALAALAEQSPVGRSTL

PDGDPFWMVSGYDEARAVLSDPRFSSDRFRYHPRFKKLSGQLGERLRNDK

ARAGSFINMDPPEHTRYRKLLTGQFTVRRMRQLTVRIEQIVTEQLDVMLA

EGNSADLVSAFAVPVPSLMICELLGVRYEDRTEFQRRAAGLLQTDLPIKQ

AVENLEAQRAFMQRLVTDKRTTPADDMISGLVHHAGAEPPLTDDELVGIA

TLLLFAGLDTTASMLGLGMFMLLQRPEQMAVLRDDPSRIGDAVEELLRYL

TVVSTGLFRFAKEDVVLGDEHIPAGSTVVVSLMAANRDGRHWPEPETLDV

TRVRSSHLAFGHGVHQCLGQQLARIELTVGITELLCRLPNVRLAVPPADV

PLRNDMITYGVHRLPILWGTP

>CYP107Q4(2562077676)*Salinispora arenicola* CNH996

MTTTAETSAETIDLFSPEVVADPFGWYARLREETGPNTGTLNIGTMMGGP

EMWLVTRYEDVRQVLTDPRFLTNPPADSPLEDIRAGVFRRLDFPPDLIPW

MANLLNVSDGEDHTRLRKLVSYALTAHRIGKLRPRVEKITADLLDKLAED

GKDGSPVDLVEEYCYPLPVTVICELVGIDEPDRPHWRAWGDSMATMNGER

IPTTLVKCIELARELIAKRRAEPQDDLVTALVQAQAEDQNRVSDDEIIGI

LFSLVTAGHQTTTYLIGNSVILLLENPDQLARLKENPSMWPQAVRELQRL

GPIQFAQPRFPSEDIELGGVTIPRGAPVAPLLLAANTDPRRFPDPNKLII

DRLAVGSEGHLGFGKGIHRCLGQHLAYQEAEVALQGLFTRFPDLTLAVPR

EEIPWILRPGFTRTRTLPLKLV

>CYP107AY2(2562077701)*Salinispora arenicola* CNH996

MTAEPTPIPRSGTRLGPEYDQLRKTGDVHQVLLPDASLAWLVTNPEVAAR

ALADPRLALNRRNSRGGWSGFALPPALDANLLNLDAPDHTRLRRLVGPAF

SRQRVAALRPGIRRAAEHLLDTLVATSGPTDLVTGYCNPLSVQVIADLMG

VPEAGRTNLRAWTDTMLTSYPPDRDAIRQAVTELHGYVVDLIDIKQQQPG

DDLLSALVTIEQDGDRLSRDELTSLAFLILFAGYENTANLIASAVRWLLD

HGGLNVVPISEAIEGTLRHEPPAPVAIRRFPTEDIIIGGVTIPAGDTVLL

SIAAATRGADGNAARLAFGNGPHYCLGAALARVEAEEALTVLARRLPGLT

LAVPPAQVRWRPTFRTHGPAELLVGW

>CYP105AB36(2562077817)*Salinispora arenicola* CNH996

MTEAASSRLTDTEFPVQRQCPFAEPVEYEQIREQSSIAMVRLTGGGEAWW

ISGHEQGRAVLADRRFSSDRRKANFPFVSTDPATRKRLHAQPLSMISMDG

AEHAQARRALIGEFTVRRLAALRPRIQQIVDQCIDEMLTTDQHCVDLVKA

LSLPVPSLVICELLGVPYDDHDFFQEHTATLVRRNTAPEVRQHCVDELNA

YLGALIDRKLANPDDDLLGRQIARQHQDGTFDRASIVSLAFLLLVAGHET

TANMISLGVVGLLQHPDQLTMIKKDPDKVPLAIEELLRFFTIVDSVTSRV

ATEDVRFGDTTINAGDGVVVSGLSADWDPTVFADPDRLDLERGARHHIAF

GFGPHQCIGQNLARLELQIVFDTLFRRIPTLRLAAPLDKIPFKTDAAIYG

ARELPVAW

>CYP107FS2(2562077871)*Salinispora arenicola* CNH996

MPVPQGEQNLTTEVFADPKALFATLGSRQPLHRISLPDGMPAVLVTGNRE

ARQALSDPRLVRSITAAAPELHKYHPLASDDYALSRHMLFADPPDHGRMR

KLVSTAFTRRRVEQMRPRIQQITDDLIDVIAAKGEADLVETLALPLPIAV

ISEMLGVPFADRSEFERHAEVLTGINASSGFDAIIAAGRWFDEYLAGLVQ

QRRREPQDDLISGMLAAQDKGDRLTDVELRSNALLLLSAGFETTVNLVAN

GLLALLRHPEAMAALRSEPNLMTTAVDELLRYDSPVSCVTYHFAQEPVEI

GGFEIRSGEHVVIAAAAANHDPTVFADPSRLDLRREGSGQILSFSHGIHF

CLGAPLARLEGEIAFGTVLRRLAGLRLAVPTDSLVWKASFVLHRLERLPV

TFTPDRAPNPIDSVHTV

>CYP105CN1(2562077872)*Salinispora arenicola* CNH996

MAAPAPQATQSTTPHPPSYPLPRECPYRPSAGTARLRDAGPVSTVRLYDG

RTAWLVTGAAEARALLADSRVSNRADFPNYPVMDERHLSMRATREMAREE

EGGFAAALFGMDPPEHTRQRQLLLPRFTVRQVAARRPAIQRIVDEHLDAM

EANGSPADLVSAFATPVPTMVVCTHLGVPYQDRTRFEPAVAGLFEPDRAD

AAMAELTAYLHQLIETRQSEPGDGVIDHLIANHLRPGAIDRAELVAIASA

ILVAGTVTTSSAIALGTLALLTAPGQYAALVDNPDLVPGAVNEILRYLSL

VEQLARVATEDIEIGGKLIRAGDGIIVSFAAGNLDPNVTTHPDRLDVALP

PTNHLAFSHGIHHCIGQNLALLELDIAFRALVSRFPTLRLAVPAEQLPTY

FAGDVPRLACLPVTW

>CYP105CP2(2562077874)*Salinispora arenicola* CNH996

MTKSMPVQDLPAFPIPRECPYRPSAQHVSLRSGGPMAKVRLYNGRTAWLV

TDSAHARAVLSDYRRVSIKPYHGNYPLLNEEFEKVVDSGYADVLFGVDPP

EHTRQRQMIMPSFTLRRTAVLRPDIQRIVDDKLDEMMRHGAPGDLVTEFA

QPVPSMVMSFLLGVPWEDHEEFETPAHKLFVPELAEEATTELGAYLERLI

QKKEQPGGTPGGTGLLDDLIRDHLRAGALSRDELVHIAMAMLVAGTDTTT

NVISLGTLALLDNPDQWAALRDNPDELIPGAVEEILRYTSLIEAFARVAV

SDIELNGAVIKEGEGILISSAGVNFDPALAPDPGRFDIRRPPRPSFSFSH

GIHRCPGDNLARLELEIAFRSLVTRMPNLRTATPIDQIPSNNNDGTLQRL

YELPVVW

>CYP247A7(2562078127)*Salinispora arenicola* CNH996

VRLTPGAARDIDLDSVNLFDLDLYTSGDPHPIWDVMRAQSPLHHQVLADG

REFWSVTRYDDVCRVLGDYREFTSERGTVVTHLGEDDIAAGKLLTSTDPP

RHTQVRRAIGAKLTARAVASWQDRIRDAIVRFLEPALDGDTFDLAEQALL

LPAIVTGPLLGIPERDWQELVQLTAMVTAPSDPHFQHGSEAATLAISHHE

LVTYVTEWVKQRRSAGGGDGSLLDHLMSVRVGGAPLTDEEIALDGYSILL

GANVTTPHTVSGTVLALIERPEQFEKAQADPSLLANLVEEGLRWTSAACN

FMRYALNDTRIGGGTVPAGGAVVAWIGSANRDESYFPDPHQFDITRSGAN

RQVAFGFGPHYCIGAPLARMTLGIFFEELVQRFGSIELAGEPQHLRSYFI

AGMTHLPIVAQKRKTP

>CYP105W2(2562078265)*Salinispora arenicola* CNH996

MTGYQDRPTGDQPGAPVPSGSTDPGIGAFPLPRRCPFSPPAEYARLRAEH

PVVRLPMLGGDTAWVVSRHADVRQVLSDPRMSADRRRPGFPKFAPTTEGQ

RQASFANFRPPLNWLDPPEHAICRRQIVDEFSVRRVRQSRALVERVVDTH

LDALTAGAPGADLVSTFAYPVPSQVICEVLGVPYGEHEFFERRSTLMFRR

STPADERARYAREIRDFLDMVVTDKERRPGDDVLSRLLYRQRSAGGMDHE

AVVSMAFVLLVAGHVTTSNMLALSVLALLTHPARLARLRAEPERFPAAVE

ELLRYFTVVEAATARTTTAEIMIGGVTIAAGEGVVALGQAANRDPRVFEH

PDEFDPDRDARAHLAFGHGRHICPGQHLARLEMEVALSRLFRRLPGLRLT

MEVSDLPLKEDSNIFGLYALPVAW

>CYP248A2(2562078273)*Salinispora arenicola* CNH996

VLADAVTAFDPTAVDVRRDPYPSYHWLLRHDPVHRGAHQVWYVSRFADVR

AVLGDERFARTGIRRFWTDLVGPGLLSQIVGDIILFQDEPDHGRLRGVVG

PAFSPSALRRLEPTIEATVNDLLRPARALGAMDVVADLAYPLALRAVLEL

LGLPAGDANAVGRWSRAVGRTLDRGATAEDMRRGHAAITEFADYVERALA

ERREDGADLLALMLAAHRSQLMSRNEIVSTVVTFIFTGHETVASQLGNGL

LSLLDHPEQLELVRRQPHLVPQAVEECLRFDPAVQSNTRQLAADVELHGR

RLRRDDVVVVLAGAANRDPGRYDRPDELDIRRDPVPSMSFGAGMRYCLGS

YLARLQLRTALGAMVALPDLRLVCNPNELAYQPRTMFRGLTRLPVAFTPA

G

>CYP107NH1(2562078695)*Salinispora arenicola* CNH996

MTEPVVSLMDPEFWNDPVSAYERLRGSGPLIRMGLPGVPPVWLVTSCEHV

KSALSDPRFVVDAANVPGHHGPGIVDQMMAASGMPDEFRDYMTNMMFTDG

KDHSRLRRLVTPGFSARRIRAMRPRVDQIAEELVESLAEKGSGELIADFS

TPLTTTVICELIGVDRADQAQMGAWMHDYTTGERVVSGRAMVNYTRDLIE

RRRAEPADDMISAMIRSGDEAGDRLSDAEIIAMALLLINAGHHSTAQFIP

NAVLVLLDHPEQLARLRAEPGQLPGAMDELMRLANPVPIATPRYATEDME

FAGVAVRRGEALTGSLEAANFDPERFPAPRQLDTGRDLGRGDGHLSFGAG

PHYCPGAALARLEGEIALDHLLLRRDSLRLAVERDEVDYVDVSLGLRMLS

SLPVRL

>CYP247A8(2562078700)*Salinispora arenicola* CNH996

VRLTPEAGRPVALGSIDLFDPDLFASGDPHSVWDVMRSEAPLHRQVLPDG

RVFWSVTRYEDVCRVLGDHRAFTSERGTVFTQLGADDIAAGKLLTSTDPP

RHTEVRRAIGGRLTARAVAHWQDLVRRAVVRFLEPALDGGCWDLAERAQQ

LPLIIAGSLLGIPDSDWEKLVQLTGMVTAPSDSMFGLGSEAATLAIAHHE

IFDYLSDLVRRRRSAGTGGDSLLDHLMTVRAGAGPLPDEEVVYDSYSLIL

GANATTPHTLSGIVLTLVERPEQCDKVRADPSLIPSMVEEGLRWTSAACN

FMRYATVDTRLTGGTVPAGEAVVAWIASANRDESQFADPHTFDVTRGENR

HVAFGFGPHYCIGAPLARMTARIFFEELFRRFWSIEIDGEPQHLRSNFIA

GMTHLPVVTRKRAQV

>CYP1051A1(2562079004)*Salinispora arenicola* CNH996

MATDAPITRARTVPAWKALPAAVRDTHRALVDVGNWSDGDVVRVSLGVSR

PYLVTNPAHVQEVLHERAAIYPRGDDTALWRSVRKLVGDGILAEGDAWAA

SRRVLAPMFRPARINAMVDTMADAIAGAVDDLHEAATAGTPIDVGRELSR

IVCSAIMRVFFADRITVRDALRIMKAQETIVTAMAPRILAPLVPWWIPMP

GDRRFRAAVRSIDDILLPVLRQAQRQPDDGDDLLSRLVRARADDGRALSE

KRMRDDLVSMVAVTTETSTVVLTWLWPLLANHPDVANRLYDEIDRVVGGG

PVRGDHLAELTYTRMVLDELLRLYPAGWILPRRAATTDVLGGVRINKGAT

VILSPYVTQRMTAWWGPTAEAFDPERFAAGREAADGRHRYAYYPFGVGMH

RCLGEHLFNLEAILIVATLLSRFRFALTDTSMPGVKVAASTRPARTVEMI

LKPVAPVPAR

>CYP244A5(2516451488)*Salinispora pacifica* CNT138

MSATTNAELGEAPETSMPVDPGLFDCMPDLIAAARIAPVVRIPYLGRHAW

VVCDRELVKQALTHPKMGKDITLVPEWMRQPGLMVTAQPPPEYARAMIMS

DGENHARIRRIHAPVLSPRNTERWGEQVATKVEGFLDELSKAAAGSNAEV

DVVTNYTHKIPLAFISEMLGLPPAAEHRLRSITDIMLYSSDYAARREAIG

GLFGAVEEWVRNPDGLRDGVITGLLAGSDGPGAAVTEGEVIVWTLGMIIT

GYETTGSLISTSLYEALRRPPHERPRTDEDITAWIEETLRVHPPFPHPTW

RFPLEDIELGGYLIPKGAPVQVSIAAANRQPGEGADSFDTERRGHGHLSF

GLGMHYCIGAPLVRLEAKIAVRGFLRRFPQARLSADAAVQWESEWMIRRM

SFLPAVLS

>CYP245A11(2516451492)*Salinispora pacifica* CNT138

MSSTTLPRFTLTGWNREDIVNPYPVYRRYREVAAVHRGEAGGDAPETFYV

FSYDQVAQVLSSSCFGRGRSLDATAASVPVPADQKALRAVVENWLVFMDP

PRHTELRSLLNRSFSPRIVTGLRPRIARIAQELLSRLGRQVETDLVEGFA

APLPILVIAELLGIPAERHGWLRTNALALQEASSSRARRDTAGYARAEAA

AQEFTEYFREQVRLRRGSAGDDLLTILANAQLRGAPVSLDAVVGTGVHLL

TAGHETTTNSLAKAVLALQAHPAVLEELRGADGLTADSIEEFLRYDPPVQ

AVTRWTHQDTTLGGWEVPRGSRVVALLGSANRDPARFPLPDALDVHRPAD

RQLGFGLGIHYCLGATLARAELEIGLQTLLNGLPTLGYPAQYVDYADDMV

FHGPSRLILVNPGERFCQ

>CYP107AW6(2516451579)*Salinispora pacifica* CNT138

VETVTGTSAPPPVPYIADPYPTLARIRANGPVSILHSDEGVPMWVIARYR

EVRAALADPRFGQDARRAQALADNRVAGVTLGGDIVHMLNSDPPDHTRLR

RHVQGAFTARRVAAMRPLVERITTSLLDGLAGRKTVDLVQDLAFPLPMLV

ICELLGFPAEERNAYRSWSTAILTHDDDPAVFATALREMTDYIAVQLRIR

RSRPGDDLLTELLAARDAGQLTDDEIVGMVFLLLIGGHETTVNLLGTATL

ALLRNPDQHRWLLANRHALPEAIDEFLRYESPVAMATLRFTTTPVTVDDV

VIPAGELVLVSLGGANRDPDRFPDADRLILDRRDTGHLAFGHGLHRCLGA

FLGKLEGEVALGALLRRHPKLALATEVRQLQWRDTIMLRGLESLPVSLHG

>CYP107AX9(2516451594)*Salinispora pacifica* CNT138

MTAQPAPVFDQRLLRDPHRRYNALRDQAPVHRVRTPDGAPAWLVTRYDDV

RAAFTDPRLSVDKRFSGTDGEHGSSLPPELDAHLLNRDPPDHTRLRRLAA

AAFTPRRVADLRPAVEKTVSTLLDGLAGNDHAELIGSLASPLPLQVMHEL

LGLPTQTSVDFRTWTNTLLSADANQPAQSRSAMANMRRFLIEQVAHKRAQ

PGDDLLTGLLCVREDDDGLTDDELVAMLFLLMFAGYDNTAALIGNAIHAL

LTNVELAEAVRTGSLAVDELVDGVLRWNPSFPLAVRRFAREPITIAGQTI

PAGDRIWLCLASANRDPAHFTEPDEIGIADMRRPHLSFGHGIHYCLGAPL

ARLQTTVAVASLFDRFPGIRLAVPVQDIQWRESFRLRGLVALPVSL

>CYP208A21(2516451606)*Salinispora pacifica* CNT138

MTTTSIDRRRPPGPPRAAALSMLLTMSRDRLGMMTAAARAYGDAAWLPVG

HKALYFFNHPDYAKHVLTDNSDNYTKGIGLVHARRALGDGLLTSEGELWR

EQRRVIRPSFRSGRAPDQASVIAEEVASLVERLRARAGGPPVNVVTEFTG

LTLGVLGRTLLDVDLTALATVGDAFAAVQDQAMFELVTLSAVPTWIPLTR

QRRFRRARAELERIVDDLVARRGDVSGRDDVLSRLILSTGAEPDARVRRQ

RLRDELVTLLLAGHETTASTLGWTLYLIDRHPPVRERLRAEAAEVLGDRL

PAYRDLPDLRFTTMVVQEAMRLYPPVWLLPRRSRRADRVGPYWVPAGSDV

VVSPYTMHRHPGFWPEPDRFDPLRFDPRNAADRPRYAYLPFGAGPRVCVG

SNLGMTEAVIAVAMLCRELRLVRVPTHAAVPEPMLSLRIRGGLPMSVHLA

D

>CYP154M16(2516451623)*Salinispora pacifica* CNT138

MPDRCPVLDPSGRDIHAEADRLRAQAPAVKVELPGGVHAWSITSYDVVRR

LLLDRNVTKNARNHWPKFINDEIPPDWEMISWVAMDNMVTAYGKHLVRLR

RLIAKAFTAQRVETVRPQVEKLVDELLDGLAAETGEVVDLREKFCYPLPA

LLIADLIGMTEQQRAQTAKAMDLMVDTTVSPEQAQAILTGWRTAMDELIA

AKRREPGKDIASDLIAARDDENGGQLTDSELTDTIFAILGAGSETTINFL

DNAVTALVTHPGQLELVRSGRAGWDDVIDEVLRVQCPLASLPLRYAVTDI

ELDGVTIPQGDPILINYAAAGRDPALHGDTAGEFDVTRENKEHVSFGHGP

HYCLGAGIARLVATIGLSRLFERFPDLRLAVPAEELQPLPTFIMNGHRAL

PVRLVPAPAAATAV

>CYP211C6(2516451858)*Salinispora pacifica* CNT138

VPDIEGLLARLYSAQGRQDPYPVYADLHAKAAIAALEPRPERQRVAAVAV

GYDLVAAVLRDPEWFKQPPPGWRDQEILRILQSSMMFINPPDHGRMRHVF

AGTFTPRRLGALEPVINRVTDELLDRMADAGPGEVDFVAEFAYPLPARVM

AEFIGIPATELAWYRERVDRVDAFLDVAGKTPERLAAANAAGAELRFFYR

ELLAHRRRTPGEDLISGLVEAVDAGGVELTEDELISNLIVLFNASFVTTV

YLLSNGLPVLLAHPEVAAALTSSPELAAGAVDEILRLQTPVHLLARAAPR

DTVLGGVSIPQGQNVLLLIAAANRDPAHFPDPDRFDPRRSGPPSLAFGLG

LHYCLGAAVSRLEGRLALPRLLSRFPRLRILEQPVYSGSLFLRGIDKLSV

SPGGREHP

>CYP125A66(2516451993)*Salinispora pacifica* CNT138

MTEPRIPVGFDFTDPAVLERRVPREEFAELRRTAPVWWNAQPKGSAGFDD

DGYWVVTRYADVMAVSRDSETYSTRENTAIARFQPGTTQADREMQRVIMI

NVDPPEHTKLRAIVSRGFTPRAINALRGSLGERAERIVRDAAGRGAGDFV

TDVACELPLQAIAELIGVPQHHRRKVFDWSNQLIGYDDPAYGVDPLAASA

ELLAYAMEMAHERQRNPSDDLVTKLVNAQIDGEHLTADEFGFFVMLLAVA

GNETTRNAITHGMLAFLENPEQWELFKAERPRSAVEEIIRWATPVNVFQR

TALVDTTLAGQAISAGQRVALFYGSANFDESVFEEPERFDITRSPNPHLG

FGGSGVHFCLGANLARLEIELIFNSIADHMPDIRKVADPQRLRSGWINGI

RELPVQYH

>CYP1005A8(2516452658)*Salinispora pacifica* CNT138

VSAVLFRSWTKTAGPHWPAVTRVADQQGTEHLVVTEHELVRQVLTDQVTY

RPDNALDAVTPIPVPALRVLAGHGFRLPPTLANNGGVSHPAIRALVADAL

HPAKVAAQRPWLTKLVAERVAAIGATLDSGGSADLHAELNADLPLLVLAR

LVELPDAPVSAVKQFARAALELFWAPLDADRQLALADEVGRFHQVLREFA

DTGGGLAAALRTTGHPPDVLVGALFFLLVAGQETTSQFLTLLLHRLAGEP

TVRAALRDGGVSVANVVEEGLRLEPPIVTWRRVAAVDSTLGGTAVPAGTS

VLLWLARAGRDPAIVPAPDEFRPGQRGSRRHLAFGAGAHRCLGDQLARME

AAVVVEQAAPLLDGISVVRAPWYPDNLTFRMPDAFVIRR

>CYP211B14(2516452863)*Salinispora pacifica* CNT138

MDASEAVALLMSPPGRLDPYPTYERLRAHGPVVPTAAGFFVVTGYTEADA

VLRNPRFGVMDDEERDGVFPHWQDSPAMMSISQSMIRANPPDHSRMRRLA

AGVFTPRRVAALREVVAAQADELIDEMIRAGRGGAPVDFLGSFAYPLPVT

VICALLGVPAADWAQFRRWASDLTGVLEPEITPQELAIADAGATELRDYF

TELIAQRRRDPTDDLTTALVQTHDADGDRLSGEELLANLVLLLVAGFETT

TNLLGNGLFVLLTHPESATALRDQPELAPGYVDELLRYDSPVQLTTRTVR

ESVPLAGVELPAGSWLLVLLGAANRDPARFTDPARFDPGRAQSPPLSFGA

GAHYCLGAGLARLEAQVAFPLLLRRLPELALAGEPIRRNRLTLRGYETLP

VTVGAVAVDHGTPAGAALSTP

>CYP105AB34(2516453251)*Salinispora pacifica* CNT138

MTETASITTPGTSSTATSGPGSGEVTDTEFPVERGCPFSTPAEYEQIREH

SPLTKVRLTTGREAWWIAGHELARAVLADRRFSSDRRRDNFPFVSTDPET

RKQLQDQPTSMLGMDGAEHAQTRRALMGEFTVRRMAGLRPRIQQIVDQHI

DEMLSSEQRSADLVEALSLPVPSLVICELLGVPYADHDFFQARSGPLVRH

HTPSKVRVRIQEELNTYLGGLIDRKVADPTDDLLSRQIAKQHAAGTFDRT

SLVSMAFLLLIAGHETTANMISLGVVGLLQHPDQLAMIKDDPEKTPPAVE

ELLRYFTITDTVTARVATEDVQLGDTTITAGDGVVISGLAADHDPTVFTD

PNQIDLERGARHHVAFGFGPHQCIGQTLARLELQIVFDTLFRRIPTLRLA

APLDDIPFKSDAIIYGAEELPVAW

>CYP107AY7(2516455216)*Salinispora pacifica* CNT138

MRAEPAPIPRSGARLGQEYDQLRKTGDVHQVLLPDTSLAWLVTSPELVSR

ALADPRLALNRKHSRGGWSGFALPPALDANLLNLDAPDHTRLRRLVGPAF

SPQRVAALRPRIERTAEELVDTVVATGSPVDLVTGYCTPLSVQVIADLLG

VPEARRTDLRAWTDTMLTSYPPDRDAIRQAVVELHGYVVNLIEAKRQRPG

DDLLSALVATEQEGDRLTRDELTSLAFLILFAGYENTANLIASTVLRLLG

HGSLRGARASEAIEETLRLEPPAPAAIRRFPIEEMTIGGATIPAGDTVLL

SIAAATRGTDGNSARLAFGNGPHFCLGAALARVEAEEAITVLARRLPSLA

LAAPGAPVRWRPTFRTHGPAELLVTW

>CYP1278A4(2516455594)*Salinispora pacifica* CNT138

MPSPVGAVVHPNPYPYYAAMVAERPFHFDEQLDTWVAASAAAAQAVLAAP

GCRVRPPHEPVPQGITGTPAGDVFGNLVRMTDGEPQHRLKAIVTQTLGAI

DRSAVAATAMQRARQVLNDSVRTPYEQLMFELPAQVVATLCGLDPAAGGE

ATRLVGHFVQCIPATASPEQQQRAAQAAAGLQELLGPKLDDTQHGLLGEL

VRMATHVGWTDRAPLLANGIGFLSQTYDATAALMGNTLLALSQQECELPT

SEMALQRFVREVIRHDAPIQNTRRFTATPIRHGDVEVPAGQAVLVLLAAA

NRDPAANPDPHMFRADRTTPNVFTFSAGAHHCPGETLAVTIVTTVVEQLL

RVGFDPAKLSTRVTYRPSPNARIPVLTE

>CYP105BL6(2516455659)*Salinispora pacifica* CNT138

MSSHPAAAPGPETATPLHTLAPELTFPQFERATPFDPPEAYTELSGRCPV

APVRMADGKPSWLITSFEGVRAALSDPRLSSDMSHPGFPNRTGKPVDDLL

KDTLGAMDGERHRYYRRMLTGELTVRRAKAMRPVITQITDEALDQLAAAG

PGADLVKHVALVVPSRVACHLVGIPLSEYELFTGMAAKLMEATSSADQIA

ALQDMVSYFDKLVTDREHHDRDDLLGHMVRRYLSTGELTREMLIRLAWTT

MAAGQETTAHMIGLGVAALLRHPDQLELLRREPHLMPGAVDELMRYLPMI

QFGIPRVAMDDVEVDGQTVTAGEGVVALPPLANRDPAVFERPDELDVRRN

ARQHLSFGYGPHQCPAHALARLELEVVYSRLLERFPTLRLADGDADLKVQ

DEDIMYNVSELAVAW

>CYP107AX4(2515991134)*Salinispora pacifica* CNT001

MTSQPAPVFDQLLLRDPHRRYNALRDEAPVHHIRTPDGAPAWLVTRYDDV

RAAFIDPRLSVDKRLSSTDGEHGSSLPPELDAHLLNRDPPDHTRLRRLAA

AAFTPRRVADLRPAVERIVSTLLDGLVGHDQAELIGSLASPLPLQVMQEL

LGLPTQTSVDFRRWTNTLLSADANQPAQSRSAMANMRRFLVEQLAHKRAR

PGDDLLTGLLAAREDDDRLTDDELVAMLFLLMFAGYDNTAALIGNVVHAL

LTNTELPAAVRTGSLALDELVDGVLRWNPSFPLAVRRFAREPITIAGQTI

PAGDRIWLCLASANRDPAQFTAPETLGTTGMRRPHLSFGHGIHYCLGAPL

ARLQTTVAVTILLDRFPGIRLAVPAHDIRWRESFRLRGLVALPVFL

>CYP107AW7(2515991149)*Salinispora pacifica* CNT001

VETVTGTSTPPPVPYIADPYPTLARIRANGPVSILHSDEGIPMWVIARYR

DVRAALADPRFGQDARRAQALADNRVAGVTLGGDIVHMLNSDPPDHTRLR

RHVQGAFTARRVAAMRPLVERITTSLLDGLTGRTTVDLVQDFAFPLPMLV

ICELLGFPAEERNAYRSWSTAILTHDDDPAAFATALREMTDYIAVQLRIR

QTRPGDDILTELLAARDAGQLTDDEIIGMVFLLLIGGHETTVNLLGTATL

ALMRNPDQHRWLLANQHALPEAIDEFLRYESPVAMATLRFTTTPVAVDDV

VIPAGELVLVSLGGANRDPDRFPDADRLILDRRDTGHLAFGHGLHRCLGA

FLGKLEGEVALGALLRRHPRLALAAEVRQLRWRDTIMLRGLESLPVSLHG

>CYP125A65(2515991951)*Salinispora pacifica* CNT001

MTEPRIPVGFDFTDPAVLERRVPREEFAELRRTAPVWWNVQPRGSAGFDD

DGYWVVTRYADVMAVSRDSETYSTRENTAIARFQPGTTQADREMQRVIMI

NVDPPEHTKLRAIVSRGFTPRAINALRGSLAERAERIVRDAAVRGTGDFV

TDVACELPLQAIAELIGVPQHHRRKVFDWSNQLIGYDDPAYGVDPMAAAA

ELLAYAMEMANERQRNPSDDLVTQLVNAQIDGEHLTTDEFGFFVMLLAVA

GNETTRNAITHGMLAFLEHPDQWELFKAERPRSAVEEIIRWATPVNVFQR

TALVDTTLGGQAITAGQRVALFYGSANFDESVFEEPERFDITRSPNPHLG

FGGSGVHFCLGANLARLEIELIFNSIADHMPDIRKVADPQRLRSGWINGI

REMPVRYR

>CYP208A22(2515992033)*Salinispora pacifica* CNT001

MTVIADRGGRIPPGPPVTAGLRLLLALGRDRLGMMTSAAAEYGDVARLPV

GPKKLYFFNHPDHAKHVLADNHANYQKGIGLVHARRALGDGLLTSEGELW

RKQRRVIQPAFQNRRLAQYAGAVGQEATRLVARLATRVDGPPVDVLDEMT

RLTLGVLGRTLLDAELTGFHGVGESFAAVQDQAMFELETLNTVPTWIPLR

RQRRFRRARQHLQEVVDVLAAERGQAVEGRDDVLSRLILSTRAEADPQLG

RERLRDELVTLLLAGHETTASTLGWSLHLLDQHPELRERVRHEARTVLGD

RVPAYEDLHQLRYTAMVVEEAIRLYPPVWILTRKARAEDEIGGYRVPAGA

DILICPYTLHRHPRFWAEPERFDPERFDPSRTTDRPRYAYIPFGAGPRFC

VGNNLGMLEATLVLAVLLRDLRLEGLPGRAVVPEPMLSLRVRGGLPMTVR

RVD

>CYP211B11(2515992548)*Salinispora pacifica* CNT001

MDASEAVALLTSPPGRLDPYPTYERLRAHGPVVSTAAGFFVVTGYTEADT

VLRNPRFEVMDDEERDGVFPHWQDSPAMISISRSMIRANPPDHSRMRRLA

AGVFTPRRVAALREVVAAQADGLVDEMIRAGRGGAAVDFMGSFAYPLPVT

VICALLGVPTADWARFRHWASDLTGVLEPEITPQELAIADAGASELRDYF

TELIAQRRRAPADDLTTALVQAHDADGDRLSGEELLANLVLLLVAGFETT

TNLLGNGLVVLLTHPDSATALRGQPELAPGYVEEFLRYDSPVQLTSRTVR

ESVSLAGVELPAGSWLLVLLGAANRDPARFTDPARFDPRRAQSPPLSFGA

GAHYCLGAGLARLEAQVAFPLLLRRLPELALAGEPTRRHRLTLRGYETLP

VTVGAVPADPGTPAGVALGTP

>CYP1005A6(2515994036)*Salinispora pacifica* CNT001

VSAVLFRSWTKTAGTHWPAVTRVADQQGTEHLVVTEHALVRQVLTDQVTY

RPDNALDAVTPIPVAALRVLAGHRFRLPPTLANNGGVSHPAIRALVADAL

HPAKVAAQRPWLTELVAERVAAIRATLDSGGSADLHAELSADLPLLVLAR

LVELPDAPVSAVKQFARVALELFWAPLDADRQLALADEVGRFHQVLREFA

DTGGGLAAALRATGHPPDVLVGALFFLLVAGQETTSQFLTLLLHRLAGEP

TVRAALRDGSVSVPNVVEEGLRLEPPIVTWRRVAAVDSTLGGTAVPAGTS

VLLWLARAGRDPAIVSAPDEFRPGQRGSRRHLAFGAGAHRCLGDQLARME

AAVVVERVSPLLDGVTVVRAPWYPDNLTFRMPDAFVIRRGPAGAAER

>CYP161N4(2515994691)*Salinispora pacifica* CNT001

VNTVAQLPFTQTHVLDVAPALRLLQSRGKVHRVRTPEGVPAWLVTGHAEV

QQLLDDDRLSRSDPGGRDGGTALLNKLLGPLADDHPRLRSLLEPQFTPER

LEPLRAVVEKLTEQHLDELATRTPPVDLRPTLAMSLPILVLCEWLGVPAE

DKGRFSVWTQDAAGVQDPERSQRGLAELFGYCRQLVAAKRQDPGDDVISR

LIATAGIGDTEVVALTALLLFGGYETTVARIGTGVLLLLTNPDQWAAVRA

DPALVPATVDELLRRSMPNPHNGGMPRFAVTGFEIDGAAIRAGDLVLLNI

IAANHDETAFPDPDRLDITRPTAGSLAFGYGRHSCVGAPLARMVLRVALS

RLITRFPDLRLAVGVDELKLRHETLVGGLVELPVTWGPR

>CYP2054A3(2515994696)*Salinispora pacifica* CNT001

MSISADSEQVQGRPFDPYGAHRDDPYTFLAGLGVFYAPLLDAWCVTRRED

MVAVLRDDRSFSARDHNPRPAVALPDDVNQMFRTWRGAGAVAVGSLDPPA

HAKIRDVLNIGFTPARVRAFEPTMRAVAADLADRIGDAPEFDFIADFAVP

FALEVIGRRLGVPDDYLDRCRTWSEQRIELMMAQGDADHDRLREFARGLM

EFGEFARSLVRDRVADPRDDLISELLHDGKAGRTLTADEVAVQIPTLIFA

GHMTCAEALGTIFYQQLRSPGGWARVVDRTIPVGDLVEEGLRFDSPLAGM

YRTATRDVTVGGIRLTAGSRLLLLYGAAGRDSRAHACPAAFRPGDGSSGH

LAFGHGIHFCLGAGFARAELRVAVEVLAARMPDLGLAPGRPPRFRPVFPL

RALTELRVTRSGGGSGP

>CYP107FH3(2515994701)*Salinispora pacifica* CNT001

VPIELDEAFVQDPYAVYEKLRAEGPAHRVRMPPGVPLIGGLPVWLITGYD

AVRAALADSRLSTDLHRIDGLFAQKDPDCSHRGGFSSALASHMMHTDPPD

HTRLRKLVSKAFTRRAIEALRPRIQQTTDELTADLAGHDTVDLLDAFAFP

LPIRVICLLLGVPVAEQENFRSWSRALVSGHSPEAAATAATEVAAYLGDL

VERKRHATTDDVLTALVAAHDVDDRLTHTELVSTAYLLFIAGFETTLNAL

GNGTLHLMLHRDQWTALRADRALLDNAVEEFLRLESPLKHATFRCATESL

RIGDAEIAAGDFVLLAIASANRDPRRFPDPHTLDVRRPAAGHLAFGHGIH

HCLGAPLARVEVRMAFDALLDAFPDMRLATDPAGLRWRNSTIIRGLDSLP

VHLNN

>CYP107AY9(2515994839)*Salinispora pacifica* CNT001

MSQDQPTRAELAPIPRSGTRIGPEYDQLRKTGDVHQVLLPDTSMAWLVTS

PDLVSRALADPRLALNRKHSRGGWSGFALPPALDANLLNLDAPDHTRLRR

LVGPAFSPQRVAALRPRIQRTAEELAETMVATGSPVDLVTGYCTPLSVQV

IADLLGVPDAGRTDLRAWTDTMLTSYPPDRDAIRQAVVELHGYVVNLIEA

KQQRPGDDLLSALVATEQEGDRLTRDELTSLAFLILFAGYENTANLIAST

VLRLLDHGSLRGVRVSEAIEETLRLEPPAPAAIRRFPTEEMTIGGATIPA

GDTVLLSIAAATRGADGNPARLAFGNGPHYCLGAALARVEAEEAITVLAR

RLPGLALAVPGAPVRWRPTFRTHGPAELLVAW

>CYP244A10(2515994859)*Salinispora pacifica* CNT001

MQDTAQANLAEAPEVRMPIELRPTDCLPELLAAARVAPVVRTPYLDQHAW

VVCDRELVKQALTHPKLGKDVALAPDWMRQPGQMVTAMPPPEYARMMVMS

DGEHHARIRRIHAPVLSPRNTERWSERVAALVDGFLDNLDSADGTEVNLI

TDYTHKIPLAFTAEMLGLPPGAERRLHDITEVMLYSADYALRQQAVGELF

EAVQEWVRDPAGLRDGVITGLLASADGPDATVTKGEAIVWTLSLIINGYE

TTGSLISAALYEALRRPARERPHTDEAVAAWIEETLRVQPPVPHTTWRFA

LADLDLGGYLIPRGAPVQISLAAANLDPDEDADSFDAQRRGRGHLSFGLG

AHYCIGAPLARVQTKIALRGFLRRFPQARLSLDTAPRWESEWMIRRMSVL

PVLLA

>CYP105AB28(2515995297)*Salinispora pacifica* CNT001

MTETASTTTPGISSTTTSGPASGEVTDAEFPLERGCPFSTPTEYEQIREH

SPLAKVRLTTGREAWWIAGHELGRAVLADRRFSSDRRRDNFPFVSTDPET

RKQLQDQPTSMIGMDGAEHAQARRALMGEFTVRRMAGLRPRIQQIVDQHI

DEMLSSDQRTADLVEALSLPVPSLVICELLGVPYADHDFFQARSGPLIRH

TTPTEVRLRIQKELNTYLGALIDRKVADPTDDLLSRQIAKHHAAGTFDRT

SLVSMAFLLLIAGHETTANMISLGVVGLLQHPDQLAMIKEDPEKTAPAVE

ELLRYFTIADTVTARVATEDVQLGGTTINAGDGVVISGLAADHDPTVFTD

PDRLDLERGARHHVAFGFGPHQCIGQTLARMELQIVFDTLFRRIPTLRLA

APLDDIPFKSDAFVYGAERLPVAW

>CYP107E37(2515995943)*Salinispora pacifica* CNT001

VTIDQEIREYPFRESRGIGIDPTYELLRRTEPLARVQLPYGEVSWLVTRY

EDVKTVLTDPRFSRAAAQGKDQPRTRAEMTYEGIIGLDPPDHTRLRRLAG

KALTARRVNAIRADAQRIANEYVDEMIAKGSPGDLVELFALPYPVTVICE

LLGVPFEDRAQFRIWTEGLTSTSEQLMVYAEQLFDYMGKLVAQRRAEPTD

DLLGALVKARDEGDRLTEQELLSIAGVGLLLTGVETVSTHIPNFVYALLT

HPELMAQLRADRSLVPAAVEELLRMIPLNPAAMFPRYAVEDVTLSGITVR

AGQPVLVSLPGANRDPEVFENPETFDFTREQNPHVAFGHGPHHCLGAQLA

RMELQVALHTVLDRFPDLRLADGDEGVSWKSGLLVRGPSRLLVGW

>CYP124M3(2515995969)*Salinispora pacifica* CNT001

MTVPIELDAVDLSDNDFWTKPLEYRHAVFNALRAQPGLPHFASPEFGSAP

RGRGYYALTRMDDVLAVSRNPSVFISGKGNVAMEVPAEFLDQSLITMDNP

RHARLRRIVSRGFTMKAVTALMDNASQMAKQIVDEVVERGECDAVVDISA

KLPLGIICNMMGIPDSQQQFVFEQTNVMLGVQDPEYVGEQNDAMLAMSVA

SQELADLMHELATLRAKNPTDDLISKLLSAEVNGEALTPSELAHFFILLT

GAGTETTRNAISWGIQLLTQSPEQREAWLSDIDGVTPTAVEEIVRWSSPV

ISQRRTVAEDAEPVKLAGQLLGPGDKVLMFYGAANRDPNYFVHPEGFDVR

RSPNPHVGYGGPGPHFCLGAHLARLEISVMFRELLTRIPDIHATGEPARL

KSPLINGIKRLPVAFTPGAR

>CYP294A4(2515996040)*Salinispora pacifica* CNT001

MTLLDTAGLGPLPSFLQQQGAGAVLPIVSPAGNTMWLVRDYTLARKVLTD

PRFSRAAAVTPQAPKFNDAQPAADSMMSMDGAEHARLRRLVSGAFTTGRV

AAIAPWVERWVDERLDRLERRGTGADLIGDLAAPLSVSVLCTLLGIPAAD

SERFRGWVEVLFDITASSPHEKGRRRLELLDYMGQTIEQKRRQPDDALLT

VLIKAQERGEMSMAELLTLGLTLLMAGYETTVGQIGLAALVILSDEVVYD

ALKRQPERLPGTVEELLRLTPATPLSFSRVATRQVQLGEVTVRAGEGVVV

SLLHGNRDPATFPDPQRLLPQGRDAGHLTFGHGVHRCLGAPLARLQIQTV

LNRLIGHFPRLRLVDALEPVAWKHGLATRGLSRLSVEWR

>CYP163B1(2515515979)*Salinispora tropica* CNH898

VTAADTGRVGSGESQTPAHTVDLADPATFANHDLTGFWQQLRDEEPIHWN

PPTAGRRGFWVVSRYADILDVYRDDVTFTSERGNVLVTLLAGGDAGAGRM

LAVTDGPRHAELRKLLLRALGPRVLAPVCAAVRTNTRQMIREAVTKGECD

FASDIASRIPMMTISNLLGVPDADRAFLLSLTKTALSADDESISETESAM

ARNEILLYFQDLMEFRRDHPGEDVVSMLVNSSIDGAPLSDDDIVLNCYSL

IIGGDETSRLTMIDSVNTLAAHPQQWRRLKDGQCEIDKAADEVLRWASPS

MHFGRVAARDTILHGVRIRADDIVTLWHASGNRDERVFHRPEVFDLGRTP

NRHLSFGHGPHYCIGSYLAKVEISELLIALRDLTSGFETTGEPQRIRSNL

LTGFATMPVRFVPDRAGLARDALDG

>CYP107NH1(2515516273)*Salinispora tropica* CNH898

MTEPVVSLMDPEFWNDPVSAYERLRGSGPLIRMGLPGVPPVWLVTSCEHV

KSALSDPRFVVDAANVPGHHGPGIVDKMMAASGMPDEFRDYMTNMMFTDG

KDHSRLRRLVTPGFSARRIRAMRPRVNQIAEELVESLAEKGSGELIADFS

APLTTTVICELIGVDRADQAQMGAWMHDYTTGERVVSGRAMVNYTRDLIE

RRRAEPADDMISAMIRSGDEAGDRLSDAEIIAMALLLINAGHHSTAQFIP

NAVLVLLDHPEQLARLRAEPGQLPGAMDELMRLANPVPIATPRYATEDME

FAGVAVRRGEALTGSLEAANFDPERFPAPRQLDTGRDLGRGDGHLSFGAG

PHYCPGAALARLEGEIALDHLLLRRDNLRLAVERDEVDYVDVSLGLRMLS

SLPVRL

>CYP247A8(2515516279)*Salinispora tropica* CNH898

VRLNPEAGRPVALGSIDLFDPDLLASGDPHSVWDVMRSEAPLHRQVLPDG

RVFWSVTRYEDVCRVLGDHRAFTSERGTVFTQLGADDIAAGKLLTSTDPP

RHTEVRRAIGGRLTARAVAHWEDLVRRAVVRFLEPALDGGCWDLAERAQH

LPLIIAGSLLGIPDSDWEKLVQLTGMVTAPSDPMFGLDSEAATLAIAHHE

IFDYLSDLVRRRRSVGTGGDSLLDHLMTVRAGAGPLPDEEVVYDSYSLIL

GANATTPHTLSGIVLTLAERPEQCDKVRADASLIPSMVEEGLRWTSAACN

FMRYATVDTRLTGGTVPAGEAVVAWIASANRDESQFADPHTFDVTRGENR

HVAFGFGPHYCIGAPLARMTARIFFEELFRLFWSIEIDGEPQHLRSNFIA

GMTHLPVVTRKRAQV

>CYP107Z27(2515516289)*Salinispora tropica* CNH898

MSCTHPCHSLMDPTLIDDPAGGFSRIRDESPLAHAMVPGVDGPVWLVTRH

ESVRKVLGDRRFVNDPTNVPGSSTPDLWAHAALAQGVPHEYLEHVRSMLQ

LDGEVHARLRRLVSRAFTMRRITALRPRMMQLTEELLDLLPAKAEDGVVD

LVEHFNYVQPISVICALVGVPAADRAAWIRWSRALTSMESGTIGDAVTGM

VDNIRALIEQRRAEAADDLLTDLVRVRDADGDRLSEREMITMVITLVTAG

HDSTGLLLSNGLAALLTHPDQLAKLRADPSLGPQAFDELMRWCSAIVAAR

PRYATEDVELEDGLVRRGDVVIPVLVSANYDPEVFEDPHRLDISRVHEQR

RFHHVGFGDGLHYCLGAALAKHEAAIAITALLERYPGLELAVPAQRLRRA

RLPLTWRLDSLPVRLGIGG

>CYP211C1(2515516685)*Salinispora tropica* CNH898

VLDVEGLLTRLYSEQGRQDPYPVYADLHAQGAIAALAPRPEGQRVAAVAV

GYDLVGAVLRDPEWSKQPPPGWMEQEILRTLQSSMMFINPPDHGRMRKVF

AGTFTPRRLGTLEPVINRVADELLDRMADAGPGEVDFVAEFAYPLPARVM

AEFIGIPATELAWYRDRVDRIDAFLDVAGKTPERLAAANAAAAELRVFYA

DLLARRRRTPGEDLISGLVEAVDAGGVQLTEDELINNLIVLFNASFVTTV

YMLSNGLPVLLEHPEVAAALADDPELTAGAIDEILRLQTPVHLLARAAPR

DTVLGGVPIPQGQNVLLLIAAANRDPAHFPDPDRFDPRRPGPPSLAFGLG

LHYCLGAAVSRLEGRLALPRLLSRFPRLRIMEQPVYSGSLFLRGIDKLSV

SPGGRMHP

>CYP125A15(2515516818)*Salinispora tropica* CNH898

MTEPRIPVGFDFTDPAVLERRVPREEFAELRRTAPVWWNVQPRGSAGFDD

DGYWVVTRYADVMAVSRDSDTYSTRENTAIARFQPGTTRADLEMQRVIML

NVDPPEHTKLRAIVSRGFTPRAINALRGSLAERAERIVRDAAVRGTGDFV

ADVACELPLQAIAELIGVPQHHRRKVFDWSNQLIGYDDPAYGVDPLTAAA

ELLAYAMEMANERQLNPSDDLVTKLVNAQIDGEHLTTDEFGFFVMLLAVA

GNETTRNAITHGMLAFLEHPEQWELFKAERPRSAVEEIIRWATPVNVFQR

TALVDTTLGGQAISAGQRVALFYGSANFDESVFEEPERFDITRSPNPHLG

FGGSGAHFCLGANLARLEIELIFHSIADHMPDIRKVAEPRRLRSGWINGI

REMPVRYR

>CYP208A4(2515516897)*Salinispora tropica* CNH898

VTVAAAGRTFSGPTGAALLRSLWQLGQDRLGLMTSAARYGDAVRLGVGSR

SLYFFNHPDHAKHVLADNSGNYTKGLGLVHARRALGDGLLTSEGELWREQ

RRVIQPVFQAKRVAGQAHAVAEEADRLIARLRARRGRGPVNLTDEFTALT

LGVLGRTLLDANLDAFTTVGAAFEEMQNQAMFEMASMSMVPMWVPLPQQL

RFRRARRELERIVGRLVADRTARGEGTGADDALSRLIASTRDEPDPGVAR

RRMRDELVTLLLAGHETTASTLGWTFHLINQDPRVRVRLREEAIDVLGGR

LPEYADLARLTYTKMVVSEAMRLYPPVWMLSRLARDADVVDGYPVPARAD

VLICPYTLHRHPAFWPEPERFDPERFDPEVTTDRPRYAYVPFGAGPRFCV

GNHLGLMEAVFVVAMVSREFDLVAPVGQPVVAEPMLSLRVRGGLSMTVEP

VS

>CYP154M1(2515516926)*Salinispora tropica* CNH898

MRRRCSVVIDPAGTDIHAEGARIRANGSVSQVELPGGVLAWSVTGQQVAR

KVLSDQRFSKDPRKHWTDYLEGRIGQDFPLIGWVLMDNLTTAYGSDHSRL

RKPCANAFTPRRVEALRPAVERAAVELLGELATVSPTESVDLKARYAHPL

PSRVICDLFGVPEEDREEMLRGGEVNVDTRVSAEEAAANVERWHQQMLDF

IEEKRRNPGPDLTSDLIAAQQAEGSRLTDSEMVGTLHIMLATGTEPVKNL

IGNAVFALLTHPEQLDLVRSGRAGWDDVIQETLRMQAPVAHLPFRFAVED

VDIDGVTIRRGDPLLVNFAAIGRDPDVHGDTAAEFDITRADKEHLSFGHG

VYRCIGQPLALREAEIALRMLFQRFPNLVLAVPPEEVTPQPTFIMNGLDT

LPVLLKGRA

>CYP211B1(2515517356)*Salinispora tropica* CNH898

MDASEAVALLMSPLGRIDPYPTYERLRAHGPVVQTAAGFFVVTGYTEADT

VLRNARLAFEVMDDELRDDVFPHWQDSPAMKSIARSMIRANPPNHGRMRR

LAAGAFTPRRIAALREVVTAQADELADEMIRAGRDGAPVDFMGSFAYPLP

VAVICALLGVPAADWARFRGWASDLTAVLEPEITPQELTVADAGASELRD

YFTELIAQRRRAPADDLTTALVQTHDADGDRLSGEELLANLVLLLVAGFE

TTTNLLGNGLVVLLAHPDSATALRGQPELAPGYVDELLRYDSPVQLTTRT

VRESVLLAGVELPAGSWVLVLLGAANRDPERFTDPTRFDPGRAQSPPLSF

GAGAHYCLGAGLARLEAQVAFPLLLRRLPELALAGEPTRRNRLTLRGYET

LPVTVSAIAADHGTPAGVARGTP

> CYP107AX1(2515518410)*Salinispora tropica* CNH898

MTSRPTAVFDQCLLRDPHSRYNALRDQAPVHHVLTPDGAPAWLVTRYNDV

RAAFTDPRLSVDKRFSGTDGEHGSSLPPELDAHLLNRDPPDHTRLRRLAA

AACTPRRVADLHPAVERIVSTLLDGLAGHDRAELIGSLASPLPLQVMHEL

LGLPTQANIDFRTWTNTLLSADANQPAQSRAAMANMRRFLIEQLAHKRAQ

PGDDLLTGLLAAREDDDRLTDDELVAMVFLLMFAGYDNTAALIGTVTHAL

LTNAELHEAVRGGSLALDELIDEVLRWNPAFPLAVRRFAREPITIAGQTI

PAGDRIWLCLASANRDPAQFTQPDELGIIGLRRPHLSFGHGIHYCLGAPL

ARLQTTIAVTSLLNRFPEMRLAVSAHDIRWRESFRLRGLIALPVYL

>CYP107AW1(2515518632)*Salinispora tropica* CNH898

MESVTSTSAPPPVPYIADPYPALARIRANGPVSILHSDEGIPMWVIARYR

NVRAALADPRFGQDARRAQTLADNRVAGVTLGGDVIHMLNSDPPDHTRLR

HLVQGAFTARRVAAMRPLVERITTSLLDGVGGRQTVDLVQDFAFPLPMLV

ICELLGFPAEERDAYRSWSTAILTHNDDPAAFATALRDMTDYIEVQLRHR

RARPGEDLLTELLAARDAGQLTDDEIVGMVFLLLIGGHETTVNLLGTATL

ALVRNPDQHRWLLANPHALSEAIDEFLRYESPVAMATLRFTTAPVTVDDV

VIPAGELVLVSLGGANRDPDRFPDADRLILDRRDTGHLAFGHGLHRCLGA

FLGKLEGEVALGALLGRYPGLTLAAEVRQLRWRDTIMLRGLESLPVSLHG

>CYP105AB2(2515519026)*Salinispora tropica* CNH898

MTETASIATTRTASGQLTDAEFPVQRGCPFTAPTEYEQIREESSIAKVRL

KNGGEAWWIAGHELGRSVLADRRFSSDRRRDNFPFVSTDPETRAQLQSQP

TSMLGMDGAEHAQTRRALMGEFTVRRMAGLRPRIQQIVDQHIDEMLATPQ

RSVDLVEALSLPVPSLVICELLGVPYADHDFFQGLTGPLLRHTTPPEVRL

RIQEELNTYLGTLIDHKLTDPTDDLLSRQIAKHRDNGTFDRASMVSLAFL

LLVAGHETTANMISLGVVGLLQHPDQLVIIKDDPDKTPLAVEELLRYFTI

ADSVTARVATEDVQLGDTTINAGDGVVISGLAADRDPTVFAEPDRLDLER

GARHHVAFGFGPHQCIGQTLARMELRIVFDTLFHRIPTLRLAAPLDDIPF

KSDAFVYGIEELPVAW

>CYP1005A1(2515519055)*Salinispora tropica* CNH898

VSAVLFRSWTKTAGTHWPAITRVADQQGTEHLVVTQHALVRQVLTDQLTY

RPDNALDAVTPIPVAALRVLAGHRFRLPPTLANNGGVSHPAIRALVADAL

HPAKVAAQRPWLTGLVAERVAAIRRTLDSGGSTDLHAELNADLPLLVLAR

LVELPDAPVSAVKQFARAALELFWAPLDADRQLALADEVGRFHQVLREFA

DTGGGLAAALRATGHPPDVLVGALFFLLVAGQETTSQFLTLLLHRLADEP

TVRAALRADSVSVADVVEEGLRLEPPIVTWRRVAAVDSTLGGSTVAAGTS

VLLWLARAGRDPVVVPAPDEFRPGQRGSRRHLAFGAGAHRCLGDQLARME

AAVVVEQVTPLLDGVTVVRPPWYPDNLTFRMPDAFVVRR

>CYP1004B1(2515519521)*Salinispora tropica* CNH898

MTTSALAPRFDALDPNVVEDPYPEYARLRAAGPLCRLGPGSWGVTRFADV

TNLQHDPRLGSEFPAGYHEISVGDGPASAFFQRVMLYRDPPDHIRLRRLM

SGAFTPAVVRRLRSHIEDLVDELLAPALAAGRMDLVPELAYPLPVRVVCR

LMGIPPESTEDVRHHATNIGRAFTAVVPEQARTEADEAVSWLREHLGALL

EQRRSHRGDDLLSRLLDAEESGDNLSADEIVDNTVFSFFAGFETTVHMIT

TGTAALLAHPDQLARLRADPSLVTTAVDEFLRWDAPIQGTARYVREPIEI

GGRTIRRGRVLVLMIGSANHDERRFAQPDRLDVGRQDNPHVAFGGGAHLC

LGAFLARMEGAVVFDRLARLAVLEPDGPTVREPNTPFRAYASVPVRIGDR

>CYP1004A1(2515519525)*Salinispora tropica* CNH898

MRANTARPHLSAAFLATKDDPYPAYAELRARGPLTRAELGQWLVTGHGAV

SALLRDGRLESRMPAEYTRLTLGDSPGVDFLHRIVLTRTPPEHTRLRRFI

GRALGTPVVRRLHDRIAAATDALLEPALDRGRLDVVTELAVPLPVGVVCD

LIGIPTGDRPAVLTRVTALAKVFDAANLSPADLADINTALPWLHDYFGDL

LAVRRAGSGGPTLTEMYWEESASDRLAVADFVDNMLFLFHAGFETTMGLV

SNGVAALLNNPEQLGRLRADPALVPSAVEEFLRYDAPIQNVIRVARKPVE

VAGQKIRAGRTVLLLLGAANRDEEVFADAERLDVGRDPNPHLGFGGGLHH

CVGTALARLMAVVVFERLVDRVTVLGPAAPAVRRRHASLRSYDHLPLAVA

AR

>CYP107AY1(2515519736)*Salinispora tropica* CNH898

MRAELAPIPRSGARLGQEYDQLRNAGDVHQVLLPDSSLAWLVTNPKLVSR

ALTDPRLALNRRHSRGSWSGFALPPALDANLLNLDAPDHTRLRRLVGPAF

SPQRVSALRPRIRRTAEHLLDTLVATSGPVDLVTGYCTPLSVQVIADLMG

VPEAGRADLRTWTDTMLTSYPPDRDAIRQAVVELHGYVVDLIDTKRQQPG

DDLLSALVTIEQDGDRLTRDELTSLAFLILFAGYENTANLIASTVLRLLD

HGGLRGVQLPEAIEETLRLEPPAPAAVRRFPTEEMTIGGATIPAGDIVLL

SIAAATRGTAGNAARLAFGNGPHFCLGAALARVEAEEALTVLARRLPDLA

LALPVAQVRWRPTFRTHGPAELLVTW

>CYP107E3(2515520828)*Salinispora tropica* CNH898

VTIDQEIRKYPFCESPGIGIDPTYGLLRSTEPLARVQLPYGEVSWLATRY

EDVKTVLTDPRFSRAAAQGKDQPRTREEMTYEGIIGLDPPDHTRLRKLAG

KALTARRVNAIRADAQRIANEYVDGMIAKGSPGDLVELFALPYPVTVICE

LLGVPFEDRAQFRIWTEGLTSTSEQLMVYAEQLFGYMGKLVAQRREEPTD

DLLGALVKARDEGDRLTEQELLSIAGVGLLLTGVETVSTHIPNFVYALLT

HPELMAQLRADRSLVPAAVEELLRMIPLNPAAMFPRYAVEDVTLSGITVR

AGEPVLVSLPGANRDPEVFENPETFDFTREQNPHVAFGHGPHHCLGAQLA

RMELQVALHTVLDRFPDLSLADGDEGVSWKSGLLVRGPSRLLLAW

>CYP107AY17(2515520887)*Salinispora tropica* CNH898

MTGNPTPIPRSGAQLGQQYDHLRKTGDVHEVLLPDASLAWLVTNPEVAAR

ALADPRLALNRRHSRGGWSGFALPPSLDANLLNLDAPDHTRLRRLVGPAF

SPQRVAALRPGIRRAAEHLLDTLVATSGPTDLVTGYCNPLSVQVIADLMG

IPEAGRTNLRAWTDTMLTSYPPDRDAIRQAVVELHGYVVDLIDVKQQQPG

DDLLSALVTIEQDGDRLTRDELTSLAFLILFAGYENTANLIASTALWLLN

HGGLNEVPISEAIEEALRHEPPAPVAIRRFPTEDIIIGDVTIPAGDTVLL

SIAAATRGLDGNAARLAFGNGPHYCLGAALARAEAEEALAVLARRLPGLT

LAVPVAQVRWRPTFRTHGPAELLVSR

>CYP107AW7(2563541222)*Salinispora pacifica* CNY498

VETVTGTSTPPPVPYIADPYPTLARIRANGPVSILHSDEGIPMWVIARYR

DVRAALADPRFGQDARRAQALADNRVAGVTLGGDIVHMLNSDPPDHTRLR

RHVQGAFTARRVAAMRPLVERITTSLLDGLTGRTTVDLVQDFAFPLPMLV

ICELLGFPAEERNAYRSWSTAILTHDDDPAAFATALREMTDYIAVQLRIR

QTRPGDDILTELLAARDAGQLTDDEIIGMVFLLLIGGHETTVNLLGTATL

ALMRNPDQHRWLLANQHALPEVIDEFLRYESPVAMATLRFTTTPVAVDDV

VIPAGELVLVSLGGANRDPDRFPDADRLILDRRDTGHLAFGHGLHRCLGA

FLGKLEGEVALGALLRRHPRLALAAEVRQLRWRDTIMLRGLESLPVSLHG

>CYP107AX4(2563541236)*Salinispora pacifica* CNY498

MTSQPAPVFDQLLLRDPHRRYNALRDEAPVHHIRTPDGAPAWLVTRYDDV

RAAFTDPRLSVDKRLSSTDGEHGSSLPPELDAHLLNRDPPDHTRLRRLAA

AAFTPRRVADLRPAVERIVSTLLDGLVGHDQAELIGSLASPLPLQVMQEL

LGLPTQTSVDFRRWTNTLLSADANQPAQSRSAMANMRRFLVEQLAHKRAR

PGDDLLTGLLAAREDDDRLTDDELVAMLFLLMFAGYDNTAALIGNVVHAL

LTNTELPAAVRTGSLALDELVDGVLRWNPSFPLAVRRFAREPITIAGQTI

PAGDRIWLCLASANRDPAQFTAPDTLGTTGMRRPHLSFGHGIHYCLGAPL

ARLQTTVAVTILLDRFPGIRLAVPAHDIRWRESFRLRGLVALPVFL

>CYP211B11(2563541657)*Salinispora pacifica* CNY498

MDASEAVALLTSPPGRLDPYPTYERLRAHGPVVSTAAGFFVVTGYTEADT

VLRNPRFEVMDDEERDGVFPHWQDSPAMISISRSMIRANPPDHSRMRRLA

AGVFTPRRVAALREVVAAQADGLVDEMIRAGRGGAAVDFMGSFAYPLPVT

VICALLGVPTADWARFRHWASDLTGVLEPEITPQELAIADAGASELRDYF

TELIAQRRRAPADDLTTALVQAHDADGDRLSGEELLANLVLLLVAGFETT

TNLLGNGLVVLLTHPDSATALRGQPELAPGYVEEFLRYDSPVQLTSRTVR

ESVSLAGVELPAGSWLLVLLGAANRDPARFTDPARFDPRRAQSPPLSFGA

GAHYCLGAGLARLEAQVAFPLLLRRLPELALAGEPTRRHRLTLRGYETLP

VTVGAVPADPGTPAGVALGTP

>CYP125A65(2563541866)*Salinispora pacifica* CNY498

MTEPRIPVGFDFTDPAVLERRVPREEFAELRRTAPVWWNVQPRGSAGFDD

DGYWVVTRYADVMAVSRDSETYSTRENTAIARFQPGTTQADREMQRVIMI

NVDPPEHTKLRAIVSRGFTPRAINALRGSLAERAERIVRDAAVRGTGDFV

TDVACELPLQAIAELIGVPQHHRRKVFDWSNQLIGYDDPAYGVDPMAAAA

ELLAYAMEMANERQRNPSDDLVTKLVNAQIDGEHLTTDEFGFFVMLLAVA

GNETTRNAITHGMLAFLEHPDQWELFKAERPRSAVEEIIRWATPVNVFQR

TALVDTTLGGQAITAGQRVALFYGSANFDESVFEEPERFDITRSPNPHLG

FGGSGVHFCLGANLARLEIELIFNSIADHMPDIRKVADPQRLRSGWINGI

REMPVRYR

>CYP208A22(2563541964)*Salinispora pacifica* CNY498

MTVIADRGGRIPPGPPVTAGLRLLLALGRDRLGMMTSAAAEYGDVARLPV

GPKKLYFFNHPDHAKHVLADNHANYQKGIGLVHARRALGDGLLTSEGELW

RKQRRVIQPAFQNRRLAQYAGAVGQEATRLVARLATRVDGPPVDVLDEMT

RLTLGVLGRTLLDAELTGFHGVGESFAAVQDQAMFELETLNTVPTWIPLR

RQRRFRRARQHLQEVVDVLAAERGQAVEGRDDVLSRLILSTRAEADPQLG

RERLRDELVTLLLAGHETTASTLGWSLHLLDQHPELRERVRHEARTVLGD

RVPAYEDLHQLRYTAMVVEEAIRLYPPVWILTRKARAEDEIGGYRVPAGA

DILICPYTLHRHPRFWAEPERFDPERFDPSRTTDRPRYAYIPFGAGPRFC

VGNNLGMLEATLVLAVLLRDLRLEGLPGRAVVPEPMLSLRVRGGLPMTVR

RVD

>CYP124M3(2563541985)*Salinispora pacifica* CNY498

MTVPIELDAVDLSDNDFWTKPLEYRHAVFNALRAQPGLPHFASPEFGSAP

RGRGYYALTRMDDVLAVSRNPSVFISGKGNVAMEVPAEFLDQSLITMDNP

RHARLRRIVSRGFTMKAVTALMDNASQMAKQIVDEVVERGECDAVVDISA

KLPLGIICNMMGIPDSQQQFVFEQTNVMLGVQDPEYVGEQNDAMLAMSVA

SQELADLMHELATLRAKNPTDDLISKLLSAEVNGEALTPSELAHFFILLT

GAGTETTRNAISWGIQLLTQSPEQREAWLSDIDGVTPTAVEEIVRWSSPV

ISQRRTVAEDAEPVKLAGQLLGPGDKVLMFYGAANRDPNYFVHPEGFDVR

RSPNPHVGYGGPGPHFCLGAHLARLEISVMFRELLTRIPDIHATGEPARL

KSPLINGIKRLPVAFTPGAR

>CYP1207A12(2563542371)*Salinispora pacifica* CNY498

MITVPLTDRYLLDEDLVADPYPYLAELRVAEPVRWSPVHRAWLVTGYEAA

ASCYAEPAISADRIGPMLAQTPLELLSPEAARAFTIMAGWMVFVDPPEHR

RLRSVFRGAFGARQVRRNRPMVQEAVTRLTARTGTVGDTVDLVAEFARPL

PATVAASWMGVPVEDTAVFQRWAIQVGDLALGTVQSPEEHERSQQALLDL

FDYLRMLVRTRRATPGEDLISAALANGLVGDSVSEDEFVAMLTHVAFAGG

ETTSNLIAVGTWNLLRHPDQLALLRAEPELAPVAIEELLRFDGPSKMSIR

HVKNDVELAGQRLRAGQRLYVVTAGANRDPDYFDHPNELDVRRHPNPHLG

FGQGGHFCLGAPLARLVAGAALTDLLAAAPGLALATAEVRWQPSLLNRSL

QALPVRF

>CYP105AB28(2563543200)*Salinispora pacifica* CNY498

MTETASTTTPGISSTTTSGPASGEVTDAEFPLERGCPFSTPTEYEQIREH

SPLAKVRLTTGREAWWIAGHELGRAVLADRRFSSDRRRDNFPFVSTDPET

RKQLQDQPTSMIGMDGAEHAQARRALMGEFTVRRMAGLRPRIQQIVDQHI

DEMLSSDQRTADLVEALSLPVPSLVICELLGVPYADHDFFQARSGPLIRH

TTPTEVRLRIQKELNTYLGALIDRKVADPTDDLLSRQIAKHHAAGTFDRT

SLVSMAFLLLIAGHETTANMISLGVVGLLQHPDQLAMIKEDPEKTAPAVE

ELLRYFTIADTVTARVATEDVQLGDTTINAGDGVVISGLAADHDPTVFTD

PDRLDLERGARHHVAFGFGPHQCIGQTLARMELQIVFDTLFRRIPTLRLA

APLDDIPFKSDAFVYGAERLPVAW

>CYP1005A6(2563544025)*Salinispora pacifica* CNY498

VSAVLFRSWTKTAGTHWPAVTRVADQQGTEHLVVTEHALVRQVLTDQVTY

RPDNALDAVTPIPVAALRVLAGHRFRLPPTLANNGGVSHPAIRALVADAL

HPAKVAAQRPWLTELVAERVAAIRATLDSGGSADLHAELSADLPLLVLAR

LVELPDAPVSAVKQFARAALELFWAPLDADRQLALADEVGRFHQVLREFA

DTGGGLAAALRATGHPPDVLVGALFFLLVAGQETTSQFLTLLSHRLAGEP

TVRAALRDGSVSVPNVVEEGLRLEPPIVTWRRVAAVDSTLGGTAVPAGTS

VLLWLARAGRDPAIVSAPDEFRPGQRGSRRHLAFGAGAHRCLGDQLARME

AAVVVERVSPLLDGVTVVRAPWYPDNLTFRMPDAFVIRRGPAGAAER

>CYP244A10(2563544348)*Salinispora pacifica* CNY498

MQDTAQANLAEAPEVRMPIELRPTDCLPELLAAARVAPVVRTPYLDQHAW

VVCDRELVKQALTHPKLGKDVALAPDWMRQPGQMVTAMPPPEYARMMVMS

DGEHHARIRRIHAPVLSPRNTERWSERVAALVDGFLDNLDSADGTEVNLI

TDYTHKIPLAFTAEMLGLPPGAERRLHDITEVMLYSADYALRQQAVGELF

EAVQEWVRDPAGLREGVITGLLASADGPDATVTKGEAIVWTLSLIINGYE

TTGSLISAALYEALRRPARERPHTDEAVAAWIEETLRVQPPVPHTTWRFA

LADLDLGGYLIPRGAPVQISLAAANLDPDEDADSFDAQRRGRGHLSFGLG

AHYCIGAPLARVQTKIALRGFLRRFPQARLSPDTAPRWESEWMIRRMSVL

PALLA

>CYP107AY9(2563544368)*Salinispora pacifica* CNY498

MSQDQPTRAELAPIPRSGARIGPEYDQLRKTGDVHQVLLPDTSMAWLVTS

PDLVSRALADPRLALNRKHSRGGWSGFALPPALDANLLNLDAPDHTRLRR

LVGPAFSPQRVAALRPRIQRTAEELAETMVATGSPVDLVTGYCTPLSVQV

IADLLGVPDAGRTDLRAWTDTMLTSYPPDRDAIRQAVVELHGYVVNLIEA

KQQRPGDDLLSALVATEQEGDRLTRDELTSLAFLILFAGYENTANLIAST

VLRLLDHGSLRGVRVSEAIEETLRLEPPAPAAIRRFPTEEMTIGGATIPA

GDTVLLSIAAATRGADGNPARLAFGNGPHYCLGAALARVEAEEAITVLAR

RLPGLALAVPGAPVRWRPTFRTHGPAELLVAW

>CYP107FH3(2563544564)*Salinispora pacifica* CNY498

VPIELDEAFVQDPYAVYEKLRAEGPAHRVRMPPGVPLIGGLPVWLITGYD

AVRAALADSRLSTDLHRIDGLFAQKDPDRSHRGGFSSALASHMMHTDPPD

HTRLRKLVSKAFTRRAIEALRPRIQQTTDELTTELTGHDTVDLLDAFAFP

LPIRVICLLLGVPVAEQENFRSWSRALVSGHSPEAAATAATEVAAYLGDL

VERKRHATTDDVLTALVAAHDVDDRLTHTELVSTAYLLFVAGFETTLNAL

GNGTLHLMLHRDQWTALRADRALLDNAVEEFLRLESPLKHATFRCATESL

RIGDAEIAAGDFVLLAIASANRDPRRFPDPHTLDVRRPAAGHLAFGHGIH

HCLGAPLARVEVRMAFDALLDAFPDMRLATDPAGLRWRNSTIIRGLDSLP

VHLNN

>CYP2054A3(2563544570)*Salinispora pacifica* CNY498

MSISADSEQVQGRPFDPYGAHRDDPYTFLAGLGVFYAPLLDAWCVTRRED

MVAVLRDDRSFSARDHNPRPAVALPDDVNQMFRTWRGAGAVAVGSLDPPA

HAKIRDVLNIGFTPARVRAFEPTMRAVAADLADRIGDAPEFDFIADFAVP

FALEVIGRRLGVPDDYLDRCRTWSEQRIELMMAQGDADHDRLREFARGLM

EFGEFARSLVRDRVADPRDDLISELLHDGKAGRTLTADEVAVQIPTLIFA

GHMTCAEALGTIFYQQLRSPGGWARVVDRTIPVGDLVEEGLRFDSPLAGM

YRTATRDVTVGGIRLTAGSRLLLLYGAAGRDSRAHACPAAFRPGDGSSGH

LAFGHGIHFCLGAGFARAELRVAVEVLAARMPDLGLAPGRPPRFRPVFPL

RALTELRVTRSGGGSGP

>CYP161N4(2563544575)*Salinispora pacifica* CNY498

VNTVAQLPFTQTHVLDVAPALRLLQSRGKVHRVRTPEGVPAWLVTGHAEV

QQLLDDDRLSRSDPGGRDGGTALLNKLLGPLADDHPRLRSLLEPQFTPER

LEPLRAVVEKLTEQHLDELATRTPPVDLRPTLAMSLPILVLCEWLGVPAE

DKGRFSVWTQDAAGVQDPERSQRGLAELFGYCRQLVAAKRQDPGDDVISR

LIATAGIGDTEVVALTALLLFGGYETTVARIGTGVLLLLTNPDQWAAVRA

DPALVPATVDELLRRSMPNPHNGGMPRFAVTGFEIDGAAIRAGDLVLLNI

IAANHDETAFPDPDRLDITRPTAGSLAFGYGRHSCVGAPLARMVLRVALS

RLITRFPDLRLAVGVDELKLRHETLVGGLVELPVTWGPR

>CYP107E37(2563545402)*Salinispora pacifica* CNY498

VTIDQEIREYPFRESRGIGIDPTYELLRRTEPLARVQLPYGEVSWLVTRY

EDVKTVLTDPRFSRAAAQGKDQPRTRAEMTYEGIIGLDPPDHTRLRRLAG

KALTARRVNAIRADAQRIANEYVDEMIAKGSPGDLVELFALPYPVTVICE

LLGVPFEDRAQFRIWTEGLTSTSEQLMVYAEQLFDYMGKLVAQRRAEPTD

DLLGALVKARDEGDRLTEQELLSIAGVGLLLTGVETVSTHIPNFVYALLT

HPELMAQLRADRSLVPAAVEELLRMIPLNPAAMFPRYAVEDVTLSGITVR

AGQPVLVSLPGANRDPEVFENPETFDFTRDQNPHVAFGHGPHHCLGAQLA

RMELQVALHTVLDRFPDLRLADGDEGVSWKSGLLVRGPSRLLVGW

>CYP154AJ2(2517974478)*Salinispora pacifica* CNT584

VDDESLFVIDPAGADIHGEGARLRASGPITRVLLPGGVEAWSVTGYHAAR

QVLADARFAKNARQHWPAYVNGDLGPDFPLIAWARMDNMSTADGESHARQ

RRLVAGAFSPQRIAALQPRVERAVARALDELAAQAAERPGEPIDLKERYA

HPVAARVIGELLGVPDGDPDGILDRAGYIESTPQRAAEEFALLRSKIETL

VAAKRRAPGEDLISDLIAAQPSGCPVAGAGAGSDAELVGLAQLMLNTGAE

PARNLITNTVLALLTRPGQRAQVMSGEVSWGDVVEETLRTDAPVAHLPFR

FATEDVTIAGTTISRGDPVLVGYAATGRDPELHGATAGEFDARRADKRHL

AFGHGVHRCVGPALTRMEVRIAVEALFTRFPGVRLAVEPKELVGQGTFVM

NGRRELPVQGLCAQP

>CYP211C9(2517974539)*Salinispora pacifica* CNT584

VPDIEGLLARLYSARGRQDPYPVYADLHAQAAIAALRLGPGRQRVAAVAV

GYDLVAAVLRDPEWCKQPPPGWRDQEILRILQSSMMFINPPDHGRMRHVF

AGTFTPRRLGALEPVINRVTDELLDRMADAGPGEVDFVAEFAYPLPARVM

AEFIGIPTTELAWYRERVDRVDAFLDVAGKTPERLAAANAAGAELRAFYR

ELLARRRRTPGEDLISGLVEAVDAGGVELTEDELISNLIVLFNASFVTTV

YLLSNGLPVLLAHPEVAAALADNPELAAGAVDEILRLQTPVHLLARAAPR

DTVLGGVSIPQGQNVLLLIAAANRDPAHFPDPDRFDPRRSGPSSLAFGLG

LHYCLGAAVSRLEGRLALPRLLSRFPRLRILEQPVYSGSLFLRGIDKLSV

SPGGKEYP

>CYP125A65(2517974672)*Salinispora pacifica* CNT584

MTEPRIPVGFDFTDPTVLERRVPREEFAELRRTAPVWWNVQPRGSAGFDD

DGYWVVTRYADVMAVSRDSETYSTRENTAIARFQPGTTRADLEMQRVIML

NVDPPEHTKLRAIVSRGFTPRAIHALRGSLAERAERIVRDAAVRGTGDFV

TDVACELPLQAIAELIGVPQHHRRKVFDWSNQLIGYDDPAYGVDPMAAAA

ELLGYAMEMADERQRNPSDDLVTKLVNAQIDGEHLTTDEFGFFVMLLAVA

GNETTRNAITHGMLAFLEHPEQWELFKAERPRSAVEEIIRWATPVNVFQR

TALVDTTLGGQAISAGQRVALFYGSANFDESVFEEPERFDITRSPNPHLG

FGGSGVHFCLGANLARLEIELIFNSIADHMPDIRKVADPQRLRSGWINGI

REMPVRYR

>CYP244A5(2517974834)*Salinispora pacifica* CNT584

MSATTNAELHEAPETAMPVDPGLFDCMPDLIAAARIAPVVRIPYLGRHAW

VVCDRELVKQALTHPKMGKDITLVPEWMRQPGLMVTAQPPPEYARAMIMS

DGENHARIRRIHAPVLSPRNTERWGEQVADKVEGFLDELSQAAAGSSAEV

DVVTNYTHKIPLAFISEMLGLPPVAEHRLRSITDIMLYSSDYAARREAIG

GLFGAVEEWVRNPAGLRDGVITGLLAGSDGPGAAVTEGEVIVWTLGMIIT

GYETTGSLISTSLYEALRRPPHERPRTDEDITAWIEETLRVHPPFPHPTW

RFPLEDIELGGYLIPKGAPVQVSIAAANRQPGEGADSFDAERRGHGHLSF

GLGMHYCIGAPLVRLEAKIAVRGFLRRFPQARLSADTAVQWESEWMIRRM

SFLPAVLS

>CYP245A11(2517974838)*Salinispora pacifica* CNT584

MSSTTLPRFTLTGWTREDIVNPYPVYRRYREVAAVHRGEAGGDAPETFYV

FSYDEVAQVLSSSCFGRGRSLDATAASVPVPADQKALRAVVENWLVFMDP

PRHTELRSLLNRSFSPRIVTGLRPRIARIAQELLSRLGQQVETDLVEGFA

APLPILVIAELLGIPAERHGWLRTNAIALQEASSSRARRDTDGYARAEAA

AQEFTEYFREQVCLRRGSTGDDLLTILANAQLRGAPVSLDAVVGTAVHLL

TAGHETTTNSLAKAVLALQAHPAVLEELRGADRLTADSIEEFLRYDPPVQ

AVTRWTHQDTTLGGWDVPRGSRVVALLGSANRDPARFPLPDALDVRRPAD

RHLGFGLGIHYCLGATLARAELEIGLQTLLNGLPTLGYPAQYVDYADDMV

FHGPSRLILVNPGERFC

>CYP107AW6(2517974921)*Salinispora pacifica* CNT584

VETVTGTSAPPPVPYIADPYPTLARIRANGPVSILHSDEGVPMWVIARYR

EVRTALADPRFGQDARRAQALADNRVAGVTLGGDIVHMLNSDPPDHTRLR

RHVQGAFTARRVAAMRPLVERITTSLLDGLAGRKTVDLVQDLAFPLPMLV

ICELLGFPAEERNAYRSWSTAILTHDDDPAVFATALREMTDYIAVQLRLR

RSRPGDDLLTELLTARDAGQLTDDEIVGMVFLLLIGGHETTVNLLGTATL

ALLRNPDQHRWLLANQHALPEAIDEFLRYESPVAMATLRFTTTPVTVDDV

VIPAGELVLVSLGGANRDPDRFPAADRLILDRRDTGHLAFGHGLHRCLGA

FLGKLEGEVALGALLRRHPKLALAAEVRQLQWRDTIMLRGLESLPVSLHG

>CYP107AX10(2517974934)*Salinispora pacifica* CNT584

MTAQPAPVFDQRLLRDPHRRYNALRDQAPVHHVRTPDGAPAWLVTRYDDV

RAAFTDPRLSVDKRFSGTDGEHGSSLPPELDAHLLNRDPPDHTRLRRLAA

AAFTPRRVADLRPAVERTVSTLLDGLAGNDHAELIGSLASPLPLQVMHEL

LGLPTQTSVDFRTWTNTLLSADANQPAQSRSAMANMRRFLVEQVAHKRAR

PGDDLLTGLLCVREDDDGLTDDELVAMLFLLMFAGYDNTAALIGNVIHAL

LTNAELAEAVRTRSLALDELVDGVLRWNPSFPLAVRRFAREPITIAGQAI

PAGDRIWLCLASANRDPAHFTEPDEIGIADMRRPHLSFGHGIHYCLGAPL

ARLQTTVAVASLFDRFPGIRLAMPVQDIQWRESFRLRGLVALPVFL

>CYP105AB27(2517975149)*Salinispora pacifica* CNT584

MTETASITTPGTSSTTTSEPASGQVTDAEFPVERGCPFSTPVEYEQIRDH

SPLAKVRLTTGREAWWIAGHELGRAVLADRRFSSDRRRDNFPFVSTDPET

RKQLQDQPTSMIGMDGAEHAQARRALMGEFTVRRMAGLRPRIQQIVDQHI

DEMLSSDQRTADLVDALSLPVPSLVICELLGVPYADHDFFQARSGPLIRH

TTPTEVRLRIQEELNTYLGALIDRKVTEPTDDLLSRQIAKQRAAGTFDRT

SLVSMAFLLLIAGHETTANMISLGVVGLLQHPDQLAMIKDDPEKTPPAVE

ELLRYFTIADTVTARVATEDVQLGGTTINAGDGVVISGLAADHDPTVFTD

PDRLDLERGARHHVAFGFGPHQCIGQTLARMELQIVFDTLFRRIPTLRLA

APLDDIPFKSDAFVYGAEKLPVAW

>CYP211B16(2517976031)*Salinispora pacifica* CNT584

MDASEAVALLMSPPGRRDPYPTYERLRAHGPVVATAAGFFVVTGYAEADT

VLRNPRFAVMDDEERDGVFPHWQDSPAMMSISQSMIRANPPDHSRMRRLA

AGVFTPRRVAALREVVAAQADELIDEMIRAGRGGAPVDFLGGFAYPLPVT

VICALLGVPAADWAQFRRWAGDLTGVLEPEITPQELAVADAGATELRDYF

TELIAQRRRDPADDLTTALVQTHDADGDRLSGEELLANLVLLLVAGFETT

TNLLGNGLFVLLTHPESATALRDQPELAPSYVDELLRYDSPVQLTSRTVR

ESVPLAGVELPAGSWLLVLLGAANRDPARFTDPARFDPGRAQSPPLSFGA

GAHYCLGAGLARLEAQVAFPLLLRRLPELALAGEPVRRSRLTLRGYETLP

VTVGAVAADHGTPAGAALGTP

>CYP105BL3(2517976240)*Salinispora pacifica* CNT584

MSSHSAAAPDPETATPLHTLAPELTFPQFERATPFDPPEAYTELSGRCPV

APVRMADGKPSWLITSFEGVRAALSDPRLSSDMSHPGFPNRTGKPVDDLL

KDTLGAMDGERHRYYRRMLTGELTVRRAKAMRPVITQITDEALDQLAAAG

PGADLVTHVALVVPSRVACHLVGIPLSEYELFTGMAAKLMDSTSSADQIA

ALQDMVSYFDTLVTDREHHDRDDLLGHMVRRYLATGELDREMLIRLAWTT

MAAGQETTAHMIGLGVAALLRHPDQLELLRREPHLMPGAVDELMRYLPMI

QFGIPRVAMDDVEVDGQTVTAGEGVVALPPLANRDPAVFERPDELDVRRN

ARQHLSFGYGPHQCPAHALARLELEVVYGRLLERFPTLRLADSDADLKVQ

DEDIMYRVSELAVAW

>CYP161T1(2517976498)*Salinispora pacifica* CNT584

VTTQVFRTKTPIGHPAWLVTDYESVRDLLSDPRLRLTHERPGEAARLSNS

VIFGRPQPSSETEAADHTRMRRKLAQWFSARRLATFRPRLRELVDGLLDE

LAATPKPADFHQVVSFPLPALVICELLGVPYADREQFRRWSDDAADMYDQ

ERSVGGYQALRDYIAELAQVRLRMPGTDLISDLVESHRTEPEGFTLDQVV

ELGLTLLFAGHETTVTAIDNGAVLLATHPEQRDRLAGDPSLLDAAVEEIL

RGSVPRMNFFARPDDTDPAGALPRWANTDLVIGDVTVRAGELVLLGLAHA

NVDERFFAKSARFDVGRVPNRHLTFGIGPHFCAGAPLARLELQELFVALL

ERFPGLRLAVPAERLEPRANLLTGGMTRVPVTW

>CYP208A27(2517976903)*Salinispora pacifica* CNT584

MTVITKRGGRVPPGPPVTAGLRLLLALGRDRLGMLTSAATEYGDVARLPV

GPKRLYFFNHPDHAKHVLADNHANYAKGIGLVNARRALGDGLLTSDGELW

RRQRRVIQPAFLNRRLTQYAGVVGQEATRLAERLATRVDGPPVDVLDEMT

RLTLGVLGRTLLDAELTGFHGVGESFAAVQDQAMFELETLSAVPTWIPLR

RQRRFRRARRHLQEVVDVLAAERGQDVEGRDDVLSRLILSTRAEADPALG

RQRLRDELVTLLLAGHETTASTLGWSLYLLDKHPQLRERVRHEATSVLGD

RVPGYADLHQLQYTAMVVQEAIRLYPPVWILTRKARAEDEVGGYRVPAGA

DLLICPYTLHRHPRFWEEPGRFDPERFDPARLANRPRYAYIPFGAGPRFC

VGNNLGMLEATLVLAVLLRDLRLEGLPGRAVVPEPMLSLRVRGGLPMMVR

RAD

>CYP1005A8(2517977568)*Salinispora pacifica* CNT584

VSAVLFRSWTKTAGTHWPPVTRVADQQGTEHLVVTEHELVRQVLTDQVTY

RPDNALDAVTPIPVPALRVLAGHRFRLPPTLANNGGVSHPAIRALVADAL

HPAKVAAQRPWLTKLVAERVAAIGATLDSGGSADLHAELSADLPLLVLAR

LVELPDAPVSAVKQFARAALELFWAPLDADRQLALADEVGRFHQVLREFA

DTGGGLAAALRATGHPPDVLVGALFFLLVAGQETTSQFLTLLLHRLAGEP

TVRAALRDGSVSVANVVEEGLRLEPPIVTWRRVAAVDSTLGGTAVPAGTS

MLLWLARAGRDPAIVPAPEEFRPGQRGSRRHLAFGAGAHRCLGDQLARME

AAVVVEQATPLLDGTSVVRAPWYPDNLTFRMPDAFVIRR

>CYP161N4(2517977794)*Salinispora pacifica* CNT584

VNTVAQLPFTQTHVLDVAPALRLLQSRGKVHRVRTPEGVPAWLVTGHAEV

QQLLDDDRLSRSDPGGRDGGTALLNKLLGPLADDHPRLRSLLEPQFTPER

LEPLRAIVEKLTEQHLDELATRTPPVDLRPALAMSLPILVLCEWLGVPAE

DKDRFSAWTQDAAGVQDPERSQRGLAELFGYCRQLVAAKRQDPGDDVISR

LIATAGIGDTEVVALTALLLFGGYETTVARIGTGVLLLLTNPDQWAAVRA

DPALMPATVDELLRRSMPNPHNGGMPRFALTGFEIDGAAIRAGDLVLLNI

IAANHDETAFPDPDRLDVTRPTAGSLAFGYGRHSCVGAPLARMVLRVTLS

RLITRFPDLCLAVGVDELKLRHETLVGGLVELPVTWGPR

>CYP205A3(2517977799)*Salinispora pacifica* CNT584

MPISADSEQVQGRPFDPYGAHRDDPYAFLAGLGVFYAPLLDAWCVTRRED

MVAVLRDDRSFSARDHNPRPAVALPDDVNQMFRTWRGAGAVAVGSLDPPA

HAKIRDVLNIGFTPARVRAFEPTMRAVATDLADRIGDAPEFDFIADFAVP

FALEVIGRRLGVPDDYLDRCRTWSEQRIELMMAQGDADHDRLREFARGLM

EFGEFARSLVRDRVADPRDDLISELLHDGKAGRTLTADEVAVQIPTLIFA

GHMTCAEALGTIFYQQLRSPGGWARVVDRTIPVGDLVEEGLRFDSPLAGM

YRTATRDVTVGGIRLTAGSRLLLLYGAAGRDSRAHACPAAFRPGDGSSGH

LAFGHGIHFCLGAGFARAELRVAVEVLATRMPDLGLAPGRPPRFRPVFPL

RALTELRVTRSGGGCGP

>CYP107FH3(2517977804)*Salinispora pacifica* CNT584

VPIELDEAFVQDPYAVYERLRAEGPAHRVRMPPGVPLIGGLPVWLITGYD

AVRAALADSRLSTDLHRIDGLFAQKDPDRSHRGGFSSALASHMMHTDPPD

HTRLRKLVSQAFTRRAIEALRPRIQQTTDELIADLAGHDTVDLLDAFAFP

LPIRVICLLLGVPVAEQENFRSWSRALVSGHSPEAAATAATEVAAYLGDL

VERKRQATTDDVLTALVTAHDVDDRLTHTELVSTAYLLFIAGFETTLNAL

GNGTLHLMLHRDQWTALRADRALLDNAVEEFLRLESPLKHATFRCATESL

RIGDAEIAAGDFVLLAIASANRDPRRFPDPHTLDVRRPAAGHLAFGHGIH

HCLGAPLARVEVRMAFDALLDAFPDLRLATDPAGLRWRNSTIIRGLDSLP

VHLNN

>CYP107AY8(2517978692)*Salinispora pacifica* CNT584

MRAEPAPIPRSGARLGQEYDQLRKTGDVHQVLLPDTSLAWLVTSPELVSR

ALADPRLALNRKHSRGGWSGFALPPALDANLLNLDAPDHTRLRRLVGPAF

SPQRVAALRPRIQRVAEELADTVVATGSPVDLVTGYCTPLSVQVIADLLG

VPEAGRTDLRAWTDTMLTSYPPDRDAIRQAVVELHGYVVNLIEAKQQRPG

DDLLSALVATEQEGDRLTRDELTSLAFLILFAGYENTANLIASTVLRLLD

HGSLRGVRASEEIEETLRLEPPAPAAIRRFPTEEMTIGGATIPAGDTVLL

SIAAATRGTDEHSARLAFGNGPHFCLGAALARVEAEEAITVLARRLPSLA

LAVPGAPVRWRPTFRTHGPAELLVTW

>CYP211B14(2563445754)*Salinispora pacifica* CNY703

MDASEAVALLMSPPGRLDPYPTYERLRAHGPVVPTAAGFFVVTGYTEADA

VLRNPRFGVMDDEERDGVFPHWQDSPAMMSISQSMIRANPPDHSRMRRLA

AGVFTPRRVAALREVVAAQADELIDEMIRAGRGGAPVDFLGSFAYPLPVT

VICALLGVPAADWAQFRRWASDLTGVLEPEITPQELAIADAGATELRDYF

TELIAQRRRDPTDDLTTALVQAHDADGDRLSGEELLANLVLLLVAGFETT

TNLLGNGLFVLLTHPESATALRDQPELAPGYVDELLRYDSPVQLTTRTVR

ESVPLAGVELPAGSWLLVLLGAANRDPARFTDPARFDPGRAQSPPLSFGA

GAHYCLGAGLARLEAQVAFPLLLRRLPELALAGEPIRRNRLTLRGYETLP

VTVGAVAVDHGTPAGAALGTP

>CYP245A11(2563445926)*Salinispora pacifica* CNY703

MSSTTLPRFTLTGWNREDIVNPYPVYRRYREVAAVHRGEAGGDAPETFYV

FSYDQVAQVLSSSCFGRGRSLDATAASVPVPADQKALRAVVENWLVFMDP

PRHTELRSLLNRSFSPRIVTGLRPRIARIAQELLSRLGRQVETDLVEGFA

APLPILVIAELLGIPAERHGWLRTNALALQEASSSRARRDTAGYARAEAA

AQEFTEYFREQVRLRRGSAGDDLLTILANAQLRGAPVSLDAVVGTGVHLL

TAGHETTTNSLAKAVLALQAHPAVLEELRGADGLTADSIEELLRYDPPVQ

AVTRWTHQDTTLGGWEVPRGSRVVALLGSANRDPARFPLPDALDVHRPAD

RQLGFGLGIHYCLGATLARAELEIGLQTLLKGLPTLGYPAQYVDYADDMV

FHGPSRLILVNPGERFCQ

>CYP244A5(2563445930)*Salinispora pacifica* CNY703

MSATTNAELGEAPETSMPVDPGLFDCMPDLIAAARIAPVVRIPYLGRHAW

VVCDRELVKQALTHPKMGKDITLVPEWMRQPGLMVTAQPPPEYARAMIMS

DGENHARIRRIHAPVLSPRNTERWGEQVAAKVEGFLDELSKAAAGSNAEV

DVVTNYTHKIPLAFISEMLGLPPAAEHRLRSITDIMLYSSDYAARREAIG

GLFGAVEEWVRNPDGLRDGVITGLLAGSDGPGAAVTEGEVIVWTLGMIIT

GYETTGSLISTSLYEALRRPPHERPRTDEDITAWIEETLRVHPPFPHPTW

RFPLEDIELGGYLIPKGAPVQVSIAAANRQPGEGADSFDTERRGHGHLSF

GLGMHYCIGAPLVRLEAKIAVRGFLRRFPQARLSADTAVQWESEWMIRRM

SFLPAVLS

>CYP208A26(2563446796)*Salinispora pacifica* CNY703

MTVIADQGGRVPPGPPVTAGLRLLLALGRDRLGMLTSAATEYGDVARLPV

GPKRLYFFNHPDHAKHVLADNHANYAKGIGLVHARRALGDGLLTSDGELW

RKQRRVIQPAFLNRRLAQYAGVVGQEATRLAERLATRVDGPPVDVLDEMT

RLTLGVLGRTLLDAELTGFHGVGESFAAVQDQAMFELETLSAVPTWIPLR

RQRRFRRARRHLQEVVDVLAAERGQNVEGRDDVLSRLILSIRAEADPALG

RQRLRDELVTLLLAGHETTASTLGWSLYLLDQHPQLRERVRHEATSVLGD

RVPGYADLHQLQYTAMVVQEAIRLYPPVWILTRKARAEDEVGGYRVPAGA

DLLICPYTLHRHPRFWEEPGRFDPERFDPARLTNRPRYAYIPFGAGPRFC

VGNNLGMLEATLVLAVLLRDLRLEGLPGRAVVPEPMLSLRVRGGLPMMVR

RAD

>CYP125A66(2563447121)*Salinispora pacifica* CNY703

MTEPRIPVGFDFTDPAVLERRVPREEFAELRRTAPVWWNAQPKGSAGFDD

DGYWVVTRYADVMAVSRDSETYSTRENTAIARFQPGTTQADREMQRVIMI

NVDPPEHTKLRAIVSRGFTPRAINALRGSLGERAERIVRDAAGRGAGDFV

TDVACELPLQAIAELIGVPQHHRRKVFDWSNQLIGYDDPAYGVDPLAASA

ELLAYAMEMAHERQRNPSDDLVTKLVNAQIDGEHLTADEFGFFVMLLAVA

GNETTRNAITHGMLAFLENPEQWELFKAERPRSAVEEIIRWATPVNVFQR

TALVDTTLAGQAISAGQRVALFYGSANFDESVFEEPERFDITRSPNPHLG

FGGSGVHFCLGANLARLEIELIFNSIADHMPDIRKVADPQRLRSGWINGI

RELPVQYH

>CYP105BL6(2563447739)*Salinispora pacifica* CNY703

MSSHPAAAPGPETATPLHTLAPELTFPQFERATPFDPPEAYTELSGRCPV

APVRMADGKPSWLITSFEGVRAALSDPRLSSDMSHPGFPNRTGKPVDDLL

KDTLGAMDGERHRYYRRMLTGELTVRRAKAMRPVITQITDEALDQLAAAG

PGADLVKHVALVVPSRVACHLVGIPLSDYELFTGMAAKLMEATSSADQIA

ALQDMVSYFDKLVTDREHHDRDDLLGHMVRRYLSTGELTREMLIRLAWTT

MAAGQETTAHMIGLGVAALLRHPDQLELLRREPHLMPGAVDELMRYLPMI

QFGIPRVAMDDVEVDGQTVTAGEGVVALPPLANRDPAVFERPDELDVRRN

ARQHLSFGYGPHQCPAHALARLELEVVYSRLLERFPTLRLADGDADLKVQ

DEDIMYNVSELAVAW

>CYP107AY7(2563447844)*Salinispora pacifica* CNY703

MRAEPAPIPRSGARLGQEYDQLRKTGDVHQVLLPDTSLAWLVTSTELVSR

ALADPRLALNRKHSRGGWSGFALPPALDANLLNLDAPDHTRLRRLVGPAF

SPQRVAALRPRIERTAEELVDTLVATGSPVDLVTGYCTPLSVQVIADLLG

VPEARRTDLRAWTDTMLTSYPPDRDAIRQAVVELHGYVVNLIEAKRQRPG

DDLLSALVATEQEGDRLTRDELTSLAFLILFAGYENTANLIASTVLRLLG

HGSLRGARASEAIEETLRLEPPAPAAIRRFPIEEMTIGGATIPAGDTVLL

SIAAATRGTDGNSARLAFGNGPHFCLGAALARVEAEEAITVLARRLPSLA

LAAPGAPVRWRPTFRTHGPAELLVTW

>CYP211C6(2563448227)*Salinispora pacifica* CNY703

VPDIEGLLARLYSAQGRQDPYPVYADLHAQAAIATLEPRPERQRVAAVAV

GYDLVAAVLRDPEWFKQPPPGWRDQEILRILQSSMMFINPPDHGRMRHVF

AGTFTPRRLGALEPVINRVTDELLDRMADAGPGEVDFVAEFAYPLPARVM

AEFIGIPATELAWYRERVDRVDAFLDVAGKTPERLAAANAAGAELRSFYR

ELLAHRRRTPGDDLISGLVEAVDAGGVELTEDELISNLVVLFNASFVTTV

YLLSNGLPVLLAHPEVAAALASSPELAAGAVDEILRLQTPVHLLARAAPR

DTVLGGVSIPQGQNVLLLIAAANRDPAHFPDPDRFDPRRSGPPSLAFGLG

LHYCLGAAVSRLEGRLALPRLLSRFPRLRILEQPVYSGSLFLRGIDKLSV

SPGGREHP

>CYP1005A8(2563449209)*Salinispora pacifica* CNY703

VSAVLFRSWTKTAGPHWPAVTRVADQQGTEHLVVTEHELVRQVLTDQVTY

RPDNALDAVTPIPVPALRVLAGHGFRLPPTLANNGGVSHPAIRALVADAL

HPAKVAAQRPWLTKLVAERVAAIGATLDSGGSADLHAELSADLPLLVLAR

LVELPDAPVSAVKQFARAALELFWAPLDADRQLALADEVGRFHQVLREFA

DTGGGLAAALRTTGHPPDVLVGALFFLLVAGQETTSQFLTLLLHRLAGEP

TVRAALRDGGVSVANVVEEGLRLEPPIVTWRRVAAVDSTLGGTAVPAGTS

VLLWLARAGRDPAIVPAPNEFRPGQRGSRRHLAFGAGAHRCLGDQLARME

AAVVVEQAAPLLDGISVVRAPWYPDNLTFRMPDAFVIRR

>CYP154M16(2563449289)*Salinispora pacifica* CNY703

MPDRCPVLDPSGRDIHAEADRLRAQAPAVKVELPGGVHAWSITSYDVVRR

LLLDRNVTKNARNHWPKFINDEIPPDWEMISWVAMDNMVTAYGKHLVRLR

RLIAKAFTVQRVETVRPQVEKLVDELLDGLAAETGEVVDLREKFCYPLPA

LLIADLIGMTEQQRAQTAKAMDLMVDTTVSPEQAQAILTGWRTAMDELIA

AKRREPGKDIASDLIAARDDENGGQLTDSELTDTIFAILGAGSETTINFL

DNAVTALVTHPGQLELVRSGRAGWDDVIDEVLRVQCPLASLPLRYAVTDI

ELDGVTIPQGDPILINYAAAGRDPALHGDTAGEFDVTRENKEHVSFGHGP

HYCLGAGIARLVATIGLSRLFERFPDLRLAVPAEELQPLPTFIMNGHRAL

PIRLVPAPAAATAV

>CYP107AW6(2563449613)*Salinispora pacifica* CNY703

VETVTGTSAPLPVPYIADPYPTLARIRANGPVSILHSDEGVPMWVIARYR

EVRAALADPRFGQDARRAQALADNRVAGVTLGGDIVHMLNSDPPDHTRLR

RHVQGAFTARRVAAMRPLVERITTSLLDGLAGRKTVDLVQDLAFPLPMLV

ICELLGFPAEERNAYRSWSTAILTHDDDPAVFATALREMTDYIAVQLRIR

RSRPGDDLLTELLAARDAGQLTDDEIVGMVFLLLIGGHETTVNLLGTATL

ALLRNPDQHRWLLANRHALPEAIDEFLRYESPVAMATLRFTTTPVTVDDV

VIPAGELVLVSLGGANRDPDRFPDADRLILDRRDTGHLAFGHGLHRCLGA

FLGKLEGEVALGALLRRHPKLALATEVRQLQWRDTIMLRGLESLPVSLHG

>CYP107AX9(2563449628)*Salinispora pacifica* CNY703

VTAQPAPVFDQRLLRDPHRRYNALRDQAPVHRVRTPDGAPAWLVTRYDDV

RAAFTDPRLSVDKRFSGTDGEHGSSLPPELDAHLLNRDPPDHTRLRRLAA

AAFTPRRVADLRPAVEKTVSTLLDGLAGNDHAELIDSLASPLPLQVMHEL

LGLPTQTSVDFRTWTNTLLSADANQPAQSRSAMANMRRFLIEQVAHKRAQ

PGDDLLTGLLCVREDDDGLTDDELVAMLFLLMFAGYDNTAALIGNVIHAL

LTNVELAEAVRTGSLAVDELVDGVLRWNPSFPLAVRRFAREPITIAGQTI

PAGDRIWLCLASANRDPAHFTEPDEIGIADMRRPHLSFGHGIHYCLGAPL

ARLQTTVAVASLFDRFPGIRLAVPVQDIQWRESFRLRGLVALPVSL

>CYP208A21(2563449639)*Salinispora pacifica* CNY703

MTTTSIERRRPPGPPRAAALSMLLTMSRDRLGMMTAAARAYGDAAWLPVG

HKALYFFNHPDYAKHVLTDNSDNYTKGIGLVHARRALGDGLLTSEGELWR

EQRRVIRPSFRSGRAPDQASVIAEEVASLVERLRARAGGPPVNVVTEFTG

LTLGVLGRTLLDVDLTALATVGDAFAAVQDQAMFELVTLSAVPTWIPLTR

QRRFRRARAELERIVDDLVARRGDVSGRDDVLSRLILSTGAEPDARVRRQ

RLRDELVTLLLAGHETTASTLGWTLYLIDRHPPVRERLRAEAAEVLGDRL

PAYRDLPDLRFTTMVVQEAMRLYPPVWLLPRRSRRADRVGPYWVPAGSDV

VVSPYTMHRHPGFWPEPDRFDPLRFDPRNAADRPRYAYLPFGAGPRVCVG

SNLGMTEAVIAVAMLCRELRLVRVPTHAAVPEPMLSLRIRGGLPMSVHLA

D

>CYP105AB34(2563450179)*Salinispora pacifica* CNY703

MTETASITTPGTSSTATSGPGSGEVTDTEFPVERGCPFSTPAEYEQIREH

SPLTKVRLTTGREAWWIAGHELARAVLADRRFSSDRRRDNFPFVSTDPET

RKQLQDQPTSMLGMDGAEHAQTRRALMGEFTVRRMAGLRPRIQQIVDQHI

DEMLSSEQRSADLVEALSLPVPSLVICELLGVPYADHDFFQARSGPLVRH

HTPSKVRVRIQEELNTYLGGLIDRKVADPTDDLLSRQIAKQHAAGTFDRT

SLVSMAFLLLIAGHETTANMISLGVVGLLQHPDQLAMIKNDPEKTPPAVE

ELLRYFTITDTVTARVATEDVQLGDTTITAGDGVVISGLAADHDPTVFTD

PNQIDLERGARHHVAFGFGPHQCIGQTLARLELQIVFDTLFRRIPTLRLA

APLDDIPFKSDAIIYGAEELPVAW

>CYP244A4(2550291184)*Salinispora arenicola* ATCC BAA-917

YLGRHAWVVCDRELVKQALTHPKMGKDIALVPEWMRQPGLMVTAQPDPEY

ARAMIMSDGENHARIRRIHAPVLSPRNTERWGERVADKVEGFLDELSRAG

SGGSTEVNVVTNYTHKIPLAFISEMLGLPPEAEHRLRGITDIMLYSSDYA

ARREAIGGLFGAVEDWVRNPADLRDGVITGLLAASDGPDAAVTEGEVIVW

TLGMIITGYETTGSLISTSLYEAIRRPPHERPKTDEDITAWIEETLRVHP

PFPHPTWRFPLEDIELGGYLIPKGAPVQVSIAAANRKPGEGADSFDAERR

GHGHLSFGLGMHYCIGAPLVRLEAQIAVRGFLQRFPRARLSAETAPQWES

EWMIRRMSVLPAVLS

>CYP208A12(2550291209)*Salinispora arenicola* ATCC BAA-917

MTLDTITPRVPLGPPRTAALRMLLVMKRDRLGMLTSAAARYGDASRLPVG

HKALWFFNHPRYAKHVLADNSANYHKGIGLVHARRALGDGLLTSEGDLWR

KQRKVIQPAFQSRRIAQQAGMIAEEAFALVERLRARAGAGPVELTAELTG

LTLGVLGRSLLDADLAGFDSIGDSFATVQDQAMFELETLNAVPMWIPLPR

QIRFRRARRKLQAVVDTLVDGRAGNLADRVDVLSRLILSARGEADPRVGR

ERLRDELVTLLLAGHETTASTLGWTLSLIDRHPGVWERLHAEAVEVLGDR

LPEYDDLRRLRYTVMVVEEAMRLFPPVWLLPRRALAPDTIGEYRVPANAD

VVISPYTLHRHPEFWPNPERFDPERFAPGQAADRPRYAYLPFGAGPRFCV

GNNLGMMEAVFVIALLCRHLRLTGVPGHRLVPEPMLSLRIRGGLPLVVRP

VS

>CYP107Q4(2550291692)*Salinispora arenicola* ATCC BAA-917

TAGHQTTTYLIGNSVILLLENPDQLARLKENPSMWPQAVRELQRLGPIQF

AQPRFPSEDIELGGVTIPRGAPVAPLLLAANTDPRRFPDPNKLIIDRLAV

GSEGHLGFGKGIHRCLGQHLAYQEAEVALQGLFTRFPDLSLAVPREEIPW

ILRPGFTRTRTLPLKLV

>CYP105CH1(2550291726)*Salinispora arenicola* ATCC BAA-917

VSSLPLPTYPKLRDPADPLLPPAEYLAIQSEKPIAKVLLPSGRPTWLITG

HALARQVLTEPCVSVDRRHPNFPYPVPNPDAVVAQVARWTYILLGDDPPL

HTERRRLLISEFTVRQAQAMRPRIQQLVDFHLEQLIAAGPGADFSKHFAM

KVPSAVICEMLGVPFADHDYFQERTALQLRRDVPVAAQKQAIDELLAYFE

QLIQEKSSHPGDDVLSRLIVSNRETEAFDHEALVALGLLLLVGGHETTAN

TLTLATATMLERPEIAEQLRTDPSLMPSAVEEFLRYFSVAVAVSRIATAD

LQVGGQLVRAGESMLLVLNTIARDGTVFPEPHRLDIRRNARNHLAFSHGI

HQCMGQNLARVEMQIALDTVLRRLPGLHLVTPFEELPFKYRHLVWGIEEL

RVAW

>CYP154M15(2550291987)*Salinispora arenicola* ATCC BAA-917

RGDKTHLSFGYGPHYCLGAGIARMVATIGLSMLFERFPDLSLAVPVTELK

RLPTFIMNGHQTLPVRLTAHAR

>CYP105W2(2550292427)*Salinispora arenicola* ATCC BAA-917

MTGYQDRPTGDQPGAPVPSGSTDPGIGAFPLPRRCPFSPPAEYARLRAEH

PVVRLPMLGGDTAWVVSRHADVRQVLSDPRMSADRRRPGFPKFAPTTEGQ

RQASFANFRPPLNWLDPPEHAICRRQIVDEFSVRRVRQSRALVERVVDTH

LDALTAAAPGADLVSTFAYPVPSQVICEVLGVPYGEHEFFERRSTLMFRR

STPADERARCAREIRDFLDVVVTDREHRPGDDVLSRLLYRQRRA

>CYP1051A1(2550292648)*Salinispora arenicola* ATCC BAA-917

MATDAAITRARTVPAWKALPAAVRDTHRALVDVGNWSDGDVVRVSLGVSR

PYLVTNPAHVQEVLHERAAIYPRGDDTALWRSVRKLVGDGILAEGDAWAA

SRRVLAPMFRPARINAMVDTMADAIAGAVDGLHGAATAGTPIDVGRELSR

IVCSAIMRVFFADRITVRDALRIMKAQETIVTAMAPRILAPLVPWWIPMP

GDRRFRAAVRSIDDILLPVLRQAQRQPDDGDDLLSRLVRARADDGQALSE

KRMRDDLVSMVAVTTETSTVVLTWLWPLLANHPDVANRLYDEIDRVVGGG

PVRGDHLAELTYTRMVLDELLRLYPAGWILPRRAATTDVLGGVRINKGAT

VILSPYVTQRMTAWWGPTAEAFDPERFAAGREAADGRHRYAYYPFGVGMH

RCLGEHLFNLEAILIVATLLSRFRFALTDTSMPGVKVAASTRPARTVEMV

LKPVAPVPAR

>CYP105BL2(2550292654)*Salinispora arenicola* ATCC BAA-917

MSSHSAAAPDPETATPLHTLAPELAFPQFERSAPFDPPQAYTELSGRCPV

APVSMADGKPSWLITSFEGVRTTLSDPHFSSDMSHPGFPNRTGKPVDDLL

KDTLGAMDGERHRYYRRMLTGELTVRRAKAMRPVITQITDEALDQLAAAG

PGADLVKHVAFVVPSRVACHLVGIPLSDYELFTGMAATLMDSTSSDDQFA

ALQNMVSYFDTLVTDREHHDRDDLLGHMVRRYLATGELTRDMLIRLAWTT

MAAGQETTAHMIGLGVAALLRHPDQLELLRREPHLLPGAVDELMRYLPLI

QFGIPRVAMDDVEVDGQTVTAGEGVVALPPLANRDPAVFERPDELDVRRN

ARQHLTFGYGPHQCPAHALARLELEVVYGRLLERFPTLRLADSDADLKVQ

DKDIMYRVSELAVTW

>CYP105J3(2550292749)*Salinispora arenicola* ATCC BAA-917

APGYRPLAVQRPLAQVTLYDGRRVWAVTTRDLARRLLVDPRISSDRTNPA

WPAIVPIVAAAVNDAQQKVLKIATALVGTDGPEHKAQRKMLIPSFTFRRM

NALRPMIQEIVDQQLDEMIKSGAPTDLIPAFASAVPVTVLYRLMGIPDDD

HGIFEKLSHQLLAGPNANEAYDQLMGYMSRLIAERRRNPGEGVLDDLLAQ

HGANDDADHDELVSTLVVQVAG

>CYP105W2(2550293871)*Salinispora arenicola* ATCC BAA-917

LLVAGHVTTSNMLALSVLALLTHPARLARLRAEPERFPAAVEELLRYFTV

VEAATARTATAEVTIGGVTIAAGEGVVALGQAANRDPRVFEHPDEFDPDR

DARAHLAFGHSRHICPGQHLARLEMEVALSRLFRRLPGLRLTMEVSDLPL

KEDSNIFGLYALPVAW

>CYP107Q4(2550294304)*Salinispora arenicola* ATCC BAA-917

RAWGDSMATMNGERIPTTLVKCIELARELIAKRRAEPQDDLVTALVQAQA

EDQNRVSDDEIIGILF

>CYP245A7(2550295164)*Salinispora arenicola* ATCC BAA-917

MPSATLPRFALTGWSRENIVNPYPVYQRYREVASVHRGEPGGDAPDTFYV

FSYDEVVQVLSSNCFGRGRSLDAAKASVPVPAEQKALRAIVENWLVFMDP

PRHTELRSLLNRSFSPRIVTELRPRIARIAQELLSRLGQQVDVDLVESFA

APLPILVISELLGIPEERRAWLRANALALQEASSSRAGRDVDGYARAEVA

AQEFTEYFREQVRLRRGRAGGDLITILANAQERGAPVSLDAIVGTCVHLL

TAGHETTTNSLAKAVLALREHPAVLDELRGAEGLTTDAVEEFLRYDPPVQ

AVTRWAHQD

>CYP107FS2(2550295471)*Salinispora arenicola* ATCC BAA-917

MPVPQGEQNLTTEVFADPKALFATLGSRQPLHRISLPDGMPAVLVTGNRE

ARQALSDPRLVRSITAAAPELHKYHPLASDDYALSRHMLFADPPDHGRMR

KLVSTAFTRRRVEQMRPRIQQITDDLIDVIAAKGEADLVETLALPLPIAV

ISEMLGVPFADRSEFERHAEVLTGINASSGFDAIIAAGRWFDEYLAGLVQ

QRRREPQDDLISGMLAAQDKGDRLTDVELRSNALLLLSAGFETTVNLIAN

GLLALLRHPEAMAALRSEPNLMTTAVDELLRYDSPVSCVTYHFAQEPVEI

GGFEIRSGEHVVIAAAAANHDPTVFAAPSRLDLRREGSGQILSFSHGIHF

CLGAPLARLEGEIAFGTVLRRLAGLRLAVPTDSLVWKASFVLHRLERLPV

TFTPDRDPNPIDSVHTV

>CYP105CN1(2550295472)*Salinispora arenicola* ATCC BAA-917

MAAPAPQATPSTTPHPPSYPLPRECPYRPSAGTAHLRDAGPVSTVRLYDG

RTAWLVTGAAEARALLADSRVSNRADFPNYPVMDERHLSMRATREMAREE

EGGFAAALFGMDPPEHTRQRQLLLPRFTVRQVAARRPAIQRIVDEHLDAM

EANGSPADLVSAFATPVPTMVVCTHLGVPYQDRTRFEPAVAGLFEPDRAD

AAMAELTAYLHQLIETKQSEPGDGVIDHLIANHLRPGAIDRAELVAIASA

ILVAGTVTTSSAIALGTLALLTAPGQYTALVDNPDLVPGAVNEILRYLSL

VEQLARVATEDIEIGGKLIRAGDGIIVSFAAGNLDPNVTTHPDRLDVALP

PTNHLAFSHGIHHCIGQNLALLELDIAFRALVSRFPTLRLAVPAEQLPTY

FAGDVPRLACLPVTW

>CYP105CP2(2550295474)*Salinispora arenicola* ATCC BAA-917

MTKSMPVQDLPAFPIPRECPYRPSAQHVSLRSGGPMAKVRLYNGRTAWLV

TDSAHARAVLSDYRRVSIKPYHGNYPLLNEEFEKVVDSGYADVLFGVDPP

EHTRQRQMIMPSFTLRRTAVLRPDIQRIVDDKLDEMMRHGAPGDLVTEFA

QPVPSMVISFLLGVPWEDHEEFETPAHKLFVPELAEEATTELGAYLERLI

QKKEQPGGTPGGTGLLDDLIRDHLRAGALSRDELVHIAMAMLVAGTDTTT

NVISLGTLALLDNPDQWAALRDNPDELIPGAVEEILRYTSLIEAFARVAV

SDIELNGAVIKEGEGILISSAGVNFDPALAPDPGRFDIRRPPRPSFSFSH

GIHRCPGDNLARLELEIAFRSLVTRMPNLRTAKPIDQIPSNNNDGTLQRL

YELPVVW

>CYP211B2(2550295749)*Salinispora arenicola* ATCC BAA-917

MDVSEAIAVLISPSGRLDPYPTYEQLRAHGPVSQTTAGLFVVTGYAEADM

VLRDPRFVVLDDDLRDDVFPHWQDSPAIKSIARSMLRTNPPDHSRIRRLA

AGAFTPRRVAAMREVVTAQADELVDEMIRAGRDGARVDFMDMFAYPLPVA

VICALLGVPAADRSRFRRWAGDLTGILEPEITPEELAGADAGADELRDYF

TGLIEQRRRAPADDLTTALVQAHDADGDRLSGEELLANLVVLLVAGFETT

TNLLGNGLVVLLTRPEAAAALRDEPDLAPGYVDELLRYDSPVQLTTRTVR

ESVSFAGTELPAGSWLLVLLGAANRDPRRFPDPARFDPGRAQSQPLSFGA

GPHYCLGAGLAR

>CYP1198B1(2550296193)*Salinispora arenicola* ATCC BAA-917

MSGELTDQRTAPGAGGNPLRSLMDHGIRANPYPLFGELREAGPTAVEDGS

VVLFGEYEHCSQILRHRDMGSDTSEAPSIKGFVVDDAERAGSSIFFMDQP

GHGRQRKLVSKSFTPRIVKSFGPQITHIVDGLFEDFRDKGELDVVTDLAY

PVSIGIICDLFGIPDDERDMLKEWSDDLALSTELPTLGAAIGVLNVFTRD

EINRFGSVAMAAHAYFADLIHRRRKNPGDDLVSSLLATESNGERLTRFEV

TSVLATLFVAAHESTTNLISGGILALLRNQDQMAVLRENPGLITNVVDES

LRYDPPVHLAARMARARTTIGGYDLDPGTIVVVLMAAGNRDPRAYENPDV

FDVNRKIRNVSLAFGAGAHFCIGSGLAKLEAEIAISAFAQRLKHPEVDES

SLEYRRHIVVRGLEHMKVSFQP

>CYP107EU1(2550296197)*Salinispora arenicola* ATCC BAA-917

MVNSDPPDHTRLRHLVGREFTGHRVEGLRPRIEEIVDDLLDGVAACGDEA

DLAETLARRLPIAVIGELLGVPEADRAEFFRWADTLYGGTASPEALGQAY

NAIVDYLGRLCDAKRDVPADDLLTALVQVSADEDRLSREELVSMALLLLV

AGHETTSKQISNGVLALLLNPEQLKLLKAQPARTAGAVEELLRFEGPSLS

ASLRFTTEPVEVAGVVIPEGEFVLLSLASGNRDPEKFPDPDRLDITRSTQ

GNLAMGHGIHHCVGAALARLELEIVLIRLVARFPQMQLAVEADDLEWLVN

SFFRAPLHLPVSLRR

>CYP125A41(2550296387)*Salinispora arenicola* ATCC BAA-917

MTEPRIPAGFDFTDPEVLAHRVPREEFAELRRTAPVWWNAQPRGSAGFDD

DGYWVVTRYADVMTVSRDSDTYSTRENTAIARLRPDTTREDIEMQRVIML

NVDPPEHTKLRAIVSRGFTPRAINALRGSLAERAEHIVRDAAVRGVGDFV

TDVACELPLQAIAELIGVPQHHRRKVFDWSNQLIGYDDPAYGTDPLTASA

ELLAYAMEMAEERQRSPSDDLVTKLVNAQIDGEHLTTDEFGFFVMLLAVA

GNETTRNAITHGMVAFLDNPEQWELFKAERPKSAVEEIIRWATPVNVFQR

TALVDTVLGGQAISAGQRVALFYGSANFDEAVFEDPERFDITRSPNPHLG

FGGSGAHFCLGANLARLEIELIFNSIADHLPDIRKVAAPQRLRSGWINGI

RQMPVRYR

>CYP211C1(2550296523)*Salinispora arenicola* ATCC BAA-917

VAVGYDLVGAVLRDPEWSKAPPPGWTEQEILRTLQTSMMFINPPDHGRMR

HVFAGTFTPRRLGALEPVVNRVADELLDRMADAGAGGLDFVAEFAYPLPA

RVMAEFIGIPETELDWYRERVDVIDAFLDVAGKTPQRLAAANAAGAELRA

FYGELLARRRRTPGEDLISGLVEAVDAGGVELTEDELVSNLIVLFNASFV

TTVYMLSNGLPVLLAHPEVAAALATDPV

>CYP105AB8(2550296688)*Salinispora arenicola* ATCC BAA-917

MTETASSRLTDTEFPVQRECPFAEPVEYEQIREQSSIAMVRLTGGGEAWW

ISGHEQGRAVLADRRFSSDRRKANFPFVSTDPAIRKRLHAQPLSLISMDG

AEHTQARRALIGEFTVRRLAALRPRIQQIVDQCIDEMLTTDQHCADLVKT

LSLPVPSLVICELLGVPYADHDFFQEHTATLVRRNTASEVRQHSIDELNA

YLGALIDRKLASPDDDLLGRQIARQHRDGTFDRSSMVSLAFLLLVAGHET

TANMISLGVVGLLQHPEQLAMIKDDPDKTPLAIEELLRFFTIVDSVTSRV

ATEDVRFGDTTINAGDGVVVSGLSADWDPTVFADPDRLDLERGARHHLAF

GFGPHQCLGQNLARLELQIVFDTLFHRIPTLRLAAPLDKIPFKTDAAIYG

ARELPVAW

>CYP154M15(2550296740)*Salinispora arenicola* ATCC BAA-917

MNDKCPFAALDVTGQDLHGEAARLREQGPAVLVELPGGVKAWSINRYEVI

RELLMDRRVTKNARKHWPAFIKGEIPPDWEMISWVAMDNMVTAYGKDHVR

LRKLVGRAFTQRRADALRPQVVALSTKLLDDLGATPPGEVVDLRERFAYP

LPAMLVASLIGMSEAAQAACSKVIDMMVHTNVSPEEAQAVLRGWRAAMAD

LIESKRRTPGEDITTDLIAAREEDGSRLSEAELADTIFAILGAGSETTIN

FFDNAITALLSRPGQLQLLRTGGVTWDDMIDEVLRVESPLAHLPLRYAVE

DI

>CYP245A7(2550296933)*Salinispora arenicola* ATCC BAA-917

GCDIPRGSRVVALLGSANRDPARFPSPDVLDVRRPADRHLSFGLGIHYCL

GATLARAELEIGLQALLDGVPTLGYGTQHVDYADDLVFHGPSRLVLVNLG

ERCT

>CYP248A2(2550297291)*Salinispora arenicola* ATCC BAA-917

VLADAVTAFDPTAVDVRRDPYPSYHWLLRHDPVHRGAHQVWYVSRFADVR

AVLGDERFARTGIRRFWTDLVGPGLLSQIVGDIILFQDEPDHGRLRGVVG

PAFSPSALRRLEPTIEATVNDLLRPARALGAMDVVA

>CYP248A2(2550297325)*Salinispora arenicola* ATCC BAA-917

GAANRDPGRYDRPDELDIRRDPVPSMSFGAGMRYCLGSYLARLQLRTALG

AMVALPDLRLVCNPNELAYQPRTMFRGLTRLPVAFTPAG

>CYP107Q4(2550297801)*Salinispora arenicola* ATCC BAA-917

MTTTAETSAETIDLFSPEVVADPFGWYARLREETGPTTGTLNIGTMMGGPEMWLVTRYEDVRQVLTDPRFLTNPPADSPLEDIRAGVFKRLDFPPDLIPW

MANLLNVSDGEDHTRLRKQVSYALTAHRIGKLRPRVEKITADLLDKLAED

GKDGSPVDLVEEYCYPLPVTVSLRAGRHRR

>CYP105G5(2550297802)*Salinispora arenicola* ATCC BAA-917

LTIETTETPPADDSLRAPLPRQFMQRDDPSKLPPALAALAEQSPVGRSTL

PDGDPFWMVSGYDEARAVLSDPRFSSDRFRYHPRFKKLSGQLGERLRNDK

ARAGSFINMDPPEHTRYRKLLTGQFTVRRMRQLTVRIEQIVTEQVDVMLA

EGNSADLVSAFAVPVPSLMICELLGVRYEDRTEFQRRAAGLLQTDLPIKQ

AVENLEAQRAFMQRLVTDKRRTPADDMISGLVHHAGAEPPLTDDELVGIA

TLLLFAGLDTTASMLGLGMFMLLQRPEQMAVLRDDPSRIGDAVEELLRYL

TVVSTGLFRFAKEDVVLGDEHIPAGSTVVVSLMAANRDGRHWPEPETLDV

TRVRSSHLAFGHGVHQCLGQQLARIELTVGITELLRRLPNVRLAVPPADV

PLRNDMITYGVHRLPILWDTP

>CYP154M16(2517964207)*Salinispora pacifica* CNT045

MPDRCPVLDPSGRDIHAEADRLRAQAPAVKVELPGGVHAWSITSYDVVKR

LLLDRNVTKNARNHWPKFINDEIPPDWEMISWVAMDNMVTAYGKHLVRLR

RLIAKAFTAQRVETVRPQVEKLVDELLDGLAAETGEVVDLREKFCYPLPA

LLIAELIGMTEQQRAQTAKAMDLMVDTTVSPDQAQAILTGWRTAMDELIA

AKRREPGKDIASDLIAARDDENGGQLTDSELTDTIFAILGAGSETTINFL

DNAVTALVTHPGQLELVRSGRAGWDDVIDEVLRVQCPLASLPLRYAVTDI

ELDGVTIPQGDPILINYAAAGRDPALHGDTAGEFDVFRENKEHVSFGHGP

HYCLGAGIARLVATIGLSRLFERFPDLRLAVPVEELQPLPTFIMNGHRAL

PVRLVPAPAAATAV

>CYP208A21(2517964219)*Salinispora pacifica* CNT045

MTTTSIDRRRPPGPPRAAALSMLLTMSRDRLGMMTAAARAYGDAAWLPVG

HKALYFFNHPDYAKHVLTDNSDNYTKGIGLVHARRALGDGLLTSEGELWR

EQRRVIRPSFRSGRAPDQASVIAEEVASLVERLRARAGGPPVNVVTEFTG

LTLGVLGRTLLDVDLTALATVGDAFAAVQDQAMFELVTLSAVPTWIPLTR

QRRFRRARAELERIVDDLVAGRGDVSGRDDVLSRLILSTGAEPDARVRRQ

RLRDELVTLLLAGHETTASTLGWTLYLIDRHPPVRERLRAEAVEVLGDRL

PAYRDLPDLRFTTMVVQEAMRLYPPVWLLPRRSRRADRVGPYRVPAGSDV

VVSPYTMHRHPGFWPEPDRFDPLRFDPRNAADRPRYAYLPFGAGPRVCVG

SNLGMMEAVIAVAMLCRELRLVRVPTHAAVPEPMLSLRIRGGLPMSVHLA

D

>CYP107AX9(2517964232)*Salinispora pacifica* CNT045

VTAQPAPVFDQRLLRDPHRRYNALRDQAPVHRVRTPDGAPAWLVTRYDDV

RAAFIDPRLSVDKRFSGTDGEHGSSLPPELDAHLLNRDPPDHTRLRRLAA

AAFTPRRVADLRPAVEKTVSTLLDGLAGNDHAELIGSLASPLPLQVMHEL

LGLPTQTSVDFRTWTNTLLSADANQPAQSRSAMANMRRFLIEQVAHKRAQ

PGDDLLTGLLCVREDDDGLTDDELVAMLFLLMFAGYDNTAALIGNVIHAL

LTNVELAEAVRTGSLAVDELVDGVLRWNPSFPLAVRRFAREPITIAGRTI

PAGDRIWLCLASANRDPAHFTEPDEIGIADMRRPHLSFGHGIHYCLGAPL

ARLQTTVAVASLFDRFPGVRLAVPVQDIQWRESFRLRGLVALPVSL

>CYP107AW6(2517964245)*Salinispora pacifica* CNT045

VETVTGTSAPPPVPYIADPYPTLARIRANGPVSILHSDEGVPMWVIARYR

EVRAALADPRFGQDARRAQALADNRVAGVTLGGDIVHMLNSDPPDHTRLR

RHVQGAFTARRVAAMRPLVERITTSLLDGLAGRKTVDLVQDLAFPLPMLV

ICELLGFPAEERNAYRSWSTAILTHDDDPAVFATALREMTDYIAVQLRLR

RSRPGDDLLTELLAARDAGQLTDDEIVGMVFLLLIGGHETTVNLLGTATL

ALLRNPDQHRWLLANGHALPEAIDEFLRYESPVAMATLRFTTTPVTVDDV

VIPAGELVLVSLGGANRDPDRFPDADRLILDRRDTGHLAFGHGLHRCLGA

FLGKLEGEVALGALLRRHPKLALATEVRQLQWRDTIMLRGLESLPVSLHG

>CYP245A11(2517964335)*Salinispora pacifica* CNT045

MSSTTLPRFTLTGWNREDIVNPYPVYRRYREVAAVHRGEAGGDAPETFYV

FSYDQVAQVLSSSCFGRGRSLDATAASVPVPADQKALRAVVENWLVFMDP

PRHTELRSLLNRSFSPRIVTGLRPRIARIAQELLSRLGGQVETDLVEGFA

APLPILVIAELLGIPAGRHGWLRTNALALQEASSSRARRDTAGYARAEAA

AQEFTEYFREQVRLRRGSTGDDLLTILANAKLRGVPVSLDAVVGTAVHLL

TAGHETTTNSLAKAVLALQAHPAVLEELRGADGLTADSIEEFLRYDPPVQ

AVTRWTHQDTTLGGWEVPRGSRVVALLGSANRDPARFPLPDALDVHRPAD

RHLGFGLGIHYCLGATLARAELEIGLQTLLNGLPTLGYPAQYVDYADDMV

FHGPSRLILVNPGERFCQ

>CYP244A5(2517964339)*Salinispora pacifica* CNT045

MSATTNAELGEAPETSMPIDPGLFDCMPDLIAAARIAPVVRIPYLGRHAW

VVCDRELVKQALTHPKMGKDIALVPEWMRQPGLMVTAQPPPEYARAMIMS

DGENHARIRRIHAPVLSPRNTERWGEQVADKVEGFLDELSKAAAGSNAEV

DVVTNYTHKIPLAFISEMLGLPPAAEHRLRSITDIMLYSSDYAARREAIG

GLFGAVEEWVRNPDGLRDGVITGLLAGSDGPGAAVTEGEVIVWTLGMIIT

GYETTGSLISTSLYEALRRPPHERPRTDEDITAWIEETLRVHPPFPHPTW

RFPLEDIELGGYLIPKGAPVQVSIAAANRQPGEGADSFDAERRGHGHLSF

GLGMHYCIGAPLVRLEAKIAVRGFLRRFPQARLSADTAVQWESEWMIRRM

SSLPAVLS

>CYP211C6(2517964690)*Salinispora pacifica* CNT045

VPDIEGLLTRLYSAQGRQDPYPVYADLHAQAAIAALEPRPERQRVAAVAV

GYDLVAAVLRDPDWFKQPPPDWRDQEILRILQSSMMFINPPDHGRMRHVF

AGTFTPRRLGALEPVINRVTDELLDRMADAGPGEVDFVAEFAYPLPARVM

AEFVGIPATELAWYRERVDRVDAFLDVAGKTPERLAAANAAGAELRSFYR

ELLAHRRRTPGEDLISGLVEAVDAGGVELTEDELISNLIVLFNASFVTTV

YLLSNGLPVLLAHPEVAAALASSPELAAGAVDEILRLQTPVHLLARAAPR

DMVLGGVSIPQGQSVLLLIAAANRDPAHFPDPDRFDPRRSSPPSLAFGLG

LHYCLGAAVSRLEGRLALPRLLSRFPRLRILEQPVYSGSLFLRGIDKLSV

SPGGREHP

>CYP125A66(2517964820)*Salinispora pacifica* CNT045

MTEPRIPVGFDFTDPAVLERRVPREEFAELRRTAPVWWNAQPKGSAGFDD

DGYWVVTRYADVMAVSRDSETYSTRENTAIARFQPGTTQADREMQRVIMI

NVDPPEHTKLRAIVSRGFTPRAINALRGSLGERAERIVRDAAGRGAGDFV

TDVACELPLQAIAELIGVPQHHRRKVFDWSNQLIGYDDPAYGVDPLAASA

ELLAYAMEMAHERQRNPSDDLVTQLVNAQIDGEHLTADEFGFFVMLLAVA

GNETTRNAITHGMLAFLENPEQWELFKAERPRSAVEEIIRWATPVNVFQR

TALVDTALAGQAISAGQRVALFYGSANFDESVFEEPERFDITRSPNPHLG

FGGSGVHFCLGANLARLEIELIFNSIADHMPDIRKVADPQRLRSGWINGI

RELPVRYH

>CYP162A8(2517965916)*Salinispora pacifica* CNT045

MISPDLSDPTFYQNGDPLPVWAELRREHPVYDNRRADGTRIWAVMTHKLC

TDMLTNPKVFSSANGMRLDSNPRVLARAAGKMLNVTDPPQHDKIRKLVSS

AFTPRTIRRLEANMRATAGRAIDTAIADGGCELTRLAHKLPVSVICDLLG

VPEQDWDFMAERTRFAWGSTAADDREEVEKVAAHTEIMAYFLDLAADRRK

RPMEDLVSALVHGEVDGRSLTDEDVLYNCDALLSGGNETTRHATVGGFLA

FVMNPDEWSAIRQNRDLLPSAIQEIVRYTSPVMHSLRTATRDVEFGGINI

RAGEYVVAWLPSANRDEQLFDDPDRFNIRRSPNRHLGFIQGNHYCIGAGL

ATMELRVMFDELLNRVSEVRLAGPARRLRSNLLWGFDSLPVTFHVGAGR

>CYP211B19(2517966055)*Salinispora pacifica* CNT045

MDASEAVALLTSPPGRRDPYPTYERLRAHGPVVATAAGFVVTGYTEADTV

LRSPRFGVMDDEERDGVFPHWQDSPAMMSISQSMIRANPPDHSRMRRLAA

GVFTPRRVAALREVVAAQADELIDELIRAGRGGAPVDFLGSFAYPLPVTV

ICALLGVPAADWAQFRRWASELTAVLEPEITPQELAVADAGATELRDYFT

ELIAQRRRDPADDLTTALVQAHDAAGDRLSGEELLANLVLLLVAGFETTT

NLLGNGLFVLLTHPDSATALRGQPELAPSYVEELLRYDSPVQLTSRTVRE

SVTLGGVELPAGSWLLVLLGAANRDPARFTDPARFDPGRAQSPPLSFGAG

AHYCLGAGLARLEAQVAFPLLLRRLPELSLAGEPVRRSRLTLRGYETLPV

TVGAVAADHGTPAGAALGTP

>CYP107CL2(2517966583)*Salinispora pacifica* CNT045

LQITPEFMHDPYPVYVGMRDSAPIHLSDANAGRTWFVPRYHDVIDVLRDD

RFSAALKAPAFINQFPPEQRAQFQPFNQSIAGWVVLQDPPAHRQLRQLMN

KGFTRHLVAALRPKVTEIATGLVDTMATRRSGDFMTDFAQPFPAAVIATM

FGVPAADLSAFISWSDDIVLFAGSLRPTVEVARAAQHGLLSMTEFFRQLL

PQRRADPGDDVISLLVSVRDNGEQLTDEQVLANCAQLIVAGHETTRNLVA

NGLWTLLSHPDQLAKLRADPSLMTSAIREMMRFESPLQFVRRVARDDFDY

LGVRIQAQDGVVTMLGSANRDPAFFTEPDRFDITRNPTGHVALGYGPHIC

LGAALAEMETEVALGLLLERFPDLRAVDEQPERVMNPMLRGFAELRLSW

>CYP1056B2(2517966592)*Salinispora pacifica* CNT045

LRHVDFLSPELGDELWRVFARYRREQPVVWVSSVRMFCVFRHADIRTALT

SPDFTVDYPFRVSRQVFGPTLLDFDGTRHTILRRRLGGLLVGRDDNVAFT

GPIERSVADVLDGLDGVAGFDFVPTVARRLPEAVTAAFLGIPQADREWVY

AHLRYLLDHLDGSSRDFAVATELRREVSALVGRLLADPGQAEHTVLGRLG

NARRAGELDPEDAVGMALLVLAAGVETSTGMLANTMATLARFPRWATAGR

DDDAVLGRVVREVLRWQPPQMDTVRFARRDTTLAGVPIPAGRPLKLLLAS

GNRDEDVFTEAEEFRPDREERASLSFGHGAHSCLGTHLAVAVATRFFAAF

LRRHPGATVPGPVPPIGGWTFRQPVTLPVRLGGPDPVGATATEGGQR

>CYP105BL1(2517967083)*Salinispora pacifica* CNT045

MSAHPAATPDSGPAPSLNELTPELTYPQFDRPNPFDPPETYTELSSRCPV

APVRMPSGQSSWLITSFEGARKVLSDPRFSADMSRPGFPNLNDKPVDVVL

KDTLRHVDGEEHMHYRRTLTGEFSVKRMEALRPVITQFTDDALDQLAAAG

SGADLIKHVARVVPSRVVCHLIGVPFSDFEFFNEMVSTLMDTSSSADQIR

SAKQGLDSYFDQLITDREHHDQDDLLGRMVRRYLATGELTREEVAELAWI

TLAGGQETTPHMIGLGVATLLRHPDQLKLLQQEPALLPGAMDELLRYLTV

VHLGMPRVAVADVEIDGQTVAAGEGVVALLPLANRDPSVFERPNELDIRR

NARQHLTFSYGPHQCPGHTLARLELEVVFGRLMERFPNLRLADENTELKT

RDKAIVYGLTELAVAW

>CYP163B20(2517967117)*Salinispora pacifica* CNT045

VTATEAKYVGQVGNRNVASTIDLADPATFAGHDLTNFWQRLRDEEPIYWN

PPTGGRRGFWVLSRHADILEVYRDDMTFTSERGNVLVTLLAGGDAGAGRM

LAVTDGPRHAELRKLLLRALGPRVLGPVCRAVRANTRQMIGEAAANGECD

FATDIASRIPMITISNLLGVPEADRASLLKMTKTALSADDESISDTDSEM

ARNEILLYFQDFVEFRRKNPGEDVVSMLVNSSIDGVPLSDDDIVLNCYSL

IIGGDETSRLTMIDSINTLAANPGQWRRLKEGRCDIDKAVDEVLRWASPS

MHFGRTAVRETVIHGERIQVDDIVTLWGASGNRDERAFKRPEVFDLDRAP

NRHLSFGHGPHYCIGSYLAKVEISELLIALRDLILGFEVIGVPQRIRSNL

LSGFSTMPVRFDADRTGLASEAREG

>CYP113R3(2517967173)*Salinispora pacifica* CNT045

MTSSAGQKDPSEELFSWLRTMLETRPVYRDEENGWQVFGYADISRTLADT

TTFSSDTARAFNPPQPDLDFFDMGNLVTTDPPRHRKLRSVISSMFTPRAV

AGLSARIERITHSLLDSVDGEERFELVETLTYALPITVICELIGLPMQDE

PLFRVWGDALGTIDAATVPPEQLENEVAPAIREMNEYLLAHARKRRRQPA

DDVISRLANAKIDDRPLEDGEIVGVIGLTMFAGHATTMALTGNAVLLFDS

HPEVDAAVRADRDLLPGAIEEILRLKPPFPRLARITTTDTEIGSHAVPAG

ELVTPWIAAANRDATRFPDPETFDIHRNTGGHLVFGQGSHFCLGAPLARL

EGRIALNALLDRYAKISIDHDCGLEFENPWQLISPRRLPVAVKPL

>CYP1005A8(2517967384)*Salinispora pacifica* CNT045

VSAVLFRSWTKTVGTHWPAVTRVADQQGTEHLVVTEHELVRQVLTDQVTY

RPDNALDAVTPIPVPALRVLAGHRFRLPPTLANNGGVSHPAIRALVADAL

HPAKVAAQRPWLTKLVAERVAAIGATLDSGGAADLHAELSADLPLLVLAR

LVELPDAPVSAVKQFARAALALFWAPLDADRQLALADEVGRFHQVLREFA

DTGGGLAAALRATGHPPDVLVGALFFLLVAGQETTSQFLTLLLHRLAGEP

AVRAALRDGDVSVANVVEEGLRLEPPIVTWRRVAAVDSTLGGTAVPAGTS

VLLWLARAGRDPAIVPAPDEFRPGQRGSRRHLAFGAGAHRCLGDQLARME

AAVVVEQAAPLLDGISVVRAPWYPDNLTFRMPDAFVIRR

>CYP107AY7(2517967745)*Salinispora pacifica* CNT045

MRAEPAPIPRSGARLGQEYDQLRKTGDVHQVLLPDTSLAWLVTSPELVSR

ALADPRLALNRKHSRGGWSGFALPPALDANLLNLDAPDHTRLRRLVGPAF

SPQRVAALRPRIERTAAELVDTVVATGSPVDLVTGYCTPLSVQVIADLLG

VPEAGRTDLRAWTDTMLTSYPPDRDAIRQAVVELHDYVVNLIEAKQQRPG

DDLLSALVATEQEGDRLTRDELTSLAFLILFAGYENTANLIASTVLRLLD

HGSLRGVRASEAIEETLRLEPPAPAAIRRFPIEETTIGGATIPAGDTVLL

SIAAATRGTDGNSARLAFGNGPHFCLGAALARVEAEEAITVLARRLPSLA

LAVPGAPVRWRPTFRTHGPAELLVTW

>CYP107CT3(2517967958)*Salinispora pacifica* CNT045

MIGASGDRTGQCPIALDHDFYADPHPAYRVIKENGNKPTPIVLKTGMAYL

PPGLEAWLVTAYEDVEFVLRDPRFRKSIDEAMPLFAAQSGGTVAARGSLL

YNNMANNDPPVHTRLRKPLNAMFTARSVAGRRDVVRSAALETLEKVAGSQ

TFDLVQDFAFPFSISVISRTLGVPDADRGTFHGWVQTITGDAPPEILRRD

AGLMVEYLRGLIRGRRESAGNGTDDVLTLLATSLAEDEAVAQAYALLAAG

YETTANLIVTGFLTLAAHPEQLRRLWSDPGLVPDAVEEMLRHQSPFNLSL

YRYVTEPVELNGVRIPRGAIVFLAFAAANRDECRFTEPDSFDITQPHRDH

LAFGGGIHNCIGKHLARMEAQVAFETLIERCPGLAVHTPDEEFDWKASPT

FRGLRTLRVGPGPAPGGWEREQ

>CYP105AB29(2517968776)*Salinispora pacifica* CNT045

MTETASITTPGTSSTTTSGPASGQVTDAEFPVERGCPFSTPVEYEQIRDH

SPLAKVRLTTGREAWWIAGHELGRAVLADRRFSSDRRRDNFPFVSTDPET

RKQLQDQPTSMIGMDGAEHAQARRALMGEFTVRRMAGLRPRIQQIVDQHI

DEMLSSEQRSADLVEALSLPVPSLVICELLGVPYADHDFFQAHSGPLIRH

TTPSEVRLRIQEELNTYLGALIDRKVTDPTDDLLSRQIAKQHAAGTFNRT

SLVSMAFLLLIAGHETTANMISLGVVGLLQHPDQLAMIKEDPEKTPPAVE

ELLRYFTIADTVTARVATEDVQLGDTTINAGDGVVISGLAADHDPTVFTD

PDRIDLERGARHHVAFGFGPHQCIGQTLARLELQIVFDTLFRRIPTLRLA

APLDDIPFKSDAFVYGAEKLPVAW

>CYP107FH3(2519092413)*Salinispora pacifica* CNY330

VPIELDEAFVQDPYAVYEKLRAEGPAHRVRMPPGVPLIGGLPVWLITGYD

AVRAALADSRLSTDLHRIDGLFAQKDPDRSHRGGFSSALASHMMHTDPPD

HTRLRKLVSKAFTRRAIEALRPRIQQTTDELTAELAGHDTVDLLDAFAFP

LPIRVICLLLGVPVAEQENFRSWSRALVSGHSPEAAATAATEVAAYLGDL

VERKRHATTDDVLTALVAAHDVDDRLTHTELVSTAYLLFIAGFETTLNAL

GNGTLHLMLHRDQWTALRADRALLDNAVEEFLRLESPLKHATFRCATESL

RIGDAEIAAGDFVLLAIASANRDPRRFPDPHTLDVRRPAAGHLAFGHGIH

HCLGAPLARVEVRMAFDALLDAFPDMRLATDPAGLRWRNSTIIRGLDSLP

VHLNN

>CYP2054A3(2519092418)*Salinispora pacifica* CNY330

MSISADSEQVQGRPFDPYGAHRDDPYTFLAGLGVFYAPLLDAWCVTRRED

MVAVLRDDRSFSARDHNPRPAVALPDDVNQMFRTWRGAGAVAVGSLDPPA

HAKIRDVLNIGFTPARVRAFEPTMRAVAADLADRIGDEPEFDFIADFAVP

FALEVIGRRLGVPDDYLDRCRTWSEQRIELMMAQGDADHDRLREFARGLM

EFGEFARSLVRDRVADPRDDLISELLHDGKAGRTLTADEVAVQIPTLIFA

GHMTCAEALGTIFYQQLRSPGGWARVVDRTIPVGDLVEEGLRFDSPLAGM

YRTATRDVTVGGIRLTAGSRLLLLYGAAGRDSRAHACPAAFRPGDGSSGH

LAFGHGIHFCLGAGFARAELRVAVEVLAARMPDLGLAPGRPPRFRPVFPL

RALTELRVTRSGGGSGP

>CYP161N4(2519092423)*Salinispora pacifica* CNY330

VNTVAQLPFTQTHVLDVAPALRLLQSRGKVHRVRTPEGVPAWLVTGHAEV

QQLLDDDRLSRSDPGGRDGGTALLNKLLGPLADDHPRLRSLLEPQFTPER

LEPLRAVVEKLTEQHLDELAARTPPVDLRPTLAMSLPILVLCEWLGVPAE

DKGRFSVWTQDAAGVQDPERSQRGLAELFGYCRQLVAAKRQDPGDDVISR

LIATAGIGDTEVVALTALLLFGGYETTVARIGTGVLLLLTNPDQWAAVRA

DPALVPATVDELLRRSMPNPHNGGMPRFAVTGFEIDGAAIRAGDLVLLNI

IAANHDETAFPDPDRLDITRPTAGSLAFGYGRHSCVGAPLARMVLRVALS

RLITRFPDLRLAVGVDELKLRHETLVGGLVELPVTWGPR

>CYP211B11(2519092665)*Salinispora pacifica* CNY330

MDASEAVALLMSPPGRLDPYPTYERLRAHGPVVSTAAGFFVVTGYTEADT

VLRSPRFGVMDDEERDGAFPHWQDSPAMTSISRSMIRANPPDHSRMRRLA

AGVFTPRRVAALREVVAAQADGLVDEMIRAGRGGAAVDFMGSFAYPLPVT

VICALLGVPTADWARFRHWASDLTGVLEPELTPQELAIADAGASELRDYF

TELIAQRRRAPADDLTTALVQAHDADGDRLSGEELLANLVLLLVAGFETT

TNLLGNGLVVLLTHPDSATALRGQPELAPDYVEEFLRYDSPVQLTSRTVR

ESVSLAGVELPAGSWLLVLLGAANRDPARFTDPARFDARRAQSPPLSFGA

GAHYCLGAGLARLEAQVAFPLLLRRLPELVLAGEPTRRHRLTLRGYETLP

VTVGAVPADPGTPAEVALGTP

>CYP125A65(2519093469)*Salinispora pacifica* CNY330

MTEPRIPVGFDFTDPAVLERRVPREEFAELRRTAPVWWNVQPRGSAGFDD

DGYWVVTRYADVMAVSRDSETYSTRENTAIARFQPGTTQADREMQRVIMI

NVDPPEHTKLRAIVSRGFTPRAINALRGSLAERAERIVRDAAVRGTGDFV

TDVACELPLQAIAELIGVPQHHRRKVFDWSNQLIGYDDPAYGVDPMAAAA

ELLAYAMEMANERQRNPSDDLVTKLVNAQIDGEHLTTDEFGFFVMLLAVA

GNETTRNAITHGMLAFLEHPDQWELFKAERPRSAVEEIIRWATPVNVFQR

TALVDTTLGGQAITAGQRVALFYGSANFDESVFEEPERFDITRSPNPHLG

FGGSGVHFCLGANLARLEIELIFNSIADHMPDIRKVADPQRLRSGWINGI

REMPVRYR

>CYP107AW7(2519093796)*Salinispora pacifica* CNY330

VETVTGTSTPPPVPYIADPYPTLARIRANGPVSILHSDEGIPMWVIARYR

DVRAALADPRFGQDARRAQALADNRVAGVTLGGDIVHMLNSDPPDHTRLR

RHVQGAFTARRVAAMRPLVERITTSLLDGLTGRTTVDLVQDFAFPLPMLV

ICELLGFPAEERNAYRSWSTAILTHDDDPAAFATALREMTDYIAVQLRIR

QTRPGDDILTELLAARDAGQLTDDEIIGMVFLLLIGGHETTVNLLGTATL

ALMRNPDQHRWLLANQHALPEAIDEFLRYESPVAMATLRFTTTPVAVDDV

VIPAGELVLVSLGGANRDHDRFPDADRLILDRRDTGHLAFGHGLHRCLGA

FLGKLEGEVALGALLRQHPKLALAAEVRQLRWRDTIMLRGLDSLPVSLHG

>CYP208A22(2519094762)*Salinispora pacifica* CNY330

MTVIADRGGRIPPGPPVTAGLRLLLALGRDRLGMMTSAAAEYGDVARLPV

GPKKLYFFNHPDHAKHVLADNHANYQKGIGLVHARRALGDGLLTSEGELW

RKQRRVIQPAFQNRRLAQYAGAVGQEATRLVARLATRVDGPPVDVLDEMT

RLTLGVLGRTLLDAELTGFHGVGESFAAVQDQAMFELETLNTVPTWIPLR

RQRRFRRARQHLQEVVDVLAAERGQAVEGRDDVLSRLILSTRAEADPQLG

RERLRDELVTLLLAGHETTASTLGWSLHLLDQHPELRERVRHEARTVLGD

RVPAYEDLHQLRYTAMVVEEAIRLYPPVWILTRKARAEDEIGGYRVPAGA

DILICPYTLHRHPRFWAEPERFDPERFDPSRTTDRPRYAYIPFGAGPRFC

VGNNLGMLEATLVLAVLLRDLRLEGLPGRAVVPEPMLSLRVRGGLPMTVR

RVD

>CYP105H11(2519095246)*Salinispora pacifica* CNY330

MIDTAGQLVVDFPLRKEGESFPPPRYAEYRQRDGLVLSYLPDGKPVWLVT

RHAAVREILTSRKVSSNPDHKGFPNIGETMGVPRQDQIPGWFVGMDSPDH

DRFRRALIPEFTVRRVREMRPAIQGVIDTVIDDLLANGPQADLINDFALP

IPSLVISALLGVPTVDREFFESRTRTLVAVRASTDQERDTASRELLRYIN

RLIEIKSKWPADDLPSRLLANGVLHPQELSGVLLLLLIAGHETTANNIGL

GIVSLLENPQWIGDERVVEELLRLHSVADLVSLRVAVEDVEICGQVIRAG

EGLVPLVAAANHDESVFACPHMFNPERSARQHVAFGYGVHQCLGQNLVRV

EMELAYRTLFERIPGLRLTVDDEGLPFKYDGVLHGLHALPVRW

>CYP1005A6(2519095318)*Salinispora pacifica* CNY330

VSAVLFRSWTKTAGTHWPAVTRVADQQGTEHLVVTEHALVRQVLTDQMTY

RPDNALDAVTPIPVAALRVLAGHRFRLPPTLANNGGVSHPAIRARVADAL

HPAKVAAQRPWLTELVAERVAAIRATLDSGGSADLHAELSADLPLLVLAR

LVELPDAPVSAVKQFARAALELFWAPLDADRQLALADEVGQFHQVLREFA

DTGGGLAAALRATGHPPDVLVGALFFLLVAGQETTSQFLTLLLHRLAGEP

TVRAALRDGSVSVPNVVEEGLRLEPPIVTWRRVAAVDSTLGGTAVPAGTS

VLLWLARAGRDPAIVSAPDEFRPGQQGSRRHLAFGAGAHRCLGDQLARME

AAVVVEQVSPLLDGVTVVRAPWYPDNLTFRMPDAFVIRRGPAGAAER

>CYP244A10(2519095571)*Salinispora pacifica* CNY330

MQDTAQANLAEAPEVRMPIELRPTDCLPELLAAARVAPVVRTPYLDQHAW

VVCDRELVKQALTHPKLGKDVALAPDWMRQPGQMVTAMPPPEYARMMVMS

DGEHHARIRRIHAPVLSPQNTERWSERVAALVDGFLDNLDSADGTEVNLI

TDYTHKIPLAFTAEMLGLPPGAQRRLHDITEVMLYSADYALRQQAVGELF

EAVQEWVRDPAGLRDGVITGLLASTDGPDATVTKGEAIVWTLSLIINGYE

TTGSLISAALYEALRRPARERPHTDEAVAAWIEETLRVQPPVPHTTWRFA

LADLDLGGYLIPRGAPVQISLAAANLDPDEDADSFDAQRRGRGHLSFGLG

AHYCIGAPLARVQTKIALRGFLRRFPQARLSPDTAPRWESEWMIRRMSVL

PALLA

>CYP107AY9(2519095591)*Salinispora pacifica* CNY330

MSSQDQPTRAELAPIPRSGARVGPEYDQLRKTGDVHQVLLPDTSMAWLVT

SPDLVSRALADPRLALNRKHSRGGWSGFALPPALDANLLNLDAPDHTRLR

RLVGPAFSPQRVAALRPRIQRTAEELAETMVATGSPVDLVTGYCTPLSVQ

VIADLLGVPEAGRTDLRAWTDTMLTSYPPDRDAVRQAVVELHGYVVNLIE

AKQQRPGDDLLSALVATEQEGDRLTRDELTSLAFLILFAGYENTANLIAS

TVLRLLDHGSLRGVRVSEAIEETLRLEPPAPAAIRRFPTEEMTIGGATIP

AGDTVLLSIAAATRGADGNPARLAFGNGPHYCLGAALARVEAEEAITVLA

RRLPGLALAVPGAPVRWRPTFRTHGPAELLVAW

>CYP105AB28(2519096309)*Salinispora pacifica* CNY330

MTETASTTTPGISSTTTSGPASGEVTDAEFPVERGCPFSTPSEYEQIREH

SPLAKVRLTTGREAWWIAGHELGRAVLADRRFSSDRRRDNFPFVSTDPET

RKQLQDQPISMIGMDGAEHAQERRALMGEFTVRRMAGLRPRIQQIVDQHI

DEMLSSDQRTADLVEALSLPVPSLVICELLGVPYADHDFFQARSGPLIRH

TTPTEVRLRIQKELNTYLGALIDRKVADPTDDLLSRQIAKHHAAGTFDRT

SLVSMAFLLLIAGHETTANMISLGVVGLLQHPDQLAMIKEDPEKTAPAVE

ELLRYFTIADTVTARVATEDVQLGGTTINAGDGVVISGLAADHDPKVFTD

PDRLDLERGARHHVAFGFGPHQCIGQTLARMELQIVFDTLFRRIPTLRLA

APLDDIPFKSDAFVYGAERLPVAW

>CYP107E37(2519096342)*Salinispora pacifica* CNY330

VTIDQEIREYPFRESRGIGIDPTYELLRRTEPLARVQLPYGEVSWLVTRY

EDVKTVLTDPRFSRAAAQGKDQPRTRAEMTYEGIIGLDPPDHTRLRRLAG

KALTARRVNAIRADAQRIANEYVDEMIAKGSPGDLVELFALPYPVTVICE

LLGVPFEDRAQFRIWTEGLTSTSEQLMVYAEQLFDYMGKLVAQRRAEPTD

DLLGALVKARDEGDRLTEQELLSIAGVGLLLTGVETVSTHIPNFVYALLT

HPELMAQLRADRSLVPAAVEELLRMIPLNPAAMFPRYAVEDVTLSGITVR

AGQPVLVSLPGANRDPEVFENPETFDFTRDQNPHVAFGHGPHHCLGAQLA

RMELQVALHTVLDRFPDLRLADGDEGVSWKSGLLVRGPSRLLVGW

>CYP245A7(2516088114)*Salinispora arenicola* CNR921

MPSATLPRFALTGWSRENIVNPYPVYQRYREVASVHRGEPGGDAPDTFYV

FSYDEVVQVLSSNCFGRGRSLDAAKASVPVPAEQKALRAIVENWLVFMDP

PRHTELRSLLNRSFSPRIVTELRPRIARIAQELLSRLGQQVDVDLVESFA

APLPILVISELLGIPEERRAWLRANALALQEASSSRAGRDVDGYARAEVA

AQEFTEYFREQVRLRRGRAGGDLITILANAQQRGAPVSLDAIVGTCVHLL

TAGHETTTNSLAKAVLALREHPTVLDELRGAEGLTTDAVEEFLRYDPPVQ

AVTRWAHQDTTLGGCDIPRGSRVVALLGSANRDPARFPSPDVLDVRRPAD

RHLSFGLGIHYCLGATLARAELEIGLQALLDGVPTLGYGTQHVDYADDLV

FHGPSRLVLVNLGERCK

>CYP244A4(2516088118)*Salinispora arenicola* CNR921

MSTTTNTELTEAPETNMPVDPGLFDCMPDLIAAARVAPVVRIPYLGRHAW

VVCDRELVKQALTHPKMGKDIALVPEWMRQPGLMVTAQPDPEYARAMIMS

DGENHARIRRIHAPVLSPRNTERWGERVADKVEGFLDELSQAGSGGSTEV

NVVTNYTHKIPLAFISEMLGLPPEAEHRLRGITDIMLYSSDYAARREAIG

GLFGAVEDWVRNPADLRDGVITGLLAASDGPDAAVTEGEVIVWTLGMIIT

GYETTGSLISTSLYEAIRRPPHERPKTDEDITAWIEETLRVHPPFPHPTW

RFPLEDIELGGYLIPKGAPVQVSIAAANRKPGEGADSFDAERRGHGHLSF

GLGMHYCIGAPLVRLEAQIAVRGFLRRFPQARLSAETAVQWESEWMIRRM

SVLPAVLS

>CYP105BL2(2516088347)*Salinispora arenicola* CNR921

MSSHSAAAPDPETATPLHTLAPELTFPQFERSTPFDPPQAYTELSGRCPV

APVSMADGKPSWLITSFEGVRTTLSDPRFSSDMSHPGFPNRTGKPVDDLL

KDTLGAMDGERHRYYRRMLTGELTVRRAKAMRPVITQITDEALDQLAAAG

PGADLVKHVAFVVPSRVACHLVGIPLSDYELFTGMAATLMDSTSSDDQFA

ALQNMVSYFDTLVTDREHHDRDDLLGHMVRRYLATGELTRDMLIRLAWTT

MAAGQETTAHMIGLGVAALLRHPDQLELLRREPHLLPGAVDELMRYLPLI

QFGIPRVAMDDVEVDGQTVTAGEGVVALPPLANRDPAVFERPDELDVRRN

ARQHLTFGYGPHQCPAHALARLELEVVYGRLLERFPTLRLADSDADLKVQ

DKDIMYRVSELAVTW

>CYP211C1(2516088779)*Salinispora arenicola* CNR921

VVDVEELLTRLYSAQGRQDPFPVYADLHAQGPIAALPPEPERRRVAAVAV

GYDLVGAVLRDPEWSKAPPPGWTEQEILRTLQTSMMFINPPDHGRMRHVF

AGTFTPRRLGALEPVVNRVADELLDRMADAGAGGLDFVAEFAYPLPARVM

AEFIGIPETELDWYRERVDVIDAFLDVAGKTPQRLAAANAAGAELRAFYG

ELLARRRRTPGEDLISGLVEAVDAGGVELTEDELVSNLIVLFNASFVTTV

YMLSNGLPVLLAHPEVAAALATDPVLTAGAVDEILRLQAPVHLLARAAPR

DTVLGGVPIPQGQNVLLLIAAANRDPAHFPDPDRFDPWRSGPPSLAFGLG

LHYCLGAAVSRLEGRLALPRLLSRFPRLRIMEQPVYSGSLFLRGIDKLSV

SPGEGSTRE

>CYP125A41(2516088928)*Salinispora arenicola* CNR921

MTEPRIPAGFDFTDPEVLAHRVPREEFAELRRTAPVWWNAQPRGSAGFDD

DGYWVVTRYADVMTVSRDSDTYSTRENTAIARLRPDTTREDIEMQRVIML

NVDPPEHTKLRAIVSRGFTPRAINALRGSLAERAEHIVRDAAVRGVGDFV

SDVACELPLQAIAELIGVPQHHRRKVFDWSNQLIGYDDPAYGTDPLTASA

ELLAYAMEMAEERQRSPSDDLVTKLVNAQIDGEHLTTDEFGFFVMLLAVA

GNETTRNAITHGMVAFLDNPEQWELFKAERPKSAVEEIIRWATPVNVFQR

TALVDTVLGGQAISAGQRVALFYGSANFDEAVFEDPERFDITRSPNPHLG

FGGSGAHFCLGANLARLEIELIFNSIADHLPDIRKVAAPQRLRSGWINGI

RQMPVRYR

>CYP105J3(2516089101)*Salinispora arenicola* CNR921

MTDSVAFPQGRVCPHQPAPGYRPLAVQRPLAQVTLYDGRRVWAVTTRDLA

RRLLVDPRISSDRTNPAWPAIVPIVAAAVNDAQQKVLKIATALVGTDGPE

HKAQRKMLIPSFTFRRMNALRPMIQEIVDQQLDEMIKSGAPTDLIPAFAS

AVPVTVLYRLMGIPDDDHGIFEKLSHQLLAGPNANEAYDQLMGYMSRLIA

ERRRNPGEGVLDDLLAQHGANDDADHDELVSTLVVQVAGNHGTTGSMIAL

GLFALLQHPEQLAELRADPSLMPTAVDELLRFLSVPDAVTRLAADDIEVE

GTIIRKGDGVFFITSLINRDTDVHDAPNSLGWHHASAADHLTFGFGAHQC

LGQSLARITMEIALGALIDRLPSLRLAVPAEEVPFLPAASLQVIAELPIT

W

>CYP208A12(2516089830)*Salinispora arenicola* CNR921

MTLDTITPRVPLGPPRTAALRMLLVMKRDRLGMLSSAAARYGDASRLPVG

HKALWFFNHPRYAKHVLADNSANYHKGIGLVHARRALGDGLLTSEGDLWR

KQRKVIQPAFQSRRIAQQAGMIAEEAFALVERLRARAGAGPVELTAELTG

LTLGVLGRSLLDADLAGFDSIGDSFATVQDQAMFELETLNAVPMWIPLPR

QIRFRRARRKLQAVVDTLVDGRAGNLADRVDVLSRLILSARGEADPRVGR

ERLRDELVTLLLAGHETTASTLGWTLSLIDRHPGVWERLHAEAVEVLGDR

LPEYDDLRRLRYTVMVVEEAMRLFPPVWLLPRRALAPDTIGEYRVPANAD

VVISPYTLHRHPEFWPNPERFDPERFAPGQAADRPRYAYLPFGAGPRFCV

GNNLGMMEAVFVIALLCRHLRLTGVPGYRLVPEPMLSLRIRGGLPLVVRP

VS

>CYP211B2(2516090187)*Salinispora arenicola* CNR921

MDVSEAIAVLISPSGRLDPYPTYEQLRVHGPVSRTTAGLFVVTGYAEADM

VLRDPRFVVLDDDLRDDVFPHWQDSPAIKSIARSMLRTNPPDHSRIRRLA

AGAFTPRRVAAMREVVTAQADELVDEMIRAGRDGARVDFMDMFAYPLPVA

VICALLGVPAADRSRFRRWAGDLTGILEPEITPEELAGADAGADELRDYF

TGLIEQRRRAPADDLTTALVQAHDADGDRLSGEELLANLVVLLVAGFETT

TNLLGNGLVVLLTRPEAAAALRDEPDLAPGYVDELLRYDSPVQLTTRTVR

ESVSFAGTELPADSWLLVLLGAANRDPRRFPDPARFDPGRAQSQPLSFGA

GPHYCLGAGLARLEAQVAFPLLLRRLPELALAGRPSRRTRLTLRGYETLP

ITVGAVTADRGTPAGVAPGTP

>CYP166A4(2516090233)*Salinispora arenicola* CNR921

MTDAISFELPWARTDKFDPPAVFDALREQRPLARMRYPDGHVGWIVSSYE

LVREVLGDPRFSHSCAVGHFPVTHQGQVIPTHPQIPGMFIHMDPPEHTRY

RRLLTGEFTVRRTSRLTGHVEGVATEQIEVMREHGAPADLVATFARPLVL

RVLSGLVGLPYGERDRYLHAVTLLHDAEADPAEAAAAYEQAGAYFDEVIK

RRRRQPEDDLISTLVGDGELTGEELRNIVTLLLFAGYETTESALAVGMFA

LLHHEDQLARLRADPTKIDAAIEELLRYLTVNQYHTYRTASEDIELHGEV

INKGDSVTVSLPAANRDPARFACPAELDIDRETSGHVAFGFGIHQCLGQN

LARVELRAGLSALLRAFPNLRLAVPADEVPLRLQGSVFAVKNLPVCW

>CYP1005A1(2516090468)*Salinispora arenicola* CNR921

VSAVLFRSWTKTAGTRWPDVTRVADQSGTEHLVVTRHALVRQVLTDQATY

RPDNALEAVTPVPVAALRVLAGHRFRLPPTLANNGGVSHPAIRALVADAL

HPTKVAAQRPWLTGLVADRVATIRTTLDSGGPVDLYADLTADLPLLVLAR

LVELPDAPVNAVKQFARAALELFWAPLDADRQLALADEVGRFHQVLREFA

DTGGGLAAALRATGHSPDVLVGALFFLLVAGQETTSQFLTLLLHRLSGEP

TIRAALRAGSISAADVVEEGLRLEPPIVTWRRVAAVDSTLGGTTVAAGTS

VLLWLARAGRDPAVVAAPDEFRPGQRGSRRHLAFGAGAHRCLGDQLARME

AAVVVEQATPLLDGVTVVRPPWYPDNLTFRMPDAFVVRR

>CYP105CT1(2516090974)*Salinispora arenicola* CNR921

MNSPNHMPADRSLTAPTSGCPMALSRGRVGLDVADEISELRDGGRLGRIT

TAFGQEATLITRYDEVRAQMADSVVFNVAGVPSPPALVDGGFDTESVRRR

RTVGNLIMLDPPEHTRLRRMVAAWFTTRRVERLRPRVVEIIDAALDEMER

SGPPVDLVAMFAKTVPITVICELIGVPEELRERYRRRAERAVSASAVSTP

LDELRRLREAGWVSRELIEYHRENPSDDIIGMLLREHGTDSHDDGITDDE

LVGLANALLIAGHETTTQMLSMGTLALLRHPDQLALLRDDPSIVAGAVEE

LLRYVGVLHGGFVRVATRDTRLGGHRIHAGELVVPALTAANRDPRLLTDG

DRLDITRPPTSHVAFGHGVHFCIGAPLARMELREAFPALLRRFPGLRLAV

PDSELEFTQGTTVYSLRGLPVTW

>CYP154M5(2516090991)*Salinispora arenicola* CNR921

VEQSCPYKLDVTGRDVHAEGEAIRARGPVAQVELPGGVQGWSVTGYQAAR

QVLADPRFAKDPKKWPAYTSGAIPPNWPLIGWLLMDNMTTNDGADHQRLR

KLVSHGFTPRQVERTRPLIVKIVNDLLDGLSSAGPDEVVDLKGRFATPLP

ARVICDMFGVPEALRASVLRGAQVNVTSSISGEEAEANVEQWHRELLELV

EAKREKPDEDMASLLIAAKEEDGSTLTQEEVVGTLHLMLGAGSETLMNAL

SYAVLGMLSNPGQYEMVRNGTSSWDDVIEETLRAQAPVAQLPLRYATEDV

AVGGAVIKAGDPVLMGFTAIGRDPAVHGETAGDYDITREDKTHLSFGHGV

HFCLGAPLARLELKIALPALFERFPNMTLAVRPDQLEPQGTFIMNGHREL

PVRLGQPATVLA

>CYP107Q4(2516091132)*Salinispora arenicola* CNR921

MTTTAETSAETIDLFSPEVVADPFGWYARLREETGPTTGTLNIGTMMGGP

EMWLVTRYEDVRQVLTDPRFLTNPPADSPLEDIRAGVFKRLDFPPDLIPW

MANLLNVSDGEDHTRLRKLVSYALTAHRIGKLRPRVEKITADLLDKLAED

GKDGSPVDLVEEYCYPLPVTVICELVGIDEPDRPHWRAWGDSMATMNGER

IPTTLVKCIELARELIAKRRAEPQDDLVTALVQAQAEDQNRVSDDEIIGI

LFSLVTAGHQTTTYLIGNSVILLLENPDQLARLKENPSMWPQAVRELQRL

GPIQFAQPRFPSEDIELGGVTIPRGAPVAPLLLAANTDPRRFPDPNKLII

DRLAVGSEGHLGFGKGIHRCLGQHLAYQEAEVALQGLFTRFPDLSLAVPR

EEIPWILRPGFTRTRTLPLKLV

>CYP105G5(2516091133)*Salinispora arenicola* CNR921

LTIETTETPPADDSLRAPLPRQFMQRDDPSKLPPALAALAEQSPVGRSTL

PDGDPFWMVSGYDEARAVLSDPRFSSDRFRYHPRFKKLSGQLGERLRNDK

ARAGSFINMDPPEHTRYRKLLTGQFTVRRMRQLTVRIEQIVTEQVDVMLA

EGNSADLVSAFAVPVPSLMICELLGVRYEDRTEFQRRAAGLLQTDLPIKQ

AVENLEAQRAFMQRLVTDKRRTPADDMISGLVHHAGAEPPLTDDELVGIA

TLLLFAGLDTTASMLGLGMFMLLQRPEQMAVLRDDPSRIGDAVEELLRYL

TVVSTGLFRFAKEDVVLGDEHIPAGSTVVVSLMAANRDGRHWPEPETLDV

TRVRSSHLAFGHGVHQCLGQQLARIELTVGITELLRRLPNVRLAVPPADV

PLRNDMITYGVHRLPILWDTP

>CYP1051A1(2516091154)*Salinispora arenicola* CNR921

MATDAAITRARTVPAWKALPAAVRDTHRAFVDVGNWSDGDVVRVSLGVSR

PYLVTNPAHVQEVLHERAAIYPRGDDTALWRSVRKLVGDGILAEGDAWAA

SRRVLAPMFRPARINAMVDTMADAIAGAIDDLHGAATAGTPIDVGRELSR

IVCSAIMRVFFADRITVRDALRIMKAQETIVTAMAPRILAPLVPWWIPMP

GDRRFRAAVRSIDDILLPVLRQAQRQPDDGDDLLSRLVHARADDGRALSE

KRMRDDLVSMVAVTTETSTVVLTWLWPLLANHPDVANRLYDEIDRVVGGG

PVRGDHLAELTYTRMVLDELLRLYPAGWILPRRAATTDVLGGVRINKGAT

VILSPYVTQRMTAWWGPTAEAFDPERFAAGREAADGRHRYAYYPFGVGMH

RCLGEHLFNLEAILIVATLLSRFRFALTDTSMPGVKVAASTRPARTVEVI

LKPVAPVPAR

>CYP248A2(2516091293)*Salinispora arenicola* CNR921

VLADAVTAFDPTAVDVRRDPYPSYHWLLRHDPVHRGAHQVWYVSRFADVR

AVLGDERFARTGIRRFWTDLVGPGLLSQIVGDIILFQDEPDHGRLRGVVG

PAFSPSALRRLEPTIEATVNDLLRPARALGAMDVVADLAYPLALRAVLEL

LGLPAGDANAVGRWSRAVGRTLDRGATAEDMRRGHAAIAEFADYVERVLA

ERREDGADLLALMLAAHRSQLMSRNEIVSTVVTFIFTGHETVASQLGNGL

LSLLDHPEQMELMRRQPHLLPHAVEECLRFDPAVQSNTRQLAADVELHGR

RLRRDDVVVVLAGAANRDPGRYDRPDELDIRRDPVPSMSFGAGMRYCLGS

YLARLQLRTALGAMVALPDLRLVCSPNELAYQPRTMFRGLTRLPVAFTPA

G

>CYP105W2(2516091301)*Salinispora arenicola* CNR921

MTGYQDRPTGDQPGAPVPSGSTDPGIGAFPLPRRCPFSPPAEYARLRAEH

PVVRLPMLGGDTAWVVSRHADVRQVLSDPRMSADRRRPGFPKFAPTTEGQ

RQASFANFRPPLNWLDPPEHAICRRQIVDEFSVRRVRQSRALVERVVDTH

LDALTAAAPGADLVSTFAYPVPSQVICEVLGVPYGEHEFFERRSTLMFRR

STPADERARCAREIRDFLDMVVTDKEHRPGDDVLSRLLYRQRRAGGVDHE

AVVSMAFVLLVAGHVTTSNMLALSVLALLTHPARLARLRAEPERFPAAVE

ELLRYFTVVEAATARTATAEVTIGGVTIAAGEGVVALGQAANRDPRVFEH

PDEFDPDRDARAHLAFGYGRHICPGQHLARLEMEVALSRLFRRLPGLRLT

MEVSDLPLKEDSNIFGLYALPVAW

>CYP105CP2(2516091725)*Salinispora arenicola* CNR921

MTKSMPVQDLPAFPIPRECPYRPSAQHVSLRSGGPMAKVRLYNGRTAWLV

TDSAHARAVLSDYRRVSIKPYHGNYPLLNEEFEKVVDSGYADVLFGVDPP

EHTRQRQMIMPSFTLRRTAVLRPDIQRIVDDKLDEMMRHGAPGDLVTEFA

QPVPSMVMSFLLGVPWEDHEEFETPAHKLFVPELAEEATTELGAYLERLI

QKKEQPGGTPGGTGLLDDLIRDHLRAGALSRDELVHIAMAMLVAGTDTTT

NVISLGTLALLDNPDQWAALRDNPDELIPGAVEEILRYTSLIEAFARVAV

SDIELNGAVIKEGEGILISSAGVNFDPALAPDPGRFDIRRPPRPSFSFSH

GIHRCPGDNLARLELEIAFRSLVTRMPNLRTAKPIDQIPSNNNDGTLQRL

YELPVVW

>CYP105CN1(2516091727)*Salinispora arenicola* CNR921

MAAPAPQATQSTTPHPPSYPLPRECPYRPSAGTARLRDAGPVSTVRLYDG

RTAWLVTGAAEARALLADSRVSNRADFPNYPVMDERHLSMRATREMAREE

EGGFAAALFGMDPPEHTRQRQLLLPRFTVRQVAARRPAIQRIVDEHLDAM

EANGSPADLVSAFATPVPTMVVCTHLGVPYQDRTRFEPAVAGLFEPDRAD

AAMAELTAYLHQLIETKQSEPGDGVIDHLIANHLRPGAIDRAELVAIASA

ILVAGTVTTSSAIALGTLALLTAPGQYAALVDNPDLVPGAVNEILRYLSL

VEQLARVATEDIEIGGKLIRAGDGIIVSFAAGNLDPNVTTHPDRLDVALP

PTNHLAFSHGIHHCIGQNLALLELDIAFRALVSRFPTLRLAVPAEQLPTY

FAGDVPRLACLPVTW

>CYP107FS2(2516091728)*Salinispora arenicola* CNR921

MPVPQGEQNLTTEVFADPKALFATLGSRQPLHRISLPDGMPAVLVTGNRE

ARQALSDPRLVRSITAAAPELHKYHPLASDDYALSRHMLFADPPDHGRMR

KLVSTAFTRRRVEQMRPRIQQITDDLIDVIAAKGEADLVETLALPLPIAV

ISEMLGVPFADRSEFERHAEVLTGINASSGFDAIIAAGRWFDEYLAELVQ

QRRREPQDDLISGMLAAQDKGDRLTDVELRSNALLLLSAGFETTVNLVAN

GLLALLRHPEAMAALRSEPNLMTTAVDELLRYDSPVSCVTYHFAQEPVEI

GGFEIRSGEHVVIAAAAANHDPTVFADPSRLDLRREGSGQILSFSHGIHF

CLGAPLARLEGEIAFGTVLRRLAGLRLAVPTDSLVWKASFVLHRLERLPV

TFTPDRAPNPIDSVHTV

>CYP107AY2(2516091879)*Salinispora arenicola* CNR921

MTAEPTPIPRSGARLGQEYDQLRKTGDVHQVLLPDTSLAWLVTNPEVAAR

ALADPRLALNRRNSRGGWSGFALPPALDANLLNLDAPDHTRLRRLVGPAF

SPQRVAALRPGIRRAAEHLLDTLVATSGPTDLVTGYCNPLSVQVIADLMG

VPEAGRTNLRAWTDTMLTSYPPDRDAIRRAVTELHGYVVDLIDIKQQQPG

DDLLSALVTIEQDGDRLSRDELTSLAFLILFAGYENTANLIASAVLWLLD

HGGLNVVPSSEAIEGTLRHEPPAPVAIRRFPTEDIIIGGVTIPAGDTVLL

SVAAATRGADGNAARLAFGNGPHYCLGAALARVEAEEALTVLARRLPGLT

LAVPPSQVRWRPTFRTHGPAELLVGW

>CYP105AB8(2516092014)*Salinispora arenicola* CNR921

MTETASSRLTDTEFPVQRECPFAEPVEYEQIRERSSIAMVRLTGGGEAWW

ISGHEQGRAVLADRRFSSDRRKANFPFVSTDPAVRKRLHAQPLSLISMDG

AEHTQARRALIGEFTVRRLAALRPRIQQIVDQCIDEMLTTDQHRADLVKT

LSLPVPSLVICELLGVPYADHDFFQEHTATLVRRNTASEVRQHSIDELNA

YLGALIDRKLASPDDDLLGRQIARQHRDGTFDRSSMVSLAFLLLVAGHET

TANMISLGVVGLLQHPEQLAMIKDDPDKTPLAIEELLRFFTIVDSVTSRV

ATEDVRFGDTTINAGDGVVVSGLSADWDPTVFADPDRLDLERGARHHLAF

GFGPHQCLGQNLARLELQIVFDTLFHRIPTLRLAAPLDKIPFKTDAAIYG

ARELPVAW

>CYP208A12(2515698251)*Salinispora arenicola* CNH964

MTLDTITPRVPLGPPRTAALRMLLVMKRDRLGMLTSAAARYGDASRLPVG

HKALWFFNHPRYAKHVLADNSANYHKGIGLVHARRALGDGLLTSEGDLWR

KQRKVIQPAFQSRRIAQQAGMIAEEAFALVERLRARAGAGPVELTAELTG

LTLGVLGRSLLDADLAGFDSIGDSFATVQDQAMFELETLNAVPMWIPLPR

QIRFRRARRKLQAVVDTLVDGRAGNLADRVDVLSRLILSARGEADPRVGR

ERLRDELVTLLLAGHETTASTLGWTLSLIDRHPGVWERLHAEAVEVLGDR

LPEYDDLRRLRYTVMVVEEAMRLFPPVWLLPRRALAPDTIGEYRVPANAD

VVISPYTLHRHPEFWPNPERFDPERFAPGQAADRPRYAYLPFGAGPRFCV

GNNLGMMEAVFVIALLCRHLRLTGVPGHRLVPEPMLSLRIRGGLPLVVRP

VS

>CYP105AB8(2515698783)*Salinispora arenicola* CNH964

MTETASSRHTDTEFPVQRECPFAEPVEYEQIREQSSIAMVRLTGGGEAWW

ISGHEQGRAVLADRRFSSDRRKANFPFVSTDPAIRKRLHAQPLSLISMDG

AEHTQARRALIGEFTVRRLAALRPRIQQIVDQCIDEMLTTDQHRADLVKT

LSLPVPSLVICELLGVPYADHDFFQEHTATLVRRNTASEVRQHSIDELNA

YLGALIDRKLASPDDDLLGRQIARQHRDGTFDRSSMVSLAFLLLVAGHET

TANMISLGVVGLLQHPEQLAMIKDDPDKTPLAIEELLRFFTIVDSVTSRV

ATEDVRFGDTTINAGDGVVVSGLSADWDPTVFADPDRLDLERGARHHLAF

GFGPHQCLGQNLARLELQIVFDTLFHRIPTLRLAAPLDKIPFKTDAAIYG

ARELPVAW

>CYP105BL2(2515699196)*Salinispora arenicola* CNH964

MSSHSAAAPDPETATPLHTLAPELAFPQFERSAPFDPPQAYTELSGRCPV

APVSMADGKPSWLITSFEGVRTTLSDPHFSSDMSHPGFPNRTGKPVDDLL

KDTLGAMDGERHRYYRRMLTGELTVRRAKAMRPVITQITDEALDQLAAAG

PGADLVKHVAFVVPSRVACHLVGIPLSDYELFTGMAATLMDSTSSDDQFA

ALQNMVSYFDTLVTDREHHDRDDLLGHMVRRYLATGELTRDMLIRLAWTT

MAAGQETTAHMIGLGVAALLRHPDQLELLRREPHLLPGAVDELMRYLPLI

QFGIPRVAMDDVEVDGQTVTAGEGVVALPPLANRDPAVFERPDELDVRRN

ARQHLTFGYGPHQCPAHALARLELEVVYGRLLERFPTLRLADSDADLKVQ

DKDIMYRVSELAVTW

>CYP211B2(2515699438)*Salinispora arenicola* CNH964

MDVSEAIAVLISPSGRLDPYPTYEQLRAHGPVSQTTAGLFVVTGYAEADM

VLRDPRFVVLDDDLRDDVFPHWQDSPAIKSIARSMLRTNPPDHSRIRRLA

AGAFTPRRVAAMREVVTAQADELVDEMIQAGRDGARVDFMDMFAYPLPVA

VICALLGVPAADRSRFRRWAGDLTGILEPEITPEELAGADAGADELRDYF

TGLIEQRRRAPADDLTTALVQAHDADGDRLSGEELLANLVVLLVAGFETT

TNLLGNGLVVLLTRPEAAAALRDEPDLAPGYVDELLRYDSPVQLTTRTVR

ESVSFAGTELPAGSWLLVLLGAANRDPRRFPDPARFDPGRAQSQPLSFGA

GPHYCLGAGLARLEAQVAFPLLLRRLPELALAGRPSRRTRLTLRGYETLP

ITVGAVTADRGTPAGVAPGTP

>CYP125A41(2515699566)*Salinispora arenicola* CNH964

MTEPRIPAGFDFTDPEVLAHRVPREEFAELRRTAPVWWNAQPRGSAGFDD

DGYWVVTRYADVMTVSRDSDTYSTRENTAIARLRPDTTREDIEMQRVIML

NVDPPEHTKLRAIVSRGFTPRAINALRGSLAERAEHIVRDAAVRGVGDFV

TDVACELPLQAIAELIGVPQHHRRKVFDWSNQLIGYDDPAYGTDPLTASA

ELLAYAMEMAEERQRSPSDDLVTKLVNAQIDGEHLTTDEFGFFVMLLAVA

GNETTRNAITHGMVAFLDNPEQWELFKAERPKSAVEEIIRWATPVNVFQR

TALVDTVLGGQAISAGQRVALFYGSANFDEAVFEDPERFDITRSPNPHLG

FGGSGAHFCLGANLARLEIELIFNSIADHLPDIRKVAAPQRLRSGWINGI

RQMPVRYR

>CYP105J3(2515699716)*Salinispora arenicola* CNH964

MTDSVAFPQGRVCPHQPAPGYRPLAVQRPLAQVTLYDGRRVWAVTTRDLA

RRLLVDPRISSDRTNPAWPAIVPIVAAAVNDAQQKVLKIATALVGTDGPE

HKAQRKMLIPSFTFRRMNALRPMIQEIVDQQLDEMIKSGAPTDLIPAFAS

AVPVTVLYRLMGIPDDDHGIFEKLSHQLLAGPNANEAYDQLMGYMSRLIA

ERRRNPGEGVLDDLLAQHGANDDADHDELVSTLVVQVAGNHGTTGSMIAL

GLFALLQHPEQLAELRADPSLMPTAVDELLRFLSVPDAVTRLAADDIEVE

GTIIRKGDGVFFITSLINRDTDVHDAPNSLGWHHASAADHLTFGFGAHQC

LGQSLARITMEIALGALIERLPSLRLAVPAEEVPFLPAASLQVIAELPIT

W

>CYP244A4(2515699873)*Salinispora arenicola* CNH964

MSTTTNTELTEAPETNMPVDPGLFDCMPDLIAAARVAPVVRIPYLGRHAW

VVCDRELVKQALTHPKMGKDIALVPEWMRQPGLMVTAQPDPEYARAMIMS

DGENHARIRRIHAPVLSPRNTERWGERVADKVEGFLDELSQAGSGGSTEV

NVVTNYTHKIPLAFISEMLGLPPEAEHRLRGITDIMLYSSDYAARREAIG

GLFGAVEDWVRNPADLRDGVITGLLAASDGPDAAVTEGEVIVWTLGMIIT

GYETTGSLISTSLYEAIRRPPHERPKTDEDITAWIEETLRVHPPFPHPTW

RFPLEDIELGGYLIPKGAPVQVSIAAANRKPGEGADSFDAERRGHGHLSF

GLGMHYCIGAPLVRLEAQIAVRGFLRRFPQARLSAETAVQWESEWMIRRM

SVLPAVLS

>CYP245A7(2515699877)*Salinispora arenicola* CNH964

MPSATLPRFALTGWSRENIVNPYPVYQRYREVASVHRGEPGGDAPDTFYV

FSYDEVVQVLSSNCFGRGRSLDAAKASVPVPAEQKALRAIVENWLVFMDP

PRHTELRSLLNRSFSPRIVTELRPRIARIAQELLSRLSQQVDVDLVESFA

APLPILVISELLGIPEERRAWLRANALALQEASSSRAGRDVDGYARAEVA

AQEFTEYFREQVRLRRGRAGGDLITILANAQQRGAPVSLDAIVGTCVHLL

TAGHETTTNSLAKAVLALREHPAVLDELRGAEGLTTDAVEEFLRYDPPVQ

AVTRWAHQDATLGGCDIPRGSRVVALLGSANRDPARFPSPDVLDVRRPAD

RHLSFGLGIHYCLGATLARVELEIGLQALLDGVPTLGYGTQHVDYADDLV

FHGPSRLVLVNLGERCK

>CYP105CP2(2515700040)*Salinispora arenicola* CNH964

MTKSMPVQDLPAFPIPRECPYRPSAQHVSLRSGGPMAKVRLYNGRTAWLV

TDSAHARAVLSDYRRVSIKPYHGNYPLLNEEFEKVVDSGYADVLFGVDPP

EHTRQRQMIMPSFTLRRTAVLRPDIQRIVDDKLDEMMRHGAPGDLVTEFA

QPVPSMVISFLLGVPWEDHEEFETPAHKLFVPELAEEATTELGAYLERLI

QKKEQPGGTPGGTGLLDDLIRDHLRAGALSRDELVHIAMAMLVAGTDTTT

NVISLGTLALLDNPDQWAALRDNPDELIPGAVEEILRYTSLIEAFARVAV

SDIELNGAVIKEGEGILISSAGVNFDPALAPDPGRFDIRRPPRPSFSFSH

GIHRCPGDNLARLELEIAFRSLVTRMPNLRTAKPIDQIPSNNNDGTLQRL

YELPVVW

>CYP105CN1(2515700042)*Salinispora arenicola* CNH964

MAAPAPQATTSTTPHPPSYPLPRECPYRPSAGTAHLRDAGPVSTVRLYDG

RTAWLVTGAAEARALLADSRVSNRADFPNYPVMDERHLSMRATREMAREE

EGGFAAALFGMDPPEHTRQRQLLLPRFTVRQVAARRPAIQRMVDEHLDAM

EANGSPADLVSAFATPVPTMVVCTHLGVPYQDRTRFEPAVAGLFEPDRAD

AAMAELTAYLHQLIETKQSEPGDGVIDHLIANHLRPGAIDRAELVAIASA

ILVAGTVTTSSAIALGTLALLTAPGQYTALVDNPDLVPGAVNEILRYLSL

VEQLARVATEDIEIGGKLIRAGDGIIVSFAAGNLDPNVTTHPDRLDVALP

PTNHLAFSHGIHHCIGQNLALLELDIAFRALVSRFPTLRLAVPAEQLPTY

FAGDVPRLACLPVTW

>CYP107FS2(2515700043)*Salinispora arenicola* CNH964

MPVPQGEQNLTTEVFADPKALIATLGSRQPLHRISLPDGMPAVLVTGNRE

ARQALSDPRLVRSITAAAPELHKYHPLASDDYALSRHMLFADPPDHGRMR

KLVSTAFTRRRVEQMRPRIQQITDDLIDVIAAKGEADLVETLALPLPIAV

ISEMLGVPFADRSEFERHAEVLTGINASSGFDAIIAAGRWFDEYLAGLVQ

QRRREPQDDLISGMLAAQDKGDRLTDVELRSNALLLLSAGFETTVNLIAN

GLLALLRHPEAMAALRSEPNLMTTAVDELLRYDSPVSCVTYHFAQEPVEI

GGFEIRSGEHVVIAAAAANHDPTVFADPSRLDLRREGSGQILSFSHGIHF

CLGAPLARLEGEIAFGTVLRRLAGLRLAVPTDSLVWKASFVLHRLERLPV

TFTPDRDPNPIDSVHTV

>CYP125G6(2515700084)*Salinispora arenicola* CNH964

VPTLLNEHVSYDGAAIAIVDSDGATSWIRLAERVNRWVHLLRAHGLDTGD

RLACVTGNRRETFEVLLAALHTGVTVVPVNWHLTVTEIGHILSDSGSRVV

ITEELHVKAVAAAADGMAGPVAGLVLGDREIEGFAAVEPLLAAASPAEPE

GQVCGATMLYTSGTTGRPKGVVNNLFVTGAPYARVGRLCDYARSVLGVPR

RERMLLDGPWYHSSQLFFALLSLLQGSRLVIRPYFDPAATLKTIDDHRIT

VTHLVPTQLVRLLRVDALTRQMFSGASLRRVWHGGGPCPPEVKRSMIDWW

GPVLVEYYGATEGGVVTLIDSAEWLARPGSVGRAVPPSEVVVVDDGGQPV

AAGQTGQVFFRRRTGNRFHYHNAPEKTQAAYLAPDTFTYGEVGHVDEDGY

LFLTGRAQDMIVSGGVNVYPAEVEAALLRHPVVRDAAVIGVADDEFGERV

VGIVVPETAVDPDDLATHLDAHCRVSLAGFKVPRTYRVVESLPRDETGKL

RKDALRSKFGWLSGAAMTVPRPATGQPTAHRPDIAHPTTYVSGVPHDEFA

RRRRDEPVGWVAEPVLTRHTAAGRTATRGSGFWAVTRYEDVVAASRRVTD

FSSAAKGAFLTDPRTPADLQQARQLLVNMDDPHHARLRKLVTSVFTPRAV

RGLLASIDAHAAALVAKVVAAGEFDVVTDLAAELPLLVLADLLGVPKQDR

ALLYGWSNHLVGFDDPDFGGGDIDAYRTAMAEAFQYALNLGVERRARPTD

DLVSLLANAEVDGTRLTDREFCNFWLLLVVAGNETTRHLVAGTMQALTEH

PGECARLVEGRVPTESAVEELLRWVTPIMQFRRTATRDTEIGGQAVTAGE

KVVLYYTSANRDATVFAEPDRLDLGRTPNRQLSFGIGPHYCLGAHLARAE

LTALLRVMSPHLGSLQLTGPVSRLASNFVNGVKAMPAVIGSR

>CYP154M15(2515700109)*Salinispora arenicola* CNH964

MNDKCPFAALDVTGQDLHGEAARLREQGPAVLVELPGGVKAWSINRYEVI

RELLMDRRVTKNARKHWPAFIKGEIPPDWEMISWVAMDNMVTAYGKDHVR

LRKLVGRAFTQRRADALRPQVVALSTKLLDDLGATPPGEVVDLRERFAYP

LPAMLVASLIGMSEAALAACSKVIDMMVHTNVSPEEAQAVLRGWRAAMAD

LIESKRRTPGEDITTDLIAAREEDGSRLSEAELADTIFAILGAGSETTIN

FFDNAITALLSRPGQLQLLRTGAVTWDDVIDEVLRVESPLAHLPLRYAVE

DIELDGVTIPQGDPILVNYAAAGRDPALHGGTADEFDLARGDKTHLSFGY

GPHYCLGAGIARMVATIGLSMLFERFPDLSLAVPATELKRLPTFIMNGHQ

TLPVRLTAHAR

>CYP107Q4(2515700322)*Salinispora arenicola* CNH964

MTTTAETSAETIDLFSPEVVADPFGWYARLREETGPTTGTLNIGTMMGGP

EMWLVTRYEDVRQVLTDPRFLTNPPADSPLEDIRAGVFKRLDFPPDLIPW

MANLLNVSDGEDHTRLRKLVSYALTAHRIGKLRPRVEKITADLLDKLAED

GKDGSPVDLVEEYCYPLPVTVICELVGIDEPDRPHWRAWGDSMATMNGER

IPTTLVKCIELARELIAKRRAEPQDDLVTALVQAQAEDQNRVSDDEIIGI

LFSLVTAGHQTTTYLIGNSVILLLENPDQLARLKENPSMWPQAVRELQRL

GPIQFAQPRFPSEDIELGGVTIPRGAPVAPLLLAANTDPRRFPDPNKLII

DRLAVGSEGHLGFGKGIHRCLGQHLAYQEAEVALQGLFTRFPDLSLAVPR

EEIPWILRPGFTRTRTLPLKLV

>CYP105G5(2515700323)*Salinispora arenicola* CNH964

LTIETTETPPADDSLRAPLPRQFMQRDDPSKLPPALAALAEQSPVGRSTL

PDGDPFWMVSGYDEARAVLSDPRFSSDRFRYHPRFKKLSGQLGERLRNDK

ARAGSFINMDPPEHTRYRKLLTGQFTVRRMRQLTVRIEQIVTEQVDVMLA

EGNSADLVSAFAVPVPSLMICELLGVRYEDRTEFQRRAAGLLQTDLPIKQ

AVENLEAQRAFMQRLVTDKRRTPADDMISGLVHHAGAEPPLTDDELVGIA

TLLLFAGLDTTASMLGLGMFMLLQRPEQMAVLRDDPSRIGDAVEELLRYL

TVVSTGLFRFAKEDVVLGDEHIPAGSTVVVSLMAANRDGRHWPEPETLDV

TRVRSSHLAFGHGVHQCLGQQLARIELTVGITELLRRLPNVRLAVPPADV

PLRNDMITYGVHRLPILWDTP

> CYP1051A1(2515700343)*Salinispora arenicola* CNH964

MATDAAITRARTVPAWKALPAAVRDTHRALVDVGNWSDGDVVRVSLGVSR

PYLVTNPAHVQEVLHERAAIYPRGDDTALWRSVRKLVGDGILAEGDAWAA

SRRVLAPMFRPARINAMVDTMADAIAGAVDDLHGAATAGTPIDVGRELSR

IVCSAIMRVFFADRITVRDALRIMKAQETIVTAMAPRILAPLVPWWIPMP

GDRRFRAAVRSIDDILLPVLRQAQRQPDDGDDLLSRLVRARADDGQALSE

KRMRDDLVSMVAVTTETSTVVLTWLWPLLANHPDVANRLYDEIDRVVGGG

PVRGDHLAELTYTRMVLDELLRLYPAGWILPRRAATTDVLGGVRINKGAT

VILSPYVTQRMTAWWGPTAEAFDPERFAAGREAADGRHRYAYYPFGVGMH

RCLGEHLFNLEAILIVATLLSRFRFALTDTSMPGVKVAASTRPARTVEMI

LKPVAPVPAR

>CYP211C1(2515701173)*Salinispora arenicola* CNH964

VVDVEELLTRLYSAQGRQDPFPVYADLHAQGPIAALPPEPERRRVAAVAV

GYDLVGAVLRDPEWSKAPPPGWTEQEILRTLQTSMMFINPPDHGRMRHVF

AGTFTPRRLGALEPVVNRVADELLDRMADAGAGGLDFVAEFAYPLPARVM

AEFIGIPETELDWYRERVDVIDAFLDVAGKTPQRLAAANAAGAELRAFYG

ELLARRRRTPGEDLISGLVEAVDAGGVELTEDELVSNLIVLFNASFVTTV

YMLSNGLPVLLAHPEVAAALATDPVLTAGAVDEILRLQAPVHLLARAAPR

DTVLGGVPIPQGQNVLLLIAAANRDPAHFPDPDRFDPWRSGPPSLAFGLG

LHYCLGAAVSRLEGRLALPRLLSRFPRLRIMEQPVYSGSLFLRGIDKLSV

SPGEGSTRE

>CYP107FJ2(2515701419)*Salinispora arenicola* CNH964

MSETLSTSVLLADAAEQRAWRAKLRGAGPVHRITTQSGETGWLIVGHEEA

RNALVDLRLQGRTATVGHGRRMPEDLERALNSHMLNVGPPDHTRLRRLVS

AAFTRRRIEQMRPRIQELTDELLDGLAGADEADLVAGLALPLPMRVLVDL

FGIPAEDCADFNVWTKVLTSAGAVDLDRLTTAAGEMVAYLRGLLDRKRQV

PESDLLSALVAVRDGADRLSDDELTSMVYLLLTAGYETTVNLIGNGLLNL

LANPEQLVAFKADPDLLPQVVEEAMRFDSPVQIAVRHSTEPVEIAGQAIP

SGALILVSLLWANRDPDRFTEPEAFRVDRQDNPQLGFGYGFHHCIGAPLA

RMEGTIAIGTVIRRFPALRLAHPAGSLTWRASMVMHGLTALPVHLR

>CYP154M20(2515701426)*Salinispora arenicola* CNH964

MERCPYVLDRAGRDLHGEAKALRARGPVTLVELHGGYTAWSVTSYEIAKQ

LLVDPRISKNTKETWPEFREGKVPQDWELYTWVAMDNMQTRDGEEHDRLR

KLVAQAFTTRQVAKVRPMIEDIVDRLLDDLEKVPAGEVVDIKGRYFYPLS

TILVCDLLGIPEADRAEALHGTVVNARTTNSAEESEANLHQWQSALSKLV

ETKRREPGNDITTLIIKAREDEQAPLTDDEVIGSLHLLIGGGTETTSNVL

CNTLIDLLTHPDQMAMIRSGAVGWEAAWEEEVRKDGAVGSMPFRCANADI

EIGGVTIAKGDLVLINYAAAGRDPERYGDTTAEFDITRADKTNLSFGYGR

HRCLGPALATMEAMISLPALFERFPDLVLAVPREELKPQGTFVFNGYAEV

PMLLRS

>CYP248A2(2515701448)*Salinispora arenicola* CNH964

VLADAVTAFDPTAVDVRRDPYPSYHWLLRHDPVHRGAHQVWYVSRFADVR

AVLGDKRFARTGIRRFWTDLVGPGLLSQIVGDIILFQDEPDHGRLRGVVG

PAFSPSALRRLEPTIEATVNDLLRPARALGAMDVVADLAYPLALRAVLEL

LGLPAGDANAIGRWSRAVGRTLDRGATAEDMRRGHAAIAEFTDYVERALA

ERREDGADLLALMLAAHRSQLMSRNEIVSTVVTFIFTGHETVASQLGNGL

LSLLDHPEQLELVRRQPHLVPQAVEECLRFDPAVQSNTRQLAADVELHGR

RLRRDDVVVVLAGAANRDPGRYDRPDELDIRRDPVPSMSFGAGMRYCLGS

YLARLQLRTALGAMVALPDLRLVCNPNELAYQPRTMFRGLTRLPVAFTPA

G

>CYP105W2(2515701455)*Salinispora arenicola* CNH964

MTGYQDRPTGDQPGAPVPSGSTDPGIGAFPLPRRCPFSPPAEYARLRAEH

PVVRLPMLGGDTAWVVSRHADVRQVLSDPRMSADRRRPGFPKFAPTTEGQ

RQASFANFRPPLNWLDPPEHAICRRQIVDEFSVRRVRQSRALVERVVDTH

LDALTAAAPGADLVSTFAYPVSSQVICEVLGVPYGEHEFFERRSTLMFRR

STPADERARCAREIRDFLDVVVTDKEHRPGDDLLSRLLYRQRRAGGVDHE

AVVSMAFVLLVAGHVTTSNMLALSVLALLTHPARLARLRAEPERFPAAVE

ELLRYFTVVEAATARTATAEVTIGGVTIAAGEGVVALGQAANRDPRVFEH

PDEFDPDRDARAHLAFGHGRHICPGQHLARLEMEVALSRLFRRLPGLRLT

MEVSDLPLKEDSNIFGLYALPVAW

>CYP154M13(2515701468)*Salinispora arenicola* CNH964

MSTGRPVVLDPTGRDIHGEADHLRALGPATLVELPGGILAWSINSYEVGK

ALLSAPNVSKSARRHWPAFYNGEIPPDWEMISWVAMDNISTTFGGDHRRL

RRLTAKAFGSRRAEQVRPMATHMVNTLLDRMADAADAGEVVDLKAAFAYP

LPGMLVAELIGMSEEARVAAAKVIDMMTATNITPEQAQGVLLGWRDAITD

LIALKRAQPGDDITSDLIAARDEDGSLLTEQELVDTIFAILGAGSETTIN

FFDNAITQLLTHPEQLELVKSGQVSWDDVIEEVLRLESPLASLPMRFAVE

DIQLDGVTIHKGDPILINYAALGRDPALHGESAGVFDVTRQHKEHLSFGH

GAHYCLGAGIARMVAKTGLSALFERFPRMTLAVSAKDLVPYPTFIMNGNR

QLPVHLSGALTWREGEQSALGRA

>CYP154M21(2515701469)*Salinispora arenicola* CNH964

MEKCPYVLDRAGSDIHKEASNLRARGPVTLVELHGGYTAWSVTSYEVAKQ

LLMDPRISKNTKAHWPEFRDGNVPQDWELYTWVAMDNMQTRDGKEHDRLR

KLVAPAFTGRQAVKSRPIIEEIVNRLLDDLETAPRGQAVDIKARYFYPLS

TILVCDLLGIAEEDRDVILHGNVVNSKTTNTAEESEANLHQWQTALGRLV

ETKRRDPGDDLTTVIIKAGEDEQTPLTDDEVIGSLHLLIGGGTETTANVL

CHTVVDMLTHPDQLAMVRSGAVSWESAWEEEVRKDGAVGSMPFRCATDDV

EIGGVTIAKGDLVLINYAAAGRDPERYGDAAAEFDITRADKANLSFGYGR

HRCLGPALATMEAMVALPALFDRFPNLALAVPPNELKPQGTFIFNGYAEV

PLLLRS

>CYP166A4(2515701500)*Salinispora arenicola* CNH964

MTDAISFELPWARTDKFDPPTVFDALREQRPLARMRYPDGHVGWIVSSYE

LVREVLGDPRFSHSCAVGHFPVTHQGQVIPTHPQIPGMFIHMDPPEHTRY

RRLLTGEFTVRRTSRLTGHVEGVATEQIEVMREHGAPADLVATFARPLVL

RVLSGLVGLPYGERDRYLHAVTLLHDAEADPAEAAAAYEQAGAYFDEVIE

RRRRQPEDDLISTLVGDGELTGEELRNIVTLLLFAGYETTESALAVGMFA

LLYHEDQLARLRADPTKIDAAIEELLRYLTVNQYHTYRTASEDIELHGEV

INKGDSVTVSLPAANRDPARFACPAELDIERETSGHVAFGFGIHQCLGQN

LARVELRAGLSALLRAFPNLRLAVPADEVPLRLQGSVFAVKNLPVCW

>CYP1005A1(2515701659)*Salinispora arenicola* CNH964

VSAVLFRSWTKTAGTRWPDVTRVADQSGTEHLVVTRHALVRQVLTDQATY

RPDNALEAVTPVPVAALRVLAGHRFRLPPTLANNGGVSHPAIRALVADAL

HPTKVAAQRPWLTGLVADRVATIRTTLDSGGPVDLYADLTADLPLLVLAR

LVELPDAPVNAVKQFARAALELFWAPLDADRQLALADEVGRFHQVLREFA

DTGGGLAAALRATGHSPDVLVGALFFLLVAGQETTSQFLTLLLHRLSGEP

TIRAALRAGSISVADVVEEGLRLEPPIVTWRRVAAVDSTLGGTTVAAGTS

VLLWLARAGRDPAVVAAPDEFRPGQRGSRRHLAFGAGAHRCLGDQLARME

AAVVVEQATPLLDGVTVVRPPWYPDNLTFRMPDAFVVRR

>CYP107AY2(2515702293)*Salinispora arenicola* CNH964

MTAEPTPIPRSGARLGQEYDQLRKTGDVHQVLLPDTSLAWLVTNPEVAAR

ALADPRLALNRRNSRGGWSGFALPPALDANLLNLDAPDHTRLRRLVGPAF

SPQRVAALRPGIRRAAEHLLDTLVATSGPTDLVTGYCNPLSVQVIADLMG

VPEAGRTNLRAWTDTMLTSYPPDRDAIRQAVTELHGYVVDLIDIKQQQPG

DDLLSALVTIEQDGDRLSRDELTSLAFLILFAGYENTANLIASAVLWLLD

HGGLNVVPSSEAIEGTLRHEPPAPVAIRRFPTEDIIIGGVTIPAGDTVLL

SIAAATRGADGNAARLAFGNGPHYCLGAALARVEAEEALTVLARRLPGLT

LAVPPSQVRWRPTFRTHGPAELLVGW

>CYP163B16(2515702571)*Salinispora arenicola* CNH964

VTAADPGRVGSGESQTPAHTVDLADPATFANRDLTGFWQQLRDEEPIHWN

PPTSGRRGFWVVSRYADILDVYRDDVTFTSERGNVLVTLLAGGDAGAGRM

LAVTDGPRHAELRKLLLRALGPRVLAPVCAAVRANTRQMVREAVTKGECD

FASDIASRIPMMTISNLLGVPDADRAFLLSLTKTALSADDESISETESAM

ARNEILLYFQDLMEFRRDHPGEDVVSMLVNSTIDGAPLSDDDIVLNCYSL

IIGGDETSRLTMIDSVNTLAAHPKQWRRLKDGQCEIDKAVDEVLRWASPS

MHFGRVAAKDTILRGVQIRADDIVTLWHASGNRDERVFHRPEVFDLGRTP

NRHLSFGHGPHYCIGSYLAKVEISELLIALRDLTSGFETTGEPQRIRSNL

LTGFSTMPVRFVPDRAGLARDALDG

>CYP162P1(2515702817)*Salinispora arenicola* CNH964

MGLPSEVDLAEPDLADPDLYAEGDPDAEWAWLRAHRPVYRNPAGATAEFW

ALTRYRDALQVYRDPSTFSSERGMVLGVDPVAGDPAAGRMLVVTDPPRHP

KLRRIVSGIFVPRTMHRLEGRVRSLVDQLLHRVVDGAGRCDFANDVAARL

PVAIICELLGVPADEQDWMYHLTSTAFGGGDPAGSAEVSAVERAEAYGDI

LLYYGELAAERRRRPGDDLVTLLVHADLDGEPLDVEDVLVNCTNLIIGGN

ETTRHAASGGLLALAQRPELWRRLRETPTAVPTAVEEVLRWTTPGMHVLR

TATRDTEIGGVPIRAGERVVVWNAAANRDEDVFADPQRFDIDRSPNRHIA

FGQGGHHCLGAALARLELTILFEEMAKQVTNVRLTGPVRRVRSCVLRGIR

ALPVELVT

>CYP211B19(2561667155)*Salinispora pacifica* CNT403

MDASEAVALLMSPPGRRDPYPTYERLRAHGPVVATAAGFVVTGYTEADTV

LRNPRFGVMDDEERDGVFPDWQDSPAMMSISQSMIRANPPDHSRMRRLAA

GVFTPRRVAALREVVAAQADELIDELIRAGRGGAPVDFLGSFAYPLPVTV

ICALLGVPAADWAQFRRWASELTAVLEPEITPQELAVADAGATELRDYFT

ELIAQRRRDPADDLTTALVQAHDADGDRLSGEELLANLVLLLVAGFETTT

NLLGNGLFVLLTHPDSATALRGQPELAPSYVEELLRYDSPVQLTSRTVRE

SVSLGGVELPAGSWLLVLLGAANRDPARFTDPARFDPGRAQSPPLSFGAG

AHYCLGAGLARLEAQVAFPLLLRRLPELSLAGEPVRRSRLTLRGYETLPV

TVGAVAADHGTPAGAALGTP

>CYP105AB29(2561667974)*Salinispora pacifica* CNT403

MTETASITTPGTSSTTTSGPASGQVTDAEFPVERGCPFSTPVEYEQIRDH

SPLAKVRLTTGREAWWIAGHELGRAVLADRRFSSDRRRDNFPFVSTDPET

RKQLQDQPTSMIGMDGAEHAQARRALMGEFTVRRMAGLRPRIQQIVDQHI

DEMLSSEQRSADLVEALSLPVPSLVICELLGVPYADHDFFQAHSGPLIRH

TTPSEVRLRIQEELNTYLGALIDRKVTDPTDDLLSRQIAKHHAAGTFNRT

SLVSMAFLLLIAGHETTANMISLGVVGLLQHPDQLAMIKEDPEKTPPAVE

ELLRYFTIADTVTARVATEDVQLGDTTINAGDGVVISGLAADHDPTVFTD

PDRIDLERGARHHVAFGFGPHQCIGQTLARLELQIVFDTLFRRIPTLRLA

APLDDIPFKSDAFVYGAEKLPVAW

>CYP244A5(2561668267)*Salinispora pacifica* CNT403

MSATTNAELGEAPETSMPIDPGLFDCMPDLIAAARIAPVVRIPYLGRHAW

VVCDRELVKQALTHPKMGKDIALVPEWMRQPGLMVTAQPPPEYARAMIMS

DGENHARIRRIHAPVLSPRNTERWGEQVADKVEGFLDELSKAAAGSNAEV

DVVTNYTHKIPLAFISEMLGLPPAAEHRLRSITDIMLYSSDYAARREAIG

GLFGAVEEWVRNPDGLRDGVITGLLAGSDGPGAAVTEGEVIVWTLGMIIT

GYETTGSLISTSLYEALRRPPHERPRTDEDITAWIEETLRVHPPFPHPTW

RFPLEDIELGGYLIPKGAPVQVSIAAANRQPGEGADSFDAERRGHGHLSF

GLGMHYCIGAPLVRLEAKIAVRGFLRRFPQARLSADTAVQWESEWMIRRM

SSLPAVLS

>CYP245A11(2561668271)*Salinispora pacifica* CNT403

MSSTTLPRFTLTGWNREDIVNPYPVYRRYREVAAVHRGEAGSDAPETFYV

FSYDQVAQVLSSSCFGRGRSLDATAASVPVPADQKALRAVVENWLVFMDP

PRHTELRSLLNRSFSPRIVTGLRPRIARIAQELLSRLGRQVETDLVEGFA

APLPILVIAELLGIPAGRHGWLRTNALALQEASSSRARRDTAGYARAEAA

AQEFTEYFREQVRLRRGSTGDDLLTILANAQLRGVPVSLDAVVGTAVHLL

TAGHETTTNSLAKAVLALQAHPAVLEELRGADGLTADSIEEFLRYDPPVQ

AVTRWTHQDTTLGGWEVPRGSRVVALLGSANRDPARFPLPDALDVHRPAD

RHLGFGLGIHYCLGATLARAELEIGLQTLLNGLPTLGHPAQYVDYADDMV

FHGPSRLILVNPGERFCQ

>CYP107AW6(2561668354)*Salinispora pacifica* CNT403

VETVTGTSAPPPVPYIADPYPTLARIRANGPVSILHSDEGVPMWVIARYR

EVRAALADPRFGQDARRAQALADNRVAGVTLGGDIVHMLNSDPPDHTRLR

RHVQGAFTARRVAAMRPLVERITTSLLDGLAGRKSVDLVQDLAFPLPMLV

ICELLGFPAEERNAYRSWSTAILTHDDDPAVFATALREMTDYIAVQLRLR

RSRPGDDLLTELLAERDAGQLTDDEIVGMVFLLLIGGHETTVNLLGTATL

ALLRNPDQHRWLLANGHALPEAIDEFLRYESPVAMATLRFTTTPVTVDDV

VIPAGELVLVSLGGANRDPDRFPDADRLILDRRDTGHLAFGHGLHRCLGA

FLGKLEGEVALGALLRRHPKLALATEVRQLQWRDTIMLRGLESLPVSLHG

>CYP107AX9(2561668367)*Salinispora pacifica* CNT403

VTAQPAPVFDQRLLRDPHRRYNALRDQAPVHRVRTPDGAPAWLVTRYDDV

RAAFIDPRLSVDKRFSGTDGEHGSSLPPELDAHLLNRDPPDHTRLRRLAA

AAFTPRRVADLRPAVEKTVSTLLDGLAGNDHAELIGSLASPLPLQVMHEL

LGLPTQTSVDFRTWTNTLLSADANQPAQSRSAMANMRRFLIEQLAHKRAQ

PGDDLLTGFLCVREDDDGLTDDELVAMLFLLMFAGYDNTAALIGNVIHAL

LTNVELAEAVRTGSLAVDELVDGVLRWNPSFPLAVRRFAREPITIAGQTI

PAGDRIWLCLASANRDPAHFTEPDEIGIADMRRPHLSFGHGIHYCLGAPL

ARLQTTVAVASLFDRFPGIRLAVPVQDLQWRESFRLRGLVALPVSL

>CYP125A66(2561668783)*Salinispora pacifica* CNT403

MTEPRIPVGFDFTDPAVLERRVPREEFAELRRTAPVWWNAQPKGSAGFDD

DGYWVVTRYADVMAVSRDSETYSTRENTAIARFQPGTTQADREMQRVIMI

NVDPPEHTKLRAIVSRGFTPRAINALRGSLGERAERIVRDAAGRGTGDFV

TDVACELPLQAIAELIGVPQHHRRKVFDWSNQLIGYDDPAYGVDPLAASA

ELLAYAMEMAHERQRNPSDDLVTKLVNAQIDGEHLTADEFGFFVMLLAVA

GNETTRNAITHGMLAFLENPEQWELFKAERPRSAVEEIIRWATPVNVFQR

TALVDTTLAGQAISAGQRVALFYGSANFDESVFEEPERFDITRSPNPHLG

FGGSGVHFCLGANLARLEIELIFNSIADHMPDIRKVADPQRLRSGWINGI

RELPVRYH

>CYP211C6(2561668914)*Salinispora pacifica* CNT403

VPDIEGLLTRLYSAQGRQDPYPVYADLHAQAAIAALEPRPERQRVAAVAV

GYDLVAAVLRDPEWFKQPPPDWRDQEILRILQSSMMFINPPDHGRMRHVF

AGTFTPRRLGALEPVINRVTDELLDRMADAGPGEVDFVAEFAYPLPARVM

AEFIGIPATELAWYRDRVDRVDAFLDVAGKTPERLAAANAAGAELRSFYR

ELLAHRRRTPGEDLISGLVEAVDAGGVELTEDELISNLIVLFNASFVTTV

YLLSNGLPVLLAHPEVAAALASSPELAAGAVDEILRLQTPVHLLARAAPR

DMVLGGVSIPQGQSVLLLIAAANRDPAHFPDPDRFDPRRSSPPSLAFGLG

LHYCLGATVSRLEGRLALPRLLSRFPRLRILEQPVYSGSLFLRGIDKLSV

SPGGREHP

>CYP208A26(2561669302)*Salinispora pacifica* CNT403

MTVIADRGGRVPPGPPVTAGLRLLLALGRDRLGMLTSAATEFGDVARLPV

GPKRLYFFNHPDHAKHVLADNHANYAKGIGLVHARRALGDGLLTSDGELW

RKQRRVIQPAFLSRRLAQYAGVVGQEATRLAERLATRVDGPPVDVLDEMT

RLTLGVLGRTLLDAELTGFHGVGESFAAVQDQAMFELETLSAVPTWIPLR

RQRRFRRARRHLQEVVDVLAAERGQNVEGRDDVLSRLILSTRAEADAALG

RQRLRDELVTLLLAGHETTASTLGWSLYLLDQHPQLRERVRHEATSVLGD

RVPGYADLHQLQYTAMVVQEAIRLYPPVWILTRKARAEDEVGGYRVPAGA

DLLICPYTLHRHPRFWEEPGRFDPERFDPARLTNRPRYAYVPFGAGPRFC

VGNNLGMLEATLVLAVLLRDLRLEGLPGRAVVPEPMLSLRVRGGLPMMVR

RAD

>CYP105BL3(2561669578)*Salinispora pacifica* CNT403

MSSHSAAAPGPETATPLHTLAPELTFPQFERATPFDPPEAYTELSGRCPV

APVRMADGKPSWLITSFEGVRAALSDPRLSSDMSHPGFPNRTGKPVDDLL

KDTLGAMDGERHRYYRRMLTGELTVRRAKAMRPVITQITDEALDQLAAAG

PGADLVTHVALVVPSRVACHLVGIPLSEYELFTGMAAKLMDSTSNADQIA

ALQDMVSYFDTLVTDREHHDRDDLLGHMVRRYLATGELDREMLIRLAWTT

MAAGQETTAHMIGLGVAALLRHPDQLELLRREPHLMPGAVDELMRYLPMI

QFGIPRVAMDDVEVDGQTVTAGEGVVALPPLANRDPAVFERPDELDVRRN

ARQHLSFGYGPHQCPAHALARLELEVVYGRLLERFPTLRLADSDADLKVQ

DEDIMYSVSELAVAW

>CYP163B20(2561669611)*Salinispora pacifica* CNT403

VTATEAKYVGQVGSRNAASTIDLADPATFAGHDLTNFWQRLRDEEPIYWN

PPTGGRRGFWVLSRHADILEVYRDDMTFTSERGNVLVTLLAGGDAGAGRM

LAVTDGPRHAELRKLLLRALGPRVLGPVCRAVRANTRQMIGEAAANGECD

FATDIASRIPMITISNLLGVPEADRASLLKMTKTALSADDESISDTDSEM

ARNEILLYFQDFVEFRRKNPGEDVVSMLVNSSIDGVPLSDDDIVLNCYSL

IIGGDETSRLTMIDSINTLAANPGQWRRLKEGRCDIDKAVDEVLRWASPS

MHFGRTAVRETVIHGERIQVDDIVTLWGASGNRDERAFKRPEVFDLDRAP

NRHLSFGHGPHYCIGSYLAKVEISELLIALRDLILGFEVIGEPQRIRSNL

LSGFSTMPVRFHADRTGLASEAREG

>CYP162A8(2561670030)*Salinispora pacifica* CNT403

MISPDLSDPTFYQNDDPLPVWAELRREHPVYDNRRADGTRIWAVMTHKLC

TDMLTNPKVFSSANGMRLDSNPRVLARAAGKMLNVTDPPQHDKIRKLVSS

AFTPRTIRRLEANMRATAGRAIDTAIADGGCELTRLAHKLPVSVICDLLG

VPEQDWDFMAERTRFAWGSTAADDREEVEKVAAHTEIMAYFLDLAADRRK

RPMEDLVSALVHGEVDGRALTDEDVLYNCDALLSGGNETTRHATVGGFLA

FVMNPDEWSAIRQNRDLLPSAIQEIVRYTSPVMHSLRTATRDVEFGGINI

RAGEYVVAWLPSANRDEQLFDDPDRFNIRRSPNRHLGFIQGNHYCIGAGL

ATMELRVMFDELLNRVSEVRLAGPARRLRSNLLWGFDSLPVTFHVGAGR

>CYP1269A2(2561670223)*Salinispora pacifica* CNT403

MTTPRTDRPYGTPGIGSAVLERGTVRTEGMALDAGQRRRLQLAQGLLWLR

AASGDPFAALLRDHDEDEAALYARLGGGAPIWRSSTGTWVVTRHRTATAL

TADPAVEDRIVGWRPLGIPVMPLGEQDLGLHPENREALLSLARAEVAKDA

LVERRPRWAVAVRRRLDRAGDGRIDLAELAAGISVDMLAEQLDLPGTEMG

WFSDLASHAGLAADGLLCPQDMRRTHDMLAGIAELRERFAGRPFAMILAV

FGVRVCADLLRNALAALLAEPAGWAGLRAEPASAAAVVDEILRHDPPVRV

QLLEAVADTTAHGVPITAGDQIAVLLAVANRDAEVFPEPDVYRPGRPVSP

DRPVLLPGWPAGPVLPLARAQTVTALTELAGRFPVLEAAGPVIREVRAPV

TRAIRELPARADRRVRRP

>CYP107AY7(2561670567)*Salinispora pacifica* CNT403

MRAEPAPIPRSGARLGQEYDQLRKAGDVHQVLLPDTSLAWLVTSPELVSR

ALADPRLALNRKHSRGGWSGFALPPALDANLLNLDAPDHTRLRRLVGPAF

SPQRVAALRPRIERTAEELVDTVVATGSPVDLVTGYCTPLSVQVIADLLG

VPEAGRTDLRAWTDTMLTSYPPDRDAIRQAVVELHGYVVNLIEAKQKRPG

DDLLSALVATEQEGDRLTRDELTSLAFLILFAGYENTANLIASTVLRLLD

HGSLRGVRASEAIEETLRLEPPAPVAIRRFPIEETTIGGATIPAGDTVLL

SIAAATRGTDGNSARLAFGNGPHFCLGAALARVEAEEAITVLARRLPSLA

LAVPGAPVRWRPTFRTHGPAELLVTW

>CYP208A21(2561671387)*Salinispora pacifica* CNT403

MTTTSIDHRRPPGPPRAAALSMLLTMSRDRLGMMTAAARAYGDAAWLPVG

HKALYFFNHPDYAKHVLTDNSDNYTKGIGLVHARRALGDGLLTSEGELWR

KQRRVIRPSFRSGRAPDQASVIAEEVASLVERLRARAGGPPVNVVTEFTG

LTLGVLGRTLLDVDLTALATVGDAFAAVQDQAMFELVTLSAVPTWIPLRR

QRRFRRARAELERIVDDLVARRGDVSGRDDVLSRLILSTGAEPDARVRRQ

RLRDELVTLLLAGHETTASTLGWTLYLIDRHPPVRERLRAEAVEVLGDRL

PAYRDLPDLRFTTMVVQEAMRLYPPVWLLPRRSRRADRVGPYRVPAGSDV

VVSPYTMHRHPGFWPEPDRFDPLRFDPRNAADRPRYAYLPFGAGPRVCVG

SNLGMMEAVIAVAMLCRELRLVRVPTHAAVPEPMLSLRIRGGLPMSVHLA

D

>CYP154M16(2561671398)*Salinispora pacifica* CNT403

MPDRCPVLDPSGRDIHAEADRLRAQAPAVKVELPGGVHAWSITSYDVVKR

LLLDRNVTKNARNHWPKFINDEIPPDWEMISWVAMDNMVTAYGKHLVRLR

RLIAKAFTAQRVETVRPQVEKLVDELLDGLAAETGEVVDLREKFCYPLPA

LLIAELIGMTEQQRAQTAKAMDLMVDTTVSPDQAQAILTGWRTAMDELIA

AKRREPGKDIASDLIAARDDENGGQLTDSELTDTIFAILGAGSETTINFL

DNAVTALVTHPGQLELVRSGRAGWDDVIDEVLRVQCPLASLPLRYAVTDI

ELDGVTIPQGDPILINYAAAGRDPALHGDTAGEFDVFRENKEHVSFGHGP

HYCLGAGIARLVATIGLSRLFERFPDLRLAVPAEELQPLPTFIMNGHRAL

PVRLVPAPAAATAV

>CYP1005A8(2561671476)*Salinispora pacifica* CNT403

VSAVLFRSWTKTVGTHWPAVTRVADQQGTEHLVVTEHELVRQVLTDQVTY

RPDNALDAVTPIPVPALRVLAGHRFRLPPTLANNGGVSHPAIRALVADAL

HPAKVAAQRPWLTKLVAERVAAIGATLDSGGAADLHAELSADLPLLVLAR

LVELPDAPVSAVKQFARAALALFWAPLDADRQLALADEVGRFHQVLREFA

DTGGGLAAALRATGHPPDVLVGALFFLLVAGQETTSQFLTLLLHRLAGEP

TVRAALRDGGVSVANVVEEGLRLEPPIVTWRRVAAVDSTLGGTAVPAGTN

VLLWLARAGRDPAIVPAPDEFRPGQRGSRRHLAFGAGAHRCLGDQLARME

AAVVVEQAAPLLDGISVVRAPWYPDNLTFRMPDAFVIRR

>CYP107CT3(2561671528)*Salinispora pacifica* CNT403

MTGASGDRTGQCPIALDHDFYADPHPAYRVIKENGNKPTPIVLKTGMAYL

PPGLEAWLVTAYEDVEFVLRDPRFRKSIDEAMPLFAAQSGGTVAARGSLL

YNNMANNDPPVHTRLRKPLNAMFTSRSVAGRRDVVRSAALETLEKVAGSQ

TFDLVQDFAFPFSISVISRTLGVPDADRGTFHGWVQTITGAAPPEILRRD

AGLMVEYLRGLIRGRRESAGNGTDDVLTLLATSLAEDEAVAQAYALLAAG

YETTANLIVTGFLTLAAHPEQLRRLWSDPGLVPDAVEEMLRHQSPFNLSL

YRYVTEPVELNGVRIPRGAIVFLAFAAANRDECRFTEPDSFDITQPHRDH

LAFGGGIHNCIGKHLARMEAQVAFETLIERCPGLAVHTPDEEFDWKASPT

FRGLRTLRVGPGPAPGGWEREQ

>CYP1005A6(2550580746)*Salinispora pacifica* CNT-133

IVPAPDEFRPGQRGSRRHLAFGAGAHRCLGDQLARMEAAVVVERVTLLDG

VTVVRAPWYPDNLTFRMPDAFVG

>CYP1005A6(2550581507)*Salinispora pacifica* CNT-133

ADEVGRFHQVLREFADTGGGLAAALRATGHPPDVLVGALFFLLVAGQETT

SQFLTLLLHRLAGEPTVRAALRDGSVSVPNVVEEGLRLEPPIVTWRRV

>CYP208A30(2550581741)*Salinispora pacifica* CNT-133

MTVTAAGRTFSGPTGSALLRSLWQLGRDRLGLMTSAARYGDAVRLGVGSR

SLYFFNHPDHAKHVLADNSGNYTKGIGLVHARRALGDGLLTSEGELWREQ

RRVMQPVFQAKRIAGQAHAVAEEADRLLARLRARRGGGPVNLTDEFTALT

LGVLGRTLLDAKLDAFASVGAAFEEMQNQAMFEMASMSLVPMWVPLPQQL

RFRRARRELERIVGRLVADRTARGEVTGADDALSRLIAATQDEPDPGFAR

RRMRDELVTLLLAGHETTASTLSWTFHLVNQHPWVRGRLRKEAIEVLGDR

LPEYADLARLTYTKMVVSEAMRLYPPVWMLSRLARDADVVDGYPVPARAD

VLICPYTLHRHPAFWPEPERFDPERFDPGAVTDRPRYAYVPFGAGPRFCI

GNHLGLMEAVFVVAMVSREFELVAPVGQPVVAEPMLSLRIRGGLSMTVEP

VS

>CYP107AW10(2550583049)*Salinispora pacifica* CNT-133

VETVTGTSTPPPMPYIADPYPTLARIRANGPVSILHSDEGIPMWVIARYR

DVRTALADPRFGQDARRAQALADNRVAGVTLGGDIVHMLNSDPPDHTRLR

RHVQGAFTARRVAAMRPLVERITTSLLDGLTGRTTVDLVQDFAFPLPMLV

ICELLGFPAEERNAYRSWSTTILTHDDDPAAFATALREMTDYIAVQLRIR

QTRPGDDILTELLAARDAGQLTDEEIIGMVFLLLIGGHETTVNLLGTAIL

ALMRNPDQHRWLLANQHALPKAIDEFLRYESPVAMATLRFTTTPVTIDDV

VIPAGELVLVSLGGANRDPDRFPEADRLILDRRDTGHLAFGHGLHRCLGA

FLGKLEGEVALGALLRRHPRLALAAEVRQLRWRDTIMLRGLESLPVSLHG

>CYP105AB37(2550583866)*Salinispora pacifica* CNT-133

RHTTSTEVRLRIQKELNTYLGALIDRKVTDPTDDLLSRQIAKHHAAGTFD

RTSLVSMAFLLLIAGHETTANMISLGVVGLLQHPDQLVMIKE

>CYP125A65(2550584638)*Salinispora pacifica* CNT-133

MTEPRIPVGFDFTDPAVLERRVPREEFAELRRTAPVWWNVQPRGSAGFDD

DGYWVVTRYADVMAVSRDSETYSTRENTAIARFQPGTTQADREMQRVIMI

NVDPPEHTKLRAIVSRGFTPRAINALRGSLAERAEHIVRDAAVRGTGDFV

TDVACELPLQAIAELIGVPQHHRRKVFDWSNQLIGYDDPAYGVDPMAAAA

ELLAYAMEMANERQRNPSDDLVTKLVNAQIDGEHLTTDEFGFFVMLLAVA

GNETTRNAITHGMLAFLEHPDQWELFKAERPRSAVEEIIRWATPVNVFQR

TALVDTTLGGQAITAGQRVALFYGSANFDESVFEEPERFDITRSPNPHLG

FGGSGVHFCLGANLARLEIELIFNSIADHMPDIRKVADPQRLRSGWINGI

REMPVRYR

>CYP1005A6(2550585481)*Salinispora pacifica* CNT-133

VSAVLFRSWTKTAGTHWPAVTRVADQQGTEHLVVTEHALVRQVLTDQVTY

RPDNALDAVTPIPVAALRVLAGHRFRLPPTLANNGGTSHPAIRALVADAL

HPAKVAAQRPWLTELVAERIAAIRATLDSGGSADLHAELSADLPLLVLAR

LVELPDAPVSAVKQFARAALELFWA

>CYP113B7(2550585872)*Salinispora pacifica* CNT-133

MTQTDNAPMPPLDLPKGADAQGLLDWFAYMREHSPVSWDETRQAWHVFNY

QDYLTVTTNPLIFSSDFSPVFPVPPELALLMGPGTIGGIDPPRHGPMRKL

VSQAFTPRRIAQMEARIETITADIFEQVRGQERIDIAADLAYPLPVTVIA

DMLGIPNEDHEKFREWVDIILSNEGLEYPNLPDDFTETVGPAIAEWSEFL

YAQIAEKRARPQDDLMSGLLAVEVEGRRLTDEEVVNIVALLLTAGHISSA

TLLSNLFLVLEEHPEALAAVRADRSLVPGVVEETLRWRSPFNCIFRLLAQ

DTEIFGQP

>CYP107LA2(2550586648)*Salinispora pacifica* CNT-133

CSGAILARSYPRLSIAAGAGRDVPRASPRIQEAEGLMGMPPEAHARLRRL

VATAFTPRRVRDIAPRVAEIADRLVNDVIETGPPADLVRQIALPVPITII

CELMGVPAEDQHVFRVFSDALMSSTRYTDDEVDQAVQEFTAYLGGLLTQR

RAHRTDDLIGALIEARDDGERLSEEELIMLTGGLLVGGHETTASQIASHL

LVLLRDRARYETLSARPELIPTAVEELLRVAPMWVTVGPTRIATEDLELN

GVTIRAGDAVIFSLSSANLDEDAFPDATDVVLDREPNPHIAFGHGPHFCI

GANLARLEIQSTIGALVRR

>CYP107E37(2550587493)*Salinispora pacifica* CNT-133

VTIDQEIRKYPFRESPGIGIDPTYELLRRTEPLARVQLPYGEVSWLVTRY

EDVKTVLTDPRFSRAAAQGKDQPRTRAEMTYEGIIGLDPPDHTRLRRLAG

KALTARRVNAIRADAQRIANEYVDEMIAKGSPGDLVELFALPYPVTVICE

LLGVPFEDRAQFRIWTEGLTSTSEQLMVYAEQLFGYMGKLVAQRRAEPTD

DLLGALVKARDEGDRLTEQELLSIAGVGLLLTGVETVSTHIPNFVYALLT

HPELMAQLRADRSLVPAAVEELLRMIPLNPAAMFPRYAVEDVTLSGITVR

AGQPVLVSLPGANRDPEVFENPETFDFTRDQNPHVAFGHGPHHCLGAQLA

RMELQVALHTVLDRFPDLRLADGDEGVSWKSGLLVRGPSRLLVGW

>CYP163B20(2585373130)*Salinispora pacifica* CNT-133

MTATDAKDVGQVGSRNVASTIDLADPATFAGHDLTNFWQRLRDEEPIYWN

PPTSGRRGFWVLSRHADILEVYRDDMTFTSERGNVLVTLLAGGDAGAGRM

LAVTDGPRHTELRKLLLRALGPRVLGPVCRAVRANTRQMIGEAAANGECD

FATDIASRIPMITISNLLGVPEADRASLLKMTKTALSADDESISDTDSEM

ARNEILLYFQDFVEFRRKNPGEDVVSMLVNSSIDGVPLSDDDIVLNCYSL

IIGGDETSRLTMIDSINTLAANPGQWRRLKEGRCDIDKAVDEVLRWASPS

MHFGRTAVRETVIHGERIQVDDIVTLWGASGNRDERAFKQPEVFDLGRVP

NRHLSFGHGPHYCIGSYLAKVEISELLIALRDLILGFEVIGEPQRIRSNL

LSGFSTMPVRFDADRTGLASEAREG

>CYP211B11(2517949349)*Salinispora pacifica* CNQ768

MDASEAVALLTSPPGRLDPYPTYERLRAHGPVVSTAAGFFVVTGYTEADT

VLRNPRFEVMDDEERDGVFPHWQDSPAMISISRSMIRANPPDHSRMRRLA

AGVFTPRRVAALREVVAAQADGLVDEMIRAGRGGAAVDFMGSFAYPLPVT

VICALLGVPTADWARFRHWASDLTGVLEPEITPQELAIADAGASELRDYF

TELIAQRRRAPADDLTTALVQAHDADGDRLSGEELLANLVLLLVAGFETT

TNLLGNGLVVLLTHPDSATALRGQPELAPGYVEEFLRYDSPVQLTSRTVR

ESVSLAGVELPAGSWLLVLLGAANRDPARFTDPARFDPRRAQSPPLSFGA

GAHYCLGAGLARLEAQVAFPLLLRRLPELALAGEPTRRHRLTLRGYETLP

VTVGAVPADPGTPAGVALGTP

>CYP124M3(2517949393)*Salinispora pacifica* CNQ768

MTVPIELDAVDLSDNDFWTKPLEYRHAVFNALRAQPGLPHFASPEFGSAP

RGRGYYALTRMDDVLAVSRNPSVFISGKGNVAMEVPAEFLDQSLITMDNP

RHARLRRIVSRGFTMKAVTALMDNASQMAKQIVDEVVERGECDAVVDISA

KLPLGIICNMMGIPDSQQQFVFEQTNVMLGVQDPEYVGEQNDAMLAMSVA

SQELADLMHELATLRAKNPTDDLISKLLSAEVNGEALTPSELAHFFILLT

GAGTETTRNAISWGIQLLTQSPEQREAWLSDIDGVTPTAVEEIVRWSSPV

ISQRRTVAEDAEPVKLAGQLLGPGDKVLMFYGAANRDPNYFVHPEGFDVR

RSPNPHVGYGGPGPHFCLGAHLARLEISVMFRELLTRIPDIHATGEPARL

KSPLINGIKRLPVAFTPGAR

>CYP208A22(2517949414)*Salinispora pacifica* CNQ768

MTVIADRGGRIPPGPPVTAGLRLLLALGRDRLGMMTSAAAEYGDVARLPV

GPKKLYFFNHPDHAKHVLADNHANYQKGIGLVHARRALGDGLLTSEGELW

RKQRRVIQPAFQNRRLAQYAGAVGQEATRLVARLATRVDGPPVDVLDEMT

RLTLGVLGRTLLDAELTGFHGVGESFAAVQDQAMFELETLNTVPTWIPLR

RQRRFRRARQHLQEVVDVLAAERGQAVEGRDDVLSRLILSTRAEADPQLG

RERLRDELVTLLLAGHETTASTLGWSLHLLDQHPELRERVRHEARTVLGD

RVPAYEDLHQLRYTAMVVEEAIRLYPPVWILTRKARAEDEIGGYRVPAGA

DILICPYTLHRHPRFWAEPERFDPERFDPSRTTDRPRYAYIPFGAGPRFC

VGNNLGMLEATLVLAVLLRDLRLEGLPGRAVVPEPMLSLRVRGGLPMTVR

RVD

>CYP125A65(2517949509)*Salinispora pacifica* CNQ768

MTEPRIPVGFDFTDPAVLERRVPREEFAELRRTAPVWWNVQPRGSAGFDD

DGYWVVTRYADVMAVSRDSETYSTRENTAIARFQPGTTQADREMQRVIMI

NVDPPEHTKLRAIVSRGFTPRAINALRGSLAERAERIVRDAAVRGTGDFV

TDVACELPLQAIAELIGVPQHHRRKVFDWSNQLIGYDDPAYGVDPMAAAA

ELLAYAMEMANERQRNPSDDLVTKLVNAQIDGEHLTTDEFGFFVMLLAVA

GNETTRNAITHGMLAFLEHPDQWELFKAERPRSAVEEIIRWATPVNVFQR

TALVDTTLGGQAITAGQRVALFYGSANFDESVFEEPERFDITRSPNPHLG

FGGSGVHFCLGANLARLEIELIFNSIADHMPDIRKVADPQRLRSGWINGI

REMPVRYR

>CYP105AB28(2517949985)*Salinispora pacifica* CNQ768

MTETASTTTPGISSTTTSGPASGEVTDAEFPLERGCPFSTPTEYEQIREH

SPLAKVRLTTGREAWWIAGHELGRAVLADRRFSSDRRRDNFPFVSTDPET

RKQLQDQPTSMIGMDGAEHAQARRALMGEFTVRRMAGLRPRIQQIVDQHI

DEMLSSDQRTADLVEALSLPVPSLVICELLGVPYADHDFFQARSGPLIRH

TTPTEVRLRIQKELNTYLGALIDRKVADPTDDLLSRQIAKHHAAGTFDRT

SLVSMAFLLLIAGHETTANMISLGVVGLLQHPDQLAMIKEDPEKTAPAVE

ELLRYFTIADTVTARVATEDVQLGDTTINAGDGVVISGLAADHDPTVFTD

PDRLDLERGARHHVAFGFGPHQCIGQTLARMELQIVFDTLFRRIPTLRLA

APLDDIPFKSDAFVYGAERLPVAW

>CYP107AX4(2517950449)*Salinispora pacifica* CNQ768

MTSQPAPVFDQLLLRDPHRRYNALRDEAPVHHIRTPDGAPAWLVTRYDDV

RAAFTDPRLSVDKRLSSTDGEHGSSLPPELDAHLLNRDPPDHTRLRRLAA

AAFTPRRVADLRPAVERIVSTLLDGLVGHDQAELIGSLASPLPLQVMQEL

LGLPTQTSVDFRRWTNTLLSADANQPAQSRSAMANMRRFLVEQLAHKRAR

PGDDLLTGLLAAREDDDRLTDDELVAMLFLLMFAGYDNTAALIGNVVHAL

LTNTELPAAVRTGSLALDELVDGVLRWNPSFPLAVRRFAREPITIAGQTI

PAGDRIWLCLASANRDPAQFTAPDTLGTTGMRRPHLSFGHGIHYCLGAPL

ARLQTTVAVTILLDRFPGIRLAVPAHDIRWRESFRLRGLVALPVFL

>CYP107AW7(2517950463)*Salinispora pacifica* CNQ768

VETVTGTSTPPPVPYIADPYPTLARIRANGPVSILHSDEGIPMWVIARYR

DVRAALADPRFGQDARRAQALADNRVAGVTLGGDIVHMLNSDPPDHTRLR

RHVQGAFTARRVAAMRPLVERITTSLLDGLTGRTTVDLVQDFAFPLPMLV

ICELLGFPAEERNAYRSWSTAILTHDDDPAAFATALREMTDYIAVQLRIR

QTRPGDDILTELLAARDAGQLTDDEIIGMVFLLLIGGHETTVNLLGTATL

ALMRNPDQHRWLLANQHALPEAIDEFLRYESPVAMATLRFTTTPVAVDDV

VIPAGELVLVSLGGANRDPDRFPDADRLILDRRDTGHLAFGHGLHRCLGA

FLGKLEGEVALGALLRRHPRLALAAEVRQLRWRDTIMLRGLESLPVSLHG

>CYP1005A6(2517951251)*Salinispora pacifica* CNQ768

VSAVLFRSWTKTAGTHWPAVTRVADQQGTEHLVVTEHALVRQVLTDQVTY

RPDNALDAVTPIPVAALRVLAGHRFRLPPTLANNGGVSHPAIRALVADAL

HPAKVAAQRPWLTELVAERVAAIRATLDSGGSADLHAELSADLPLLVLAR

LVELPDAPVSAVKQFARAALELFWAPLDADRQLALADEVGRFHQVLREFA

DTGGGLAAALRATGHPPDVLVGALFFLLVAGQETTSQFLTLLLHRLAGEP

TVRAALRDGSVSVPNVVEEGLRLEPPIVTWRRVAAVDSTLGGTAVPAGTS

VLLWLARAGRDPAIVSAPDEFRPGQRGSRRHLAFGAGAHRCLGDQLARME

AAVVVERVSPLLDGVTVVRAPWYPDNLTFRMPDAFVIRRGPAGAAER

>CYP107FH3(2517951870)*Salinispora pacifica* CNQ768

VPIELDEAFVQDPYAVYEKLRAEGPAHRVRMPPGVPLIGGLPVWLITGYD

AVRAALADSRLSTDLHRIDGLFAQKDPDRSHRGGFSSALASHMMHTDPPD

HTRLRKLVSKAFTRRAIEALRPRIQQTTDELTADLAGYDTVDLLDAFAFP

LPIRVICLLLGVPVAEQENFRSWSRALVSGHSPEAAATAATEVAAYLGDL

VERKRHATTDDVLTALVAAHDVDDRLTHTELVSTAYLLFIAGFETTLNAL

GNGTLHLMLHRDQWTALRADRALLDNAVEEFLRLESPLKHATFRCATESL

RIGDAEIAAGDFVLLAIASANRDPRRFPDPHTLDVRRPAAGHLAFGHGIH

HCLGAPLARVEVRMAFDALLDAFPDMRLATDPAGLRWRNSTIIRGLDSLP

VHLNN

>CYP2054A3(2517951875)*Salinispora pacifica* CNQ768

MSISADSEQVQGRPFDPYGAHRDDPYTFLAGLGVFYAPLLDAWCVTRRED

MVAVLRDDRSFSARDHNPRPAVALPDDVNQMFRTWRGAGAVAVGSLDPPA

HAKIRDVLNIGFTPARVRAFEPTMRAVAADLADRIGDAPEFDFIADFAVP

FALEVIGRRLGVPDDYLDRCRTWSEQRIELMMAQGDADHDRLREFARGLM

EFGEFARSLVRDRVADPRDDLISELLHDGKAGRTLTADEVAVQIPTLIFA

GHMTCAEALGTIFYQQLRSPGGWARVVDRTIPVGDLVEEGLRFDSPLAGM

YRTATRDVTVGGIRLTAGSRLLLLYGAAGRDSRAHACPAAFRPGDGSSGH

LAFGHGIHFCLGAGFARAELRVAVEVLAARMPDLGLAPGRPPRFRPVFPL

RALTELRVTRSGGGSGP

>CYP161N4(2517951880)*Salinispora pacifica* CNQ768

VNTVAQLPFTQTHVLDVAPALRLLQSRGKVHRVRTPEGVPAWLVTGHAEV

QQLLDDDRLSRSDPGGRDGGTALLNKLLGPLADDHPRLRSLLEPQFTPER

LEPLRAVVEKLTEQHLDELATRTPPVDLRPALAMSLPILVLCEWLGVPAE

DKGRFSVWTQDAAGVQDPERSQRGLAELFGYCRQLVAAKRQDPGDDVISR

LIATAGIGDTEVVALTALLLFGGYETTVARIGTGVLLLLTNPDQWAAVRA

DPALVPATVDELLRRSMPNPHNGGMPRFAVTGFEIDGAAIRAGDLVLLNI

IAANHDETAFPDPDRLDITRPTAGSLAFGYGRHSCVGAPLARMVLRVALS

RLITRFPDLRLAVGVDELKLRHETLVGGLVELPVTWGPR

>CYP107AY9(2517952012)*Salinispora pacifica* CNQ768

MSQDQPTRAELAPIPRSGARIGPEYDQLRKTGDVHQVLLPDTSMAWLVTS

PDLVSRALADPRLALNRKHSRGGWSGFALPPALDANLLNLDAPDHTRLRR

LVGPAFSPQRVAALRPRIQRTAEELAETMVATGSPVDLVTGYCTPLSVQV

IADLLGVPDAGRTDLRAWTDTMLTSYPPDRDAIRQAVVELHGYVVNLIEA

KQQRPGDDLLSALVATEQEGDRLTRDELTSLAFLILFAGYENTANLIAST

VLRLLDHGSLRGVRVSEAIEETLRLEPPAPAAIRRFPTEEMTIGGATIPA

GDTVLLSIAAATRGADGNPARLAFGNGPHYCLGAALARVEAEEAITVLAR

RLPGLALAVPGAPVRWRPTFRTHGPAELLVAW

>CYP244A10(2517952032)*Salinispora pacifica* CNQ768

MQDTAQANLAEAPEVRMPIELRPTDCLPELLAAARVAPVVRTPYLDQHAW

VVCDRELVKQALTHPKLGKDVALAPDWMRQPGQMVTAMPPPEYARMMVMS

DGEHHARIRRIHAPVLSPRNTERWSERVAALVDGFLDNLDSADGTEVNLI

TDYTHKIPLAFTAEMLGLPPGAERRLHDITEVMLYSADYALRQQAVGELF

EAVQEWVRDPAGLRDGVVTGLLASADGPDATVTKGEAIVWTLSLIINGYE

TTGSLISAALYEALRRPARERPHTDEAVAAWIEETLRVQPPVPHTTWRFA

LADLDLGGYLIPRGAPVQISLAAANLDPDEDADSFDAQRRGRGHLSFGLG

AHYCIGAPLARVQTKIALRGFLRRFPQARLSLDTAPRWESEWMIRRMSVL

PVLLA

>CYP107E37(2517953525)*Salinispora pacifica* CNQ768

VTIDQEIREYPFRESRGIGIDPTYELLRRTEPLARVQLPYGEVSWLVTRY

EDVKTVLTDPRFSRAAAQGKDQPRTRAEMTYEGIIGLDPPDHTRLRRLAG

KALTARRVNAIRADAQRIANEYVDEMIAKGSPGDLVELFALPYPVTVICE

LLGVPFEDRAQFRIWTEGLTSTSEQLMVYAEQLFDYMGKLVAQRRAEPTD

DLLGALVKARDEGDRLTEQELLSIAGVGLLLTGVETVSTHIPNFVYALLT

HPELMAQLRADRSLVPAAVEELLRMIPLNPAAMFPRYAVEDVTLSGITVR

AGQPVLVSLPGANRDPEVFENPETFDFTREQNPHVAFGHGPHHCLGAQLA

RMELQVALHTVLDRFPDLRLADGDEGVSWKSGLLVRGPSRLLVGW

>CYP208A12(2571086696)*Salinispora arenicola* CNS325

MTLDTITPRVPLGPPRTAALRMLLVMKRDRLGMLSSAAARYGDASRLPVG

HKALWFFNHPRYAKHVLADNSANYHKGIGLVHARRALGDGLLTSEGDLWR

KQRKVIQPAFQSRRIAQQAGMIAEEAFALVERLRARAGAGPVELTAELTG

LTLGVLGRSLLDADLAGFDSIGDSFATVQDQAMFELETLNAVPMWIPLPR

QIRFRRARRKLQAVVDTLVDGRAGNLADRVDVLSRLILSARGEADPRVGR

ERLRDELVTLLLAGHETTASTLGWTLSLIDRHPGVWERLHAEAVEVLGDR

LPEYDDLRRLRYTVMVVEEAMRLFPPVWLLPRRALAPDTIGEYRVPANAD

VVISPYTLHRHPEFWPNPERFDPERFAPGQAADRPRYAYLPFGAGPRFCV

GNNLGMMEAVFVIALLCRHLRLTGVPGYRLVPEPMLSLRIRGGLPLVVRP

VS

>CYP107AY2(2571087154)*Salinispora arenicola* CNS325

MTAEPTPIPRSGARLGQEYDQLRKTGDVHQVLLPDASLAWLVTNPEVAAR

ALADPRLALNRRNSRGGWSGFALPPALDANLLNLDAPDHTRLRRLVGPAF

SPQRVAALRPGIRRAAEHLLDTLVATSGPTDLVTGYCNPLSVQVIADLMG

VPEAGRTNLRAWTDTMLTSYPPDRDAIRRAVTELHGYVVDLIDIKQQQPG

DDLLSTLVTIEQDGDRLSRDELTSLAFLILFAGYENTANLIASAVLWLLD

HGGLNVVPISEAIEATLRHEPPAPVAIRRFPTEDIIIGGVTIPAGDTVLL

SVAAATRGADGNAARLAFGNGPHYCLGAALARVEAEEALTVLARRLPGLT

LAVPPSQVRWRPTFRTHGPAELLVGW

>CYP105CT1(2571087186)*Salinispora arenicola* CNS325

MNSPNHMPADRSLTAPTSGCPMALSRGRVGLDVADEISELRDGGRLGRIT

TAFGQEATLITRYDEVRAQMADSVVFNVAGVPSPPALVDGGFDTESVRRR

RTVGNLIMLDPPEHTRLRRMVAAWFTTRRVERLRPRVVEIIDAALDEMER

SGPPVDLVAMFAKTVPITVICELIGVPEELRERYRRRAERAVSASAVSTP

LDELRRLREAGWVSRELIEYHRENPSDDIIGMLLREHGTDSYDDGITDDE

LVGLANALLIAGHETTTQMLSMGTLALLRHPDQLALLRDDPSIVAGAVEE

LLRYVGVLHGGFVRVATRDTRLGGHRIHAGELVVPALTAANRDPRLLTDG

DRLDITRPPTSHVAFGHGVHFCIGAPLARMELREAFPALLRRFPGLRLAV

PDSELEFTQGTTVYSLRGLPVTW

>CYP154M5(2571087203)*Salinispora arenicola* CNS325

VEQSCPYKLDVTGRDVHAEGEAIRARGPVAQVELPGGVQGWSVTGYQAAR

QVLADPRFAKDPKKWPAYTSGAIPPNWPLIGWLLMDNMTTNDGADHQRLR

KLVSHGFTPRQVERTRPLIVKIVNDLLDGLSSAGPDEVVDLKGRFATPLP

ARVICDMFGVPEALRASVLRGAQVNVTSSISGEEAEANVEQWHRELLELV

EAKREKPDEDMASLLIAAKEEDGSTLTQEEVVGTLHLMLGAGSETLMNAL

SYAVLGMLSNPGQYEMVRNGTSSWDDVIEETLRAQAPVAQLPLRYATEDV

AVGGAVIKAGDPVLMGFTAIGRDPAVHGETAGDYDITREDKTHLSFGHGV

HFCLGAPLARLELKIALPALFERFPHMTLAVRPDQLEPQGTFIMNGHREL

PVRLGQPATVLA

>CYP125A41(2571087405)*Salinispora arenicola* CNS325

MTEPRIPAGFDFTDPEVLAHRVPREEFAELRRTAPVWWNAQPRGSAGFDD

DGYWVVTRYADVMTVSRDSDTYSTRENTAIARLRPDTTREDIEMQRVIML

NVDPPEHTKLRAIVSRGFTPRAINALRGSLAERAEHIVRDAAVRGVGDFV

TDVACELPLQAIAELIGVPKHHRRKVFDWSNQLIGYDDPAYGTDPLTASA

ELLAYAMEMAEERQRSPSDDLVTKLVNAQIDGEHLTTDEFGFFVMLLAVA

GNETTRNAITHGMVAFLDNPEQWELFKAERPKSAVEEIIRWATPVNVFQR

TALVDTVLGGQAISAGQRVALFYGSANFDEAVFEDPERFDITRSPNPHLG

FGGSGAHFCLGANLARLEIELIFNSIADHLPDIRKVAAPQRLRSGWINGI

RQMPVRYR

>CYP211C1(2571087554)*Salinispora arenicola* CNS325

VVDVEELLTRLYSAQGRQDPFPVYADLHAQGPIAALPPEPERRRVAAVAV

GYDLVGAVLRDPEWSKAPPPGWTEQEILRTLQTSMMFINPPDHGRMRHVF

AGTFTPRRLGALEPVVNRVADELLDRMADAGAGGLDFVAEFAYPLPARVM

AEFIGIPETELDWYRERVDVIDAFLDVAGKTPQRLAAANAAGAELRAFYG

ELLARRRRTPGEDLISGLVEAVDAGGVELTEDELVSNLIVLFNASFVTTV

YMLSNGLPVLLAHPEVAAALATDPVLTAGAVDEILRLQAPVHLLARAAPR

DTVLGGVPIPQGQNVLLLVAAANRDPAHFPDPDRFDPWRSGPPSLAFGLG

LHYCLGAAVSRLEGRLALPRLLSRFPRLRIMEQPVYSGSLFLRGIDKLSV

SPGEGSTRE

>CYP211B2(2571087644)*Salinispora arenicola* CNS325

MDVSEAIAVLISPSGRLDPYPTYEQLRAHGPVSRTTAGLFVVTGYAEADM

VLRDPRFVVLDDDLRDDVFPHWQDSPAIKSIARSMLRTNPPDHSRIRRLA

AGAFTPRRVAAMREVVTAQADELVDEMIRAGRDGARVDFMDMFAYPLPVA

VICALLGVPAADRSRFRRWAGDLTGILEPEITPEELAGADAGADELRDYF

TGLIEQRRRAPADDLTTALVQAHDADGDRLSGEELLANLVVLLVAGFETT

TNLLGNGLVVLLTRPEAAAALRDEPDLAPGYVDELLRYDSPVQLTTRTVR

ESVSFAGTELPADSWLLVLLGAANRDPRRFPDPARFDPGRAQSQPLSFGA

GPHYCLGAGLARLEAQVAFPLLLRRLPELALAGRPSRRTRLTLRGYETLP

ITVGAVTADRGTPAGVAPGTP

>CYP105BL2(2571087965)*Salinispora arenicola* CNS325

MSSHSAAAPDPETATPLHTLAPELTFPQFERSTPFDPPQAYTELSGRCPV

APVSMADGKPSWLITSFEGVRTTLSDPRFSSDMSHPGFPNRTGKPVDDLL

KDTLGAMDGERHRYYRRMLTGELTVRRAKAMRPVITQITDEALDQLAAAG

PGADLVKHVAFVVPSRVACHLVGIPLSDYELFTGMAATLMDSTSSDDQFA

ALQNMVSYFDTLVTDREHHDRDDLLGHMVRRYLATGELTRDMLIRLAWTT

MAAGQETTAHMIGLGVAALLRHPDQLELLRREPHLLPGAVDELMRYLPLI

QFGIPRVAMDDVEVDGQTVTAGEGVVALPPLANRDPAVFERPDELDVRRN

ARQHLTFGYGPHQCPAHALARLELEVVYGRLLERFPTLRLADSDADLKVQ

DKDIMYRVSELAVTW

>CYP105CH1(2571088044)*Salinispora arenicola* CNS325

VSSLPLPTYPKLRDPADPLLPPAEYLAIQSEKPIAKVLLPSGRPTWLITG

HALARQVLTEPCVSVDRRHPNFPYPVPNPDAVVAQVARWTYILLGDDPPL

HTERRRLLISEFTVRQAQAMRPRIQQLVDFHLEQLIAAGPGADFSKHFAM

QVPSAVICEMLGVPFADHDYFQERTALQLRRDVPVAAQKQAIDELLAYFE

QLIQEKSSHPGDDVLSRLIVSNRETEAFDHEALVALGLLLLVGGHETTAN

TLTLATATMLERPEIAEQLRTDPSLMPSAVEEFLRYFSVAVAVSRIATAD

LQVGGQLVRAGESMLLVLNTIARDGTVFPEPHRLDIRRNARNHLAFSHGI

HQCMGQNLARVEMQIALDTVLRRLPGLHLVAPFEELPFKYRHLVWGIEEL

RVAW

>CYP244A4(2571088188)*Salinispora arenicola* CNS325

MSTTTNTELTEAPETNMPVDPGLFDCMPDLIAAARVAPVVRIPYLGRHAW

VVCDRELVKQALTHPKMGKDIALVPEWMRQPGLMVTAQPDPEYARAMIMS

DGENHARIRRIHAPVLSPRNTERWGERVADKVEGFLDELSQAGSGGSTEV

NVVTNYTHKIPLAFISEMLGLPPEAEHRLRGITDIMLYSSDYAARREAIG

GLFGAVEDWVRNPADLRDGVITGLLAASDGPDAAVTEGEVIVWTLGMIIT

GYETTGSLISTSLYEAIRRPPHERPKTDEDITAWIEETLRVHPPFPHPTW

RFPLEDIELGGYLIPKGAPVQVSIAAANRKPGEGADSFDAERRGHGHLSF

GLGMHYCIGAPLVRLEAQIAVRGFLRRFPQARLSAETAVQWESEWMIRRM

SVLPAVLS

>CYP245A7(2571088192)*Salinispora arenicola* CNS325

MPSATLPRFALTGWSRENIVNPYPVYQRYREVASVHRGEPGGDAADTFYV

FSYDEVVQVLSSNCFGRGRSLDAAKASVPVPAEQKALRAIVENWLVFMDP

PRHTELRSLLNRSFSPRIVTELRPRIARIAQELLSRLGQQVDVDLVESFA

APLPILVISELLGIPEERRAWLRANALALQEASSSRAGRDVDGYAQAEVA

AQEFTEYFREQVRLRRGRAGGDLITILANAQERGAPVSLDAIVGTCVHLL

TAGHETTTNSLAKAVLALREHPAVLDELRGAEGLTTDAVEEFLRYDPPVQ

AVTRWAHQDTTLGGCDIPRGSRVVALLGSANRDPARFPSPDVLDVRRPAD

RHLSFGLGIHYCLGATLARAELEIGLQALLDGVPTLGYGTQHVDYADDLV

FHGPSRLVLVNLGERCK

>CYP166A4(2571088462)*Salinispora arenicola* CNS325

MTDAISFELPWARTDKFDPPAVFDALREQRPLARMRYPDGHVGWIVSSYE

LVREVLGDPRFSHSCAVGHFPVTHQGQVIPTHPQIPGMFIHMDPPEHTRY

RRLLTGEFTVRRTSRLTGHVEGVATEQIEVMREHGAPADLVATFARPLVL

RVLSGLVGLPYGERDRYLHAVTLLHDAEADPAEAAAAYEQAGAYFDEVIE

RRRRQPEDDLISTLVGDGELTGEELRNIVTLLLFAGYETTESALAVGMFA

LLHHEDQLARLRADPTKIDAAIEELLRYLTVNQYHTYRTASEDIELHGEV

INKGDSVTVSLPAANRDPARFACPAELDIDRETSGHVAFGFGIHQCLGQN

LARVELRAGLSALLRAFPNLRLAVPADEVPLRLQGSVFAVKNLPVCW

>CYP105W2(2571088687)*Salinispora arenicola* CNS325

MTGYQDRPTGDQPGAPVPSGSTDPGIGAFPLPRRCPFSPPAEYARLRAEH

PVVRLPMLGGDTAWVVSRHADVRQVLSDPRMSADRRRPGFPKFAPTTEGQ

RQASFANFRPPLNWLDPPEHAICRRQIVDEFSVRRVRQSRALVERVVDTH

LDALTAAAPGADLVSTFAYPVPSQVICEVLGVPYGEHEFFERRSTLMFRR

STPADERARCAREIRDFLDMVVTDKEHRPGDDVLSRLLYRQRRAGGVDHE

AVVSMAFVLLVAGHVTTSNMLALSVLALLTHPARLARLRAEPERFPAAVE

ELLRYFTVVEAATARTATAEVTIGGVTIAAGEGVVALGQAANRDPRVFEH

PDEFDPDRDARAHLAFGYGRHICPGQHLARLEMEVALSRLFRRLPGLRLT

MEVSDLPLKEDSNIFGLYALPVAW

>CYP248A2(2571088695)*Salinispora arenicola* CNS325

VLADAVTAFDPTAVDVRRDPYPSYHWLLRHDPVHRGAHQVWYVSRFADVR

AVLGDERFARTGIRRFWTDLVGPGLLSQIVGDIILFQDEPDHGRLRGVVG

PAFSPSALRRLEPTIEATVNDLLRPARALGAMDVVADLAYPLALRAVLEL

LGLPAGDANTVGRWSRAVGRTLDRGATAEDMRRGHAAIAEFADYVERVLA

ERREDGADLLALMLAAHRSQLMSRNEIVSTVVTFIFTGHETVASQLGNGL

LSLLDHPEQLELVRRQPHLVPQAVEECLRFDPAVQSNTRQLAADVELHGR

RLRRDDVVVVLAGAANRDPGRYDRPDELDIRRDPVPSMSFGAGMRYCLGS

YLARLQLRTALGAMVALPDLRLVCSPNELAYQPRTMFRGLTRLPVAFTPA

G

>CYP107FS2(2571088758)*Salinispora arenicola* CNS325

MPVPQGEQNLTTEVFADPKALFATLGSRQPLHRISLPDGMPAVLVTGNRE

ARQALSDPRLVRSITAAAPELHKYHPLASDDYALSRHMLFADPPDHGRMR

KLVSTAFTRRRVEQMRPRIQQITDDLIDVIAAKGEADLVETLALPLPIAV

ISEMLGVPFADRSEFERHAEVLTGINASSGFDAIIAAGRWFDEYLAELVQ

QRRREPQDDLISGMLAAQDKGDRLTDVELRSNALLLLSAGFETTVNLVAN

GLLALLRHPEAMAALRSEPNLMTTAVDELLRYDSPVSCVTYHFAQEPVEI

GGFEIRSGEHVVIAAAAANHDPTVFADPSRLDLRREGSGQILSFSHGIHF

CLGAPLARLEGEIAFGTVLRRLAGLRLAVPTDSLVWKASFVLHRLERLPV

TFTPDRAPNPIDSVHTV

>CYP105CN1(2571088759)*Salinispora arenicola* CNS325

MAAPAPQATQSTTPHPPSYPLPRECPYRPSAGTARLRDAGPVSTVRLYDG

RTAWLVTGAAEARALLADSRVSNRADFPNYPVMDERHLSMRATREMAREE

EGGFAAALFGMDPPEHTRQRQLLLPRFTVRQVAARRPAIQRIVDEHLDAM

EANGSPADLVSAFATPVPTMVVCTHLGVPYQDRTRFEPAVAGLFEPDRAD

AAMAELTAYLHQLIETKQSEPGDGVIDHLIANHLRPGAIDRAELVAIASA

ILVAGTVTTSSAIALGTLALLTAPGQYAALVDNPDLVPGAVNEILRYLSL

VEQLARVATEDIEIGGKLIRAGDGIIVSFAAGNLDPNVTTHPDRLDVALP

PTNHLAFSHGIHHCIGQNLALLELDIAFRALVSRFPTLRLAVPAEQLPTY

FAGDVPRLACLPVTW

>CYP105CP2(2571088761)*Salinispora arenicola* CNS325

MTKSMPVQDLPAFPIPRECPYRPSAQHVSLRSVGPMAKVRLYNGRTAWLV

TDSAHARAVLSDYRRVSIKPYHGNYPLLNEEFEKVVDSGYADVLFGVDPP

EHTRQRQMIMPSFTLRRTAVLRPDIQRIVDDKLDEMMRHGAPGDLVTEFA

QPVPSMVMSFLLGVPWEDHEEFETPAHKLFVPELAEEATTELGAYLERLI

QKKEQPGGTPGGTGLLDDLIRDHLRAGALSRDELVHIAMAMLVAGTDTTT

NVISLGTLALLDNPDQWAALRDNPDELIPGAVEEILRYTSLIEAFARVAV

SDIELNGAVIKEGEGILISSAGVNFDPALAPDPGRFDIRRPPRPSFSFSH

GIHRCPGDNLARLELEIAFRSLVTRMPNLRTAKPIDQIPSNNNDGTLQRL

YELPVVW

>CYP1005A1(2571088899)*Salinispora arenicola* CNS325

VSAVLFRSWTKTAGTRWPDVTRVADQSGTEHLVVTRHALVRQVLTDQATY

RPDNALEAVTPIPVAALRVLAGHRFRLPPTLANNGGVSHPAIRALVADAL

HPTKVAAQRPWLTGLVADRVASIRTTLDSGGPVDLYADLTADLPLLVLAR

LVELPDAPVNAVKQFARAALELFWAPLDADRQLALADEVGRFHQVLREFA

DTGGGLAAALRATGHSPDVLVGALFFLLVAGQETTSQFLTLLLHRLSGEP

TIRAALRAGSISVADVVEEGLRLEPPIVTWRRVAAVDSTLGGTTVAAGTS

VLLWLARAGRDPAVVAAPDEFRPGQRGSRRHLAFGAGAHRCLGDQLARME

AAVVVEQATPLLDGVTVVRPPWYPDNLTFRMPDAFVVRR

>CYP105BN4(2571089532)*Salinispora arenicola* CNS325

MTATADNPVSTEPFPYTRQGPFAEPPQYPAMVEQKVCPVTLANTGLRTWA

VTGHERVRRILTDPRASASRKHENFPFYFVAPPQARTETSFIGYDAPAHA

QARRKVAAAFTRQRVQTWRPRIEAIVDEHIDRLLSMPPPVDFHRLFSLSL

PTTIICEMLGIPQDDHDMIIEHSTNMFGGHSTPQQRVAAIVEMNAYLDTL

IAQREKEPAAGLISTIIDEYRTSGEDFTRTDVVNMVRMLMNGGHETTASM

LSLGTACLLDNPDELAELLADPENRIEAATEELLRMVTIGDVGVPRVALE

DIEIDGVTIPAGDGILCLLLTANRDPEVFPEPDRLNLSRGSRKHLAFGHG

AHLCIGAELARLEMQVAWLSLFRRIPGLRLATPLQHIPRKEGAIVYGVWN

MPITWDA

>CYP105J3(2571089687)*Salinispora arenicola* CNS325

MTDSVAFPQGRVCPHQPAPGYRPLAVQRPLAQVTLYDGRRVWAVTTRDLA

RRLLVDPRISSDRTNPAWPAIVPIVAAAVNDAQQKVLKIATALVGTDGPE

HKAQRKMLIPSFTFRRMNALRPMIQEIVDQQLDEMIQSGAPTDLIPAFAS

AVPVTVLYRLMGIPDDDHGIFEKLSHQLLAGPNANEAYDQLMGYMSRLIA

ERRRNPGEGVLDDLLAQHGANDDADHDELVSTLVVQVAGNHGTTGSMIAL

GLFALLQHPEQLAELRADPSLMPTAVDELLRFLSVPDAVTRLAADDIEVE

GTIIRQGDGVFFITSLINRDTDVHDAPNSLGWHHASAADHLTFGFGAHQC

LGQSLARITMEIALGALIDRLPSLRLAVPAEEVPFLPAASLQVIAELPIT

W

>CYP107Q4(2571089729)*Salinispora arenicola* CNS325

MTTTAETSAETIDLFSPEVVADPFGWYARLREETGPTTGTLNIGTMMGGP

EMWLVTRYEDVRQVLTDPRFLTNPPADSPLEDIRAGVFKRLDFPPDLIPW

MANLLNVSDGEDHTRLRKLVSYALTAHRIGKLRPRVEKITADLLDKLAED

GKDGSPVDLVEEYCYPLPVTVICELVGIDEPDRPHWRAWGDSMATMNGER

IPTTLVKCIELARELIAKRRAEPQDDLVTALVQAQAEDQNRVSDDEIIGI

LFSLVTAGHQTTTYLIGNSVILLLENPDQLARLKENPSMWPQAVRELQRL

GPIQFAQPRFPSEDIELGGVTIPRGAPVAPLLLAANTDPRRFPDPNKLII

DRLAVGSEGHLGFGKGIHRCLGQHLAYQEAEVALQGLFTRFPDLSLAVPR

EEIPWILRPGFTRTRTLPLKLV

>CYP105G5(2571089730)*Salinispora arenicola* CNS325

LTIETTETPPADDSLRAPLPRQFMQRDDPSKLPPALAALAEQSPVGRSTL

PDGDPFWMVSGYDEARAVLSDPRFSSDRFRYHPRFKKLSGQLGERLRNDK

ARAGSFINMDPPEHTRYRKLLTGQFTVRRMRQLTVRIEQIVTEQVDVMLA

EGNSADLVSAFAVPVPSLMICELLGVRYEDRTEFQRRAAGLLQTDLPIKQ

AVENLEAQRAFMQRLVTDKRRTPADDMISGLVHHAGAEPPLTDDELVGIA

TLLLFAGLDTTASMLGLGMFMLLQRPEQMAVLRDDPSRIGDAVEELLRYL

TVVSTGLFRFAKEDVVLGDEHIPAGSTVVVSLMAANRDGRHWPEPETLDV

TRVRSSHLAFGHGVHQCLGQQLARIELTVGITELLRRLPNVRLAVPPADV

PLRNDMITYGVHRLPILWDTP

>CYP1051A1(2571089754)*Salinispora arenicola* CNS325

MATDAAITRARTVPAWKALPAAVRDTHRALVDVGNWSDGDVVRVSLGVSR

PYLVTNPAHVQEVLHERAAIYPRGDDTALWRSVRKLVGDGILAEGDAWAA

SRRVLAPMFRPARINAMVDTMADAIAGAVDDLHGAATAGTPIDVGRELSR

IVCSAIMRVFFADRITVRDALRIMKAQETIVTAMAPRILAPLVPWWIPMP

GDRRFRAAVRSIDDILLPVLRQAQRQPDDGDDLLSRLVRARADDGRALSE

KRMRDDLVSMVAVTTETSTVVLTWLWPLLANHPDVANRLYDEIDRVVGGG

PVRGDHLAELTYTRMVLDELLRLYPAGWILPRRAATTDVLGGVRINKGAT

VILSPYVTQRMTAWWGPAAEAFDPERFAAGREAADGRHRYAYYPFGVGMH

RCLGEHLFNLEAILIVATLLSRFRFALTDTSMPGVKVAASTRPARTVEMI

LKPVAPVPAR

>CYP105AB8(2571090269)*Salinispora arenicola* CNS325

MTETASSRLTDTEFPVQRECPFAEPVEYEQIREQSSIAMVRLTGGGEAWW

ISGHEQGRAVLADRRFSSDRRKANFPFVSTDPAIRKRLHAQPLSLISMDG

AEHTQARRALIGEFTVRRLAALRPRIQQIVDQCIDEMLTTDQHRADLVKT

LSLPVPSLVICELLGVPYADHDFFQEHTATLVRRNTASEVRQHSIDELNA

YLGALIDRKLASPDDDLLGRQIARQHRDGTFDRSSMVSLAFLLLVAGHET

TANMISLGVVGLLQHPEQLAMIKDDPDKTPLAIEELLRFFTIVDSVTSRV

ATEDVRFGDTTINAGDGVVVSGLSADWDPTVFADPDRLDLERGARHHLAF

GFGPHQCLGQNLARLELQIVFDTLFHRIPTLRLAAPLDKIPFKTDAAIYG

ARELPVAW

>CYP107EU1(2571090877)*Salinispora arenicola* CNS325

VTIGQTLPDLVYSPEFTRDPYAIFARLREQAPVCRVTTHRGMSAWMVTRH

ADVRALLADNRLAKDGNRIGELMPRHSTLTGAATGFPPGLTTNMVNSDPP

DHTRLRHLVGREFTGHRVEGLRPRIEEIVDDLLDGVAACGDEADLAETLA

RRLPIAVIGELLGVPEADRAEFFRWADTLYGGTASPEALGQAYNAIVDYL

GRLCDAKRDVPADDLLTALVQVSADEDRLSREELVSMALLLLVAGHETTS

KQISNGVLALLLNPEQLKLLKAQPARTAGAVEELLRFEGPSLSASLRFTT

EPVEVAGVVIPEGEFVLLSLASGNRDPEKFPDPDRLDITRSTQGNLAMGH

GIHHCVGAALARLELEIVLSRLVARFPQMQLAVEADDLEWLVNSFFRAPL

HLPVSLRR

>CYP1198B1(2571091025)*Salinispora arenicola* CNS325

MSGELTDQRTAPGAGGNPLRSLMDHGIRANPYPLFGELREAGPTAVEDGS

VVLFGEYEHCSQILRHRDMGSDTSEAPSIKGFVVDDAERAGSSIFFMDQP

GHGRQRKLVSKSFTPRIVKSFGPQITHIVDGLFEDFRDKGELDVVTDLAY

PVSIGIICDLFGIPDDERDMLKEWSDDLALSTELPTLGAAIGVLNVFTRD

EINRFGSVAMAAHAYFADLIHRRRKNPGDDLVSSLLATESNGERLTRFEV

TSVLATLFVAAHESTTNLISGGILALLRNQDQMAVLRENPGLITNVVDES

LRYDPPVHLAARMARARTTIGGYDLDPGTIVVVLMAAGNRDPRAYENPDV

FDVNRKIRNVSLAFGAGAHFCIGSGLAKLEAEIAISAFAQRLKHPEVDES

SLEYRRHIVVRGLEHMKVSFQP

>CYP105BL2(2566058131)*Salinispora arenicola* CNS296

MSSHSAAAPDPETATPLHTLAPELTFPQFERSTPFDPPQAYTELSGRCPV

APVSMADGKPSWLITSFEGVRTTLSDPRFSSDMSHPGFPNRTGKPVDDLL

KDTLGAMDGERHRYYRRMLTGELTVRRAKAMRPVITQITDEALDQLAAAG

PGADLVKHVAFVVPSRVACHLVGIPLSDYELFTGMAATLMDSTSSDDQFA

ALQNMVSYFDTLVTDREHHDRDDLLGHMVRRYLATGELTRDMLIRLAWTT

MAAGQETTAHMIGLGVAALLRHPDQLELLRREPHLLPGAVDELMRYLPLI

QFGIPRVAMDDVEVDGQTVTAGEGVVALPPLANRDPAVFERPDELDVRRN

ARQHLTFGYGPHQCPAHALARLELEVVYGRLLERFPTLRLADSDADLKVQ

DKDIMYRVSELAVTW

>CYP105CH1(2566058206)*Salinispora arenicola* CNS296

VSSLPLPTYPKLRDPADPLLPPAEYLAIQSEKPIAKVLLPSGRPTWLITG

HALARQVLTEPCVSVDRRHPNFPYPVPNPDAVVAQVARWTYILLGDDPPL

HTERRRLLISEFTVRQAQAMRPRIQQLVDFHLEQLIAAGPGADFSKHFAM

KVPSAVICEMLGVPFADHDYFQERTALQLRRDVPVAAQKQAIDELLAYFE

QLIQEKSSHPGDDVLSRLIVSNRETEAFDHEALVALGLLLLVGGHETTAN

TLTLATATMLERPEIAEQLRTDPSLMPSAVEEFLRYFSVAVAVSRIATAD

LQVGGQLVRAGESMLLVLNTIARDGTVFPEPHRLDIRRNARNHLAFSHGI

HQCMGQNLARVEMQIALDTVLRRLPGLHLVTPFEELPFKYRHLVWGIEEL

RVAW

>CYP107Q4(2566059072)*Salinispora arenicola* CNS296

MTTTAETSAETIDLFSPEVVADPFGWYARLREETGPTTGTLNIGTMMGGP

EMWLVTRYEDVRQVLTDPRFLTNPPADSPLEDIRAGVFKRLDFPPDLIPW

MANLLNVSDGEDHTRLRKLVSYALTAHRIGKLRPRVEKITADLLDKLAED

GKDGSPVDLVEEYCYPLPVTVICELVGIDEPDRPHWRAWGDSMATMNGER

IPTTLVKCIELARELIAKRRAEPQDDLVTALVQAQAEDQNRVSDDEIIGI

LFSLVTAGHQTTTYLIGNSVILLLENPDQLARLKENPSMWPQAVRELQRL

GPIQFAQPRFPSEDIELGGVTIPRGAPVAPLLLAANTDPRRFPDPNKLII

DRLAVGSEGHLGFGKGIHRCLGQHLAYQEAEVALQGLFTRFPDLSLAVPR

EEIPWILRPGFTRTRTLPLKLV

>CYP105G5(2566059073)*Salinispora arenicola* CNS296

LTIETTETPPADDSLRAPLPRQFMQRDDPSKLPPALAALAEQSPVGRSTL

PDGDPFWMVSGYDEARAVLSDPRFSSDRFRYHPRFKKLSGQLGERLRNDK

ARAGSFINMDPPEHTRYRKLLTGQFTVRRMRQLTVRIEQIVTEQVDVMLA

EGNSADLVSAFAVPVPSLMICELLGVRYEDRTEFQRRAAGLLQTDLPIKQ

AVENLEAQRAFMQRLVTDKRRTPADDMISGLVHHAGAEPPLTDDELVGIA

TLLLFAGLDTTASMLGLGMFMLLQRPEQMAVLRDDPSRIGDAVEELLRYL

TVVSTGLFRFAKEDVVLGDEHIPAGSTVVVSLMAANRDGRHWPEPETLDV

TRVRSSHLAFGHGVHQCLGQQLARIELTVGITELLRRLPNVRLAVPPADV

PLRNDMITYGVHRLPILWDTP

>CYP1051A1(2566059098)*Salinispora arenicola* CNS296

MATDAAITRARTVPAWKALPAAVRDTHRAFVDVGNWSDGDVVRVSLGVSR

PYLVTNPAHVQEVLHERAAIYPRGDDTALWRSVRKLVGDGILAEGDAWAA

SRRVLAPMFRPARINAMVDTMADAIAGAIDDLHGAATAGTPIDVGRELSR

IVCSAIMRVFFADRITVRDALRIMKAQETIVTAMAPRILAPLVPWWIPMP

GDRRFRAAVRSIDDILLPVLRQAQRQPDDGDDLLSRLVRARADDGRALSE

KRMRDDLVSMVAVTTETSTVVLTWLWPLLANHPDVANRLYDEIDRVVGGG

PVRGDHLAELTYTRMVLDELLRLYPAGWILPRRAATTDILGGVRINKGAT

VILSPYVTQRMTAWWGPTAEAFDPERFAAGREAADGRHRYAYYPFGVGMH

RCLGEHLFNLEAILIVATLLSRFRFALTDTSMPGVKVAASTRPARTVEMI

LQPVAPVPAR

>CYP1005A1(2566059348)*Salinispora arenicola* CNS296

VSAVLFRSWTKTAGTRWPDVTRVADQSGTEHLVVTRHALVRQVLTDQATY

RPDNALEAVTPIPVAALRVLAGHRFRLPPTLANNGGVSHPAIRALVADAL

HPTKVAAQRPWLTGLVADRVASIRTTLDSGGPVDLYADLTADLPLLVLAR

LVELPDAPVNAVKQFARAALELFWAPLDADCQLALADEVGRFHQVLREFA

DTGGGLAAALRATGHSPDVLVGALFFLLVAGQETTSQFLTLLLHRLSGEP

TIRAALRAGSSSVADVVEEGLRLEPPIVTWRRVAAVDSTLGGTTVAAGTS

VLLWLARAGRDPAVVAAPDEFRPGQRGSRRHLAFGAGAHRCLGDQLARME

AAVVVEQATPLLDGVTVVRPPWYPDNLTFRMPDAFVVRR

>CYP105CT1(2566059568)*Salinispora arenicola* CNS296

MNSPNHMPADRSLTAPTSGCPMALSRGRVGLDVADEISELRDGGRLGRIT

TAFGQEATLITRYDEVRAQMADSVVFNVAGVPSPPALVDGGFDTESVRRR

RTVGNLIMLDPPEHTRLRRMVAAWFTTRRVERLRPRVVEIIDAALDEMER

SGPPVDLVAMFAKTVPITVICELIGVPEELRERYRRRAERAVSASAVSTP

LDELRRLREAGWVSRELIEYHRENPSDDIIGMLLREHGTDSHDDGITDDE

LVGLANALLIAGHETTTQMLSMGTLALLRHPDQLALLRDDPSIVAGAVEE

LLRYVGVLHGGFVRVATRDTRLGGHRIHAGELVVPALTAANRDPRLLTDG

DRLDITRPPTSHVAFGHGVHFCIGAPLARMELREAFPALLRRFSGLRLAV

PDSELEFTQGTTVYSLRGLPVTW

>CYP154M5(2566059585)*Salinispora arenicola* CNS296

VEQSCPYKLDVTGRDVHAEGEAIRARGPVAQVELPGGVQGWSVTGYQAAR

QVLADPRFAKDPKKWPAYTSGAIPPNWPLIGWLLMDNMTTNDGADHQRLR

KLVSHGFTPRQVERTRPLIVKIVNDLLDGLSSAGPDEVVDLKGRFATPLP

ARVICDMFGVPEALRASVLRGAQVNVTSSISGEEAEANVEQWHRELLELV

EAKREKPDEDMASLLIAAKEEDGSTLTQEEVVGTLHLMLGAGSETLMNAL

SYAVLGMLSNPGQYEMVRNGTSSWDDVIEETLRAQAPVAQLPLRYATEDV

AVGGAVIKAGDPVLMGFTAIGRDPAVHGETAGDYDITREDKTHLSFGHGV

HFCLGAPLARLELKIALPALFERFPNMTLAVRPDQLEPQGTFIMNGHREL

PVRLGQPATVLA

>CYP208A12(2566059954)*Salinispora arenicola* CNS296

MTLDTITPRVPLGPPRTAALRMLLVMKRDRLGMLSSAAARYGDASRLPVG

HKALWFFNHPRYAKHVLADNSANYHKGIGLVHARRALGDGLLTSEGDLWR

KQRKVIQPAFQSRRIAQQAGMIAEEAFALVERLRARAGAGPVELTAELTG

LTLGVLGRSLLDADLAGFDSIGDSFATVQDQAMFELETLNAVPMWIPLPR

QIRFRRARRKLQAVVDTLVDGRAGNLADRVDVLSRLILSARGEADPRVGR

ERLRDELVTLLLAGHETTASTLGWTLSLIDRHPGVWERLHAEAVEVLGDR

LPEYDDLRRLRYTVMVVEEAMRLFPPVWLLPRRALAPDTIGEYRVPANAD

VVISPYTLHRHPEFWPNPERFDPERFAPGQAADRPRYAYLPFGAGPRFCV

GNNLGMMEAVFVIALLCRHLRLTGVPGYRLVPEPMLSLRIRGGLPLVVRP

VS

>CYP125A41(2566060078)*Salinispora arenicola* CNS296

MTEPRIPAGFDFTDPEVLAHRVPREEFAELRRTAPVWWNAQPRGSAGFDD

DGYWVVTRYADVMTVSRDSDTYSTRENTAIARLRPDTTREDIEMQRVIML

NVDPPEHTKLRAIVSRGFTPRAINALRGSLAERAEHIVRDAAVRGVGDFV

SDVACELPLQAIAELIGVPQHHRRKVFDWSNQLIGYDDPAYGTDPLTASA

ELLAYAMEMAEERQRSPSDDLVTKLVNAQIDGEHLTTDEFGFFVMLLAVA

GNETTRNAITHGMVAFLDNPEQWELFKAERPKSAVEEIIRWATPVNVFQR

TALVDTVLGGQAISAGQRVALFYGSANFDEAVFEDPERFDITRSPNPHLG

FGGSGAHFCLGANLARLEIELIFNSIADHLPDIRKVAAPQRLRSGWINGI

RQMPVRYR

>CYP244A4(2566060370)*Salinispora arenicola* CNS296

MSTTTNTELTEAPETNMPVDPGLFDCMPDLIAAARVAPVVRIPYLGRHAW

VVCDRELVKQALTHPKMGKDIALVPEWMRQPGLMVTAQPDPEYARAMIMS

DGENHARIRRIHAPVLSPRNTERWGERVADKVEGFLDELSQAGSGGSTEV

NVVTNYTHKIPLAFISEMLGLPPEAEHRLRGITDIMLYSSDYAARREAIG

GLFGAVEDWVRNPADLRDGVITGLLAASDGPDAAVTEGEVIVWTLGMIIT

GYETTGSLISTSLYEAIRRPPHERPKTDEDITAWIEETLRVHPPFPHPTW

RFPLEDIELGGYLIPKGAPVQVSIAAANRKPGEGADSFDAERRGHGHLSF

GLGMHYCIGAPLVRLEAQIAVRGFLRRFPQARLSAETAVQWESEWMIRRM

SVLPAVLS

>CYP245A7(2566060374)*Salinispora arenicola* CNS296

MPSATLPRFALTGWSRENIVNPYPVYQRYREVASVHRGESGGDAPDTFYV

FSYDEVVQVLSSNCFGRGRSLDAAKASVPVPAEQKALRAIVENWLVFMDP

PRHTELRSLLNRSFSPRIVTELRPRIARIAQELLSRLGQQVDVDLVESFA

APLPILVISELLGIPEERRAWLRANALALQEASSSRAGRDVDGYAQAEVA

AQEFTEYFREQVRLRRGRAGGDLITILANAQERGAPVSLDAIVGTCVHLL

TAGHETTTNSLAKAVLALREHPAVLDELRGAEGLTTDAVEEFLRYDPPVQ

AVTRWAHQDTTLGGCDIPRGSRVVALLGSANRDPARFPSPDVLDVRRPAD

RHLSFGLGIHYCLGATLARAELEIGLQALLDGVPTLGYGTQHVDYADDLV

FHGPSRLVLVNLGERCK

>CYP166A4(2566060868)*Salinispora arenicola* CNS296

MTDAISFELPWARTDKFDPPAVFDALREQRPLARMRYPDGHVGWIVSSYE

LVREVLGDPRFSHSCAVGHFPVTHQGQVIPTHPQIPGMFIHMDPPEHTRY

RRLLTGEFTVRRTSRLTGHVEGVATEQIEVMREHGAPADLVATFARPLVL

RVLSGLVGLPYGERDRYLHAVTLLHDAEADPAEAAAAYEQAGAYFDEVIE

RRRRQPEDDLISTLVGDGELTGEELRNIVTLLLFAGYETTESALAVGMFA

LLHHEDQLARLRADPTKIDAAIEELLRYLTVNQYHTYRTASEDIELHGEV

INKGDSVTVSLPAANRDPARFACPAELDIDRETSGHVAFGFGIHQCLGQN

LARVELRAGLSALLRAFPNLRLAVPADEVPLRLQGSVFAVKNLPVCW

>CYP105CP2(2566060956)*Salinispora arenicola* CNS296

MTKSMPVQDLPAFPIPRECPYRPSAQHVSLRSGGPMAKVRLYNGRTAWLV

TDSAHARAVLSDYRRVSIKPYHGNYPLLNEEFEKVVDSGYADVLFGVDPP

EHTRQRQMIMPSFTLRRTAVLRPDIQRIVDDKLDEMMRHGAPGDLVTEFA

QPVPSMVMSFLLGVPWEDHEEFETPAHKLFVPELAEEATTELGAYLERLI

QKKEQPGGTPGGTGLLDDLIRDHLRAGALSRDELVHIAMAMLVAGTDTTT

NVISLGTLALLDNPDQWAALRDNPDELIPGAVEEILRYTSLIEAFARVAV

SDIELNGAVIKEGEGILISSAGVNFDPALAPDPGRFDIRRPPRPSFSFSH

GIHRCPGDNLARLELEIAFRSLVTRMPNLRTAKPIDQIPSNNNDGTLQRL

YELPVVW

>CYP105CN1(2566060958)*Salinispora arenicola* CNS296

MAAPAPQATQSTTPHPPSYPLPRECPYRPSAGTARLRDAGPVSTVRLYDG

RTAWLVTGAAEARALLADSRVSNRADFPNYPVMDERHLSMRATREMAREE

EGGFAAALFGMDPPEHTRQRQLLLPRFTVRQVAARRPAIQRIVDEHLDAM

EANGSPADLVSAFATPVPTMVVCTHLGVPYQDRTRFEPAVAGLFEPDRAD

AAMAELTAYLHQLIETKQSEPGDGVIDHLIANHLRPDAIDRAELVAIASA

ILVAGTVTTSSAIALGTLALLTAPGQYAALVDNPDLVPGAVNEILRYLSL

VEQLARVATEDIEIGGKLIRAGDGIIVSFAAGNLDPNVTTHPDRLDVALP

PTNHLAFSHGIHHCIGQNLALLELDIAFRALVSRFPTLRLAVPAEQLPTY

FAGDVPRLACLPVTW

>CYP107FS2(2566060959)*Salinispora arenicola* CNS296

MPVPQGEQNLTTEVFADPKALFATLGSRQPLHRISLPDGMPAVLVTGNRE

ARQALSDPRLVRSITAAAPELHKYHPLASDDYALSRHMLFADPPDHGRMR

KLVSTAFTRRRVEQMRPRIQQITDDLIDVIAAKGEADLVETLALPLPIAV

ISEMLGVPFADRSEFERHAEVLTGINASSGFDAIIAAGRWFDEYLAELVQ

QRRREPQDDLISGMLAAQDKGDRLTDVELRSNALLLLSAGFETTVNLVAN

GLLALLRHPEAMAALRSEPNLMTTAVDELLRYDSPVSCVTYHFAQEPVEI

GGFEIRSGEHVVIAAAAANHDPTVFADPSRLDLRREGSGQILSFSHGIHF

CLGAPLARLEGEIAFGTVLRRLAGLRLAVPTDSLVWKASFVLHRLERLPV

TFTPDRAPNPIDSVHTV

>CYP248A2(2566061019)*Salinispora arenicola* CNS296

VLADAVTAFDPTAVDVRRDPYPSYHWLLRHDPVHRGAHQVWYVSRFADVR

AVLGDERFARTGIRRFWTDLVGPGLLSQIVGDIILFQDEPDHGRLRGVVG

PAFSPSALRRLEPTIEATINDLLRPARALGAMDVVADLAYPLALRAVLEL

LGLPAGDANAVGRWSRAVGRTLDRGATAEDMRRGHAAIAEFADYVERALA

ERREDGADLLALMLAAHRSQLMSRNEIVSTVVTFIFTGHETVASQLGNGL

LSLLDHPEQLELMRRQPHLVPQAVEECLRFDPAVQSNTRQLAADVELHGR

RLRRDDVVVVLAGAANRDPGRYDRPDELDIRRDPVPSMSFGAGMRYCLGS

YLARLQLRTALGAMVALPDLRLVCSPNELAYQPRTMFRGLTRLPVAFTPA

G

>CYP105W2(2566061027)*Salinispora arenicola* CNS296

MTGYQDRPTGDQPGAPVPSGSTDPGIGAFPLPRRCPFSPPAEYARLRAEH

PVVRLPMLGGDTAWVVSRHADVRQVLSDPRMSADRRRPGFPKFAPTTEGQ

RQASFANFRPPLNWLDPPEHAICRRQIVDEFSVRRVRQSRALVERVVDTH

LDALTAAAPGADLVSTFAYPVPSQVICEVLGVPYGEHEFFERRSTLMFRR

STPADERARCAREIRDFLDMVVTDKERRPGDDVLSRLLYRQRSAGGMDHE

AVVSMAFVLLVAGHVTTSNMLALSVLALLTHPARLARLRAEPERFPAAVE

ELLRYFTVVEAATARTTTAEVTIGGVTIAAGEGVVALGQAANRDPRVFEH

PDEFDPDRDARAHLAFGHGRHICPGQHLARLEMEVALSRLFRRLPGLRLT

MEVSDLPLKEDSNIFGLYALPVAW

>CYP105AB8(2566061536)*Salinispora arenicola* CNS296

MTETASSRLTDTEFPVQRECPFAEPVEYEQIREQSSIAMVRLTGGGEAWW

ISGHEQGRAVLADRRFSSDRRKANFPFVSTDPAIRKRLHAQPLSLISMDG

AEHTQARRALIGEFTVRRLAALRPRIQQIVDQCIDEMLTTDQHRADLVKT

LSLPVPSLVICELLGVPYADHDFFQEHTATLVRRNTASEVRQHSIDELNA

YLGALIDRKLASPDDDLLGRQIARQHRDGTFDRSSMVSLAFLLLVAGHET

TANMISLGVVGLLQHPEQLAMIKDDPDKTPLAIEELLRFFTIVDSVTSRV

ATEDVRFGDTTINAGDGVVVSGLSADWDPTVFADPDRLDLERGARHHLAF

GFGPHQCLGQNLARLELQIVFDTLFHRIPTLRLAAPLDKIPFKTDAAIYG

ARELPVAW

>CYP211C1(2566061659)*Salinispora arenicola* CNS296

VVDVEELLTRLYSAQGRQDPFPVYADLHAQGPIAALPPEPERRRVAAVAV

GYDLVGAVLRDPEWSKAPPPGWTEQEILRTLQTSMMFINPPDHGRMRHVF

AGTFTPRRLGALEPVVNRVADELLDRMADAGAGGLDFVAEFAYPLPARVM

AEFIGIPETELDWYRERVDVIDAFLDVAGKTPQRLAAANAAGAELRAFYG

ELLARRRRTPGEDLISGLVEAVDAGGVELTEDELVSNLIVLFNASFVTTV

YMLSNGLPVLLAHPEVAAALATDPVLTAGAVDEMLRLQAPVHLLARAAPR

DTVLGGVPIPQGQNVLLLIAAANRDPAHFPDPDRFDPWRSGPPSLAFGLG

LHYCLGAAVSRLEGRLALPRLLSRFPRLRIMEQPVYSGSLFLRGIDKLSV

SPGEGSTRE

>CYP247A7(2566061833)*Salinispora arenicola* CNS296

VRLTPGAARDIDLDSVNLFDLDLYTSGDPHPIWDVMRAQSPLHHQVLADG

REFWSVTRYDDVCRVLGDYREFTSERGTVVTHLGEDDIAAGKLLTSTDPP

RHTQVRRAIGAKLTARAVASWQDRIRDAIVRFLEPALDGDTFDLAEQALL

LPAIVTGPLLGIPERDWQELVQLTAMVTAPSDPHFQHGSEAATLAISHHE

LVTYVTEWVKQRRSAGGGDGSLLDHLMSVRVGGAPLTDEEIALDGYSILL

GANVTTPHTVSGTVLALIERPEQFEKAQADPSLLANLVEEGLRWASAACN

FMRYAVNDTRIGGGTVPAGGAVVAWIGSANRDESYFPDPHQFDITRSGAN

RQVAFGFGPHYCIGAPLARMTLGIFFEELVQRFGSIELAGEPQHLRSYFI

AGMTHLPIVAQKRKTP

>CYP211B2(2566062180)*Salinispora arenicola* CNS296

MDVSEAIAVLISPSGRLDPYPTYEQLRAHGPVSRTTAGLFVVTGYAEADM

VLRDPRFVVLDDDLRDDVFPHWQDSPAIKSIARSMLRTNPPDHSRIRRLA

AGAFTPRRVAAMREVVTAQADELVDEMIRAGRDGARVDFMDMFAYPLPVA

VICALLGVPAADRSRFRRWAGDLTGILEPEITPEELAGADAGADELRDYF

TGLIEQRRRAPADDLTTALVQAHDADGDRLSGEELLANLVVLLVAGFETT

TNLLGNGLVVLLTRPEAAAALRDEPDLAPGYVDELLRYDSPVQLTTRTVR

ESVSFAGTELPADSWLLVLLGAANRDPRRFPDPARFDPGRAQSQPLSFGA

GPHYCLGAGLARLEAQVAFPLLLRRLPELALAGRPSRRTRLTLRGYETLP

ITVGAVTADRGTPAGVAPGTP

>CYP107EU1(2566062569)*Salinispora arenicola* CNS296

VTIGQTLPDLVYSPEFTRDPYAIFARLREQAPVCRVTTHRGMSAWMVTRH

ADVRALLADNRLAKDGNRIGELMPRHSTLTGAATGFPPGLTTNMVNSDPP

DHTRLRHLVGREFTGHRVEGLRPRIEEIVDDLLDGVAACGDEADLAETLA

RRLPIAVIGELLGVPEADRAEFFRWADTLYGGTASPEALGQAYNAIVDYL

GRLCDAKRDVPADDLLTALVQVSADEDRLSREELVSMALLLLVAGHETTS

KQISNGVLALLLNPEQLKLLKAQPARTAGAVEELLRFEGPSLSASLRFTT

EPVEVAGVVIPEGEFVLLSLASGNRDPEKFPDPDRLDITRSTQGNLAMGH

GIHHCVGAALARLELEIVLSRLVARFPQMQLAVEADDLEWLVNSFFRAPL

HLPVSLRR

>CYP105J3(2566062867)*Salinispora arenicola* CNS296

MTDSVAFPQGRVCPHQPAPGYRPLAVQRPLAQLTLYDGRRVWAVTTRDLA

RRLLVDPRISSDRTNPAWPAIVPIVAAAVNDAQQKVLKIATALVGTDGPE

HKAQRKMLIPSFTFRRMNALRPMIQEIVDQQLDEMIKSGAPTDLIPAFAS

AVPVTVLYRLMGIPDDDHGFFEKLSHQLLAGPNANEAYDQLMGYMSRLIA

ERRRNPGEGVLDDLLAQHGANDDADHDELVSTLVVQVAGNHGTTGSMIAL

GLFALLQHPEQLAELRADPSLMPTAVDELLRFLSVPDAVTRSAADDIEVE

GTIIRKGDGVFFITSLINRDTDVHDAPNSLGWHHASAADHLTFGFGAHQC

LGQSLARITMEIALGALIDRLPSLRLAVPAEEVPFLPAASLQVIAELPIT

W

>CYP1198B1(2566062920)*Salinispora arenicola* CNS296

MSGELTDQRTAPGAGGNPLRSLMDHGIRANPYPLFGELREAGPTAVEDGS

VVLFGEYEHCSQILRHRDMGSDTSEAPSIKGFVVDDAERAGSSIFFMDQP

GHGRQRKLVSKSFTPRIVKSFGPQITHIVDGLFEDFRDKGELDVVTDLAY

PVSIGIICDLFGIPDDERDMLKEWSDDLALSTELPTLGAAIGVLNVFTRD

EINRFGSVAMAAHAYFADLIHRRRKNPGDDLVSSLLATESNGERLTRFEV

TSVLATLFVAAHESTTNLISGGILALLRNQDQMAVLRENPGLITNVVDES

LRYDPPVHLAARMARARTTIGGYDLDPGTIVVVLMAAGNRDPRAYENPDV

FDVNRKIRNVSLAFGAGAHFCIGSGLAKLEAEIAISAFAQRLKHPEVDES

SLEYRRHIVVRGLEHMKVSFQP

>CYP105AH4(2517455912)*Salinispora pacifica* DSM 45549

MTLLQAEEKVLTWPFARTEWGTPPPILAQLRQAPPCAVIIPAGAASERRA

WLVTRYPDVRQALMDPRLSADELLPGAPVRIQVPQEERPSSFLRMDDPEH

GRLRGMIASQFTARRVRQLVPAVQRRTDDLLDAFVAGPCPADLHDAFSRK

LPTLVIASLLGVPDEDSAFFVEKTRVTLSQDDPEVSFAAYREMTDYLGQL

AARKAKEPQDDLMSQLAVNNLAVGDITMDELVGIARLVLVAGHETTTNQI

ALNILSLLLDPGLRAQVLADDGRLLPEYIEEAMRYWSISQDGIVRLVTED

LVLGEVQMRAGDAVVISIPAANHDPTVFPEPNRIDVTRSPRDHLQWGNGP

HYCQGAPLARLEMVIALRTLFARLPGLRLATDDVPSLFRRGTVFHGVENL

PVAW

>CYP245A13(2517456708)*Salinispora pacifica* DSM 45549

MPSATLPRFTLTGWAREDIINPYPVYQRYREAAAVHRGEASGDAPETFYV

FSYDEVAQVLSSNCFGRGRSLDAAAAAVPVPTDQKALRAIVENWLVFMDP

PRHTELRALLNRSFSPRIVTGLRPRIARIAQELLTRLGQQVETDLVAGFA

APLPILVIAELLGIPAERHGWLRANAMALQEASSSRARRDVDGYARAESA

AQEFTEYFREQVYLRRGSTGDDLMTILANAQLRGAPVGLEAVVGTCVHLL

TAGHETTTNSLAKAVLALRAHPAVLEELRSADGLTADSIAEFLRYDPPVQ

AVTRWTHQDTTLGDWEVPRGSRVVALLGSANRDPARFPLPDALDVRRTAD

RHLGFGLGIHYCLGATLARAELEIGLQTLLDGLPALDYSVQYVDYADDMV

FHGPSRLLLVNPGERFCS

>CYP244A5(2517456712)*Salinispora pacifica* DSM 45549

MSVTTNAELGEAPETNMPVDPGLFDCLPDLIAAARIAPVVRIPYLGRHAW

VVCDRELVRQALTHPKMGKDITLVPEWMRQPGLMVTAQPPPEYARAMIMS

DGENHARIRRIHAPVLSPRNTERWGEQVADKVEGFLDELGQAASGGNAEV

DVVTNYTHKIPLAFISEMLGLPPEAEHRLRSITDIMLYSSDYAARREAIG

GLFGAVEEWVRDPAGLRDGVITGLLAASDGPGAAVTEGEVIVWTLGMIIT

GYETTGSLISTSLYEALRRPPHERPKTDEDIAAWIEETLRVHPPFPHPTW

RFPLEDIELGGYLIPKGAPVQVSIAAANRKPDEGADSFDAERRGHGHLSF

GLGMHYCIGAPLVRLEAKIAVRGFLRRFPRARLSADTAVQWESEWMIRRM

SLLPAVLS

>CYP113E2(2517456885)*Salinispora pacifica* DSM 45549

MAAVPRLTFADGGKRVLPWFRRMRDDQPVWYDQGTKSWNVFRYADIAQIL

KDPVTFSSDPGRSMPPELAEEAEGSLVAVDPPRHTRLRGLISAAFTPRLV

EQLAPRVRSIGELLLNRAFVDRRVEGEFDIIGDLAYLLPVYVIGELLGLP

ESDRGYLVRAADEFYAISADDPFDGAYMASMQSTLDELGSYMLDHAERRR

AKPGDDLISALAHAEIDGERLNDREIRNFAILLLTAGHITTTALLGNTLL

ALGERQDIMLRWRQGQVDTAILLEEVLRHRTPFTEVYRFTTTEVTIGNQV

VPGDQLLRLWIASGNRDERQFADPDTFVLGRDSKHLGFGLGIHYCLGASL

ARMESSVVLGLLAERTTSLAPAVEALSYYDAPGIFCLRSLPVSYRRT

>CYP211B18(2517457068)*Salinispora pacifica* DSM 45549

MDASEAVALLMSPPGRLDPYPTYEQLRAHGPVVPTAAGFFVVTGYTEADT

VLRNPRFGVMDDEERADVFPHWQDSPAMMSISRSMIRANPPDHSRMRRLA

AGVFTPRRVAALREVVAAQADELIDEMTRAGRGGAPVDFMGSFAYPLPVT

VICALLGVPAADWAQFRRWASELTGVLEPEITPEELAVADAGASGLRDYF

TELIAERRRTPAEDLTTALVQAHDADGDRLSGEELLANLVLLLVAGFETT

TNLLGNGLVVLLNQPGSATALRDQPELAPRYVDELLRYDSPVQLTTRTVR

ESMPLAGVELPAGSWLLVLLGAANRDPARFTDPARFDPGRAQSPPLSFGA

GAHYCLGAGLARLEAQVAFPLLLRRLPELALAGEPTRRNRLTLRGYETLP

VTVGAAPADHGTPAGAALGTP

>CYP1005A6(2517457395)*Salinispora pacifica* DSM 45549

MSAVLFRSWTKTAGTHWPAVTRVADQQGTEHLVVTEHALVRQVLTDQVTY

RPDNALDAVTPIPVAALRVLTGHRFRLPPTLANNGGVSHPAIRALVADAL

HPAKVAAQRPWLTGLVAERVAAIRATLDSGGSADLHAELSADLPLLVLAR

LVELPDAPVSAVKQFARAALELFWAPLDAGRQLALADEVGRFHQVLREFA

DTGGGLAAALRATGHPPDVLVGALFFLLVAGQETTSQFLTLLLHRLAGEP

TVRAALRDGSVSVTNVVEEGLRLEPPIVTWRRVAAVDSTLGGTAVPAGTS

VLLWLARAGRDPAIVPAPDEFRPGQRGSRRHLAFGAGAHRCLGDQLARME

AAVVVEQVTPLLDGITVVRAPWYPDNLTFRMPDAFVIRR

>CYP105AB24(2517457638)*Salinispora pacifica* DSM 45549

MPETASITTTGTASTATTGAASGKVTDAEFPVARGCPFSTPAEYEQIREH

SPIAKVRLTTGREAWWIAGHELGRTVLADRRFSSDRRRDNFPFVSTDPET

RKQLQDQPTSMLGMDGAEHAQTRRALMGEFTVRRMAGLRPRIQQIVDQHI

DEMLSSEQRSADLVEALSLPVPSLVICELLGVPYADHDFFQGLTGPLLRH

TTPPEARGRIQEELNTYLGTLIDRKLTDPTDDLLGRQIAKQHAAGTFDRT

SLVSMAFLLLIAGHETTANMISLGVVGLLQHPDQLAMIKNDPEKTPPAVE

ELLRYFTIADTVTARVATEDVQLGDTTINAGDGVVISGLAADHDPTVFTD

PDRIDLERGARHHVAFGFGPHQCIGQTLARMELQIVFDTLFRRIPTLRLA

APLDDIPFKSDAFVYGAEKLPVAW

>CYP107NH1(2517458341)*Salinispora pacifica* DSM 45549

MTEPVVSLMDPEFWNDPVSAYERLRGSGPLIRMGLPGVPPVWLVTSCEHV

KSALSDPRFVVDAANVPGHHGPGIVDQMMAASGMPDEFRDYMTNMMFTDG

KDHSRLRRLVTPGFSARRIRAMRPRVDQIAGELVESLAEKGSGELIADFS

APLTTTVICELIGVDRADQAQMGAWMRDYTTGERVVSGRAMVNYTRDLIE

RRRAEPADDMISAMIRSGDEAGDRLSDAEIIAMALLLINAGHHSTAQFIP

NAVLVLLDHPEQLARLRAEPGQLPGAMDELMRLANPVPIATPRYATEDME

FAGVAVRRGEALTGSLEAANFDPERFPAPRQLDTGRDLGRGDGHLSFGAG

PHYCPGAALARLEGEIALDHLLLRRDSLRLAVERDEVDYVDVSLGLRMLS

SLPVRL

>CYP247A8(2517458347)*Salinispora pacifica* DSM 45549

MRLTPEAGRPVALGSIDLFDPDLLASGDPHSVWDVMRSEAPLHRQVLPDG

RVFWSVTRYEDVCRVLGDHRAFTSERGTVFTQLGADDIAAGKLLTSTDPP

RHTEVRRAIGGRLTARAVAHWQDLVRRAVVRFLEPALDGGCWDLAERAQQ

LPLIIAGSLLGIPESDWEKLVQLTGMVTAPSDPMFGLGSEAATLAIAHHE

IFDYLSDLVRRRRSAGTGGDSLLDHLMTVRAGAGPLPDEEVVYDSYSLIL

GANATTPHTLSGIVLTLVEWPEQCDKVRADPSLIPSMVEEGLRWTSAACN

FMRYATVDTRLTGGTVPAGEAVVAWIASANRDESQFTDPHTFDVTRGENR

HVAFGFGPHYCIGAPLARMTARIFFEELFRLFWSIEIDGEPQHLRSNFIA

GMTHLPVVTRKRAQV

>CYP107Z27(2517458357)*Salinispora pacifica* DSM 45549

MSRTLPCHSLMDPTLIDDPAGGFSRIRDESPLAHAMVPGVDGPVWLVTRH

ESVRKVLGDRRFVNDPTNVPGSSTPDLWAHAALAQGVPHEYLEHVRSMLQ

LDGEVHARLRRLVSRAFTMRRITALRPRMMQLTEELLDLLPAKAEDGVVD

LVEHFNYVQPISVICALVGVPAADRAAWIRWSRVLTSMESGTIGDAVTGM

VDNIRALIEQRRAEAADDLLTDLVRVRDADGDRLSEREMITLVITLVTAG

HDSTGLLLSNGLAALLTHPDQLAKLRADPSLGPQAFDELMRWCSAIVAAR

PRYATEDVELEDGLVRRGDAVIPVLVSANYDPEVFEDPHRLDITRVHEQR

RFHHVGFGDGLHYCLGAALAKHEAAIAITALLERYPGLELAVPAQRLRRA

RLPLTWRLDSLPVRLGIGG

>CYP107AY6(2517459214)*Salinispora pacifica* DSM 45549

MRAELAPIPRSGVRLGQEYDEFRKTGDVHQVLLPDSSLAWLVTDPELVSR

ALADPRLALNRRHSRGGWSGFALPPALDANLLNLDAPDHTRLRRLVGPAF

SPQRVAALRPRIRRTAEHLLDALVTAGGPVDLVTGYCTPLSVQVIADLMG

VPEGGRTDLRTWTDTMLTSYPPDRAAIRRTVVELHGYVVNLIAAKQQQPG

DDLLSALVTIEQDGDRLTRDELTSLAFLILFAGYENTANLIASTVLRLLD

RGGIREARILEAIEETLRLEPPAPVAVRRFPTEEMAIGGATIPAGDTVLL

SIAAATRGVDGSATRLAFGNGPHFCLGAALARVEAEEALTVLGRRLPGLA

LAVPVAQVRWRPTFRTHGPAELLVTW

> CYP244A9(2517459227)*Salinispora pacifica* DSM 45549

MQNTAHTNLAEAPEVTMPIELRPTDCLPELLAAARVAPVVRIPYLDQHAW

VVCDRELVKQALTHPKLGKDVTLAPDWMRQPGQMVTAIPPPEYARMMVMS

DGEHHARIRRIHAPVLSPRNAERWSERVAALVDGFLDNLDSTAGAQVDLI

TDYTHKIPLAFTSEMLGLPPGAERRLHDITEVMLYSADYALRQQAVGELF

EAVQEWVRNPAGLREGVITGLLASSDGPDATVTEGEAIVWTLSLIINGYE

TTGSLISAALYEALRRPARERPHTDEAIAAWIEETLRVQPPVPHTTWRFA

LADLDLGGYLIPKGAPVQISLAAANLDPDEDADSFDARRRGRGHLSFGRG

AHYCIGAPLARVQTKIALRGFLRRFPQARLSADTAPQWESEWMIRRMSVL

PVLLA

>CYP107NG1(2517459604)*Salinispora pacifica* DSM 45549

MTIPAGHSAAVCDFPVVQPDRLAVEPLFADLRSRTPIVRVRLPFGGTAWL

LTRYRDIRAVLASAQCCRAATTDPDTPRILPRAGGEGLLMSLDAPEHTRL

RGLVTAWFTTRRVESLRPATEQAARQLIADMRGTGRADLVEQFSQKLSAT

VIGDLLGVPRSDRETFQRWSEALLSSTSYTQTQVQQATAELNNYFGYLID

QRDSHPSDDLVGTLVRNMQAGKLSRREVLALVTDLLVAGFETTAGQLTNS

VYTLSTIPGAWAWLAADRTRIPAAVEELLRALPLGAGGFRARVTTAPITL

GGDTDRPTTIPAGHVIIAPTIAANTDPEAFDEPLTIRLDRPRNQHLAFGH

GAHRCLGAPLARMELTTALDELIEAFPSLTLAAPETDLQWKSGLQIRGPR

ALPVTW

>CYP154M18(2517459672)*Salinispora pacifica* DSM 45549

MHETCPVLDTAGRDIHGESDRLRQRGAAVKVKLPGGVLAWAVTGHSAIKK

VLLDPNVTKNARAHWPAFINGEIPMDWELISWVAMDNMVTAYGAHLLQLR

KLSAKAFTPHRVEMMRPRVVALVNQLLDDLTAGGDEVVDLRERFCYPLPA

LTVADLIGMSEEHRQQTAKAMDMMVDTTVTPAQAQETLAGWRGAMAELIA

EKRRNPGEDIASDLIAARDDENGAKLTDTELTDTIFAILGAGSETTINFI

DNAVTALLSHPDQLAMVRSGQISWAEVIEETLRVECPLGSLPLRYAVTDI

ELEGVTIPKGDPILVNYAAAGRDPALHGETAGQFDVTRPDKEHISFGRGP

HYCLGAGIARMVATVGLSTLFERFPDLALAVPKGELQPLPTFIMNGHRAL

PVRLGVHRRAAVAG

>CYP208A28(2517459698)*Salinispora pacifica* DSM 45549

MTVIADRGGRVPPGPPVTAGLRLLLALGRDRLGMMTSAASEYGDVARLPV

GPKRLYFFNHPDHAKHVLADNNANYQKGIGLVHARRALGDGLLTSDGELW

RKQRRVIQPAFLNRRLAQYAGVVGQEATRLAERLATRVDGPPVDVLDEMT

RLTLGVLGRTLLDAELTGFRGVGESFAAVQDQAMFELETLSAVPTWIPLR

RQRRFRRARRHLQEVVDVLAAERGQEVEGRDDVLSRLILSTRAEADPQLG

RQRLRDELVTLLLAGHETTASTLGWSLHLLDQHPRLRERVRHEATSVLGG

RVPVYEDLHQLRYTAMVVQETIRLYPPVWILTRRARADDEVGGYRVPAGA

DLLICPYTLHRHPRFWEEPERFDPERFDPAGLTNRPRYAYIPFGAGPRFC

VGTNLGMLEATLVLAVLLRDLRLEGLPGRAVTPEPMLSLRVRGGLPMTVR

RAD

>CYP125A15(2517459788)*Salinispora pacifica* DSM 45549

MTEPRIPVGFDFTDPAVLERRVPREEFAELRRTAPVWWNVQPRGSAGFDD

DGYWVVTRYADVMAVSRDSETYSTRENTAIARFQPGTTRADLEMQRVIML

NVDPPEHTKLRAIVSRGFTPRAINALRGSLAERAERIVRDAAVRGTGDFV

TDVACELPLQAIAELIGVPQHHRRKVFDWSNQLIGYDDPTYGVDPLTAAA

ELLAYAMEMADERQRNPSDDLVTKLVNAQIDGEHLTTDEFGFFVMLLAVA

GNETTRNAITHGMLAFLEHPEQWELFKAERPRSAVEEIIRWATPVNVFQR

TALVDTTLGGQAISAGQRVALFYGSANFDESVFEEPDRFDITRSPNPHLG

FGGSGAHFCLGANLARLEIELIFNSIADHMPDIRKAAEPQRLRSGWINGI

REMPVRYR

>CYP211C8(2517459912)*Salinispora pacifica* DSM 45549

MLDVEGLLARLYSAQGRQEPYPVYADLHAQGAIAALASRPERQRVAAVAV

GYDLVASVLRDPEWFKQPPPDWTEQEILRTLQSSMMFINPPDHGRMRHVF

AGTFTPRRLGALEPVISRVTDELLDRMADAGPGEVDFVAEFAYPLPARVM

AEFIGIPAAELAWYRERVDRIDAFLDVAGKTPERLAAANVAGAELRAFYG

ELLAHRRRTPGEDLISGLVQAVDSGGVELTEDELISNLIVLFNASFVTTV

YLLSNGLPVLLAHPEVAAAMADDPELAAGAVDEILRLQTPVHLLARAAPR

DTVLGGVSIPQGQSVLLLIAAANRDPAHFPDPDRFDPRRSGPPSLAFGLG

LHYCLGAAVSRLEGRLALPRLLSRFPRLRILEQPVYSGSLFLRGIDKLSV

SPGGREHP

>CYP105AB28(2517984974)*Salinispora pacifica* CNT851

MTETASTTTPGISSTTTSGPASGEVTDAEFPLERGCPFSTPTEYEQIREH

SPLAKVRLTTGREAWWIAGHELGRAVLADRRFSSDRRRDNFPFVSTDPET

RKQLQDQPTSMIGMDGAEHAQARRALMGEFTVRRMAGLRPRIQQIVDQHI

DEMLSSDQRTADLVEALSLPVPSLVICELLGVPYADHDFFQARSGPLIRH

TTPTEVRLRIQKELNTYLGALIDRKVTDPTDDLLSRQIAKHHAAGTFDRT

SLVSMAFLLLIAGHETTANMISLGVVGLLQHPDQLAMIKEDPEKTAPAVE

ELLRYFTIADTVTARVATEDVQLGGTTINAGDGVVISGLAADHDPTVFTD

PDRLDLERGARHHVAFGFGPHQCIGQTLARMELQIVFDTLFRRIPTLRLA

APLDDIPFKSDAFVYGAERLPVAW

>CYP107FH3(2517985263)*Salinispora pacifica* CNT851

VPIELDEAFVQDPYAVYEKLRAEGPAHRVRMPPGVPLIGGLPVWLITGYD

AVRAALADSRLSTDLRRIDGLFAQKDPDRSHRGGFSSALASHMMHTDPPD

HTRLRKLVSKAFTRRAIEALRPRIQQTTDELTAEMAGHDTVDLLDAFAFP

LPIRVICLLLGVPVAEQENFRSWSRALVSGHSPEAAATAATEVAAYLGDL

VERKRHATTDDVLTALVAAHDVDDRLTHTELVSTAYLLFIAGFETTLNAL

GNGTLHLMLHRDQWTALRADRALLDNAVEEFLRLESPLKHATFRCATESL

RIGDAEIAAGDFVLLAIASANRDPRRFPDPHTLDVRRPAAGHLAFGHGIH

HCLGAPLARVEVRMAFDALLDAFPDMRLATDPAGLRWRNSTIIRGLDSLP

VHLNN

>CYP2054A3(2517985267)*Salinispora pacifica* CNT851

MSISADSEQVQGRPFDPYGAHRDDPYTFLAGLGVFYAPLLDAWCVTRRED

MVAVLRDDRSFSARDHNPRPAVALPDDVNQMFRTWRGAGAVAVGSLDPPA

HAKIRDVLNLGFTPARVRAFEPTMRAVATDLADRIGDAPEFDFIADFAVP

FALEVIGRRLGVPDDYLDRCRTWSEQRIELMMAQGDADHDRLREFARGLM

EFGEFARSLVRDRVADPRDDLISELLHDGKAGRTLTADEVAVQIPTLIFA

GHMTCAEALGTIFYQQLRSPGGWARVVDRTIPVGDLVEEGLRFDSPLAGM

YRTATRDVTVGGIRLTAGSRLLLLYGAAGRDSRAHACPAAFRPGDGSSGH

LAFGHGIHFCLGAGFARAELRVAVEVLAARMPDLGLAPGRPPRFRPVFPL

RALTELRVTRSGGGSGP

>CYP161N4(2517985273)*Salinispora pacifica* CNT851

VNTVAQLPFTQTHVLDVAPALRLLQSRGKVHRVRTPEGVPAWLVTGHAEV

QQLLDDDRLSRSDPGGRDGGTALLNKLLGPLADDHPRLRSLLEPQFTPER

LKPLRAVVEKLTEQHLDELATRTPPVDLRPALAMSLPILVLCEWLGVPAE

DKGRFSVWTQDAAGVQDPERSQRGLAELFGYCRQLVAAKRQDPGDDVISR

LIATAGIGDTEAVALTALLLFGGYETTVARIGTGVLLLLTNPDQWAAVRA

DPALVPATVDELLRRSMPNPHNGGMPRFAVTGFEIDGAAIRAGDLVLLNI

IAANHDETAFPDPDRLDITRPTAGSLAFGHGRHSCVGAPLARMVLRVVLS

RLITRLPDLRLAVGVDELKLRHETLVGGLVELPVTWGPR

>CYP107AW7(2517985556)*Salinispora pacifica* CNT851

VETVTGTSTPPPVPYIADPYPTLARIRANGPVSILHSDEGIPMWVIARYR

DVRAALADPRFGQDARRAQALADNRVAGVALGGDIVHMLNSDPPDHTRLR

RHVQRAFTARRVAAMRPLVERITTSLLDGLTGRTTVDLVQDFAFPLPMLV

ICELLGFPAEERNAYRSWSTAILTHDDDPAAFATALREMTDYIAVQLRIR

QTRPGDDILTELLAARDAGQLTDDEIIGMVFLLLIGGHETTVNLLGTATL

ALMRNPDQHQWLLANQHALPEAIDEFLRYESPVAMATLRFTTTPVAVDDV

VIPAGELVLVSLGGANRDPDRFPDADRLILDRRDTGHLAFGHGLHRCLGA

FLGKLEGEVALGALLRRHPRLALAAEVRQLRWRDTIMLRGLESLPVSLHG

>CYP211B11(2517985899)*Salinispora pacifica* CNT851

MDASEAVALLTSPPGRLDPYPTYERLRAHGPVVSTAPGFFVVTGYTEADT

VLRNPRFEVMEDEERDGVFPHWQDSPAMISISRSMIRANPPDHSRMRRLA

AGVFTPRRVAALREVVAAQADGLVDEMIRAGRGGAAVDFMGSFAYPLPVT

VICALLGVPTADWARFRHWASDLTGVLEPEITPQELAIADAGASELRDYF

TELIAQRRRAPADDLTTALVQAHDADGDRLSGEELLANLVLLLVAGFETT

TNLLGNGLVVLLTHPDSATALRGQPELAPGYVEEFLRYDSPVQLTSRTVR

ESVSLAGVELPAGSWLLVLLGAANRDPARFTDPARFDPRRAQSPPLSFGA

GAHYCLGAGLARLEAQVAFPLLLRRLPELALAGEPTRRHRLTLRGYETLP

VTVGAVPADPGTPAGVALGTP

>CYP125A65(2517986732)*Salinispora pacifica* CNT851

MTEPRIPVGFDFTDPAVLEHRVPREEFAELRRTAPVWWNVQPRGSAGFDD

DGYWVVTRYADVMAVSRDSETYSTRENTAIARFQPGTTQADREMQRVIMI

NVDPPEHTKLRAIVSRGFTPRAINALRGSLAERAERIVRDAAVRGTGDFV

TDVACELPLQAIAELIGVPQHHRRKVFDWSNQLIGYDDPAYGVDPMAAAA

ELLAYAMEMANERQRNPSDDLVTKLVNAQIDGEHLTTDEFGFFVMLLAVA

GNETTRNAITHGMLAFLEHPDQWELFKAERPRSAVEEIIRWATPVNVFQR

TALVDTTLGGQAITAGQRVALFYGSANFDESVFEEPERFDITRSPNPHLG

FGGSGVHFCLGANLARLEIELIFNSIADHMPDIRKVADPQRLRSGWINGI

REMPVRYR

>CYP208A22(2517987587)*Salinispora pacifica* CNT851

MTVIADRGGRIPPGPPVTAGLRLLLALGRDRLGMMTSAAAEYGDVARLPV

GPKKLYFFNHPDHAKHVLADNHANYQKGIGLVHARRALGDGLLTSEGELW

RKQRRVIQPAFQNRRLAQYAGAVGQEATRLVARLATRVDGPPVDVLDEMT

RLTLGVLGRTLLDAELTGFHGVGESFAAVQDQAMFELETLNTVPTWIPLR

RQRRFRRARQHLQEVVDVLAAERGQAVEGRDDVLSRLILSTRAEADPQLG

RERLRDELVTLLLAGHETTASTLGWSLHLLDQHPELRERVRHEARTVLGD

RVPAYEDLHQLRYTAMVVEEAIRLYPPVWILTRKARAEDEIGGYRVPAGA

DILICPYTLHRHPRFWAEPERFDPERFDPSRTTDRPRYAYIPFGAGPRFC

VGNNLGMLEATLVLAVLLRDLRLEGLPGRAVVPEPMLSLRVRGGLPMTVR

RVD

>CYP244A13(2517988563)*Salinispora pacifica* CNT851

MQDTAQAKLAEAPEVTMPIELRPTDCLPELLAAARVAPVVRTPYLDQHAW

VVCDRELVKQALTHPKLGKDVALAPDWMRQPGQMVTAMPPPEYARMMVMS

DGEHHARIRRIHAPVLSPRNTERWSERVAALIDGFLDNLDSADGTEVNLI

TDYTHKIPLAFTAEMLGLPPGAERRLHDITEVTLYSADYALRQQAVGELF

EAVQEWVRDPAGLRDGVVTGLLACTDGPDATVTESEAIVWTLSLIINGYE

TTGSLISAALYEALRRPARERPHTDEAVAAWIEETLRVQPPVPNTTWRFA

LADLDLGGYLIPRGAPVQISLAAANLNPDEDADSFDAQRRGRGHLSFGLG

AHYCIGAPLARVQTKIALRGFLRRFPQARLSPDTTPRWESEWMIRRMSVL

PVLLA

>CYP107AY9(2517988584)*Salinispora pacifica* CNT851

MSQDQPTRAELAPIPRSGARIGPEYDQLRKTGDVHQVLLPDTSMAWLVTS

PELVSRALADPRLALNRKHSRGGWSGFALPPALDANLLNLDAPDHTRLRR

LVGPAFSPQRVAALRPRIQRTAEELAETVVATGSPVDLVTGYCTPLSVQV

IADLLGVPEAGRTDLRAWTDTMLTSYPPDRDAIRQAVVELHGYVVNLIEA

KQQRPGDDLLSALVATEQEGDRLTRDELTSLAFLILFAGYENTANLIAST

VLRLLDHGSLRGVRASEAIEETLRLEPPAPAAIRRFPTEEMTIGGATIPA

GDTVLLSIAAATRGADGNPARLAFGNGPHYCLGAALARAEAEEAITVLAR

RLPGLALVVPGAPVRWRPTFRTHGPAELLVAW

>CYP1005A6(2517988959)*Salinispora pacifica* CNT851

VSVVLFRSWTKTAGTHWPAVTRVADQQGTEHLVVTEHALVRQVLTDQVTY

RPDNALDAVTPIPVAALRVLAGHRFRLPPTLANNGGVSHPAIRALVADAL

HPAKVAAQRPWLTELVAERVAAIRATLDSGGSADLHAELSADLPLLVLAR

LVELPDAPVAAVKQFARAALELFWAPLDADRQLALADEVGRFHQVLREFA

DTGGGLAAALRATGHPPDVLVGALFFLLVAGQETTSQFLTLLLHRLAGEP

TVRAALRDGSVSVTNVVEEGLRLEPPIVTWRRVAAVDSTLGGTAVPAGTS

VLLWLARAGRDPAIVSAPDEFRPGQRGSRRHLAFGAGAHRCLGDQLARME

AAVVVERVSPLLDGVAVVRAPWYPDNLTFRMPDAFVIRRGPAGSAER

>CYP107NG1(2517989055)*Salinispora pacifica* CNT851

VTIPAGHSPAVCDFPVVQPDRLAVEPLFADLRSRTPIVRVRLPFGGTAWL

LTRYRDIRAVLASAQCCRAATTDPDTPRILPRAGGEGLLMSLDAPEHTRL

RGLVTAWFTTRRVESLRPATEQAARQLIADMRGTGRADLVEQFSQKLSAT

VIGDLLGVPRSDRETFQRWSEALLSSTSYTQTQVQQATAELNNYFGYLID

QRDSHPSDDLVGTLVRNMKAGKLSRREVLALVTDLLVAGFETTAGQLTNS

VYTLSTTPGAWAWLAADRTRIPAAVEELLRALPLGAGGFRARVTTAPLTL

GGDTDRPTTIPAGHVIIAPTIAANTDPEAFDEPLTIRLDRPRNQHLAFGH

GAHRCLGAPLARMELTTALGELIEAFPSLTLVAPETDLQWKSGLQIRGPR

ALPVTW

>CYP107E37(2517989167)*Salinispora pacifica* CNT851

VTIDQEIREYPFRESSGIGIDPTYELLRRTEPLARVQLPYGEVSWLVTRY

EDVKTVLTDPRFSRAAAQGKDQPRTRAEMTYEGIIGLDPPDHTRLRRLAG

KALTARRVNAIRADAQRIAHEYVDEMIAKGSPGDLVELVALPYPVTVICE

LLGVPFEDRAQFRIWTEGLTSTSEQLMAYAEQLFDYMGKLVAQRRAEPTD

DLLGALVKARDEGDRLTEQELLSIAGVGLLLTGVETVSTHIPNFVYALLT

HPELMAQLRADRSLVPAAVEELLRMIPLNPAAMFPRYAVEDVTLSGFTVR

AGQPVLVSLPGANRDPEVFENPETFDFTRDQNPHVAFGHGPHHCLGAQLA

>CYP125A65(2517969954)*Salinispora pacifica* CNT124

MTEPRIPVGFDFTDPTVLERRVPREEFAELRRTAPVWWNVQPRGSAGFDD

DGYWVVTRYADVMAVSRDSETYSTRENTAIARFQPGTTRADLEMQRVIML

NVDPPEHTKLRAIVSRGFTPRAIHALRGSLAERAERIVRDAAVRGTGDFV

TDVACELPLQAIAELIGVPQHHRRKVFDWSNQLIGYDDPAYGVDPMAAAA

ELLGYAMEMADERQRNPSDDLVTKLVNAQIDGEHLTTDEFGFFVMLLAVA

GNETTRNAITHGMLAFLEHPEQWELFKAERPRSAVEEIIRWATPVNVFQR

TALVDTTLGGQAISAGQRVALFYGSANFDESVFEEPERFDITRSPNPHLG

FGGSGVHFCLGANLARLEIELIFNSIADHMPDIRKVADPQRLRSGWINGI

REMPVRYR

>CYP211C9(2517970085)*Salinispora pacifica* CNT124

VPDIEGLLARLYSARGRQDPYPVYADLHAQAAIAALTPGPGRQRVAAVAV

GYDLIAAVLRDPEWCKQPPPGWRDQEILRILQSSMMFINPPDHGRMRHVF

AGTFTPRRLGALEPVINRVTDELLDRMADAEPGEVDFVAEFAYPLPARVM

AEFIGIPTTELAWYRERVDRVDAFLDVAGKTPERLAAANAAGAELRAFYR

ELLARRRRTPGEDLISGLVEAVDAGGVELTEDELISNLIVLFNASFVTTV

YLLSNGLPVLLAHPEVAAALADNPELAAGAVDEILRLQTPVHLLARAAPR

DTVLGGVSIPQGQNVLLLIAAANRDPAHFPDPDRFDPRRSGPSSLAFGLG

LHYCLGAAVSRLEGRLALPRLLSRFPRLRILEQPVYSGSLFLRGIDKLSV

SPGGRSIRE

>CYP154AJ2(2517970143)*Salinispora pacifica* CNT124

VDDESLFVIDPAGADIHGEGARLRASGPITRVLLPGGVEAWSVTGYHAAR

QVLADARFAKNARQHWPAYVNGDLGPDFPLIAWARMDNMSTADGESHARQ

RRLVAGAFSPQRIAALQPRVERAVARALDELAAQAAERPGEPIDLKERYA

HPVAARVIGELLGVPDGDPDGILDRAGYIESTPQRAAEEFALLRSKIETL
[truncated: 1,009,834 more chars]
