# Supplementary figures and images for: An Unprecedented Number of Cytochrome P450s Are Involved in Secondary Metabolism in Salinispora Species
Source: Microorganisms. 2022 Apr 21;10(5):871. doi: 10.3390/microorganisms10050871 (PMC9143469; doi:10.3390/microorganisms10050871)

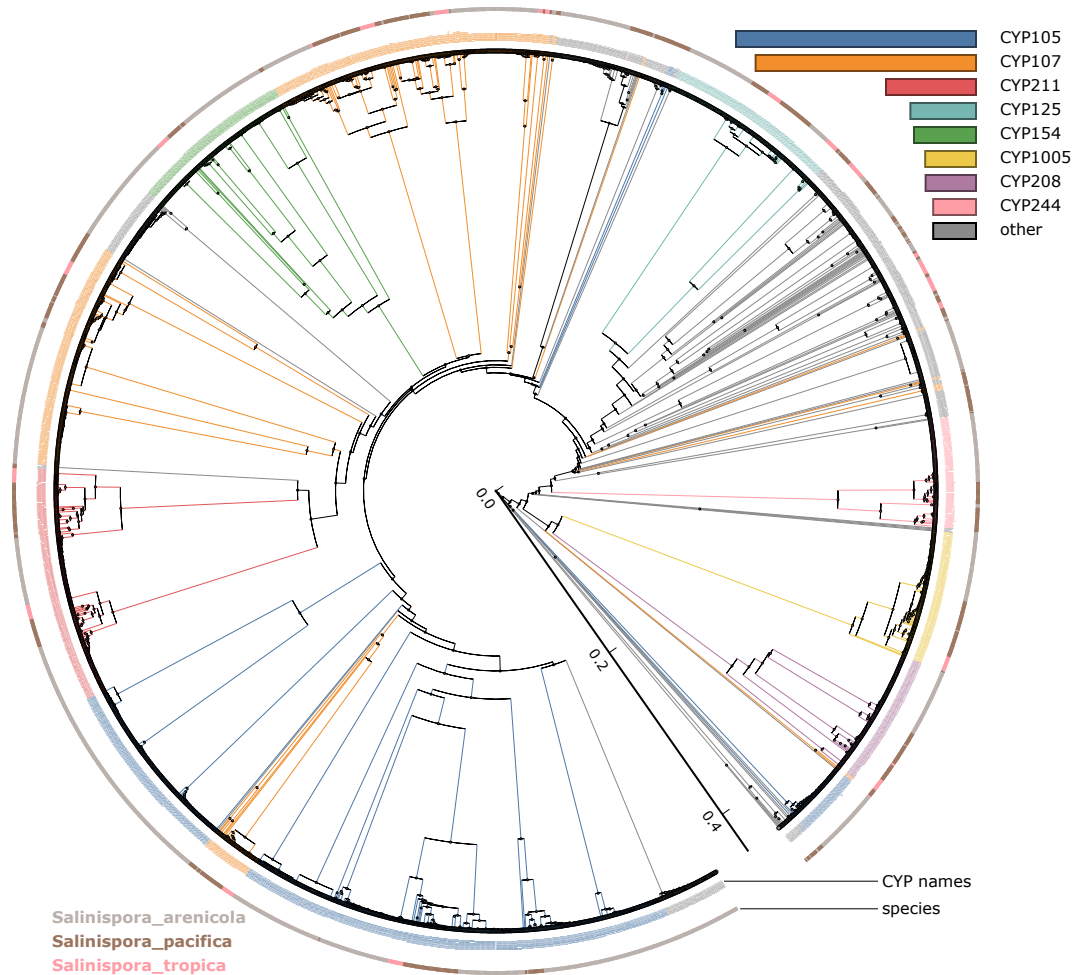

Supplement: Supplementary file 1 [file microorganisms-10-00871-s001.zip › Figure S1.pdf]
